# Multisubstituted pyrimidines effectively inhibit bacterial growth and biofilm formation of *Staphylococcus aureus*

**DOI:** 10.1038/s41598-021-86852-5

**Published:** 2021-04-12

**Authors:** Riccardo Provenzani, Paola San-Martin-Galindo, Ghada Hassan, Ashenafi Legehar, Aleksi Kallio, Henri Xhaard, Adyary Fallarero, Jari Yli-Kauhaluoma

**Affiliations:** 1 Drug Research Program, Division of Pharmaceutical Chemistry and Technology, Faculty of Pharmacy, University of Helsinki, P.O. Box 56, (Viikinkaari 5 E), FI-00014 Helsinki, Finland; 2 Drug Research Program, Division of Pharmaceutical Biosciences, Faculty of Pharmacy, University of Helsinki, P.O. Box 56, (Viikinkaari 5 E), FI-00014 Helsinki, Finland

**Keywords:** Antibiotics, Drug discovery and development, Screening, Medicinal chemistry, Chemical synthesis, Drug screening, Medicinal chemistry, Antimicrobials, Biofilms, Drug discovery, Microbiology, Chemistry

## Abstract

Biofilms are multicellular communities of microorganisms that generally attach to surfaces in a self-produced matrix. Unlike planktonic cells, biofilms can withstand conventional antibiotics, causing significant challenges in the healthcare system. Currently, new chemical entities are urgently needed to develop novel anti-biofilm agents. In this study, we designed and synthesized a set of 2,4,5,6-tetrasubstituted pyrimidines and assessed their antibacterial activity against planktonic cells and biofilms formed by *Staphylococcus aureus*. Compounds **9e**, **10d**, and **10e** displayed potent activity for inhibiting the onset of biofilm formation as well as for killing pre-formed biofilms of *S. aureus* ATCC 25923 and Newman strains, with half-maximal inhibitory concentration (IC_50_) values ranging from 11.6 to 62.0 µM. These pyrimidines, at 100 µM, not only decreased the number of viable bacteria within the pre-formed biofilm by 2–3 log_10_ but also reduced the amount of total biomass by 30–50%. Furthermore, these compounds were effective against planktonic cells with minimum inhibitory concentration (MIC) values lower than 60 µM for both staphylococcal strains. Compound **10d** inhibited the growth of *S. aureus* ATCC 25923 in a concentration-dependent manner and displayed a bactericidal anti-staphylococcal activity. Taken together, our study highlights the value of multisubstituted pyrimidines to develop novel anti-biofilm agents.

## Introduction

Antimicrobial resistance is a recognized growing global threat that can affect the life of anyone at any age and currently it accounts for 700,000 deaths every year^1^. The number is estimated to rise to 10 million by 2050 and will be accompanied by an economic loss of about 100 trillion USD during the same period. Antibiotics are vital in treating bacterial infections, in preventing post-surgery infections, and they are also used in combination with cancer treatments^2^.

Bacteria can adhere to surfaces in a self-produced extracellular matrix to form multicellular structured communities known as biofilms. This allows them to tolerate chemical and environmental stresses^3^. Compared to the planktonic lifestyle, the biofilm state is challenging to counteract since it can withstand 10–1000 times higher doses of conventional antibiotics. Approximately 80% of nosocomial infections are biofilm-related and many of them are caused by the Gram-positive bacterium *S. aureus*^4,5^. This bacterium also causes respiratory tract and skin-related infections as well as food contamination^6^. Currently, there are no approved antimicrobial drugs specifically targeting biofilm infections^7^. Therefore, there is an urgent need for drugs that can either interfere with the biofilm formation at early stages or disrupt already established biofilms.

Since the 1980s, antibiotic development has strongly declined due to low economic incentives, low profit potential, and challenging regulatory requirements^2,8^. Meanwhile, current antibiotics are losing their efficacy due to the rising of antibiotic resistance to a dangerously high level. Therefore, new antibiotics that can antagonize multidrug-resistant bacteria are highly needed. During the past 20 years, pharmaceutical companies and academic research groups have mainly developed new antibiotic analogs by modifying the molecular scaffolds of existing drugs^9^. In fact, only four novel classes of antibiotics have entered the market during this period: oxazolidinones, ketolides, glycylcyclines and lipoglycopeptides^9,10^. With the current chemotherapeutic portfolio, the number of chemical modifications applicable to existing antibiotics are limited and they may prolong the efficacy of the known classes for up to a decade^11^. Hence, there is a need for new classes of antibiotics acting with different mechanisms and/or via new target sites.

Nitrogen-containing heterocyclic compounds are of great significance as they abound in nature and synthetic/semisynthetic derivatives exhibit a broad spectrum of biological activities^12^. Pyrimidines are heterocyclic aromatic compounds that are present in many natural and synthetic products and constitute essential building blocks for DNA and RNA biosynthesis^13^. Pyrimidine derivatives have shown a wide spectrum of biological activities including both non-chemotherapeutic, such as cardiovascular^14^ and anti-inflammatory^15^, as well as chemotherapeutic, such as anticancer^16^, antiviral^17^, and antibacterial^18,19^. In our previous work, we prepared a set of multisubstituted pyrimidines designed to target the C1 domain of protein kinase C^20^. However, a preliminary screening of these derivatives highlighted a potential antibacterial activity. Here, we report the design and synthesis of novel tetrasubstituted pyrimidines and their in vitro antimicrobial activity against planktonic and biofilm cells of *S. aureus*.

## Results and discussion

### Antimicrobial screening, design, synthesis, and structure–activity relationship analysis

We initially assessed a library of nineteen multisubstituted pyrimidines for antimicrobial activity against planktonic cells as well as biofilms formed by *S. aureus* ATCC 25923 at the high concentration of 400 µM (all structures and biological data available in Supplementary Table S1). Of this library, seventeen compounds were already available, and their syntheses were previously reported by us^20^, while compounds **9a** and **10d** were prepared following the synthesis described below. Compound **10d** was the most potent as it successfully inhibited the planktonic cells and biofilm viability by ≥ 90%.

**Figure 1:**
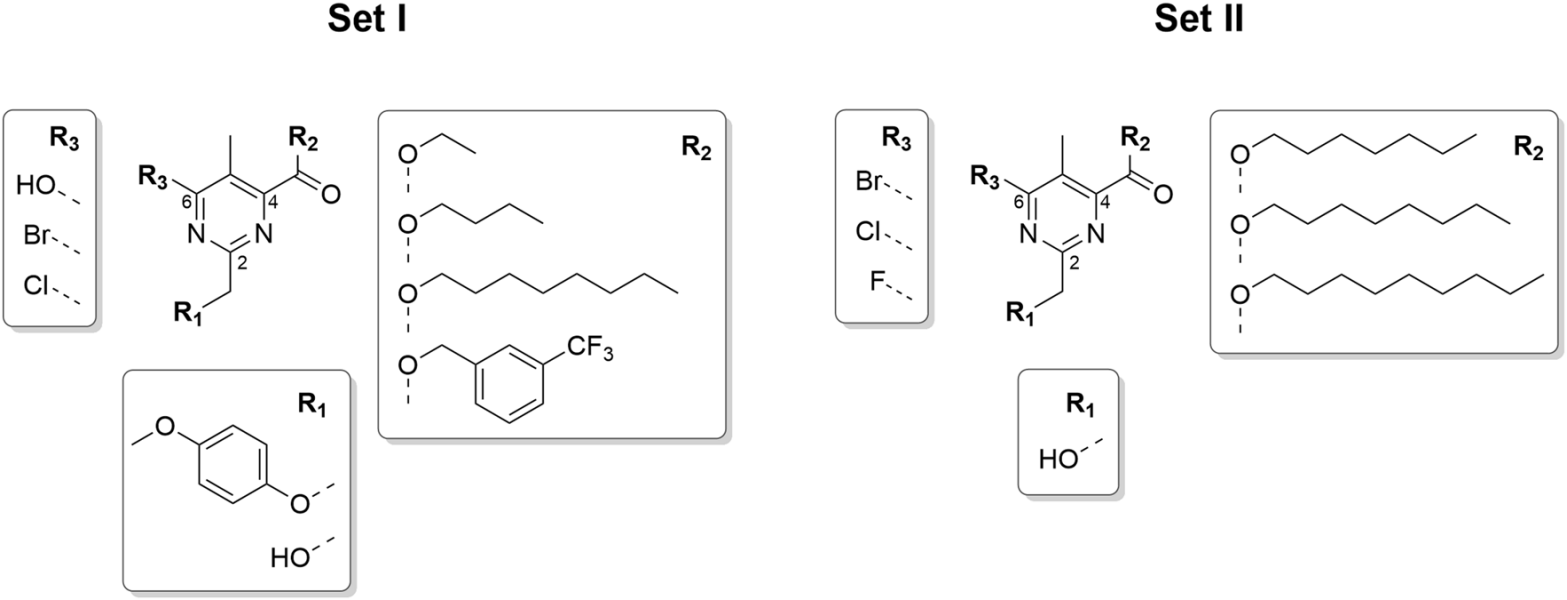
Sets I and II of the designed 2,4,5,6-tetrasubstituted pyrimidines with the scaffold and substituents.

Based on this primary screening, we designed a first set of tetrasubstituted pyrimidines bearing: (1) a free or *para*-methoxyphenyl (PMP)–protected hydroxymethyl moiety in position C2, (2) an ester functionality in position C4 bearing linear alkyl substituents of different length (i.e. 2, 4, or 8 carbons) or a 3-(trifluoromethyl)benzyl substituent in C4, and (3) a phenolic hydroxy group, chloride or bromide in position C6 (Fig. 1, Set I). After completing the biological evaluation, we then fine-tuned a smaller set of derivatives focusing on the presence of (1) only the free hydroxymethyl moiety in C2, (2) an 1-heptyl or 1-nonyl ester in C4, and (3) also a fluoride in position C6 (Fig. 1, Set II).

**Figure 2:**
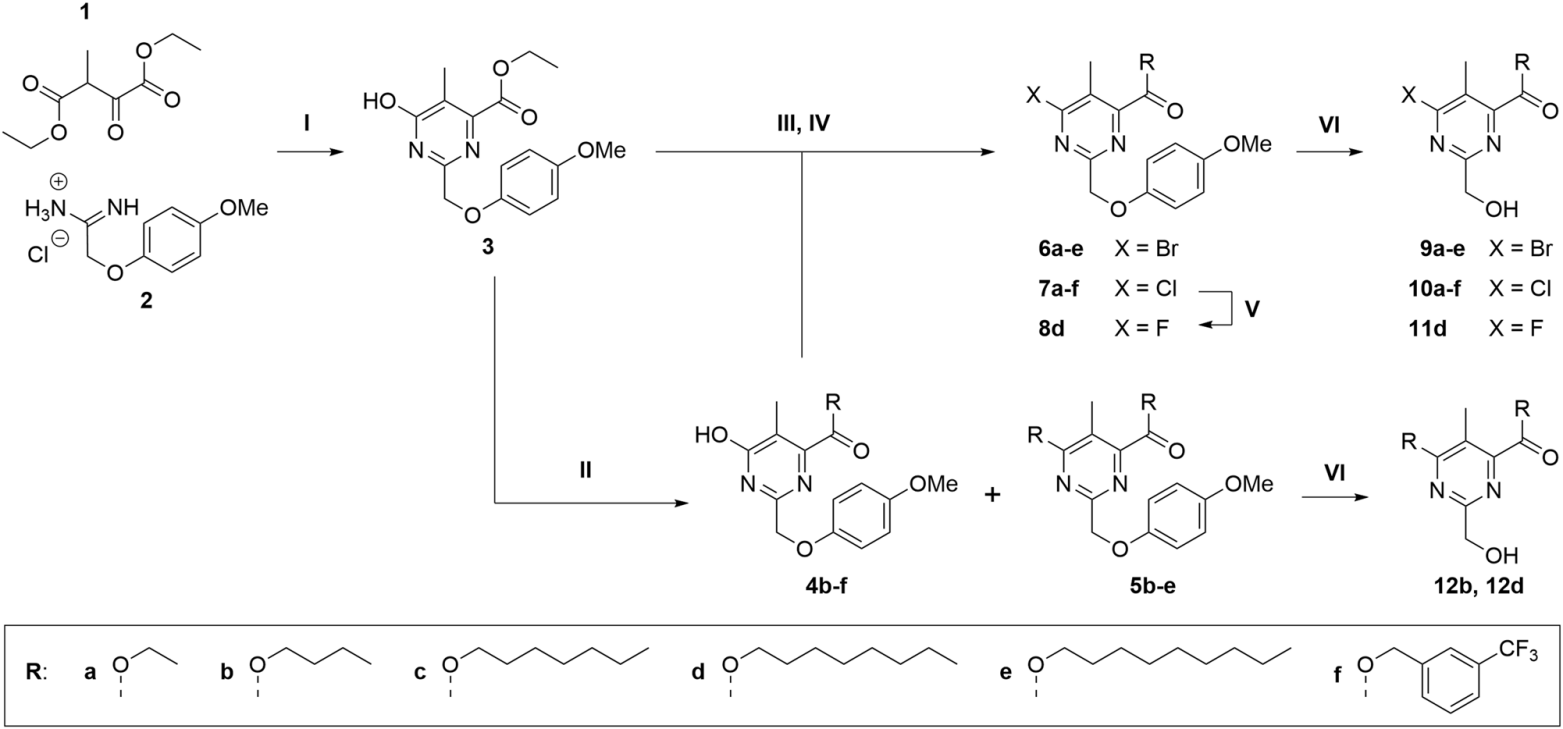
Synthesis and derivatization routes of sets I and II of 2,4,5,6-tetrasubstituted pyrimidines. Conditions: (I) TEA, EtOH, reflux, 2.5 h, 31%; (II) alcohol, H_2_SO_4_ (cat.), 100 °C, 3–48 h, 18–58%; (III) POBr_3_, DMF, MW 90 °C, 10–15 min, 41%–quant.; (IV) POCl_3_, DMF, MW 90 °C, 10–15 min, 72%–quant.; (V) KF, TBAB, sulfolane, MW 150 °C, 2 h, 35%; (VI) CAN, MeCN/H_2_O, − 15 °C, 10 min, 12–75%.

All synthesized compounds presented in this study are grouped in the synthesis routes of Fig. 2. As previously reported^20^, we reacted the commercially available diethyl oxalpropionate (**1**) and 2-(4-methoxyphenoxy)acetamidine hydrochloride (**2**) in the presence of triethylamine (TEA) in absolute ethanol to obtain the pyrimidine **3** containing the PMP-protected hydroxymethyl moiety in position C2 (Fig. 2). The PMP protection is suitable to withstand the subsequent acid-catalyzed transesterifications of **3** in presence of the selected alcohols such as 1-butanol, 1-heptanol, 1-octanol, 1-nonanol, or 3-(trifluoromethyl)benzyl alcohol to give the derivatives **4b**–**f**. Except for the 3-(trifluoromethyl)benzyl alcohol, the other alcohols reacted also in position C6, yielding enough disubstituted derivatives **5b**–**e** to be isolated for both testing and subsequent transformations. To explore the possible roles of different halogens on C6, compounds **3** and **4b**–**f** were reacted with phosphoryl bromide or phosphoryl chloride in *N*,*N*-dimethylformamide (DMF) to give the corresponding halogenated derivatives **6a**–**e** and **7a**–**f**, respectively. The bromination of **4f** was not successful in the described conditions (Fig. 2, III). The chloride group of **7e** was substituted with a fluoride when stirred with KF and *n*-tetrabutylammonium bromide in sulfolane as reported by Floersheimer and coworkers^21^. To remove the PMP protecting group, the intermediates **6a**–**e**, **7a**–**f**, **8d**, **5b**, and **5d** were treated with ceric ammonium nitrate (CAN) in MeCN/H_2_O to yield the final compounds **9a**–**e**, **10a**–**f**, **11d**, **12b**, and **12d**, respectively.

To screen the biological activity of the first set of 21 pyrimidines, we determined their activity at 400 µM against planktonic cells and biofilm formed by *S. aureus* ATCC 25923, with azithromycin as the control antibiotic in all viability assays (Supplementary Table S2). Compounds **9d** and **12b** exhibited the best activity as they caused over 80% inhibition of the viability of planktonic cells and over 81% of the biofilm formation of *S. aureus* ATCC 25923. The preliminary structure–activity relationship (SAR) analysis suggested that the PMP group did not contribute to the antimicrobial activity as all PMP-containing derivatives were inactive. Except for the C4–C6 dialkyl-substituted **12b**, compounds bearing the ethyl, 1-butyl or 3-(trifluoromethyl)benzyl ester in C4 also lacked activity. The bromide-containing **9d** exhibited slightly lower activity at preventing biofilm formation compared to the chloride-containing **10d**. The halogen-related decrease in activity may be due to differences in steric properties or electron-withdrawing properties, which can affect the overall electron distribution in the conjugated ring system.

We explored further the SAR by synthesizing and testing a second set of derivatives comprising five additional compounds: **9c**, **9e**, **10c**, **10d**, and **11d** (Table 1). As the most active **10d** contains a 1-octyl ester, we introduced either 1-heptyl (**10c**, **9c**) or 1-nonyl (**10e**, **9e**) ester groups in position C4 to investigate the effect of ± 1 carbon on the alkyl substituent. We also performed a chloride–fluoride replacement (**11d**) to investigate the effect of a smaller and more electronegative halogen in C6. In addition, to evaluate possible strain-specific activity, we tested the set II (**9c**, **9e**, **10c**, **10d**, and **11d**) and the most active compounds of set I (**9d** and **12b**) at a lower concentration of 100 µM against planktonic cells and biofilms formed by *S. aureus* ATCC 25923 and Newman (Table 1).

**Table 1:** Antimicrobial activity of the second set of 2,4,5,6-tetrasubstituted pyrimidines, including the most active compounds identified in the previous screenings against *S. aureus* ATCC 25923 and Newman clinical strains.

Cmpd	*Staphylococcus aureus* ATCC 25923				*Staphylococcus aureus* Newman	
	Planktonic phase		Biofilm pre-exposure		Biofilm pre-exposure	
	Mean	± SD	Mean	± SD	Mean	± SD
**Inhibition percentage of bacterial viability (%)**						
9c	1.42	12.44	8.58	5.32	49.32	7.48
9d	58.22	15.34	51.77	3.62	− 3.12	3.40
9e	94.34	2.11	98.07	0.09	99.78	0.11
10c	− 3.35	2.31	6.32	3.32	− 10.56	7.00
10d	92.51	4.98	97.85	2.25	97.85	3.00
10e	92.37	3.72	98.89	0.34	99.94	0.21
11d	35.49	15.06	45.14	6.11	− 0.67	4.64
12b	8.32	3.74	2.07	3.62	9.09	0.42
Azm*	99.58	0.09	96.97	0.44	99.26	0.05

All compounds were tested at 100 µM.

Results are expressed as the mean value ± standard deviation (SD) for three parallel replicates and two biological repetitions.

*Azithromycin (azm), the antibiotic used as the positive control, was tested at 400 µM.

The most active compounds, **9e**, **10d**, and **10e**, inhibited the biofilm viability of both *S. aureus* ATCC 25923 and Newman strains by over 98%. These three compounds also displayed antimicrobial activity against planktonic cells of *S. aureus* ATCC 25923 causing over 92% inhibition of viability at 100 µM. The fluoride in C6 did not improve the antimicrobial activity as **11d** displayed a marginal inhibition compared to the chloride-containing **10d**. The length of the alkyl ester in C4 deeply influenced the activity starting from eight carbons and shorter substituents caused loss of activity. Of note, compounds **9e** and **10e** displayed comparable results against biofilm and planktonic cells and it seems that the presence of the 1-nonyl ester chain contributed more to their activity independently of the nature of the halogen in C6. However, this was not the case for the 1-octyl ester-containing derivatives **9d** and **10d**. At this concentration (100 µM), the presence of the bromide in **9d** reduced the overall activity but also highlighted a strain specificity compared to the chloride-containing **10d**.

### Antibacterial effect of 9e, 10d, and 10e on planktonic cells

To determine the antibacterial effects on suspended cells, we measured the minimal inhibitory concentration (MIC) and the minimal bactericidal concentration (MBC) values of the most active pyrimidines **9e**, **10d**, and **10e** (Table 2). Interestingly, they exhibited equal MIC values in both staphylococcal strains and **9e** prevailed as the most active antibacterial derivative with a MIC value of 40 µM. Comparing the MBC/MIC ratios, the three compounds displayed an equal ratio of 2 against *S. aureus* ATCC 25923, whereas a ratio of 1.5 against *S. aureus* Newman. This suggests that **9e**, **10d**, and **10e** possess a bactericidal anti-staphylococcal behavior^22^.

**Table 2:** Antibacterial and anti-biofilm activity of the most active compounds against *S. aureus* ATCC 25923 and *S. aureus* Newman strains.

Cmpd	Planktonic bacteria		MBIC µM [mg/L]	Anti-biofilm activity—IC_50_ (µM)^a^		Log R^b^ log_10_ (CFU/mL)
	MIC µM [mg/L]	MBC µM [mg/L]		Pre-exposure	Post-exposure	
***Staphylococcus aureus*** **ATCC 25923**						
**9e**	40 [14.9]	80 [29.9]	40 [14.9]	11.6 (9.9–13.3)	23.3 (16.5–37.3)	2.3
**10d**	60 [18.9]	120 [37.8]	60 [18.9]	23.6 (19.6–28.9)	54.9 (50.8–59.1)	2.8
**10e**	60 [19.7]	120 [39.5]	60 [19.7]	16.8 (14.4–20.1)	51.8 (48.3–55.7)	2.6
***Staphylococcus aureus*** **Newman**						
**9e**	40 [14.9]	60 [22.4]	40 [14.9]	29.4 (27.8–30.5)	31.9 (26.0–38.0)	1.8
**10d**	60 [18.9]	90 [28.3]	60 [18.9]	38.4 (36.6–41.2)	62.0 (57.8–74.4)	3.6
**10e**	60 [19.7]	90 [29.6]	60 [19.7]	37.3 (33.9–43.4)	44.8 (38.5–53.8)	2.3

^a^The 95% confidence interval are shown in parentheses.

^b^Logarithm of reduction of biofilm burden on a pregrown biofilm. Compounds were tested at 100 µM.

**Figure 3:**
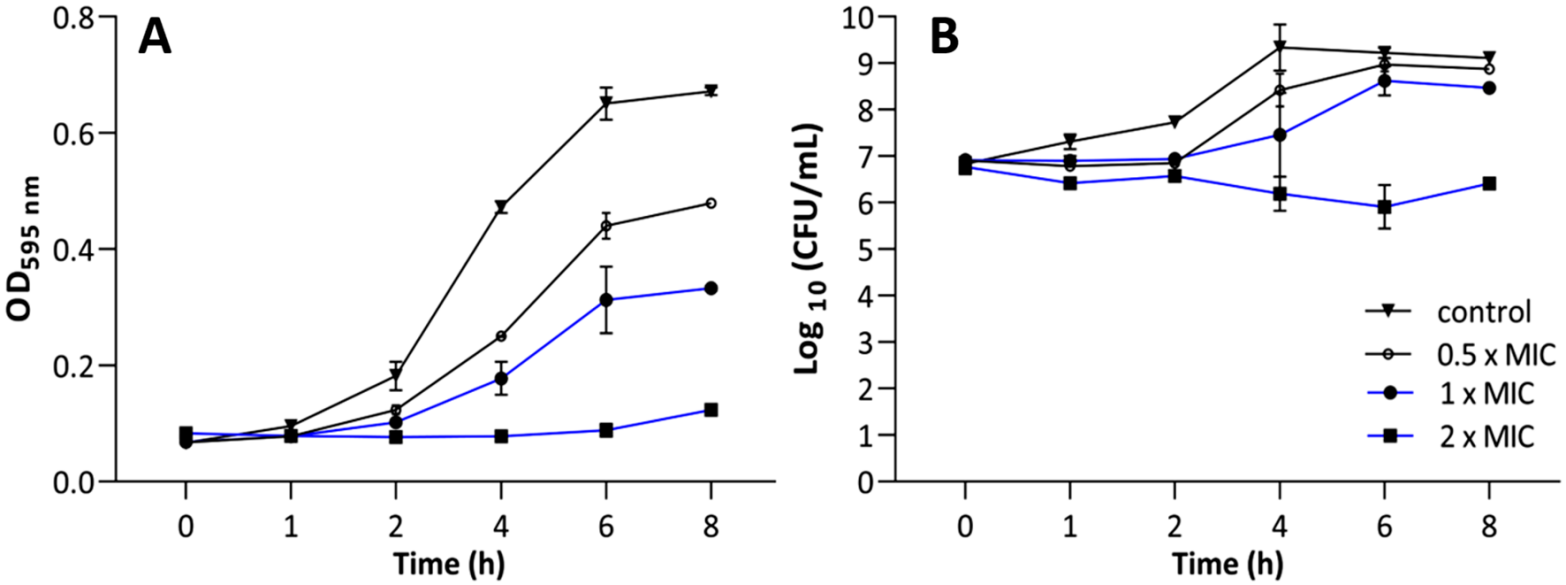
Time-kill assay of compound **10d** for *S. aureus* ATCC 25923 during an 8-h incubation period represented by the bacterial growth curve (**A**) and number of CFUs (**B**). Data represent the mean of two biological repetitions with two technical replicates each ± SEM.

To further investigate the bactericidal activity rate of these pyrimidines, we performed a time-killing assay using **10d** against *S. aureus* ATCC 25923. We treated the cells with three concentrations (0.5, 1, and 2 times the MIC value) within 8 h. The time-kill curve shows a concentration-dependent bacterial death (Fig. 3A-B). No differences emerged between treated and non-treated cells during the first 2 h, neither in optical density (OD) nor in number of colony-forming units (CFUs). After a 4-h exposure, **10d** exhibited a concentration-dependent activity. The OD curves of **10d** at 0.5 × MIC and 1 × MIC displayed lower OD values compared to the bacterial control; however, they still represent a bacterial growth pattern (4–8 h). The OD curve of **10d** at 2 × MIC, instead, represents a maintained bacterial suspension during the tested time, which indicates an effective bacterial growth inhibition (Fig. 3A). Similarly, **10d** at 2 × MIC inhibited the bacterial growth of the initial inoculum as well as reduced the CFUs by over 2 log_10_ from 4 h of exposure (Fig. 3B).

### Anti-biofilm effect of 9e, 10d, and 10e

We explored the activity of **9e**, **10d**, and **10e** against *S. aureus* biofilms in both pre- and post-exposure inhibition assays by measuring the biofilm viability (Table 2). Of note, the minimal biofilm inhibitory concentration (MBIC) values of the pyrimidines equal their MIC values. For instance, at the MIC, **10d** also inhibited by 90% the biofilm formation of both *S. aureus* strains in terms of viability (Table 2). Furthermore, the three pyrimidines exhibited MBIC values lower than their corresponding bactericidal (MBC) values.

Comparing the anti-biofilm potencies in terms of half maximal inhibitory concentration (IC_50_), a twofold higher concentration of **9e** and **10d** reduced the viability of pre-formed *S. aureus* ATCC 25923 biofilms by 50%, compared to the concentration needed to prevent the biofilm formation (Table 2). Biofilms can typically withstand 10–1000 times higher concentrations of conventional antibiotics compared to planktonic cells. Despite this, **9e**, **10d**, and **10e** required only twice their MIC to effectively reduce by > 91% the viability of pre-formed biofilms. We measured inhibition potencies, in terms of biofilms viability, below 62 µM for both tested staphylococcal strains. The bacterial growth states can dynamically switch between planktonic and biofilm stages depending on their nature and complex external driving forces^23,24^. Since our compounds act against both microbial states at relatively similar concentrations, they hold promise and could be further investigated as antimicrobial candidates.

To confirm the efficacy of these three compounds against pre-formed biofilms, we calculated the reduction of viable cells within the biofilm and measured the log reduction (log R) after a 24-h exposure at 100 µM (Table 2). Viable cell counts of the untreated control in the post-exposure assay were 2.4 × 10^8^ CFU/mL and 2 × 10^8^ CFU/mL for *S. aureus* ATCC 25923 and Newman, respectively. Compounds **9e** and **10e** reduced close to and higher than 2-log (> 99%) the number of viable cell counts on pre-formed *S. aureus* biofilms. Compound **10d** caused close to or more than a 3-log reduction in the viability of pre-formed *S. aureus* biofilms. Of note, a 3-log reduction corresponds to a killing efficacy of at least 99.9% of the viable biofilms. In addition, the viable cell density after treatment in the post-exposure phase suggested that the three compounds maintained a bacterial density of the planktonic suspension close to or more than 1 × 10^6^ CFU/mL (Supplementary Fig. S1) in both staphylococcal strains. This demonstrates that these pyrimidines reduce the cell viability of established biofilms without considerably killing planktonic cells, and thus, this confirms their effect as potent anti-biofilm agents. Taken together, **10d** emerged as the most potent anti-biofilm agent with the highest measured efficacy.

To investigate the effect of the new pyrimidines on Gram-negative biofilm-forming strains, we quantified the viable burden of two *Pseudomonas aeruginosa* strains (ATCC 9027 and ATCC 15442) after a 24-h exposure to **9e**, **10d**, and **10e** at 400 µM (Supplementary Fig. S2). None of the compounds showed significant inhibition in the number of viable cell count against either *P. aeruginosa* strain, thus we concluded that these tetrasubstituted pyrimidines preferably act against Gram-positive bacterial biofilms.

### Effect of 9e, 10d, and 10e on the biofilm biomass

**Figure 4:**
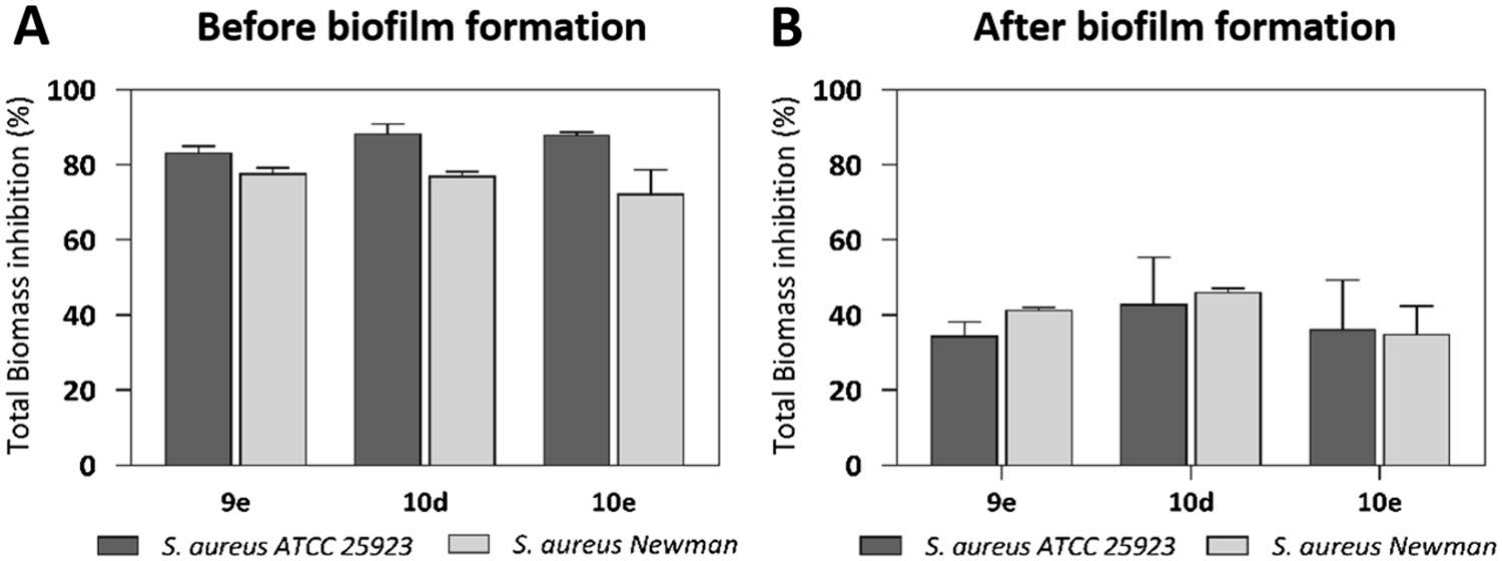
Anti-biofilm activities of **9e**, **10d**, and **10e** at 100 µM in the pre- (**A**) and post-exposure (**B**) assays. The total biomass was quantified using the crystal violet staining. Data represent the mean of two independent repetitions with three technical replicates each ± SEM.

Once we confirmed the effectivity of these pyrimidines to reduce viable bacteria within the biofilm, we assessed the impact of **9e**, **10d**, and **10e** at 100 µM on the biofilm biomass by performing a crystal violet assay. The three pyrimidines inhibited the biofilm biomass of *S. aureus* ATCC 25923 and Newman by 80% and 70%, respectively, compared to the untreated control, after an 18-h exposure (Fig. 4A). This suggests that the compounds prevent the biofilm formation not only in terms of viability (as shown in Table 1) but also in terms of total biomass. In addition, the compounds reduced the total biofilm biomass of established biofilm by 30–50%, with **10d** showing the highest efficacy among them (Fig. 4B). Comparing the outcomes in pre-formed biofilms, the percentage of inhibition of the total biomass differed from the biofilm viability (which resulted in a > 90% reduction; Table 2). As the crystal violet stains both extracellular matrix and live/dead cells^25^, we next investigated specifically the proportion of live and dead cells in the biofilms.

**Figure 5:**
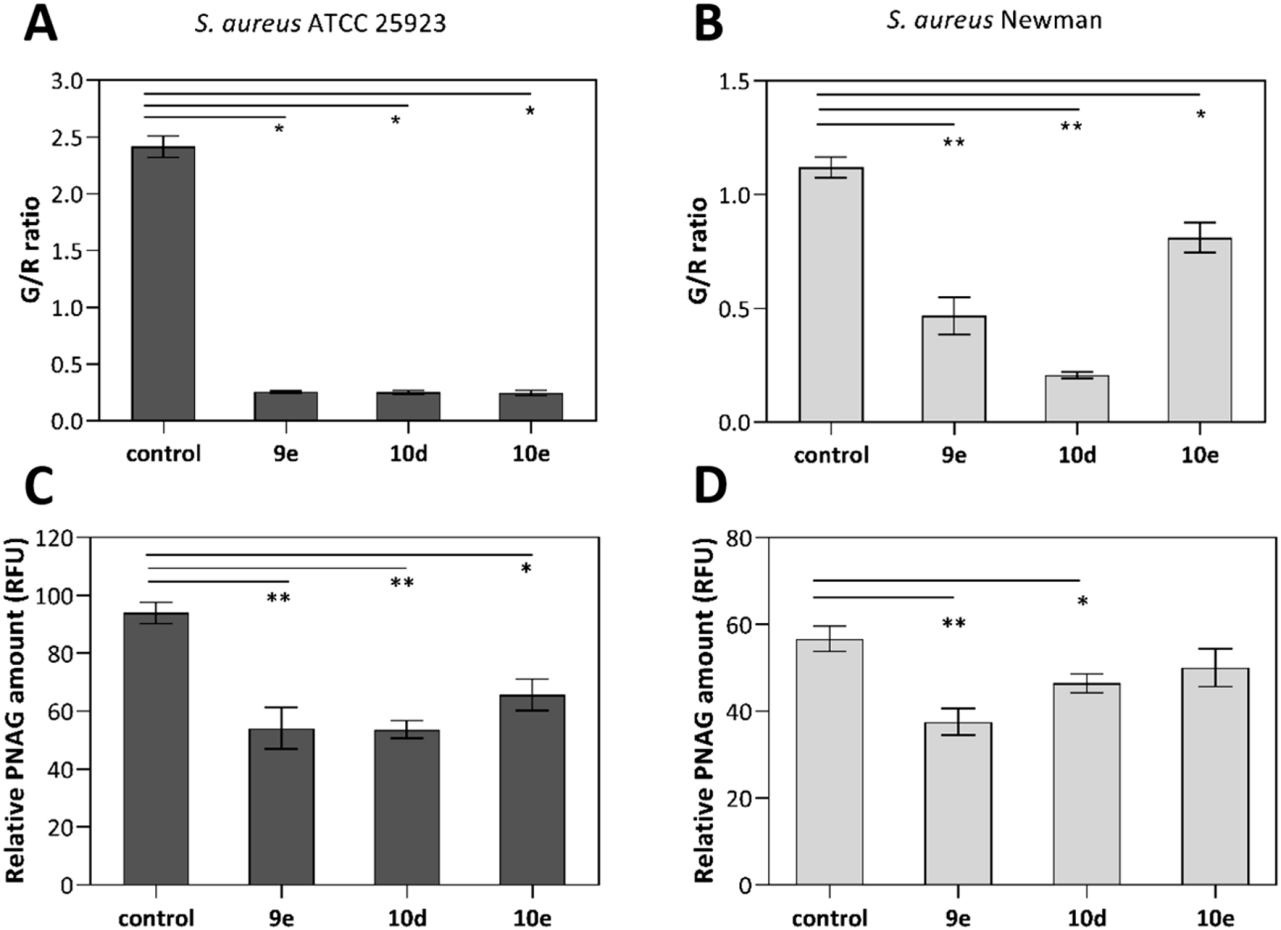
Effect of the pyrimidines **9e**, **10d,** and **10e** at 100 µM after a 24-h incubation with pre-formed biofilms *of S. aureus* ATCC 25923 (**A** and **C**) and *S. aureus* Newman (**B** and **D**). Quantification of the green-to-red-fluorescence ratios (G/R), using SYTO 9 and PI fluorescent nucleic acid stains (**A** and **B**), and quantification of the poly-*N*-acetylglucosamine (PNAG) content (**C** and **D**) in terms of relative fluorescence units (RFU) measured by the wheat germ agglutinin Alexa Fluor 488 (WGA) staining. An unpaired *t*-test with Welch’s correction was used to determine statistical differences (*p < 0.05; **p < 0.01). Data represent the mean of two independent repetitions with at least two technical replicates each ± SEM.

**Figure 6:**
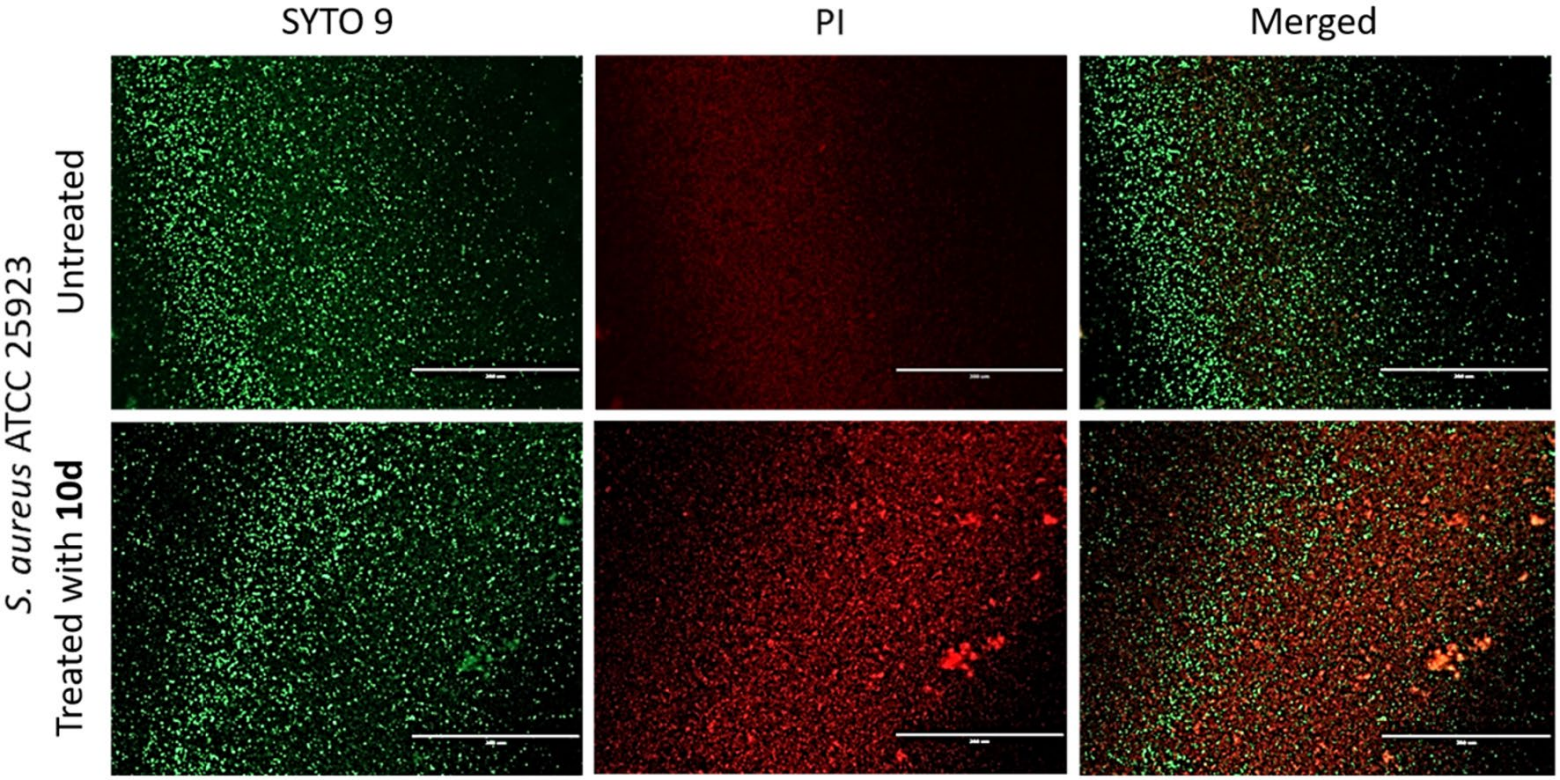
Fluorescence microscopy images of 18-h old *S. aureus* ATCC 25923 biofilms in a 96-well plate exposed to **10d** at 100 µM for 24 h. Representative images of biofilms stained with LIVE/DEAD BacLight kit are displayed. Scale bars correspond to 200 µm.

By applying the live/dead fluorescence kit, SYTO 9/PI, we gained insights into the physiological state of biofilm cells upon exposure to the pyrimidines as it quantifies viable and dead bacteria differentiated by their membrane integrity^26^. The fluorescent nucleic acid dyes, SYTO 9 and PI, penetrate bacterial cells differently. While the former labels all bacteria (green fluorescence), the latter labels only those with a damaged membrane (red fluorescence). We measured the green-to-red (G/R) fluorescence ratio after a 24-h exposure of pre-formed staphylococcal biofilms with **9e**, **10d**, and **10e** at 100 µM. The two strains of untreated *S. aureus* biofilms displayed different G/R ratios. In untreated biofilms, *S. aureus* ATCC 25923 live cells predominated over dead cells (2.4 ratio; Fig. 5A) while the assay on *S. aureus* Newman yielded a more equal distribution of viable and non-viable bacteria (1.1 ratio; Fig. 5B). Our pyrimidines remarkably damaged bacterial cells of established biofilms as **9e**, **10d**, and **10e** decreased the G/R ratio to roughly 0.25 and 0.21–0.81 in *S. aureus* ATCC 25923 and Newman strains, respectively (Fig. 5A,B). Particularly, **10d** showed the lowest G/R ratios (0.25 and 0.21) in both staphylococcal strains and we visualized its efficacy to decrease viable biofilm cells of *S. aureus* ATCC 25923 by fluorescence imaging (Fig. 6). Altogether, these results support the anti-biofilm activity of the pyrimidines, at a low concentration of 100 µM, to disturb pre-formed biofilm.

To further characterize the activity of **9e**, **10d**, and **10e** on the biomass of existing biofilms, we assessed their effect on the exopolysaccharide poly-*N*-acetylglucosamine (PNAG). PNAG, a positively charged polymer, is the major component of the extracellular polysaccharide substance (EPS) in *S. aureus* biofilms which is mediated by the expression of *icaADBC*-encoded enzymes^27^. Since the production of PNAG influences the cell-to-cell adhesion as well as the biofilm structure and integrity, PNAG is an attractive target to counteract biofilm-associated infections^28^. Here we quantified the bound PNAG residues in the EPS by using the wheat germ agglutinin-Alexa Fluor 488 fluorescent conjugate (WGA)^29^. In both *S. aureus* strains, **9e** and **10d** significantly inhibited the PNAG content of the EPS (Fig. 5C,D), and particularly, they reduced by nearly 50% the relative PNAG content of *S. aureus* ATCC 25923 biofilms in comparison to untreated biofilms. The PNAG content of *S. aureus* Newman biofilms, instead, were less susceptible to the pyrimidines as **9e** and **10d** decreased the WGA signal by about 40% and 23%, respectively. Compound **10e** showed no significant inhibition. These results corroborate the effect of **9e** and **10d** to disturb *S. aureus* biofilms as they act in existing biofilms not only by killing cells within the biofilms but also by inhibiting the biomatrix production (in terms of PNAG content), pointing to a significant fitness as anti-biofilm agents.

### Cytotoxicity of 9e, 10d, and 10e on human cells

We measured the in vitro cytotoxicity of these three most active pyrimidines against human Hep2 cell line, derived from epidermoid carcinoma cells, as an initial evaluation of unspecific toxicity. This is a common concern in the development of antimicrobials. Compounds **9e**, **10d**, and **10e** exhibited cytotoxic activities when tested at the highest concentration of 100 µM (see Supplementary Table S3). Compounds **9e** and **10e** maintained over 73% of cell viability when tested at 40 µM, which corresponds to the MIC of **9e** and it is a concentration relatively higher than their determined potencies (IC_50_s) to prevent the biofilm formation of *S. aureus* strains (Table 2). In contrast, **10d** prompted a slightly higher unspecific cytotoxicity, under the same exposure conditions, as 55% of the cells remained viable.

### Follow-up computational studies with 9e, 10d and 10e

To assess the novelty of compounds **9e**, **10d**, and **10e**, we collected a dataset from ChEMBLv27^30,31^ containing compound-bioactivity data for Gram-positive bacteria (n = 21,922 compounds; n = 2846 assays; n = 56,670 MIC data points). The histogram distribution of published log (MIC) showed that the activities of **9e**, **10d**, and **10e** are average (Supplementary Fig. S3). We focused on identifying bioactive compounds sharing substructure or global similarity to our compounds. Neither close analogs (Tanimoto > 0.7) nor pyrimidine-based compounds emerged, which supports the chemical novelty of **9e**, **10d**, and **10e**. Among the distant active hits, several series contain an alkyl tail and an aromatic or nitrogen-containing ring, similar to our compounds. To note, the structure–activity relationships of compounds presented by Yarinich and coworkers hints to the optimal length of the alkyl tail containing at least seven carbons. This structural similarity extends our knowledge about antibacterial chemical frameworks and supports the findings presented in this manuscript. Considering the mode of action of some compounds extracted from the database, CHEMBL4166911 and CHEMBL3410371 (Supplementary Fig. S4) seem to exert their activity through interaction with the membrane^32,33^, and CHEMBL3417347, although it exhibits low intrinsic antibacterial activity, it seems to inhibit the efflux transporter NorA^34^. It appears that the mode of action of the molecular framework is not understood fully yet.

## Conclusion

In this study, we designed and synthesized a novel set of 2,4,5,6-tetrasubstituted pyrimidines and highlighted the value of a suitable scaffold to develop antibacterial/anti-biofilm agents against Gram-positive staphylococcal strains. We identified three active compounds (**9e**, **10d**, and **10e**), prepared via a four-step synthesis. These compounds inhibited the biofilm formation as well as disrupted the pre-formed biofilms of two strains of *S. aureus* (ATCC 25923 and Newman) in the low micromolar range. Furthermore, **10d** effectively reduced the viability of pre-formed *S. aureus* Newman biofilm by 99.9% when tested at 100 µM (31.48 mg/L). We confirmed that these pyrimidines not only decreased the viability of cells within the *S. aureus* biofilms but also decreased the biofilm biomass, at least partially by reducing the PNAG content. We observed no significant reduction of viable cells of *P. aeruginosa* strains, thus suggesting that these compounds may act specifically against Gram-positive bacteria. Additionally, these pyrimidines displayed only marginal cytotoxicity on Hep2 cells at concentrations comparable to their corresponding pre-exposure IC_50_values on *S. aureus* biofilm. Our findings enhance the promising role of multisubstituted pyrimidines in the research field of new anti-biofilm agents.

## Methods

### Synthesis procedures

General information and experimental procedures of all synthesized compounds are available in Supplementary Information. All NMR spectra are available in Supplementary NMR Appendix.

### Bacterial strains and growth conditions

Biofilm-forming clinical strains of *S. aureus* (ATCC 25923 and Newman) and *P. aeruginosa* (ATCC 15442 and ATCC 9027) were provided by the Faculty of Pharmacy, University of Helsinki, Finland. Bacterial strains were stored as cryogenic stocks (− 80 °C) and were routinely propagated on either trypticase soy agar (TSA, Neogen) for *S. aureus* strains or Miller’s Luria-Bertani agar (LBA, Fisher BioReagents) for *P. aeruginosa* strains. The bacterial cultures conditions are described in Supplementary Information.

### Anti-biofilm and antibacterial assays

Biofilms were formed in flat-bottomed 96-well plate (Nunclon D Surface) by adding 200 µL of *S. aureus* bacterial suspension (bacterial culture diluted to obtain 10^6^ CFU/mL) per well, and plates were incubated under aerobic conditions for 18 h (37 °C, 200 rpm)^35^. The anti-biofilm activity of the compounds was primarily assessed against *S. aureus* strains (ATCC 25923 and Newman) under two modes of exposure (pre- and post-exposure), as previously described^36,37^. The compounds were initially tested at 400 µM final concentration and further at 100 µM. TSB and DMSO 10% (v/v) in TSB were included as the control solvents throughout the experiments, as earlier reported by us^38^. Azithromycin at 400 µM was used as the viability control antibiotic. The bacterial viability of both planktonic phase and biofilms were quantified using the redox dye resazurin^39^. The procedures are described in Supplementary Information.

### Determination of MIC and MBC

Bacteria were exposed to various dilutions of the selected compounds (0.01–400 µM) using a 96-microtiter plate in similar conditions to the pre-exposure assay, as described above. To determine the MIC, after an 18-h incubation, the planktonic phase was transferred to a clean 96-microtiter plate^40^. The MIC value was defined as the lowest concentration that prevented visible growth. To determine the MBC, aliquots of 50 µL from wells with non-visible bacterial growth were seeded onto TSA plates, which were incubated for 18–20 h at 37 °C. The MBC was defined as the lowest compound concentration that caused ≥ 3-log reduction in the number of CFU. Each test condition was performed using two biological repetitions, with each repetition undergoing three technical replicates.

### Growth curve and time-killing studies

The dynamic of *S. aureus* ATCC 25923 treated with **10d** was studied based on both the measurement of the OD at 595 nm as well as the quantification of viable cells at different time points (0, 1, 2, 4, 6, and 8 h), when incubated at 37 °C under shaking conditions. The procedure is described in Supplementary Information.

### Determination of anti-biofilm potencies and MBIC

To determine the anti-biofilm potencies of **9e**, **10d** and **10e** in both exposure modes of *S. aureus* ATCC 25923 and Newman strains, biofilms were exposed to the compounds using at least eleven concentrations serially diluted (0.01–400 µM). These assays were performed using four replicates and two biological repetitions per tested compound. The cell viability was assessed using the resazurin dye, as mentioned above. The IC_50_ and 95% confidence interval were calculated by a nonlinear regression analysis (sigmoidal dose–response with variable slope) using GraphPad Prism 8.00 software. The MBIC was referred to the compound concentration that caused ≥ 90% inhibition of biofilm formation, compared to the untreated controls, when bacteria and compounds were added simultaneously^41,42^.

### Antibacterial activity against gram-negative strains

The procedure is available in Supplementary Information.

### Efficacy testing

The efficacy of the most active compounds was evaluated based on the logarithm reduction (log_10_ R) assay for both *S. aureus* strains, which was calculated by deducting the average log_10_ cell density (CFU/mL) in treated wells from the average log_10_ cell density in solvent control wells^37,43^. The 18-h-old *S. aureus* biofilm was exposed to the compounds at a final concentration of 100 µM. The procedure for quantifying the number of viable colonies in the biofilm is detailed in Supplementary Information.

### Biofilm biomass and biomatrix assays

The biofilm total biomass was quantified with crystal violet (Sigma-Aldrich), whereas the biomatrix was determined with the WGA probe (Alexa Fluor 488, Molecular Probes Inc.), as reported in our previous work^29,35^. The procedure is described in Supplementary Information.

### Determination of live/dead ratio within pre-formed biofilms and fluorescence microscopy imaging

The 18-h-old biofilms were treated with the compounds (at 100 µM) in 96-microtiter plates, as described above. After a 24-h exposure, the planktonic suspension was removed, and biofilms were washed once with sterile PBS. The LIVE/DEAD Baclight Bacterial Viability kit (Molecular Probes Inc.) was used to determine the green-to-red fluorescence ratio as well as to visualize the live and dead cells by fluorescence microscopy^44^. The procedure is described in Supplementary Information.

### Cytotoxicity study

The procedure is available in Supplementary Information.

### Statistical analysis

The assay performance was monitored by calculating the statistical parameters of screening window coefficient Z′ factor, signal to noise (S/N), and signal to background (S/B) using Microsoft Excel 2016 software. Control wells containing bacteria and TSB were considered as maximum and minimal signal, respectively. For paired comparisons, an unpaired *t*-test with Welch’s correction was applied (*p* < 0.05 was considered statistically significant) and processed with GraphPad Prism 8.00 software.

## Supplementary Information

Supplementary Information 1.

Supplementary Information 2.

## Data Availability

All data generated or analyzed during this study are included in this published article (and its Supplementary Information files).
