# Supplementary material for: Multisubstituted pyrimidines effectively inhibit bacterial growth and biofilm formation of Staphylococcus aureus
Source: Sci Rep. 2021 Apr 12;11:7931. doi: 10.1038/s41598-021-86852-5 (PMC8041844; doi:10.1038/s41598-021-86852-5)
Supplement: Supplementary file 1 — Supplementary Information 1. [file 41598_2021_86852_MOESM1_ESM.pdf]

Supplementary Information

Multisubstituted pyrimidines effectively inhibit bacterial growth and biofilm formation of *Staphylococcus aureus*

Riccardo Provenzani[1‡], Paola San-Martin-Galindo[2‡], Ghada Hassan[1‡], Ashenafi Legehar[1], Aleksi Kallio[1], Henri Xhaard[1], Adyary Fallarero[2], Jari Yli-Kauhaluoma[1*]

[1]Drug Research Program, Division of Pharmaceutical Chemistry and Technology, Faculty of Pharmacy, University of Helsinki, P.O. Box 56 (Viikinkaari 5 E), FI-00014, Helsinki, Finland

[2]Drug Research Program, Division of Pharmaceutical Biosciences, Faculty of Pharmacy, University of Helsinki, P.O. Box 56 (Viikinkaari 5 E), FI-00014, Helsinki, Finland

[*]email: jari.yli-kauhaluoma@helsinki.fi

[‡]These authors contributed equally to this work.

Supplementary Tables

Table S1. Screening of multisubstituted pyrimidines for antimicrobial activity against the planktonic cells as well as biofilm formation of *S. aureus* ATCC 25923. Rifampicin (RMP) was used as the viability control antibiotic. The syntheses of compounds **S1**–**S17** were reported previously[1]; the synthesis of compounds **9a** and **10d** is described in this study. All compounds, unless indicated, were tested at 400 μM concentration. Results are expressed as the mean value ± standard deviation (SD) of two biological repetitions with three parallel replicates.

Scaffold	Cmpd	R_1	R_2	Inhibition percentage of bacterial viability (%) *S. aureus* ATCC 25923			
				Planktonic phase		Biofilm pre-exposure	
				Mean	± SD	Mean	± SD
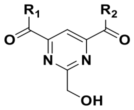	**S1**	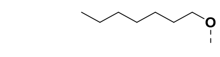	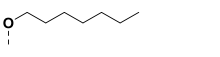	58.70	2.05	90.94	0.98
	**S2**	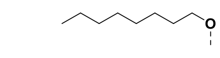	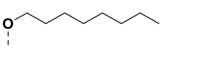	29.83	14.27	15.62	4.33
	**S3**	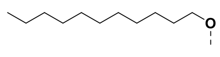	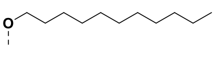	-2.14	4.86	-12.56	14.99
	**S4**	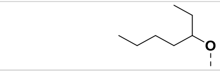	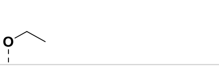	1.55	9.58	-34.32	7.91
	**S5**	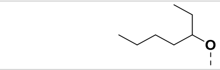	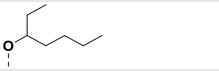	15.45	6.84	-26.09	8.94
	**S6**	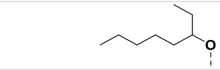	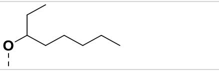	7.44	8.87	-41.73	3.67
	**S7**[a]	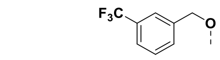	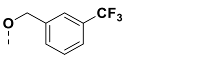	23.64	2.68	32.21	0.18
	**S8**	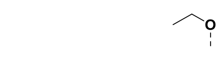	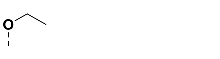	22.32	8.98	-13.08	5.26
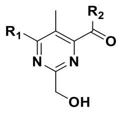	**S9**	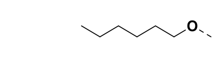	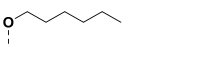	28.80	3.71	-10.95	18.28
	**S10**	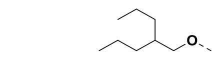	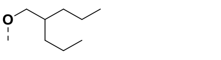	15.54	14.20	-105.25	11.10
	**S11**	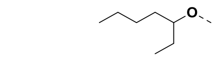	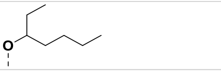	14.80	3.40	-102.28	11.90
	**S12**	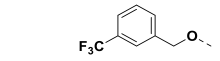	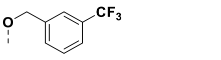	32.33	4.31	-11.42	6.65
	**S13**	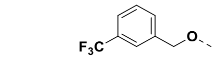	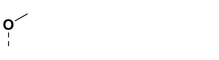	44.19	5.86	56.14	11.59
	**S14**	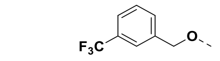	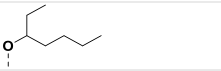	7.08	4.87	-57.64	20.10
	**S15**	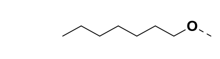	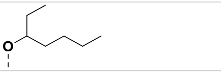	10.22	10.78	-87.67	4.79
	**S16**	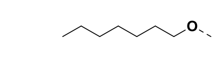	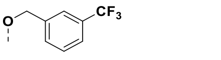	5.66	10.68	-57.05	14.24
	**S17**	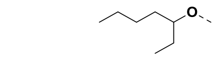	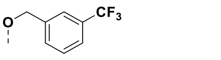	-39.40	43.04	-70.91	17.176
	**9a**	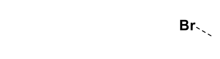	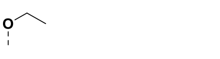	27.68	0.54	-25.43	9.51
	**10d**	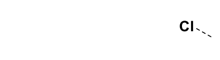	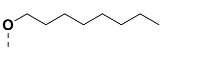	89.41	2.04	91.02	4.747
**Control**	**RMP**[b]			100.37	0.16	98.46	1.05

[a]Tested at 100 μM; [b]tested at 8 μM.

Table S2. Antimicrobial activity of the first set of 2,4,5,6-tetrasubstituted pyrimidines and azithromycin (azm, reference) against *S. aureus* ATCC 25923. All compounds were tested at 400 μM concentration. Results are expressed as the mean value ± standard deviation (SD) of two biological repetitions with three parallel replicates.

	**Inhibition percentage of bacterial viability (%) *S. aureus* ATCC 25923**			
	**Planktonic phase**		**Biofilm pre-exposure**	
**Cmpd**	Mean	±SD	Mean	±SD
**3**	39.70	8.16	-11.85	13.43
**4b**	28.18	15.64	1.40	7.82
**4d**	41.44	15.90	-8.11	4.30
**4f**	49.47	9.26	9.39	18.94
**5b**	16.55	19.23	-6.78	1.69
**5d**	14.69	3.08	-18.95	3.63
**6a**	29.62	20.69	-14.84	16.74
**6b**	13.96	16.44	3.94	13.87
**6d**	32.64	7.06	-14.85	5.80
**7a**	29.06	10.24	9.84	5.28
**7b**	42.61	19.68	22.71	8.56
**7d**	36.82	17.28	-8.82	10.48
**7f**	25.27	14.05	0.15	4.24
**9a**	15.45	28.26	-8.16	7.63
**9b**	56.52	11.80	-5.89	9.32
**9d**	91.65	3.65	83.43	17.56
**10a**	32.89	32.03	-27.85	15.12
**10b**	31.54	19.13	-0.74	9.94
**10f**	3.24	3.35	-21.72	15.85
**12b**	81.84	9.73	88.26	6.36
**12d**	44.80	5.18	45.14	6.11
**azm**	100.23	0.87	98.30	0.25

Table S3. Cytotoxicity study of the most active compounds using Hep2 ATCC cell line. The mean values ± SD are shown for three biological repetitions.

	**Percentage of Hep2 cell line viability**		
**Concentration (μM)**	**9e**	**10d**	**10e**
1	81.5 ± 9.1	96.4 ± 10.6	100.2 ± 3.3
20	82.6 ± 2.1	80.0 ± 7.1	90.5 ± 3.0
40	73.2 ± 17.8	54.8 ± 8.6	75.2 ± 16.1
60	26.2 ± 2.3	-0.1 ± 0.1	22.2 ± 3.5
100	-0.3 ± 0.1	9.4 ± 9.5	0.2 ± 0.8

Supplementary Figures

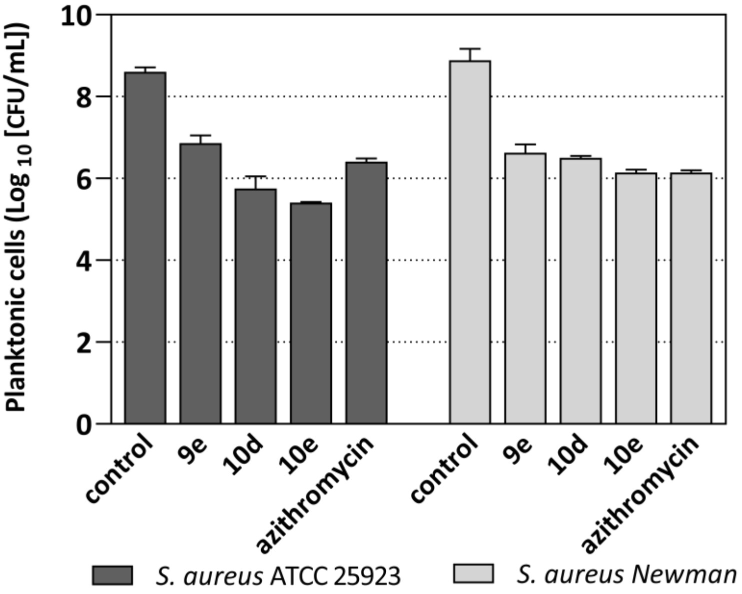

Figure S1. Effect of the most active compounds at 100 μM on the planktonic suspension after the post-exposure assay (mature biofilms) of *S. aureus* ATCC 25923 and *S. aureus* Newman. Viable plate counts of the bacterial suspension were measured after 24 hours of incubation. Results are represented as mean of two biological repetitions ± SD.

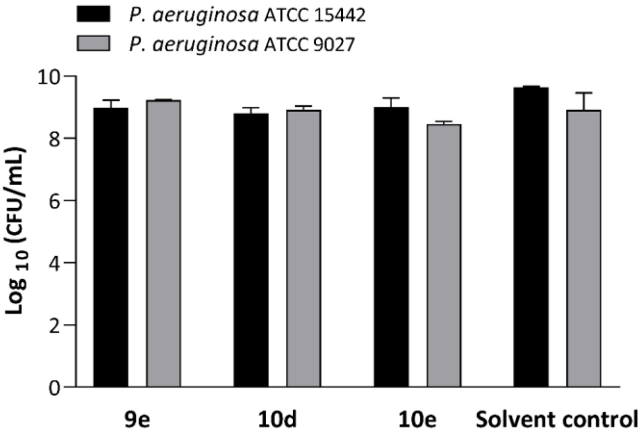

Figure S2. Effect of the most active compounds at 400 μM on bacterial growth of *P. aeruginosa* ATCC 15442 and ATCC 9027 strains. Viable plate counts of the bacterial suspension were measured after 24 hours of incubation. Results are represented as mean of two biological repetitions ± SD. No significant inhibition of viable cells was detected, when compared to biofilms in solvent control (unpaired *t*-test with Welch's correction).

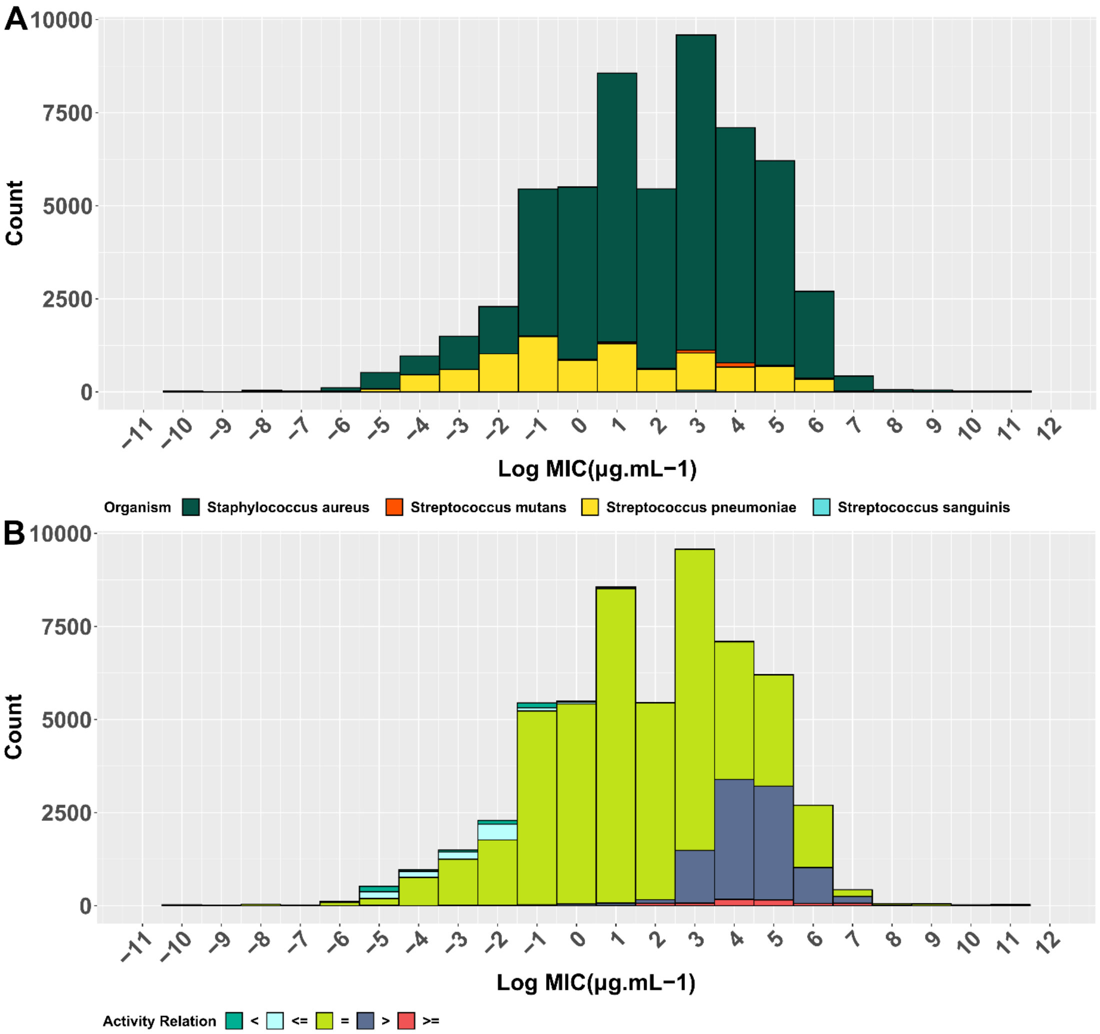

Figure S3. Distribution of bioactivities (Gram-positive) for compounds present in ChEMBLv27. A) Number of compounds by microorganisms. B) Number of compounds distributed by antimicrobial activity, highlighting inactive compounds (with > or ≥ relations). The log (MIC) for **9e**, **10d**, and **10e** are 1.60, 1.78, and 1.78 respectively.

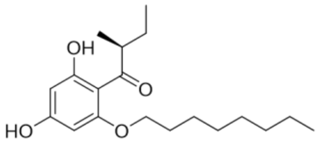

CHEMBL4166911
MIC: 0.25 µg/mL

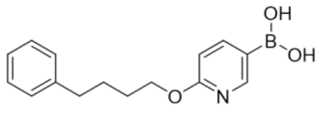

CHEMBL3417347
MIC: 64 µg/mL

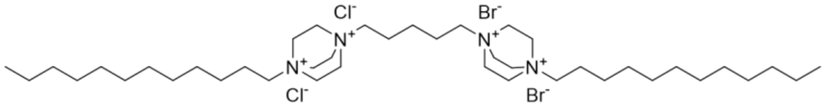

CHEMBL3410371
MIC: 4.7 µg/mL

Figure S4. Selected examples of the closest analogues active towards *S. aureus* strain (Assay ID: ATCC 25923) found in ChEMBLv27.

Supplementary Methods

Chemistry and Experimental Procedures

General information

All reagents were acquired from Fluka, Fluorochem, Merck and Sigma-Aldrich, and were used without further purification. The progress of the chemical reactions was monitored by thin-layer chromatography on Silica Gel 60 F254 aluminum sheets, or amino-functionalized KP-NH TLC glass plates, visualized under UV light (λ: 254/366 nm) and, when necessary, stained with phosphomolybdic acid (10% w/v in EtOH). Microwave reactions were performed with a Biotage Initiator$^+$ SP Wave Microwave Synthesizer. Flash SiO_2 (when specified, amino-functionalized NH_2-SiO_2) column chromatography was performed with a Biotage Isolera Spektra Systems equipped with prepacked columns. The volume of the eluents is expressed in column-volume (CV). ^{1}H, ^{13}C and ^{19}F NMR spectra (available with assignments in Supplementary NMR Appendix including ^{13}C HSQC, ^{13}C HMBC and ^{15}N HMBC 2D NMR spectra) were acquired on a Bruker Ascend 400 MHz - Avance III HD NMR spectrometer and processed with MestReNova 14.2 software. Chemical shifts (δ) are reported as parts per million (ppm) relative to the solvent peaks ($CDCl_3$) at 7.26 and 77.16 ppm for ^{1}H and ^{13}C NMR, respectively. Multiplicities of peaks are represented by s (singlet), d (doublet), t (triplet), q (quartet), quint (quintet), and m (multiplet). Visual features of peaks including broad (br) or apparent (app) are also indicated. In ^{13}C NMR data, peaks referring to two symmetrical carbons (sym, 2C) or two different carbons with overlapping signals (2C) are also indicated. Low resolution mass (MS-APCI) analyses were performed on a MS Advion expression CMS spectrometer equipped with an APCI ion source and an Atmospheric Solids Analysis Probe (ASAP). Exact mass and purity (>95%) of all tested compounds was confirmed by LC-MS analyses with a Waters Acquity UPLC system equipped with an Acquity UPLC BEH C18 column (1.7 mm, 50 mm × 2.1 mm), an Acquity PDA detector and a Waters Synapt G2 HDMS mass spectrometer *via* an ESI ion source in positive mode. Mass data is reported for the molecular ions $[M+H]^+$.

Ethyl 6-hydroxy-2-[(4-methoxyphenoxy)methyl]-5-methylpyrimidine-4-carboxylate (3)

The procedure is reported in our previous work[1].

General procedure I: Acid-catalyzed transesterification

Compound **3** was dissolved in alcohol (3.5–20 equiv) and heated to 100 °C for 3–48 h in the presence of a catalytic amount of H_2SO_4 (0.1 equiv). In some cases, complete dissolution of the starting material occurred while heating. The reaction was quenched by adding a saturated aqueous solution of $NaHCO_3$ and the mixture was extracted with EtOAc. The organic layers were combined, and the solvent was evaporated under reduced pressure at 40 °C. The residual alcohol was removed by vacuum distillation. The crude residue was purified by flash column chromatography with appropriate eluents and gradient.

1-Butyl 6-hydroxy-2-[(4-methoxyphenoxy)methyl]-5-methylpyrimidine-4-carboxylate (4b) and 1-butyl 6-butoxy-2-[(4-methoxyphenoxy)methyl]-5-methylpyrimidine-4-carboxylate (5b)

General procedure I was followed. Compound **3** (0.140 g, 0.440 mmol), *n*-butanol (1.3 mL, 8.8 mmol, 20 equiv), 3 h. Flash chromatography eluents: cyclohexane (A), EtOAc (B); gradient: 5% → 70% B in 14 CV. Compound **4b** was isolated as a pale-yellow solid (73.1 mg, 0.211 mmol, 47.9% yield). TLC (cyclohexane:EtOAc 3:1 v/v): R_f = 0.15; ^{1}H NMR (400 MHz, $CDCl_3$) δ 11.09 (br s, 1H), 7.00–6.90 (m, 2H), 6.90–6.77 (m, 2H), 4.98 (s, 2H), 4.37 (t, *J* = 6.8 Hz, 2H), 3.77 (s, 3H), 2.24 (s, 3H), 1.82–1.70 (m, 2H), 1.52–1.38 (m, 2H), 0.97 (t, *J* = 7.4 Hz, 3H); ^{13}C NMR (101 MHz, $CDCl_3$) δ 165.6, 163.7, 155.1, 154.7, 151.1, 150.1, 125.0, 115.9 (sym, 2C), 115.0 (sym, 2C), 67.3, 66.2, 55.8, 30.7, 19.3, 13.8, 11.8; MS-APCI (*m/z*): $[M+H]^+$ calcd. for $C_{18}H_{23}N_2O_5$, 347.2; found, 347.4; HRMS (*m/z*): $[M+H]^+$ calcd. for $C_{18}H_{23}N_2O_5$, 347. 1607; found, 347. 1605. Compound **5b** was isolated as a colorless to pale-yellow oil (52.7 mg, 0.131 mmol, 29.8% yield). TLC (cyclohexane:EtOAc 3:1 v/v): R_f = 0.66; ^{1}H NMR (400 MHz, $CDCl_3$) δ 6.99–6.88 (m, 2H), 6.88–6.72 (m, 2H), 5.13 (s, 2H), 4.38 (t, *J* = 6.8 Hz, 2H), 4.36 (t, *J* = 6.6 Hz, 2H), 3.75 (s, 3H), 2.26 (s, 3H), 1.86–1.72 (m, 2H), 1.74–1.62 (m, 2H), 1.55–1.31 (m, 4H), 0.96 (t, *J* = 7.4 Hz, 3H), 0.94 (t, *J* = 7.4 Hz, 3H); ^{13}C NMR (101 MHz, $CDCl_3$) δ 169.3, 165.9, 163.1, 155.1, 154.1, 152.9, 116.6 (sym, 2C), 116.1 (sym, 2C), 114.6, 71.2, 67.3, 66.1, 55.8, 30.8, 30.7, 19.28, 19.27, 13.9, 13.8, 11.1; MS-APCI (*m/z*): $[M+H]^+$ calcd. for $C_{22}H_{31}N_2O_5$ 403.2; found, 403.2; HRMS (*m/z*): $[M+H]^+$ calcd. for $C_{22}H_{31}N_2O_5$, 403.2233; found, 403.2232.

1-Heptyl 6-hydroxy-2-[(4-methoxyphenoxy)methyl]-5-methylpyrimidine-4-carboxylate (4c) and 1-heptyl 6-(heptyloxy)-2-[(4-methoxyphenoxy)methyl]-5-methylpyrimidine-4-carboxylate (5c)

General procedure I was followed. Compound **3** (0.200 g, 0.628 mmol), *n*-heptanol (1.78 mL, 12.5 mmol, 20 equiv), 23 h. Flash chromatography eluents: cyclohexane (A), EtOAc (B); gradient: 20% → 60% B in 10 CV. Compound **4c** was isolated as a white to pale-yellow solid (0.120 g, 0.309 mmol, 49.2% yield). TLC (cyclohexane:EtOAc 1:1 v/v): R_f = 0.5; ^{1}H NMR (400 MHz, $CDCl_3$) δ 10.98 (br s, 1H), 6.98–6.89 (m, 2H), 6.89–6.79 (m, 2H), 4.98 (s, 2H), 4.36 (t, *J* = 6.9 Hz, 2H), 3.77 (s, 3H), 2.24 (s, 3H), 1.83–1.70 (m, 2H), 1.47–1.22 (m, 8H), 0.88 (app t, *J* = 6.9 Hz, 3H); ^{13}C NMR (101 MHz, $CDCl_3$) δ 165.7, 163.5, 155.2, 154.7, 151.1, 150.2, 125.0, 115.9 (sym, 2C), 115.0 (sym, 2C), 67.3, 66.5, 55.8, 31.8, 29.0, 28.6, 25.9, 22.7, 14.2, 11.8; HRMS (*m/z*): $[M+H]^+$ calcd. for $C_{21}H_{29}N_2O_5$, 389.2075; found, 389.2076. Compound **5c** was isolated as a pale-yellow oil (112 mg, 0.231 mmol, 36.7% yield). TLC (cyclohexane:EtOAc 1:1 v/v): R_f = 0.95; ^{1}H NMR (400 MHz, $CDCl_3$) δ 6.99–6.86 (m, 2H), 6.86–6.70 (m, 2H), 5.13 (s, 2H), 4.37 (t, *J* = 6.9 Hz, 2H), 4.35 (t, *J* = 6.6 Hz, 2H), 3.75 (s, 3H), 2.26 (s, 3H), 1.83–1.73 (m, 2H), 1.77–1.66 (m, 2H), 1.49–1.20 (m, 16H), 0.89 (app t, *J* = 6.9 Hz, 3H), 0.88 (app t, *J* = 6.9 Hz, 3H); ^{13}C NMR (101 MHz, $CDCl_3$) δ 169.3, 165.9, 163.1, 155.1, 154.1, 153.0, 116.6 (sym, 2C), 116.1 (sym, 2C), 114.6, 71.2, 67.7, 66.4, 55.8, 31.9, 31.8, 29.1, 29.0, 28.74, 28.67, 26.03, 25.98, 22.73, 22.70, 14.21, 14.19, 11.1; HRMS (*m/z*): $[M+H]^+$ calcd. for $C_{28}H_{43}N_2O_5$, 487.3172; found, 487.3170.

1-Octyl 6-hydroxy-2-[(4-methoxyphenoxy)methyl]-5-methylpyrimidine-4-carboxylate (4d) and 1-octyl 2-[(4-methoxyphenoxy)methyl]-5-methyl-6-(octyloxy)pyrimidine-4-carboxylate (5d)

General procedure I was followed. Compound **3** (138 mg, 0.434 mmol), *n*-octanol (1.3 mL, 8.7 mmol, 20 equiv), 24 h. Flash chromatography eluents: cyclohexane (A), EtOAc (B); gradient: 5% → 66% B in 10 CV. Compound **4d** was isolated as a white solid (75.3 mg, 0.187 mmol, 43.2% yield). TLC (cyclohexane:EtOAc 7:1 v/v): R_f = 0.17; ^{1}H NMR (400 MHz, $CDCl_3$) δ 11.05 (br s, 1H), 7.03–6.89 (m, 2H), 6.89–6.75 (m, 2H), 4.98 (d, *J* = 0.8 Hz, 2H), 4.36 (t, *J* = 6.9 Hz, 2H), 3.77 (s, 3H), 2.24 (app t, *J* = 0.7 Hz, 3H), 1.86–1.67 (m, 2H), 1.47–1.36 (m, 2H), 1.36–1.18 (m, 8H), 0.88 (app t, *J* = 6.9 Hz, 3H); ^{13}C NMR (101 MHz, $CDCl_3$) δ 165.6, 163.6, 155.2, 154.7, 151.1, 150.0, 125.1, 115.9 (sym, 2C), 115.0 (sym, 2C), 67.3, 66.5, 55.8, 31.9, 29.28, 29.26, 28.6, 26.0, 22.7, 14.2, 11.9; MS-APCI (*m/z*): $[M+H]^+$ calcd. for $C_{22}H_{31}N_2O_5$ 403.2; found, 403.2; HRMS (*m/z*): $[M+H]^+$ calcd. for $C_{22}H_{31}N_2O_5$, 403.2233; found, 403.2233. Compound **5d** was isolated as a colorless to pale-yellow oil (118 mg, 0.228 mmol, 52.7% yield). TLC (cyclohexane:EtOAc 3:1 v/v): R_f = 0.7; ^{1}H NMR (400 MHz, $CDCl_3$) δ 7.03–6.87 (m, 2H), 6.87–6.70 (m, 2H), 5.13 (s, 2H), 4.37 (t, *J* = 6.9 Hz, 2H), 4.35 (t, *J* = 6.7 Hz, 2H), 3.75 (s, 3H), 2.26 (s, 3H), 1.83–1.73 (m, 2H), 1.75–1.64 (m, 2H), 1.48–1.35 (m, 4H), 1.35–1.16 (m, 16H), 0.883 (app t, *J* = 6.9 Hz, 3H), 0.877 (t, *J* = 6.9 Hz, 3H); ^{13}C NMR (101 MHz, $CDCl_3$) δ 169.3, 165.9, 163.1, 155.1, 154.1, 153.0, 116.6, 116.1 (sym, 2C), 114.6 (sym, 2C), 71.2, 67.7, 66.4, 55.8, 31.93, 31.89, 29.4, 29.34, 29.31, 29.28, 28.72, 28.66, 26.1, 26.0, 22.78, 22.76, 14.23, 14.21, 11.1; MS-APCI (*m/z*): $[M+H]^+$ calcd. for $C_{30}H_{47}N_2O_5$ 515.3; found, 515.3; HRMS (*m/z*): $[M+H]^+$ calcd. for $C_{30}H_{47}N_2O_5$, 515.3485; found, 515.3482.

1-Nonyl 6-hydroxy-2-[(4-methoxyphenoxy)methyl]-5-methylpyrimidine-4-carboxylate (4e) and 1-nonyl 2-[(4-methoxyphenoxy)methyl]-5-methyl-6-(nonyloxy)pyrimidine-4-carboxylate (5e)

General procedure I was followed. Compound **3** (0.200 g, 0.628 mmol), *n*-nonanol (2.19 mL, 12.6 mmol, 20 equiv), 3.5 h. Flash chromatography eluents: cyclohexane (A), EtOAc (B); gradient: 5% → 50% B in 15 CV. Compound **4e** was isolated as a white to pale-yellow solid (152 mg, 0.364 mmol, 57.9% yield). TLC (cyclohexane:EtOAc 3:2 v/v): R_f = 0.32; ^{1}H NMR (400 MHz, $CDCl_3$) δ 10.86 (br s, 1H), 6.96–6.89 (m, 2H), 6.89–6.82 (m, 2H), 4.98 (s, 2H), 4.36 (t, *J* = 6.9 Hz, 2H), 3.77 (s, 3H), 2.25 (s, 3H), 1.83–1.71 (m, 2H), 1.45–1.37 (m, 2H), 1.37–1.23 (m, 10H), 0.87 (app t, *J* = 6.9 Hz, 3H); ^{13}C NMR (101 MHz, $CDCl_3$) δ 165.6, 163.5, 155.2, 154.7, 151.1, 150.8, 125.1, 115.9 (sym, 2C), 115.0 (sym, 2C), 67.3, 66.5, 55.8, 32.0, 29.6, 29.3 (2C), 28.7, 26.0, 22.8, 14.2, 11.9; MS-APCI (*m/z*): $[M+H]^+$ calcd. for $C_{23}H_{33}N_2O_5$ 417.2; found, 417.5. Compound **5e** was isolated as a pale-yellow oil (96.4 mg, 0.178 mmol, 28.3% yield). TLC (cyclohexane:EtOAc 3:2 v/v): R_f = 0.82; ^{1}H NMR (400 MHz, $CDCl_3$) δ 6.96–6.90 (m, 2H), 6.84–6.76 (m, 2H), 5.13 (s, 2H), 4.37 (t, J = 6.9 Hz, 2H), 4.35 (t, J = 6.9 Hz, 2H), 3.75 (s, 3H), 2.26 (s, 3H), 1.83–1.71 (m, 2H), 1.75–1.65 (m, 2H), 1.45–1.35 (m, 4H), 1.34–1.21 (m, 20H), 0.880 (app t, *J* = 6.9 Hz, 3H), 0.875 (app t, *J* = 6.9 Hz, 3H); ^{13}C NMR (101 MHz, $CDCl_3$) δ 169.3, 165.9, 163.1, 155.1, 154.1, 153.0, 116.6, 116.1 (sym, 2C), 114.6 (sym, 2C), 71.2, 67.7, 66.4, 55.8, 32.00, 31.98, 29.64, 29.59, 29.43, 29.37, 29.36 (2C), 28.73, 28.66, 26.1, 26.0, 22.80, 22.79, 14.2 (2C), 11.1; MS-APCI (*m/z*): $[M+H]^+$ calcd. for $C_{32}H_{51}N_2O_5$ 543.4; found, 543.7.

3-(Trifluoromethyl)benzyl 6-hydroxy-2-[(4-methoxyphenoxy)methyl]-5-methylpyrimidine-4-carboxylate (4f)

General procedure I was followed. Compound **3** (330 mg, 1.04 mmol), 3-(trifluoromethyl)benzyl alcohol (0.700 mL, 3.63 mmol, 3.5 equiv), 48 h. Flash chromatography eluents: cyclohexane (A), EtOAc (B); gradient: 5% → 70% B in 10 CV. Compound **4f** was isolated as a white to pale-yellow solid (112 mg, 0.184 mmol, 17.8% yield). TLC (cyclohexane:EtOAc 2:1 v/v): R_f = 0.42; ^{1}H NMR (400 MHz, $CDCl_3$) δ 10.44 (br s, 1H), 7.73 (br s, 1H), 7.67–7.63 (m, 1H), 7.63–7.59 (m, 1H), 7.55–7.50 (m, 1H), 6.96–6.89 (m, 2H), 6.89–6.81 (m, 2H), 5.45 (s, 2H), 4.98 (s, 2H), 3.77 (s, 3H), 2.24 (s, 3H); ^{13}C NMR (101 MHz, $CDCl_3$) δ 165.1, 163.0, 155.3, 154.6, 150.9, 149.1, 136.2, 131.8 (app q, J = 1.4 Hz), 131.3 (q, J = 32.5 Hz), 129.4, 126.2, 125.6 (q, J = 3.8 Hz), 125.2 (q, J = 3.9 Hz), 124.0 (q, J = 272.3 Hz), 115.9 (sym, 2C), 115.1 (sym, 2C), 67.1, 66.8, 55.8, 11.8; ^{19}F NMR (376 MHz, $CDCl_3$) δ -62.71; MS-APCI (*m/z*): $[M+H]^+$ calcd. for $C_{22}H_{20}F_3N_2O_5$, 449.1; found, 449.4; HRMS (*m/z*): $[M+H]^+$ calcd. for $C_{22}H_{20}F_3N_2O_5$, 449.1324; found, 449.1324.

General procedure II: Conversion of 6-hydroxy pyrimidines to 6-bromo/chloro pyrimidines

Phosphoryl bromide or phosphoryl chloride (2–3.5 equiv) was added to a solution of a 6-hydroxy pyrimidine in anhydrous DMF. The obtained light brown solution was microwave-irradiated at 90 °C for 10–30 minutes. The reaction was quenched by adding a saturated aqueous solution of $NaHCO_3$ and the mixture was extracted with EtOAc. The organic layers were combined, and the solvent was evaporated under reduced pressure at 40 °C. Some of the products were isolated as pure without further purification, others were purified by flash column chromatography with appropriate eluents and gradient.

Ethyl 6-bromo-2-[(4-methoxyphenoxy)methyl]-5-methylpyrimidine-4-carboxylate (6a)

The procedure is reported in our previous work[1].

1-Butyl 6-bromo-2-[(4-methoxyphenoxy)methyl]-5-methylpyrimidine-4-carboxylate (6b)

General procedure II was followed. Compound **4b** (75.0 mg, 0.217 mmol), $POBr_3$ (155 mg, 0.541 mmol, 2.5 equiv), DMF (641 μL), 10 min. After the liquid–liquid extraction, compound **6b** was isolated as a white to pale-yellow solid (88.6 mg, 0.216 mmol, quant.). TLC (cyclohexane:EtOAc 2:1 v/v): R_f = 0.75; ^{1}H NMR (400 MHz, $CDCl_3$) δ 6.98–6.90 (m, 2H), 6.85–6.76 (m, 2H), 5.20 (s, 2H), 4.40 (t, J = 6.7 Hz, 2H), 3.75 (s, 3H), 2.49 (s, 3H), 1.81–1.70 (m, 2H), 1.51–1.37 (m, 2H), 0.97 (t, J = 7.4 Hz, 3H); ^{13}C NMR (101 MHz, $CDCl_3$) δ 164.7, 164.0, 158.2, 157.0, 154.4, 152.6, 130.1, 116.4 (sym, 2C), 114.7 (sym, 2C), 70.9, 66.7, 55.8, 30.6, 19.2, 17.8, 13.8; HRMS (*m/z*): $[M+H]^+$ calcd. for $C_{18}H_{22}BrN_2O_4$, 409.0763; found, 409. 0764.

1-Heptyl 6-bromo-2-[(4-methoxyphenoxy)methyl]-5-methylpyrimidine-4-carboxylate (6c)

General procedure II was followed. Compound **4c** (41.4 mg, 0.107 mmol), $POBr_3$ (76.4 mg, 0.266 mmol, 2.5 equiv), DMF (337 μL), 15 min. Flash chromatography eluents: cyclohexane (A), EtOAc (B); gradient: 10% → 60% B in 9 CV. Compound **6c** was isolated as a pale-yellow wax (47.0 mg, 0.104 mmol, 97.7 % yield). TLC (cyclohexane:EtOAc 2:1 v/v): R_f = 0.86; ^{1}H NMR (400 MHz, $CDCl_3$) δ 6.99–6.88 (m, 2H), 6.88–6.73 (m, 2H), 5.20 (s, 2H), 4.39 (t, J = 6.8 Hz, 2H), 3.76 (s, 3H), 2.49 (s, 3H), 1.82–1.72 (m, 2H), 1.45–1.24 (m, 8H), 0.89 (app t, J = 7.0 Hz, 3H); ^{13}C NMR (101 MHz, $CDCl_3$) δ 164.7, 164.1, 158.2, 157.0, 154.5, 152.6, 130.1, 116.4 (sym, 2C), 114.7 (sym, 2C), 70.9, 67.0, 55.8, 31.8, 29.0, 28.6, 25.9, 22.7, 17.8, 14.2; MS-APCI (*m/z*): $[M+H]^+$ calcd. for $C_{21}H_{28}BrN_2O_4$, 451.0; found, 451.0.

1-Octyl 6-bromo-2-[(4-methoxyphenoxy)methyl]-5-methylpyrimidine-4-carboxylate (6d)

General procedure II was followed. Compound **4d** (60.0 mg, 0.149 mmol), $POBr_3$ (106.8 mg, 0.343 mmol, 2.5 equiv), DMF (440 μL), 15 min. After the liquid–liquid extraction, compound **6d**, was isolated as a pale-yellow wax (64.8 mg, 0.139 mmol, 93.5% yield). TLC (cyclohexane:EtOAc 2:1 v/v): R_f = 0.89; ^{1}H NMR (400 MHz, $CDCl_3$) δ 7.04–6.85 (m, 2H), 6.85–6.68 (m, 2H), 5.20 (s, 2H), 4.39 (t, J = 6.8 Hz, 2H), 3.75 (s, 3H), 2.49 (s, 3H), 1.88–1.68 (m, 2H), 1.48–1.37 (m, 2H), 1.37–1.16 (m, 8H), 0.88 (app t, J = 7.0 Hz, 3H); ^{13}C NMR (101 MHz, $CDCl_3$) δ 164.7, 164.0, 158.2, 157.0, 154.5, 152.6, 130.1, 116.4 (sym, 2C), 114.7 (sym, 2C), 70.9, 67.0, 55.8, 31.9, 29.3 (2C), 28.6, 26.0, 22.8, 17.8, 14.2; HRMS (*m/z*): $[M+H]^+$ calcd. for $C_{22}H_{30}BrN_2O_4$, 465.1389; found, 465.1388.

1-Nonyl 6-bromo-2-[(4-methoxyphenoxy)methyl]-5-methylpyrimidine-4-carboxylate (6e)

General procedure II was followed. Compound **4e** (63.0 mg, 0.151 mmol), $POBr_3$ (151.8 mg, 0.529 mmol, 3.5 equiv), DMF (594 μL), 15 min. Flash chromatography eluents: cyclohexane (A), EtOAc (B); gradient: 2% → 20% B in 10 CV. Compound **6e**, was isolated as a pale-yellow wax (56.4 mg, 0.118 mmol, 77.8% yield). TLC (cyclohexane:EtOAc 2:1 v/v): R_f = 0.9; ^{1}H NMR (400 MHz, $CDCl_3$) δ 7.01–6.86 (m, 2H), 6.86–6.72 (m, 2H), 5.20 (s, 2H), 4.39 (t, J = 6.8 Hz, 2H), 3.76 (s, 3H), 2.49 (s, 3H), 1.85–1.68 (m, 2H), 1.45–1.36 (m, 2H), 1.37–1.18 (m, 10H), 0.88 (app t, J = 6.8 Hz, 3H); ^{13}C NMR (101 MHz, $CDCl_3$) δ 164.7, 164.0, 158.2, 157.0, 154.5, 152.6, 130.1, 116.4 (sym, 2C), 114.7 (sym, 2C), 70.9, 67.0, 55.8, 32.0, 29.6, 29.4, 29.3, 28.6, 26.0, 22.8, 17.8, 14.2; MS-APCI (*m/z*): $[M+H]^+$ calcd. for $C_{23}H_{32}BrN_2O_4$, 479.1; found, 479.0.

Ethyl 6-chloro-2-[(4-methoxyphenoxy)methyl]-5-methylpyrimidine-4-carboxylate (7a)

General procedure II was followed. Compound **3** (0.500 g, 1.57 mmol), $POCl_3$ (293 μL, 3.14 mmol, 2 equiv), DMF (5 mL), 10 min. Flash chromatography eluents: cyclohexane (A), EtOAc (B); gradient: 5% → 40% B in 9 CV. Compound **7a**, was isolated as a white solid (382 mg, 1.13 mmol, 72.2% yield). TLC (cyclohexane:EtOAc 2:1 v/v): R_f = 0.4; ^{1}H NMR (400 MHz, $CDCl_3$) δ 7.05–6.90 (m, 2H), 6.90–6.71 (m, 2H), 5.20 (s, 2H), 4.47 (q, J = 7.2 Hz, 2H), 3.75 (s, 3H), 2.49 (s, 3H), 1.42 (t, J = 7.1 Hz, 3H); ^{13}C NMR (101 MHz, $CDCl_3$) δ 164.6, 164.10, 164.09, 157.6, 154.5, 152.6, 127.7, 116.3 (sym, 2C), 114.7 (sym, 2C), 71.0, 62.9, 55.8, 15.2, 14.2; MS-APCI (*m/z*): $[M+H]^+$ calcd. for $C_{16}H_{18}ClN_2O_4$, 337.1; found, 337.1; HRMS (*m/z*): $[M+H]^+$ calcd. for $C_{16}H_{18}ClN_2O_4$, 337.0955; found, 337.0954.

1-Butyl 6-chloro-2-[(4-methoxyphenoxy)methyl]-5-methylpyrimidine-4-carboxylate (7b)

General procedure II was followed. Compound **4b** (50.5 mg, 0.146 mmol), $POCl_3$ (27.2 μL, 0.292 mmol, 2 equiv), DMF (502 μL), 10 min. After the liquid–liquid extraction, compound **7b** was isolated as a yellow wax (52.2 mg, 0.143 mmol, 98.1% yield). TLC (cyclohexane:EtOAc 2:1 v/v): R_f = 0.69; ^{1}H NMR (400 MHz, $CDCl_3$) δ 6.94 (d, J = 0.6 Hz, 2H), 6.85–6.76 (m, 2H), 5.20 (app d, J = 0.6 Hz, 2H), 4.41 (t, J = 6.7 Hz, 2H), 3.75 (s, 3H), 2.48 (app t, J = 0.6 Hz, 3H), 1.82–1.70 (m, 2H), 1.51–1.37 (m, 2H), 0.97 (t, J = 7.4 Hz, 3H); ^{13}C NMR (101 MHz, $CDCl_3$) δ 164.8, 164.12, 164.06, 157.7, 154.4, 152.6, 127.5, 116.3(sym, 2C), 114.7 (sym, 2C), 71.0, 66.7, 55.8, 30.6, 19.2, 15.3, 13.8; HRMS (*m/z*): $[M+H]^+$ calcd. for $C_{18}H_{22}ClN_2O_4$, 365.1268; found, 365.1268.

1-Heptyl 6-chloro-2-[(4-methoxyphenoxy)methyl]-5-methylpyrimidine-4-carboxylate (7c)

General procedure II was followed. Compound **4c** (59.6 mg, 0.153 mmol), $POCl_3$ (42.9 μL, 0.460 mmol, 3 equiv), DMF (600 μL), 15 min. After the liquid–liquid extraction, compound **7c** was isolated as a yellow wax (62.4 mg, 0.153 mmol, quant.). TLC (cyclohexane:EtOAc 2:1 v/v): R_f = 0.72; ^{1}H NMR (400 MHz, $CDCl_3$) δ 6.99–6.90 (m, 2H), 6.85–6.77 (m, 2H), 5.20 (app d, *J* = 0.7 Hz, 2H), 4.40 (t, *J* = 6.8 Hz, 2H), 3.76 (s, 3H), 2.49 (app t, *J* = 0.7 Hz, 3H), 1.84–1.72 (m, 2H), 1.47–1.35 (m, 2H), 1.38–1.23 (m, 6H), 0.89 (app t, *J* = 6.9 Hz, 3H); ^{13}C NMR (101 MHz, $CDCl_3$) δ 164.8, 164.13, 164.07, 157.8, 154.5, 152.6, 127.5, 116.4 (sym, 2C), 114.7 (sym, 2C), 71.0, 67.0, 55.8, 31.8, 29.0, 28.6, 25.9, 22.7, 15.3, 14.2; MS-APCI (*m/z*): $[M+H]^+$ calcd. for $C_{21}H_{28}ClN_2O_4$, 407.2; found, 407.1.

1-Octyl 6-chloro-2-[(4-methoxyphenoxy)methyl]-5-methylpyrimidine-4-carboxylate (7d)

General procedure II was followed. Compound **4d** (48.6 mg, 0.121 mmol), $POCl_3$ (22.5 μL, 0.241 mmol, 2 equiv), DMF (500 μL), 10 min. After the liquid–liquid extraction, compound **7d** was isolated as a yellow wax (50.9 mg, 0.121 mmol, quant.). TLC (cyclohexane:EtOAc 2:1 v/v): R_f = 0.75; ^{1}H NMR (400 MHz, $CDCl_3$) δ 6.99–6.90 (m, 2H), 6.85–6.77 (m, 2H), 5.20 (app d, *J* = 0.6 Hz, 2H), 4.40 (t, *J* = 6.8 Hz, 2H), 3.75 (s, 3H), 2.49 (app t, *J* = 0.6 Hz, 3H), 1.83–1.71 (m, 2H), 1.47–1.37 (m, 2H), 1.36–1.22 (m, 8H), 0.88 (app t, *J* = 7.0 Hz, 3H); ^{13}C NMR (101 MHz, $CDCl_3$) δ 164.8, 164.13, 164.06, 157.8, 154.5, 152.6, 127.5, 116.3 (sym, 2C), 114.7 (sym, 2C), 71.0, 67.0, 55.8, 31.9, 29.3 (2C), 28.6, 26.0, 22.8, 15.3, 14.2; HRMS (*m/z*): $[M+H]^+$ calcd. for $C_{22}H_{30}ClN_2O_4$, 421.1894; found, 421.1895.

1-Nonyl 6-chloro-2-[(4-methoxyphenoxy)methyl]-5-methylpyrimidine-4-carboxylate (7e)

General procedure II was followed. Compound **4e** (63.0 mg, 0.151 mmol), $POCl_3$ (35.2 μL, 0.378 mmol, 2.5 equiv), DMF (500 μL), 15 min. After the liquid–liquid extraction, compound **7e** was isolated as a yellow wax (62.4 mg, 0.143 mmol, 94.8% yield). TLC (cyclohexane:EtOAc 2:1 v/v): R_f = 0.8; ^{1}H NMR (400 MHz, $CDCl_3$) δ 6.99–6.90 (m, 2H), 6.85–6.77 (m, 2H), 5.20 (s, 2H), 4.40 (t, *J* = 6.8 Hz, 2H), 3.76 (s, 3H), 2.49 (s, 3H), 1.83–1.72 (m, 2H), 1.46–1.36 (m, 2H), 1.35–1.24 (m, 10H), 0.88 (app t, *J* = 6.9 Hz, 3H); ^{13}C NMR (101 MHz, $CDCl_3$) δ 164.8, 164.1, 164.1, 157.8, 154.5, 152.6, 127.5, 116.4 (sym, 2C), 114.7 (sym, 2C), 71.0, 67.0, 55.8, 32.0, 29.6, 29.4, 29.3, 28.6, 26.0, 22.8, 15.3, 14.2; MS-APCI (*m/z*): $[M+H]^+$ calcd. for $C_{23}H_{32}ClN_2O_4$, 435.2; found, 435.1.

3-(Trifluoromethyl)benzyl 6-chloro-2-[(4-methoxyphenoxy)methyl]-5-methylpyrimidine-4-carboxylate (7f)

General procedure II was followed. Compound **4f** (63.0 mg, 0.149 mmol), $POCl_3$ (34.9 μL, 0.374 mmol, 2.5 equiv), DMF (1 mL), 30 min. After the liquid–liquid extraction, compound **7f** was isolated as a yellow wax (58.6 mg, 0.126 mmol, 84.0% yield). TLC (cyclohexane:EtOAc 2:1 v/v): R_f = 0.79; ^{1}H NMR (400 MHz, $CDCl_3$) δ 7.71 (br s, 1H), 7.67–7.61 (m, 1H), 7.65–7.59 (m, 1H), 7.57–7.48 (m, 1H), 6.98–6.87 (m, 2H), 6.84–6.76 (m, 2H), 5.47 (s, 2H), 5.21 (app d, *J* = 0.6 Hz, 2H), 3.75 (s, 3H), 2.47 (app t, *J* = 0.6 Hz, 3H); ^{13}C NMR (101 MHz, $CDCl_3$) δ 164.32, 164.30, 164.2, 156.7, 154.5, 152.5, 135.9, 131.8 (app q, *J* = 1.1 Hz), 131.4 (q, *J* = 32.5 Hz), 129.5, 128.1, 125.7 (q, *J* = 3.8 Hz), 125.2 (q, *J* = 3.8 Hz), 124.0 (q, *J* = 272.3 Hz), 116.3 (sym, 2C), 114.7 (sym, 2C), 70.9, 67.2, 55.8, 15.2; ^{19}F NMR (376 MHz, $CDCl_3$) δ -62.72; HRMS (*m/z*): $[M+H]^+$ calcd. for $C_{22}H_{19}ClF_3N_2O_4$, 467.0985; found, 467.0985.

1-Octyl 6-fluoro-2-[(4-methoxyphenoxy)methyl]-5-methylpyrimidine-4-carboxylate (8d)

A mixture of **7d** (79.8 mg, 0.190 mmol), potassium fluoride (35.2 mg, 0.607 mmol, 3.2 equiv), and tetrabutylammonium bromide (0.6 mg, 0.002 mmol, 0.01 equiv) in sulfolane (1.45 mL) was microwave irradiated at 150 °C for 1 h. The mixture was diluted with water, extracted with EtOAc and the combined organic layers were washed with abundant volume of water and brine. The residue was purified by flash column chromatography: cyclohexane (A), EtOAc (B); gradient: 5% → 27% B in 6 CV. Compound **8d** was isolated as a white solid (27.4 mg, 0.0677 mmol, 35.7% yield). TLC (cyclohexane:EtOAc 3:1 v/v): R_f = 0.65; ^{1}H NMR (400 MHz, $CDCl_3$) δ 7.02–6.86 (m, 2H), 6.86–6.71 (m, 2H), 5.20 (s, 2H), 4.41 (t, *J* = 6.9 Hz, 2H), 3.75 (s, 3H), 2.42 (s, 3H), 1.79 (quint, *J* = 6.9 Hz, 2H), 1.46–1.37 (m, 2H), 1.37–1.23 (m, 8H), 0.88 (t, *J* = 6.9 Hz, 3H); ^{13}C NMR (101 MHz, $CDCl_3$) δ 169.9 (d, *J* = 255.4 Hz), 164.6 (d, *J* = 13.5 Hz), 164.4 (d, *J* = 5.9 Hz), 159.4 (d, *J* = 5.8 Hz), 154.5, 152.5, 116.4 (d, *J* = 27.4 Hz), 116.3, 114.7, 70.8, 66.9, 55.8, 31.9, 29.28, 29.29, 28.6, 26.0, 22.8, 14.2, 10.5; ^{19}F NMR (376 MHz, $CDCl_3$) δ -60.82; HRMS (*m/z*): $[M+H]^+$ calcd. for $C_{22}H_{30}FN_2O_4$, 405.2190; found, 405.2191.

General procedure III: oxidative cleavage of the alcohol-protecting *p*-methoxyphenyl group

Ceric (IV) ammonium nitrate (3 equiv) was added to a cooled (-15 °C) solution of a PMP-protected compound in CH_3CN:H_2O 5:1 v/v (0.8–3.9 mL) and stirred for 10 min. The mixture was diluted with water and extracted with EtOAc. The combined organic layers were washed with brine, and the solvent was evaporated under reduced pressure at 40 °C. The residual benzoquinone (yellow needle-shaped crystals) was removed by sublimation in high vacuum. The crude residue was purified by flash column chromatography with appropriate eluents and a gradient or isocratic elution.

Ethyl 6-bromo-2-(hydroxymethyl)-5-methylpyrimidine-4-carboxylate (9a)

General procedure III was followed. Compound **6a** (61.0 mg, 0.160 mmol), CH_3CN/H_2O 5:1 (2.3 mL). Flash chromatography eluents: cyclohexane (A), Et_2O (B); gradient: 18% → 80% B in 9 CV. Compound **9a**, was isolated as an orange oil (0.020 g, 0.072 mmol, 45% yield). TLC (cyclohexane:Et_2O 1:3 v/v): R_f = 0.38; ^{1}H NMR (400 MHz, $CDCl_3$) δ 4.82 (br s, 3H), 4.46 (q, *J* = 7.2 Hz, 3H), 3.33 (br s, 1H), 2.49 (app t, *J* = 0.8 Hz, 4H), 1.41 (t, *J* = 7.2 Hz, 4H); ^{13}C NMR (101 MHz, $CDCl_3$) δ 166.5, 164.5, 158.0, 156.4, 129.6, 64.1, 62.9, 17.7, 14.2; HRMS (*m/z*): $[M+H]^+$ calcd. for $C_9H_{12}BrN_2O_3$, 275.0031; found, 275.0031.

1-Butyl 6-bromo-2-(hydroxymethyl)-5-methylpyrimidine-4-carboxylate (9b)

General procedure III was followed. Compound **6b** (67.6 mg, 0.165 mmol), CH_3CN/H_2O 5:1 (2.4 mL). Flash chromatography (NH_2-SiO_2) eluent: $CHCl_3$ in 4 CV. Compound **9b**, was isolated as a colorless oil (37.4 mg, 0.123 mmol, 74.7% yield). NH_2-TLC ($CHCl_3$): R_f = 0.45; ^{1}H NMR (400 MHz, $CDCl_3$) δ 4.83 (d, *J* = 5.1 Hz, 2H), 4.41 (t, *J* = 6.8, 1.3 Hz, 2H), 3.27 (t, *J* = 5.5 Hz, 1H), 2.49 (app t, *J* = 0.8 Hz, 3H), 1.82–1.70 (m, 2H), 1.52–1.38 (m, 2H), 0.97 (t, *J* = 7.4 Hz, 3H); ^{13}C NMR (101 MHz, $CDCl_3$) δ 166.5, 164.6, 158.0, 156.5, 129.5, 66.7, 64.1, 30.6, 19.2, 17.7, 13.8; HRMS (*m/z*): $[M+H]^+$ calcd. for $C_{11}H_{16}BrN_2O_3$, 303.0344; found, 303.0346.

1-Heptyl 6-bromo-2-(hydroxymethyl)-5-methylpyrimidine-4-carboxylate (9c)
General procedure III was followed. Compound **6c** (41 mg, 0.091 mmol), CH_3CN/H_2O 5:1 (1.3 mL). Flash chromatography eluents: cyclohexane (A), Et_2O (B); gradient: 0% → 70% B in 7 CV. Compound **9c**, was isolated as an orange oil (15 mg, 0.042 mmol, 46% yield). TLC (cyclohexane:Et_2O 1:1 v/v): R_f = 0.4; ^{1}H NMR (400 MHz, $CDCl_3$) δ 4.83 (d, *J* = 4.5 Hz, 2H), 4.40 (t, *J* = 6.8 Hz, 2H), 3.25 (t, *J* = 5.4 Hz, 1H), 2.50 (app t, *J* = 0.8 Hz, 3H), 1.84–1.72 (m, 2H), 1.47–1.38 (m, 2H), 1.37–1.24 (m, 6H), 0.89 (app t, *J* = 6.9 Hz, 3H); ^{13}C NMR (101 MHz, $CDCl_3$) δ 166.5, 164.7, 158.1, 156.6, 129.5, 67.1, 64.1, 31.8, 29.0, 28.6, 25.9, 22.7, 17.7, 14.2; HRMS (*m/z*): $[M+H]^+$ calcd. for $C_{14}H_{22}BrN_2O_3$, 345.0814; found, 345.0817.

1-Octyl 6-bromo-2-(hydroxymethyl)-5-methylpyrimidine-4-carboxylate (9d)
General procedure III was followed. Compound **6d** (51.4 mg, 0.110 mmol), CH_3CN/H_2O 5:1 (1.6 mL). Flash chromatography (NH_2-SiO_2) eluent: $CHCl_3$ in 3 CV. Compound **9d**, was isolated as a pale-yellow oil (27 mg, 0.075 mmol, 68% yield). NH_2-TLC ($CHCl_3$): R_f = 0.5; ^{1}H NMR (400 MHz, $CDCl_3$) δ 4.83 (app q, *J* = 0.8 Hz, 2H), 4.40 (t, *J* = 6.8 Hz, 2H), 3.62 (br s, 1H), 2.50 (app t, *J* = 0.8 Hz, 3H), 1.84–1.72 (m, 2H), 1.47–1.37 (m, 2H), 1.40–1.21 (m, 8H), 0.88 (t, *J* = 6.9 Hz, 3H); ^{13}C NMR (101 MHz, $CDCl_3$) δ 166.5, 164.6, 158.0, 156.6, 129.5, 67.0, 64.1, 31.9, 29.3 (2C), 28.6, 26.0, 22.7, 17.7, 14.2; HRMS (*m/z*): $[M+H]^+$ calcd. for $C_{15}H_{24}BrN_2O_3$, 359.0970; found, 359.0969.

1-Nonyl 6-bromo-2-(hydroxymethyl)-5-methylpyrimidine-4-carboxylate (9e)
General procedure III was followed. Compound **6e** (47 mg, 0.099 mmol), CH_3CN/H_2O 5:1 (1.4 mL). Flash chromatography eluents: cyclohexane (A), Et_2O (B), DCM (C); isocratic elution: 20% B + 16% C in 8 CV. Compound **9e**, was isolated as a pale-yellow oil (22 mg, 0.060 mmol, 61% yield). TLC (cyclohexane:Et_2O:DCM 2:2:1 v/v): R_f = 0.49; ^{1}H NMR (400 MHz, $CDCl_3$) δ 4.82 (d, *J* = 4.9 Hz, 2H), 4.39 (t, *J* = 6.8 Hz, 2H), 3.29 (t, *J* = 5.4 Hz, 1H), 2.49 (app t, *J* = 0.8 Hz, 3H), 1.82–1.72 (m, 2H), 1.46–1.37 (m, 2H), 1.36–1.19 (m, 10H), 0.87 (app t, *J* = 6.9 Hz, 3H); ^{13}C NMR (101 MHz, $CDCl_3$) δ 166.5, 164.6, 158.0, 156.6, 129.5, 67.0, 64.1, 32.0, 29.6, 29.33, 29.30, 28.6, 26.0, 22.8, 17.7, 14.2; HRMS (*m/z*): $[M+H]^+$ calcd. for $C_{16}H_{26}BrN_2O_3$, 373.1127; found, 373.1128.

Ethyl 6-chloro-2-(hydroxymethyl)-5-methylpyrimidine-4-carboxylate (10a)
General procedure III was followed. Compound **7a** (66.2 mg, 0.197 mmol), CH_3CN/H_2O 5:1 (2.8 mL). Flash chromatography eluents: cyclohexane (A), Et_2O (B); gradient: 18% → 100% B in 7 CV. Compound **10a**, was isolated as a pale-yellow oil (18 mg, 0.077 mmol, 39% yield). TLC (cyclohexane:Et_2O 1:3 v/v): R_f = 0.38; ^{1}H NMR (400 MHz, $CDCl_3$) δ 4.82 (app d, *J* = 0.8 Hz, 2H), 4.47 (q, *J* = 7.1 Hz, 2H), 3.30 (br s, 1H), 2.49 (app t, *J* = 0.8 Hz, 3H), 1.42 (t, *J* = 7.1 Hz, 3H); ^{13}C NMR (101 MHz, $CDCl_3$) δ 166.5, 164.6, 163.9, 157.2, 127.1, 64.2, 62.9, 15.2, 14.2; HRMS (*m/z*): $[M+H]^+$ calcd. for $C_9H_{12}ClN_2O_3$, 231.0536; found, 231.0536.

1-Butyl 6-chloro-2-(hydroxymethyl)-5-methylpyrimidine-4-carboxylate (10b)
General procedure III was followed. Compound **7b** (97.5 mg, 0.267 mmol), CH_3CN/H_2O 5:1 (3.9 mL). Flash chromatography (NH_2-SiO_2) eluents: $CHCl_3$ (A), MeOH (B); gradient: 0% → 1% B in 4 CV. Compound **10b**, was isolated as a pale-yellow oil (50.0 mg, 0.193 mmol, 72.3% yield). NH_2-TLC ($CHCl_3$:MeOH 50:1 v/v): R_f = 0.65; ^{1}H NMR (400 MHz, $CDCl_3$) δ 4.82 (app d, *J* = 0.8 Hz, 2H), 4.41 (t, *J* = 6.7 Hz, 2H), 3.28 (br s, 1H), 2.49 (app t, *J* = 0.8 Hz, 3H), 1.83–1.71 (m, 2H), 1.52–1.39 (m, 2H), 0.97 (t, *J* = 7.4 Hz, 3H); ^{13}C NMR (101 MHz, $CDCl_3$) δ 166.5, 164.7, 163.9, 157.3, 127.0, 66.7, 64.2, 30.6, 19.2, 15.2, 13.8; HRMS (*m/z*): $[M+H]^+$ calcd. for $C_{11}H_{16}ClN_2O_3$, 259.0849; found, 259.0851.

1-Heptyl 6-chloro-2-(hydroxymethyl)-5-methylpyrimidine-4-carboxylate (10c)

General procedure III was followed. Compound **7c** (47.3 mg, 0.116 mmol), CH_3CN/H_2O 5:1 (1.7 mL). Flash chromatography eluents: cyclohexane (A), Et_2O (B), DCM (C); isocratic elution: 20% B + 16% C in 8 CV. Compound **10c**, was isolated as a pale-yellow oil (19 mg, 0.063 mmol, 54% yield). TLC (cyclohexane:Et_2O:DCM 2:2:1 v/v): R_f = 0.55; ^1H NMR (400 MHz, $CDCl_3$) δ 4.82 (app d, J = 0.8 Hz, 2H), 4.40 (t, J = 6.8 Hz, 2H), 3.13 (br s, 1H), 2.48 (app t, J = 0.8 Hz, 3H), 1.83–1.72 (m, 2H), 1.46–1.37 (m, 2H), 1.37–1.23 (m, 6H), 0.88 (app t, J = 7.0 Hz, 3H); ^{13}C NMR (101 MHz, $CDCl_3$) δ 166.5, 164.7, 163.9, 157.3, 127.0, 67.0, 64.2, 31.8, 29.0, 28.6, 25.9, 22.7, 15.2, 14.2; HRMS (m/z): $[M+H]^+$ calcd. for $C_{14}H_{22}ClN_2O_3$, 301.1319; found, 301.1318.

1-Octyl 6-chloro-2-(hydroxymethyl)-5-methylpyrimidine-4-carboxylate (10d)

General procedure III was followed. Compound **7d** (101.5 mg, 0.241 mmol), CH_3CN/H_2O 5:1 (3.5 mL). Flash chromatography (NH_2-SiO_2) eluents: $CHCl_3$ (A), MeOH (B); gradient: 0% → 1% B in 4 CV. Compound **10d**, was isolated as a pale-yellow oil (49.0 mg, 0.156 mmol, 64.5% yield). NH_2-TLC ($CHCl_3$:MeOH 50:1 v/v): R_f = 0.7; ^1H NMR (400 MHz, $CDCl_3$) δ 4.82 (app q, J = 0.8 Hz, 2H), 4.40 (t, J = 6.8 Hz, 2H), 3.12 (br s, 1H), 2.49 (app t, J = 0.8 Hz, 3H), 1.84–1.72 (m, 2H), 1.47–1.37 (m, 2H), 1.36–1.21 (m, 8H), 0.88 (app t, J = 7.0 Hz, 3H); ^{13}C NMR (101 MHz, $CDCl_3$) δ 166.5, 164.7, 163.9, 157.3, 127.0, 67.0, 64.2, 31.9, 29.3 (2C), 28.6, 26.0, 22.8, 15.2, 14.2; HRMS (m/z): $[M+H]^+$ calcd. for $C_{15}H_{24}ClN_2O_3$, 315.1476; found, 315.1475.

1-Nonyl 6-chloro-2-(hydroxymethyl)-5-methylpyrimidine-4-carboxylate (10e)

General procedure III was followed. Compound **7e** (47.3 mg, 0.109 mmol), CH_3CN/H_2O 5:1 (1.6 mL). Flash chromatography eluents: cyclohexane (A), Et_2O (B), DCM (C); gradient: 5% → 35% B + 16% C in 8 CV. Compound **10e**, was isolated as an orange oil (4.3 mg, 0.013 mmol, 12% yield). TLC (cyclohexane:Et_2O:DCM 3:2:1 v/v): R_f = 0.37; ^1H NMR (400 MHz, $CDCl_3$) δ 4.83 (app d, J = 3.4 Hz, 2H), 4.40 (t, J = 6.8 Hz, 2H), 3.25 (app t, J = 5.0 Hz, 1H), 2.49 (app t, J = 0.8 Hz, 3H), 1.84–1.72 (m, 2H), 1.47–1.36 (m, 2H), 1.38–1.19 (m, 10H), 0.88 (app t, J = 7.0 Hz, 3H); ^{13}C NMR (101 MHz, $CDCl_3$) δ 166.5, 164.7, 163.9, 157.3, 127.0, 67.0, 64.2, 32.0, 29.6, 29.34, 29.32, 28.6, 26.0, 22.8, 15.2, 14.2; HRMS (m/z): $[M+H]^+$ calcd. for $C_{16}H_{26}ClN_2O_3$, 329.1632; found, 329.1632.

3-(Trifluoromethyl)benzyl 6-chloro-2-(hydroxymethyl)-5-methylpyrimidine-4-carboxylate (10f)

General procedure III was followed. Compound **7f** (32.0 mg, 0.069 mmol), CH_3CN/H_2O 5:1 (1 mL). Flash chromatography eluents: cyclohexane (A), Et_2O (B); gradient: 12% → 100% B in 10 CV. Compound **10f**, was isolated as an orange wax (13 mg, 0.036 mmol, 53% yield). TLC (cyclohexane:Et_2O 1:1 v/v): R_f = 0.18; ^1H NMR (400 MHz, $CDCl_3$) δ 7.74–7.69 (m, 1H), 7.68–7.64 (m, 1H), 7.64–7.61 (m, 1H), 7.58–7.49 (m, 1H), 5.48 (s, 2H), 4.83 (app q, J = 0.8 Hz, 2H), 3.15 (br s, 1H), 2.48 (app t, J = 0.8 Hz, 3H); ^{13}C NMR (101 MHz, $CDCl_3$) δ 166.6, 164.24, 164.19, 156.3, 135.8, 131.8 (q, J = 1.1 Hz), 131.4 (q, J = 32.7 Hz), 129.5, 127.5, 125.8 (q, J = 3.7 Hz), 125.3 (q, J = 4.0 Hz), 124.0 (q, J = 272.4 Hz), 67.3, 64.2, 15.2; ^{19}F NMR (376 MHz, $CDCl_3$) δ -62.76; HRMS (m/z): $[M+H]^+$ calcd. for $C_{15}H_{13}ClF_3N_2O_3$, 361.0567; found, 361.0567.

1-Octyl 6-fluoro-2-(hydroxymethyl)-5-methylpyrimidine-4-carboxylate (11d)

General procedure III was followed. Compound **8d** (23 mg, 0.056 mmol), CH_3CN/H_2O 5:1 (0.8 mL). Flash chromatography eluents: cyclohexane (A), Et_2O (B); gradient: 10% → 30% B in 9 CV. Compound **11d**, was isolated as an orange wax (0.10 g, 0.034 mmol, 60% yield). TLC (cyclohexane:Et_2O 1:2 v/v): R_f = 0.45; ^{1}H NMR (400 MHz, $CDCl_3$) δ 4.81 (app t, J = 0.9 Hz, 2H), 4.41 (t, J = 6.8 Hz, 2H), 2.86 (br s, 1H), 2.42 (app q, J = 0.8 Hz, 3H), 1.86–1.71 (m, 2H), 1.47–1.38 (m, 2H), 1.38–1.21 (m, 8H), 0.87 (app t, 3H); ^{13}C NMR (101 MHz, $CDCl_3$) δ 169.7 (d, J = 256.4 Hz), 167.0 (d, J = 13.3 Hz), 164.3 (d, J = 5.8 Hz), 158.9 (d, J = 5.9 Hz), 115.9 (d, J = 27.3 Hz), 67.0, 64.2, 31.9, 29.3 (2C), 28.6, 26.0, 22.8, 14.2, 10.4; ^{19}F NMR (376 MHz, $CDCl_3$) δ -61.28; HRMS (*m/z*): $[M+H]^+$ calcd. for $C_{15}H_{24}FN_2O_3$, 299.1771; found, 299.1772.

1-Butyl 6-butoxy-2-(hydroxymethyl)-5-methylpyrimidine-4-carboxylate (12b)

General procedure III was followed. Compound **5b** (77.0 mg, 0.191 mmol), CH_3CN/H_2O 5:1 (2.8 mL). Flash chromatography eluents: cyclohexane (A), Et_2O (B), DCM (C); isocratic elution: 35% B + 16% C in 12 CV. Compound **12b**, was isolated as a pale-yellow oil (37.0 mg, 0.125 mmol, 65.3% yield). TLC (cyclohexane:Et_2O:DCM 2:2:1 v/v): R_f = 0.29; ^{1}H NMR (400 MHz, $CDCl_3$) δ 4.69 (app d, J = 0.8 Hz, 2H), 4.41 (t, J = 6.5 Hz, 2H), 4.38 (t, J = 6.8 Hz, 2H), 3.50 (br s, 1H), 2.27 (app t, J = 0.8 Hz, 3H), 1.86–1.68 (m, 4H), 1.55–1.37 (m, 4H), 0.98 (t, J = 7.4 Hz, 3H), 0.97 (t, J = 7.4 Hz, 3H); ^{13}C NMR (101 MHz, $CDCl_3$) δ 169.2, 165.8, 165.3, 154.5, 116.3, 67.4, 66.1, 64.2, 30.8, 30.7, 19.33, 19.29, 13.9, 13.8, 11.0; HRMS (*m/z*): $[M+H]^+$ calcd. for $C_{15}H_{25}N_2O_4$, 297.1814; found, 297.1815.

1-Octyl 2-(hydroxymethyl)-5-methyl-6-(octyloxy)pyrimidine-4-carboxylate (12d)

General procedure III was followed. Compound **5d** (74.0 mg, 0.144 mmol), CH_3CN/H_2O 5:1 (2.1 mL). Flash chromatography eluents: cyclohexane (A), Et_2O (B); gradient: 10% → 33% B in 4 CV + 33% B in 3 CV. Compound **12d** was isolated as a pale-yellow wax (0.020 g, 0.049 mmol, 34% yield). TLC (cyclohexane:Et_2O 1:2 v/v): R_f = 0.43; ^{1}H NMR (400 MHz, $CDCl_3$) δ 4.69 (s, 2H), 4.40 (t, J = 6.6 Hz, 2H), 4.37 (t, J = 6.8 Hz, 2H), 3.49 (br s, 1H), 2.28 (s, 3H), 1.85–1.71 (m, 4H), 1.50–1.38 (m, 4H), 1.37–1.22 (m, 16H), 0.885 (app t, J = 6.8 Hz, 3H), 0.877 (app t, J = 6.8 Hz, 3H); ^{13}C NMR (101 MHz, $CDCl_3$) δ 169.2, 165.7, 165.3, 154.5, 116.3, 67.8, 66.4, 64.2, 31.92, 31.89, 29.4, 29.34, 29.31, 29.29, 28.8, 28.7, 26.1, 26.0, 22.75, 22.67, 14.23, 14.22, 11.1; MS-APCI (*m/z*): $[M+H]^+$ calcd. for $C_{23}H_{41}N_2O_4$, 409.3; found, 409.2; HRMS (*m/z*): $[M+H]^+$ calcd. for $C_{23}H_{41}N_2O_4$, 409.3068; found, 409.3067.

Biology

Bacterial strains and growth conditions

Bacterial cultures, of *S. aureus* (ATCC 25923 and Newman) and *P. aeruginosa* (ATCC 15442 and ATCC 9027), were initiated by inoculating single colonies onto 5 mL of tryptic soy broth (TSB, Neogen) or Miller's Luria-Bertani broth (LBB, VWR Chemicals). Cultures were incubated at 37 °C, 220 rpm, up to reach approx. 10^8 CFU/mL in *S. aureus* and approx. 10^9 CFU/mL in *P. aeruginosa* strains. The bacterial density was estimated by measuring the optical density at 595 nm (0.3–0.4) using a Multiskan Sky Microplate Spectrophotometer, followed by 10-fold serial dilution and plate counting on their corresponding nutrient agar.

Anti-biofilm and antibacterial assays

The anti-biofilm activity of the compounds was primarily assessed against *S. aureus* strains (ATCC 25923 and Newman) under two modes of exposure (pre- and post-exposure) as previously described[2,3]. Due to the poor solubility of some of the compounds in TSB, DMSO 10% (v/v) in TSB was included as a solvent control throughout the experiments, as earlier reported by us[4]. To verify that such DMSO concentration did not considerably affect the activity of the compounds, the inhibitory concentration value of **9e**, **10d** and **10e** to prevent *S. aureus* Newman biofilm growth was recorded using 2% and 10% DMSO. In the pre-exposure experiment, the compounds and the bacteria suspension were added to a 96-well plate, followed by incubation for 18 h (37 °C, 200 rpm). In the post-exposure assay, after 18 h of biofilm formation as mentioned above, the bacterial suspension was discarded, the compounds were added with fresh TSB, and the plates were incubated for additional 24 h under the same growth conditions.

Quantification of bacterial viability with the redox dye resazurin[5]

The resazurin staining was used to determine the viability of bacteria cells in the two mode of exposures as follows. The planktonic suspension was quantified by carefully transferring it to a clean plate and adding 10 μL of resazurin solution (400 μM in PBS, pH 7.3) for 5–10 min, whereas the biofilms were once washed using sterile PBS and then stained with the resazurin solution (200 μL, 20 μM) for 30 min. After the incubation with the dye in darkness at 37 °C and 200 rpm, the fluorescence signal was measured using a Varioskan LUX multimode microplate reader (λ_{ex}=560 nm; λ_{em}=590 nm) with SkanIt 6.0 software.

Growth curve and Time-killing studies

The bacterial initial suspension used was 10^6 CFU/mL, corresponding to OD approx. 0.06. *S. aureus* was exposed to the compound, followed by incubation at 37 °C under shaking conditions. Aliquots were taken at different time points (0, 1, 2, 4, 6, and 8 h). Three concentrations of **10d** were tested at a final concentration related to its MIC value (0.5×MIC, 1×MIC, and 2×MIC). Bacteria exposed to the solvent and TSB were also included in the assay as controls of maximum viability. The test was performed in duplicate in two independent assays. The results were plotted in $OD_{595\ nm}$ vs. time (h) and a time-kill curve of log_{10} (CFU/mL) vs. time (h).

Antibacterial activity against Gram-negative strains

The most active compounds against *S. aureus* strains were tested using two strains of *P. aeruginosa* (ATCC 15442 and ATCC 9027). Thus, the compounds and the bacteria suspension (ca. 10^7 CFU/mL) were added to a 96-well plate, followed by incubation for 24 h (37 °C, 200 rpm). The compounds were tested at a final concentration of 400 μM. After treatment, the bacterial suspensions were recovered, serially diluted in fresh LBB, and plated on LBA. The assay was performed in triplicate with two biological repetitions. The results were expressed as mean of the CFU counted and represented in Briggsian logarithm (log_{10}) (CFU/mL).

Efficacy testing[2,6]

The 18-h-old *S. aureus* biofilm was exposed to the compounds at a final concentration of 100 μM and the plates were incubated for an additional 24 h (37 °C, 200 rpm). Solvent controls wells were included in the assay. After treatment, an aliquot of the planktonic suspensions was subjected to a serial dilution in TSB followed by seeding on TSA plates. The remaining biofilms in the planktonic suspension was discarded, the wells were washed once with sterile MQ-water, and then TSB (100 μL) was added twice to vigorously scrape the biofilm using sterile plastic inoculation loops. The TSB suspension of each sample was recovered in sterile Eppendorf tubes and further homogenized in a water bath sonicator for 5 min (25 °C, 35 kHz), followed by serial dilution in TSB and plating on TSA plates. The CFUs were counted after 18 h incubation at 37 °C.

Biofilm biomass and biomatrix assays[5,7]

In the quantification of the quantification of the total biomass, bacterial cells were treated with the compounds (at 100 μM) using 96-microtiter plates in both pre- and post-exposure conditions, after which the biofilm was stained with 190 μL of crystal violet solution (0.023%; Sigma-Aldrich) in each well and incubated for 5 min. Biofilms were washed twice with MQ-water and let to dry. The bound dye to biofilms was solubilized in ethanol 96% for 1 h. The absorbance measurement was recorded at 595 nm using a Varioskan LUX multimode microplate reader with SkanIt 6.0 software.
In the quantification of the biomatrix, the compounds (at 100 μM) were added on pre-formed biofilm, after which the WGA assay was conducted[7]. Before measuring the fluorescence signal (λ_{ex}=495 nm; λ_{em}=520 nm), the resulting suspensions were diluted 1:1 in 33% acetic acid. The fluorescence signals were blank-corrected. Each assay was performed in two biological replicates in at least duplicate.

Determination of live/dead ratio within preformed biofilms and fluorescence microscopy imaging

The LIVE/DEAD Baclight Bacterial Viability kit (Molecular Probes Inc.) was used to determine the green-to-red fluorescence ratio[8]. Thus, 200 μL of the solution of SYTO 9 and propidium iodide (PI) was added per well, at a final concentration of 5 μM and 30 μM, respectively. After a 15-min incubation in darkness, the fluorescence of SYTO 9 (green) and PI (red) was measured at excitation/emission wavelengths 485/535 nm and 485/635 nm, respectively. The fluorescence signals were blank-corrected before the calculations. For visualizing the live and dead cells in the biofilms, 6 μL of the dye mixture (SYTO 9 and PI) was added per well, followed by incubation in darkness for 15 min. Images were captured with an Invitrogen EVOS FL Imaging System, with a 20× objective, using light filter cubes for the Green Fluorescent Protein and Red Fluorescent Protein. The merged imaged was created directly on the microscope using the multiple-channel function.

Cytotoxicity study

The human epithelial carcinoma Hep-2 cell line (ATCC CCL-23) was used to evaluate the *in vitro* cytotoxicity of the compounds[9,10]. The cells were cultured in 75 cm^2 cell culture flasks containing Dulbecco's modified Eagle's medium (Gibco; Thermo Fisher Scientific) supplemented with fetal bovine serum 10%, L-glutamine 2 mM and gentamicin 20 μg/mL and were incubated at 37 °C with 5% CO_2 in an air-ventilated humidified incubator to reach around 90% confluence. After harvesting by adding 0.25% trypsin in PBS, the cells suspension of 4×10^5 cells/mL was added into 96-well plates (200 μL per well) and incubated at 37 °C. After 24 h, 20 μL of culture media was discarded and replaced with a same volume of compound dilutions (1–100 μM final concentration) and incubated at 37 °C for 24 h. The cell viability was determined by replacing the culture media with the resazurin solution at 20 μM and incubating for 2 h at 37 °C. The reduced resazurin was measured using a Varioskan LUX multimode microplate reader (λ_{ex}=570 nm; λ_{em}=590 nm). Untreated cells were included as positive controls, wells containing only media as negative control as well as 0.5% DMSO as solvent control.

References

1 Provenzani, R. *et al.* Scaffold hopping from (5-hydroxymethyl) isophthalates to multisubstituted pyrimidines diminishes binding affinity to the C1 domain of protein kinase C. *PLoS One* **13**, e0195668, doi:10.1371/journal.pone.0195668 (2018).

2 Fallarero, A. *et al.* (+)-Dehydroabietic Acid, an Abietane-Type Diterpene, Inhibits *Staphylococcus aureus* Biofilms *in Vitro*. *Int J Mol Sci* **14**, 12054-12072, doi:10.3390/ijms140612054 (2013).

3 Manner, S. *et al.* New derivatives of dehydroabietic acid target planktonic and biofilm bacteria in *Staphylococcus aureus* and effectively disrupt bacterial membrane integrity. *Eur J Med Chem* **102**, 68-79, doi:10.1016/j.ejmech.2015.07.038 (2015).

4 Oja, T. *et al.* Effective Antibiofilm Polyketides against *Staphylococcus aureus* from the Pyranonaphthoquinone Biosynthetic Pathways of Streptomyces Species. *Antimicrob Agents Chemoter* **59**, 6046-6052, doi:10.1128/Aac.00991-15 (2015).

5 Sandberg, M., Maattanen, A., Peltonen, J., Vuorela, P. M. & Fallarero, A. Automating a 96-well microtitre plate model for *Staphylococcus aureus* biofilms: an approach to screening of natural antimicrobial compounds. *Int J Antimicrob Agents* **32**, 233-240, doi:10.1016/j.ijantimicag.2008.04.022 (2008).

6 Pitts, B., Hamilton, M. A., Zelver, N. & Stewart, P. S. A microtiter-plate screening method for biofilm disinfection and removal. *J Microbiol Meth* **54**, 269-276, doi:10.1016/S0167-7012(03)00034-4 (2003).

7 Skogman, M. E., Vuorela, P. M. & Fallarero, A. Combining biofilm matrix measurements with biomass and viability assays in susceptibility assessments of antimicrobials against *Staphylococcus aureus* biofilms. *J Antibiot* **65**, 453-459, doi:10.1038/ja.2012.49 (2012).

8 Skogman, M. E., Vuorela, P. M. & Fallarero, A. A Platform of Anti-biofilm Assays Suited to the Exploration of Natural Compound Libraries. *JoVe*, doi:10.3791/54829 (2016).

9 George, S. E. *et al.* Biochemical characterization and evaluation of cytotoxicity of antistaphylococcal chimeric protein P128. *BMC Research Notes* **5**, 280, doi:10.1186/1756-0500-5-280 (2012).

10 Karlsson, D. *et al.* The exploration of thienothiazines as selective butyrylcholinesterase inhibitors. *Eur J Pharm Sci* **47**, 190-205, doi:10.1016/j.ejps.2012.05.014 (2012).
