# Supplementary material for: Multisubstituted pyrimidines effectively inhibit bacterial growth and biofilm formation of Staphylococcus aureus
Source: Sci Rep. 2021 Apr 12;11:7931. doi: 10.1038/s41598-021-86852-5 (PMC8041844; doi:10.1038/s41598-021-86852-5)
Supplement: Supplementary file 2 — Supplementary Information 2. [file 41598_2021_86852_MOESM2_ESM.pdf]

Supplementary NMR Appendix

Multisubstituted pyrimidines effectively inhibit bacterial growth and biofilm formation of *Staphylococcus aureus*

Riccardo Provenzani[1ǂ], Paola San-Martin-Galindo[2ǂ], Ghada Hassan[1ǂ], Ashenafi Legehar[1], Aleksi Kallio[1], Henri Xhaard[1], Adyary Fallarero[2], Jari Yli-Kauhaluoma[1*]

[1]Drug Research Program, Division of Pharmaceutical Chemistry and Technology, Faculty of Pharmacy, University of Helsinki, P.O. Box 56 (Viikinkaari 5 E), FI-00014, Helsinki, Finland
[2]Drug Research Program, Division of Pharmaceutical Biosciences, Faculty of Pharmacy, University of Helsinki, P.O. Box 56 (Viikinkaari 5 E), FI-00014, Helsinki, Finland

[*]email: jari.yli-kauhaluoma@helsinki.fi

[ǂ]These authors contributed equally to this work.

NMR Spectra

Peak reports and multiplet assignments are provided for all compounds. Compound peaks are highlighted in green and solvent peaks in yellow. All 2D-NMR spectra are edited to highlight the correlation peaks. ^{13}C-HSQC spectra include DEPT-135 information with –CH– and –CH_3 correlation peaks in positive phase (**red**) while –CH_2– correlation peaks in negative phase (**blue**). For most of the compounds, ^{15}N-HMBC spectrum and corresponding ^{15}N 1D projection are also reported.

Table of contents

Compounds **Page**

Compound **4b** 005

Compound **4c** 011

Compound **4d** 017

Compound **4e** 023

Compound **4f** 029

Compound **5b** 037

Compound **5c** 043

Compound **5d** 049

Compound **5e** 055

Compound **6b** 061
Compound **6c** 067
Compound **6d** 073
Compound **6e** 079
Compound **7a** 085
Compound **7b** 091
Compound **7c** 097
Compound **7d** 103
Compound **7e** 109
Compound **7f** 115
Compound **8d** 123
Compound **9a** 130
Compound **9b** 134
Compound **9c** 140
Compound **9d** 144
Compound **9e** 150
Compound **10a** 156
Compound **10b** 160

Compound **10c** 166
Compound **10d** 172
Compound **10e** 178
Compound **10f** 182
Compound **11d** 188
Compound **12b** 193
Compound **12d** 199

1H ^{1}H NMR (400 MHz, CDCl$_3$) δ 11.09 (br s, 1H), 7.00 – 6.90 (m, 2H), 6.90 – 6.77 (m, 2H), 4.98 (s, 2H), 4.37 (t, J = 6.8 Hz, 2H), 3.77 (s, 3H), 2.24 (s, 3H), 1.82 – 1.70 (m, 2H), 1.52 – 1.38 (m, 2H), 0.97 (t, J = 7.4 Hz, 3H).

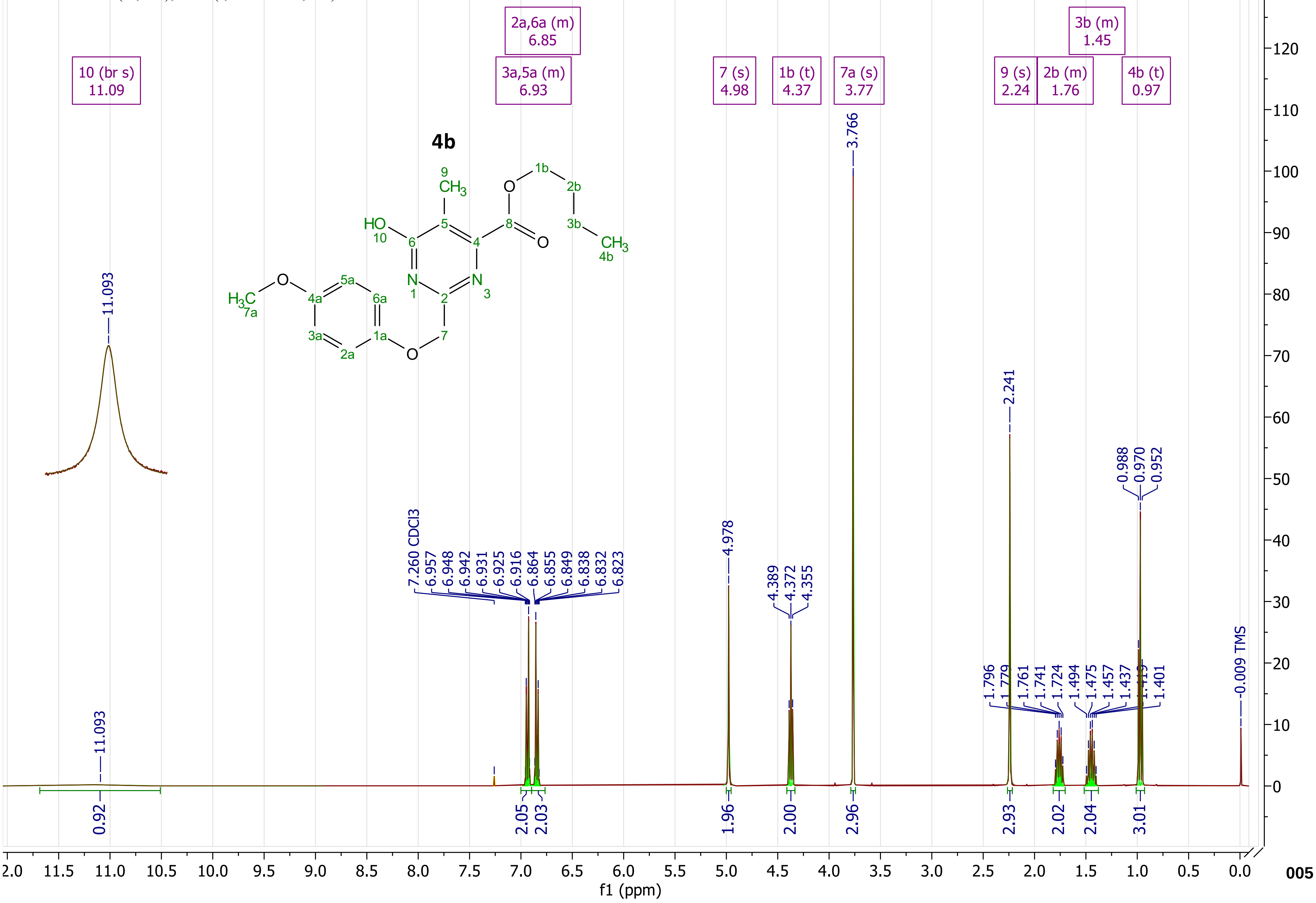

005

13C ^{13}C NMR (101 MHz, $CDCl_3$) δ 165.6, 163.7, 155.1, 154.7, 151.1, 150.1, 125.0, 115.9, 115.0, 67.3, 66.2, 55.8, 30.7, 19.3, 13.8, 11.8.

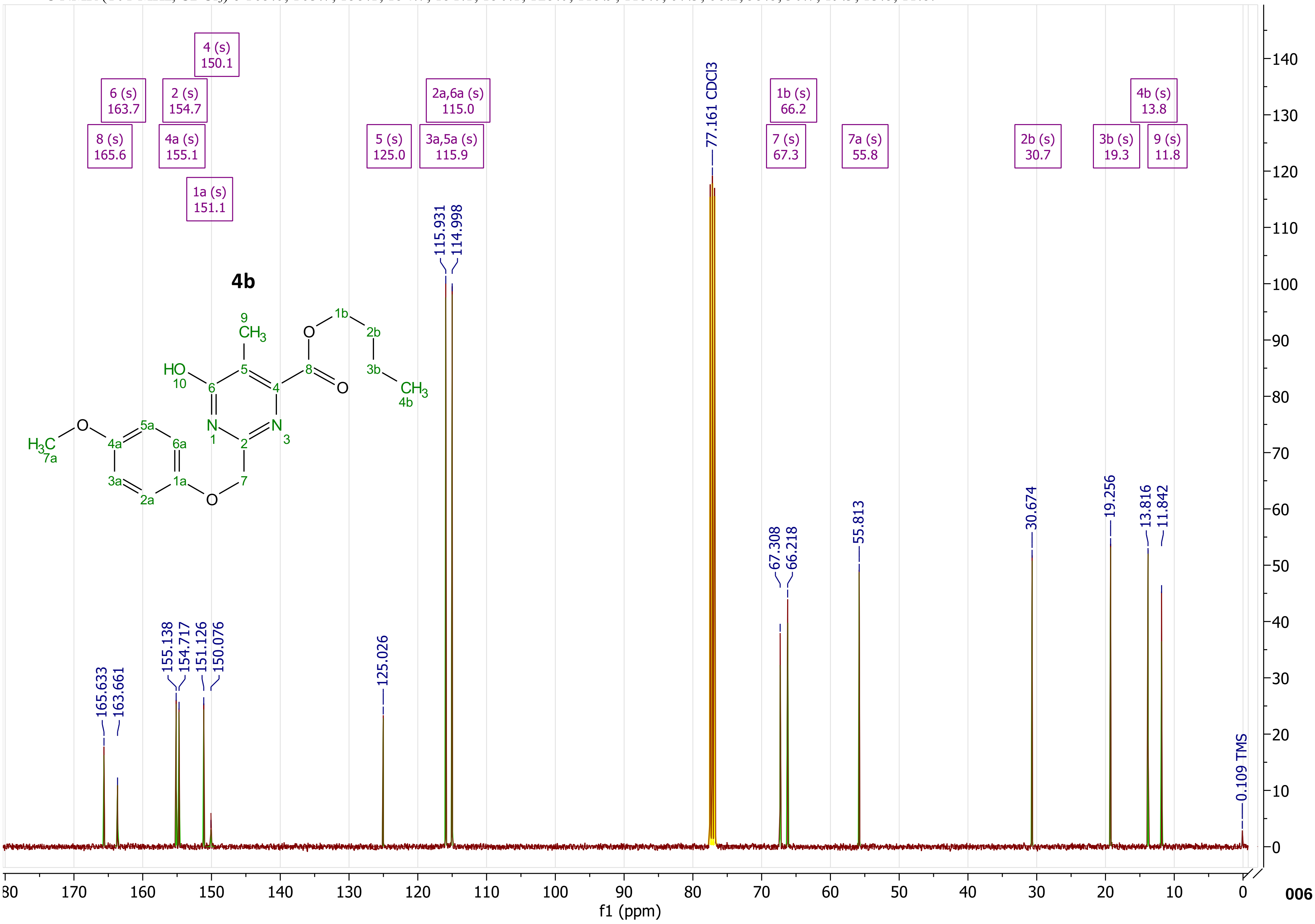

006

13C HSQC

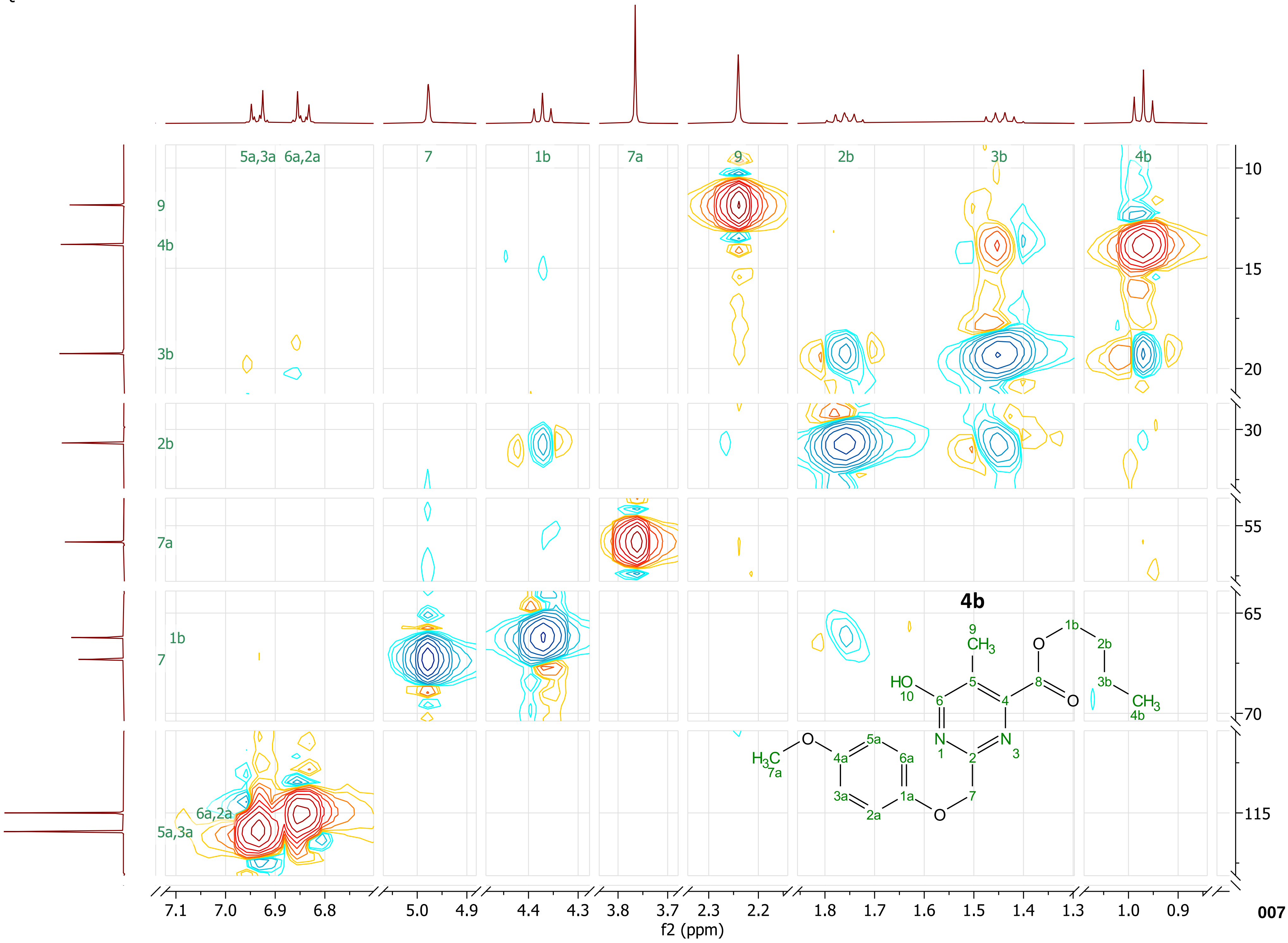
5a,3a
6a,2a
7
1b
7a
9
2b
3b
4b
f1 (ppm)
f2 (ppm)
4b
HO
10
CH3
H3C
7a
O
N
1
3
007

13C HMBC

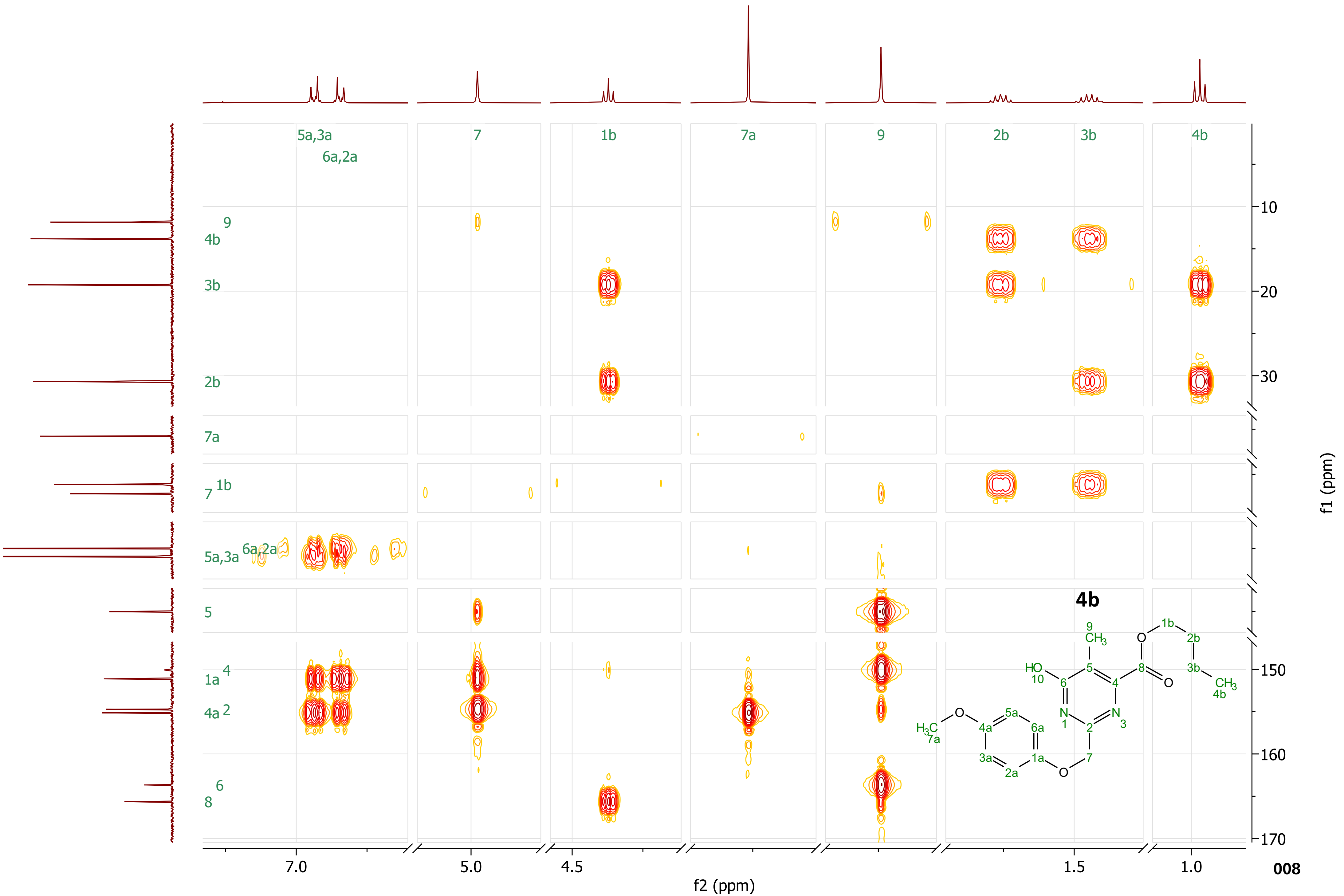
5a,3a
6a,2a
7
1b
7a
9
2b
3b
4b
5a,3a
4b
3b
2b
7a
7
1b
5a,3a
6a,2a
5
1a
4
4a
2
6
8
7.0
5.0
4.5
1.5
1.0
f2 (ppm)
10
20
30
150
160
170
f1 (ppm)

15N HMBC

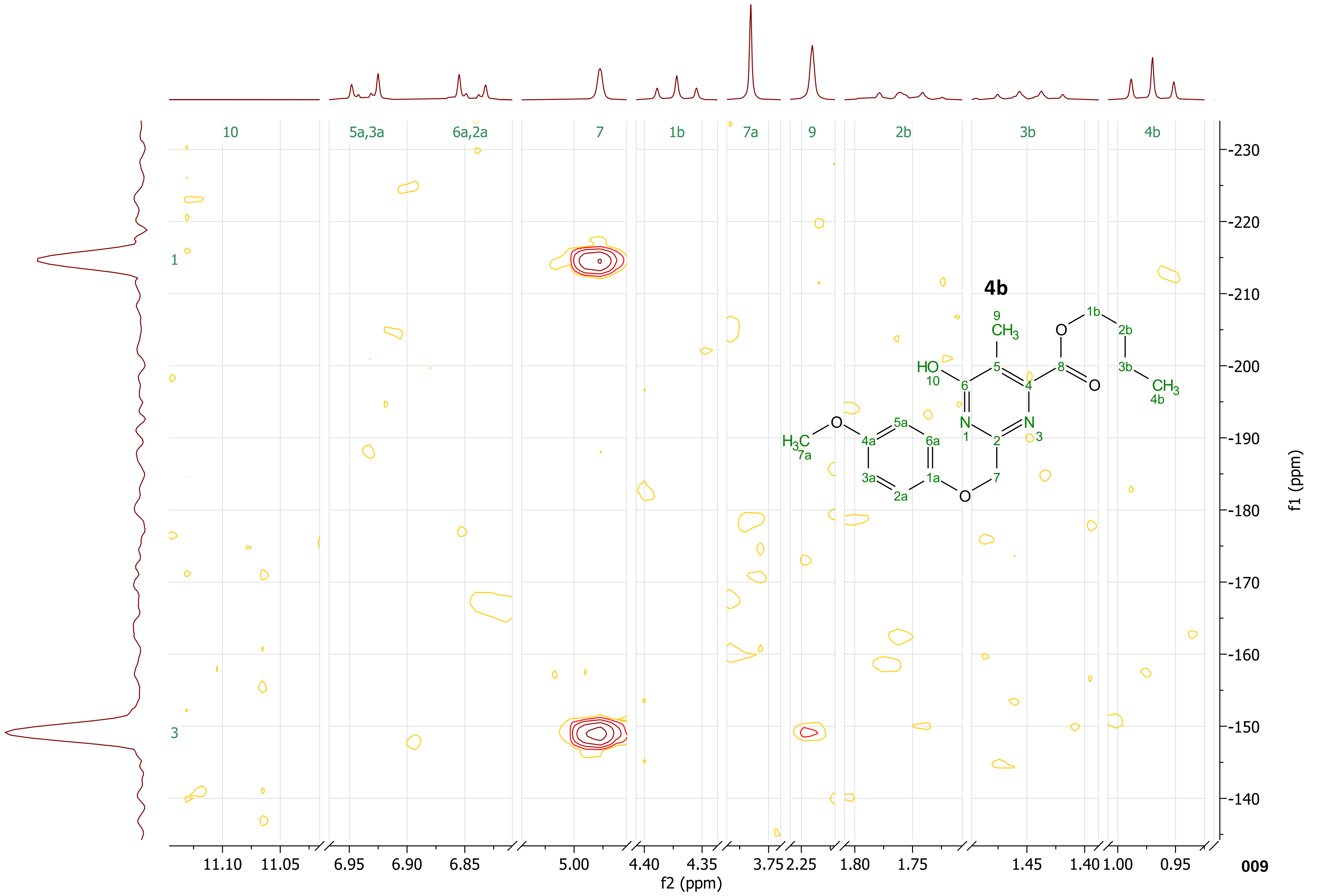
10
5a,3a
6a,2a
7
1b
7a
9
2b
3b
4b
1
3
4b
9
CH3
1b
O
2b
HO
10
6
5
4
8
O
3b
CH3
4b
H3C
7a
O
4a
5a
6a
N
1
2
N
3
3a
2a
1a
7
O
-230
-220
-210
-200
-190
-180
-170
-160
-150
-140
f1 (ppm)
11.10
11.05
6.95
6.90
6.85
5.00
4.40
4.35
3.75
2.25
1.80
1.75
1.45
1.40
1.00
0.95
f2 (ppm)

15N HMBC - f1 internal projection ^{15}N NMR (41 MHz, $CDCl_3$) δ -149.16, -214.81.

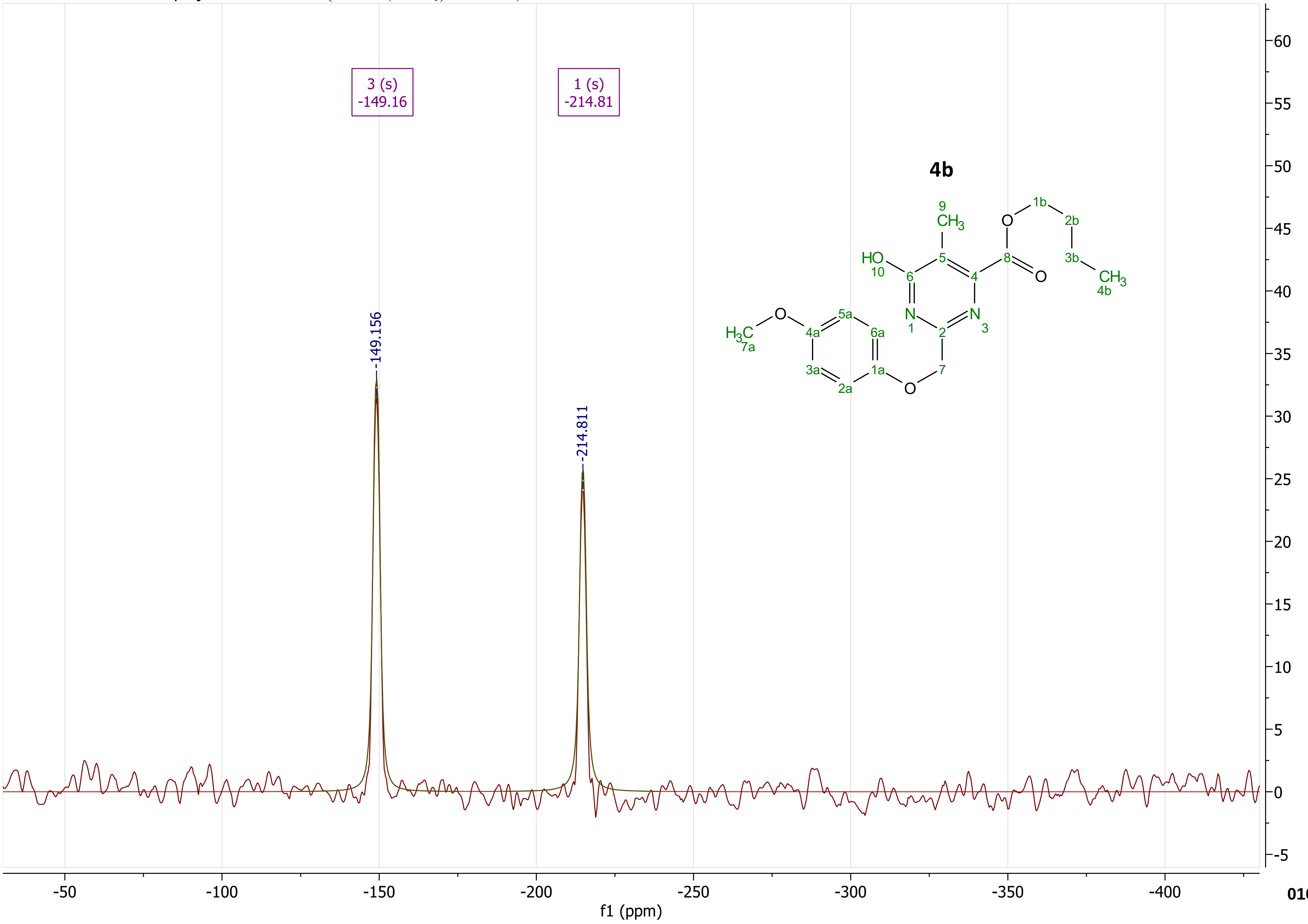

1H ^{1}H NMR (400 MHz, $CDCl_3$) δ 10.98 (br s, 1H), 6.98 – 6.89 (m, 2H), 6.89 – 6.79 (m, 2H), 4.98 (s, 2H), 4.36 (t, J = 6.9 Hz, 2H), 3.77 (s, 3H), 2.24 (s, 3H), 1.83 – 1.70 (m, 2H), 1.47 – 1.22 (m, 8H), 0.88 (app t, J = 6.9 Hz, 3H).

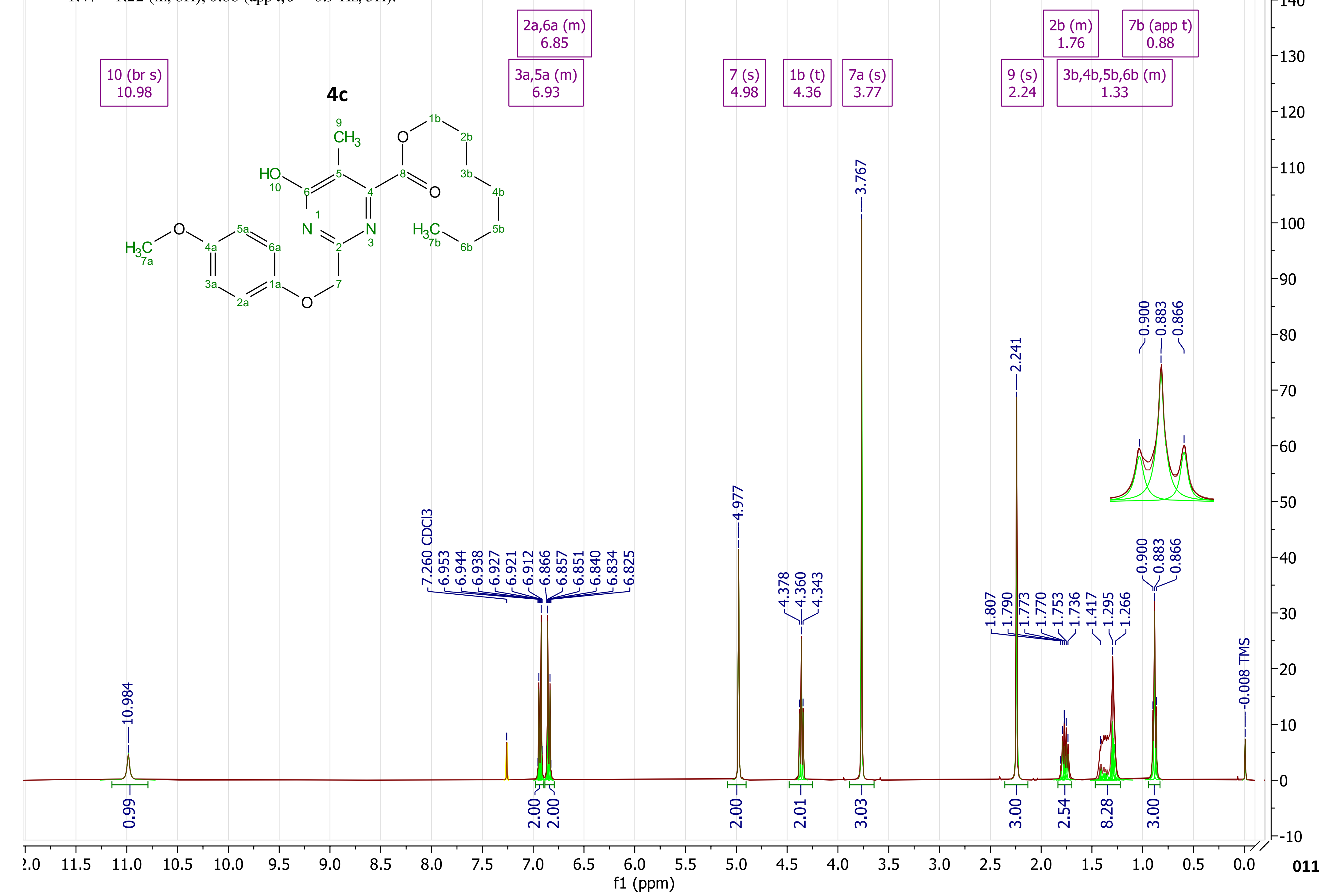

13C ^{13}C NMR (101 MHz, $CDCl_3$) δ 165.7, 163.5, 155.2, 154.7, 151.1, 150.2, 125.0, 115.9 (sym, 2C), 115.0 (sym, 2C), 67.3, 66.5, 55.8, 31.8, 29.0, 28.6, 25.9, 22.7, 14.2, 11.8.

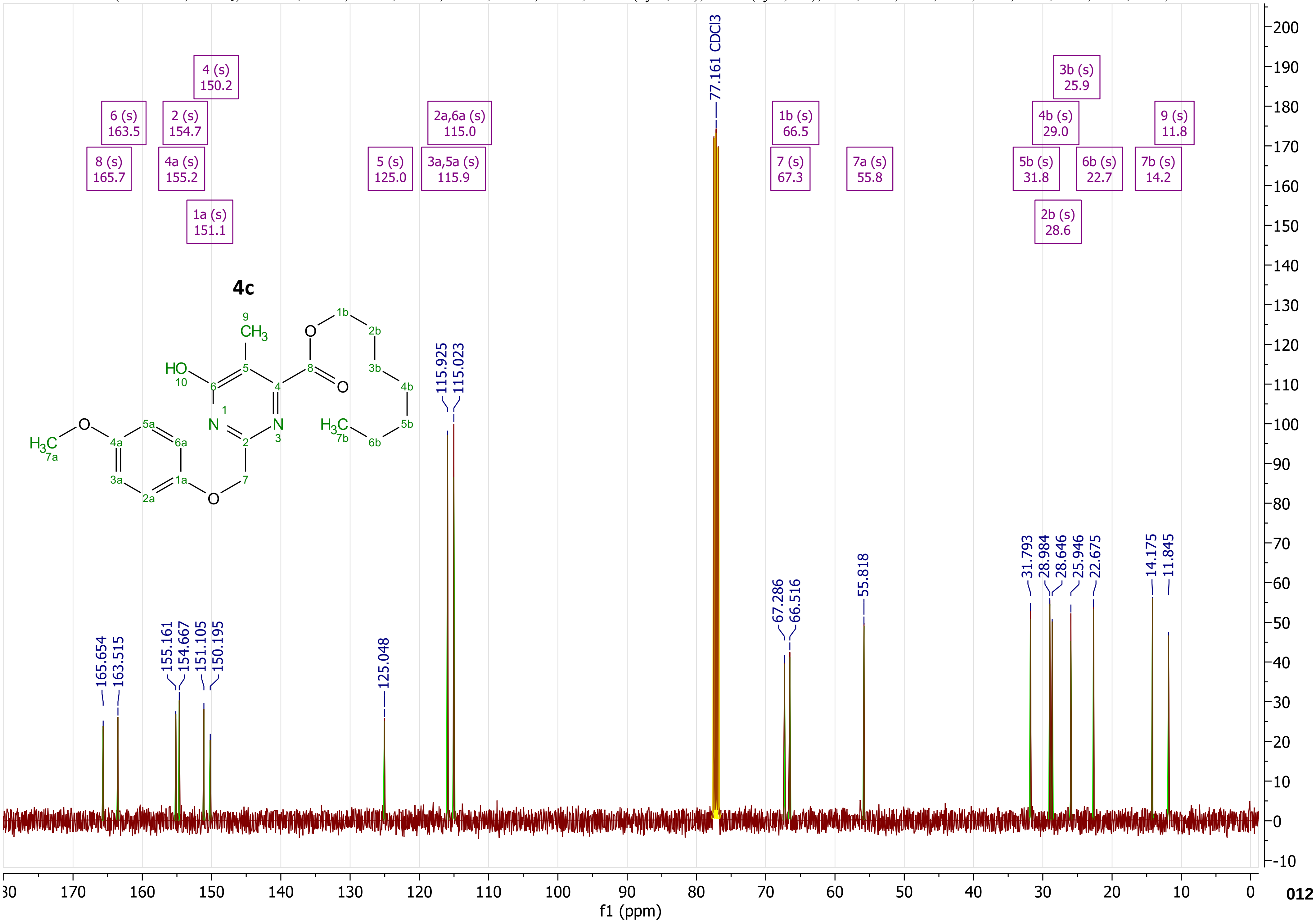

13C HSQC

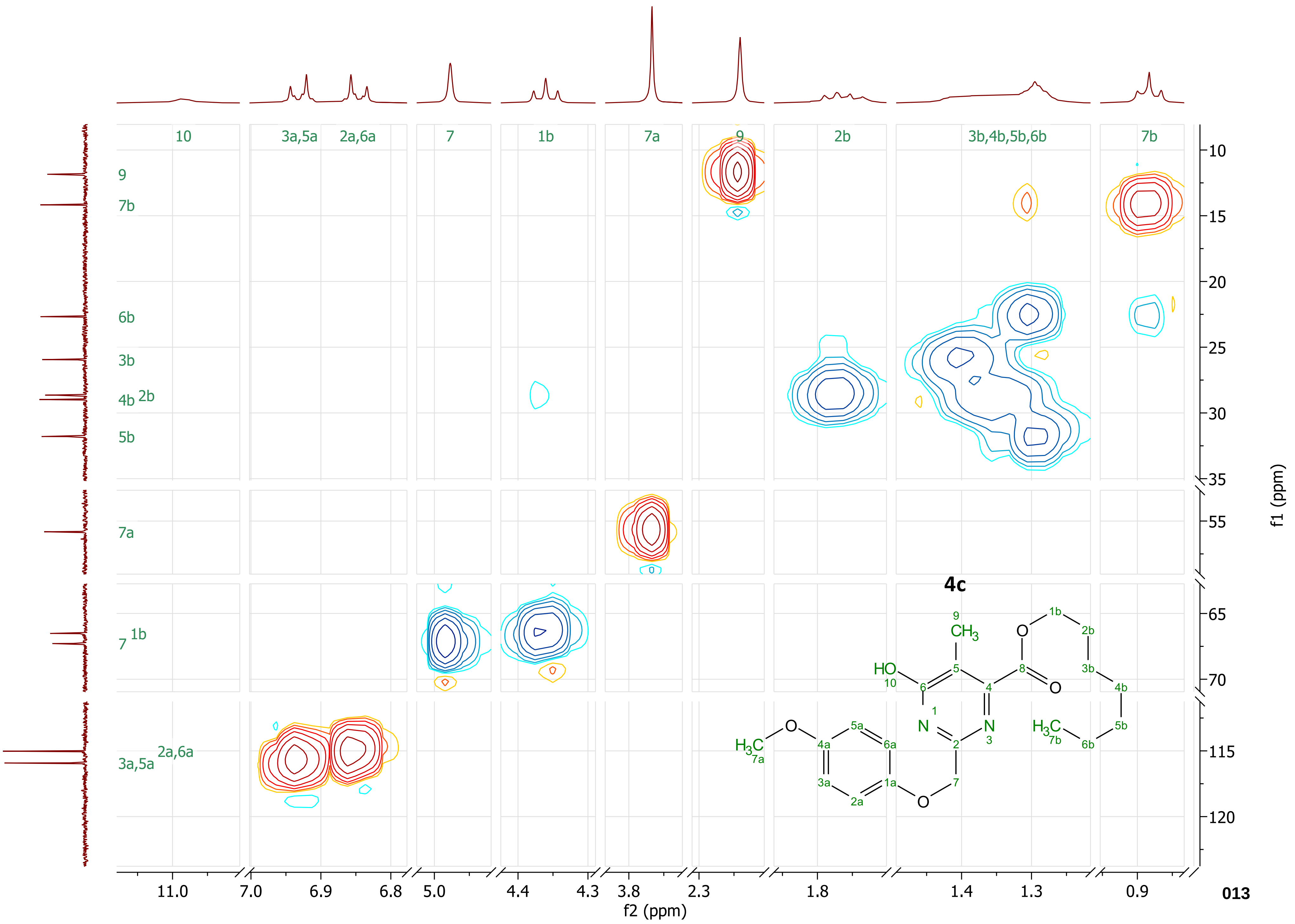
10
3a,5a
2a,6a
7
1b
7a
9
2b
3b,4b,5b,6b
7b
9
7b
6b
3b
4b 2b
5b
7a
7 1b
3a,5a 2a,6a
11.0
7.0
6.9
6.8
5.0
4.4
4.3
3.8
2.3
1.8
1.4
1.3
0.9
f2 (ppm)
f1 (ppm)
4c
CH3
HO
H3C
O
N
N
O
O
H3C
O
O

13C HMBC

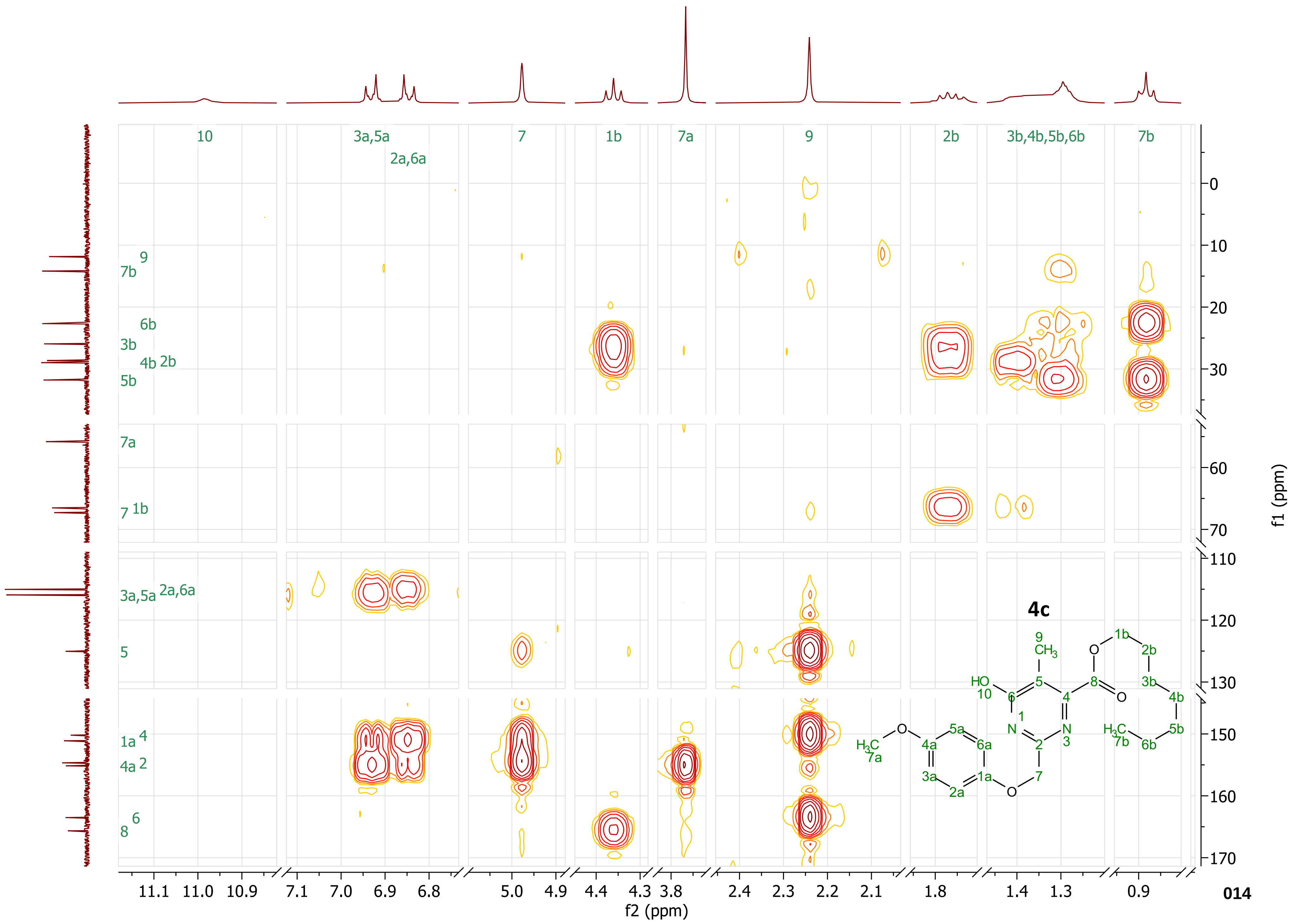

15N HMBC

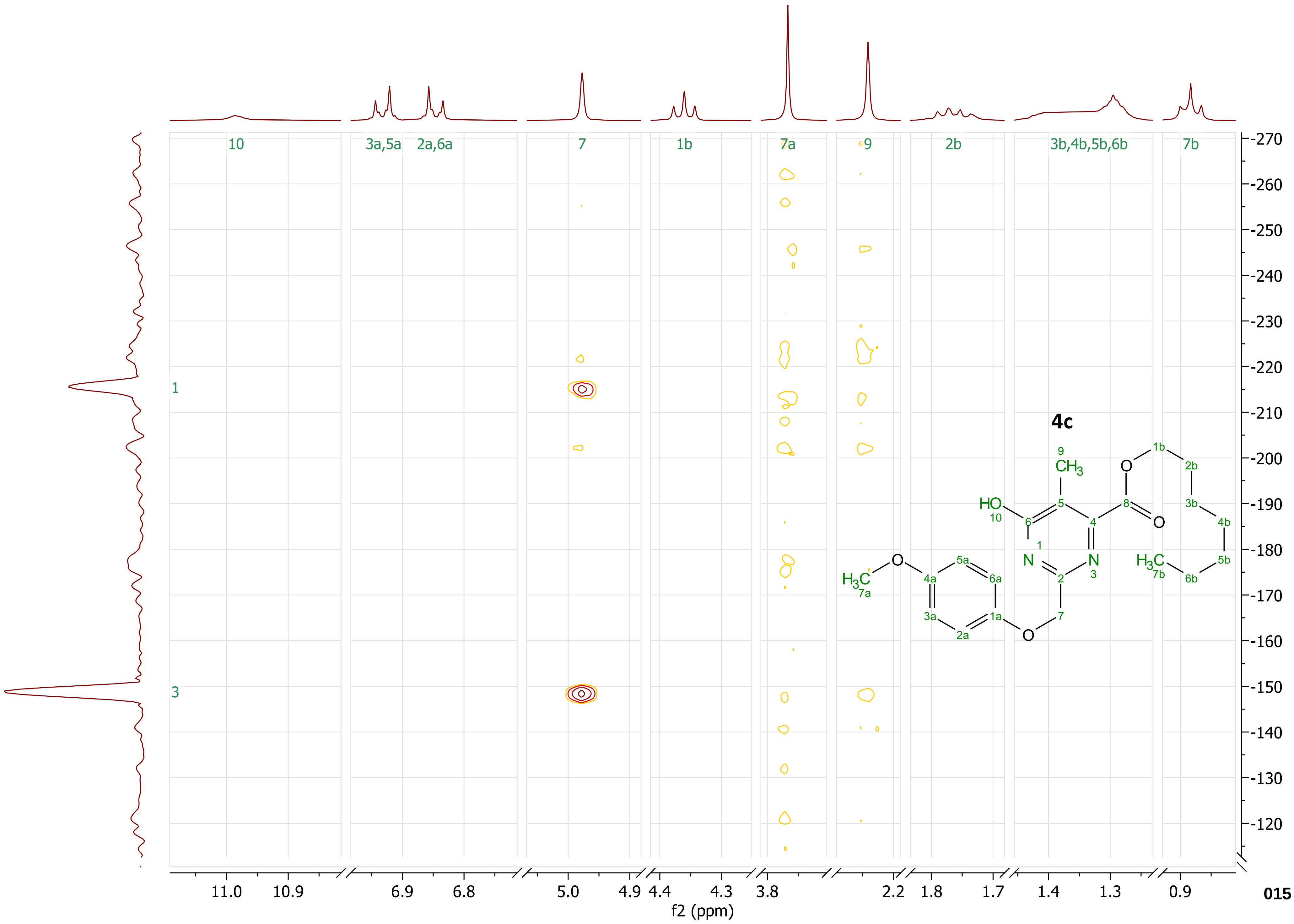

15N HMBC - f1 internal projection ^{15}N NMR (41 MHz, $CDCl_3$) δ -148.84, -215.57.

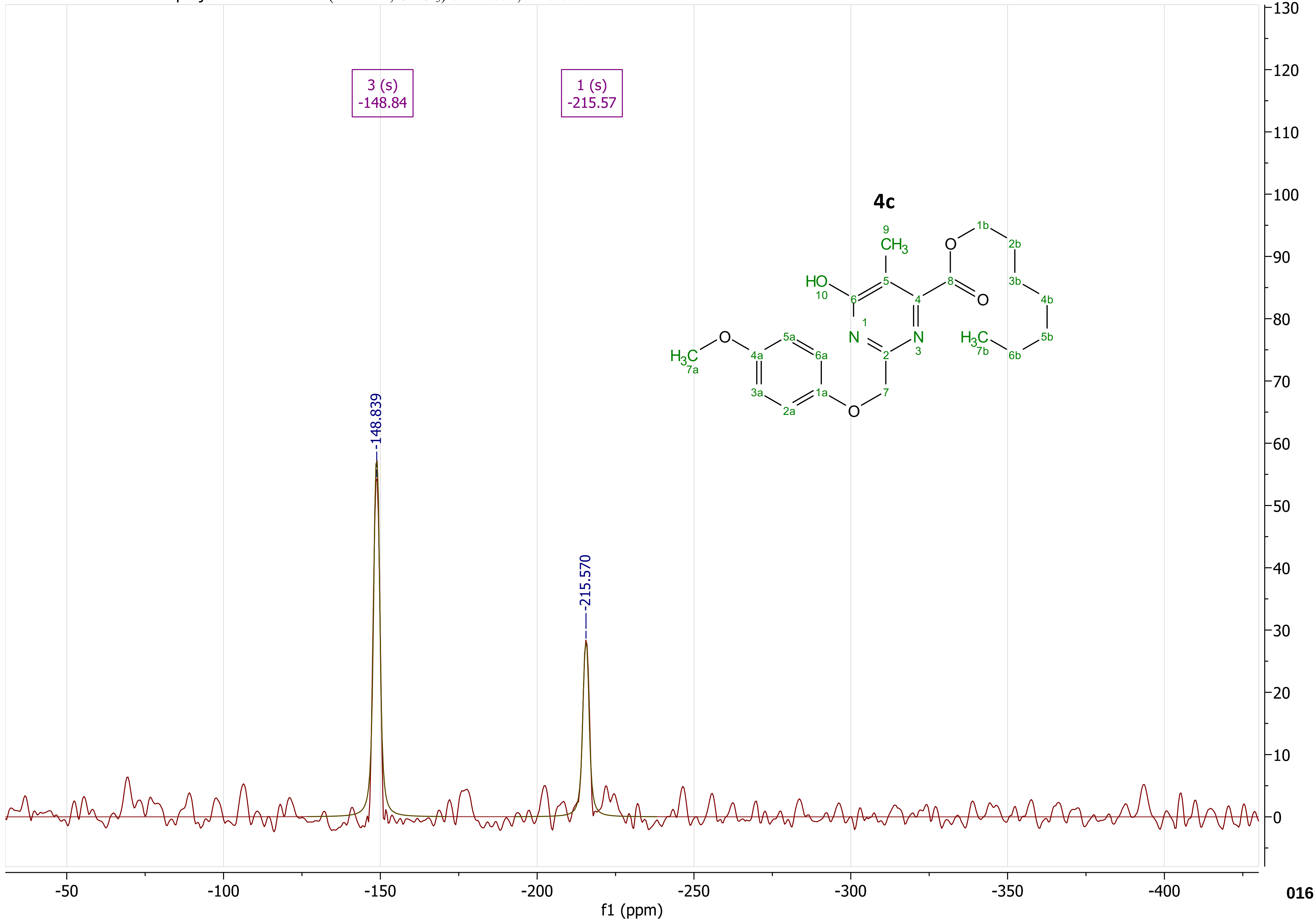

1H ^{1}H NMR (400 MHz, $CDCl_3$) δ 11.05 (br s, 1H), 7.03 – 6.89 (m, 2H), 6.89 – 6.75 (m, 2H), 4.98 (app d, *J* = 0.8 Hz, 2H), 4.36 (t, *J* = 6.9 Hz, 2H), 3.77 (s, 3H), 2.24 (app t, *J* = 0.7 Hz, 3H), 1.86 – 1.67 (m, 2H), 1.47 – 1.36 (m, 2H), 1.36 – 1.18 (m, 8H), 0.88 (app t, *J* = 6.9 Hz, 3H).

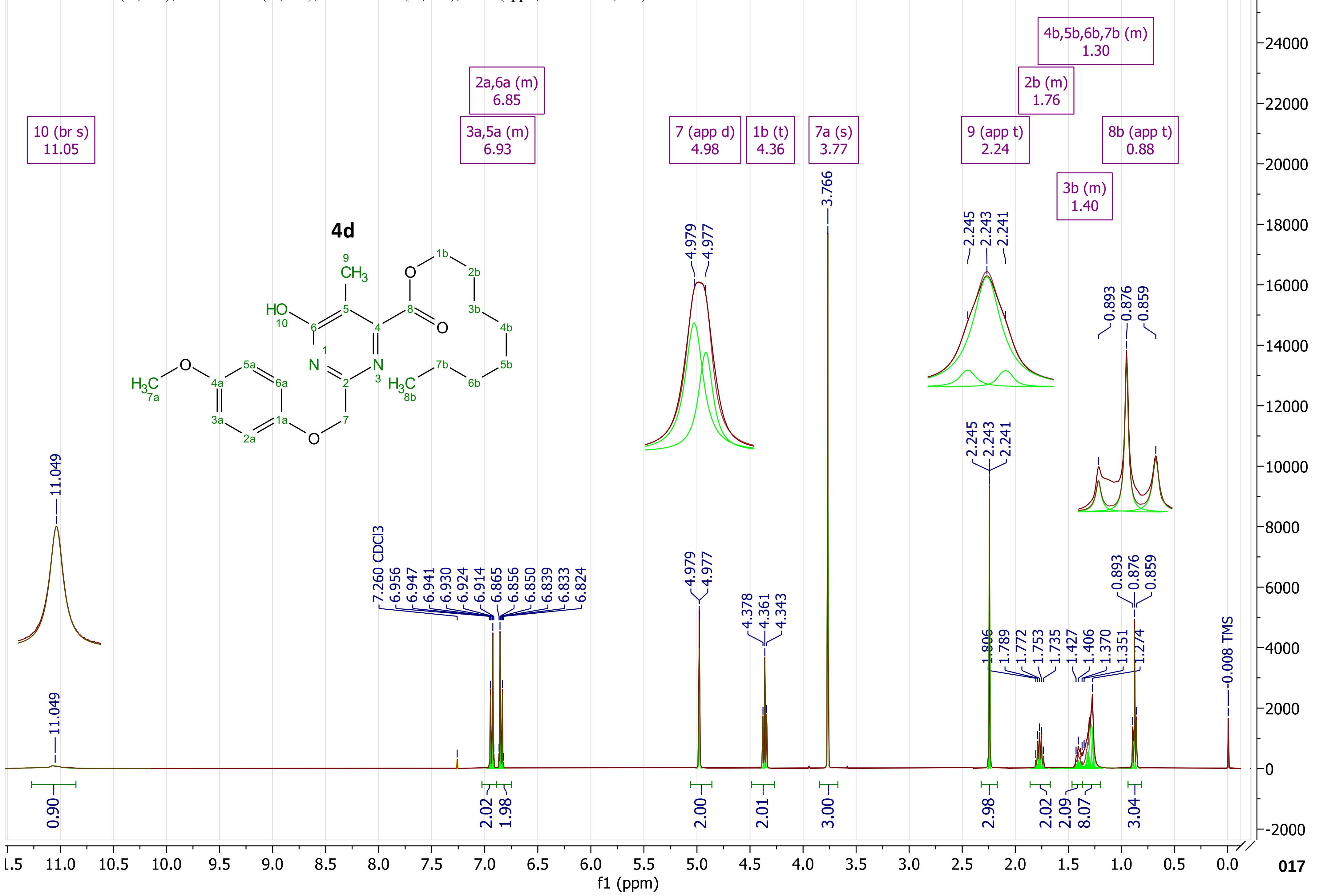

13C ^{13}C NMR (101 MHz, $CDCl_3$) δ 165.6, 163.6, 155.2, 154.7, 151.1, 150.0, 125.1, 115.9 (sym, 2C), 115.0 (sym, 2C), 67.3, 66.5, 55.8, 31.9, 29.28, 29.26, 28.6, 26.0, 22.7, 14.2, 11.9.

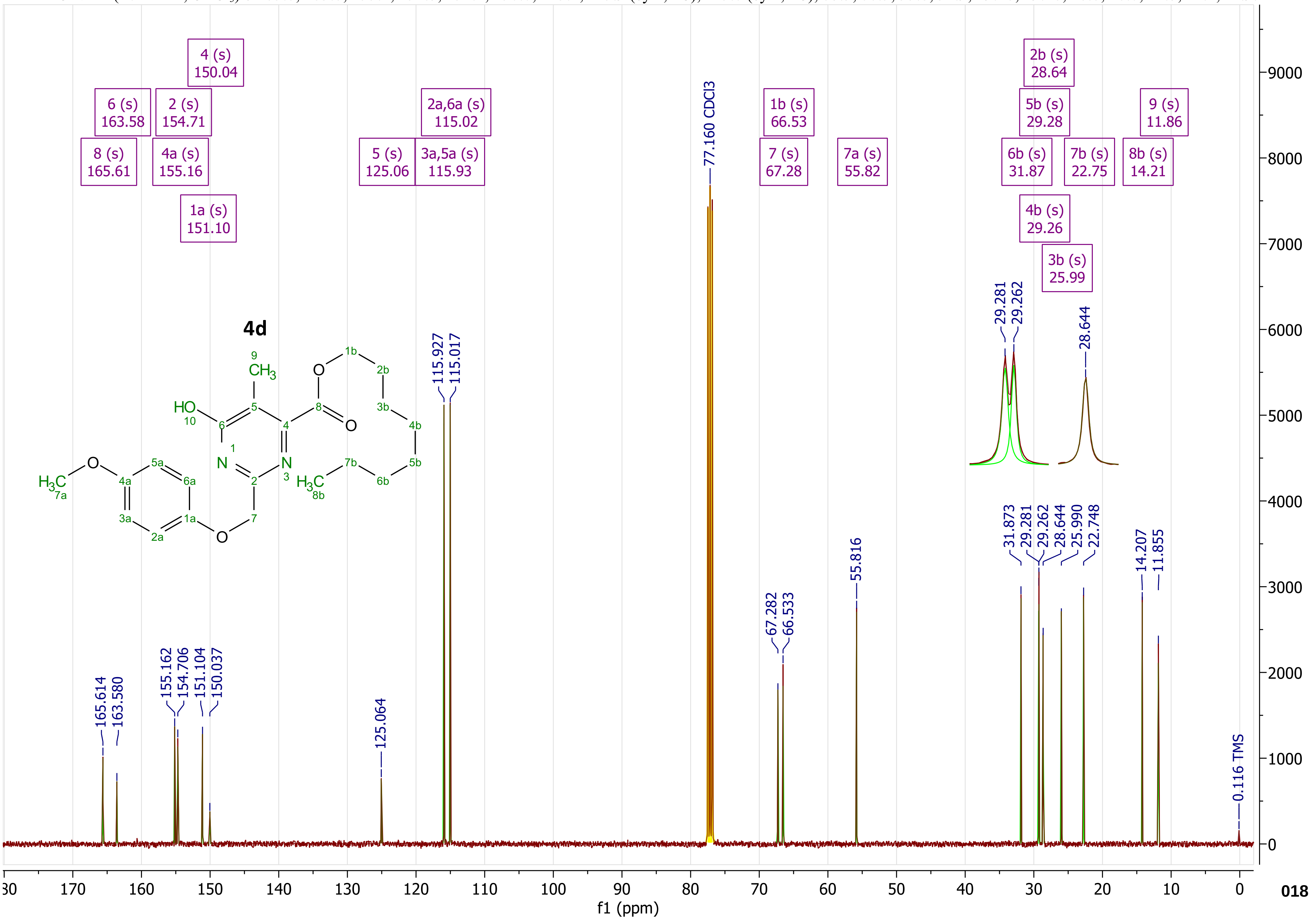

13C HSQC

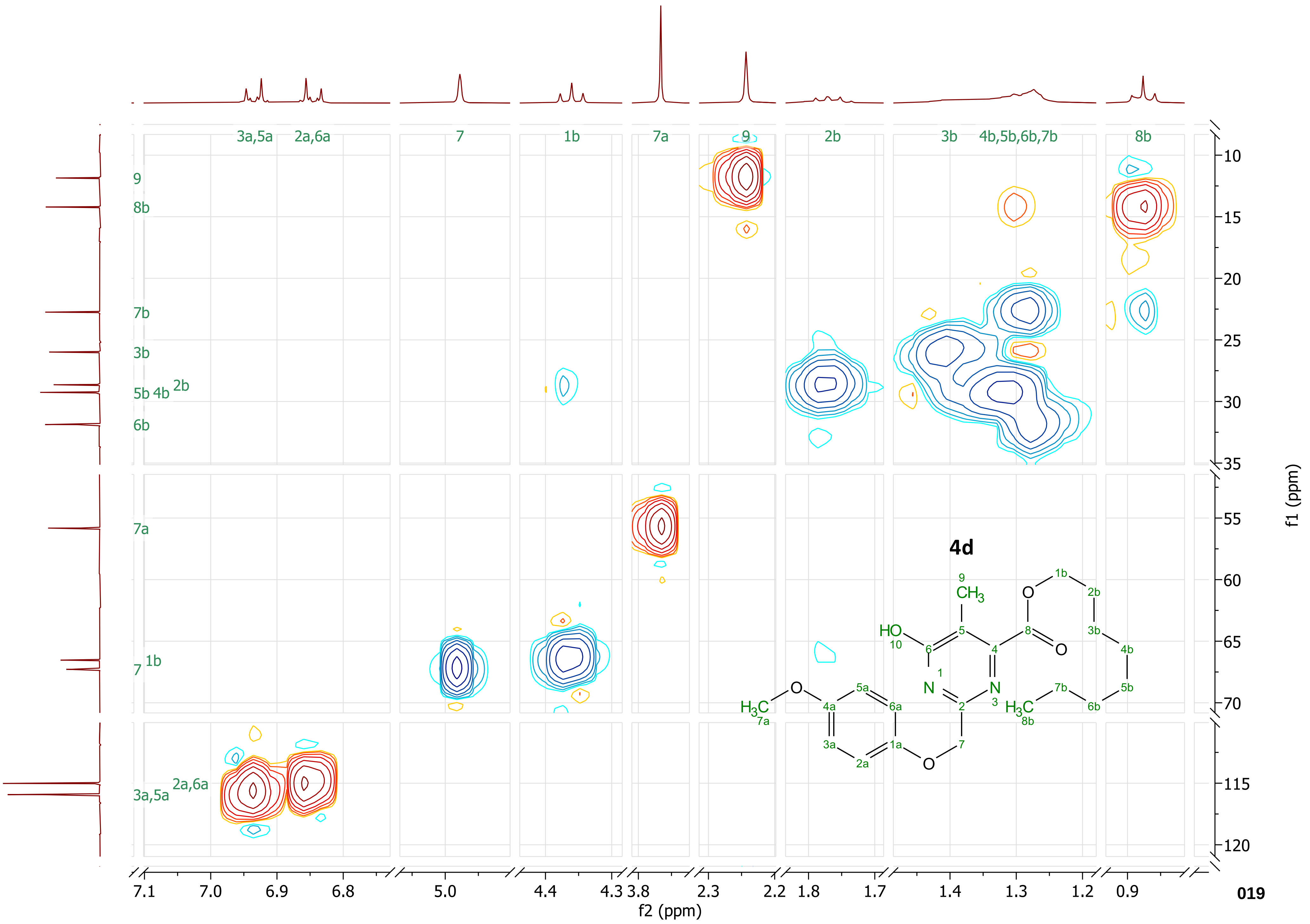
3a,5a
2a,6a
7
1b
7a
9
2b
3b
4b,5b,6b,7b
8b
5b 4b
6b
f1 (ppm)
f2 (ppm)
4d
CH3
HO
O
N
H3C

13C HMBC

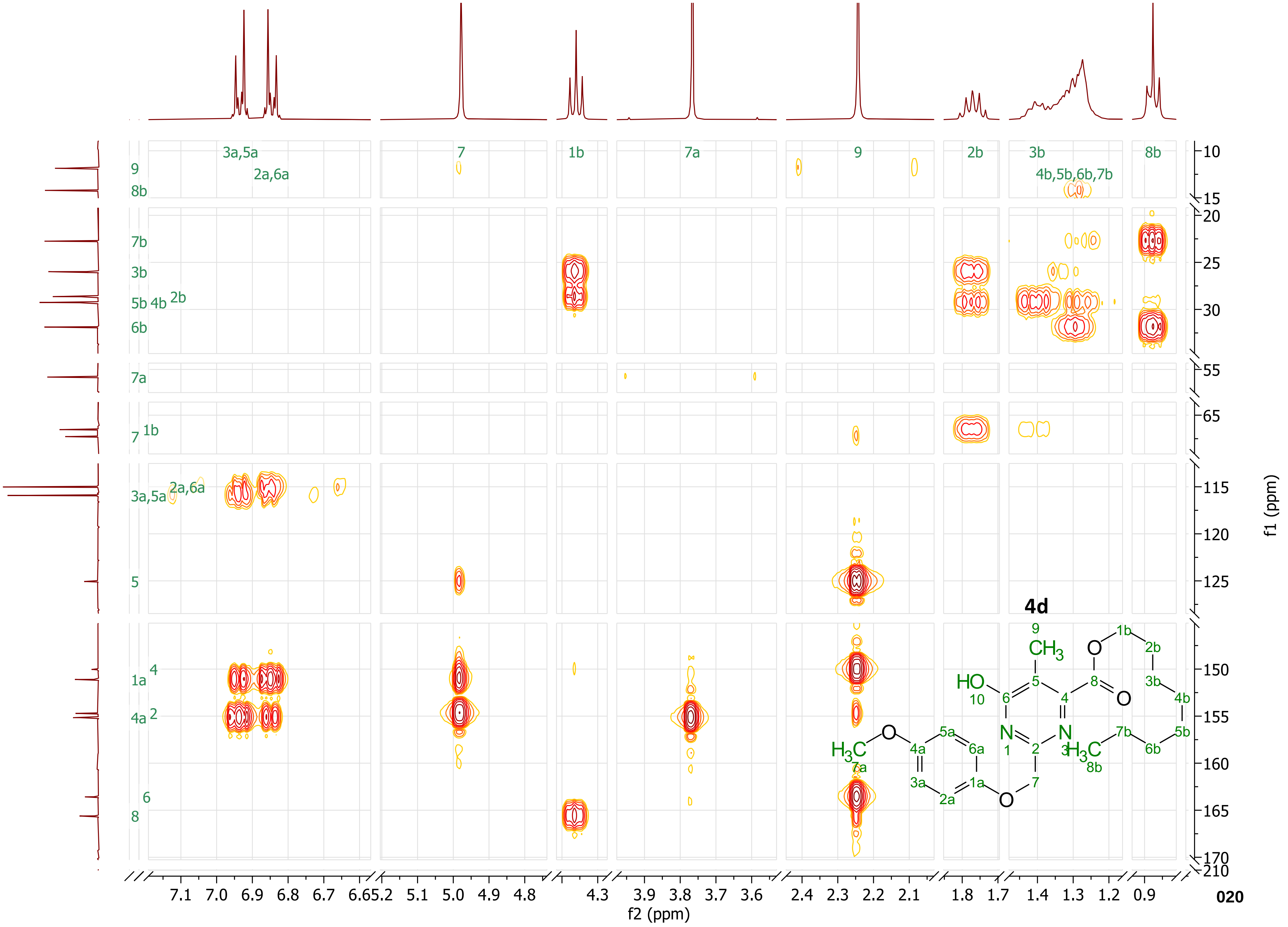

3a,5a
2a,6a
7
1b
7a
9
2b
3b
4b,5b,6b,7b
8b
7b
5b 4b 2b
6b
1b
2a,6a
3a,5a
5
4
1a
4a
2
6
8
4d
CH3
HO
10
H3C
7a
f2 (ppm)
f1 (ppm)

15N HMBC

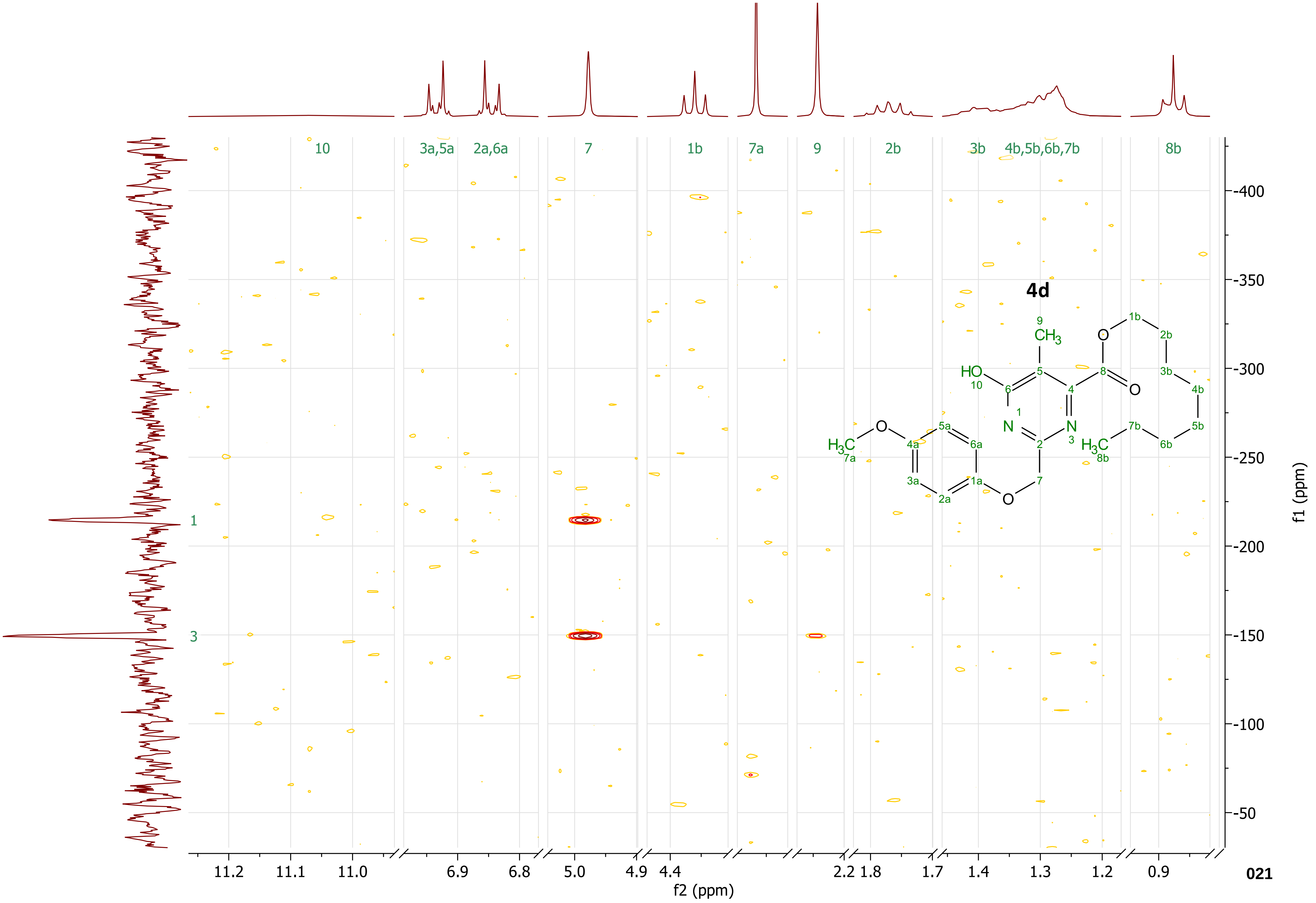

021

15N HMBC - f1 internal projection ^{15}N NMR (41 MHz, $CDCl_3$) δ -149.53, -214.70.

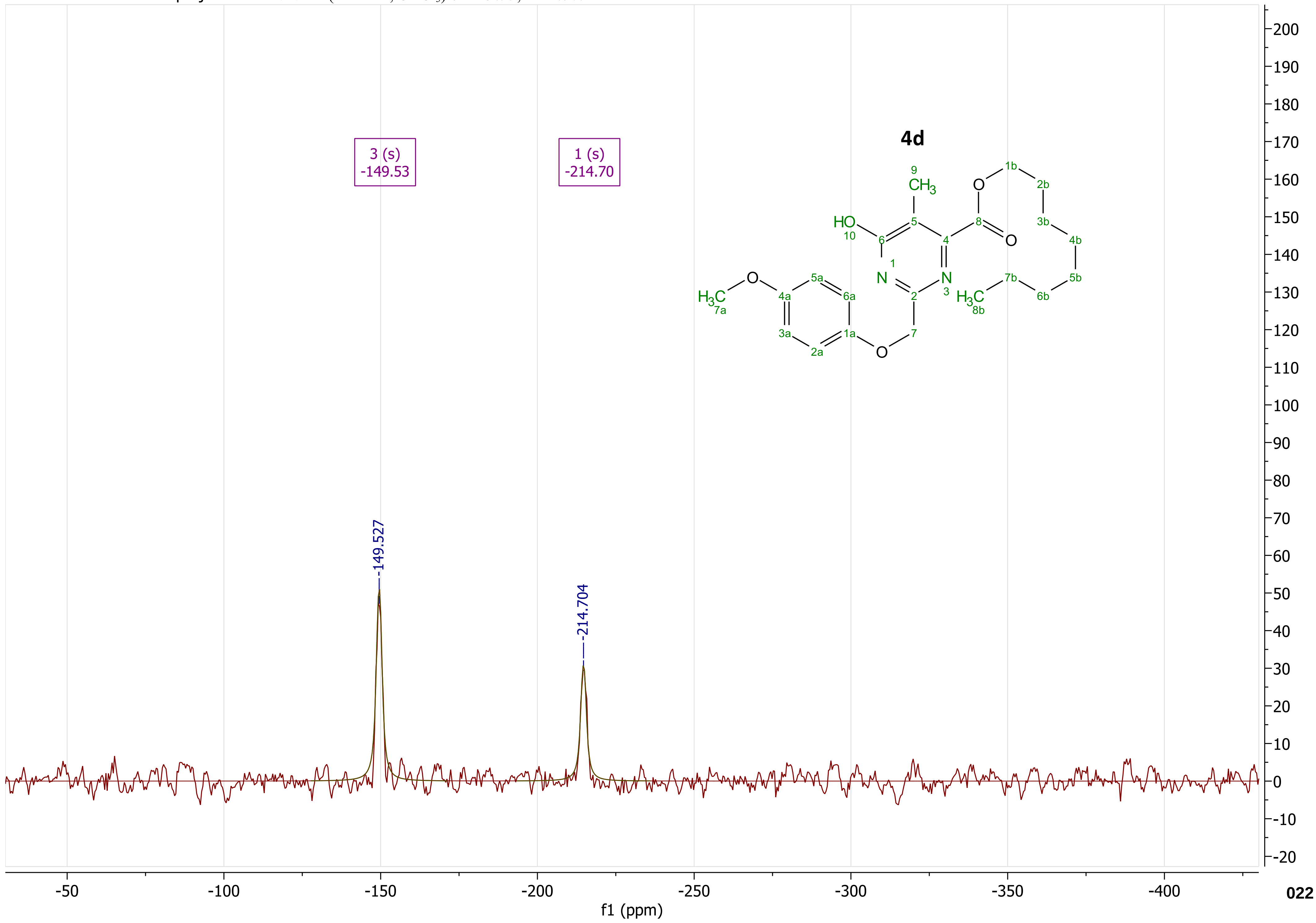

1H ^{1}H NMR (400 MHz, $CDCl_3$) δ 10.86 (br s, 1H), 6.96 – 6.89 (m, 2H), 6.89 – 6.82 (m, 2H), 4.98 (s, 2H), 4.36 (t, J = 6.9 Hz, 2H), 3.77 (s, 3H), 2.25 (s, 3H), 1.83 – 1.71 (m, 2H), 1.45 – 1.37 (m, 2H), 1.37 – 1.23 (m, 10H), 0.87 (app t, J = 6.9 Hz, 3H).

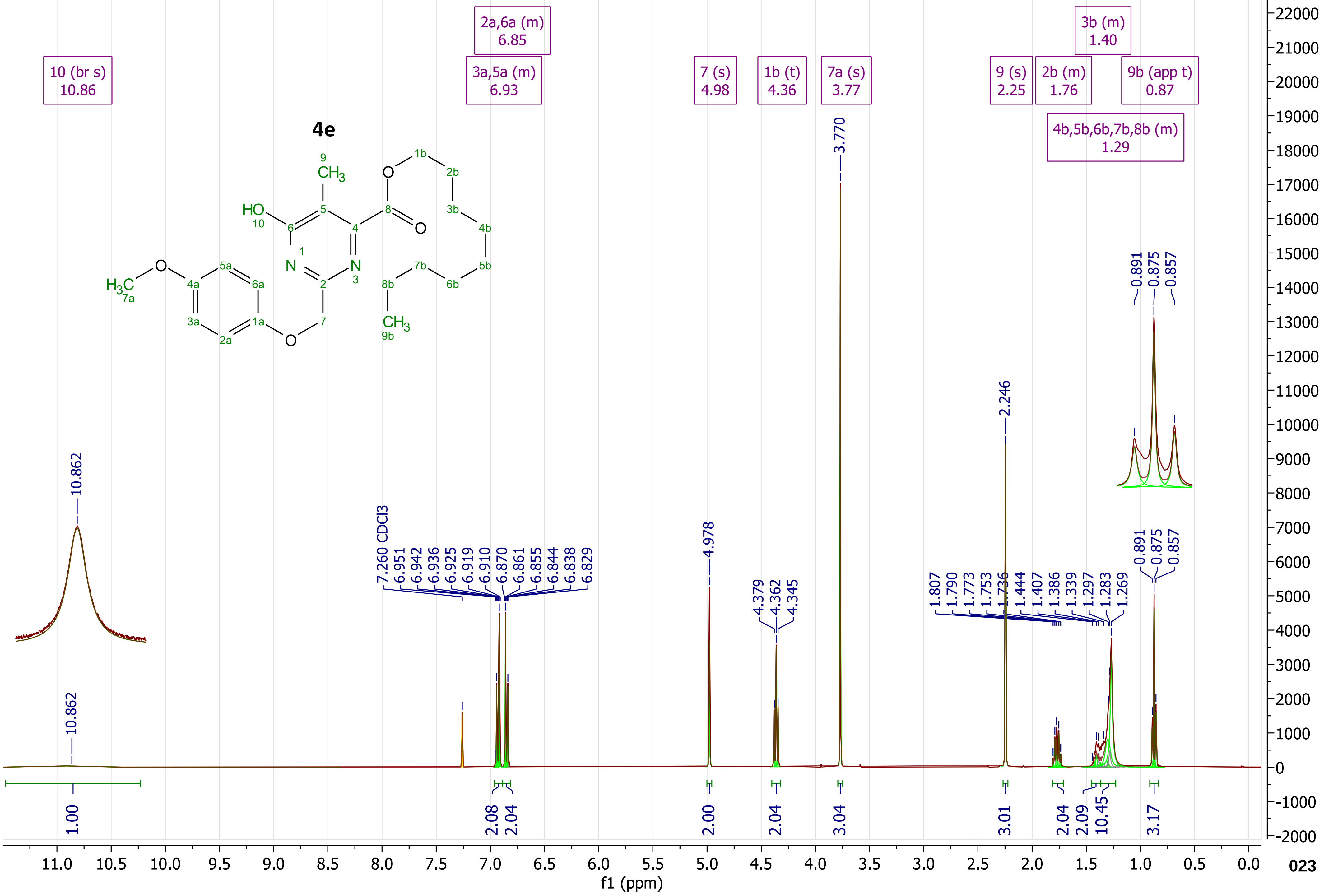

13C ^{13}C NMR (101 MHz, $CDCl_3$) δ 165.6, 163.5, 155.2, 154.7, 151.1, 150.8, 125.1, 115.9 (sym, 2C), 115.0 (sym, 2C), 67.3, 66.5, 55.8, 32.0, 29.6, 29.3 (2C), 28.7, 26.0, 22.8, 14.2, 11.9

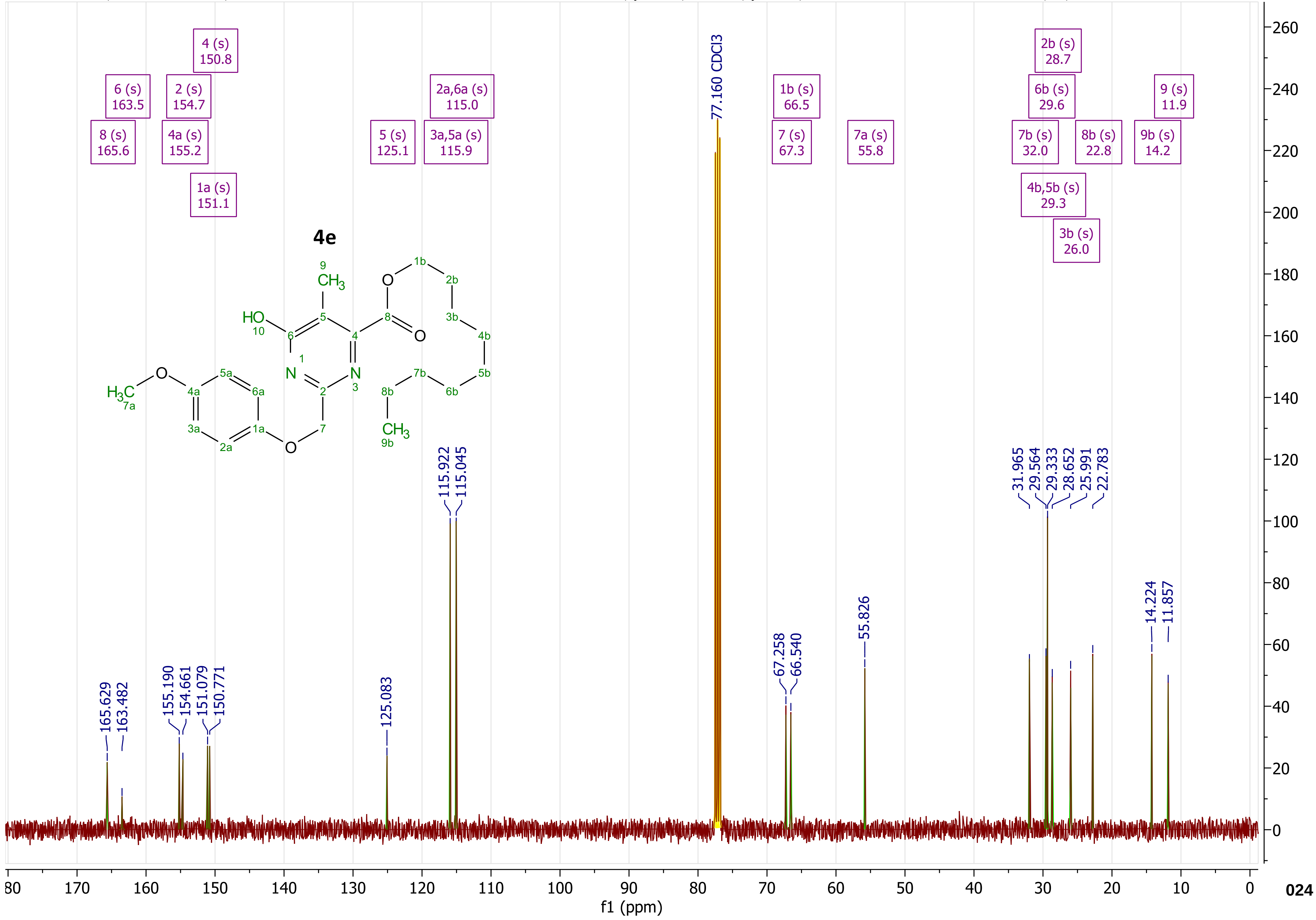

13C HSQC

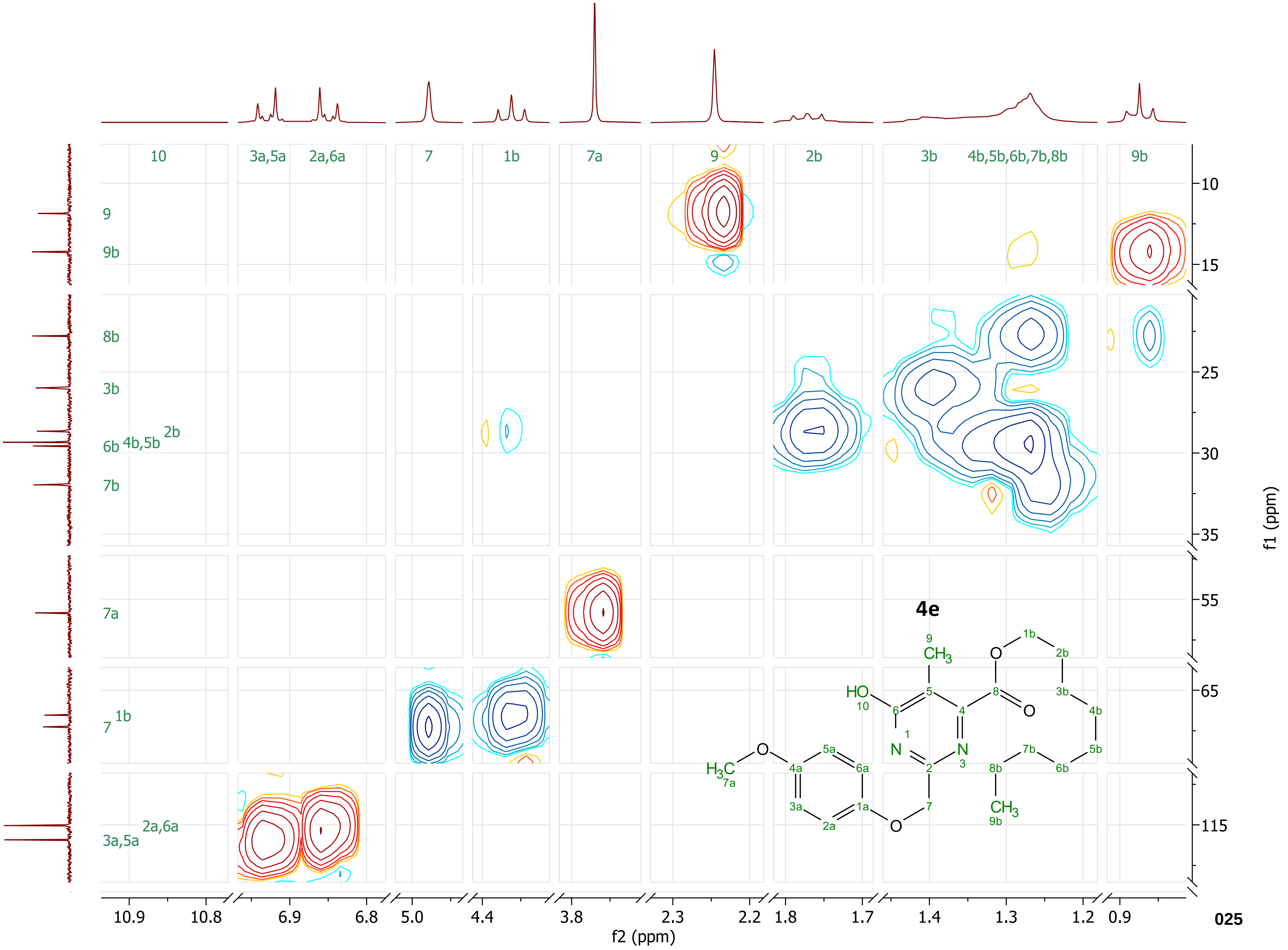

10
3a,5a
2a,6a
7
1b
7a
9
2b
3b
4b,5b,6b,7b,8b
9b
8b
6b
4b,5b
7b
3a,5a
4e
f1 (ppm)
f2 (ppm)

13C HMBC

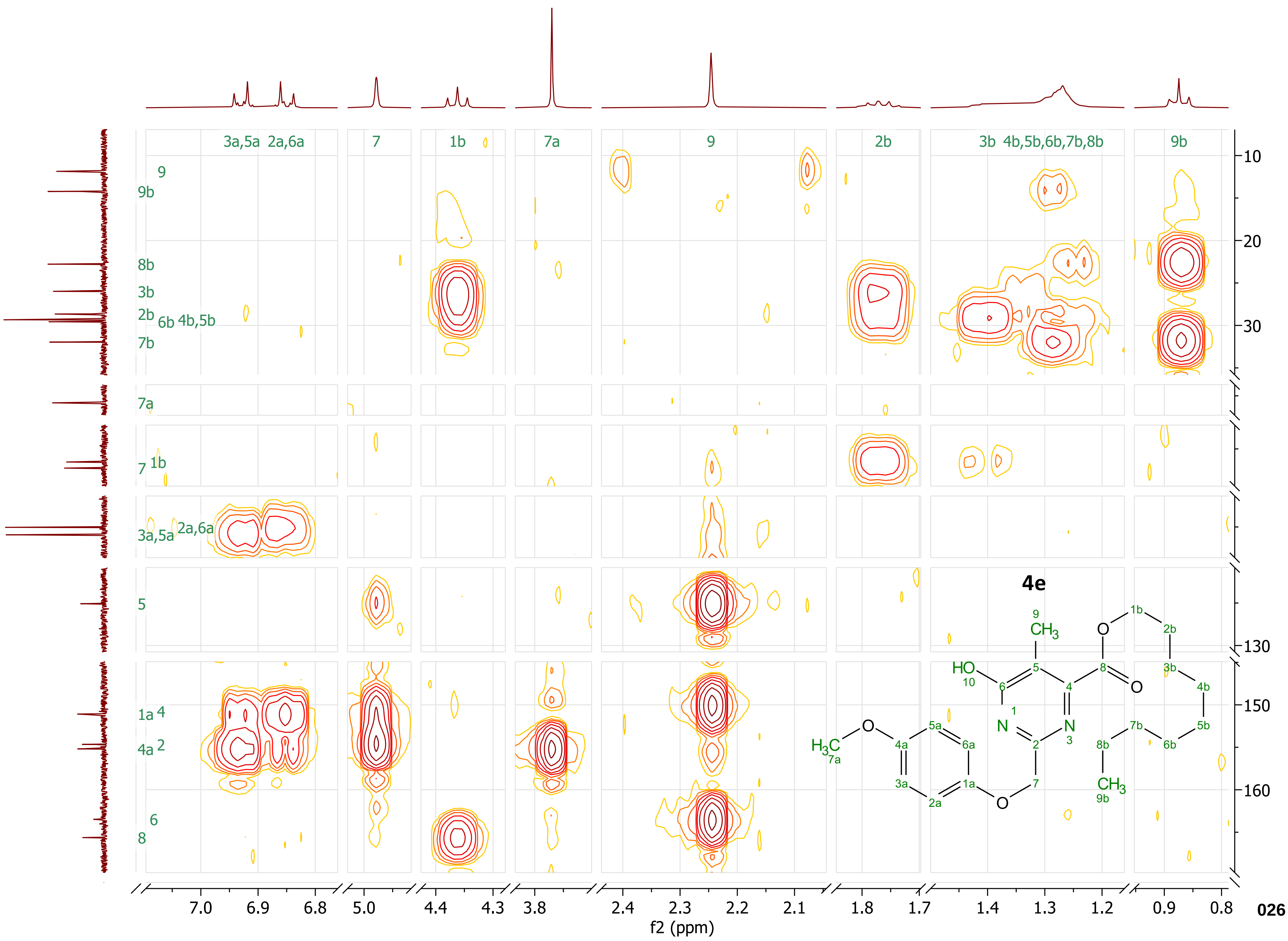

3a,5a 2a,6a
7
1b
7a
9
2b
3b 4b,5b,6b,7b,8b
9b
8b
3b
2b 6b 4b,5b
7b
7a
7 1b
3a,5a 2a,6a
5
1a 4
4a 2
6
8
4e
f2 (ppm)
f1 (ppm)

15N HMBC

^{15}N NMR (41 MHz, $CDCl_3$) δ -149.28, -215.40

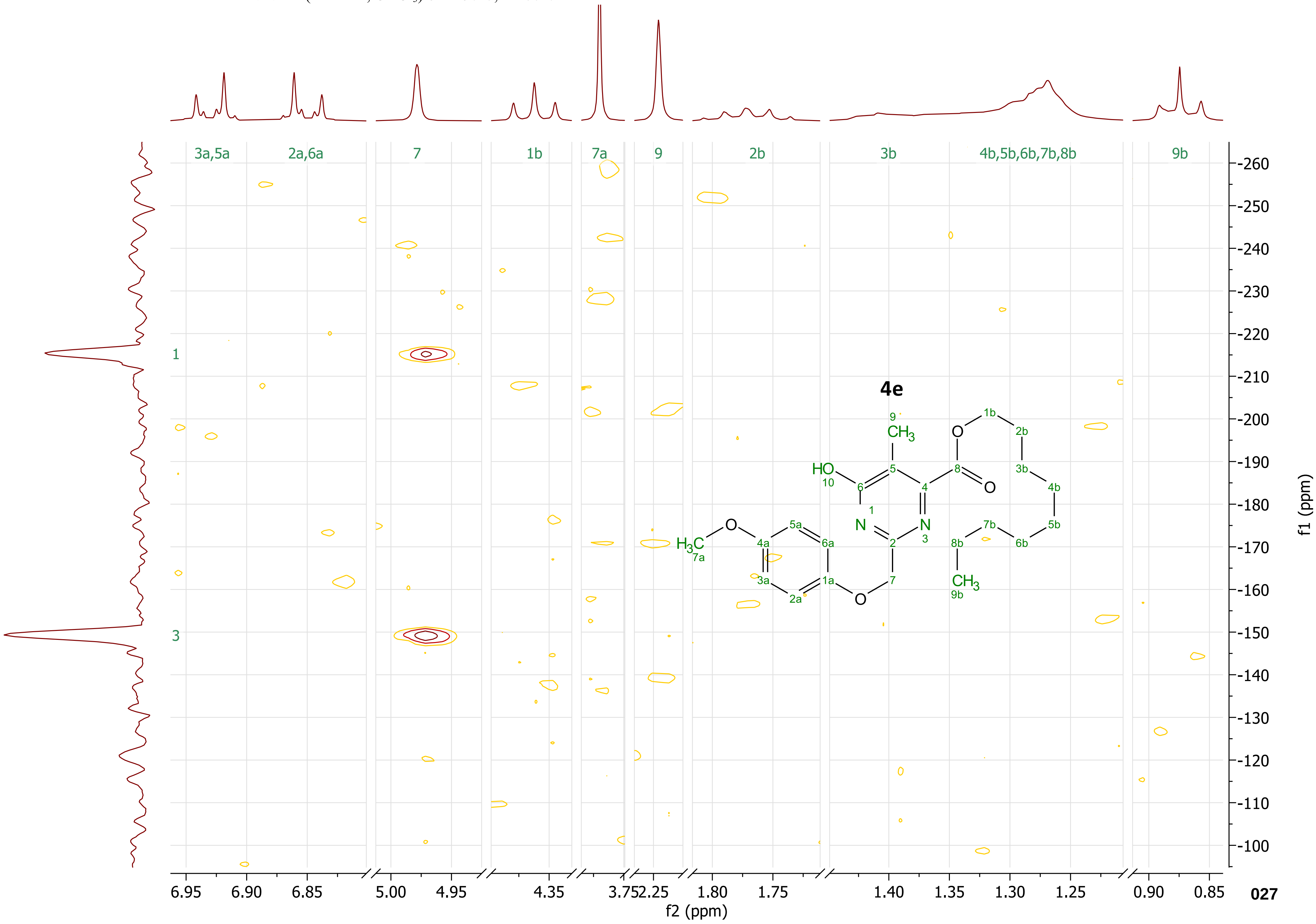

15N HMBC - f1 internal projection ^{15}N NMR (41 MHz, $CDCl_3$) δ -149.32, -215.37.

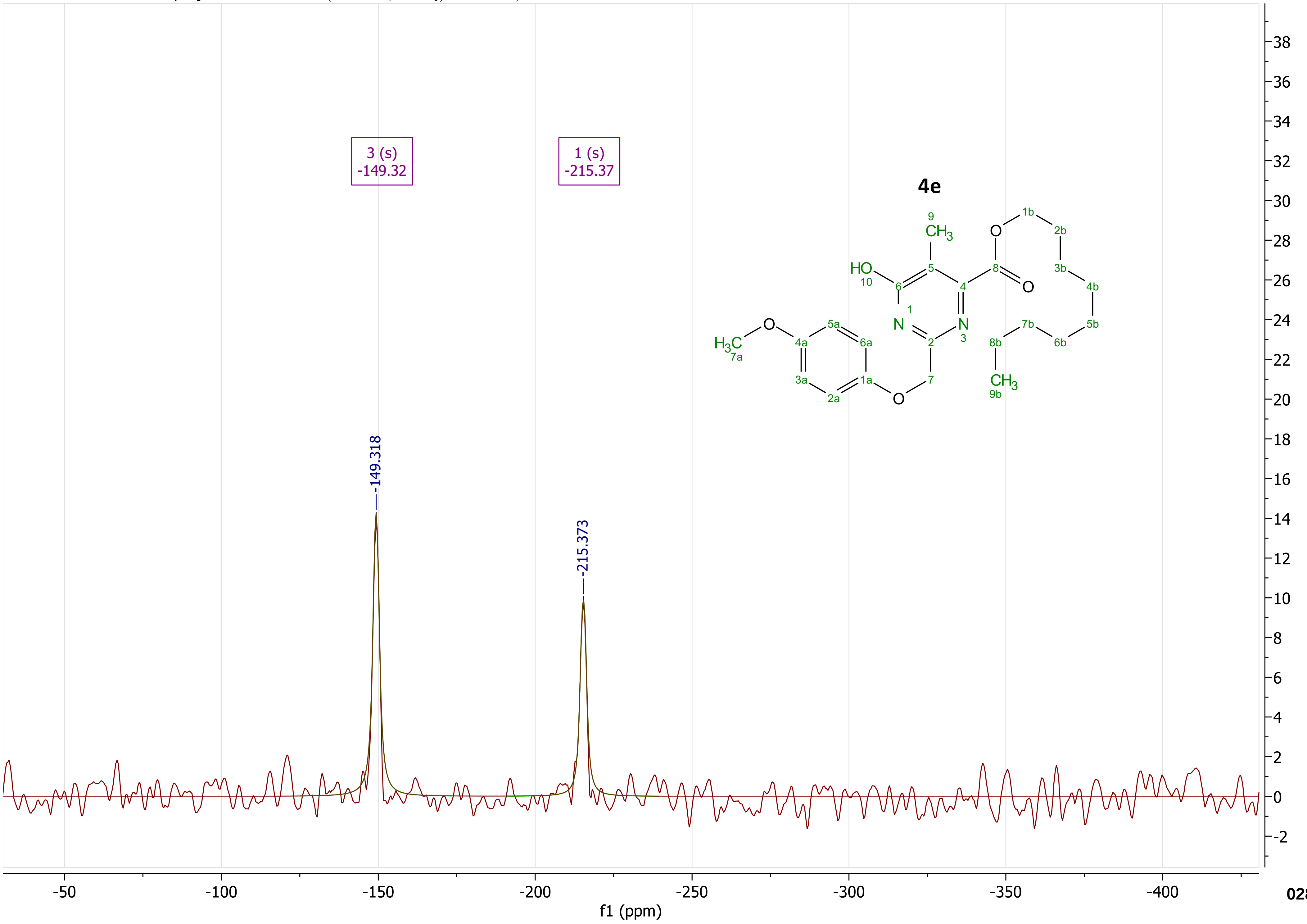

1H ^{1}H NMR (400 MHz, $CDCl_3$) δ 10.44 (br s, 1H), 7.73 (br s, 1H), 7.67 – 7.61 (m, 2H), 7.63 – 7.59 (m, 1H), 7.55 – 7.50 (m, 1H), 6.96 – 6.89 (m, 2H), 6.89 – 6.81 (m, 2H), 5.45 (s, 2H), 4.98 (s, 2H), 3.77 (s, 3H), 2.24 (s, 3H).

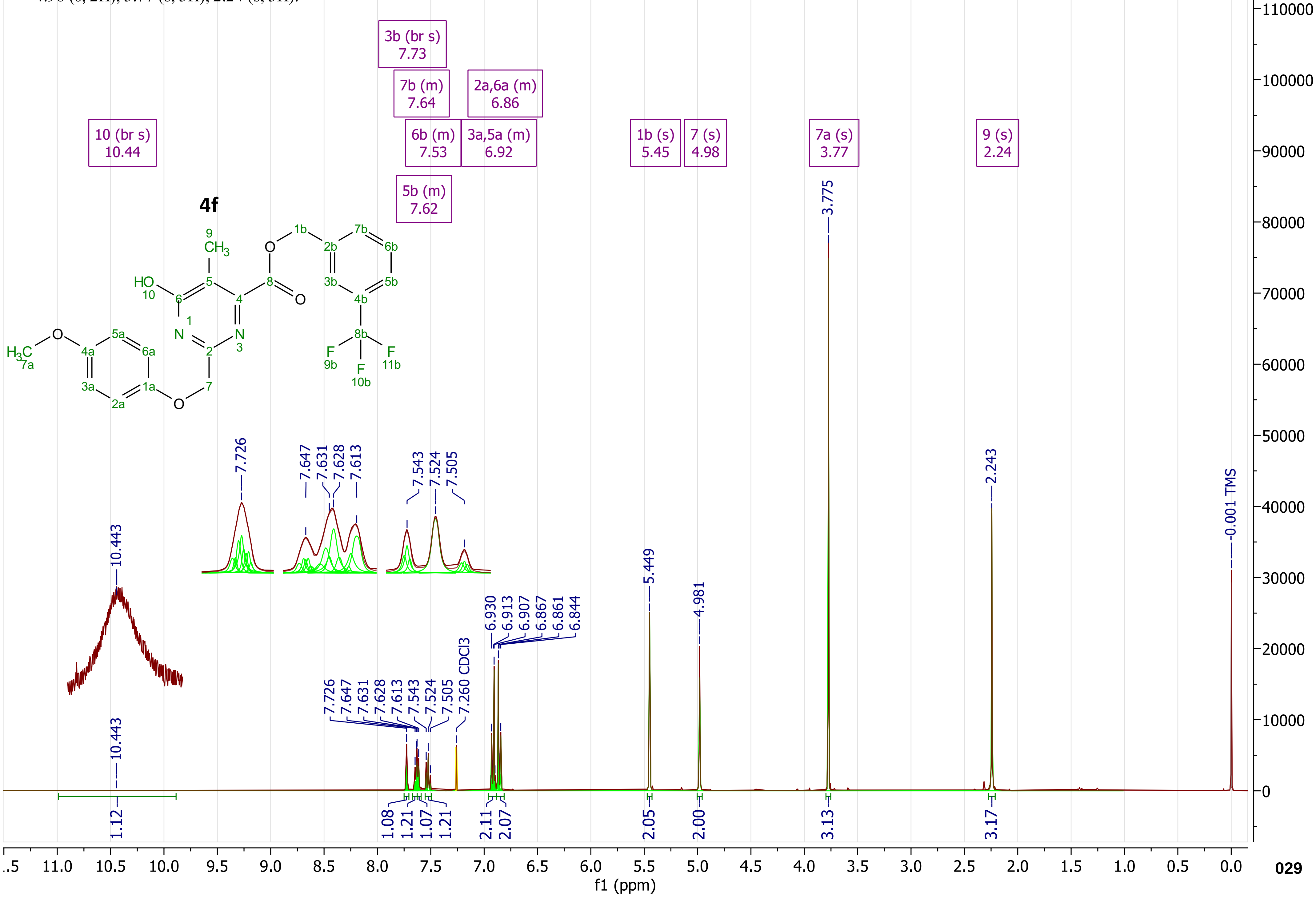

13C ^{1}H NMR (400 MHz, $CDCl_3$) δ 10.4 (br s, 1H), 7.7 (br s, 1H), 7.7 – 7.6 (m, 2H), 7.6 – 7.6 (m, 1H), 7.6 – 7.5 (m, 1H), 7.0 – 6.9 (m, 2H), 6.9 – 6.8 (m, 2H), 5.4 (s, 2H), 5.0 (s, 2H), 3.8 (s, 3H), 2.2 (s, 3H).

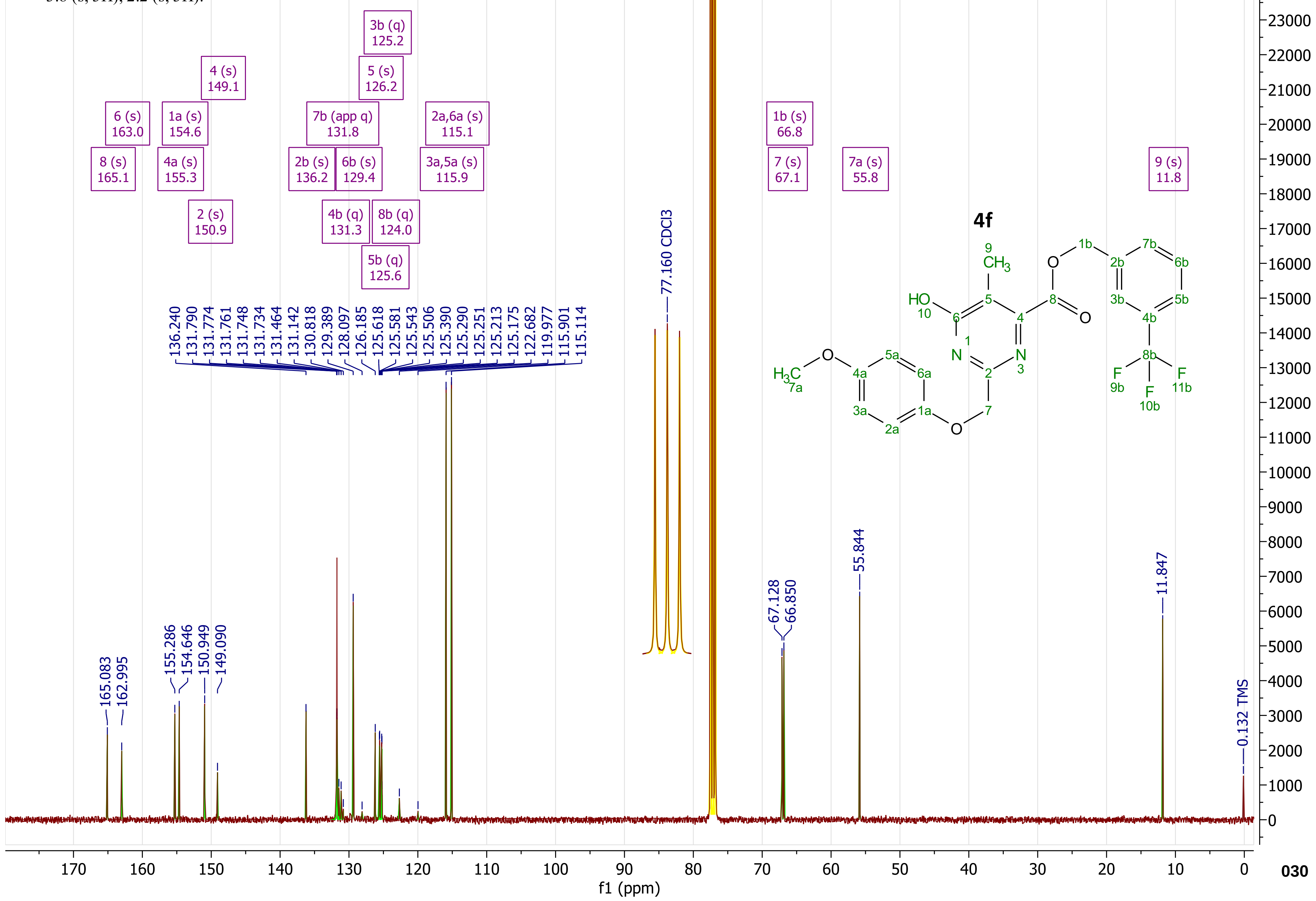

13C — 133–119 ppm

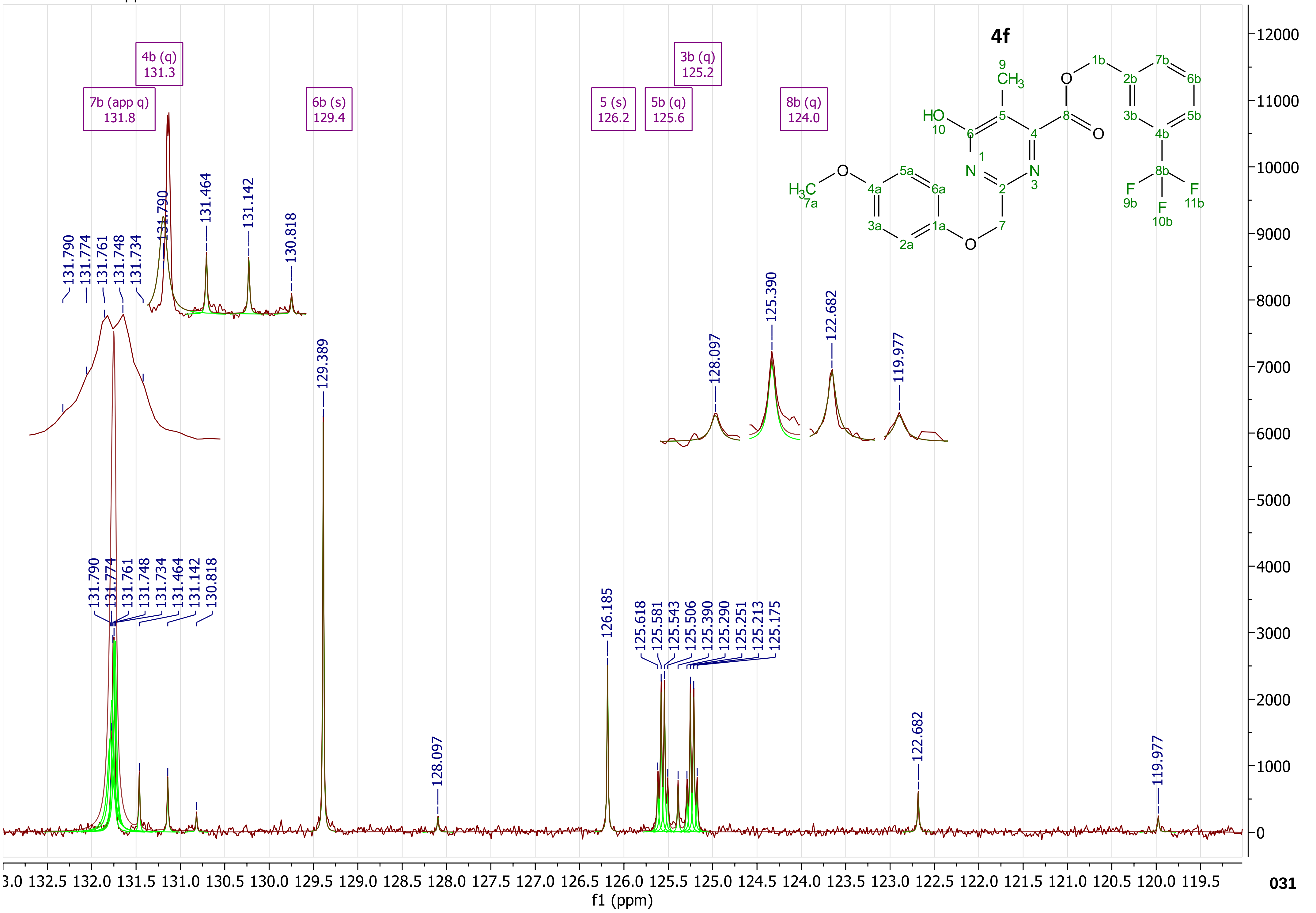

13C HSQC

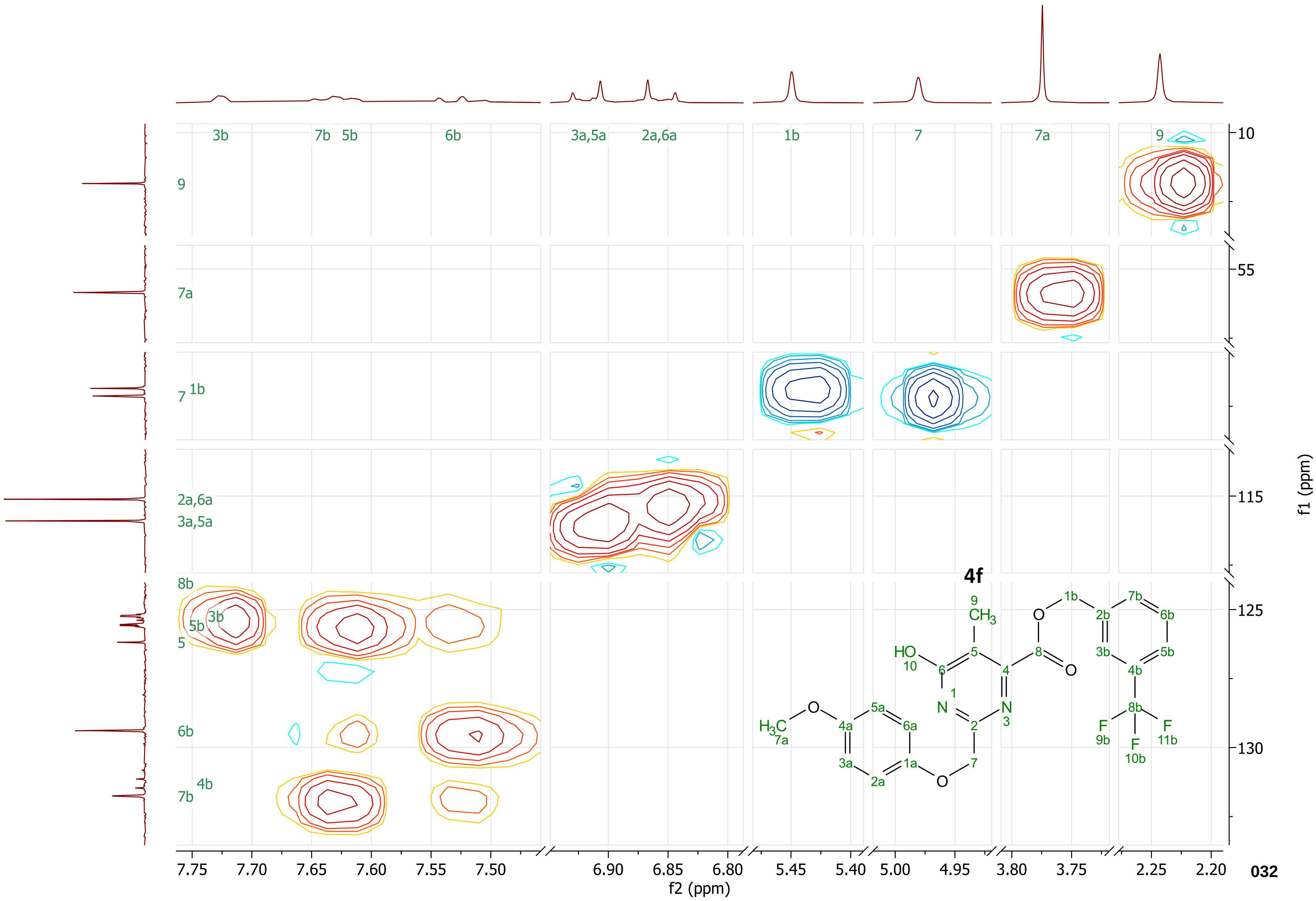
3b
7b
5b
6b
3a,5a
2a,6a
1b
7
7a
9
8b
5b
3b
5
6b
4b
7b
2a,6a
3a,5a
f2 (ppm)
f1 (ppm)
4f
HO
10
CH3
H3C
7a
F
9b
F
10b
F
11b

032

13C HMBC

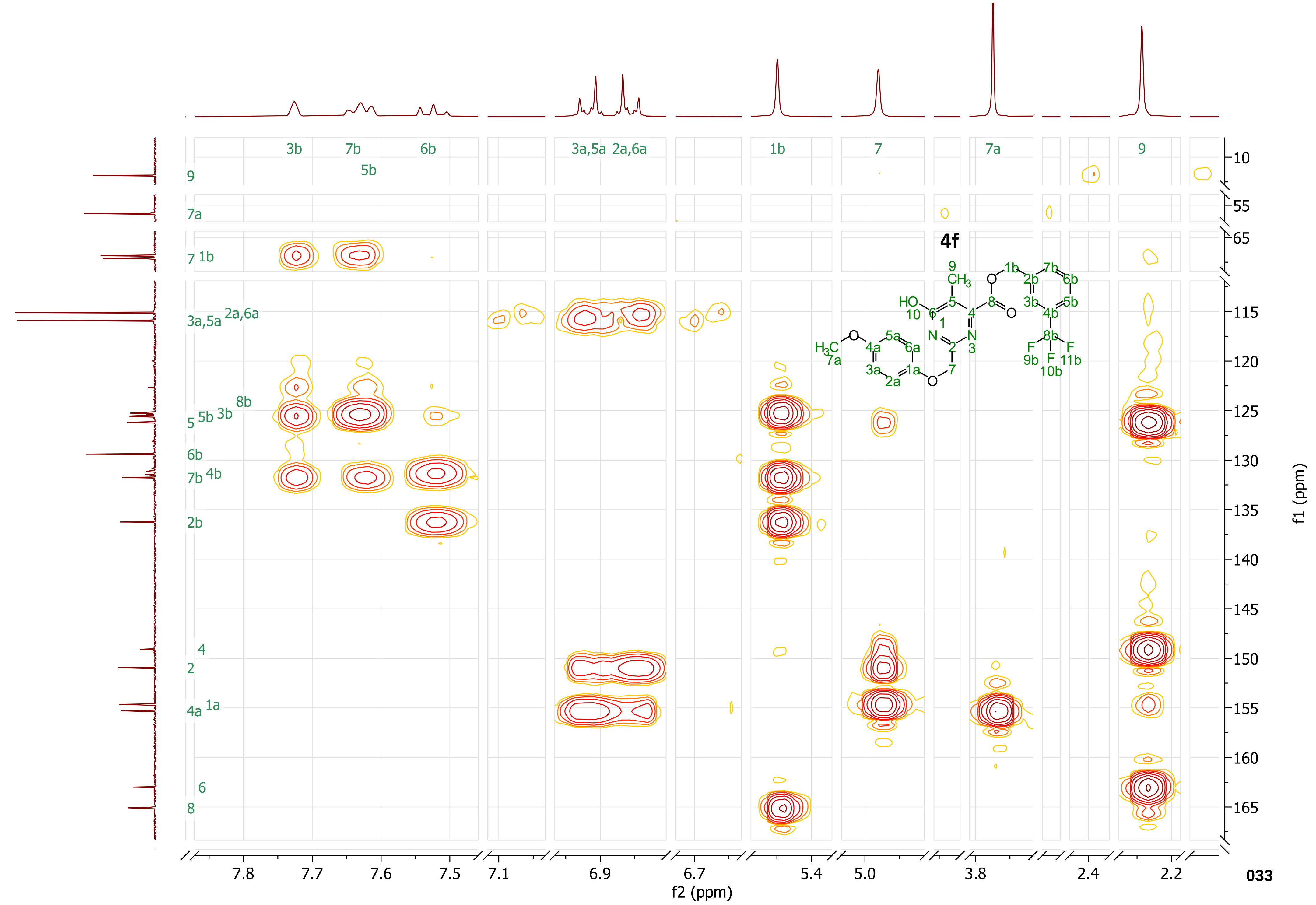

4f
3b
7b
6b
5b
3a,5a 2a,6a
1b
7
7a
9
1b
3a,5a 2a,6a
5 5b 3b 8b
6b
7b 4b
2b
4
2
4a 1a
6
8
f2 (ppm)
f1 (ppm)

15N HMBC

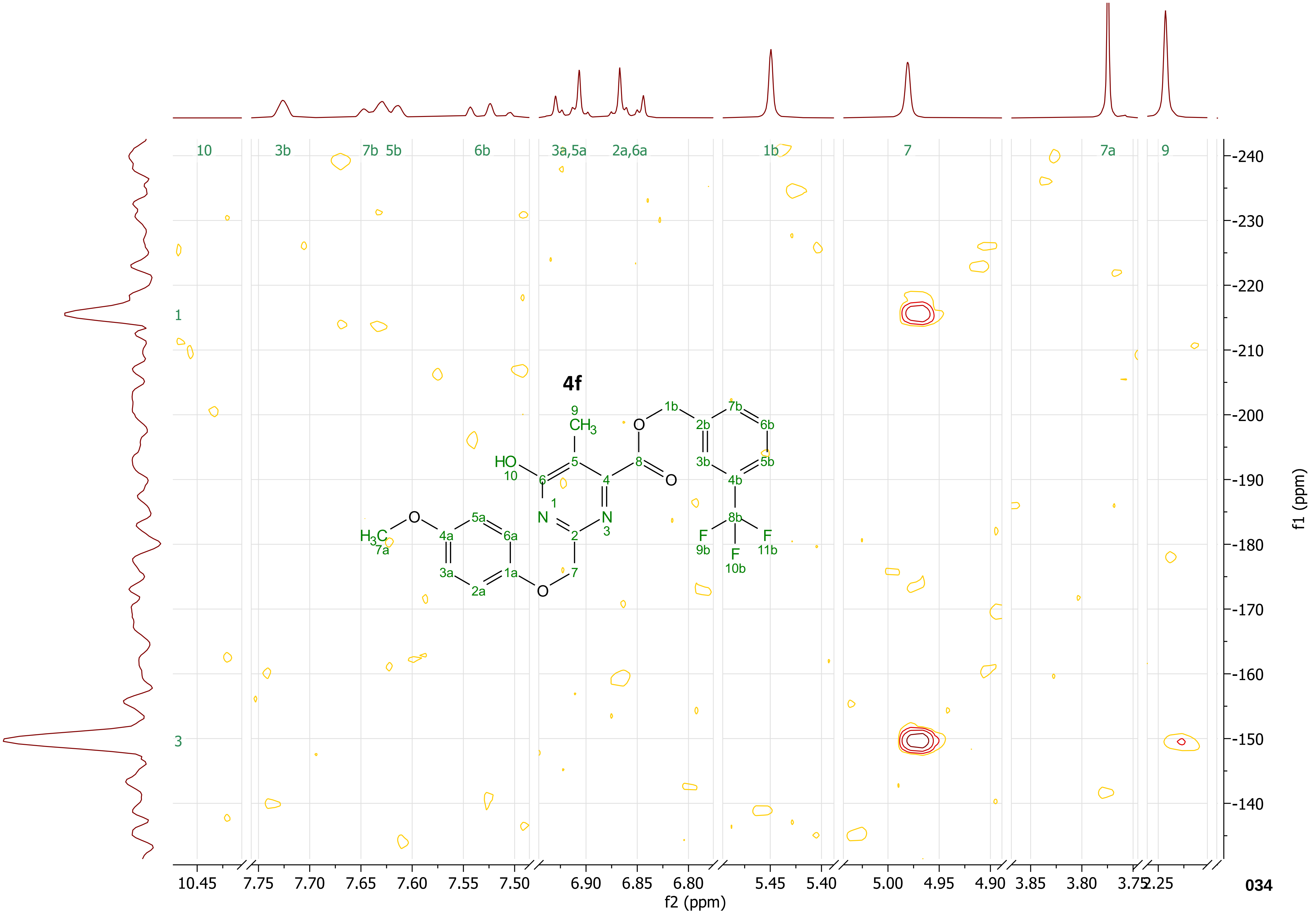

15N HMBC - f1 internal projection ^{15}N NMR (41 MHz, $CDCl_3$) δ -149.70, -215.52.

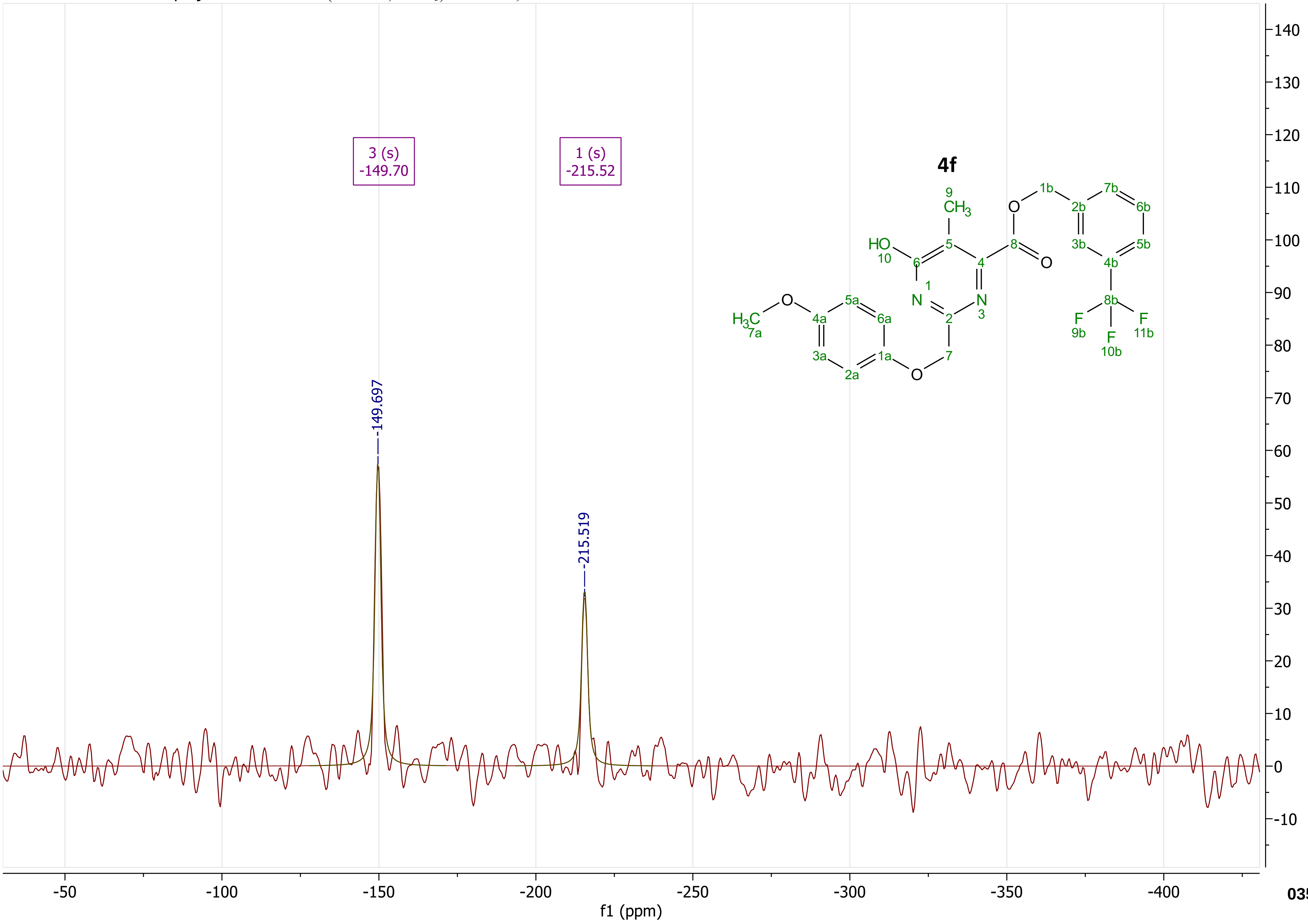

19F ^{19}F NMR (376 MHz, $CDCl_3$) δ -62.71.

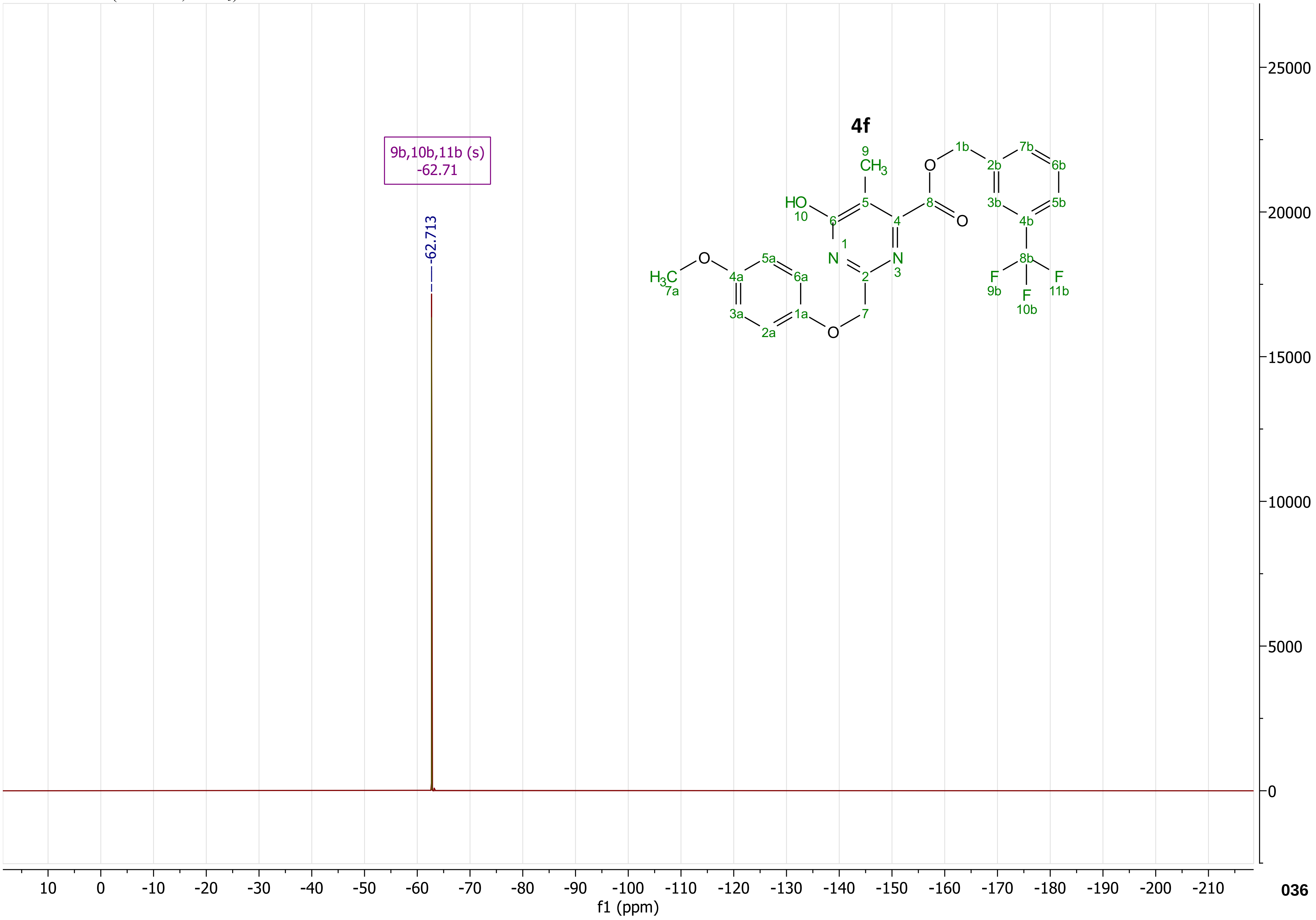

1H ^{1}H NMR (400 MHz, $CDCl_3$) δ 6.99 – 6.88 (m, 2H), 6.88 – 6.72 (m, 2H), 5.13 (s, 2H), 4.38 (t, *J* = 6.8 Hz, 2H), 4.36 (t, *J* = 6.6 Hz, 2H), 3.75 (s, 3H), 2.26 (s, 3H), 1.86 – 1.72 (m, 2H), 1.74 – 1.62 (m, 2H), 1.55 – 1.31 (m, 4H), 0.96 (t, *J* = 7.4 Hz, 3H), 0.94 (t, *J* = 7.4 Hz, 3H).

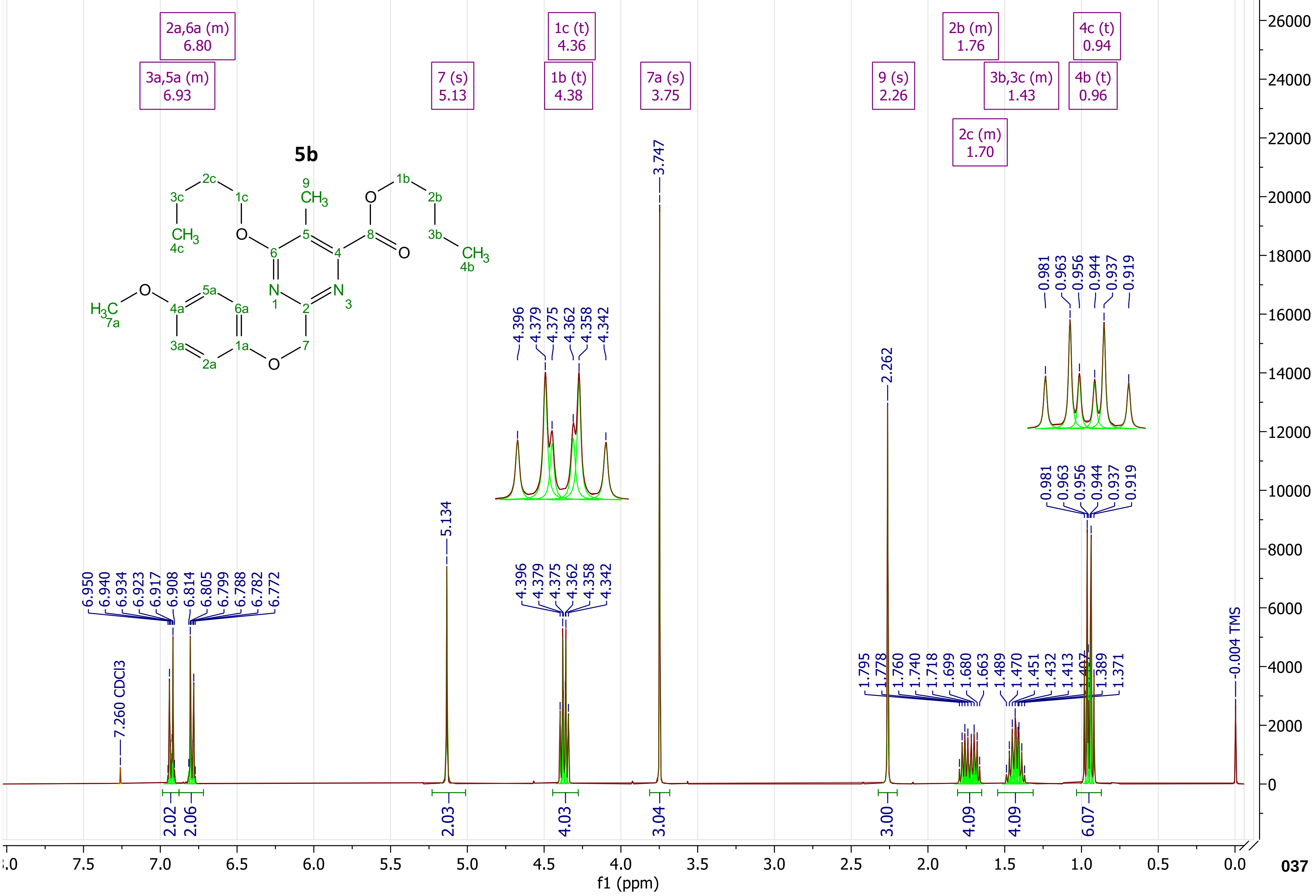

13C ^{13}C NMR (101 MHz, $CDCl_3$) δ 169.3, 165.9, 163.1, 155.1, 154.1, 152.9, 116.6, 116.1 (sym, 2C), 114.6 (sym, 2C), 71.2, 67.3, 66.1, 55.8, 30.8, 30.7, 19.28, 19.27, 13.9, 13.8, 11.1.

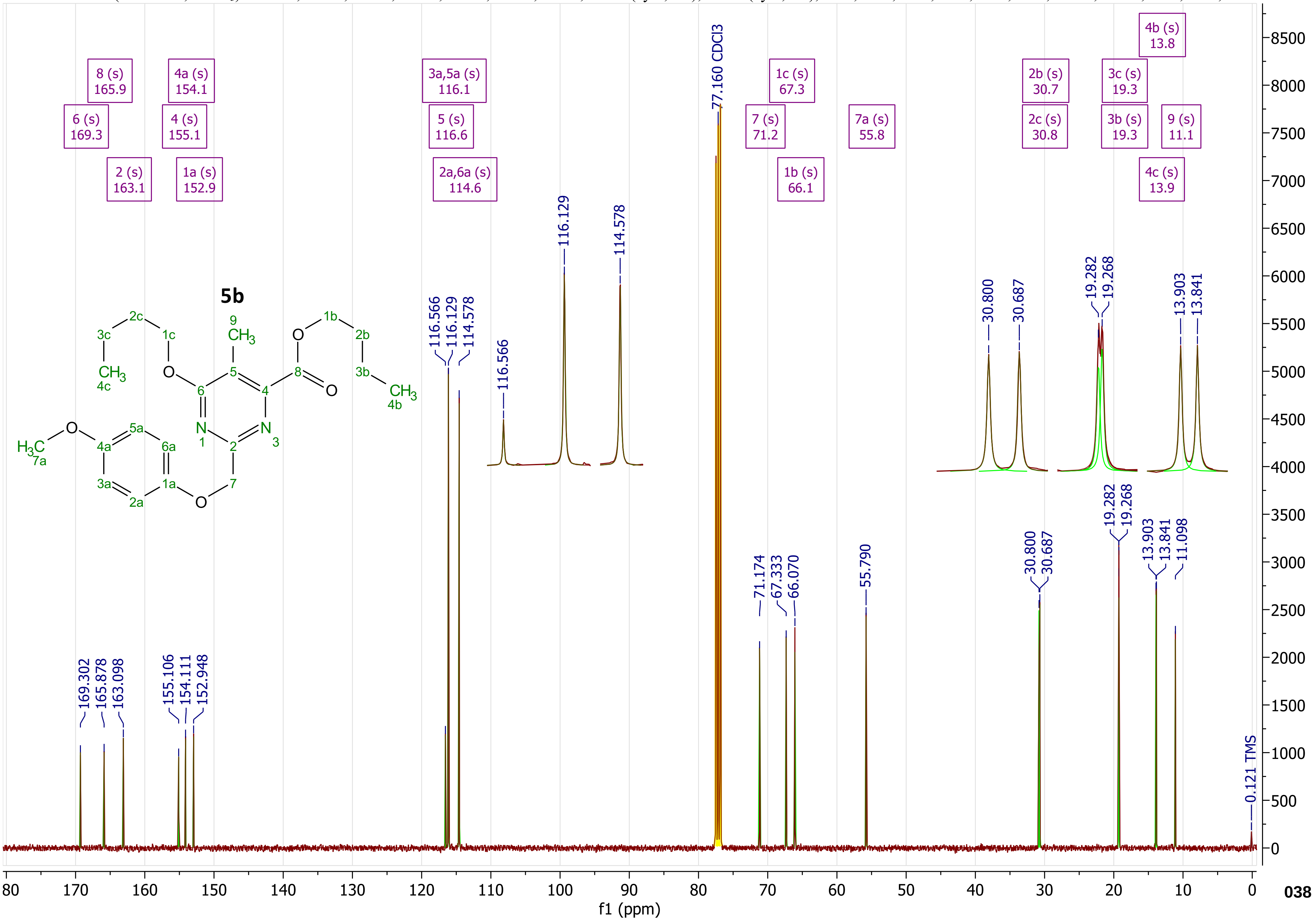

13C HSQC

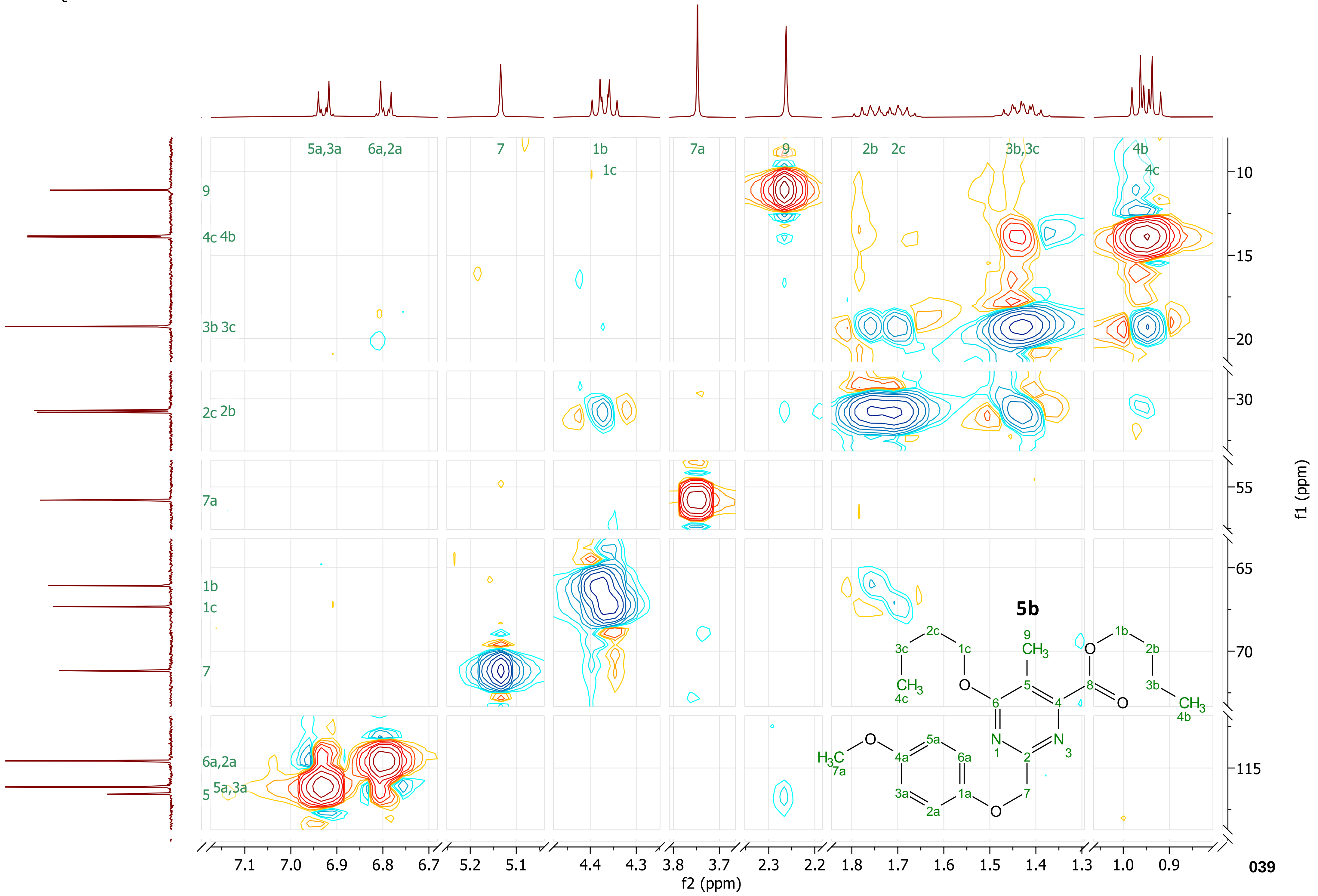
5a,3a
6a,2a
7
1b
1c
7a
9
2b
2c
3b,3c
4b
4c
4c 4b
3b 3c
2c 2b
6a,2a
5
5a,3a
5b
f2 (ppm)
f1 (ppm)

13C HMBC

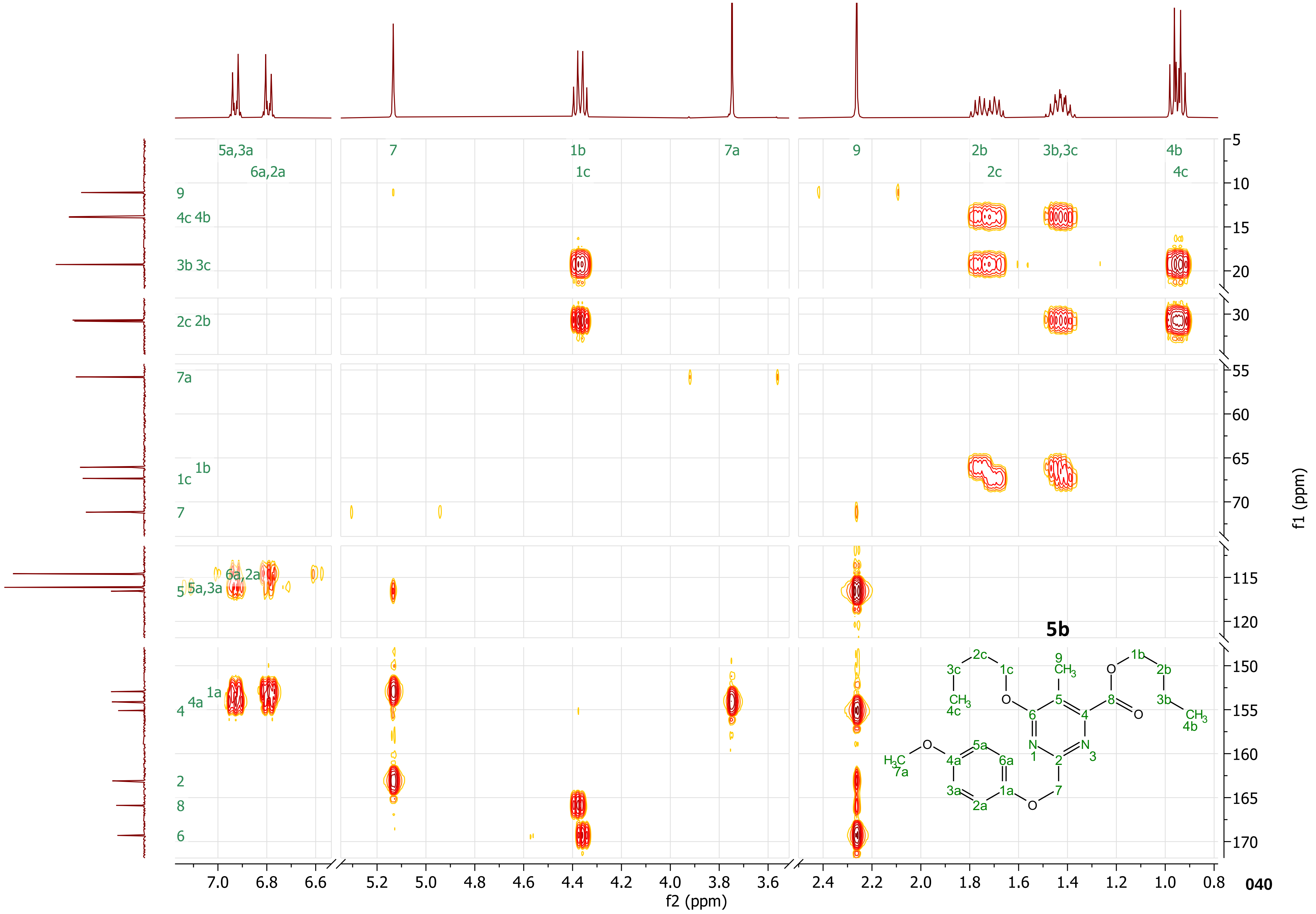

5a,3a
6a,2a
7
1b
1c
7a
9
2b
2c
3b,3c
4b
4c
4c 4b
3b 3c
2c 2b
1c
7
5
4
4a
1a
2
8
6
5b
f2 (ppm)
f1 (ppm)

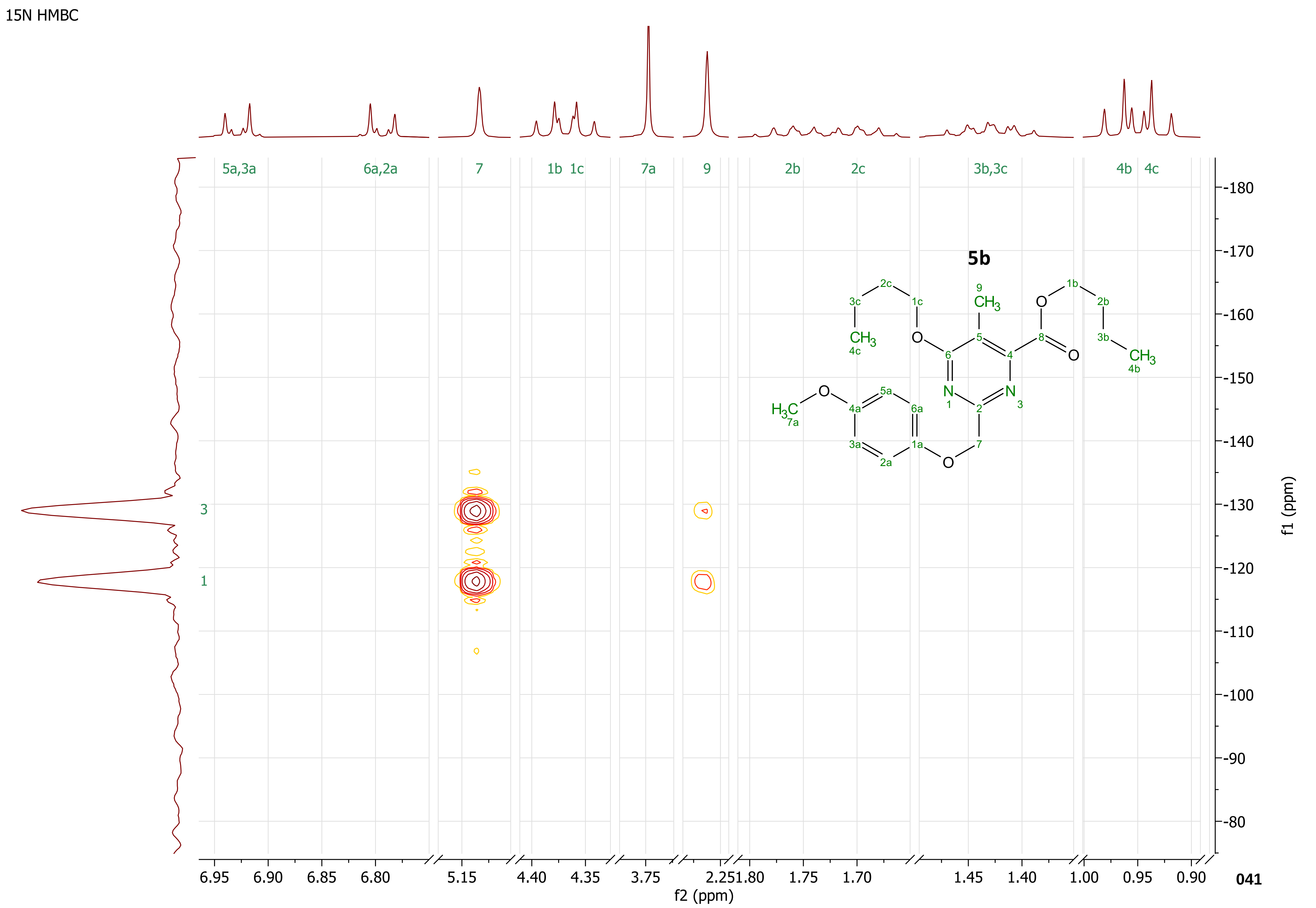
15N HMBC
5a,3a
6a,2a
7
1b 1c
7a
9
2b
2c
3b,3c
4b 4c
3
1
5b
f2 (ppm)
f1 (ppm)

15N HMBC - f1 internal projection ^{15}N NMR (41 MHz, $CDCl_3$) δ -118.0, -129.3.

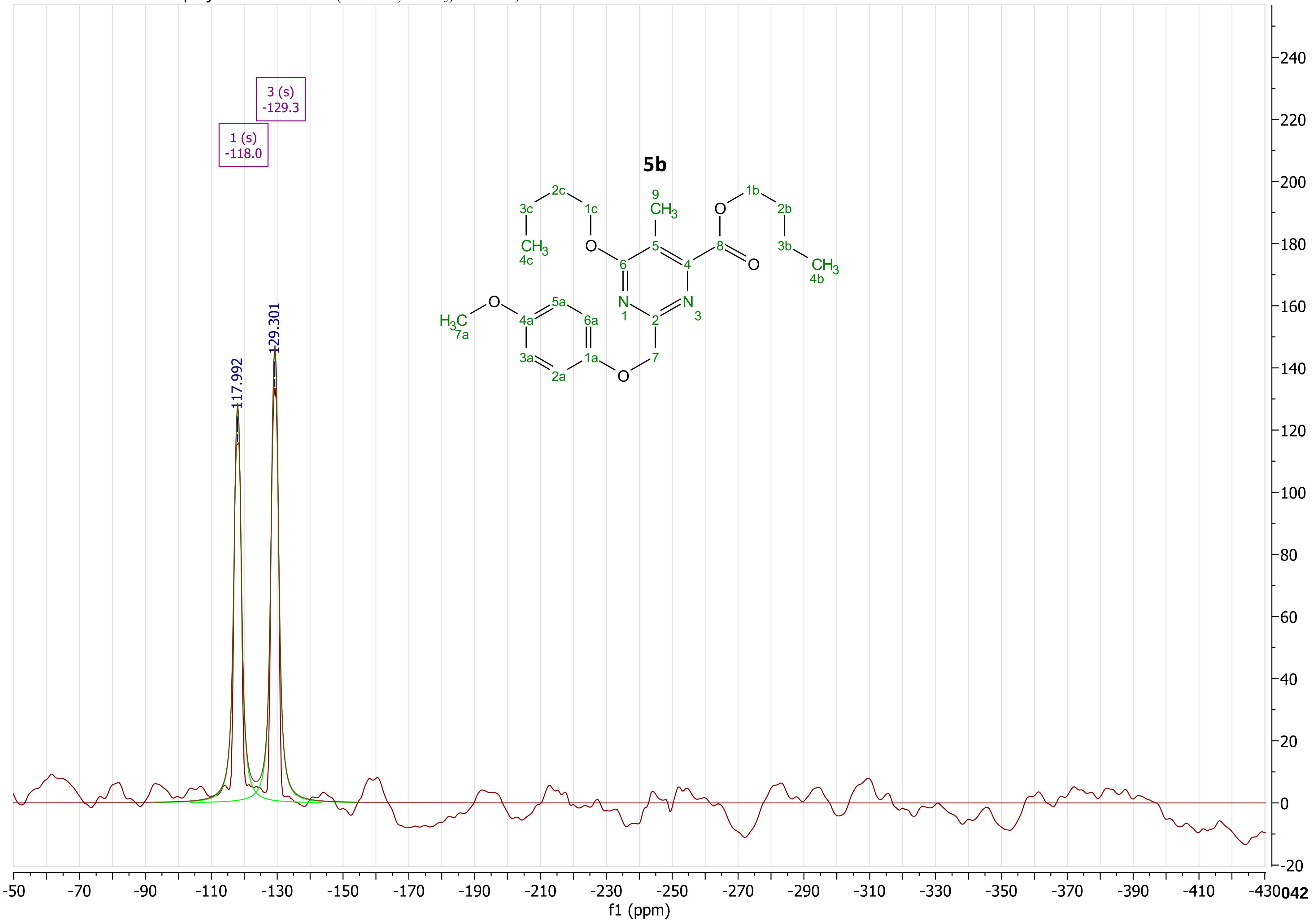

1H ^{1}H NMR (400 MHz, $CDCl_3$) δ 6.99 – 6.86 (m, 2H), 6.86 – 6.70 (m, 2H), 5.13 (s, 2H), 4.37 (t, *J* = 6.9 Hz, 2H), 4.35 (t, *J* = 6.6 Hz, 2H), 3.75 (s, 3H), 2.26 (s, 3H), 1.83 – 1.73 (m, 2H), 1.77 – 1.66 (m, 2H), 1.49 – 1.20 (m, 16H), 0.89 (app t, *J* = 6.9 Hz, 3H), 0.88 (app t, *J* = 6.9 Hz, 3H).

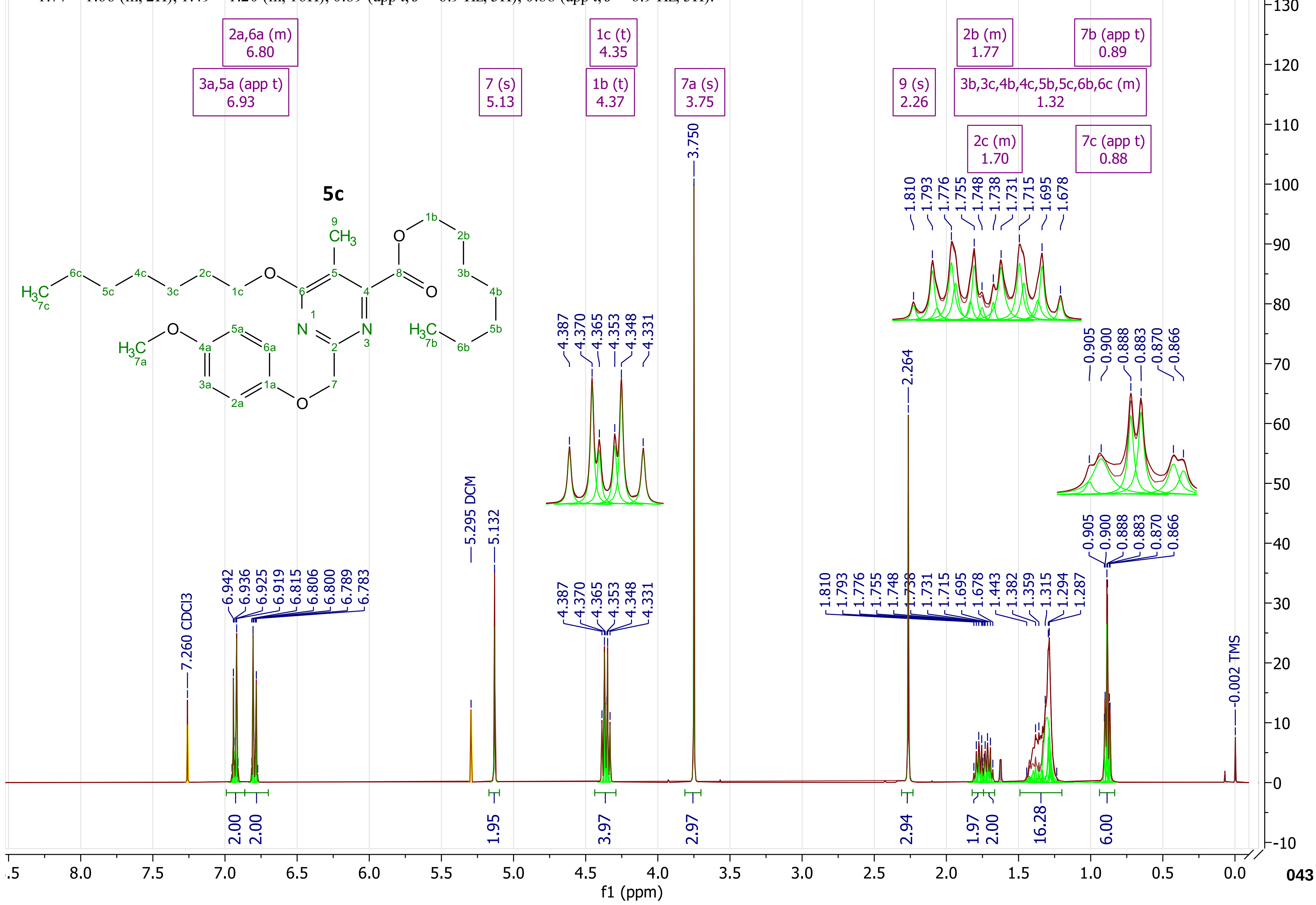

13C

^{13}C NMR (101 MHz, $CDCl_3$) δ 169.3, 165.9, 163.1, 155.1, 154.1, 153.0, 116.6 (sym, 2C), 116.1 (sym, 2C), 114.6, 71.2, 67.7, 66.4, 55.8, 31.9, 31.8, 29.1, 29.0, 28.74, 28.67, 26.03, 25.98, 22.73, 22.70, 14.21, 14.19, 11.1.

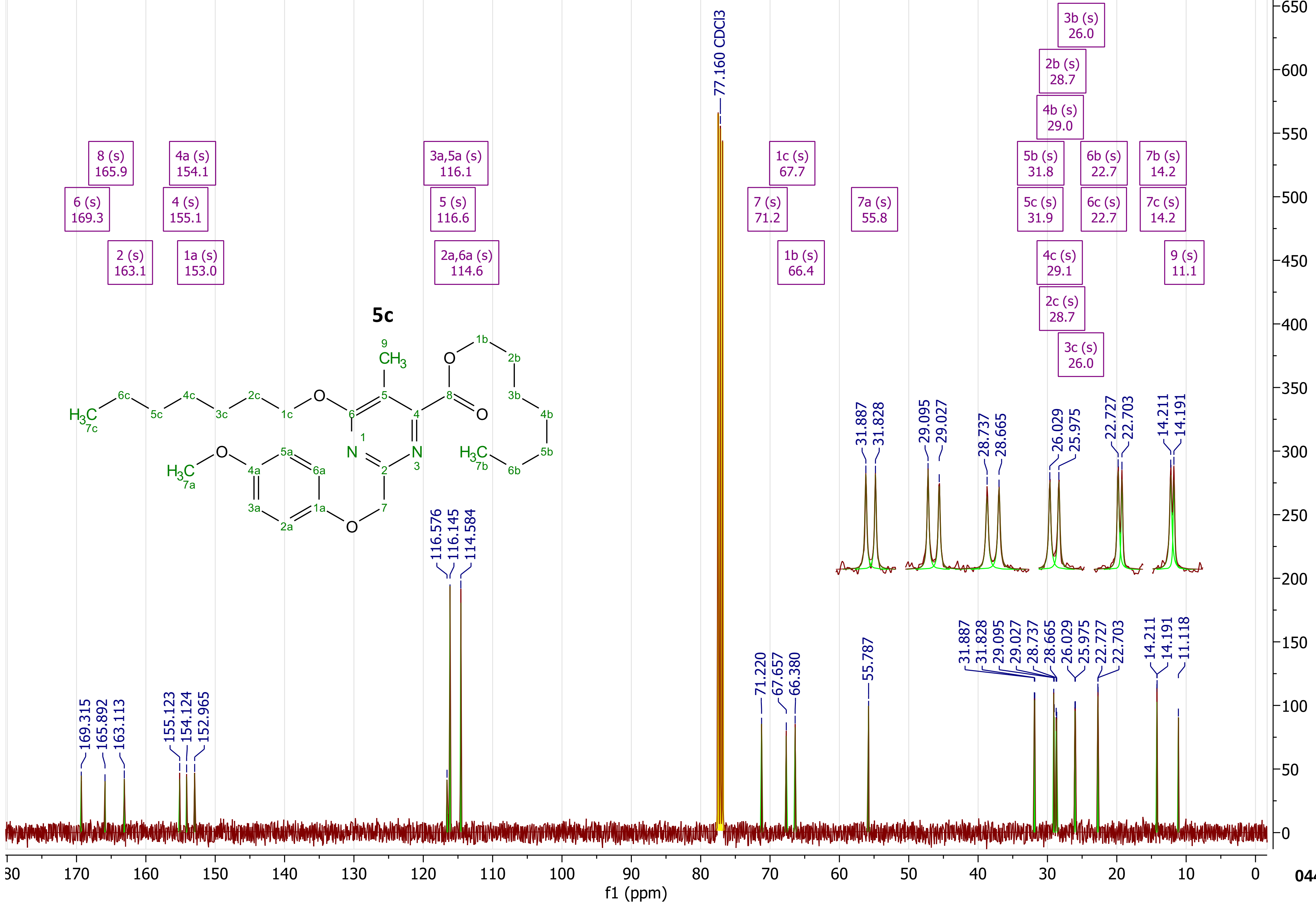

13C HSQC

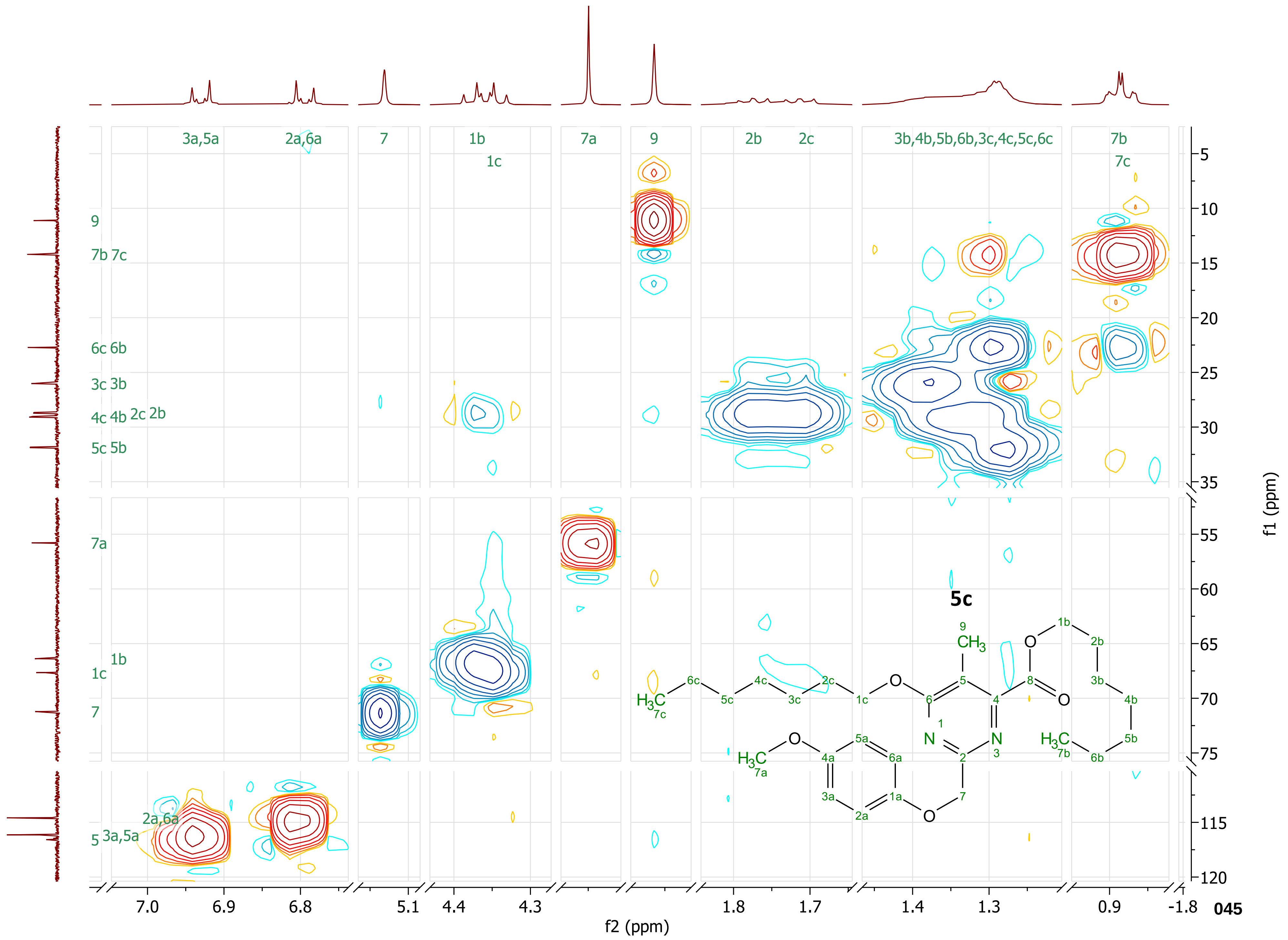
3a,5a
2a,6a
7
1b
1c
7a
9
2b
2c
3b,4b,5b,6b,3c,4c,5c,6c
7b
7c
9
7b 7c
6c 6b
3c 3b
4c 4b 2c 2b
5c 5b
7a
1b
1c
7
2a,6a
5 3a,5a
5c
f2 (ppm)
f1 (ppm)

13C HMBC

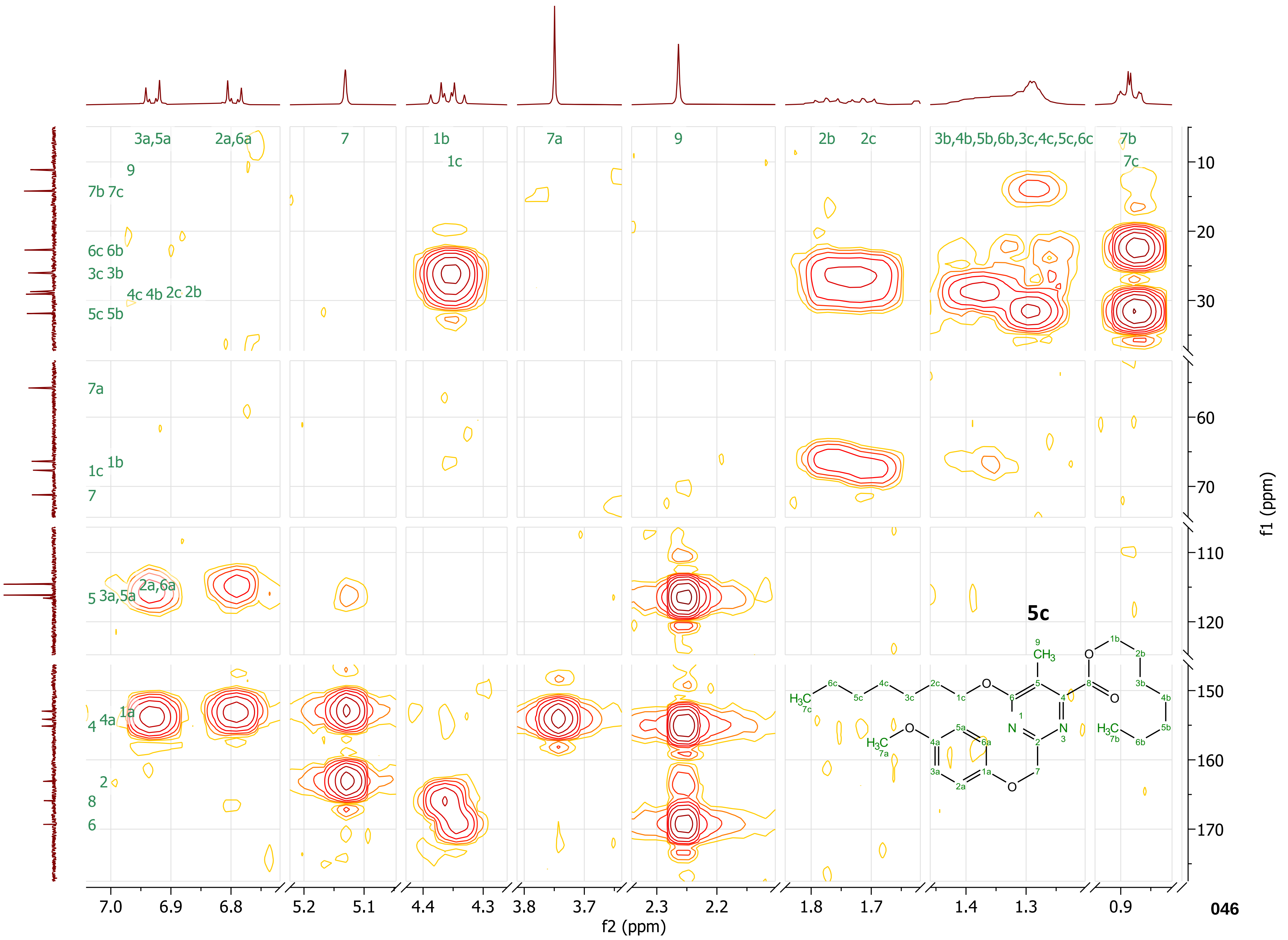

3a,5a
2a,6a
7
1b
1c
7a
9
2b
2c
3b,4b,5b,6b,3c,4c,5c,6c
7b
7c
7b 7c
6c 6b
3c 3b
4c 4b 2c 2b
5c 5b
1c 1b
5 3a,5a
4 4a 1a
2
8
6
5c
f2 (ppm)
f1 (ppm)

046

15N HMBC

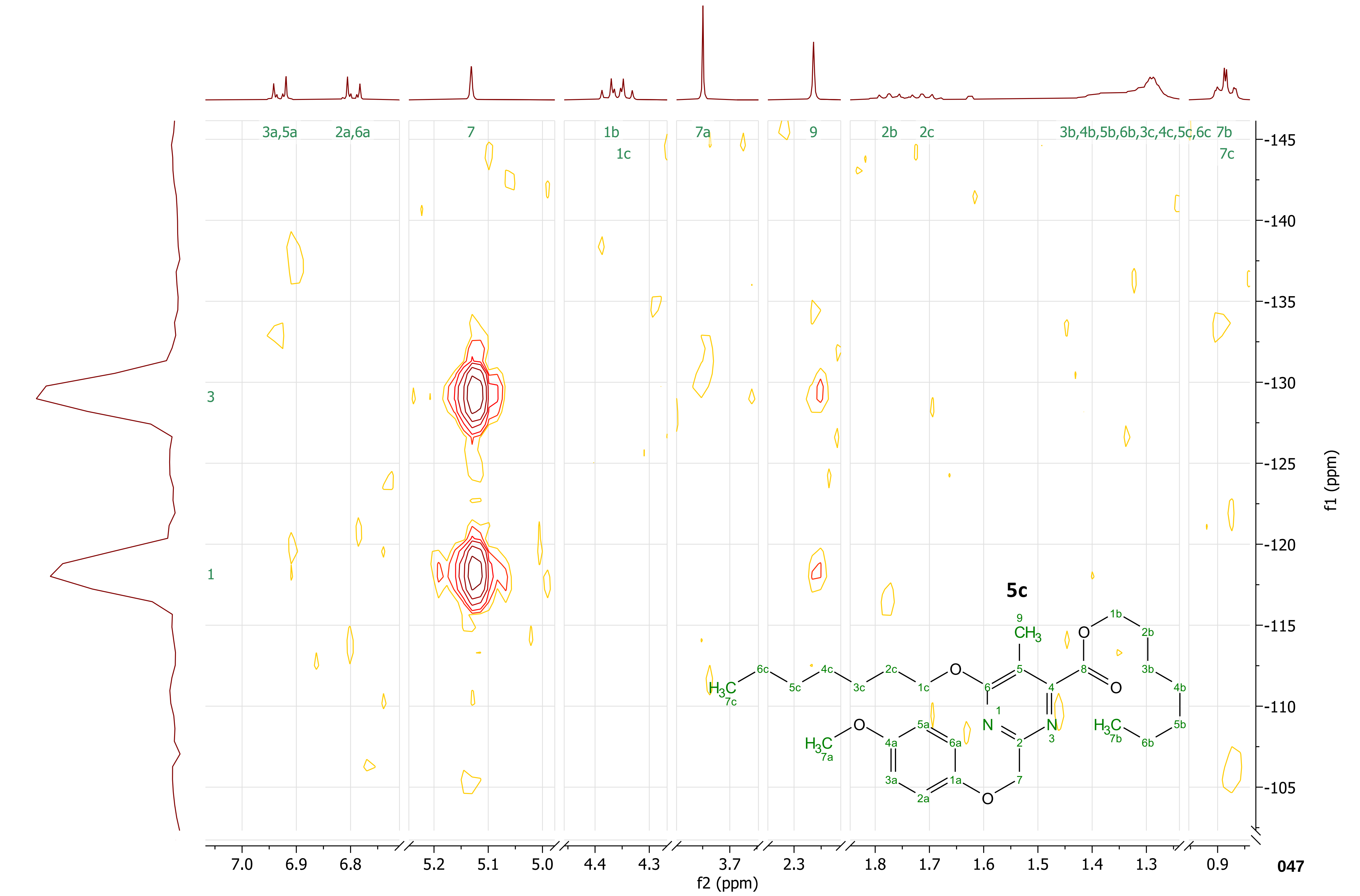
3a,5a
2a,6a
7
1b
1c
7a
9
2b
2c
3b,4b,5b,6b,3c,4c,5c,6c
7b
7c
3
1
5c
f1 (ppm)
f2 (ppm)

15N HMBC - f1 internal projection ^{15}N NMR (41 MHz, $CDCl_3$) δ -118.18, -129.13.

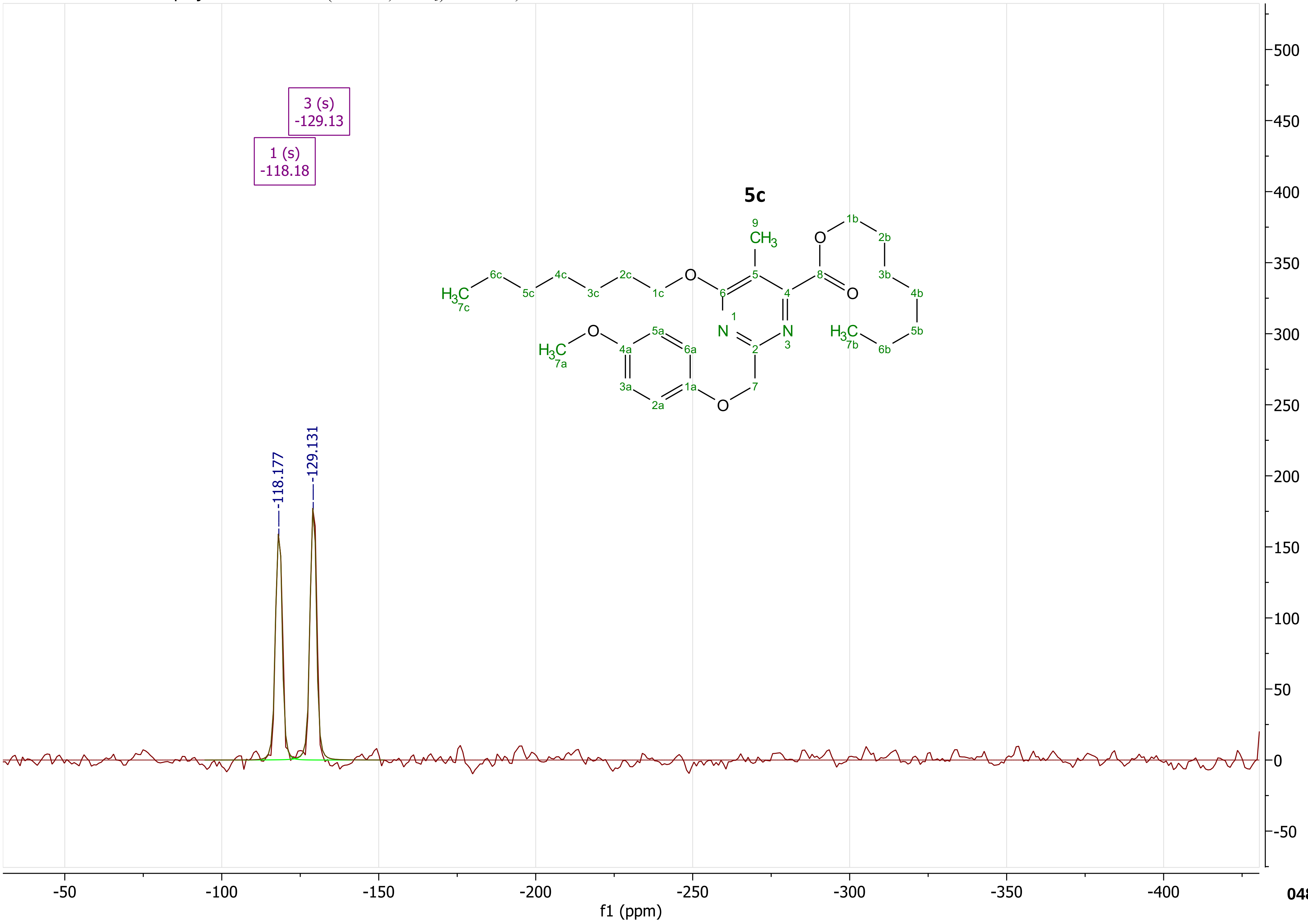

1H ^{1}H NMR (400 MHz, $CDCl_3$) δ 7.03 – 6.87 (m, 2H), 6.87 – 6.70 (m, 2H), 5.13 (s, 2H), 4.37 (t, *J* = 6.9 Hz, 2H), 4.35 (t, *J* = 6.7 Hz, 2H), 3.75 (s, 3H), 2.26 (s, 3H), 1.83 – 1.73 (m, 2H), 1.75 – 1.64 (m, 2H), 1.48 – 1.35 (m, 4H), 1.35 – 1.16 (m, 16H), 0.883 (app t, *J* = 6.9 Hz, 3H), 0.877 (t, *J* = 6.9 Hz, 3H).

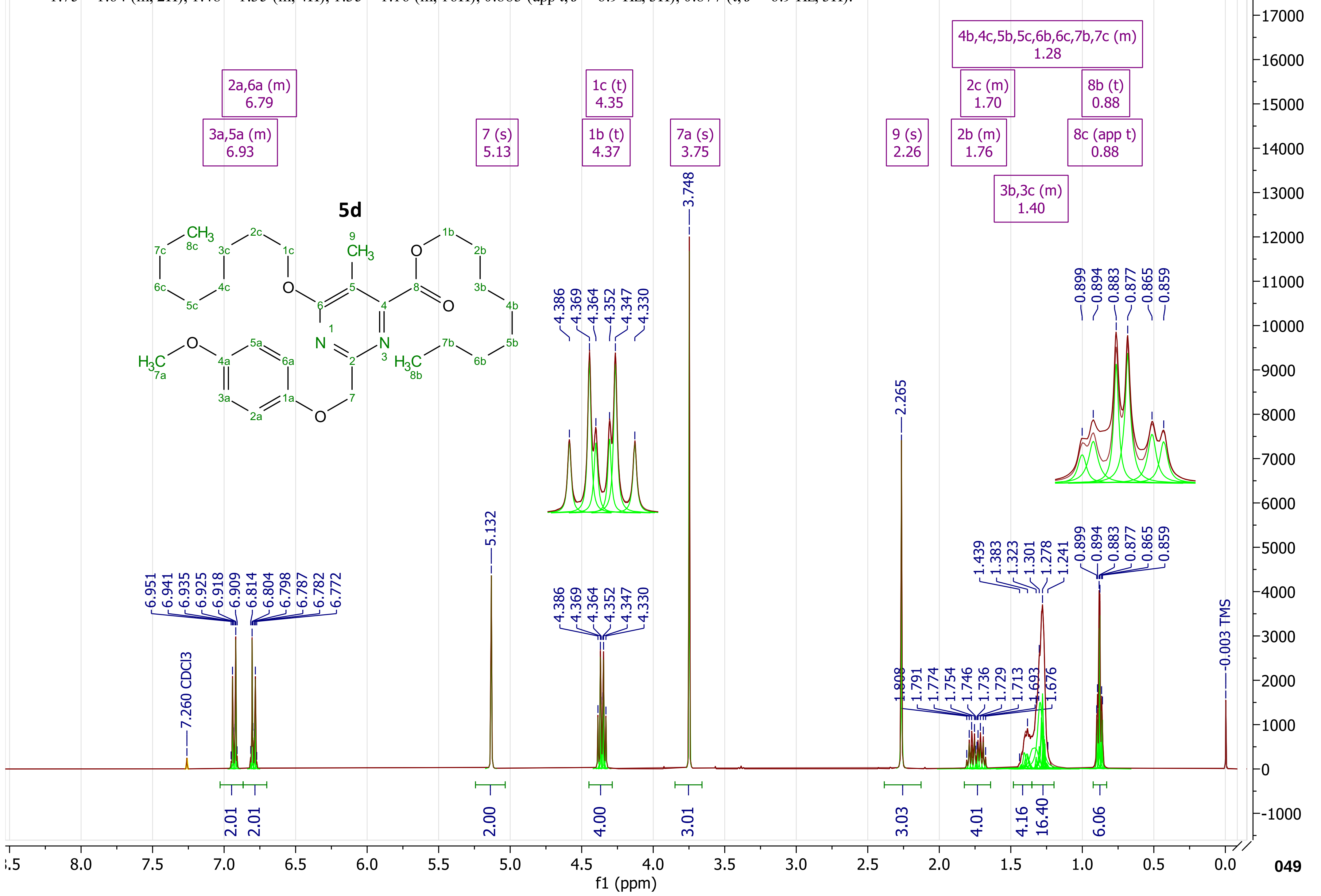

13C ^{13}C NMR (101 MHz, $CDCl_3$) δ 169.3, 165.9, 163.1, 155.1, 154.1, 153.0, 116.6, 116.1 (sym, 2C), 114.6 (sym, 2C), 71.2, 67.7, 66.4, 55.8, 31.93, 31.89, 29.4, 29.34, 29.31, 29.28, 28.72, 28.66, 26.1, 26.0, 22.78, 22.76, 14.23, 14.21, 11.1.

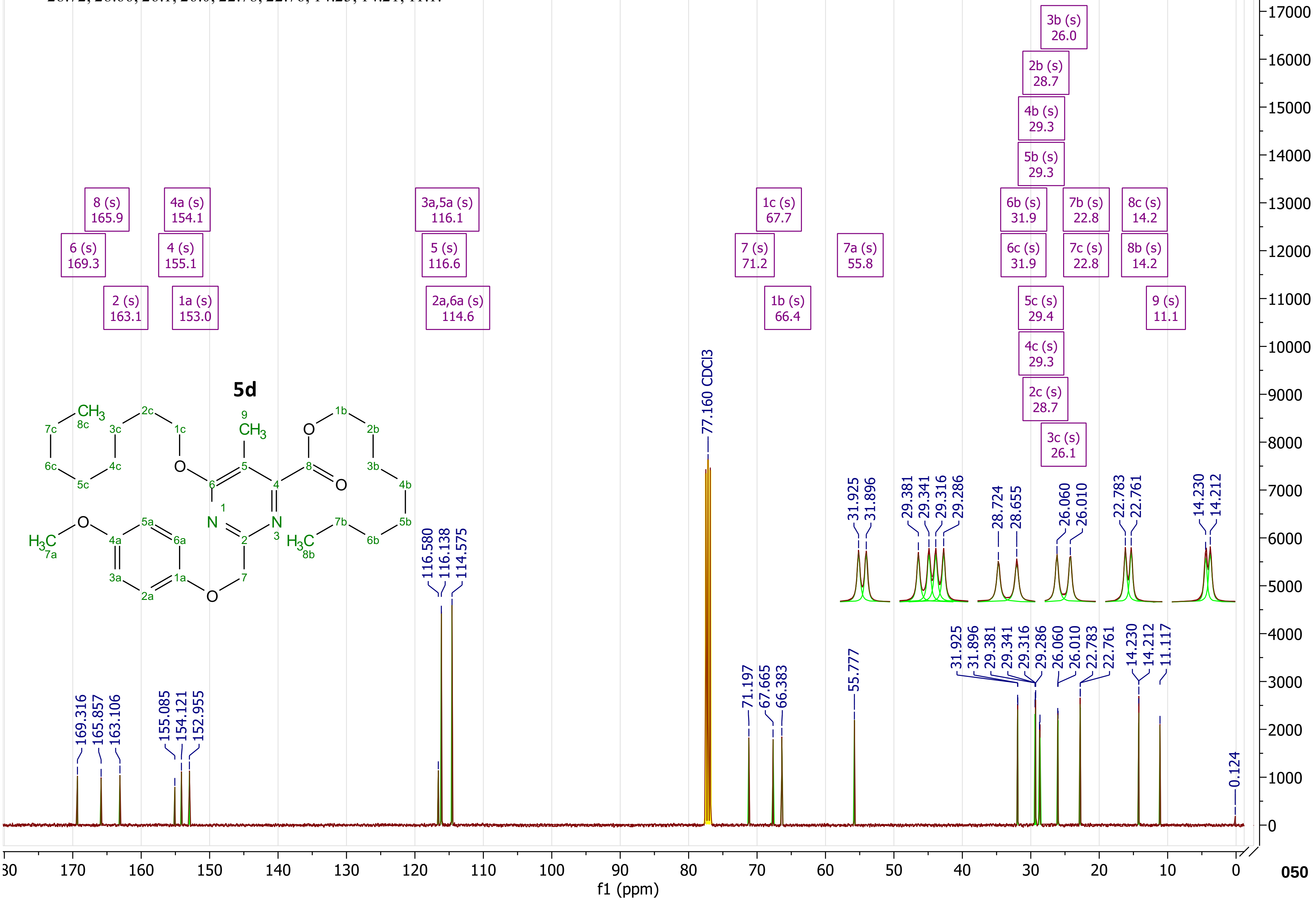

13C HSQC

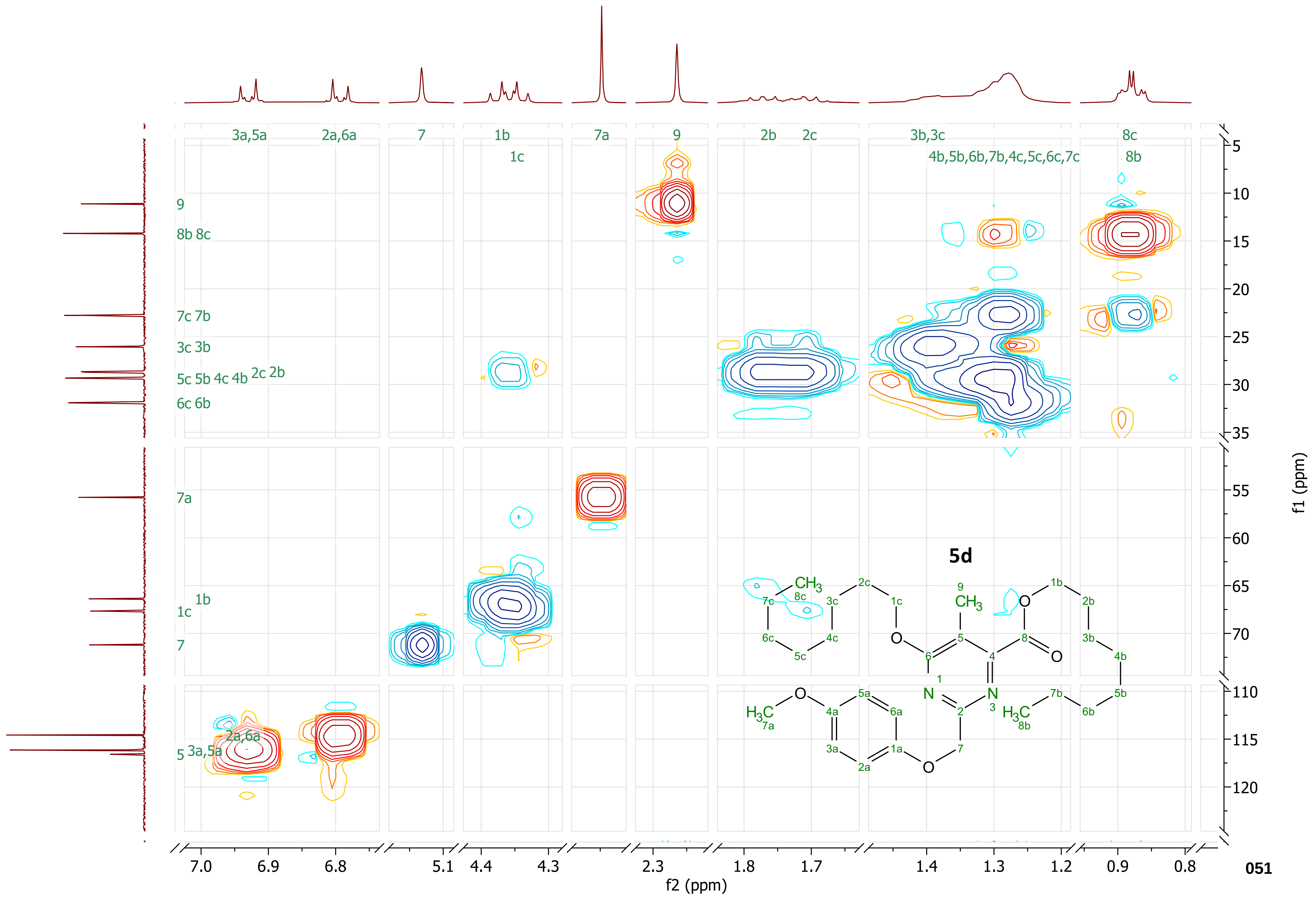

3a,5a
2a,6a
7
1b
1c
7a
9
2b
2c
3b,3c
4b,5b,6b,7b,4c,5c,6c,7c
8c
8b
9
8b 8c
7c 7b
3c 3b
5c 5b 4c 4b 2c 2b
6c 6b
7a
1b
1c
7
5 3a,5a
2a,6a
5d
f1 (ppm)
f2 (ppm)

13C HMBC

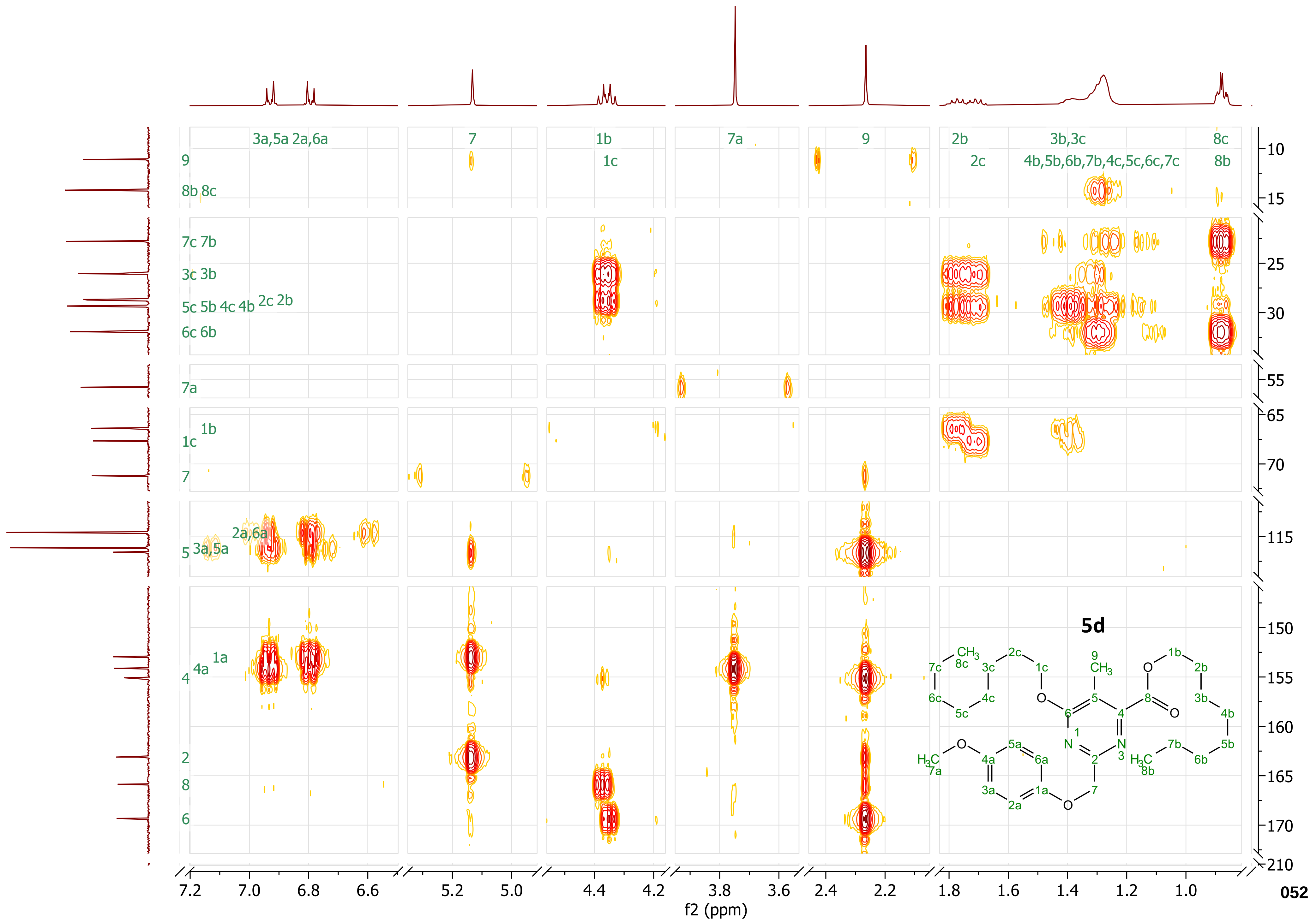
3a,5a 2a,6a
7
1b
1c
7a
9
2b
2c
3b,3c
4b,5b,6b,7b,4c,5c,6c,7c
8c
8b
9
8b 8c
7c 7b
3c 3b
5c 5b 4c 4b 2c 2b
6c 6b
7a
1c 1b
7
5 3a,5a 2a,6a
4 4a 1a
2
8
6
5d
f1 (ppm)
f2 (ppm)

15N HMBC

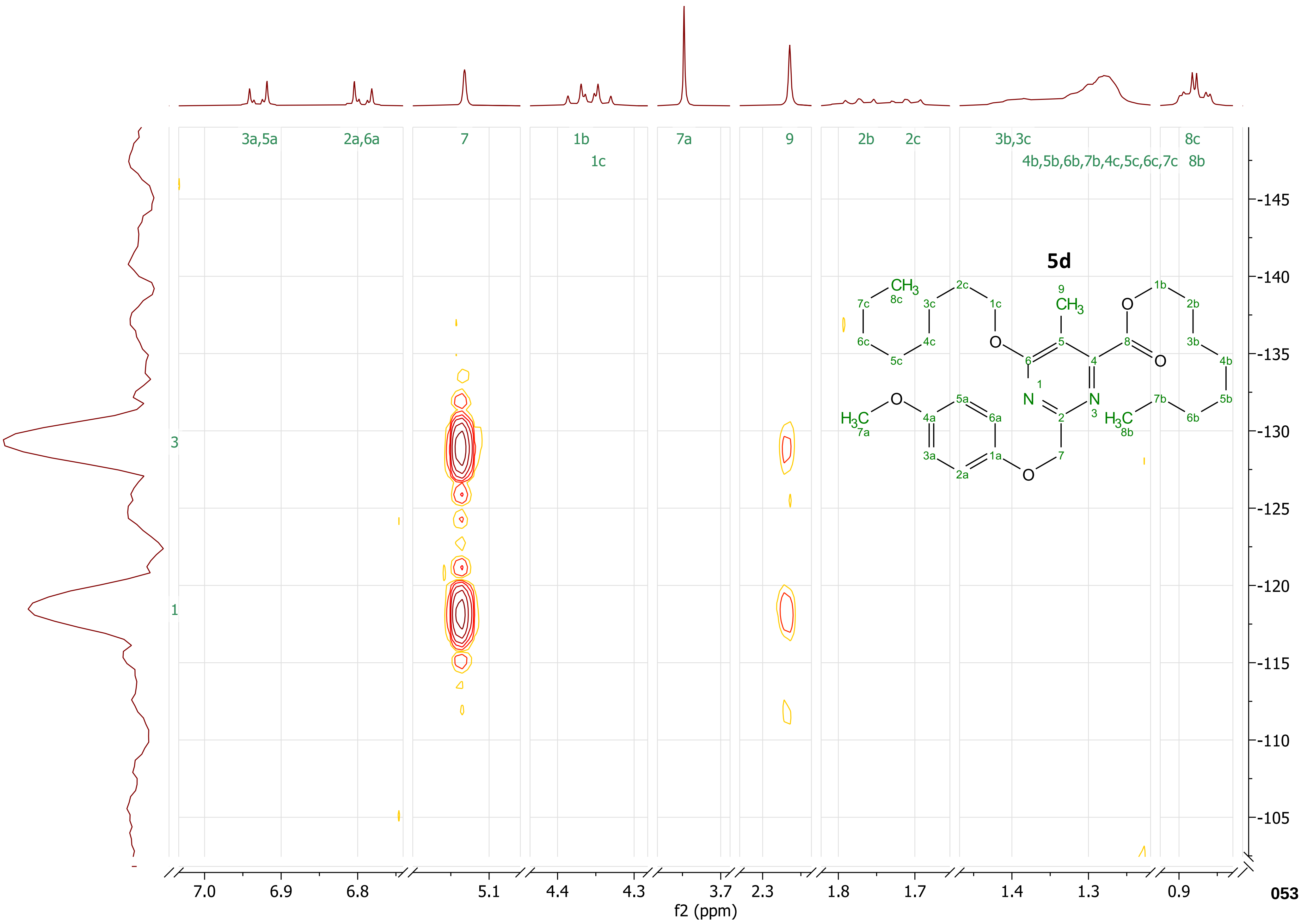

3a,5a
2a,6a
7
1b
1c
7a
9
2b
2c
3b,3c
4b,5b,6b,7b,4c,5c,6c,7c
8c
8b
5d
3
1
f1 (ppm)
f2 (ppm)

15N HMBC - f1 internal projection ^{15}N NMR (41 MHz, $CDCl_3$) δ -118.47, -129.30.

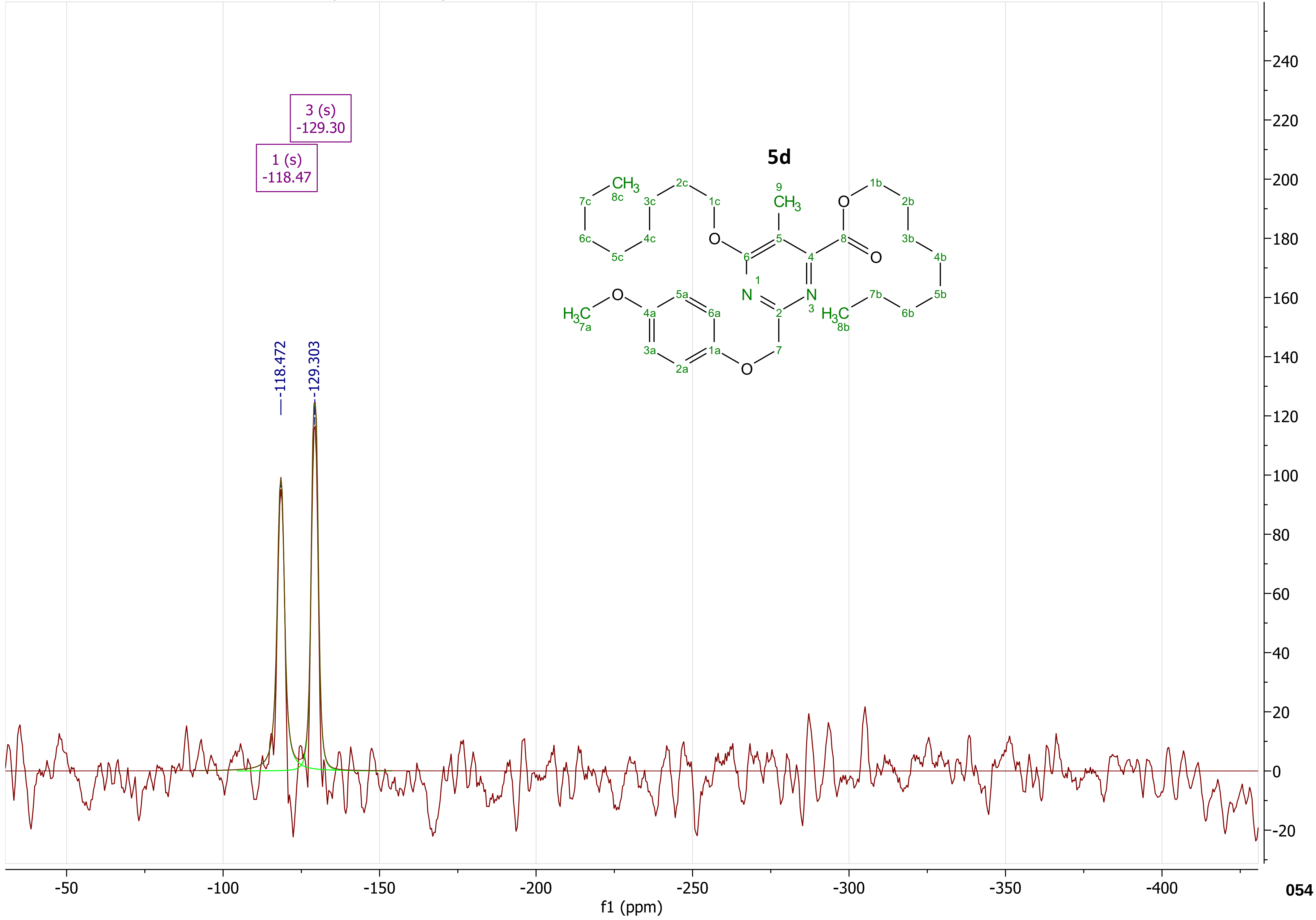

1H ^{1}H NMR (400 MHz, $CDCl_3$) δ 6.96 – 6.90 (m, 2H), 6.84 – 6.76 (m, 2H), 5.13 (s, 2H), 4.37 (t, J = 6.9 Hz, 2H), 4.35 (t, J = 6.9 Hz, 2H), 3.75 (s, 3H), 2.26 (s, 3H), 1.83 – 1.71 (m, 2H), 1.75 – 1.65 (m, 2H), 1.45 – 1.35 (m, 4H), 1.34 – 1.21 (m, 20H), 0.880 (app t, J = 6.9 Hz, 3H), 0.875 (app t, J = 6.9 Hz, 3H).

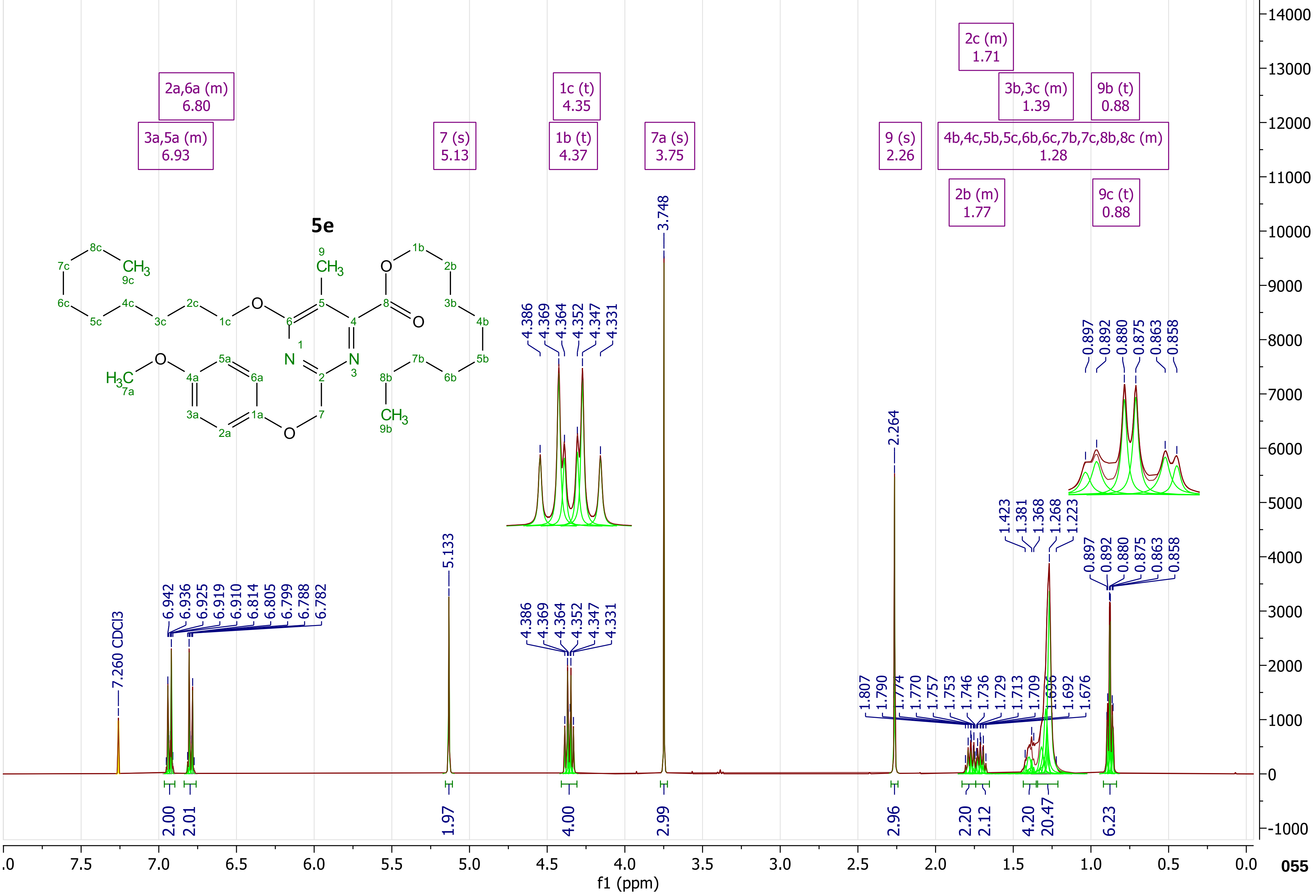

13C ^{13}C NMR (101 MHz, $CDCl_3$) δ 169.3, 165.9, 163.1, 155.1, 154.1, 153.0, 116.6, 116.1 (sym, 2C), 114.6 (sym, 2C), 71.2, 67.7, 66.4, 55.8, 32.00, 31.98, 29.64, 29.59, 29.43, 29.37, 29.36 (2C), 28.73, 28.66, 26.1, 26.0, 22.80, 22.79, 14.2 (2C), 11.1.

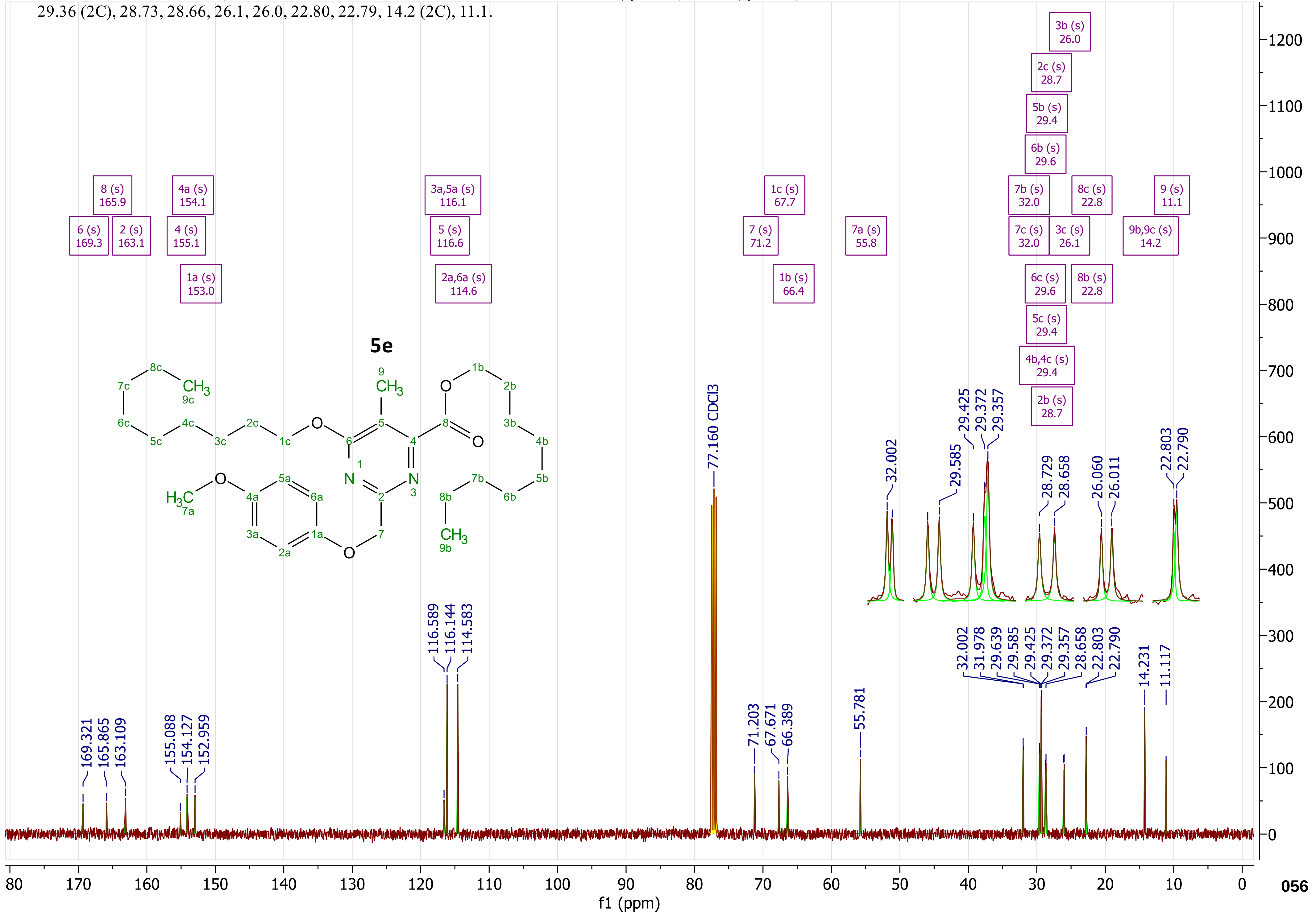

13C HSQC

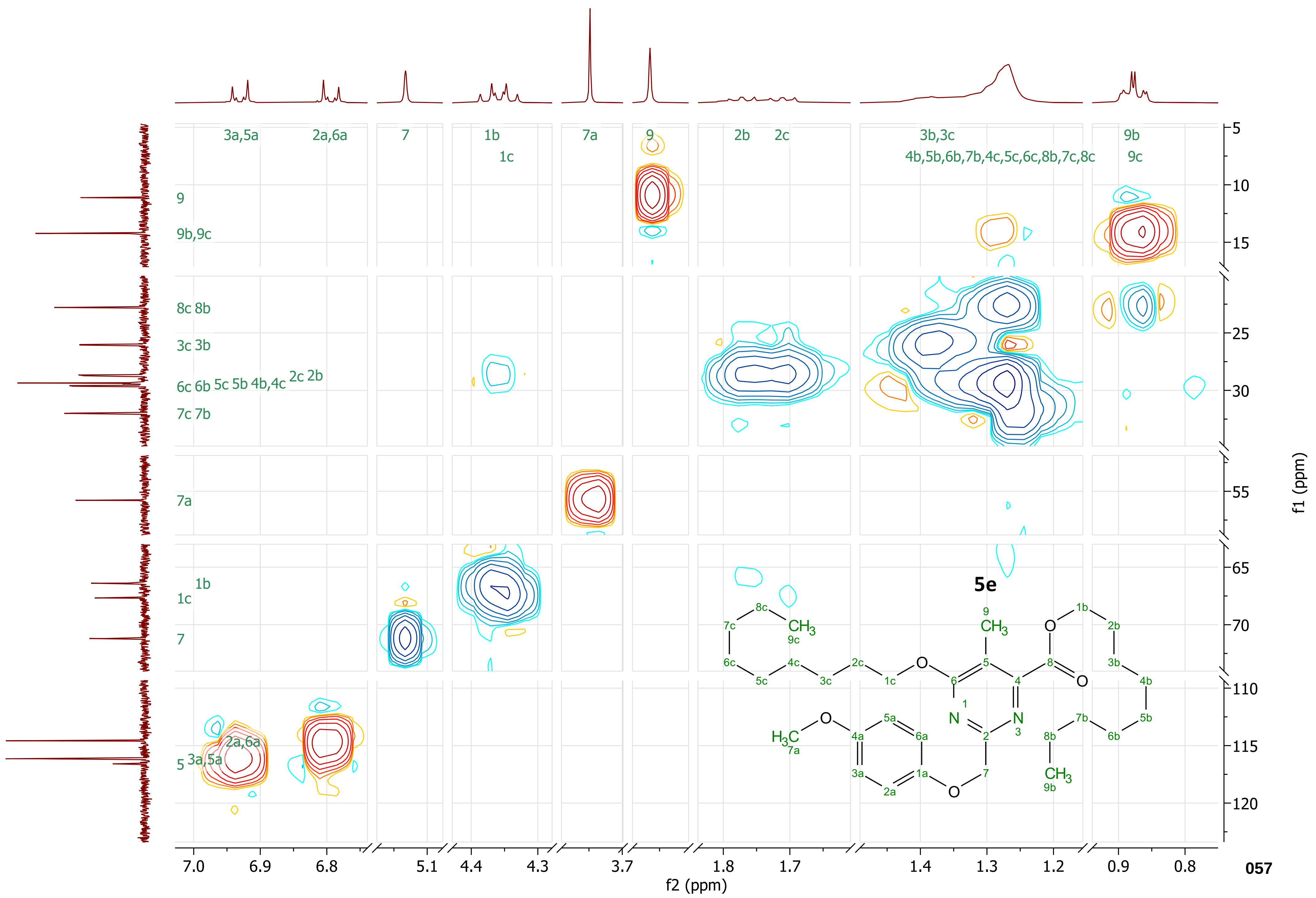
3a,5a
2a,6a
7
1b
1c
7a
9
2b
2c
3b,3c
4b,5b,6b,7b,4c,5c,6c,8b,7c,8c
9b
9c
9
9b,9c
8c 8b
3c 3b
6c 6b 5c 5b 4b,4c
2c 2b
7c 7b
7a
1b
1c
7
2a,6a
5 3a,5a
5e
f1 (ppm)
f2 (ppm)

13C HMBC

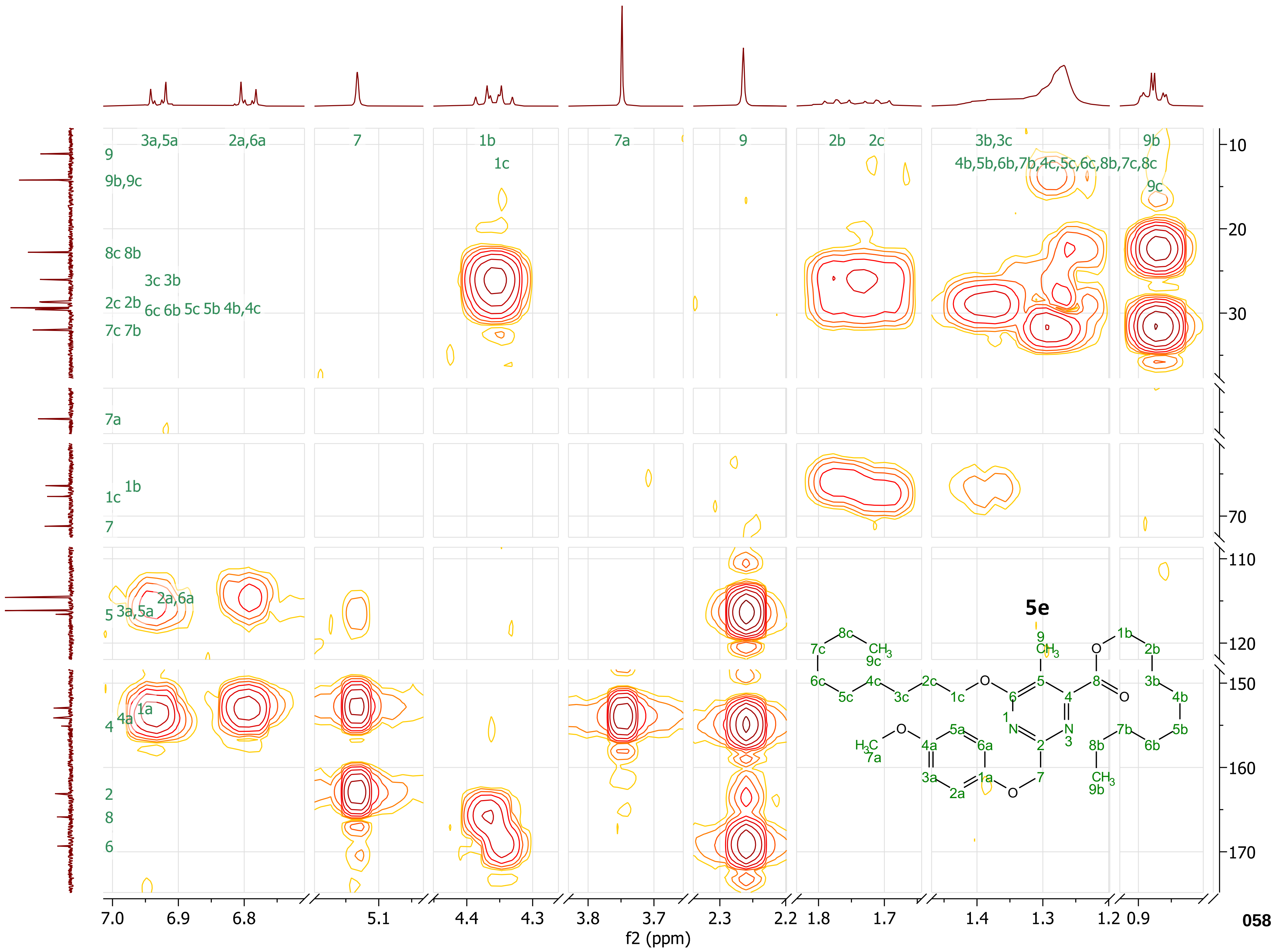

15N HMBC

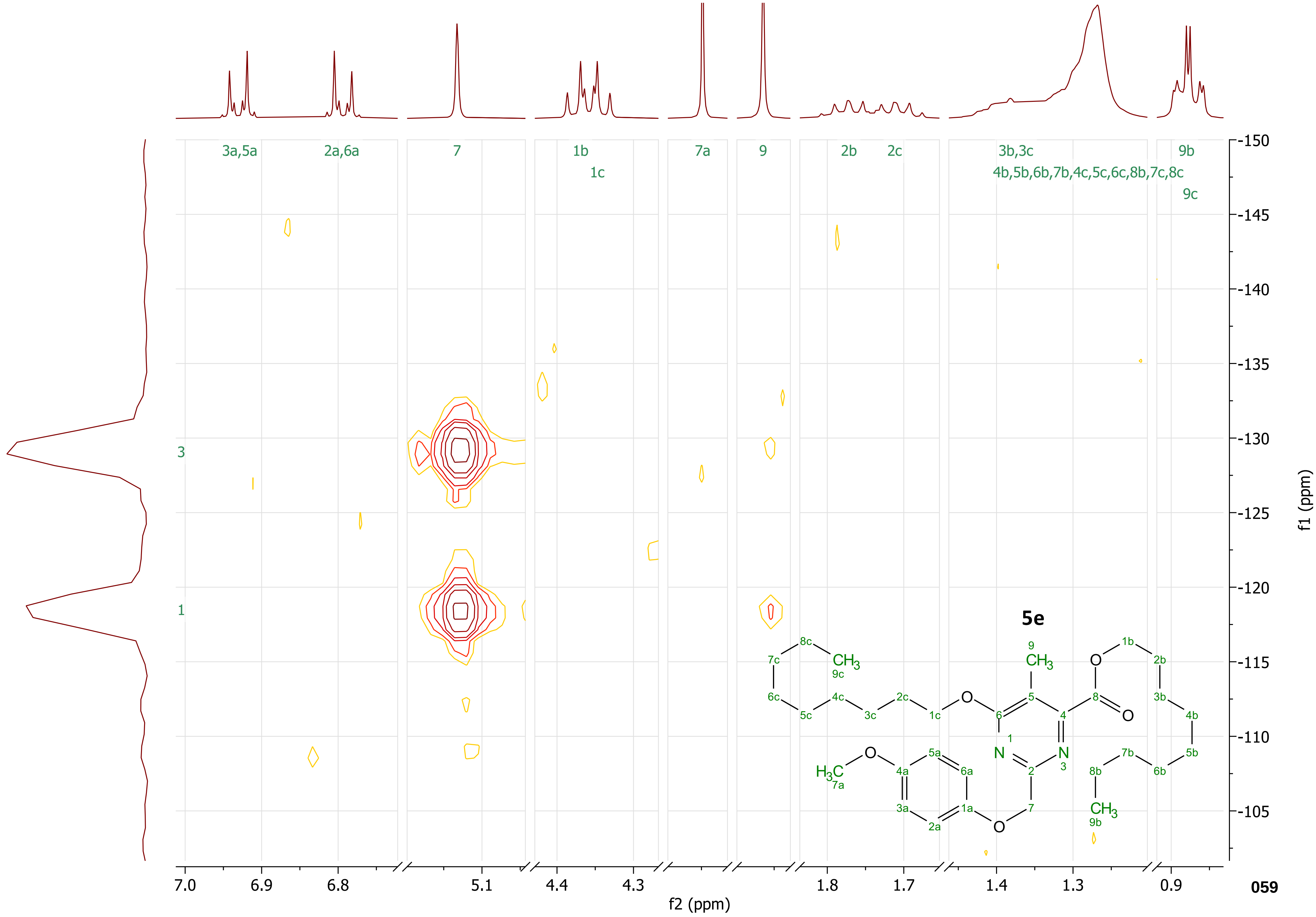
3a,5a
2a,6a
7
1b
1c
7a
9
2b
2c
3b,3c
4b,5b,6b,7b,4c,5c,6c,8b,7c,8c
9c
9b
3
1
5e
f2 (ppm)
f1 (ppm)

15N HMBC - f1 internal projection ^{15}N NMR (41 MHz, $CDCl_3$) δ -118.51, -129.10.

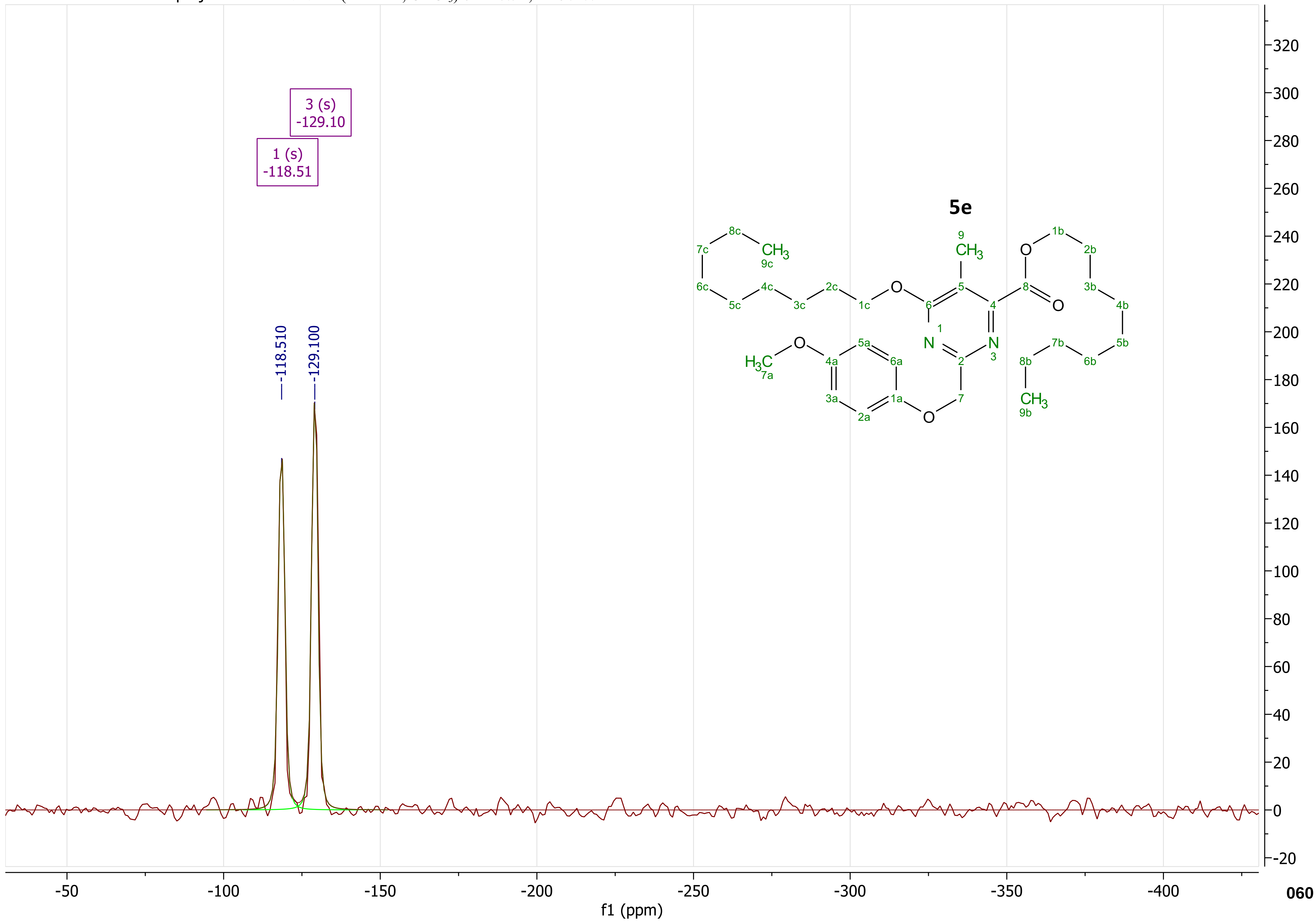

1H ^{1}H NMR (400 MHz, $CDCl_3$) δ 6.98 – 6.90 (m, 2H), 6.85 – 6.76 (m, 2H), 5.20 (s, 2H), 4.40 (t, J = 6.7 Hz, 2H), 3.75 (s, 3H), 2.49 (s, 3H), 1.81 – 1.70 (m, 2H), 1.51 – 1.37 (m, 2H), 0.97 (t, J = 7.4 Hz, 3H).

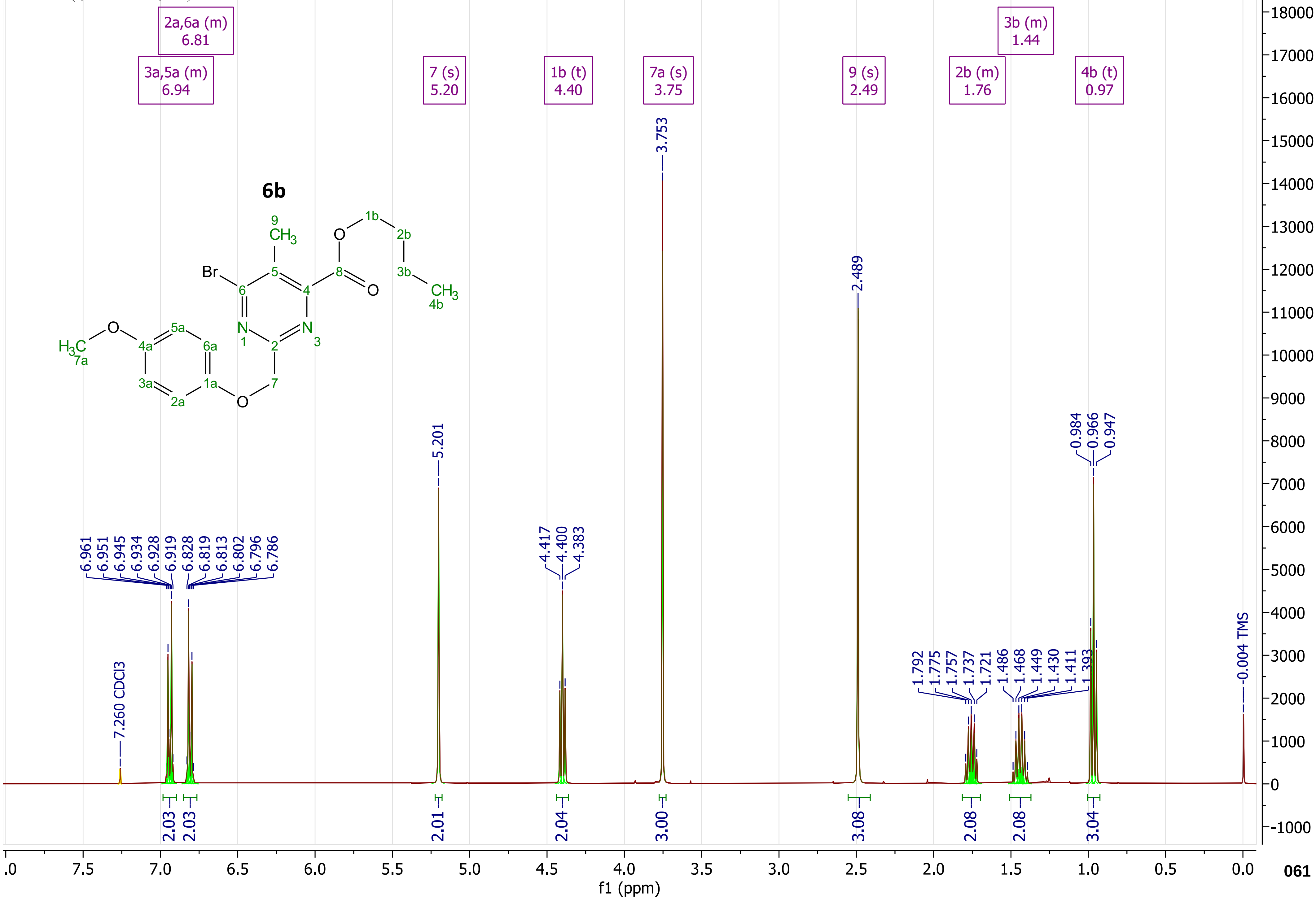

13C ^{13}C NMR (101 MHz, $CDCl_3$) δ 164.7, 164.0, 158.2, 157.0, 154.4, 152.6, 130.1, 116.4 (sym, 2C), 114.7 (sym, 2C), 70.9, 66.7, 55.8, 30.6, 19.2, 17.8, 13.8.

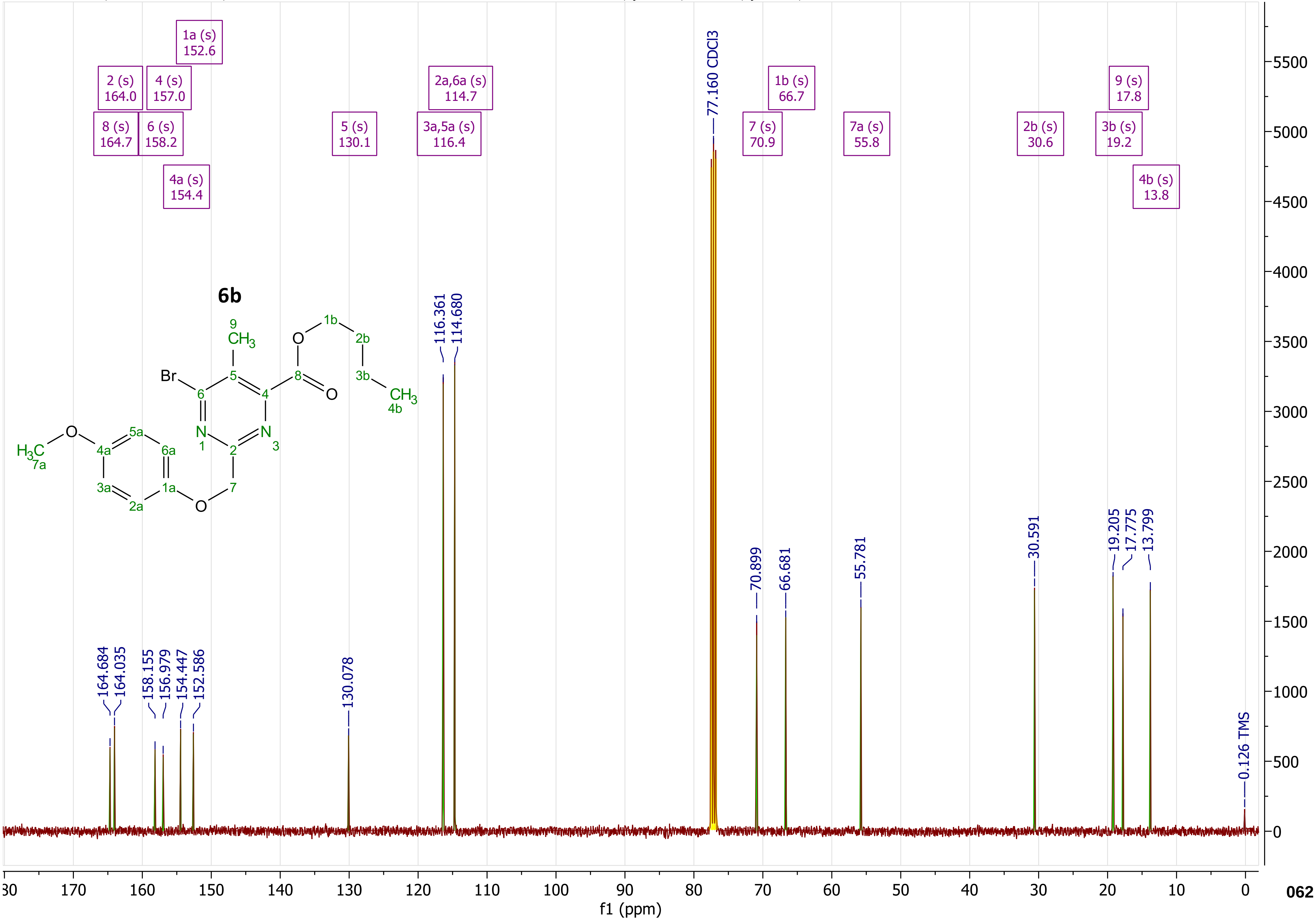

13C HSQC

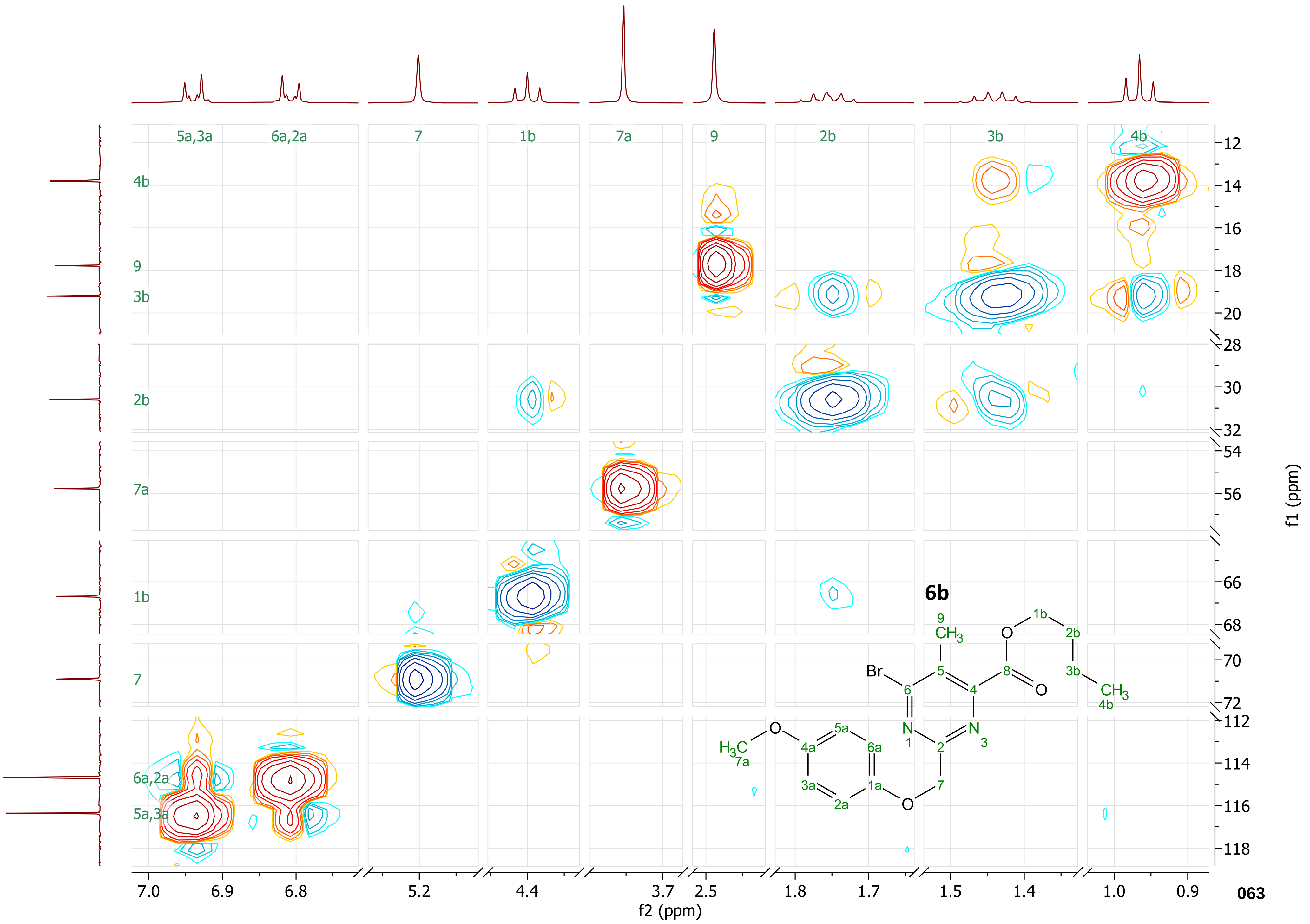

13C HMBC

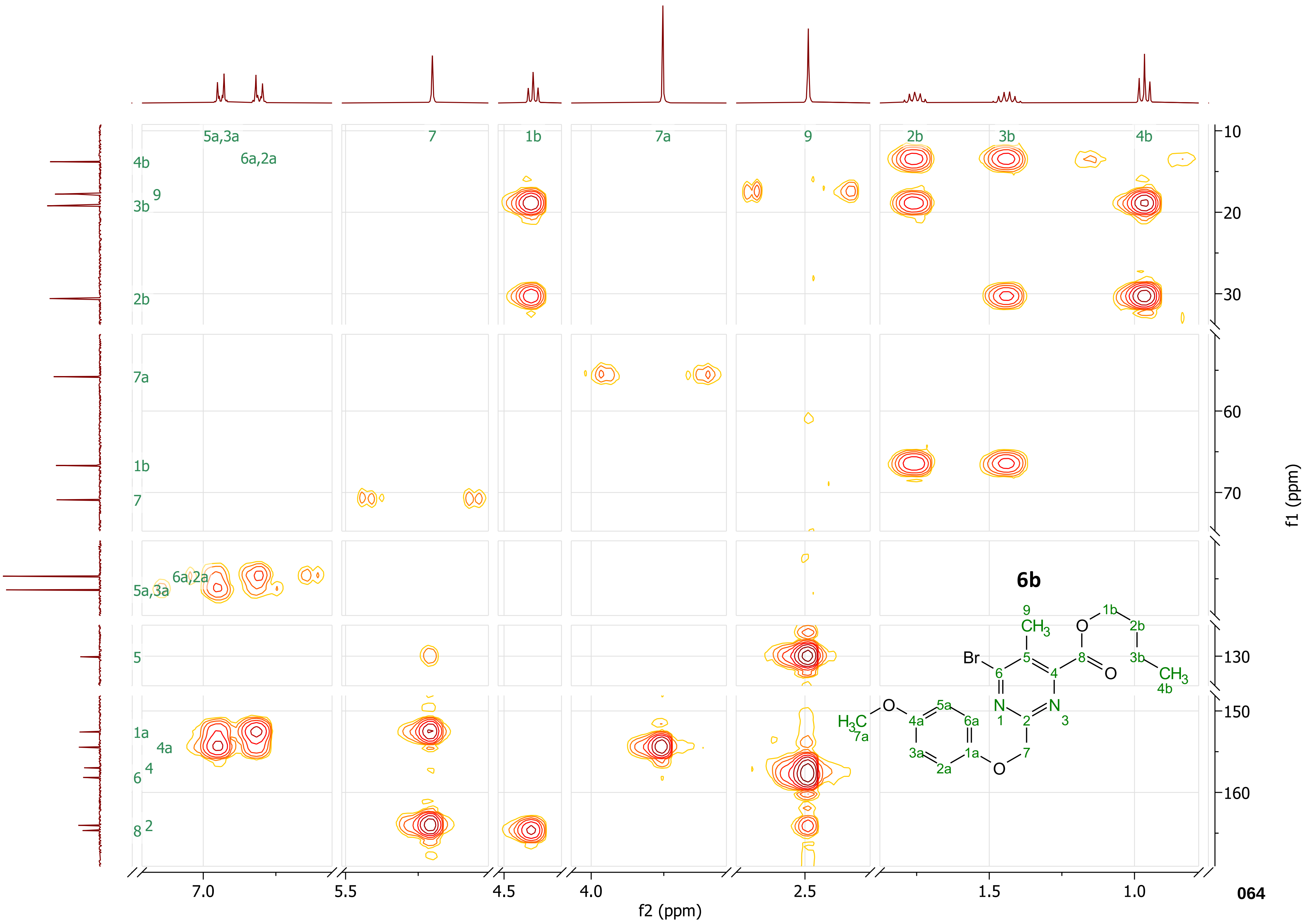

15N HMBC

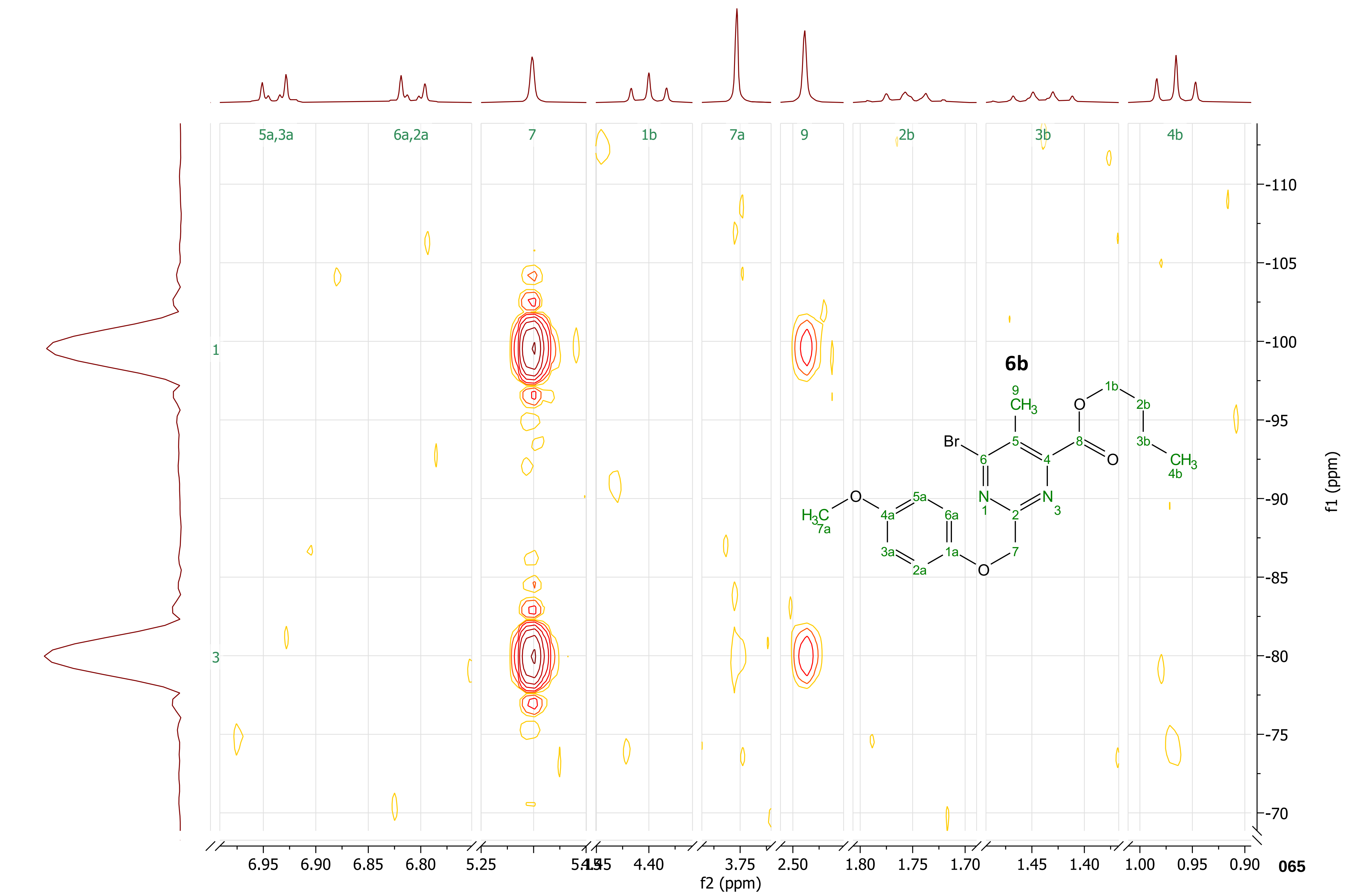

15N HMBC - f1 internal projection ^{15}N NMR (41 MHz, $CDCl_3$) δ -79.93, -99.50.

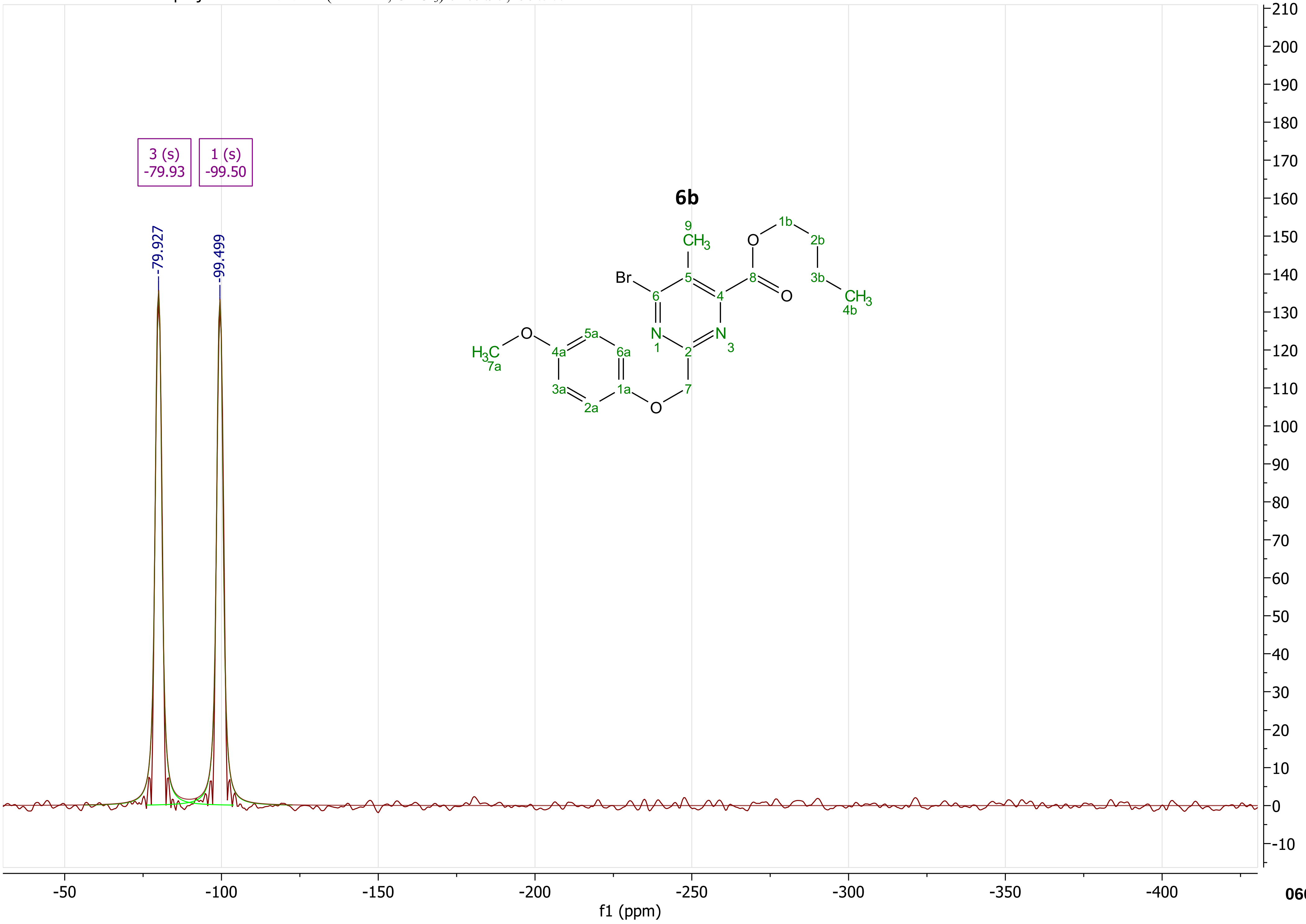

1H ^{1}H NMR (400 MHz, $CDCl_3$) δ 6.99 – 6.88 (m, 2H), 6.88 – 6.73 (m, 2H), 5.20 (s, 2H), 4.39 (t, *J* = 6.8 Hz, 2H), 3.76 (s, 3H), 2.49 (s, 3H), 1.82 – 1.72 (m, 2H), 1.45 – 1.24 (m, 8H), 0.89 (app t, *J* = 7.0 Hz, 3H).

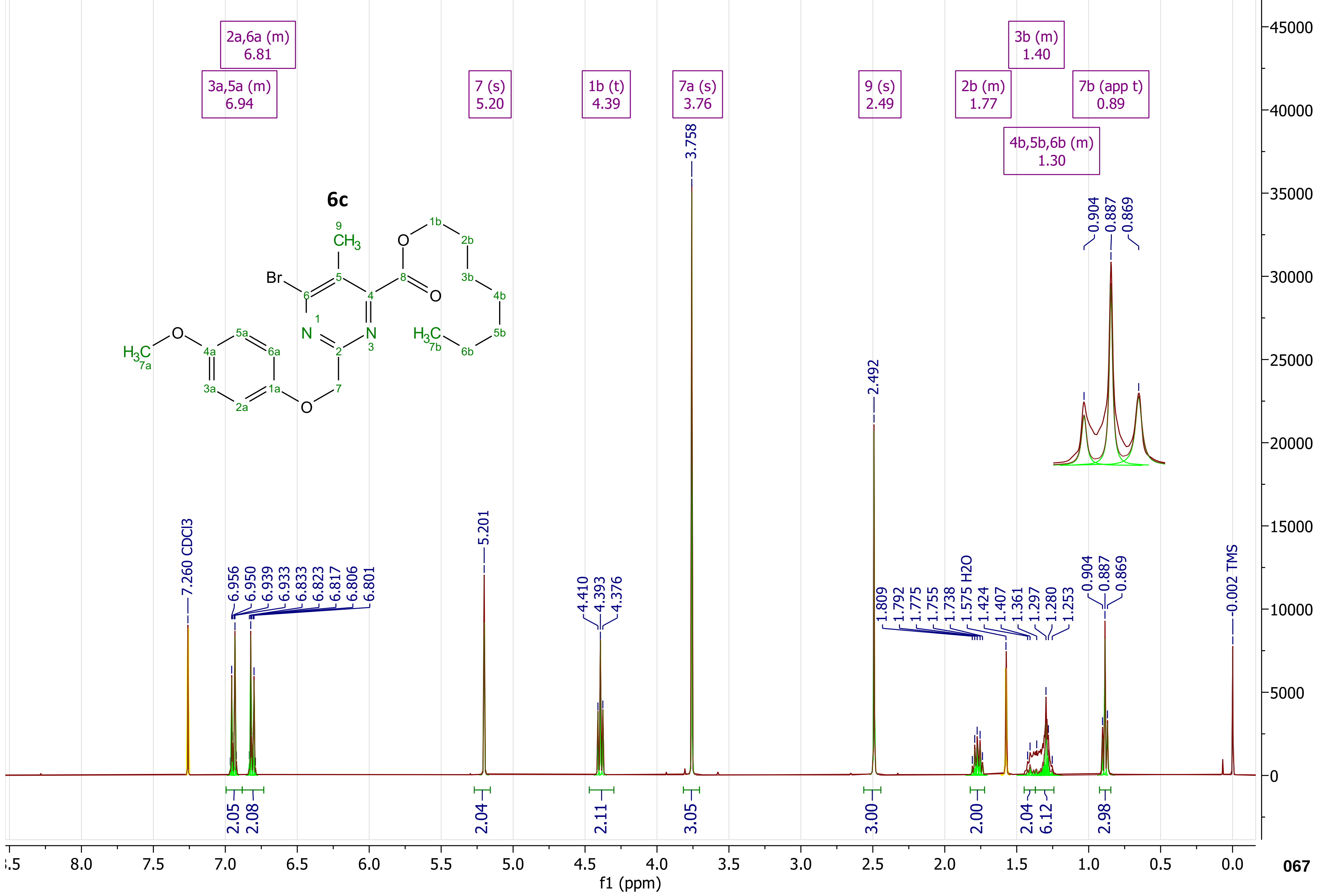

13C ^{13}C NMR (101 MHz, $CDCl_3$) δ 164.7, 164.1, 158.2, 157.0, 154.5, 152.6, 130.1, 116.4 (sym, 2C), 114.7 (sym, 2C), 70.9, 67.0, 55.8, 31.8, 29.0, 28.6, 25.9, 22.7, 17.8, 14.2.

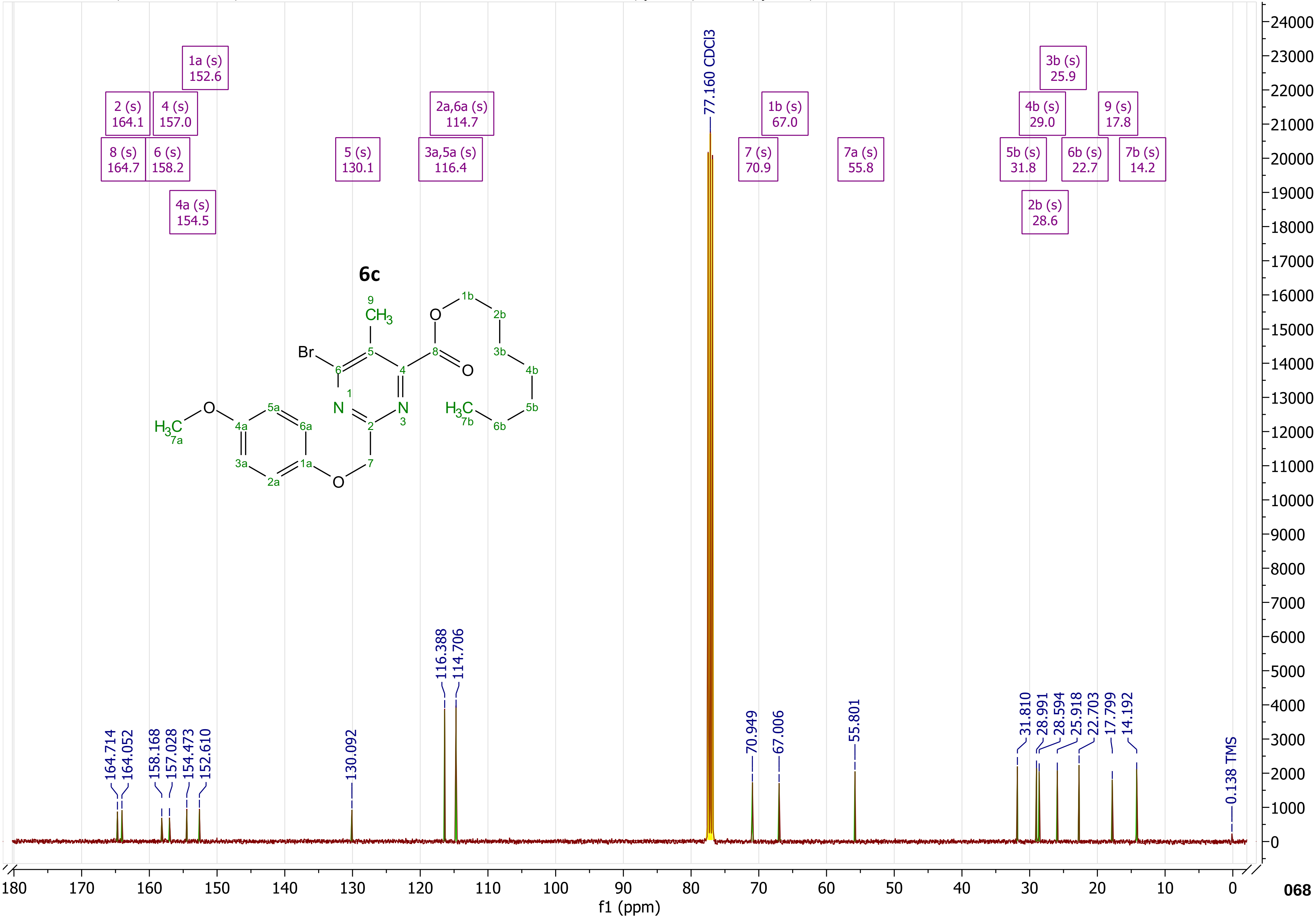

13C HSQC

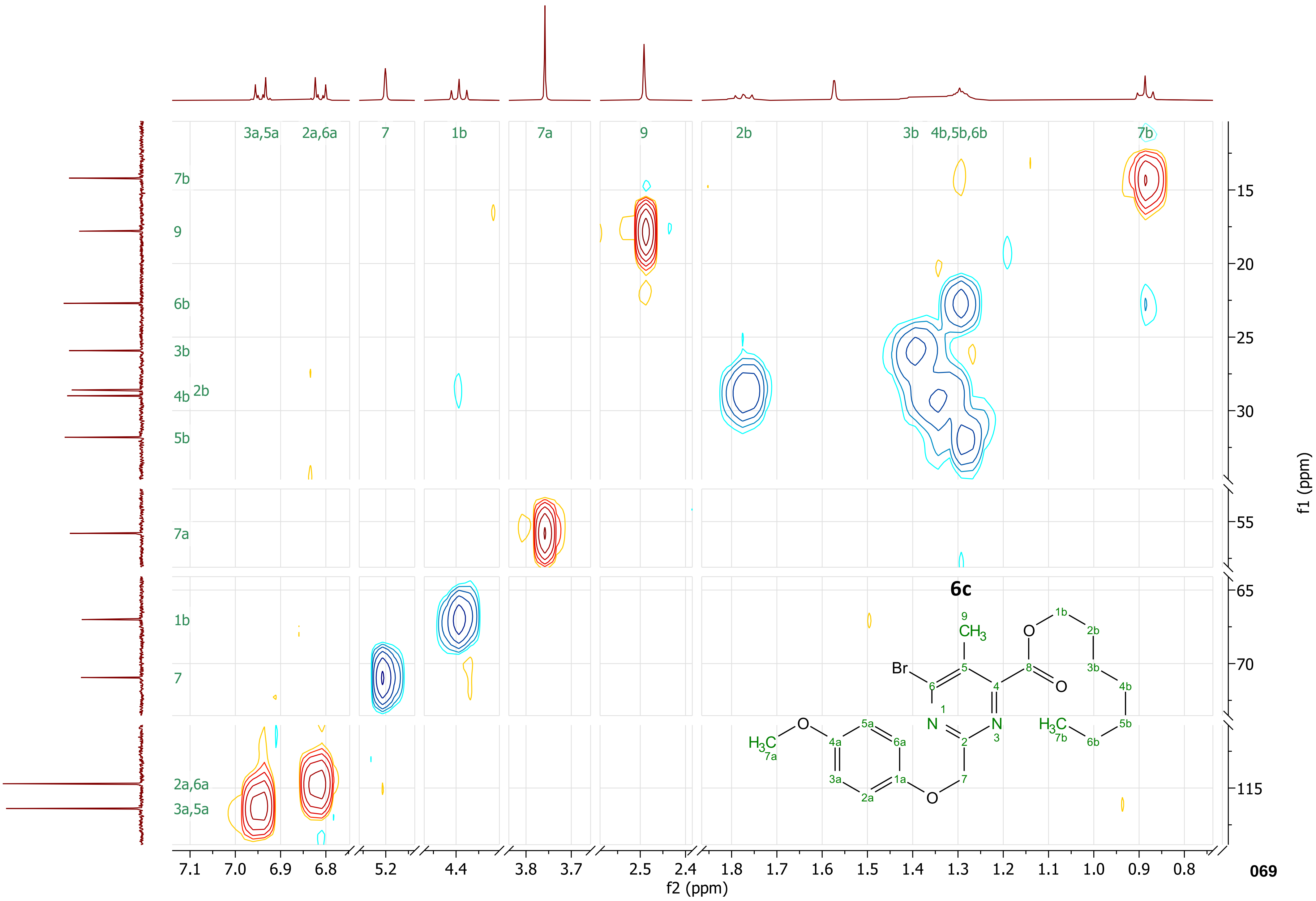

3a,5a
2a,6a
7
1b
7a
9
2b
3b
4b,5b,6b
7b
7b
9
6b
3b
4b
2b
5b
7a
1b
7
2a,6a
3a,5a
15
20
25
30
55
65
70
115
f1 (ppm)
7.1
7.0
6.9
6.8
5.2
4.4
3.8
3.7
2.5
2.4
1.8
1.7
1.6
1.5
1.4
1.3
1.2
1.1
1.0
0.9
0.8
f2 (ppm)
6c
Br
O
O
N
N
O
O
CH_3
H_3C
H_3C

13C HMBC

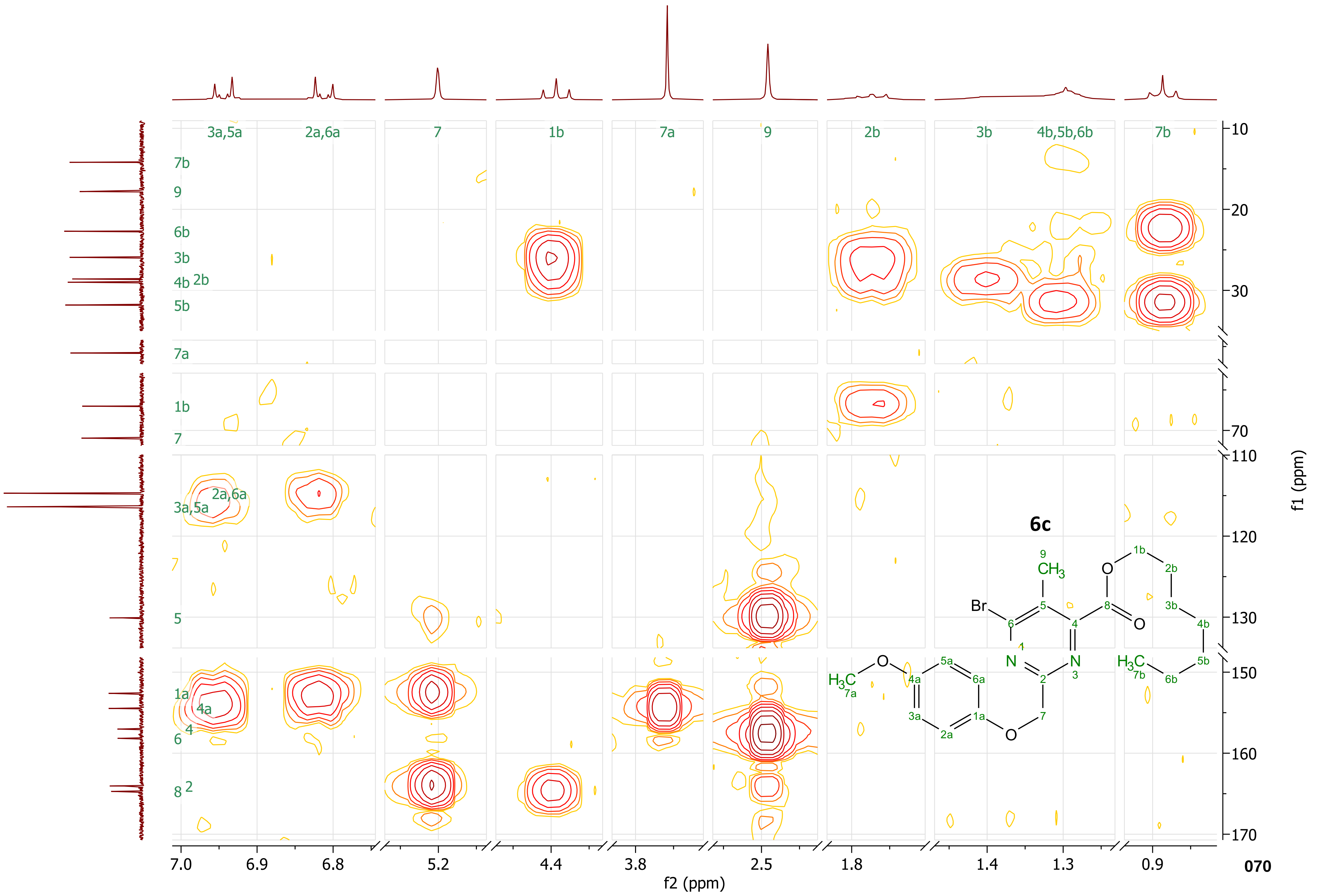

3a,5a
2a,6a
7
1b
7a
9
2b
3b
4b,5b,6b
7b
6b
3b
4b 2b
5b
1b
3a,5a
5
1a
4a
4
6
8 2
6c
Br
CH3
N
O
H3C
f2 (ppm)
f1 (ppm)

15N HMBC

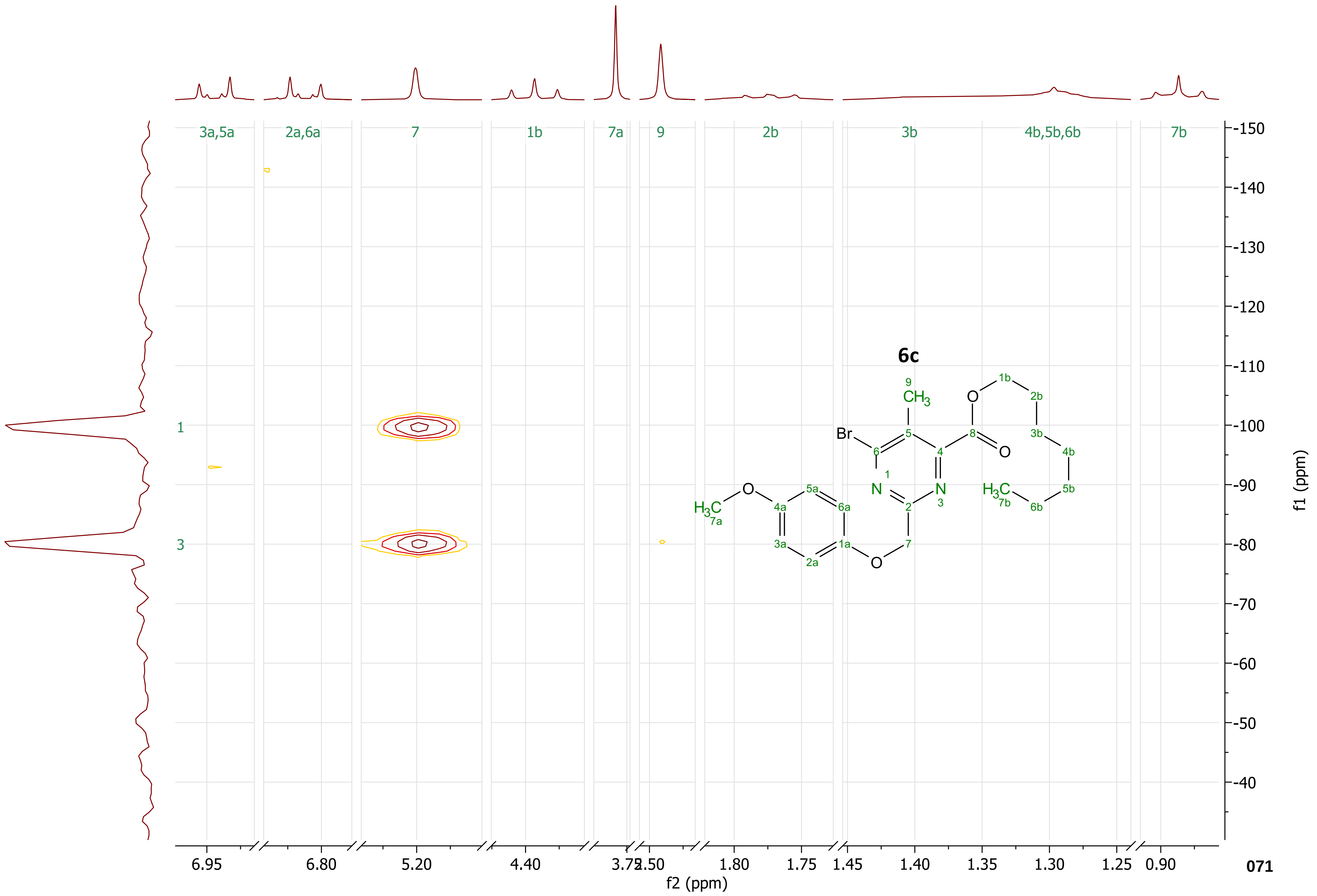
3a,5a
2a,6a
7
1b
7a
9
2b
3b
4b,5b,6b
7b
1
3
6c
f2 (ppm)
f1 (ppm)
6.95
6.80
5.20
4.40
3.75
2.50
1.80
1.75
1.45
1.40
1.35
1.30
1.25
0.90
-150
-140
-130
-120
-110
-100
-90
-80
-70
-60
-50
-40

15N HMBC - f1 internal projection ^{15}N NMR (41 MHz, $CDCl_3$) δ -79.98, -99.75.

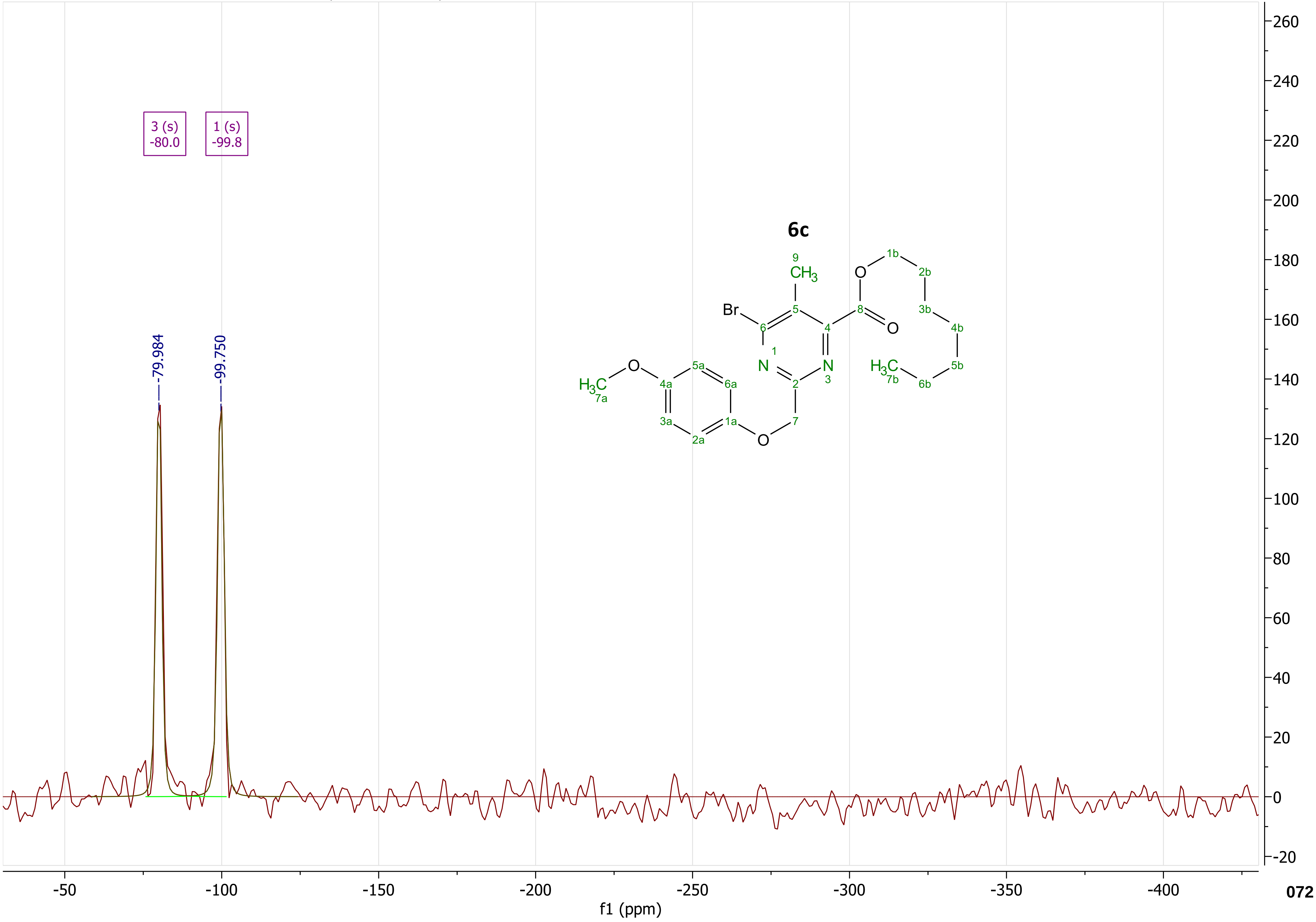

1H ^{1}H NMR (400 MHz, $CDCl_3$) δ 7.04 – 6.85 (m, 2H), 6.85 – 6.68 (m, 2H), 5.20 (s, 2H), 4.39 (t, J = 6.8 Hz, 2H), 3.75 (s, 3H), 2.49 (s, 3H), 1.88 – 1.68 (m, 2H), 1.48 – 1.37 (m, 2H), 1.37 – 1.16 (m, 8H), 0.88 (app t, J = 7.0 Hz, 3H).

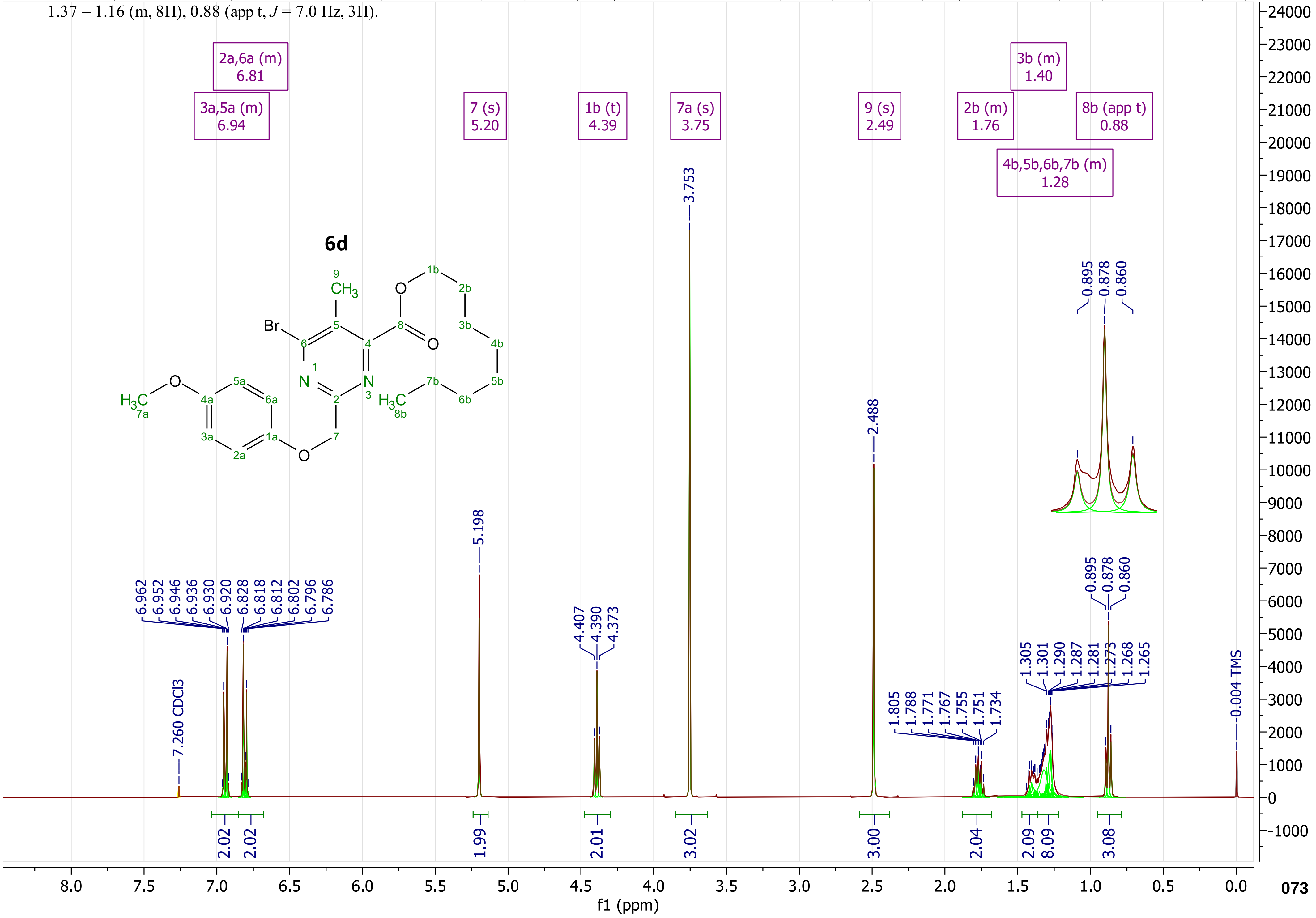

13C ^{13}C NMR (101 MHz, $CDCl_3$) δ 164.7, 164.0, 158.2, 157.0, 154.5, 152.6, 130.1, 116.4 (sym, 2C), 114.7 (sym, 2C), 70.9, 67.0, 55.8, 31.9, 29.3 (2C), 28.6, 26.0, 22.8, 17.8, 14.2.

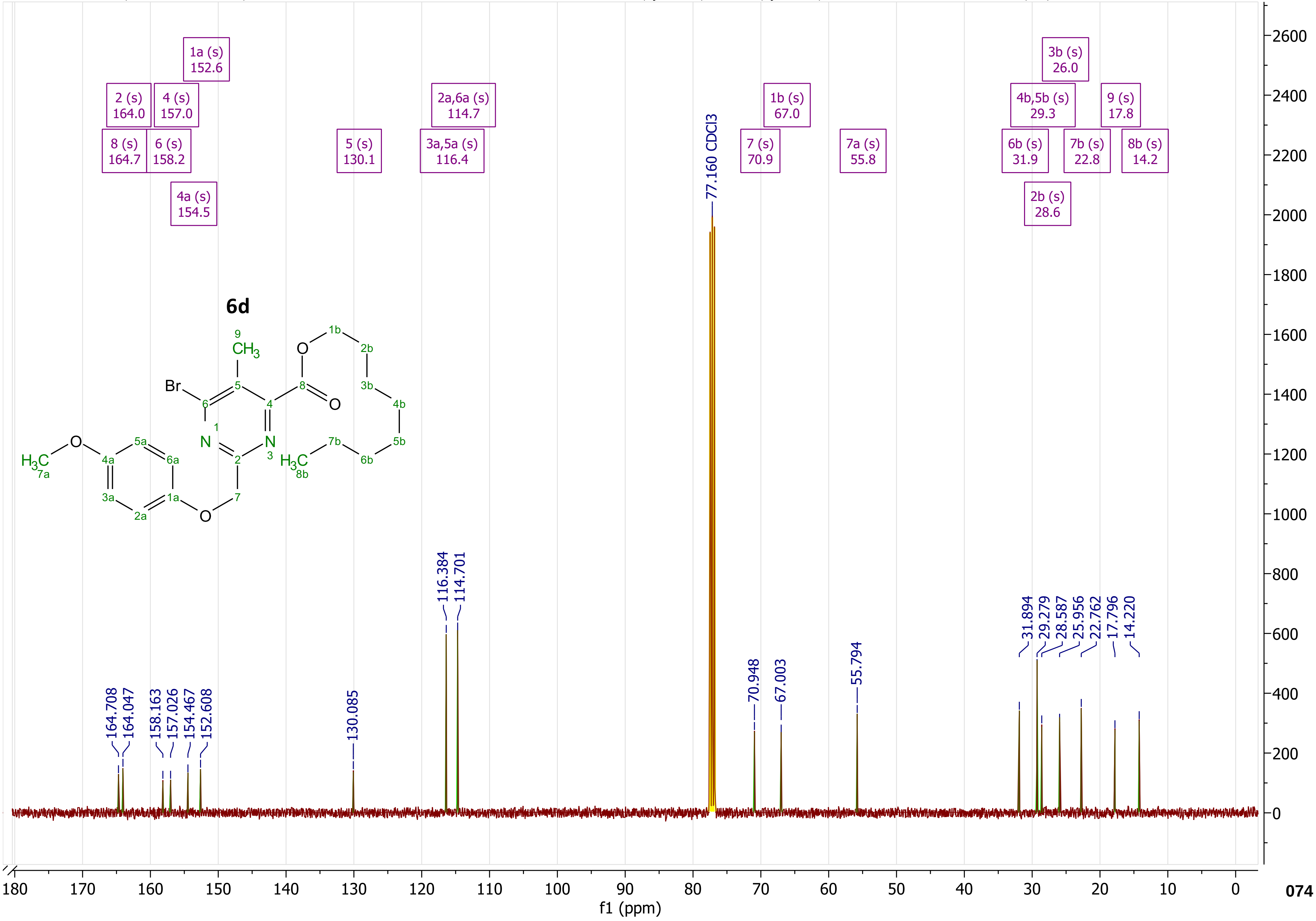

13C HSQC

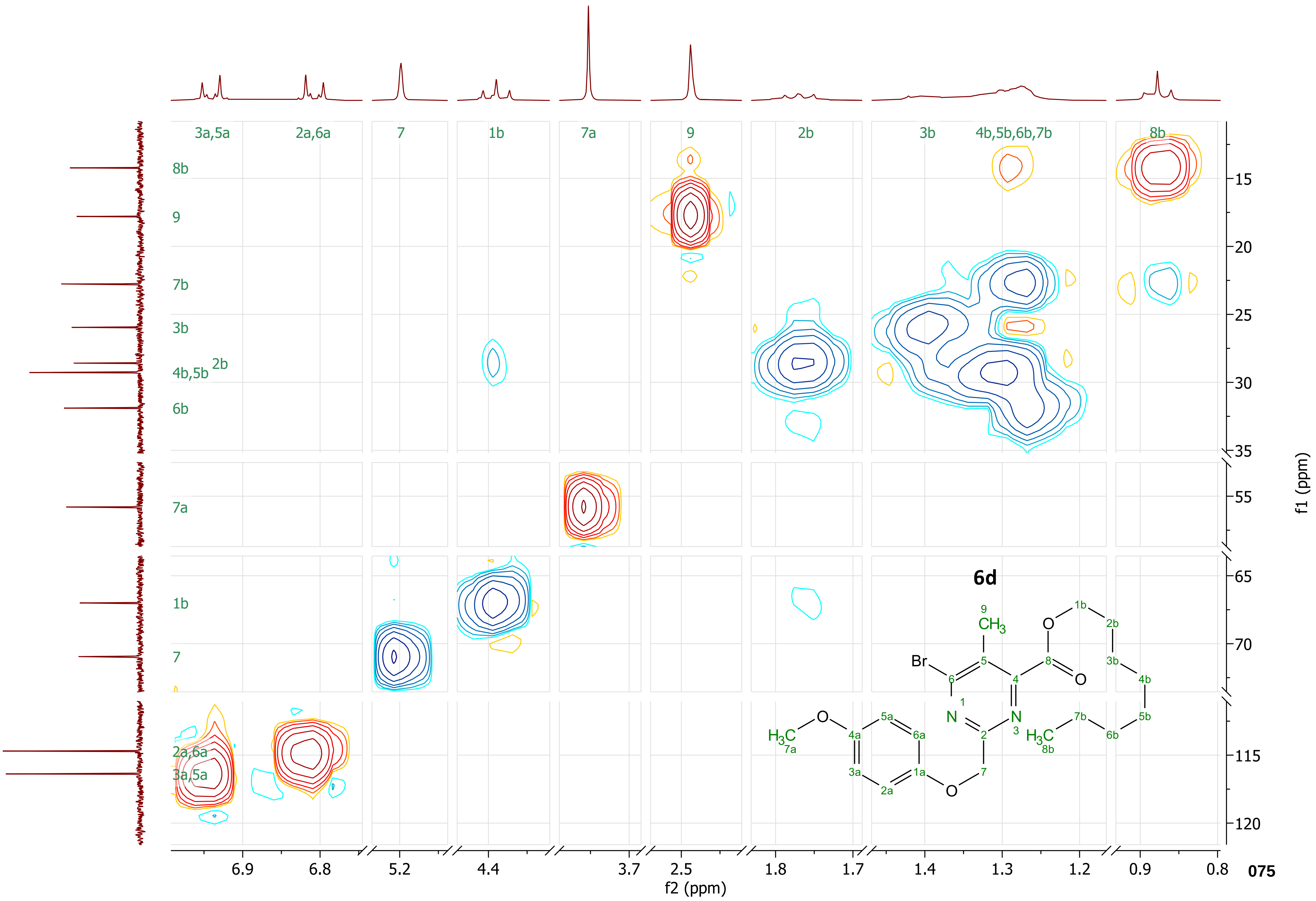

3a,5a
2a,6a
7
1b
7a
9
2b
3b
4b,5b,6b,7b
8b
4b,5b
6b
1b
2a,6a
3a,5a
6d
f2 (ppm)
f1 (ppm)
6.9
6.8
5.2
4.4
3.7
2.5
1.8
1.7
1.4
1.3
1.2
0.9
0.8
15
20
25
30
35
55
65
70
115
120

13C HMBC

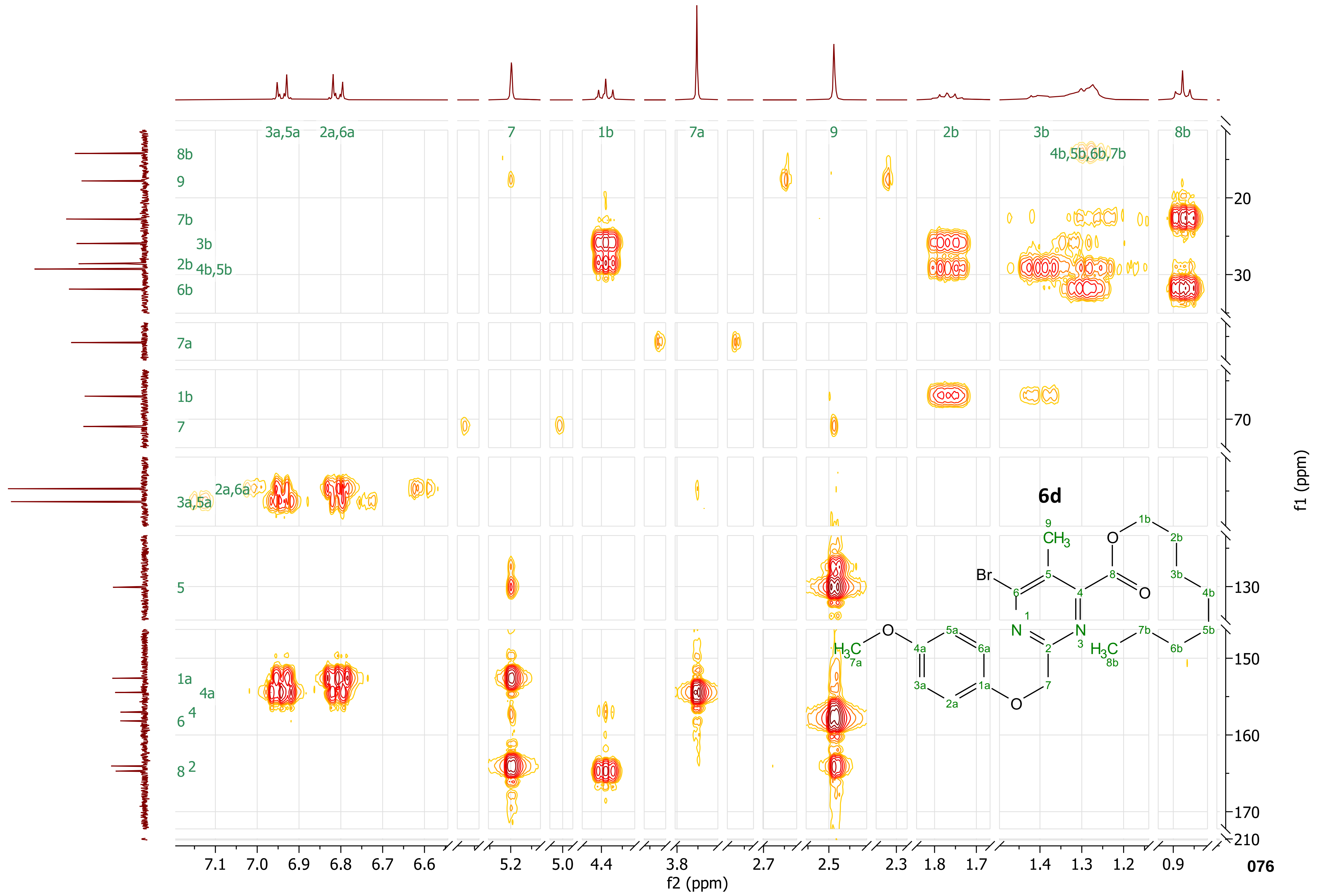

3a,5a
2a,6a
7
1b
7a
9
2b
3b
8b
4b,5b,6b,7b
8b
9
7b
3b
2b
4b,5b
6b
7a
1b
7
2a,6a
3a,5a
5
1a
4a
6
4
8
2
6d
Br
CH3
O
O
N
N
H3C
H3C
O
O
f1 (ppm)
f2 (ppm)

15N HMBC

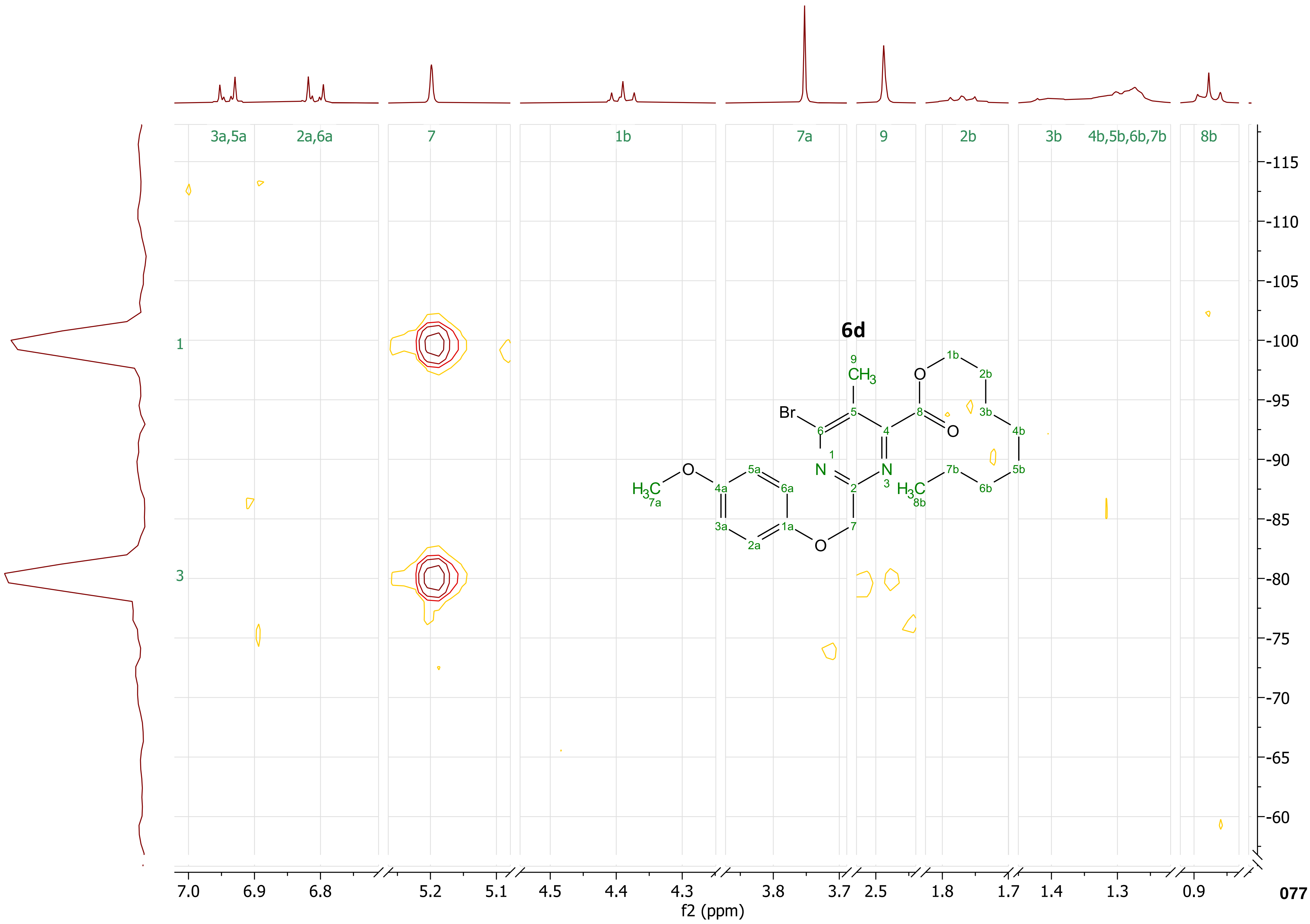

15N HMBC - f1 internal projection ^{15}N NMR (41 MHz, $CDCl_3$) δ -80.29, -99.75.

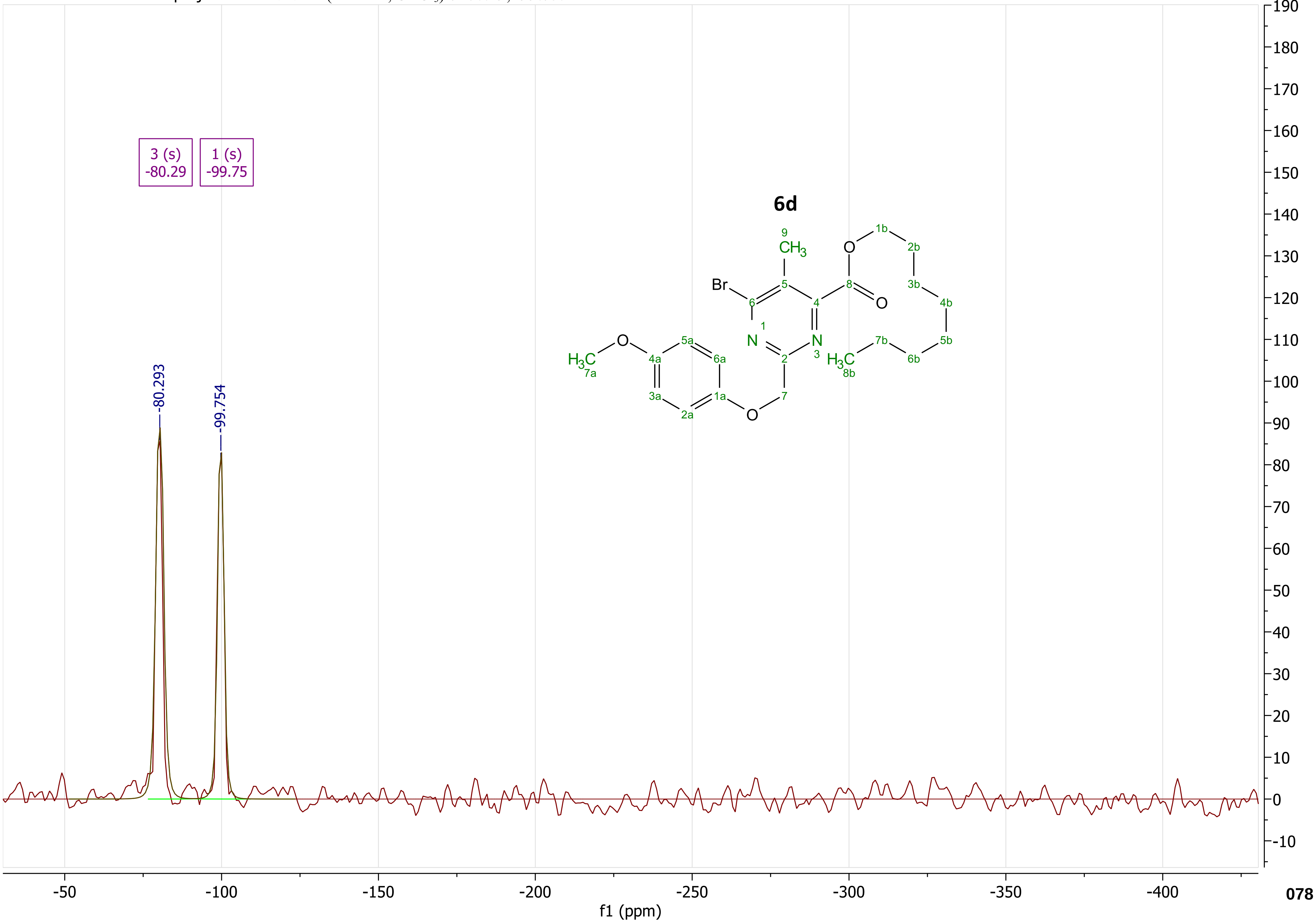

1H ^{1}H NMR (400 MHz, $CDCl_3$) δ 7.01 – 6.86 (m, 2H), 6.86 – 6.72 (m, 2H), 5.20 (s, 2H), 4.39 (t, *J* = 6.8 Hz, 2H), 3.76 (s, 3H), 2.49 (s, 3H), 1.85 – 1.68 (m, 2H), 1.45 – 1.36 (m, 2H), 1.37 – 1.18 (m, 10H), 0.88 (app t, *J* = 6.8 Hz, 3H).

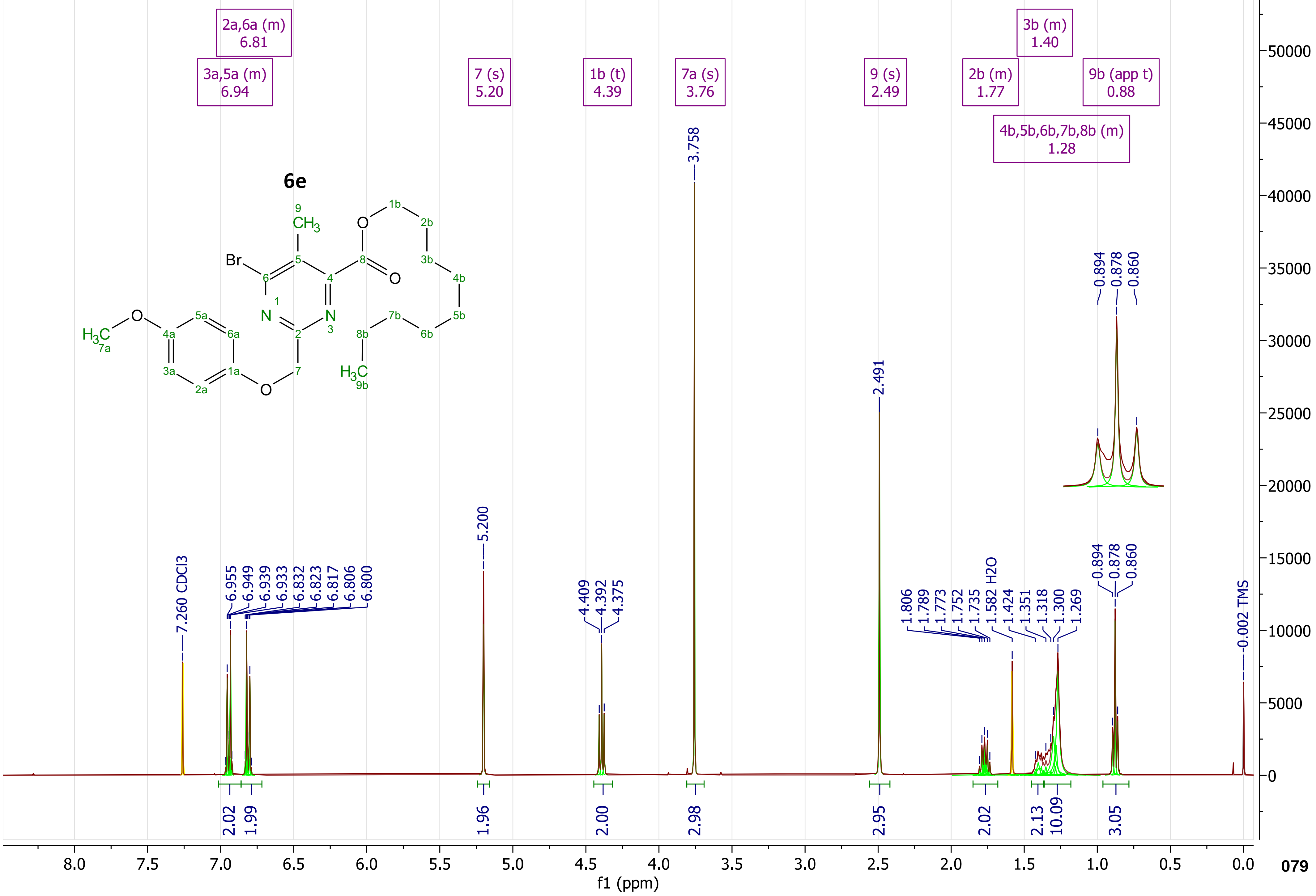

13C ^{13}C NMR (101 MHz, $CDCl_3$) δ 164.7, 164.0, 158.2, 157.0, 154.5, 152.6, 130.1, 116.4 (sym, 2C), 114.7 (sym, 2C), 70.9, 67.0, 55.8, 32.0, 29.6, 29.4, 29.3, 28.6, 26.0, 22.8, 17.8, 14.2.

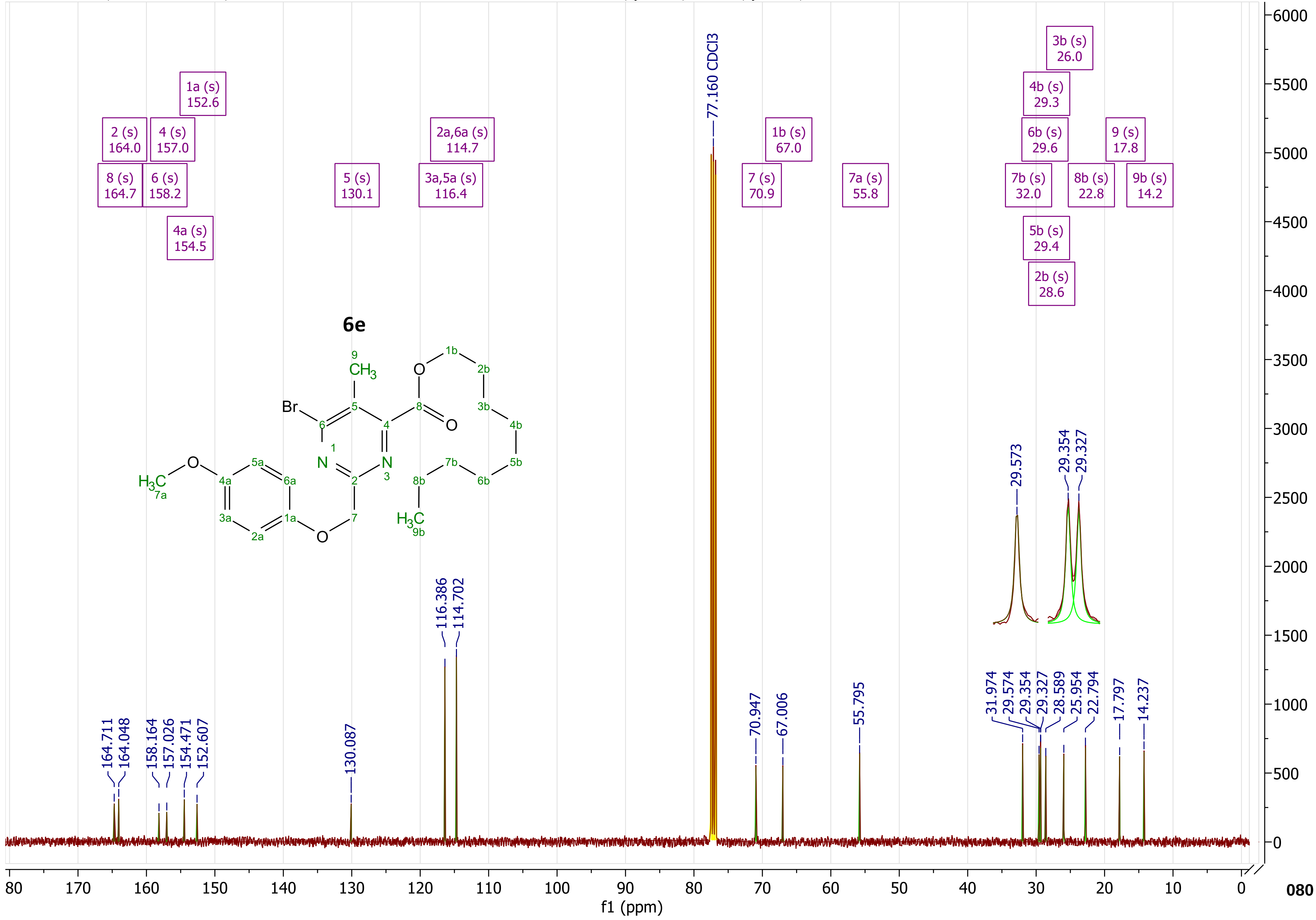

13C HSQC

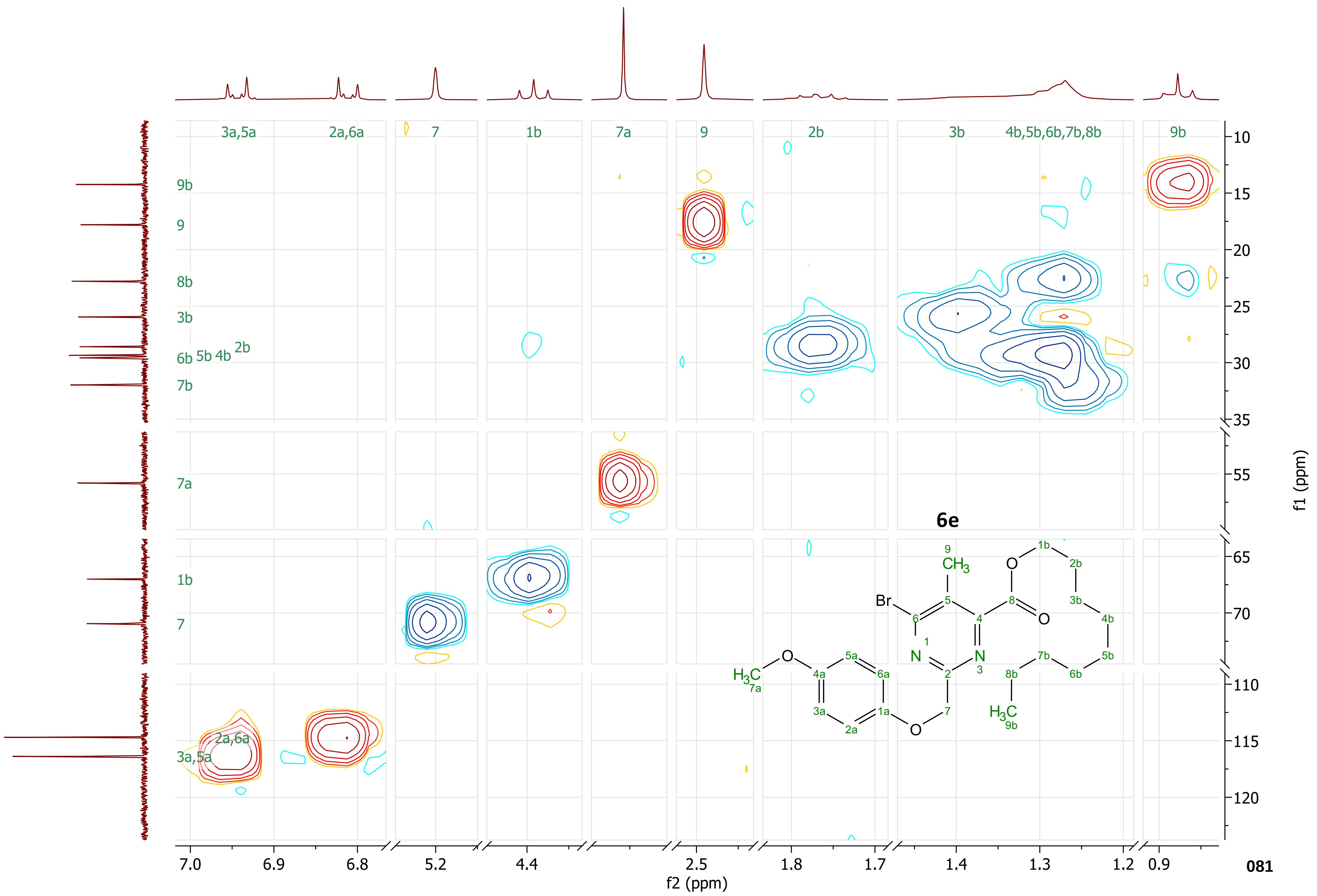
3a,5a
2a,6a
7
1b
7a
9
2b
3b
4b,5b,6b,7b,8b
9b
6b 5b 4b
8b
7b
1b
f1 (ppm)
f2 (ppm)
6e
Br
CH3
H3C
O
N

13C HMBC

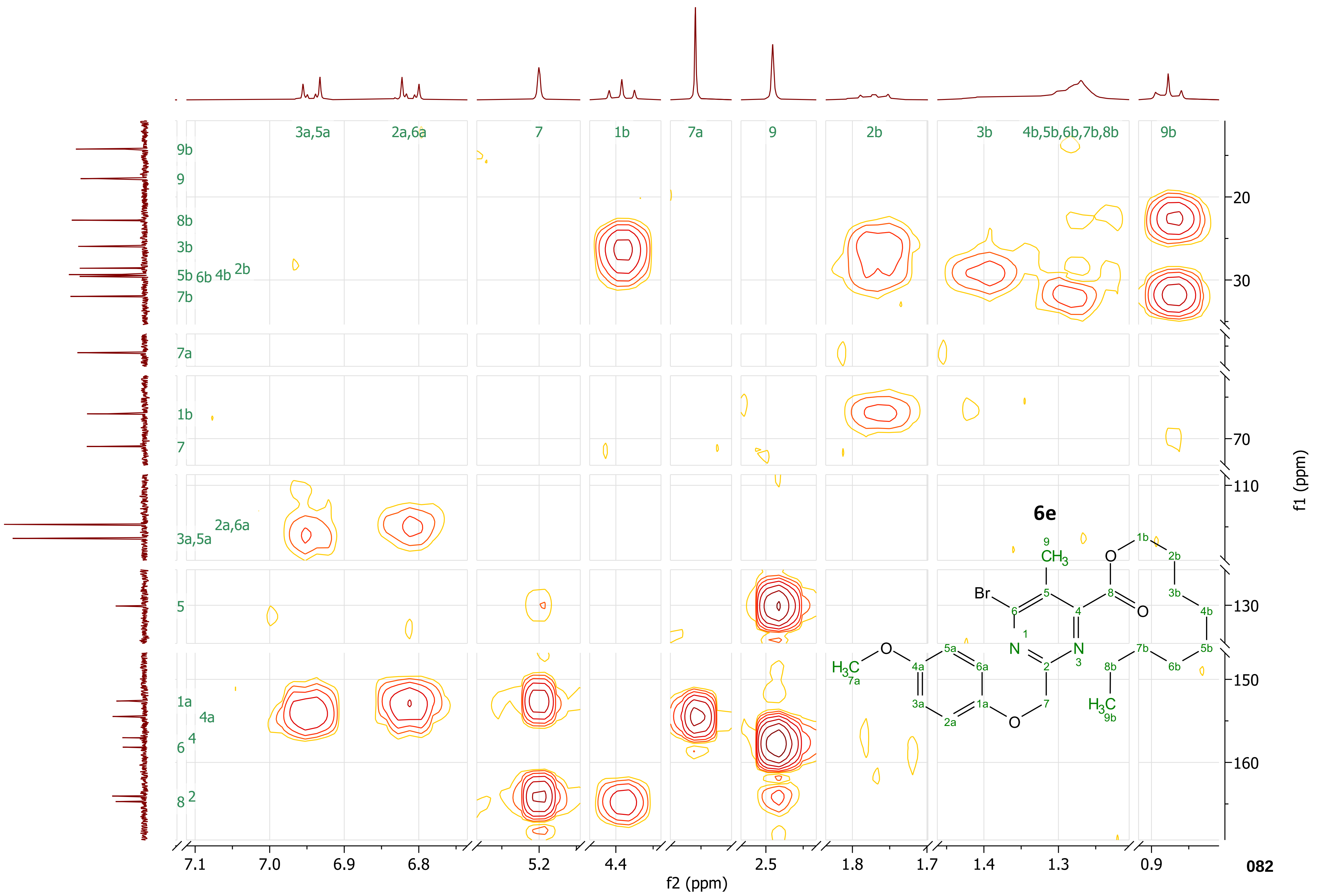

3a,5a
2a,6a
7
1b
7a
9
2b
3b
4b,5b,6b,7b,8b
9b
8b
5b 6b 4b 2b
7b
1b
3a,5a
1a
4a
4
6
8 2
6e
Br
CH3
O
N
H3C
f2 (ppm)
f1 (ppm)

15N HMBC

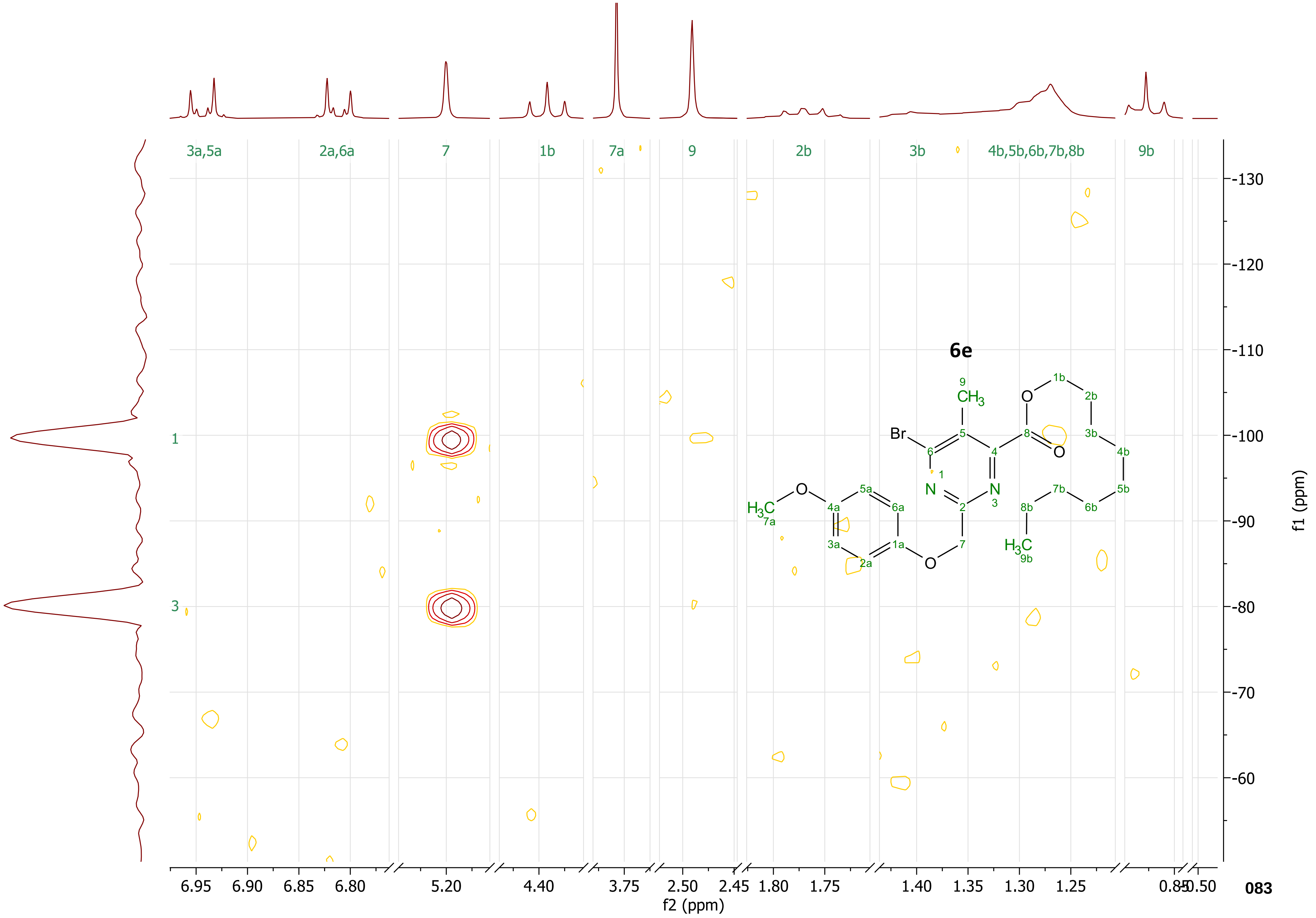

15N HMBC - f1 internal projection ^{15}N NMR (41 MHz, $CDCl_3$) δ -80.12, -99.68.

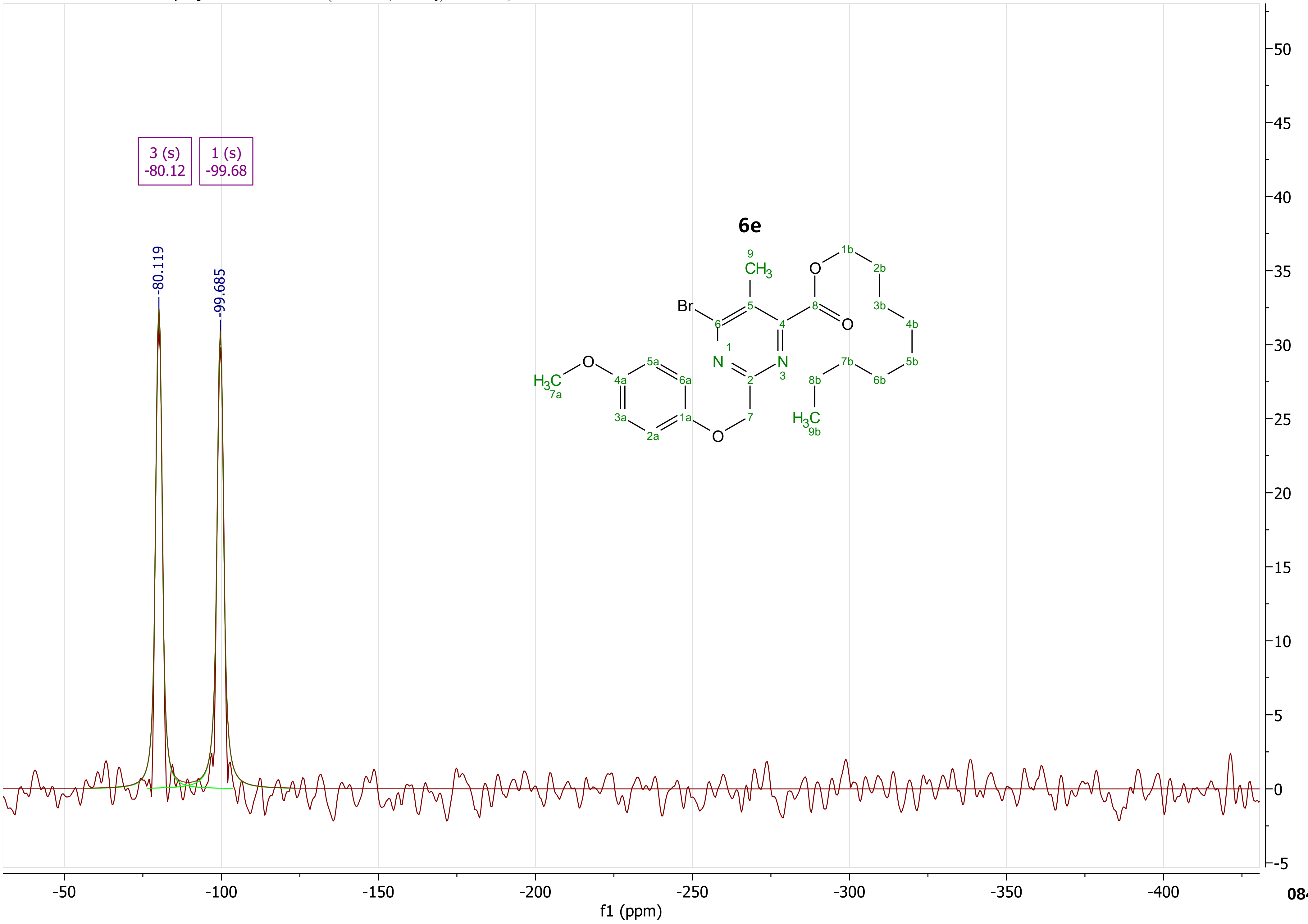

1H ^{1}H NMR (400 MHz, $CDCl_3$) δ 7.05 – 6.90 (m, 2H), 6.90 – 6.71 (m, 2H), 5.20 (s, 2H), 4.47 (q, J = 7.2 Hz, 2H), 3.75 (s, 3H), 2.49 (s, 3H), 1.42 (t, J = 7.1 Hz, 3H).

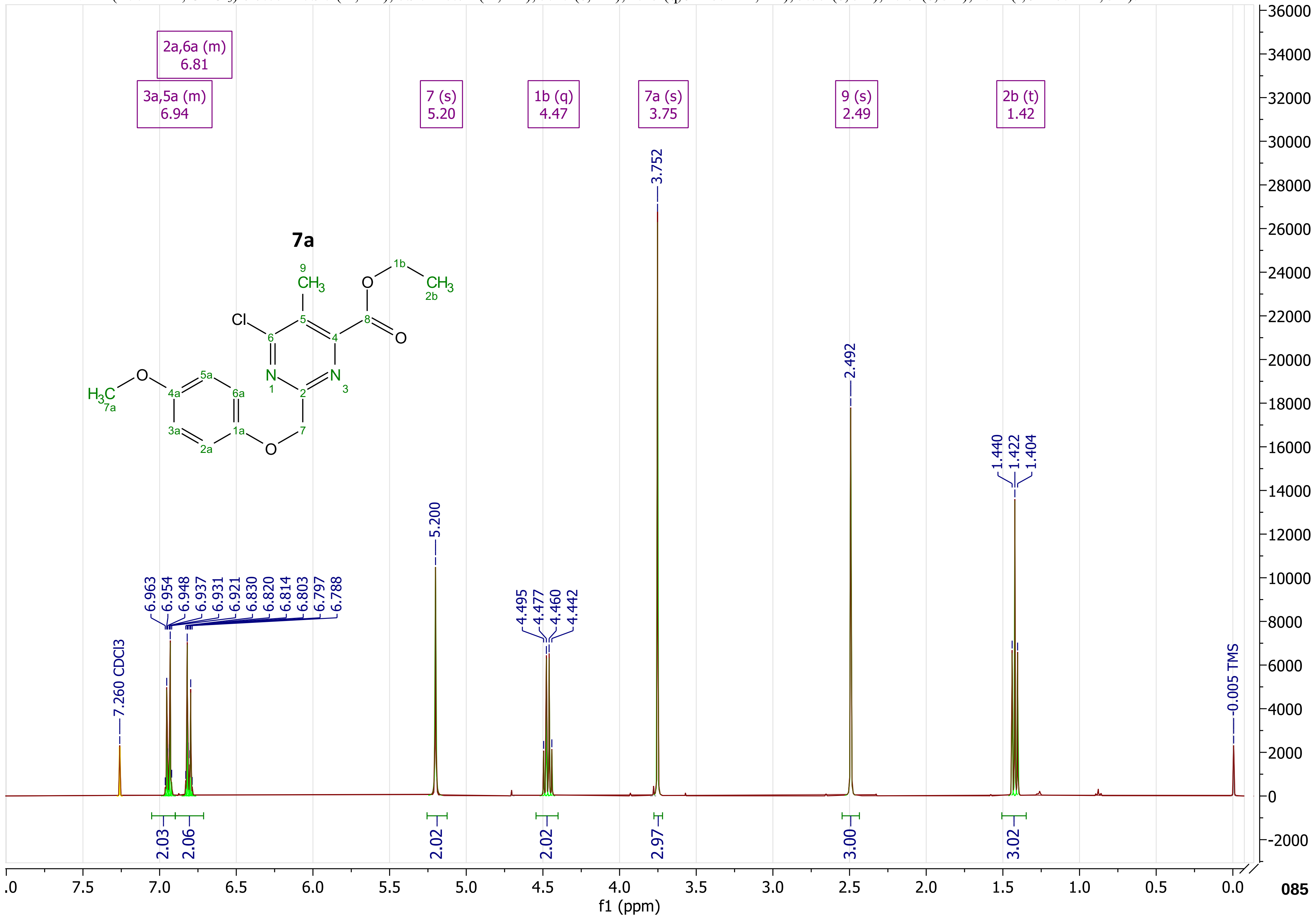

13C ^{13}C NMR (101 MHz, $CDCl_3$) δ 164.6, 164.10, 164.09, 157.6, 154.5, 152.6, 127.7, 116.3 (sym, 2C), 114.7 (sym, 2C), 71.0, 62.9, 55.8, 15.2, 14.2.

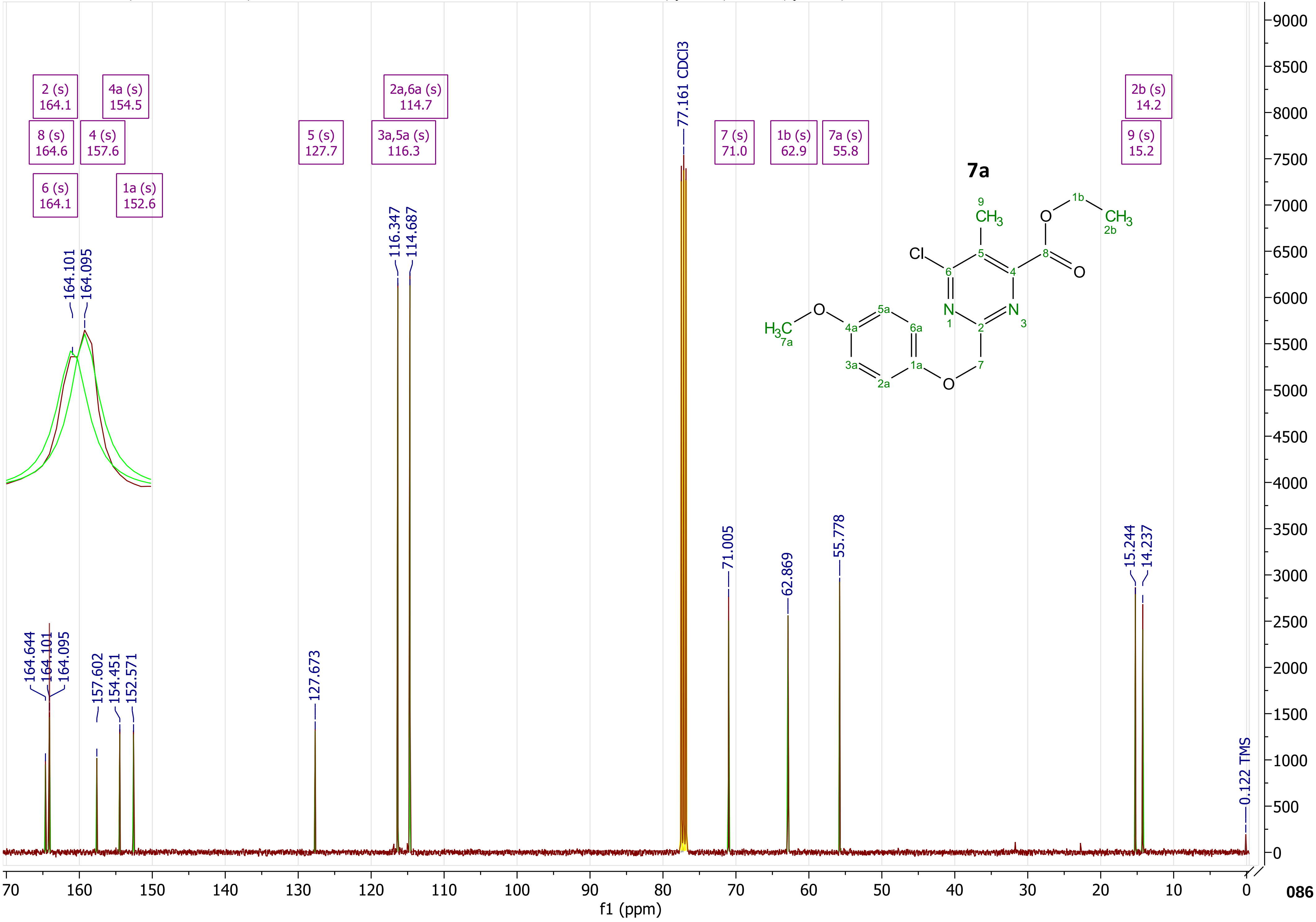

13C HSQC

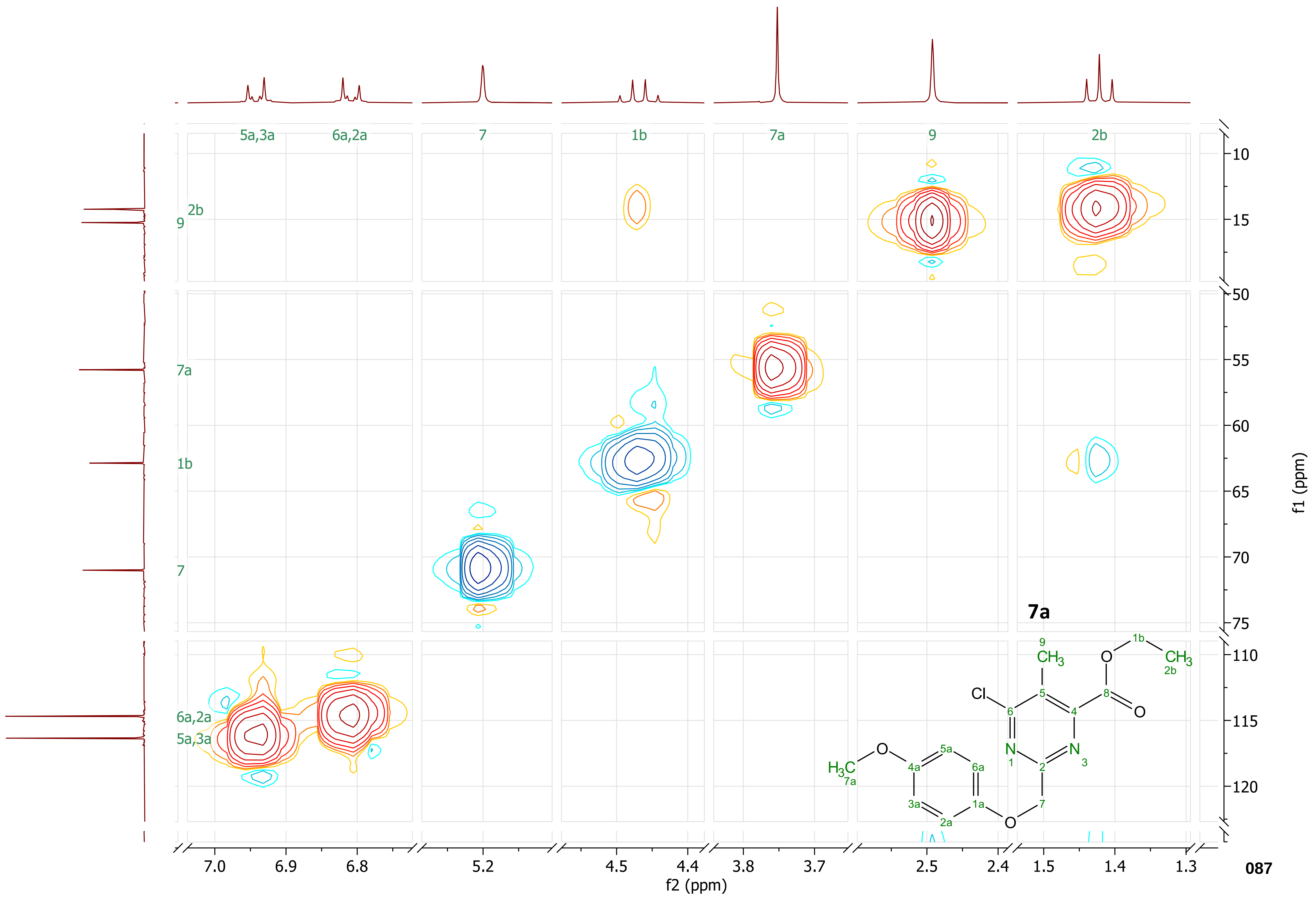
5a,3a
6a,2a
7
1b
7a
9
2b
f1 (ppm)
f2 (ppm)
7a
H3C
O
4a
5a
6a
3a
2a
1a
O
7
N
1
2
N
3
6
5
4
Cl
CH3
9
8
O
1b
CH3
2b
O

13C HMBC

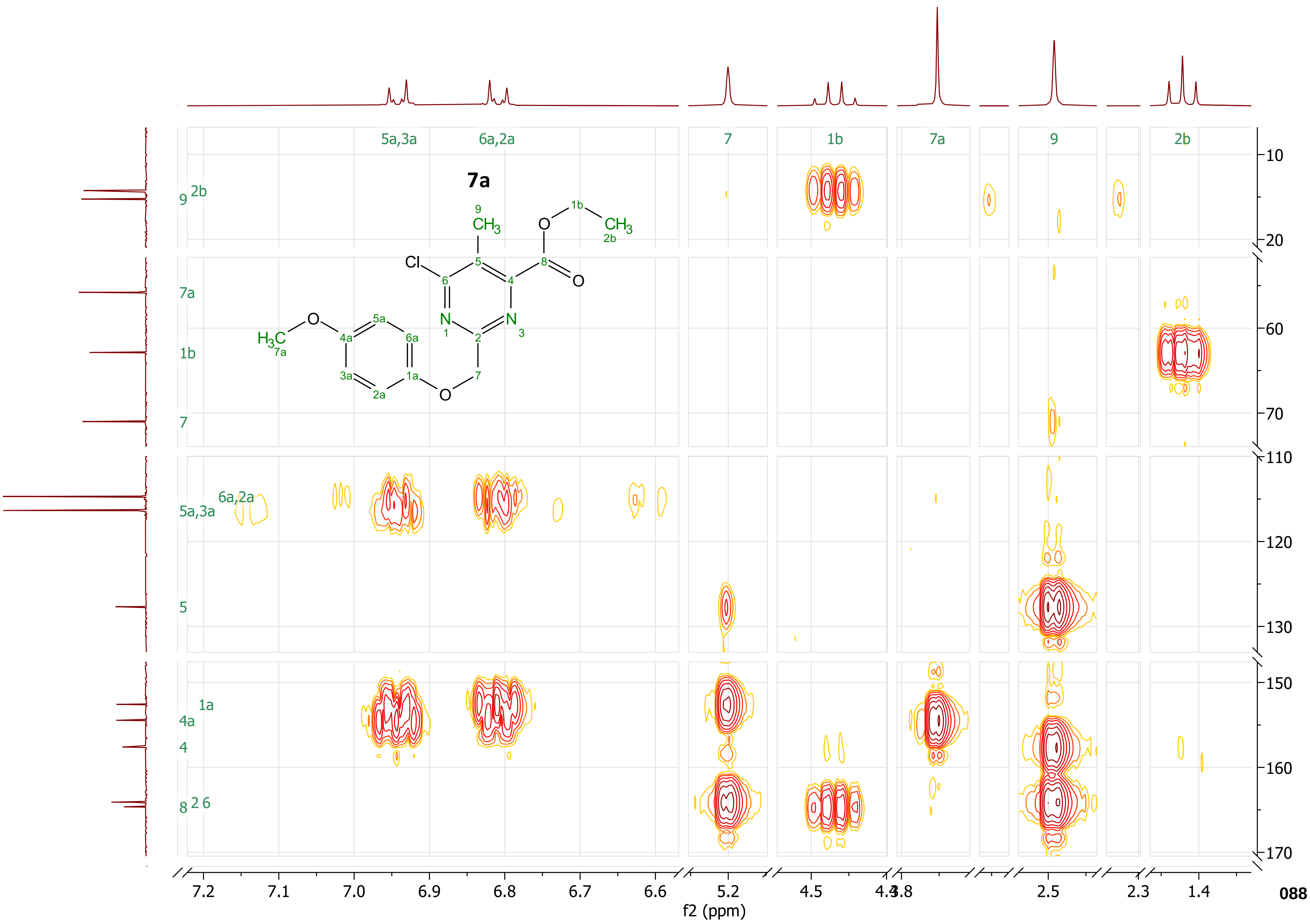
5a,3a
6a,2a
7
1b
7a
9
2b
7a
CH3
Cl
O
O
CH3
N
N
H3C
O
O
9
2b
7a
1b
7
6a,2a
5a,3a
5
1a
4a
4
8 2 6
f2 (ppm)
f1 (ppm)

15N HMBC

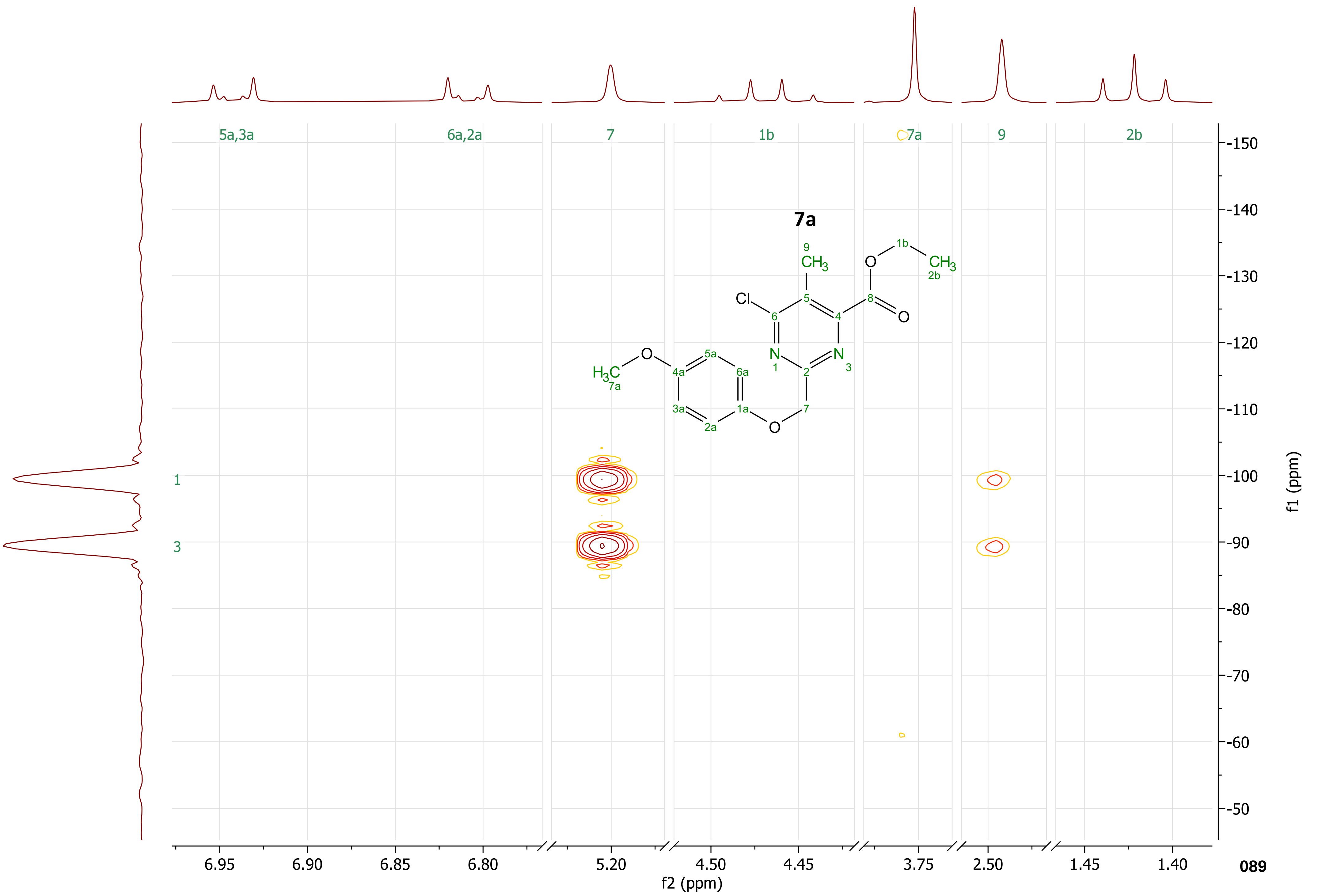

5a,3a
6a,2a
7
1b
7a
9
2b
7a
Cl
CH3
O
O
N
N
H3C
O
O
1
3
6.95
6.90
6.85
6.80
5.20
4.50
4.45
3.75
2.50
1.45
1.40
f2 (ppm)
f1 (ppm)
-150
-140
-130
-120
-110
-100
-90
-80
-70
-60
-50

15N HMBC - f1 internal projection ^{15}N NMR (41 MHz, $CDCl_3$) δ -89.37, -99.45.

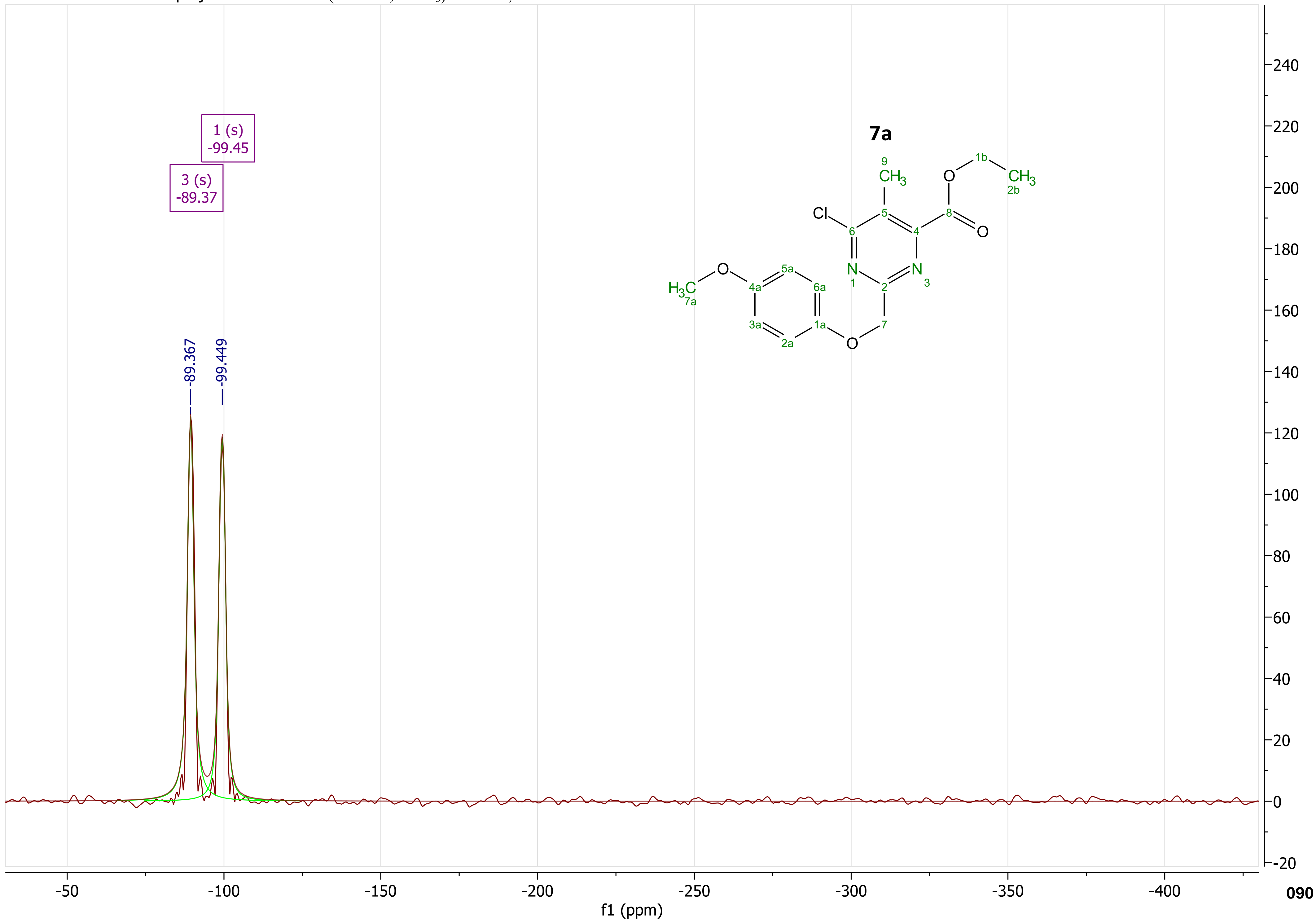

1H

^{1}H NMR (400 MHz, CDCl$_3$) δ 6.94 (d, J = 0.6 Hz, 2H), 6.85 – 6.76 (m, 2H), 5.20 (app d, J = 0.6 Hz, 2H), 4.41 (t, J = 6.7 Hz, 2H), 3.75 (s, 3H), 2.48 (app t, J = 0.6 Hz, 3H), 1.82 – 1.70 (m, 2H), 1.51 – 1.37 (m, 2H), 0.97 (t, J = 7.4 Hz, 3H).

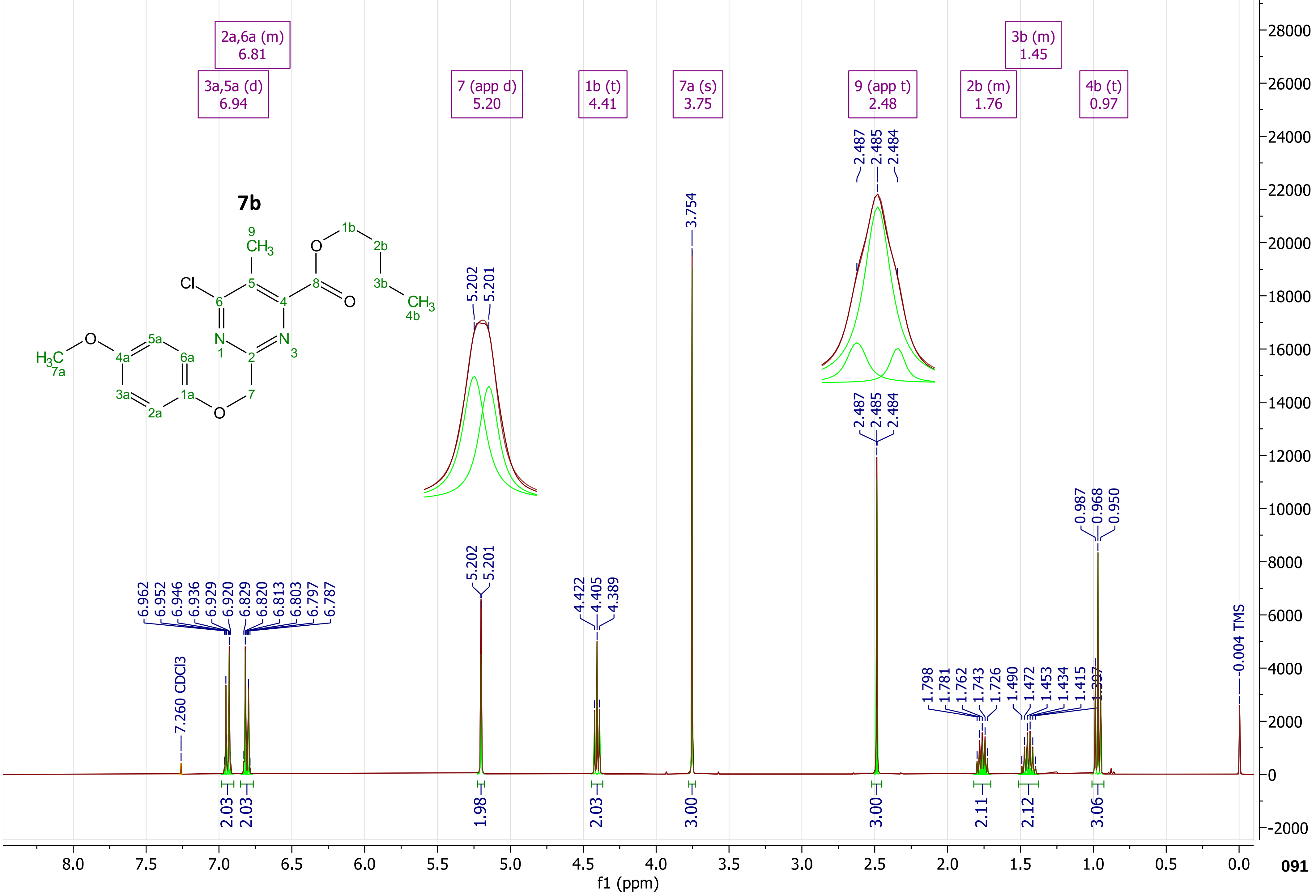

13C ^{13}C NMR (101 MHz, $CDCl_3$) δ 164.8, 164.12, 164.06, 157.7, 154.4, 152.6, 127.5, 116.3(sym, 2C), 114.7 (sym, 2C), 71.0, 66.7, 55.8, 30.6, 19.2, 15.3, 13.8.

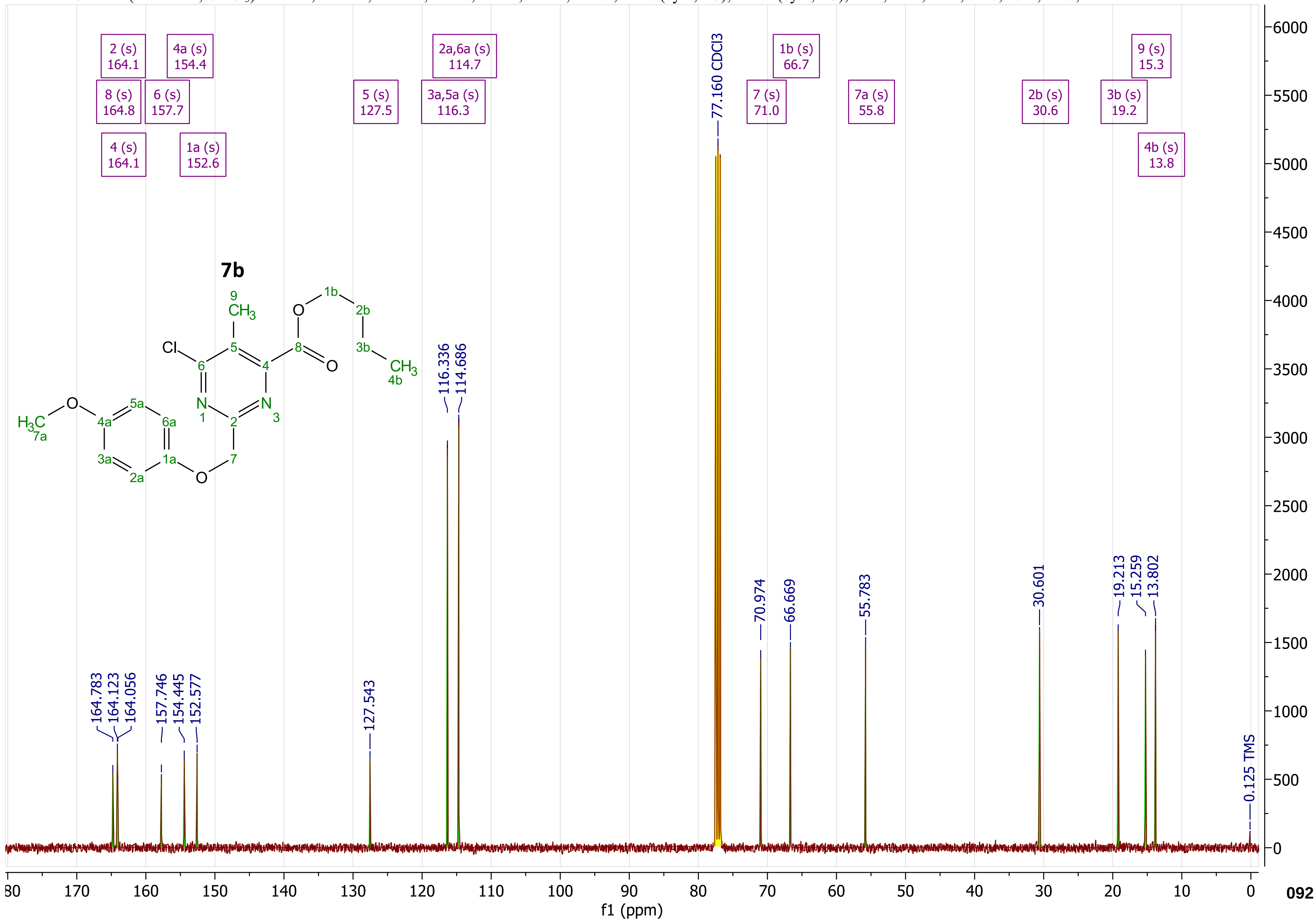

13C HSQC

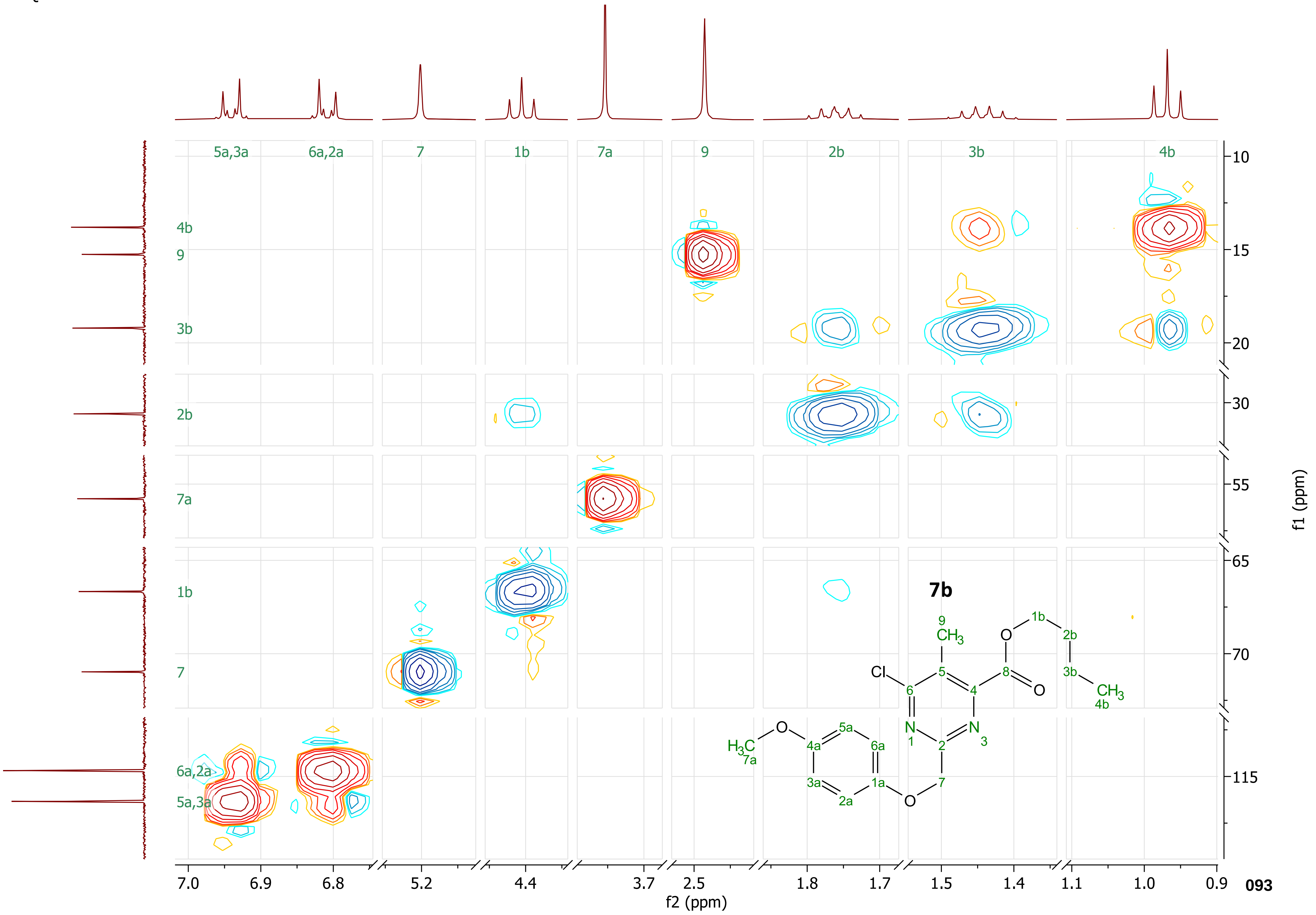

5a,3a
6a,2a
7
1b
7a
9
2b
3b
4b
4b
9
3b
2b
7a
1b
7
6a,2a
5a,3a
7b
f2 (ppm)
f1 (ppm)

13C HMBC

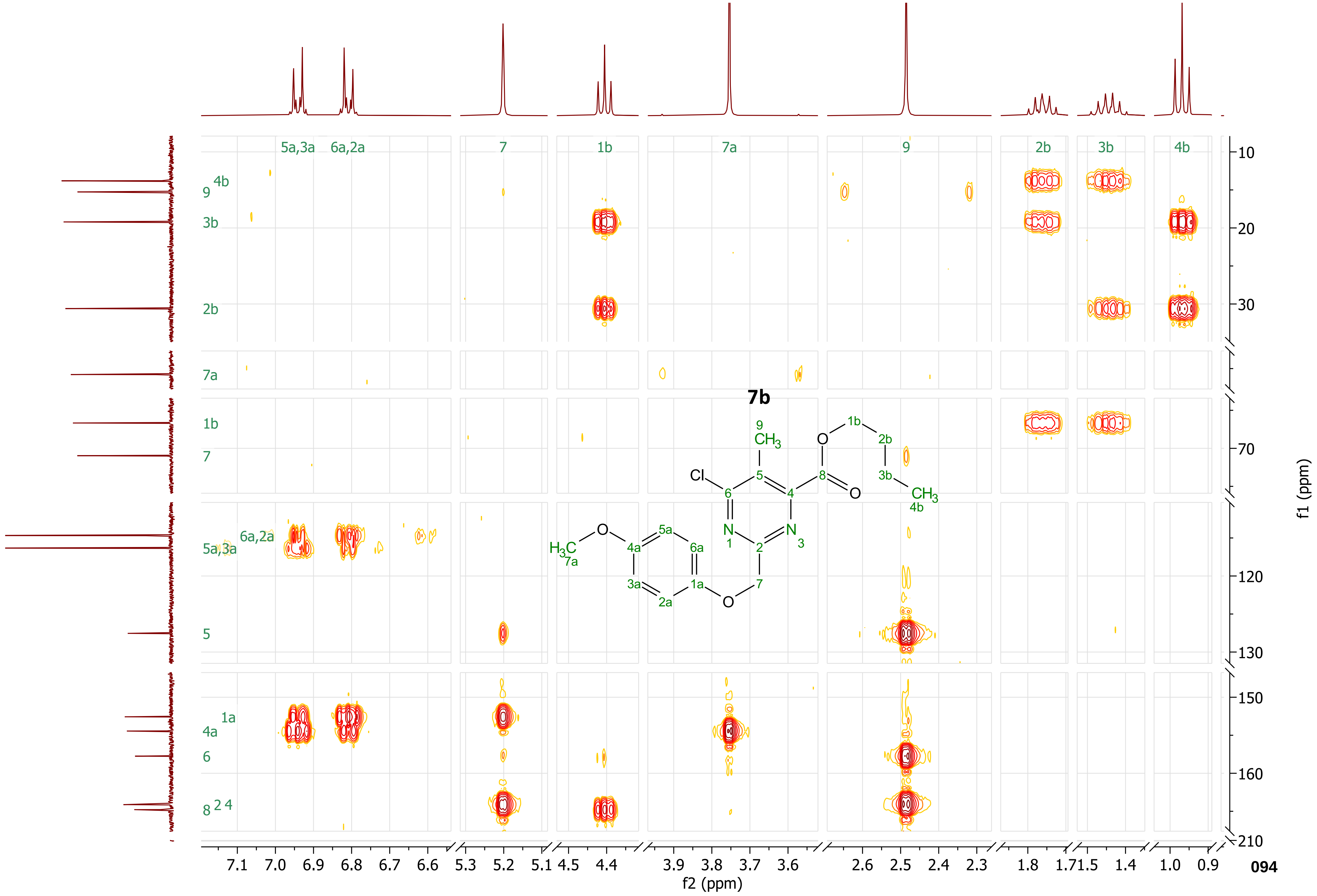
7b
5a,3a
6a,2a
7
1b
7a
9
2b
3b
4b
4b
9
3b
2b
7a
1b
7
5a,3a
6a,2a
5
1a
4a
6
8 2 4
f2 (ppm)
f1 (ppm)
Cl
N
O
CH3
H3C

15N HSQC

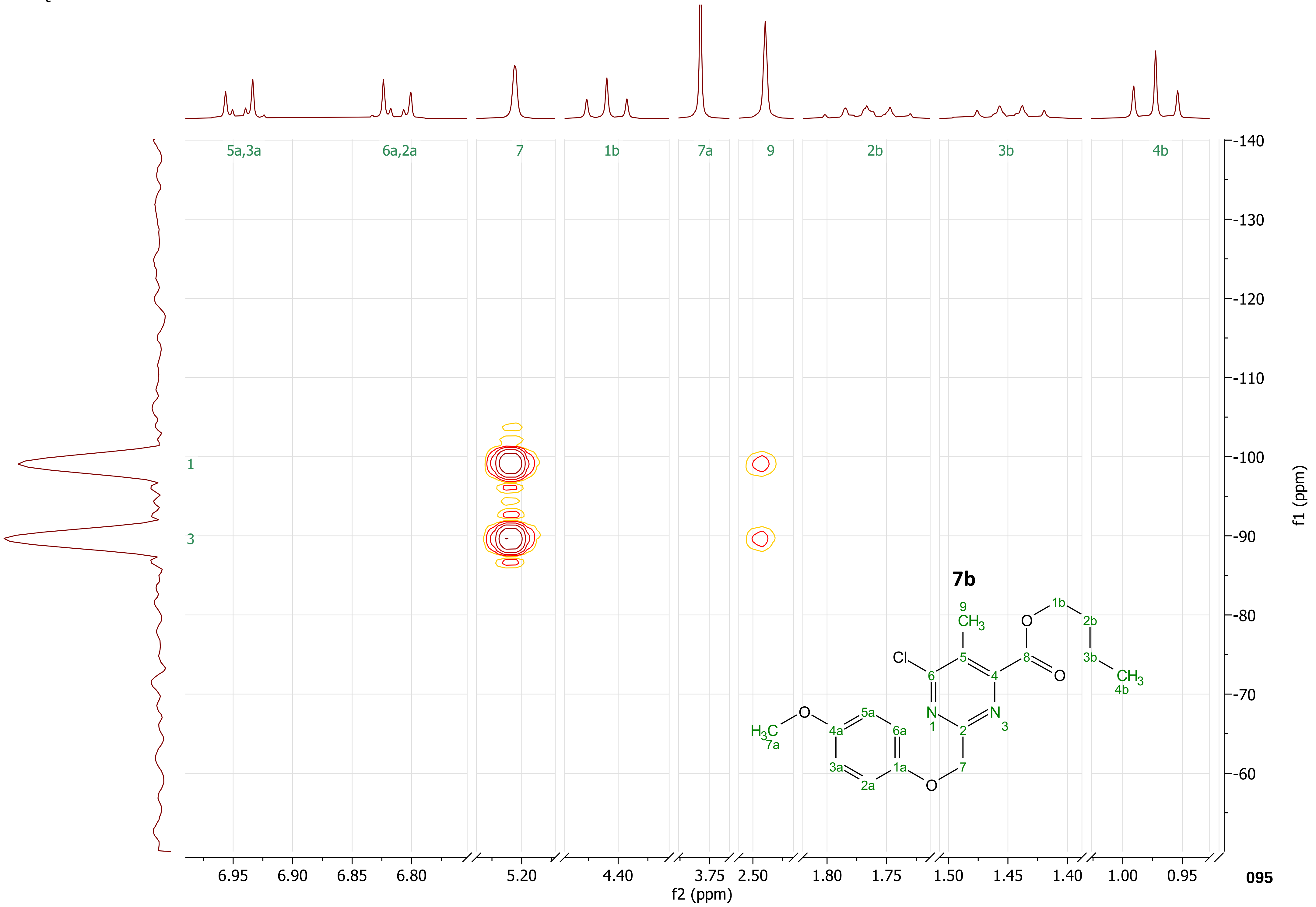
5a,3a
6a,2a
7
1b
7a
9
2b
3b
4b
1
3
7b
f1 (ppm)
f2 (ppm)
6.95
6.90
6.85
6.80
5.20
4.40
3.75
2.50
1.80
1.75
1.50
1.45
1.40
1.00
0.95
-140
-130
-120
-110
-100
-90
-80
-70
-60

15N HMBC - f1 internal projection ^{15}N NMR (41 MHz, $CDCl_3$) δ -89.67, -99.15.

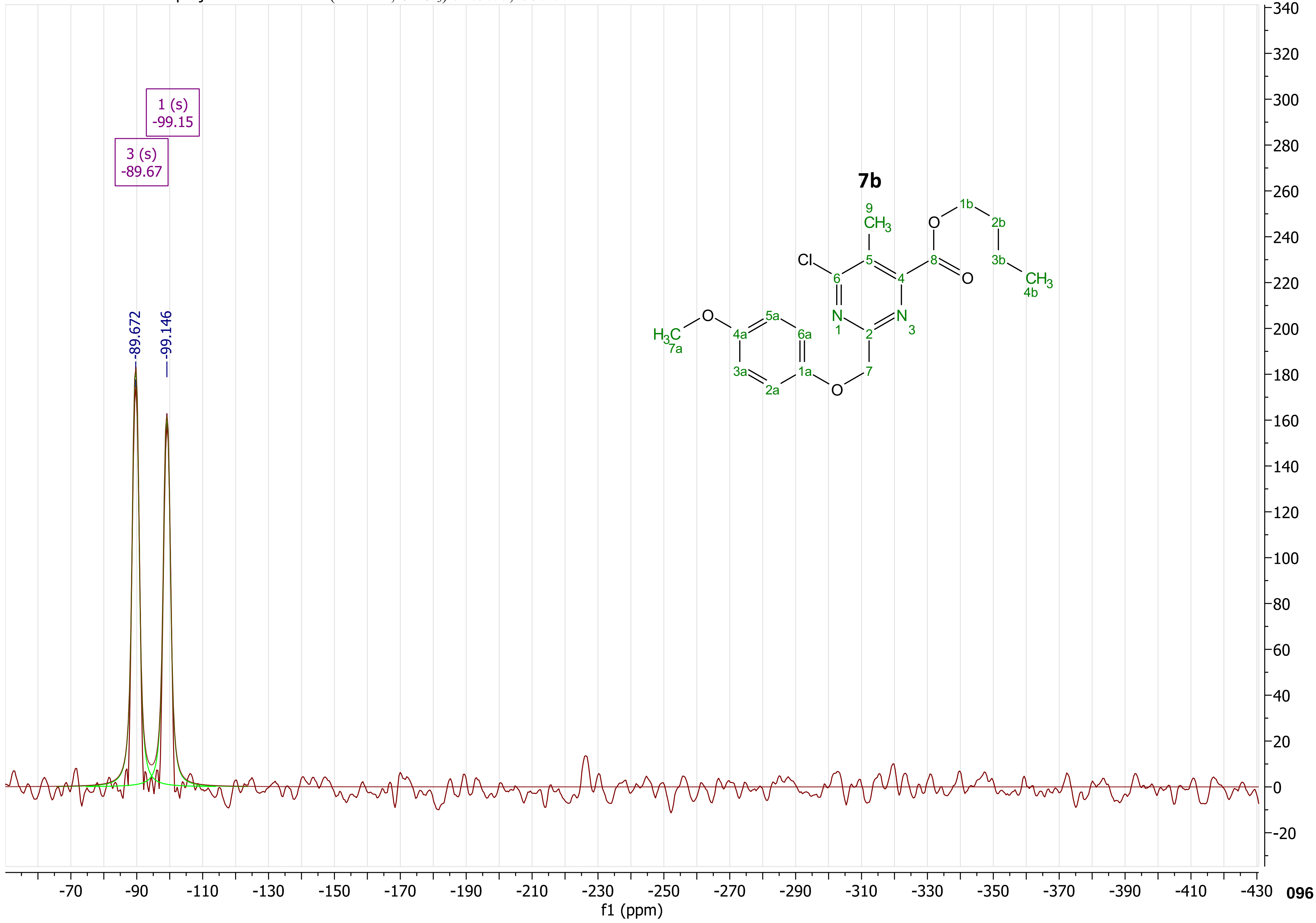

1H ^{1}H NMR (400 MHz, $CDCl_3$) δ 6.99 – 6.90 (m, 2H), 6.85 – 6.77 (m, 2H), 5.20 (app d, *J* = 0.7 Hz, 2H), 4.40 (t, *J* = 6.8 Hz, 2H), 3.76 (s, 3H), 2.49 (app t, *J* = 0.7 Hz, 3H), 1.84 – 1.72 (m, 2H), 1.47 – 1.35 (m, 2H), 1.38 – 1.23 (m, 6H), 0.89 (app t, *J* = 6.9 Hz, 3H).

7c

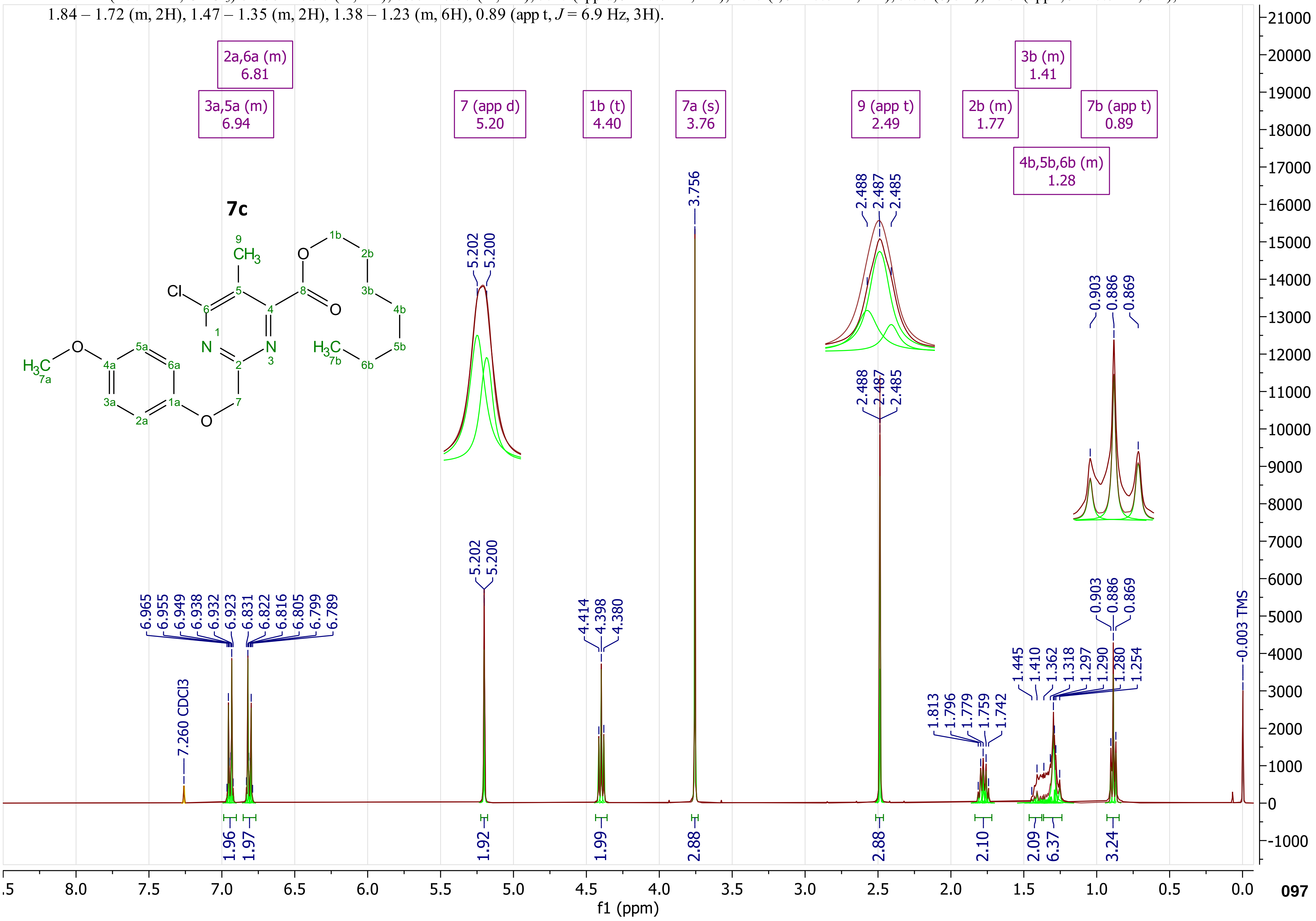

13C ^{13}C NMR (101 MHz, $CDCl_3$) δ 164.8, 164.13, 164.07, 157.8, 154.5, 152.6, 127.5, 116.4 (sym, 2C), 114.7 (sym, 2C), 71.0, 67.0, 55.8, 31.8, 29.0, 28.6, 25.9, 22.7, 15.3, 14.2.

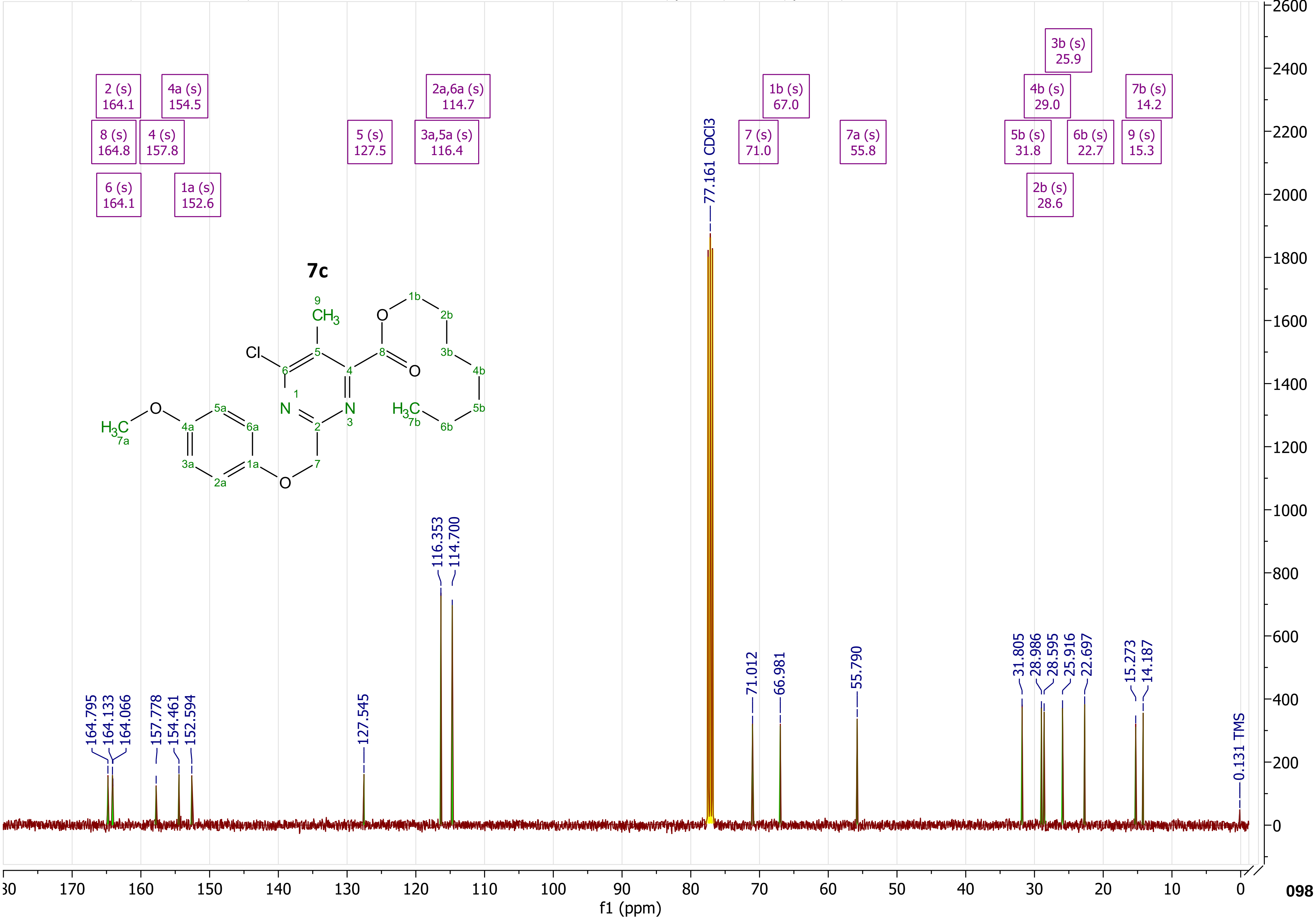

13C HSQC

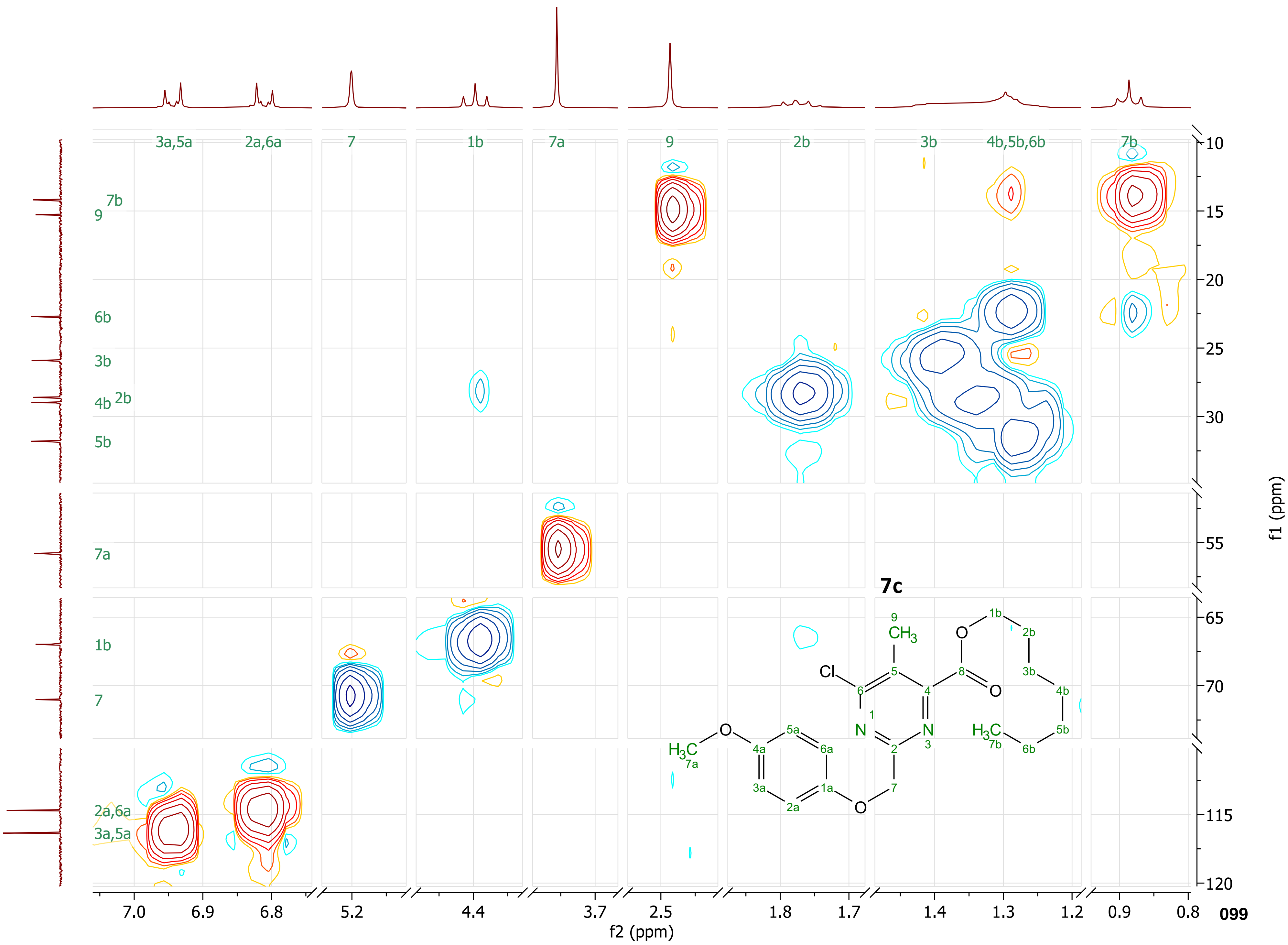

3a,5a
2a,6a
7
1b
7a
9
2b
3b
4b,5b,6b
7b
6b
3b
4b
2b
5b
1b
2a,6a
3a,5a
7c
CH3
Cl
O
N
H3C
f2 (ppm)
f1 (ppm)

13C HMBC

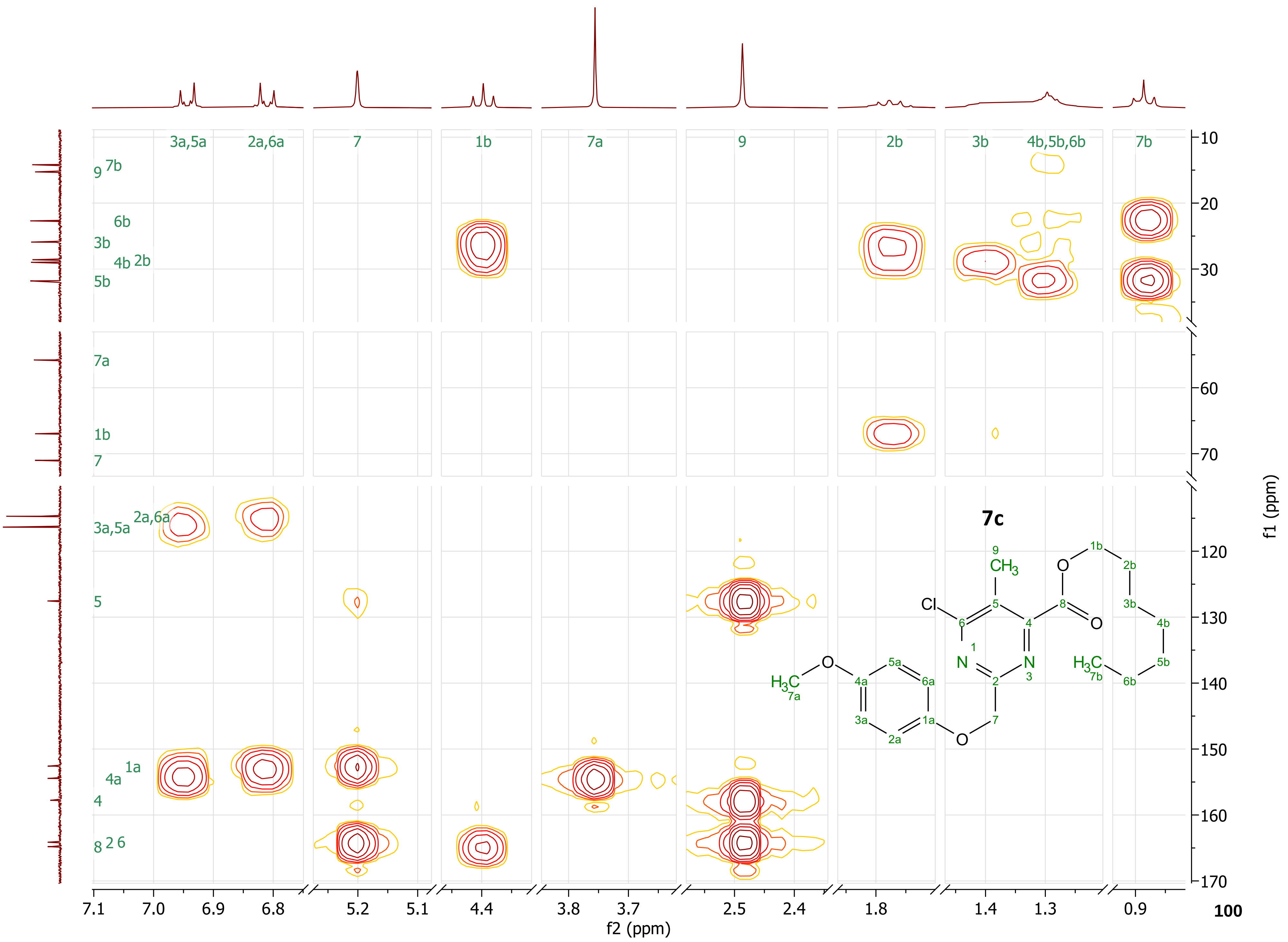

3a,5a
2a,6a
7
1b
7a
9
2b
3b
4b,5b,6b
7b
9 7b
6b
3b
4b 2b
5b
7a
1b
7
3a,5a 2a,6a
5
1a
4a
4
8 2 6
7c
f1 (ppm)
f2 (ppm)

15N HMBC

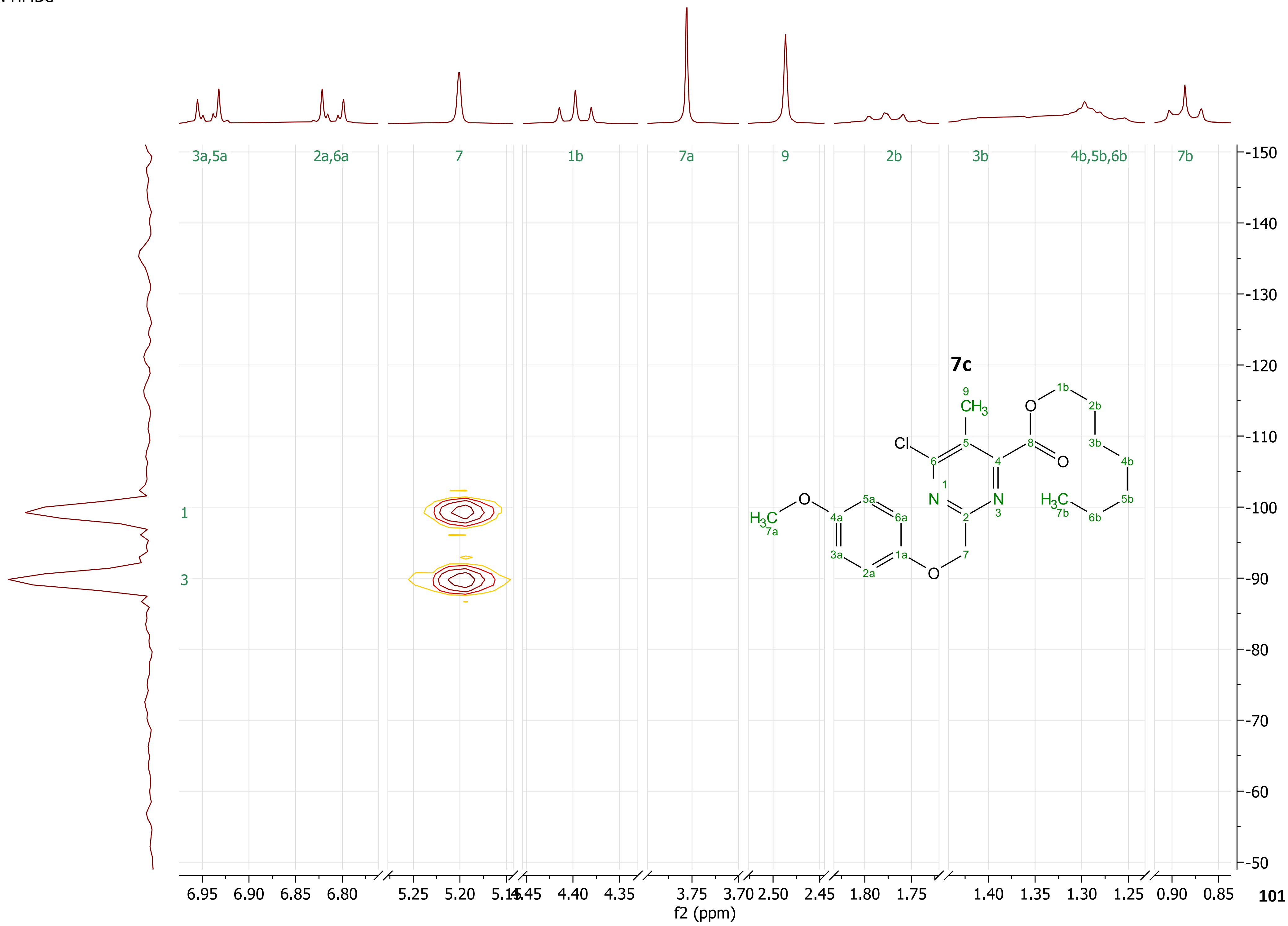

15N HMBC - f1 internal projection ^{15}N NMR (41 MHz, $CDCl_3$) δ -89.80, -99.27.

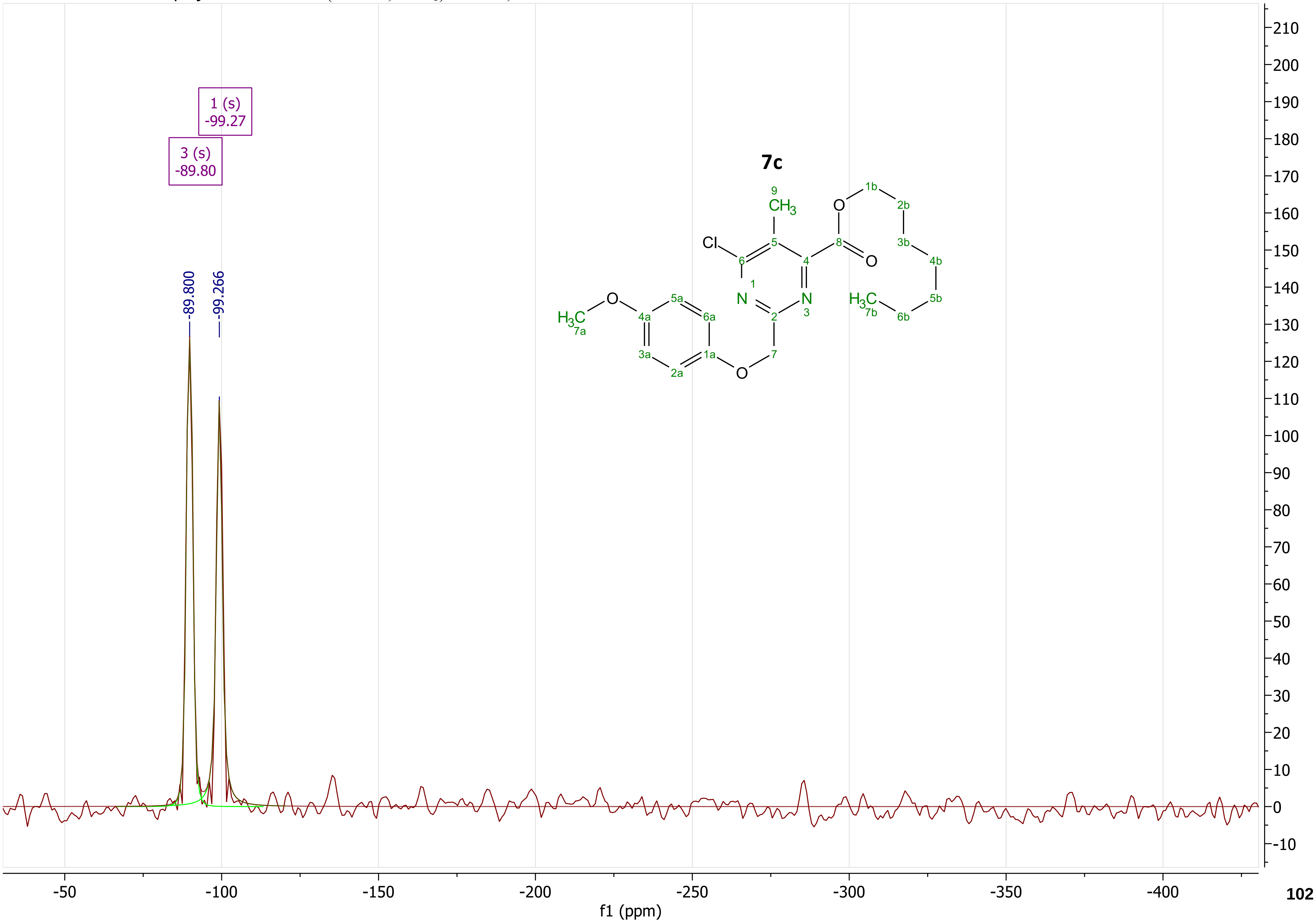

1H ^{1}H NMR (400 MHz, $CDCl_3$) δ 6.99 – 6.90 (m, 2H), 6.85 – 6.77 (m, 2H), 5.20 (app d, *J* = 0.6 Hz, 2H), 4.40 (t, *J* = 6.8 Hz, 2H), 3.75 (s, 3H), 2.49 (app t, *J* = 0.6 Hz, 3H), 1.83 – 1.71 (m, 2H), 1.47 – 1.37 (m, 2H), 1.36 – 1.22 (m, 8H), 0.88 (app t, *J* = 7.0 Hz, 3H).

7d

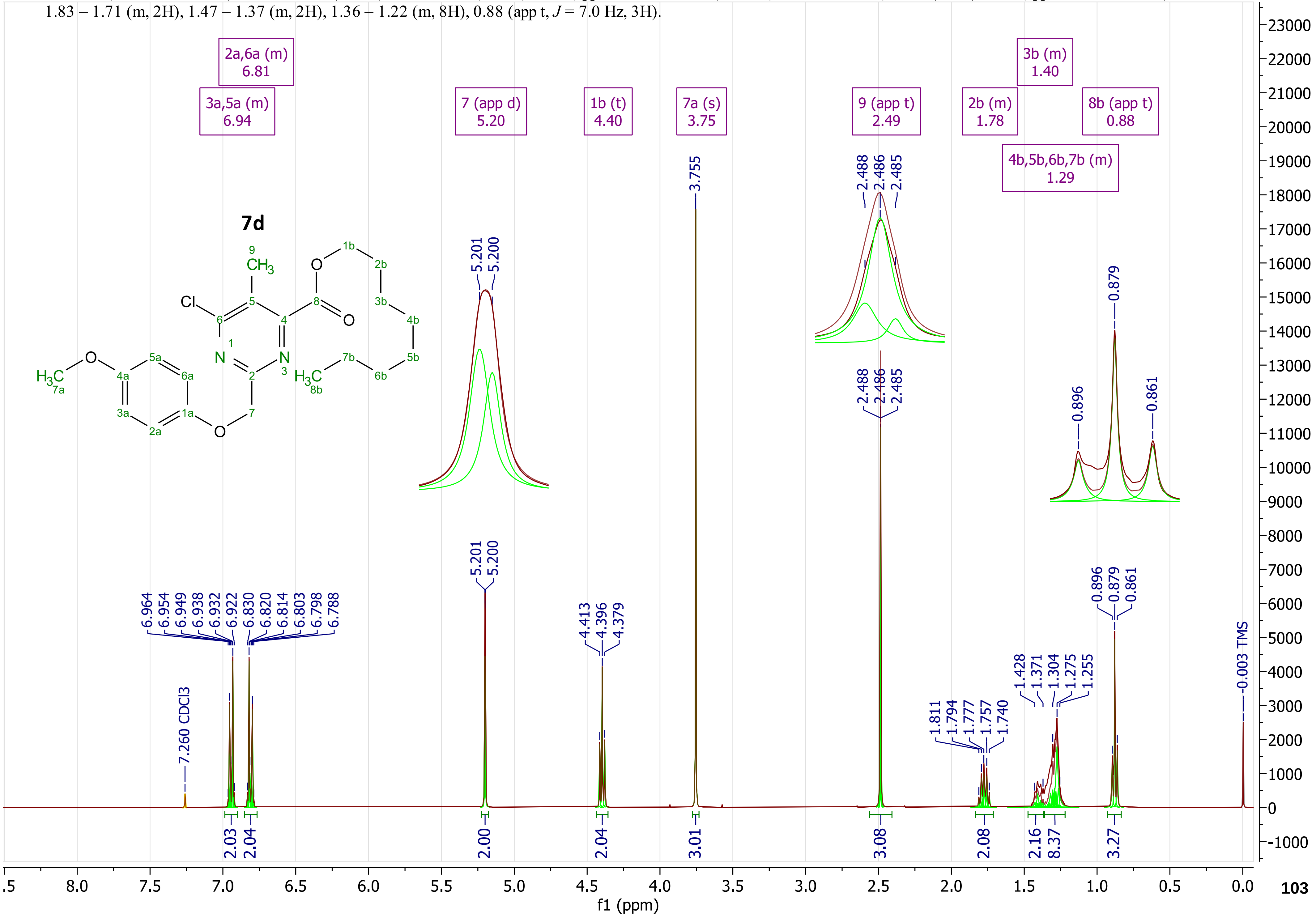

13C ^{13}C NMR (101 MHz, $CDCl_3$) δ 164.8, 164.13, 164.06, 157.8, 154.5, 152.6, 127.5, 116.3 (sym, 2C), 114.7 (sym, 2C), 71.0, 67.0, 55.8, 31.9, 29.3 (2C), 28.6, 26.0, 22.8, 15.3, 14.2.

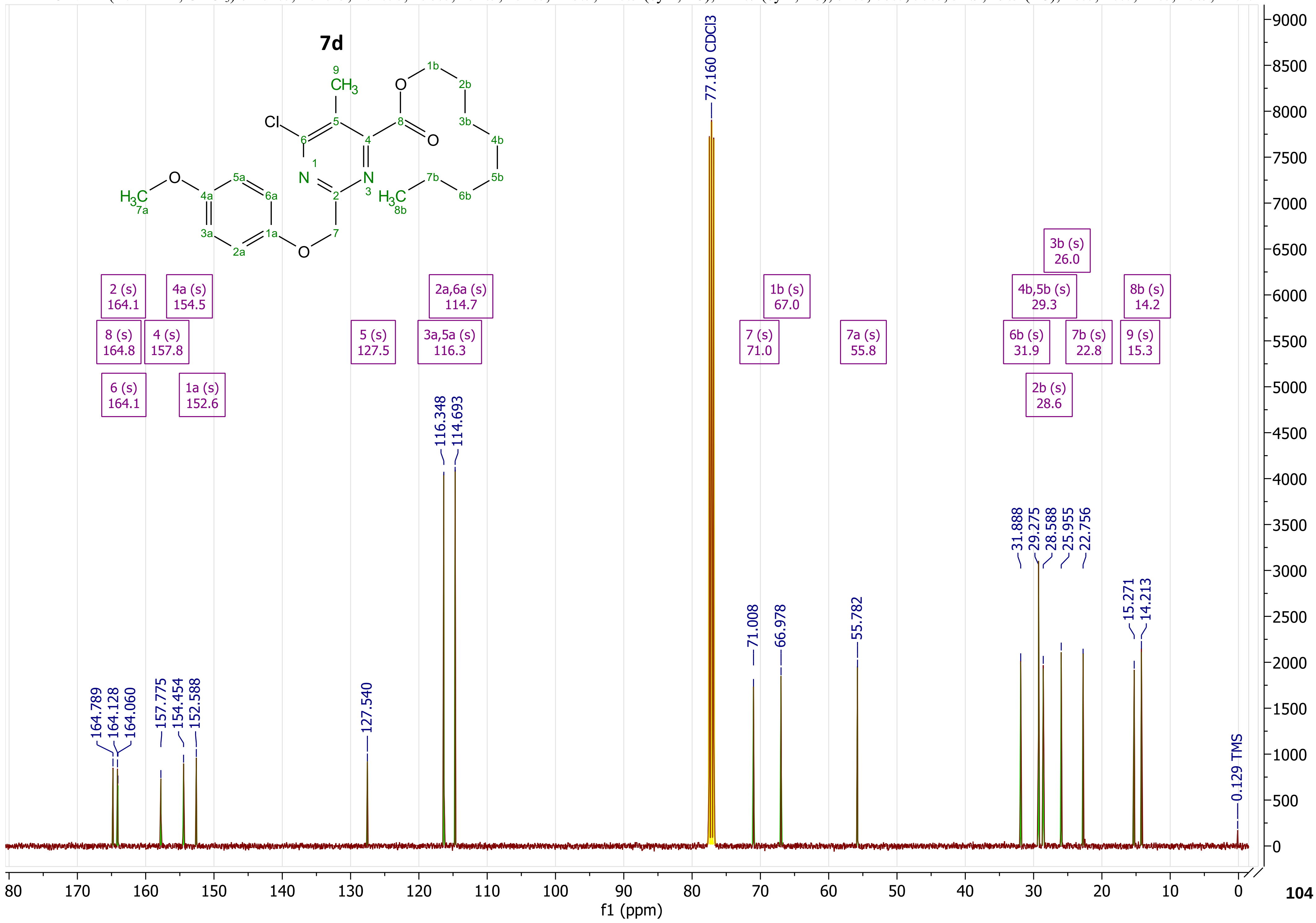

13C HSQC

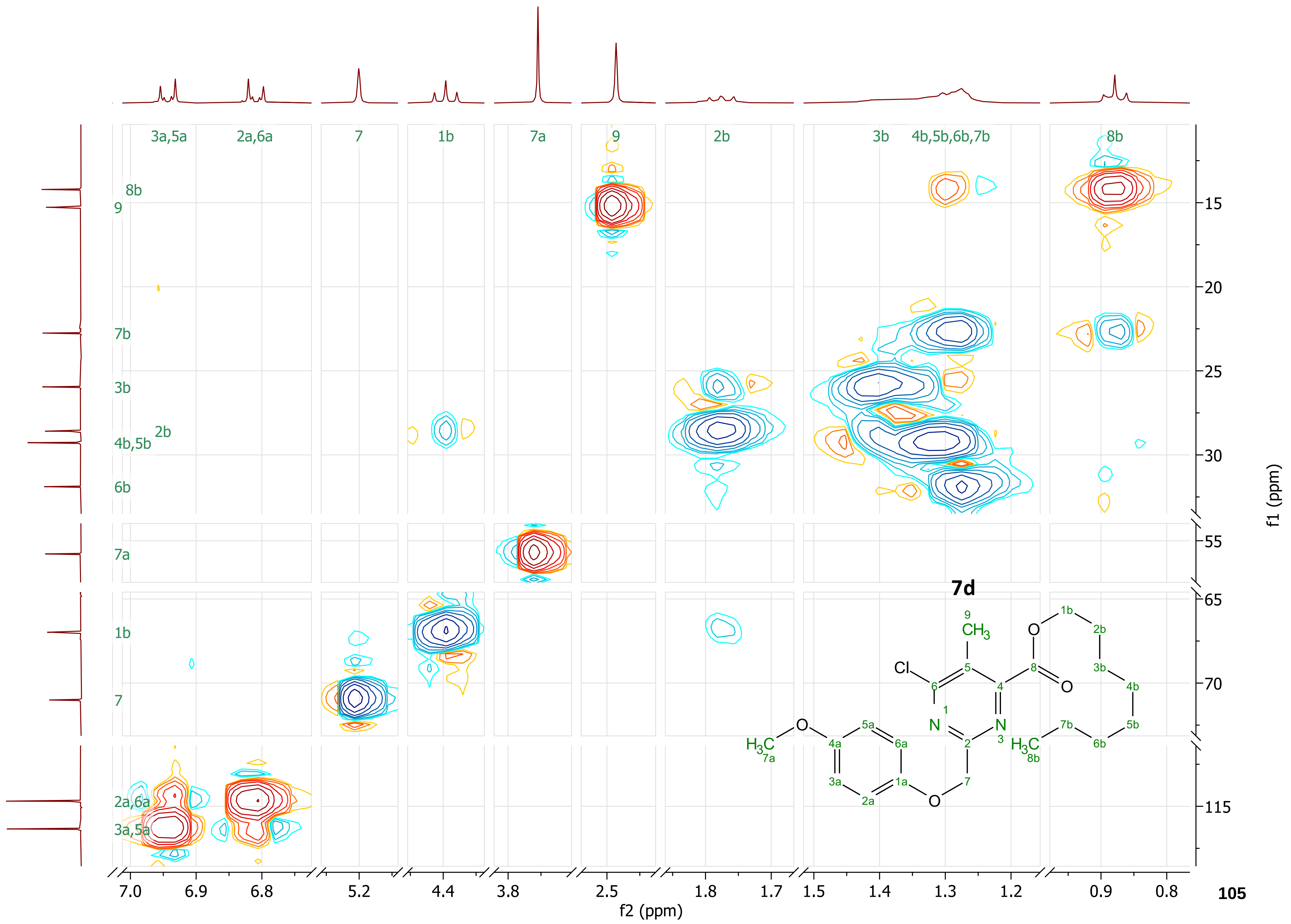
3a,5a
2a,6a
7
1b
7a
9
2b
3b
4b,5b,6b,7b
8b
9
7b
3b
2b
4b,5b
6b
7a
1b
7
2a,6a
3a,5a
7d
f2 (ppm)
f1 (ppm)

13C HMBC

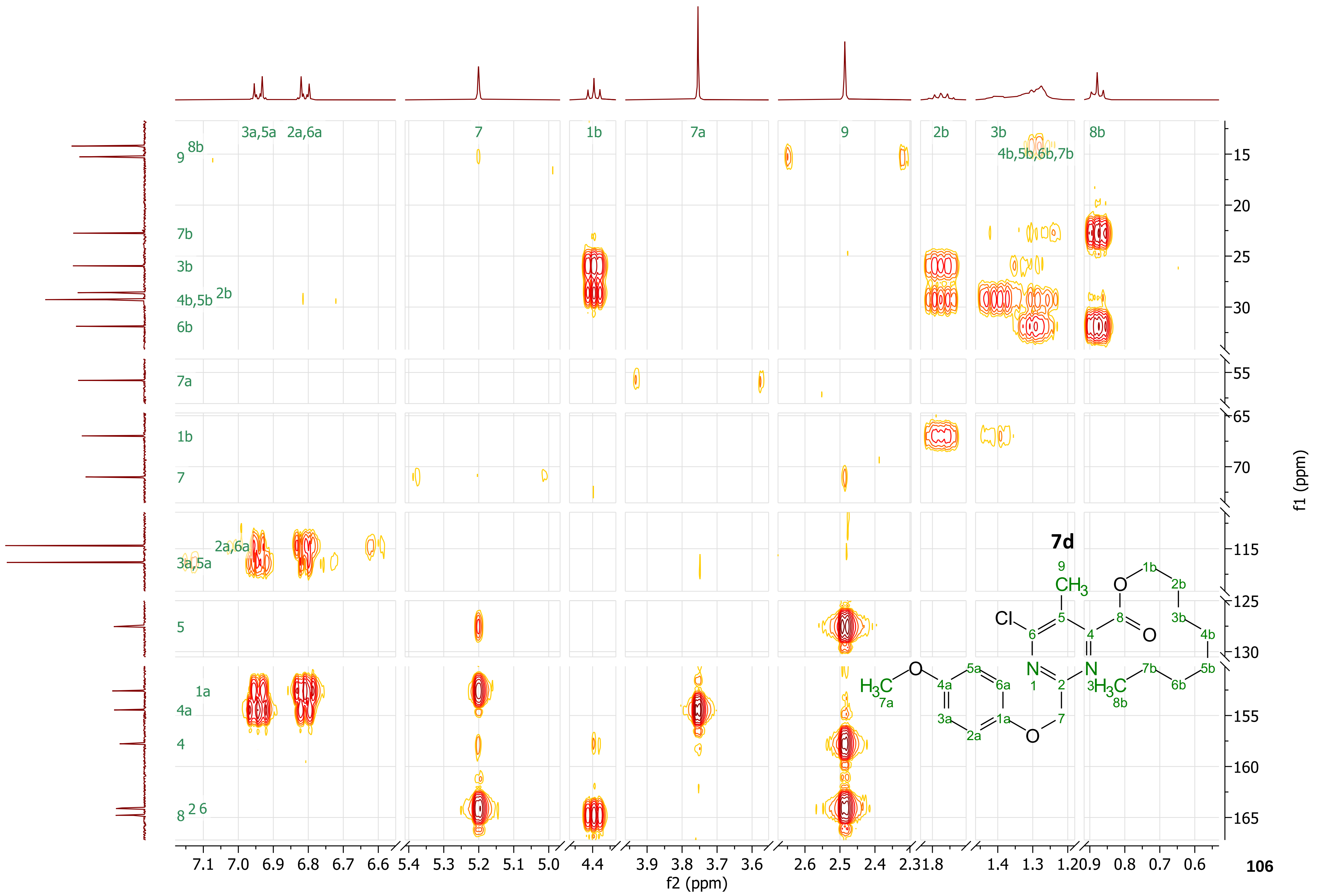
3a,5a
2a,6a
7
1b
7a
9
2b
3b
8b
4b,5b,6b,7b
9
8b
7b
3b
4b,5b
2b
6b
7a
1b
7
2a,6a
3a,5a
5
1a
4a
4
8
2
6
7d
CH3
O
O
Cl
N
N
O
H3C
O
H3C
f2 (ppm)
f1 (ppm)

15N HMBC

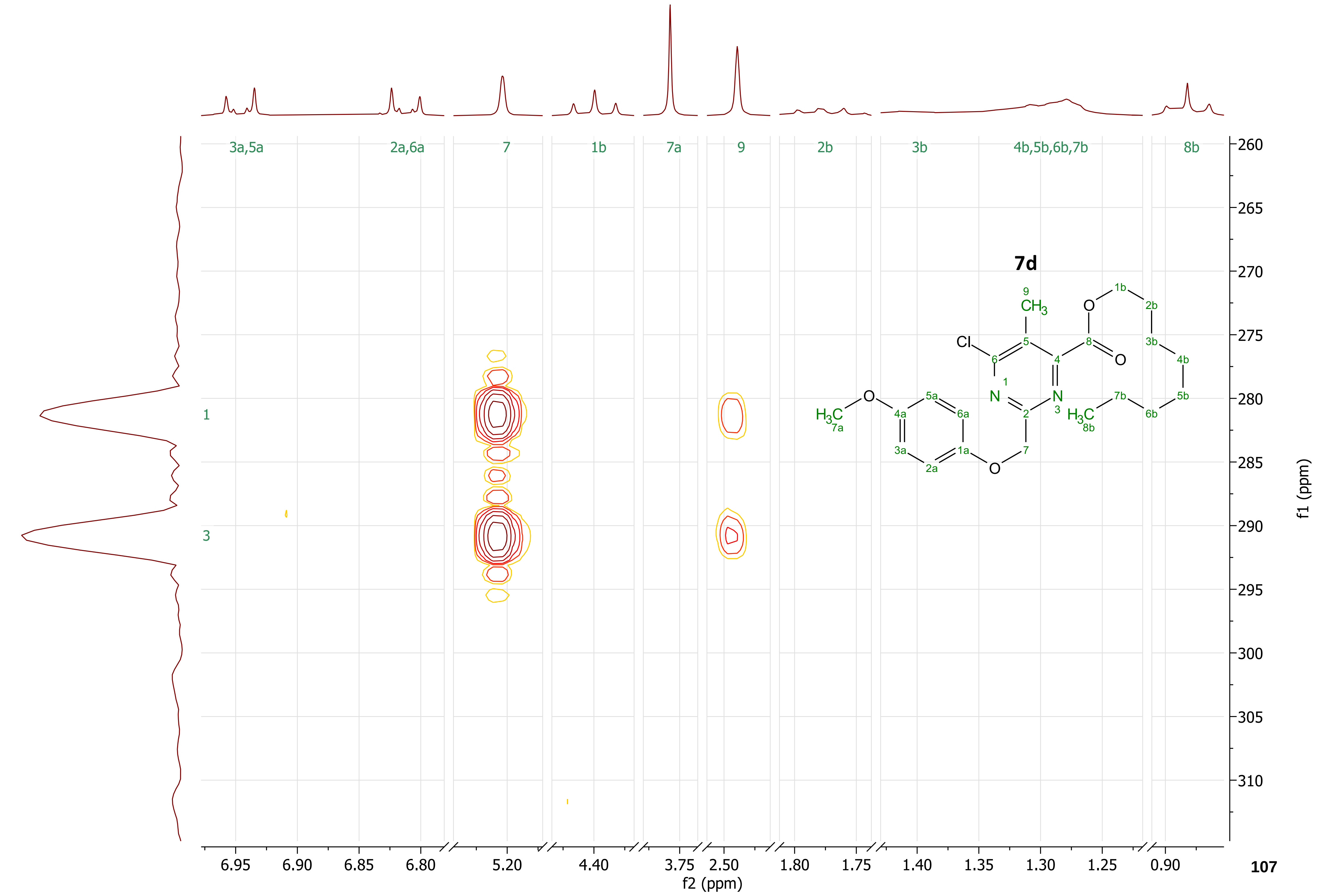
3a,5a
2a,6a
7
1b
7a
9
2b
3b
4b,5b,6b,7b
8b
1
3
7d
f1 (ppm)
f2 (ppm)
6.95
6.90
6.85
6.80
5.20
4.40
3.75
2.50
1.80
1.75
1.40
1.35
1.30
1.25
0.90
260
265
270
275
280
285
290
295
300
305
310

15N HMBC - f1 internal projection ^{15}N NMR (41 MHz, $CDCl_3$) δ 290.75, 281.23.

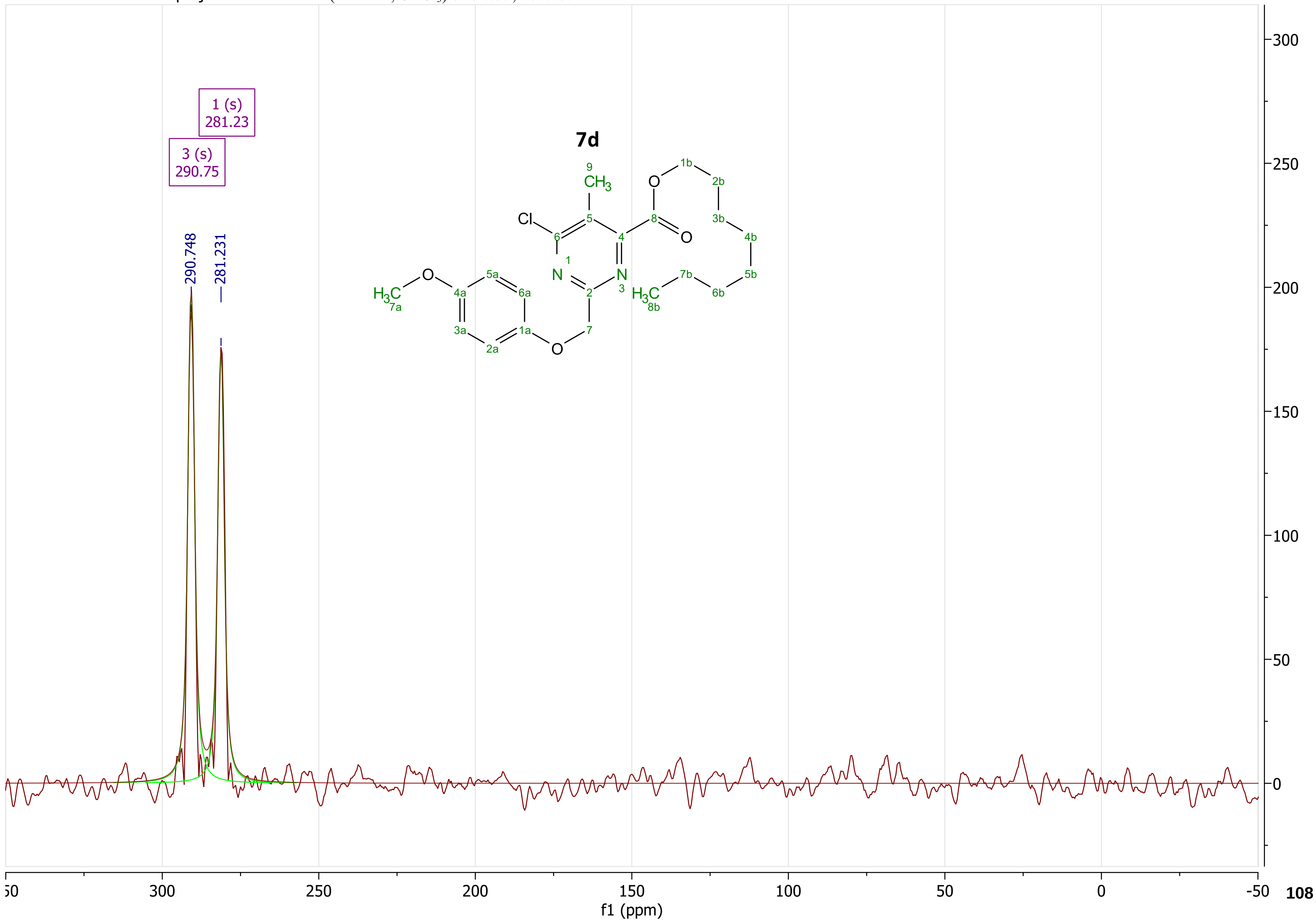

1H ^{1}H NMR (400 MHz, $CDCl_3$) δ 6.99 – 6.90 (m, 2H), 6.85 – 6.77 (m, 2H), 5.20 (s, 2H), 4.40 (t, *J* = 6.8 Hz, 2H), 3.76 (s, 3H), 2.49 (s, 3H), 1.83 – 1.72 (m, 2H), 1.46 – 1.36 (m, 2H), 1.35 – 1.24 (m, 10H), 0.88 (app t, *J* = 6.9 Hz, 3H).

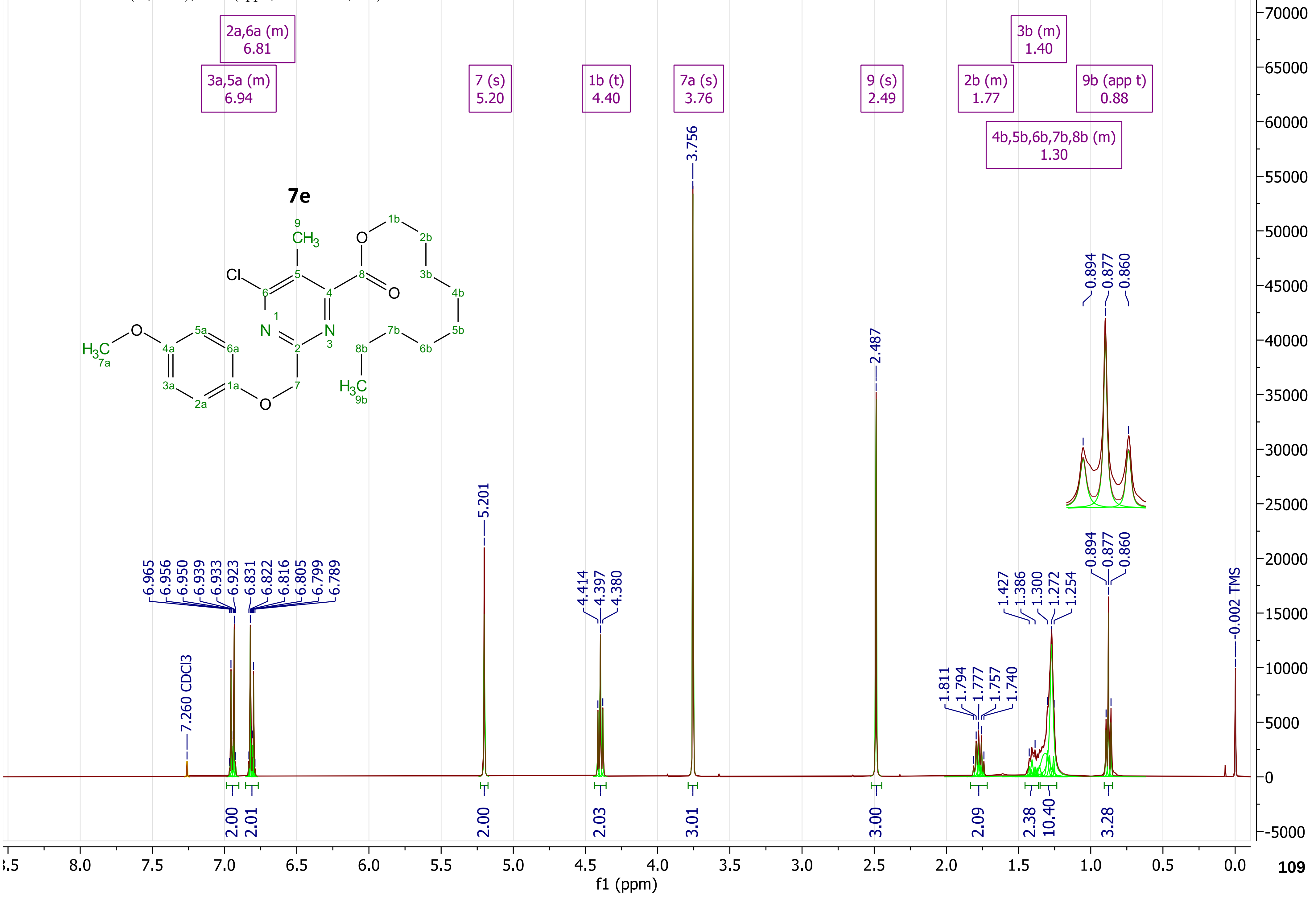

13C ^{13}C NMR (101 MHz, $CDCl_3$) δ 164.8, 164.1, 164.1, 157.8, 154.5, 152.6, 127.5, 116.4 (sym, 2C), 114.7 (sym, 2C), 71.0, 67.0, 55.8, 32.0, 29.6, 29.4, 29.3, 28.6, 26.0, 22.8, 15.3, 14.2.

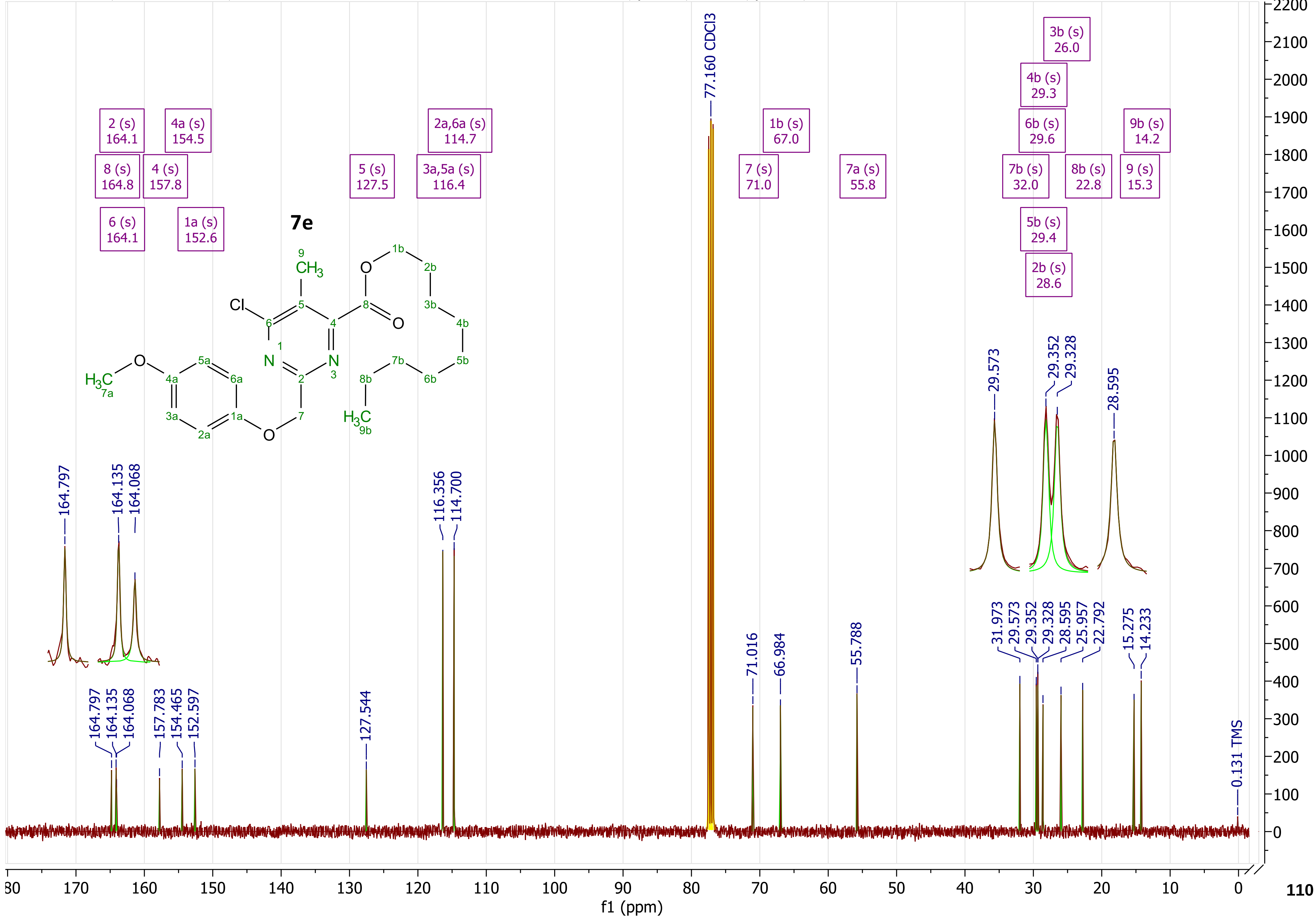

13C HSQC

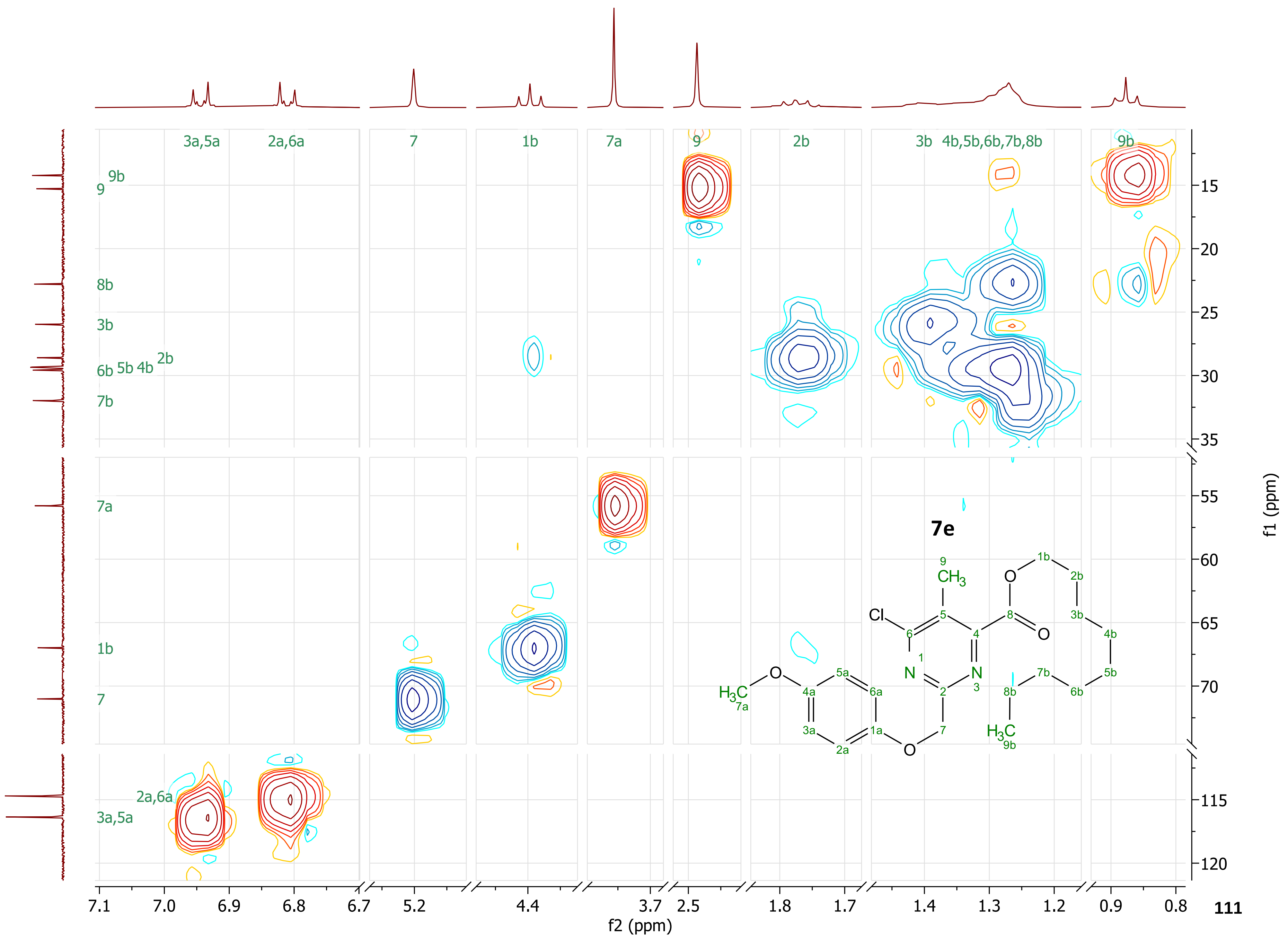

13C HMBC

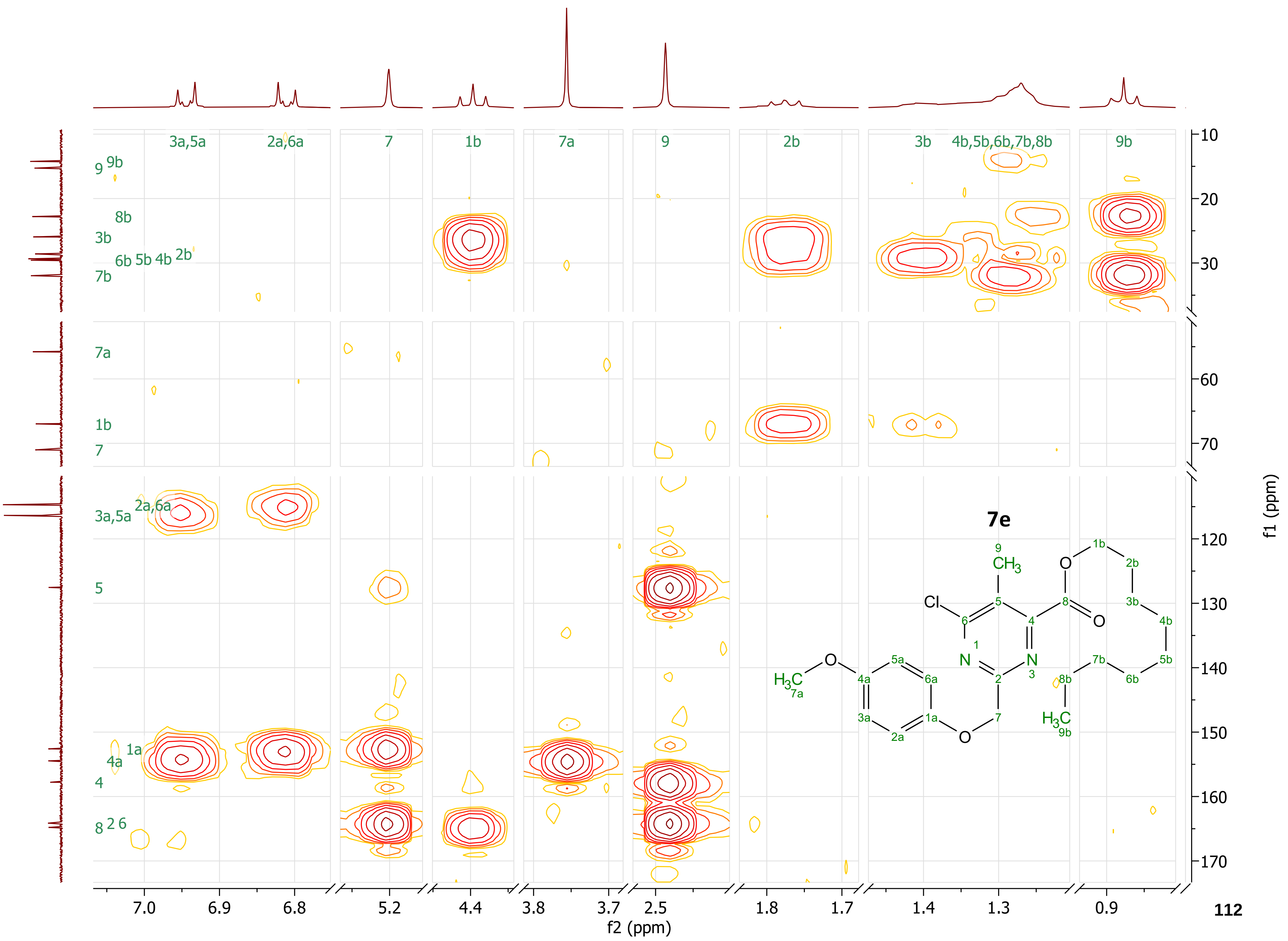

15N HMBC

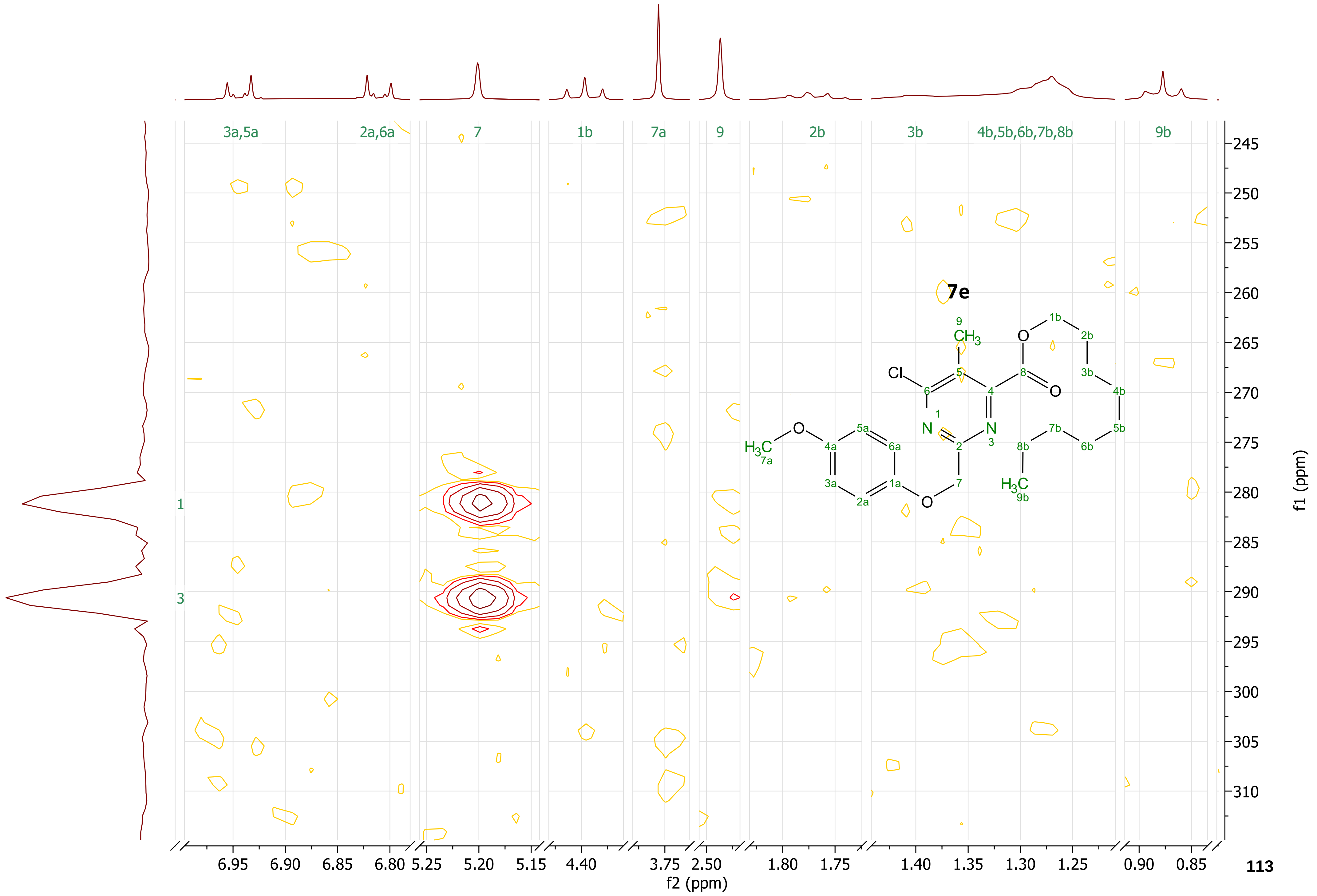
3a,5a
2a,6a
7
1b
7a
9
2b
3b
4b,5b,6b,7b,8b
9b
7e
1
3
6.95
6.90
6.85
6.80
5.25
5.20
5.15
4.40
3.75
2.50
1.80
1.75
1.40
1.35
1.30
1.25
0.90
0.85
f2 (ppm)
f1 (ppm)
245
250
255
260
265
270
275
280
285
290
295
300
305
310

15N HMBC - f1 internal projection ^{15}N NMR (41 MHz, $CDCl_3$) δ 290.62, 281.14.

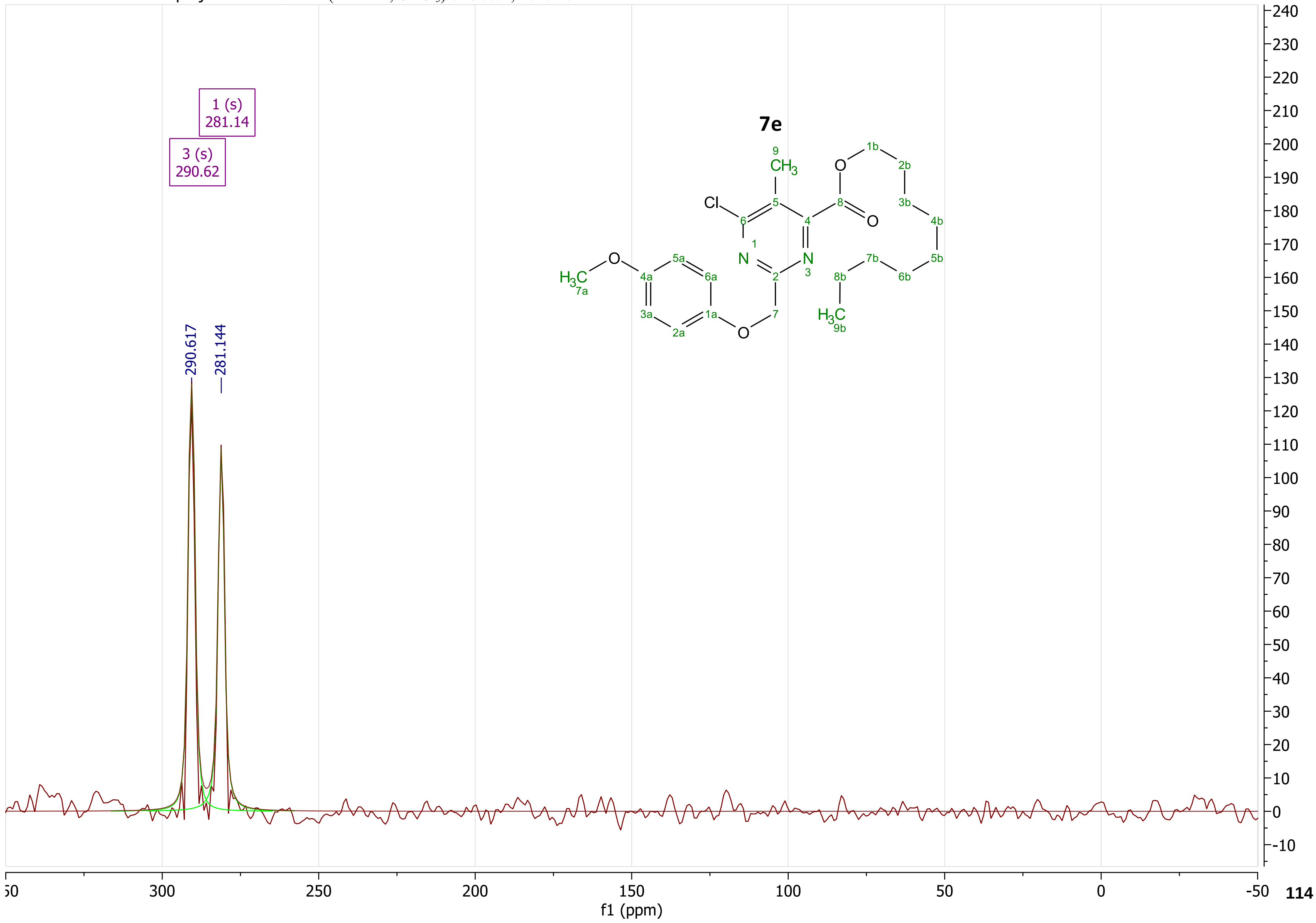

1H ^{1}H NMR (400 MHz, $CDCl_3$) δ 7.71 (br s, 1H), 7.67 – 7.61 (m, 1H), 7.65 – 7.59 (m, 1H), 7.57 – 7.48 (m, 1H), 6.98 – 6.87 (m, 2H), 6.84 – 6.76 (m, 2H), 5.47 (s, 2H), 5.21 (app d, J = 0.6 Hz, 2H), 3.75 (s, 3H), 2.47 (app t, J = 0.6 Hz, 3H).

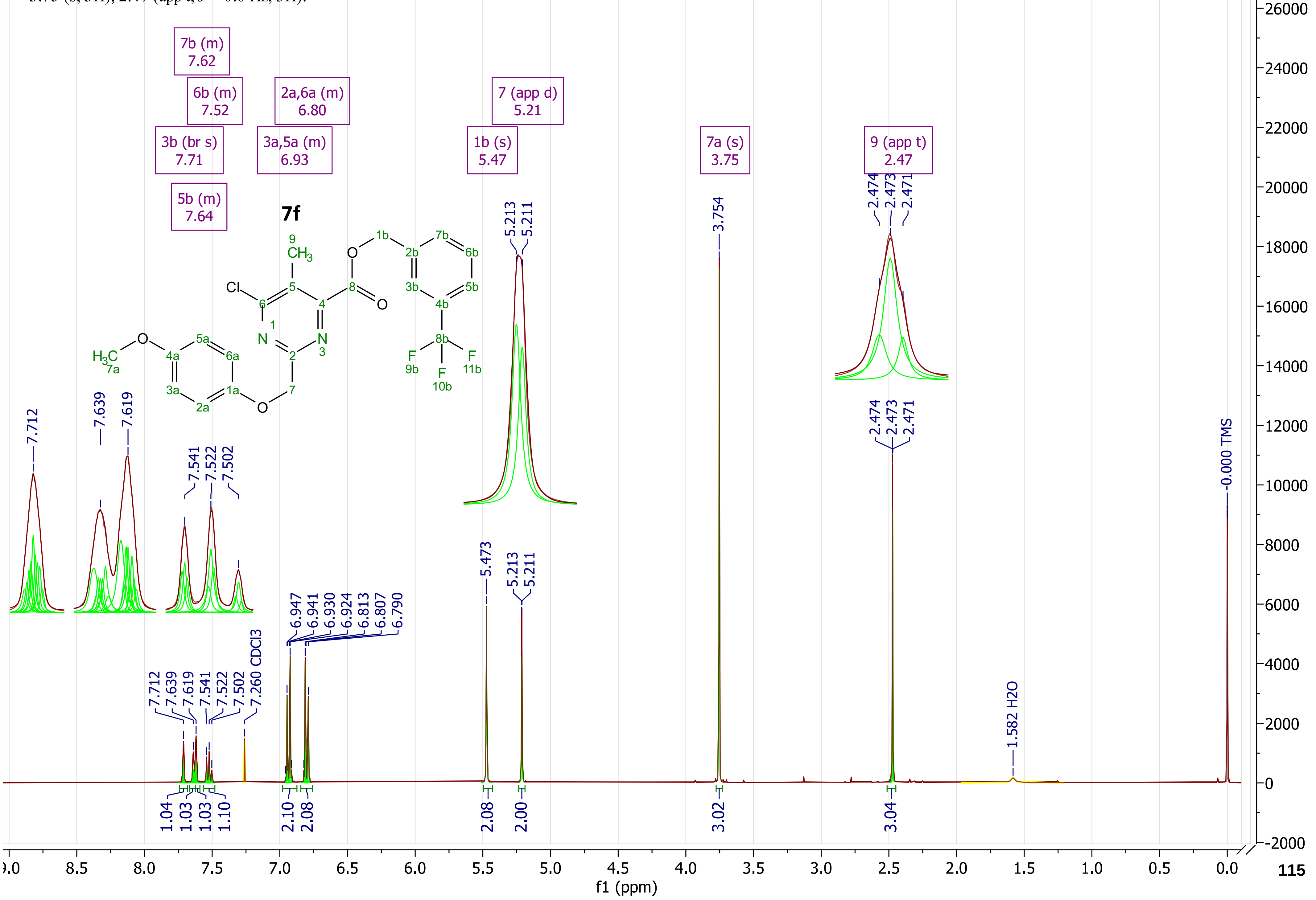

GHG103.10.fid — ^{13}C NMR (101 MHz, $CDCl_3$) δ 164.32, 164.30, 164.2, 156.7, 154.5, 152.5, 135.9, 131.8 (app q, J = 1.1 Hz), 131.4 (q, J = 32.5 Hz), 129.5, 128.1, 125.7 (q, J = 3.8 Hz), 125.2 (q, J = 3.8 Hz), 124.0 (q, J = 272.3 Hz), 116.3 (sym, 2C), 114.7 (sym, 2C), 70.9, 67.2, 55.8, 15.2.

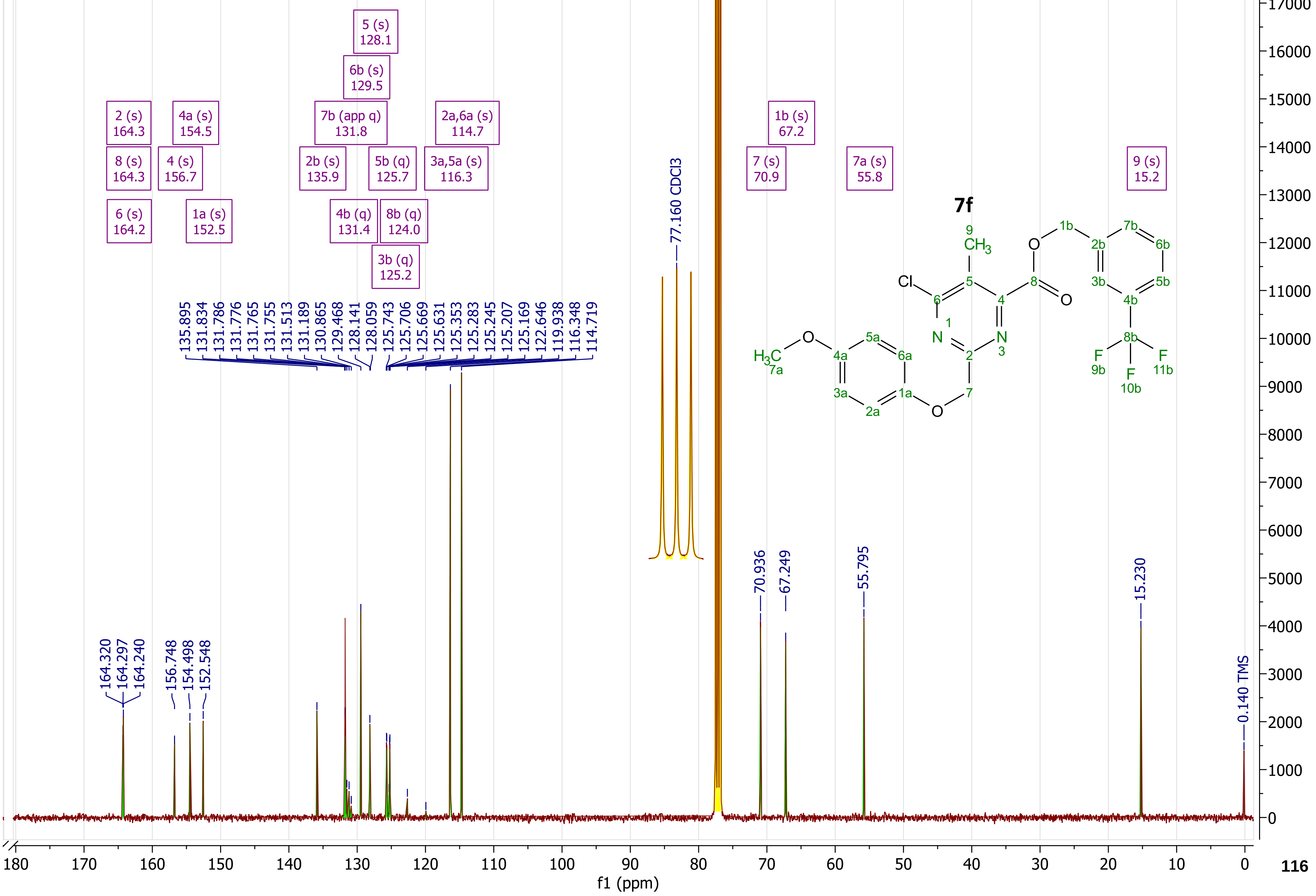

13C — 165–119 ppm

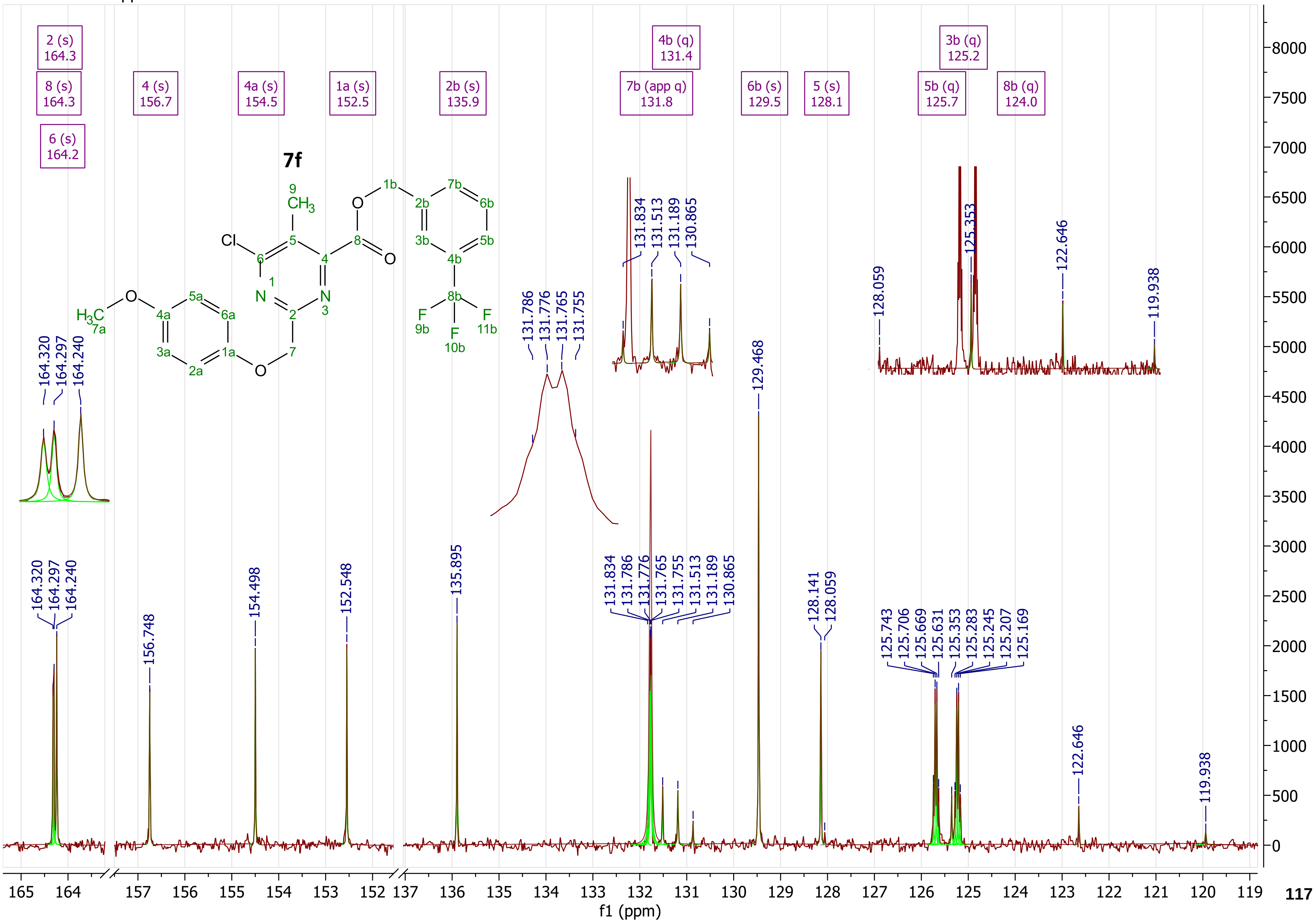

13C HSQC

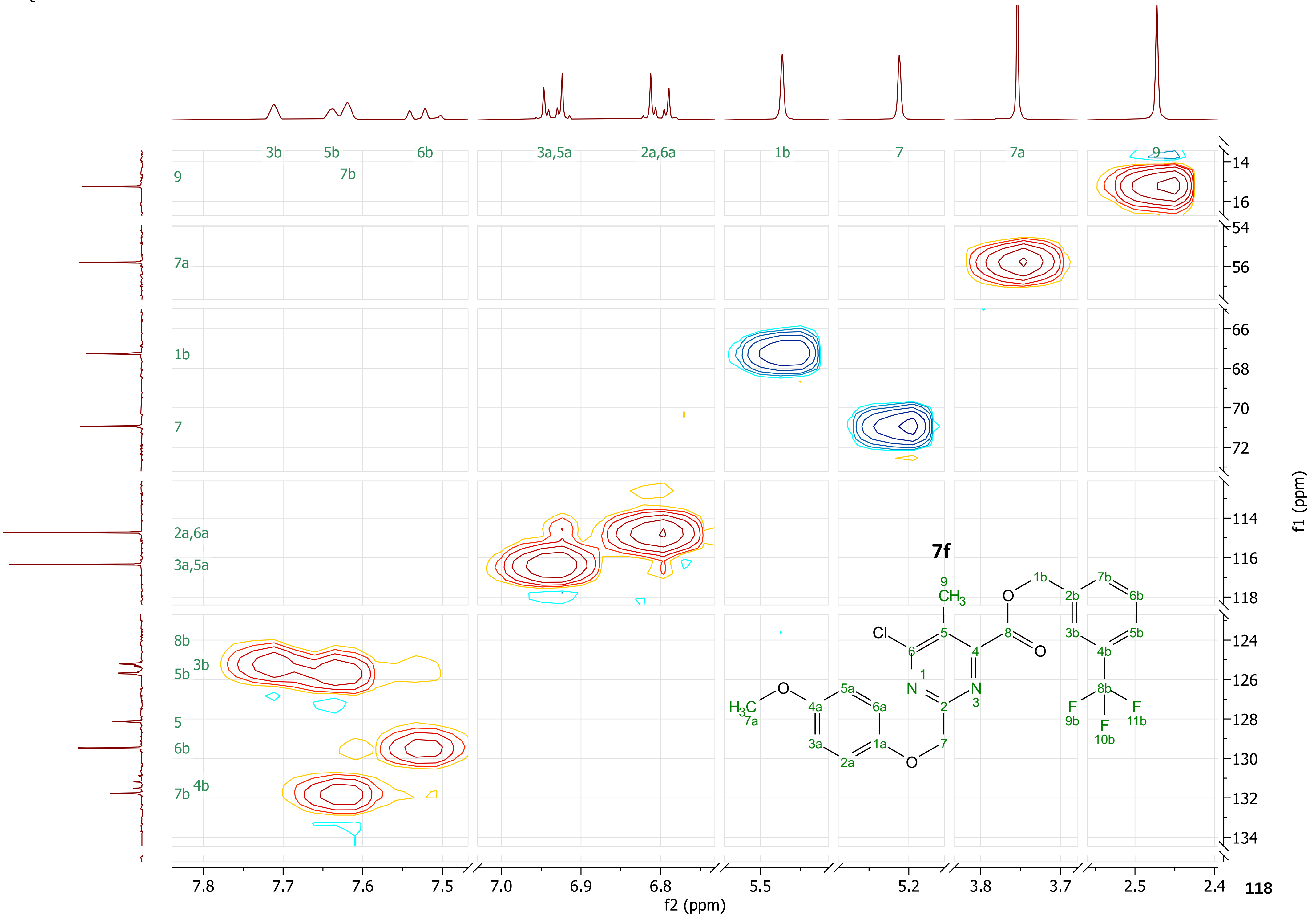
3b
5b
7b
6b
3a,5a
2a,6a
1b
7
7a
9
8b
5
6b
4b
7f
f2 (ppm)
f1 (ppm)
7.8
7.7
7.6
7.5
7.0
6.9
6.8
5.5
5.2
3.8
3.7
2.5
2.4
14
16
54
56
66
68
70
72
114
116
118
124
126
128
130
132
134

13C HMBC

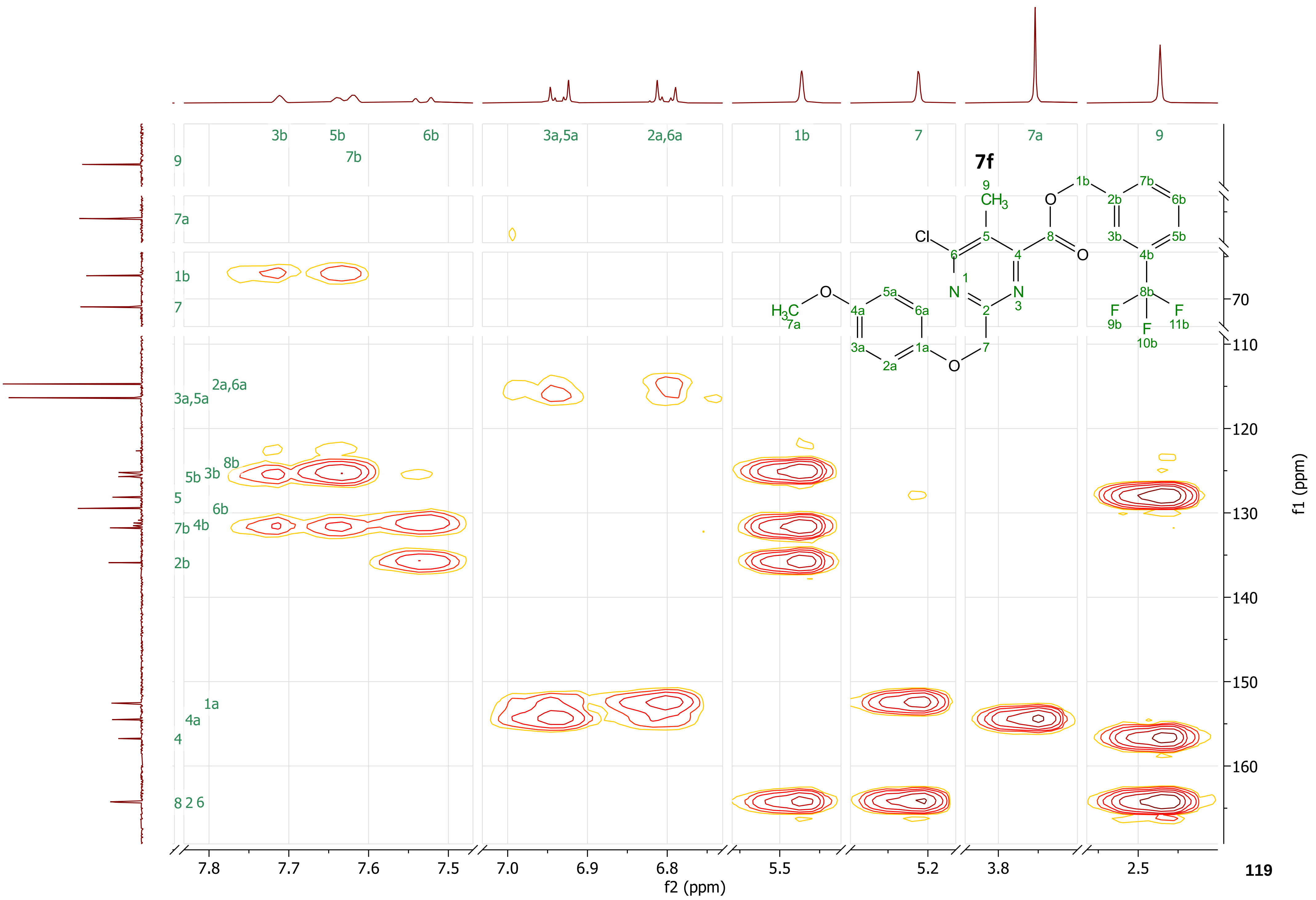

15N HMBC

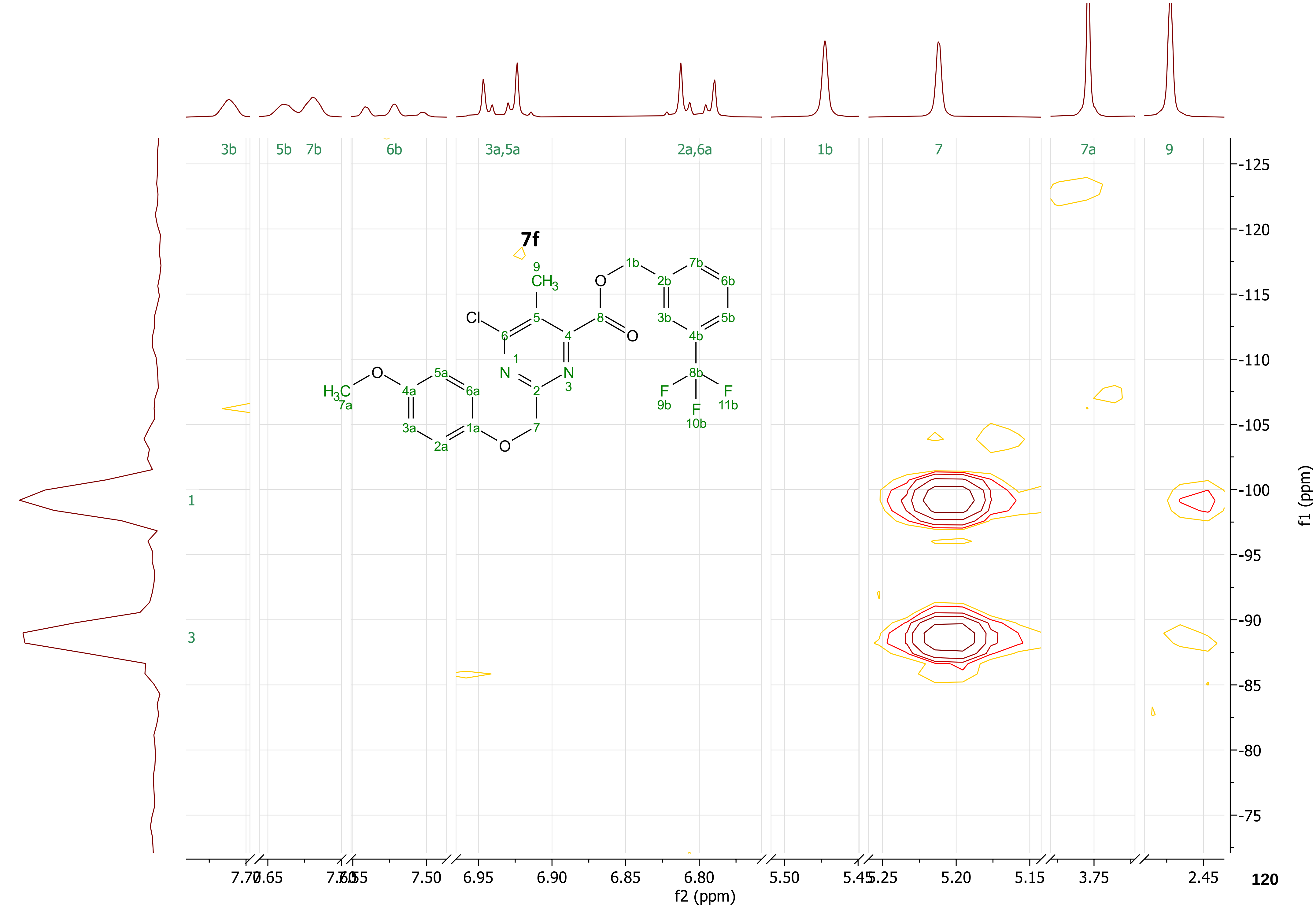

15N HMBC - f1 internal projection ^{15}N NMR (41 MHz, $CDCl_3$) δ -88.66, -99.21.

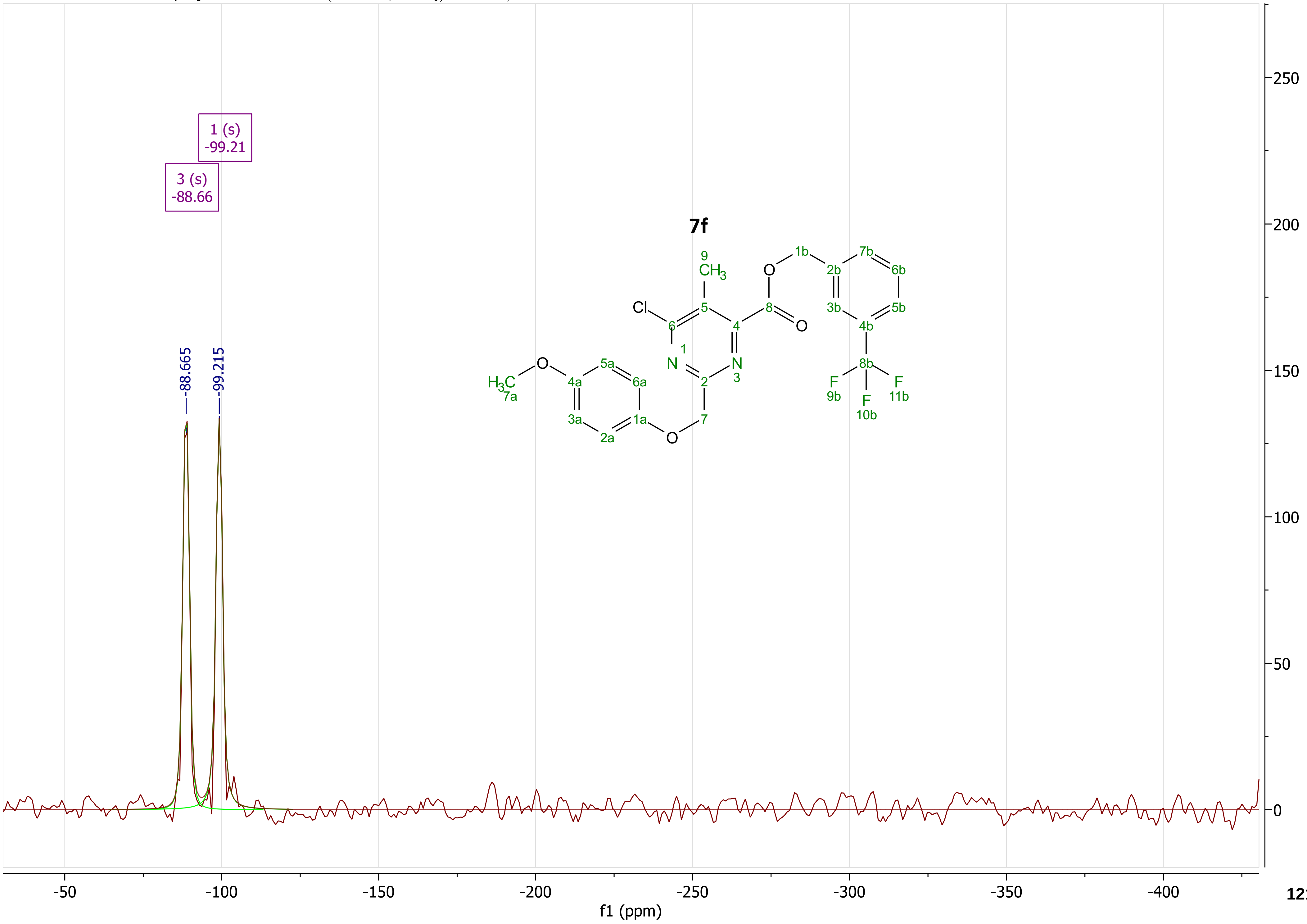

19F ^{19}F NMR (376 MHz, $CDCl_3$) δ -62.72.

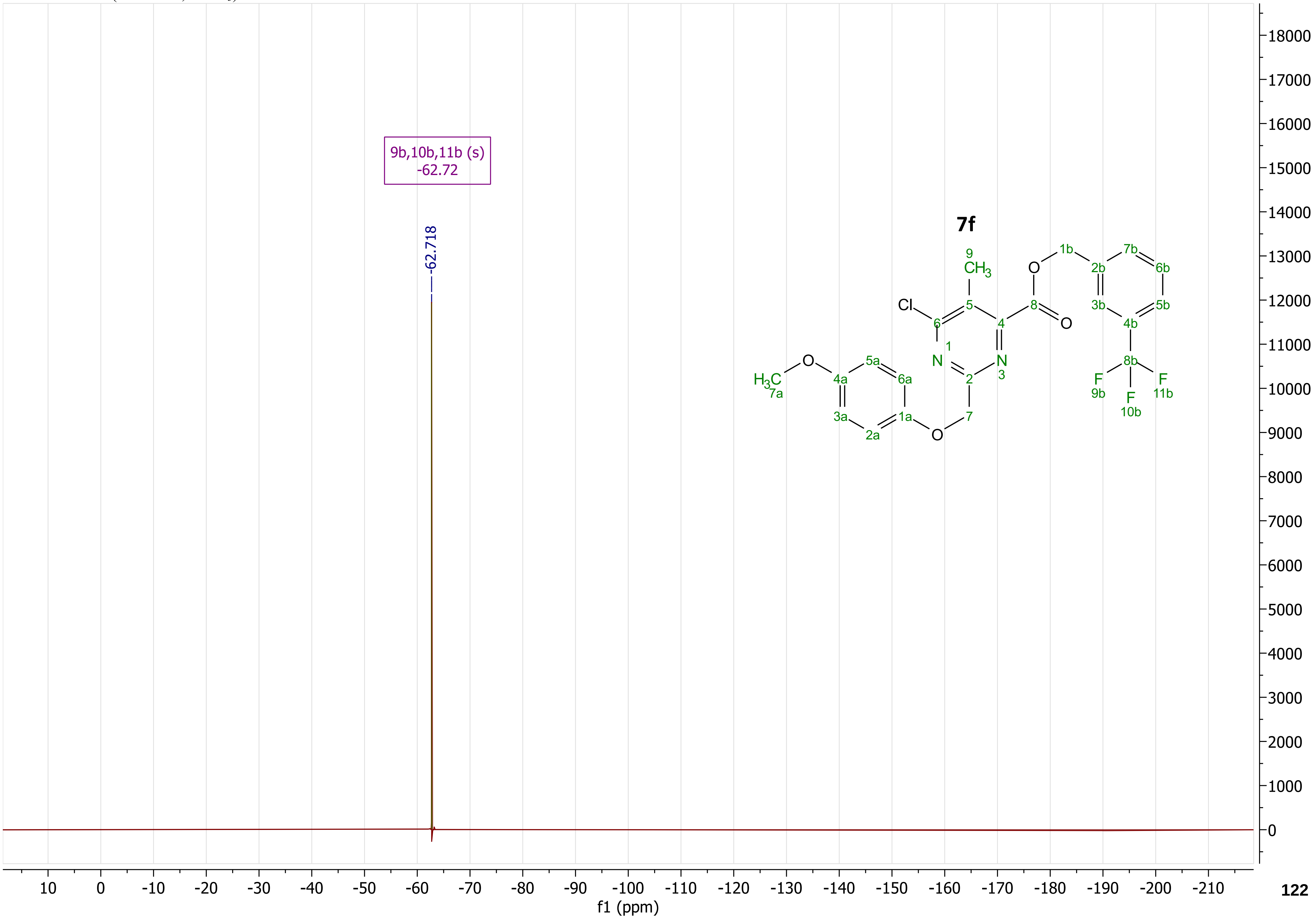

1H ^{1}H NMR (400 MHz, $CDCl_3$) δ 7.02 – 6.86 (m, 2H), 6.86 – 6.71 (m, 2H), 5.20 (s, 2H), 4.41 (t, J = 6.9 Hz, 2H), 3.75 (s, 3H), 2.42 (s, 3H), 1.79 (quint, J = 6.9 Hz, 2H), 1.46 – 1.37 (m, 2H), 1.37 – 1.23 (m, 8H), 0.88 (t, J = 6.9 Hz, 3H).

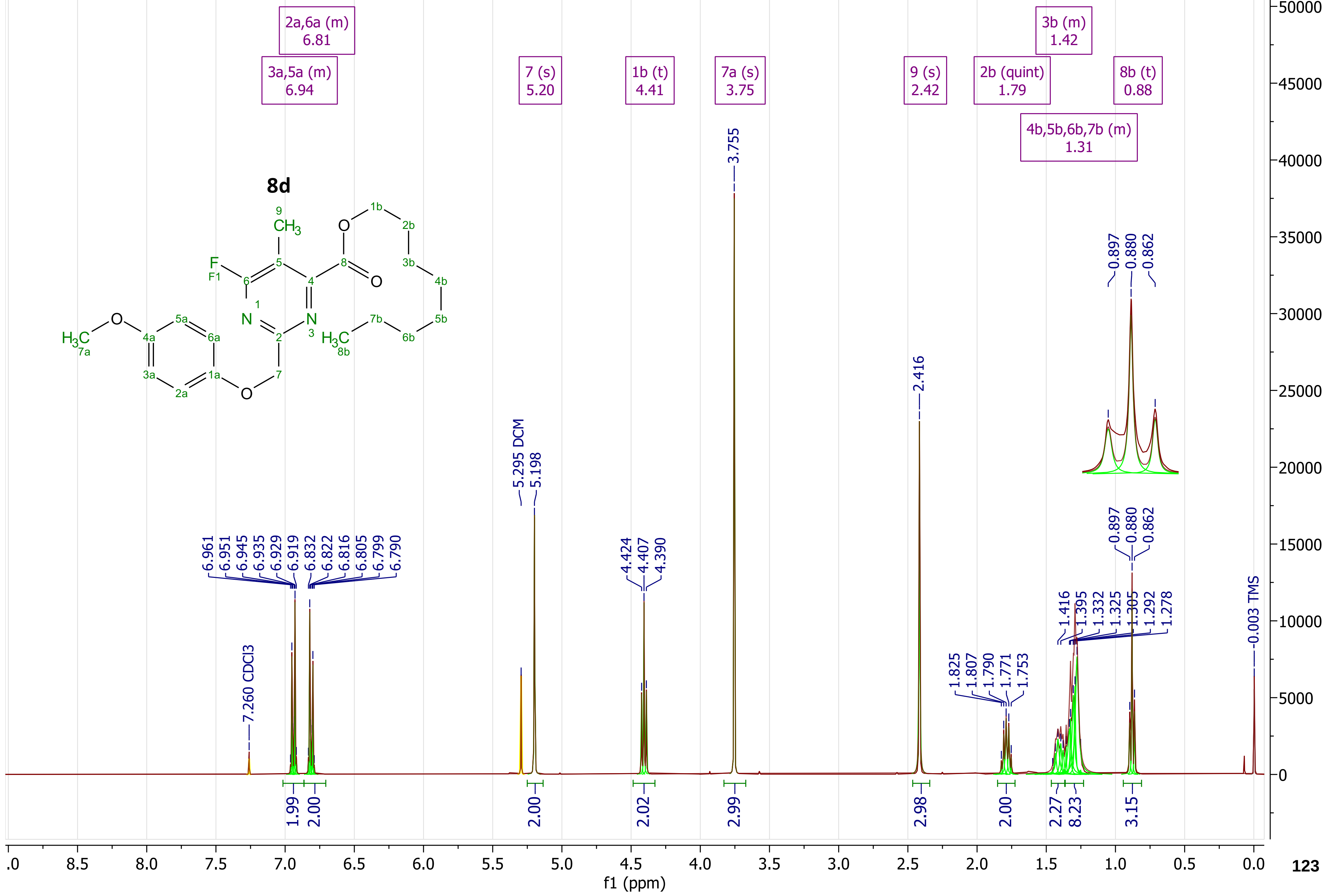

13C ^{13}C NMR (101 MHz, $CDCl_3$) δ 169.9 (d, J = 255.4 Hz), 164.6 (d, J = 13.5 Hz), 164.4 (d, J = 5.9 Hz), 159.4 (d, J = 5.8 Hz), 154.5, 152.5, 116.4 (d, J = 27.4 Hz), 116.3, 114.7, 70.8, 66.9, 55.8, 31.9, 29.28, 29.29, 28.6, 26.0, 22.8, 14.2, 10.5.

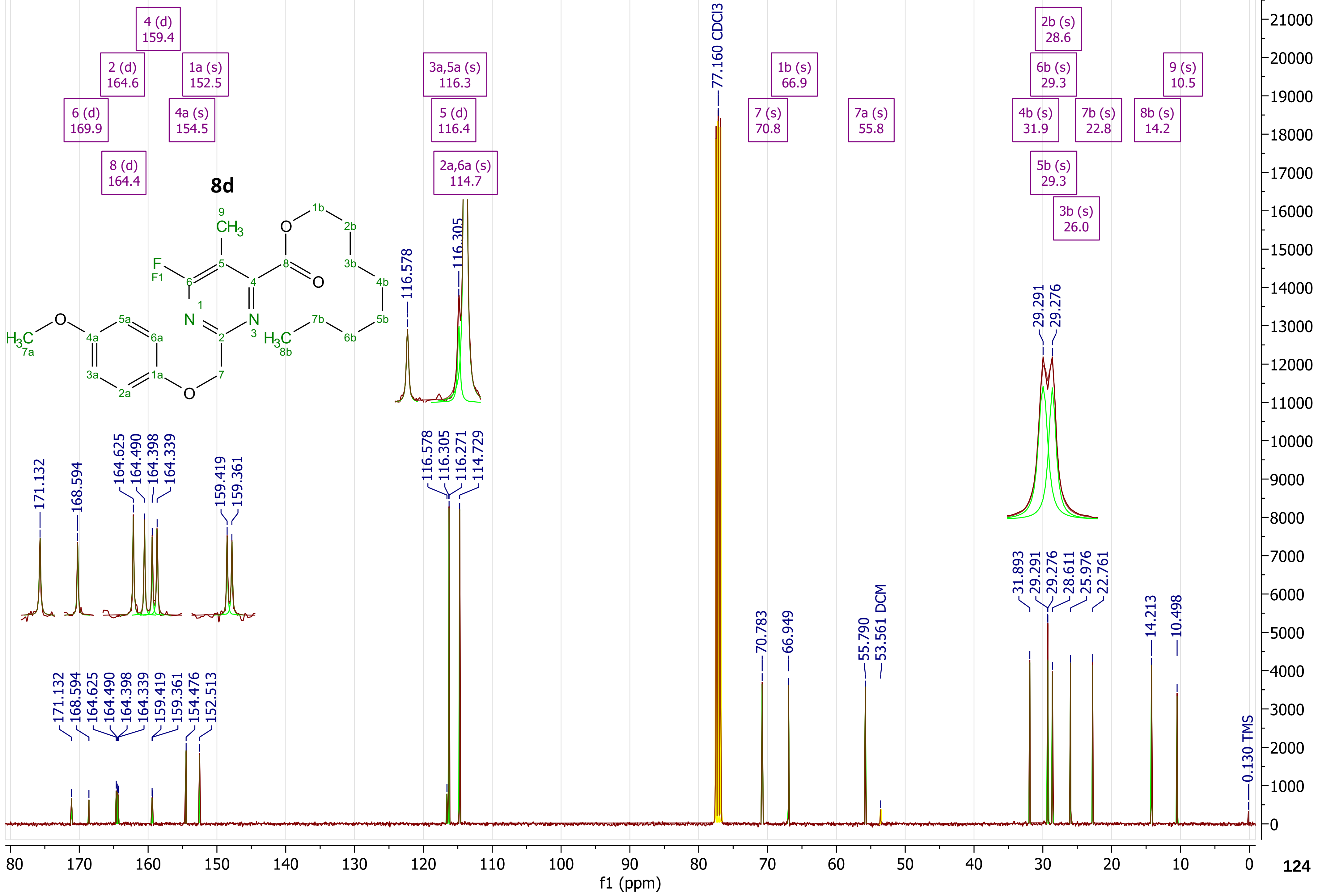

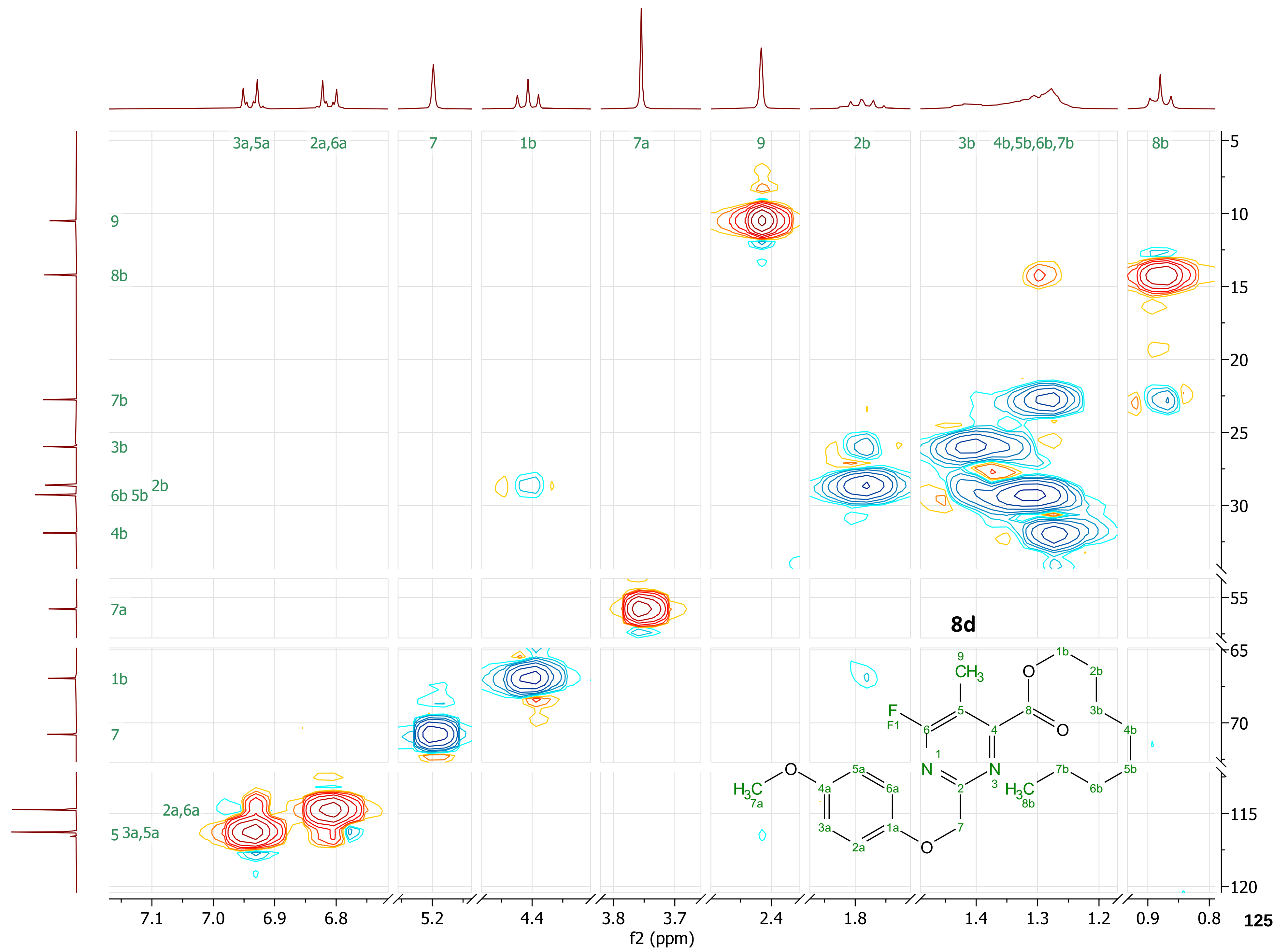
3a,5a
2a,6a
7
1b
7a
9
2b
3b
4b,5b,6b,7b
8b
8d
f2 (ppm)
f1 (ppm)

13C HMBC

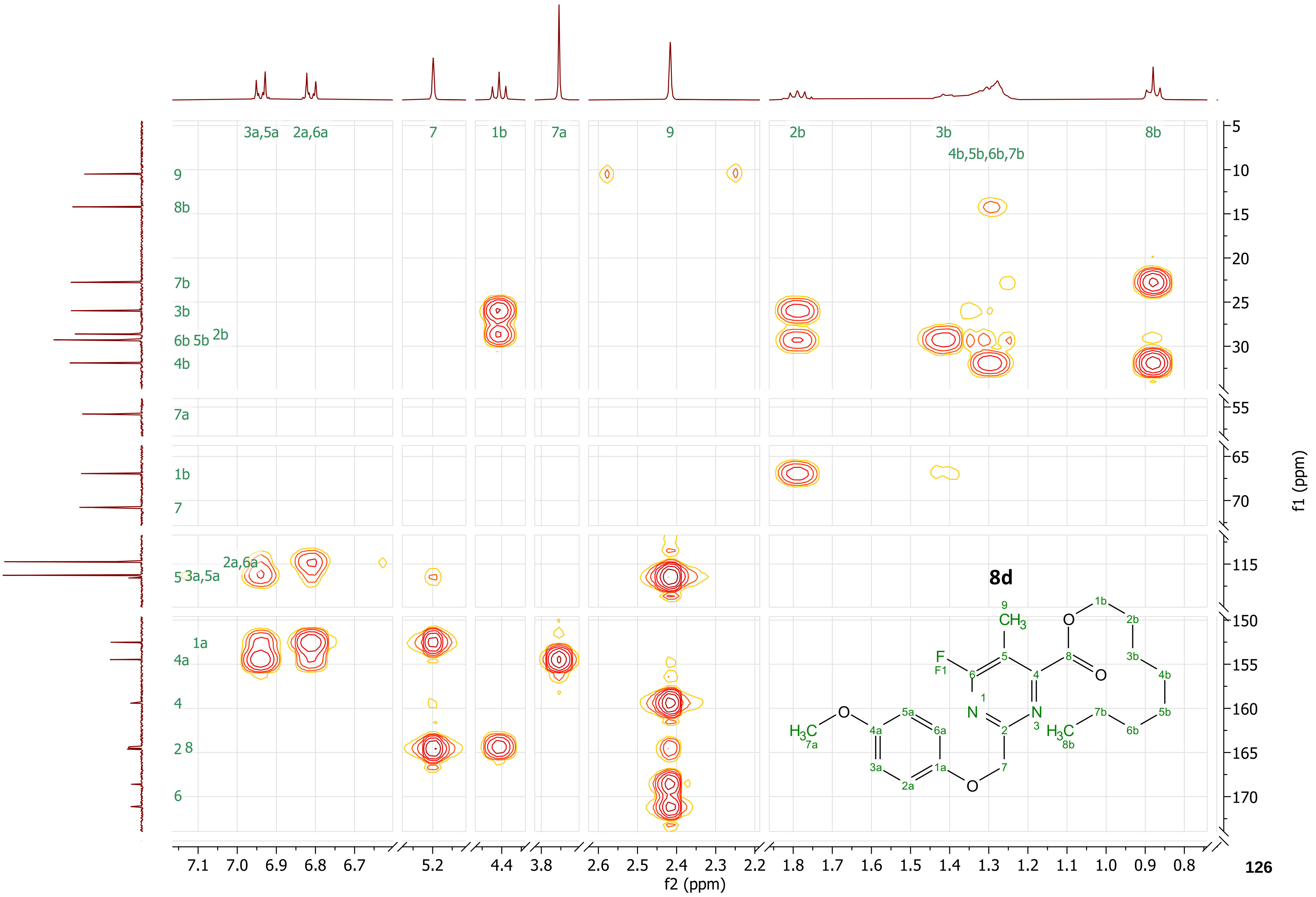
3a,5a
2a,6a
7
1b
7a
9
2b
3b
4b,5b,6b,7b
8b
9
8b
7b
3b
6b 5b 2b
4b
7a
1b
7
2a,6a
5 3a,5a
1a
4a
4
2 8
6
8d
f1 (ppm)
f2 (ppm)

15N HMBC

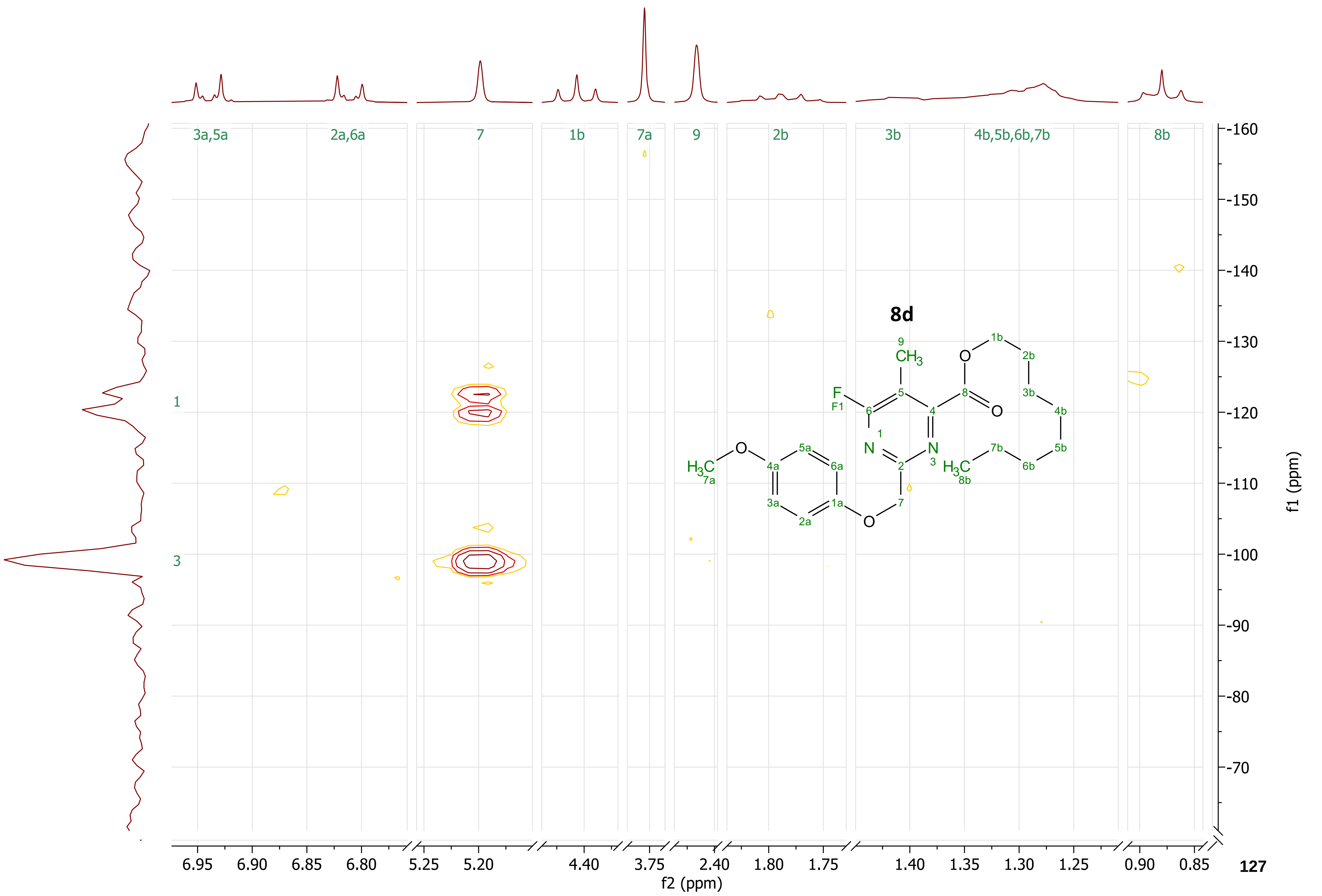

15N HMBC - f1 internal projection ^{15}N NMR (41 MHz, $CDCl_3$) δ -99.13, -121.57 (d, J = 98.5 Hz).

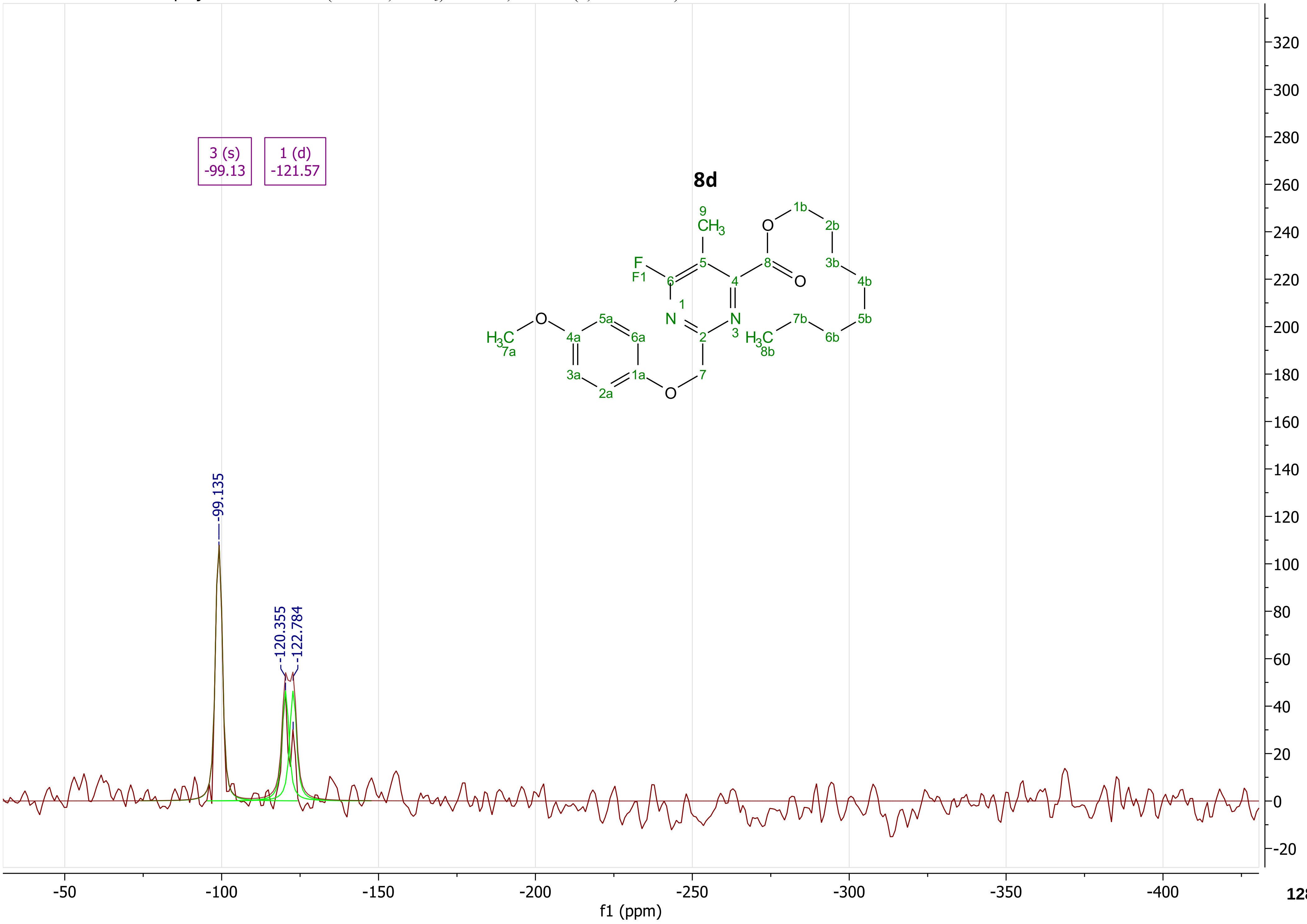

19F ^{19}F NMR (376 MHz, $CDCl_3$) δ -60.82.

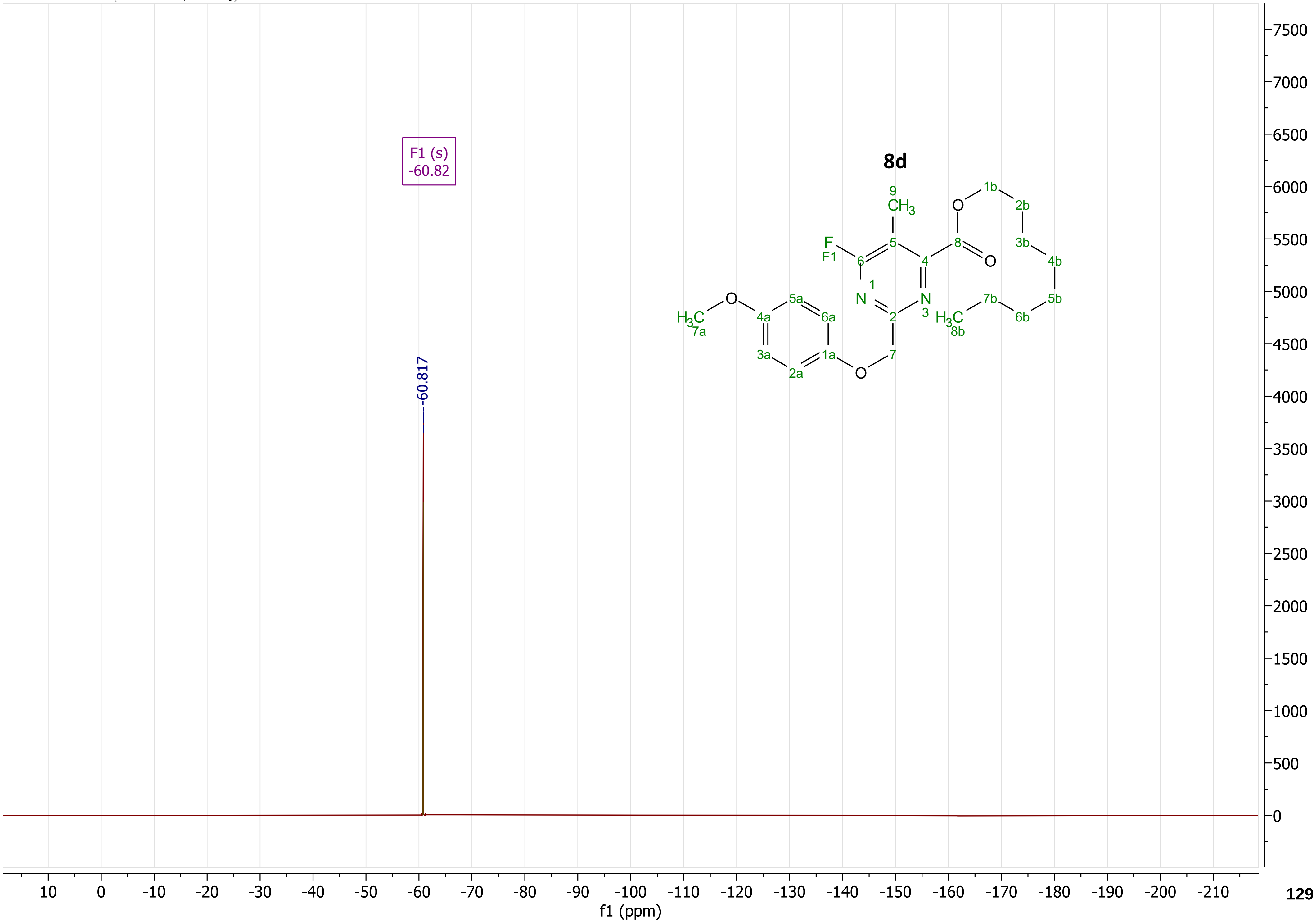

1H ^1H NMR (400 MHz, $CDCl_3$) δ 4.82 (br s, 3H), 4.46 (q, J = 7.2 Hz, 3H), 3.33 (br s, 1H), 2.49 (app t, J = 0.8 Hz, 4H), 1.41 (t, J = 7.2 Hz, 4H).

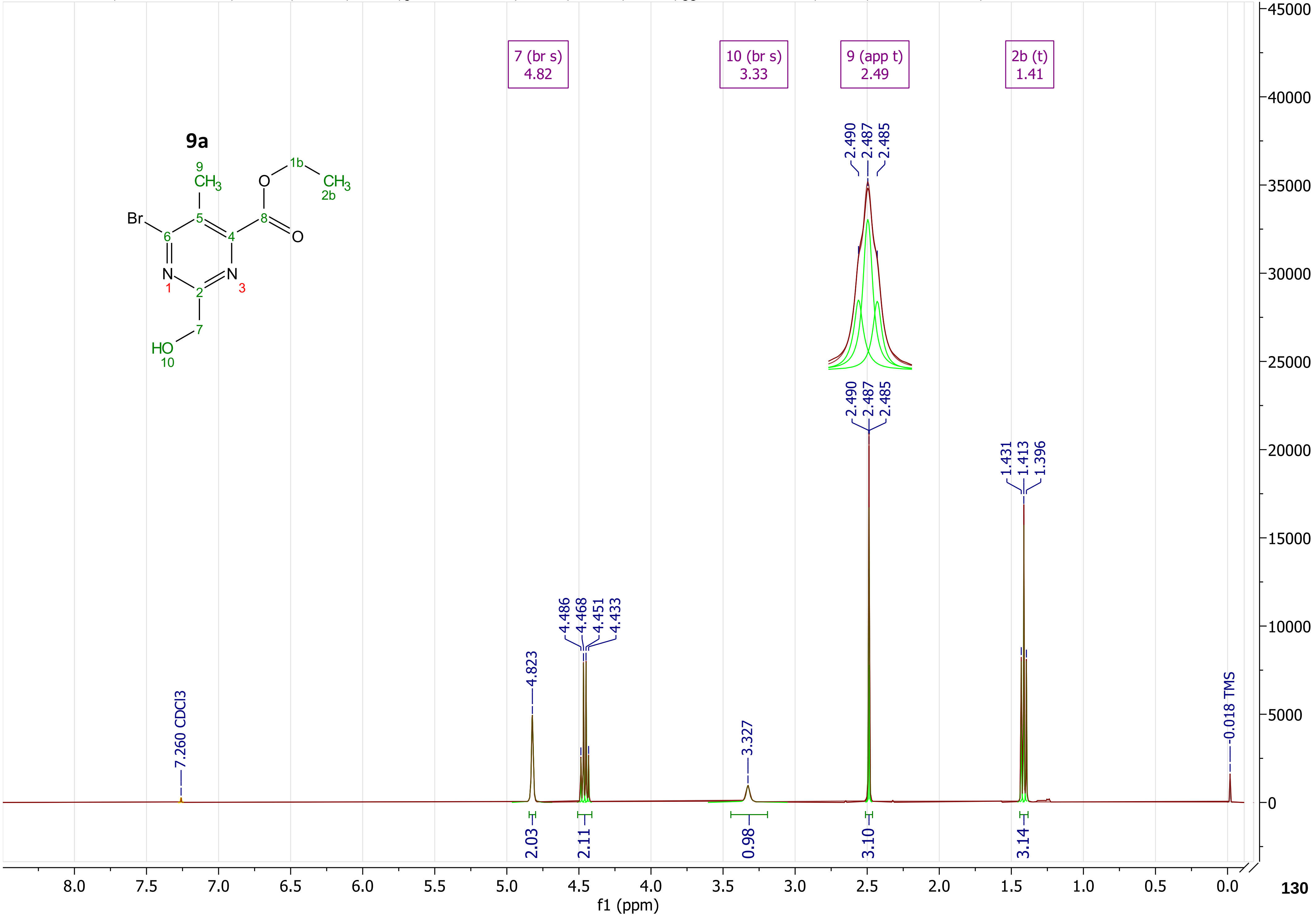

13C ^{13}C NMR (101 MHz, $CDCl_3$) δ 166.5, 164.5, 158.0, 156.4, 129.6, 64.1, 62.9, 17.7, 14.2.

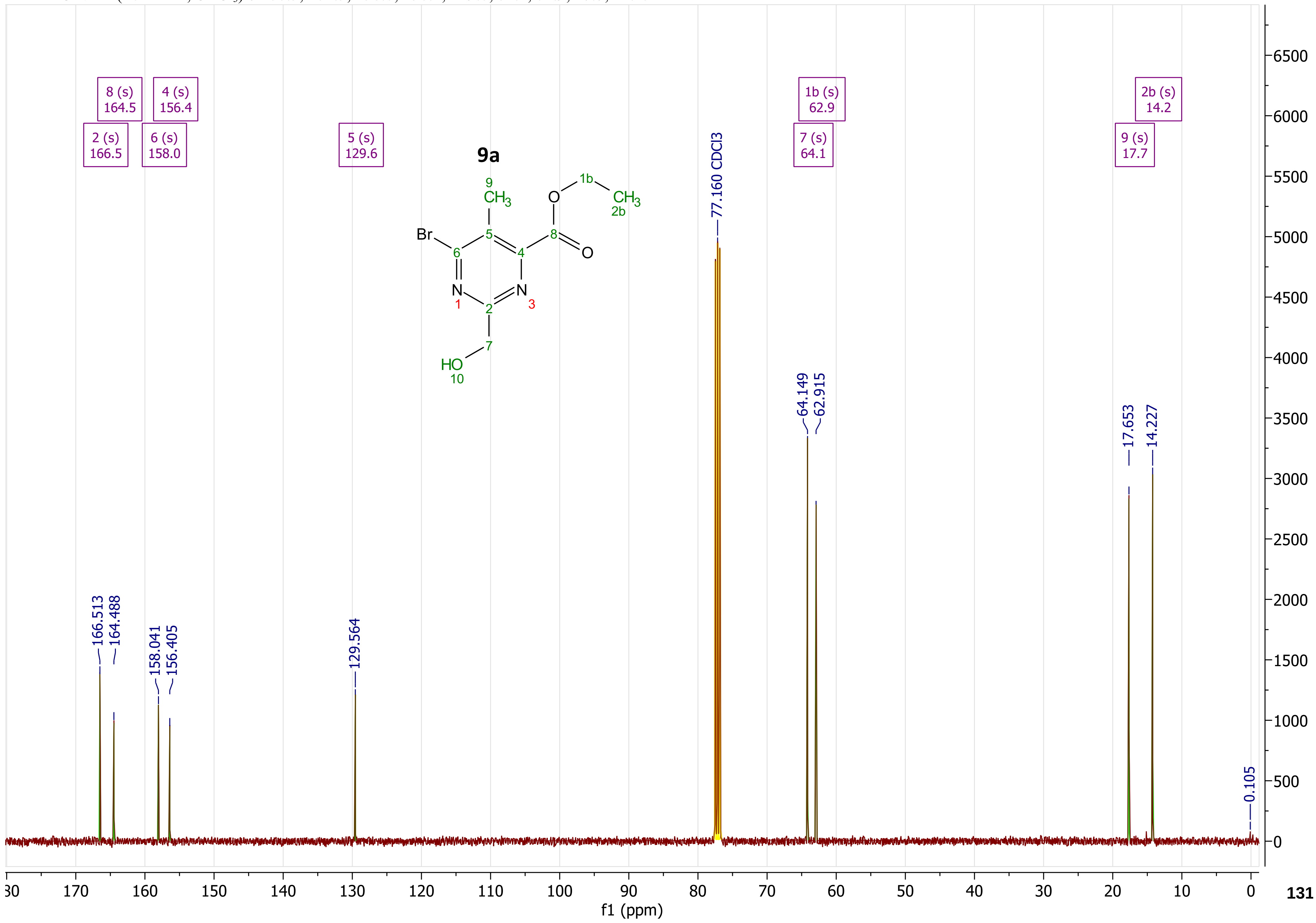

13C HSQC

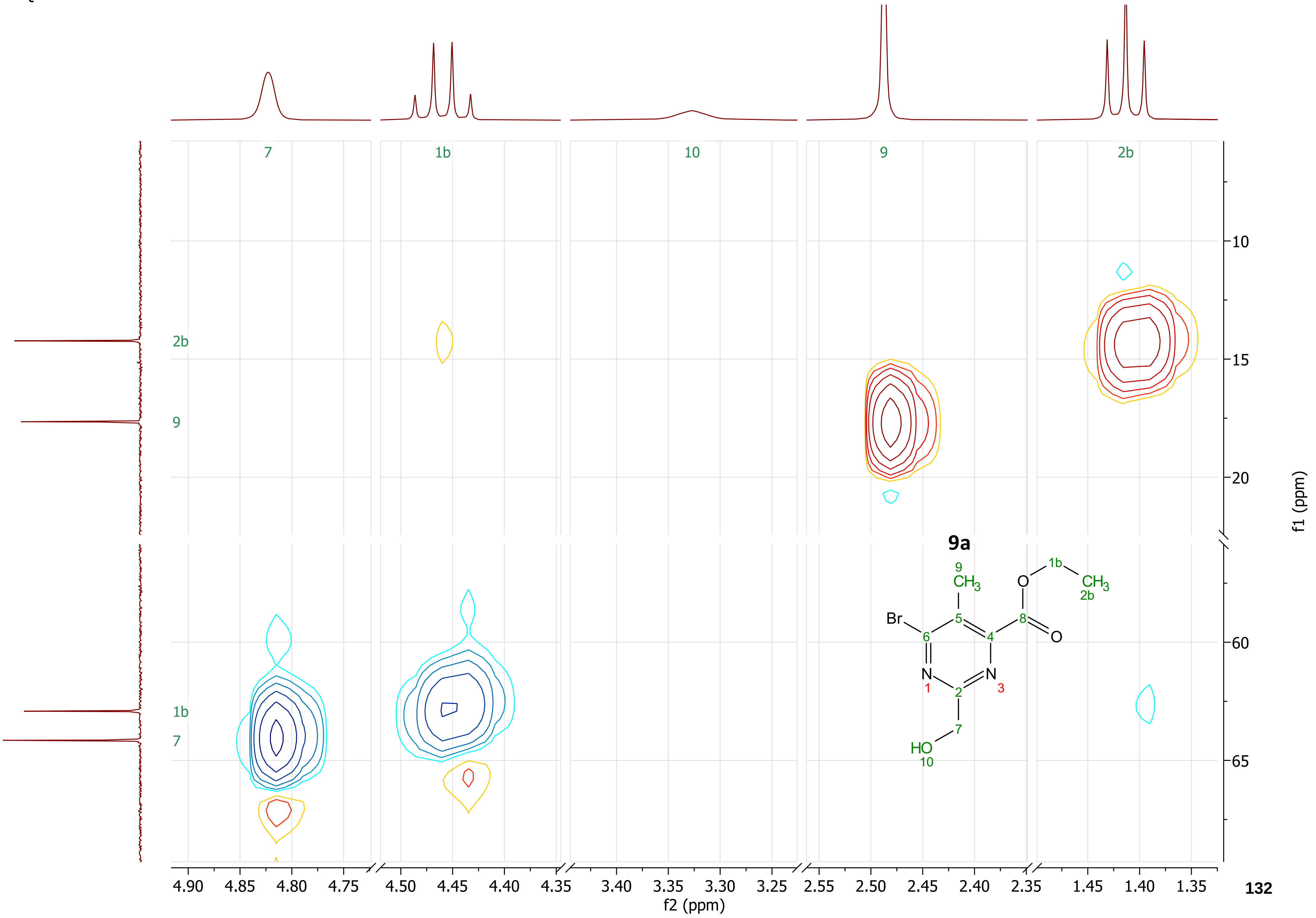

13C HMBC

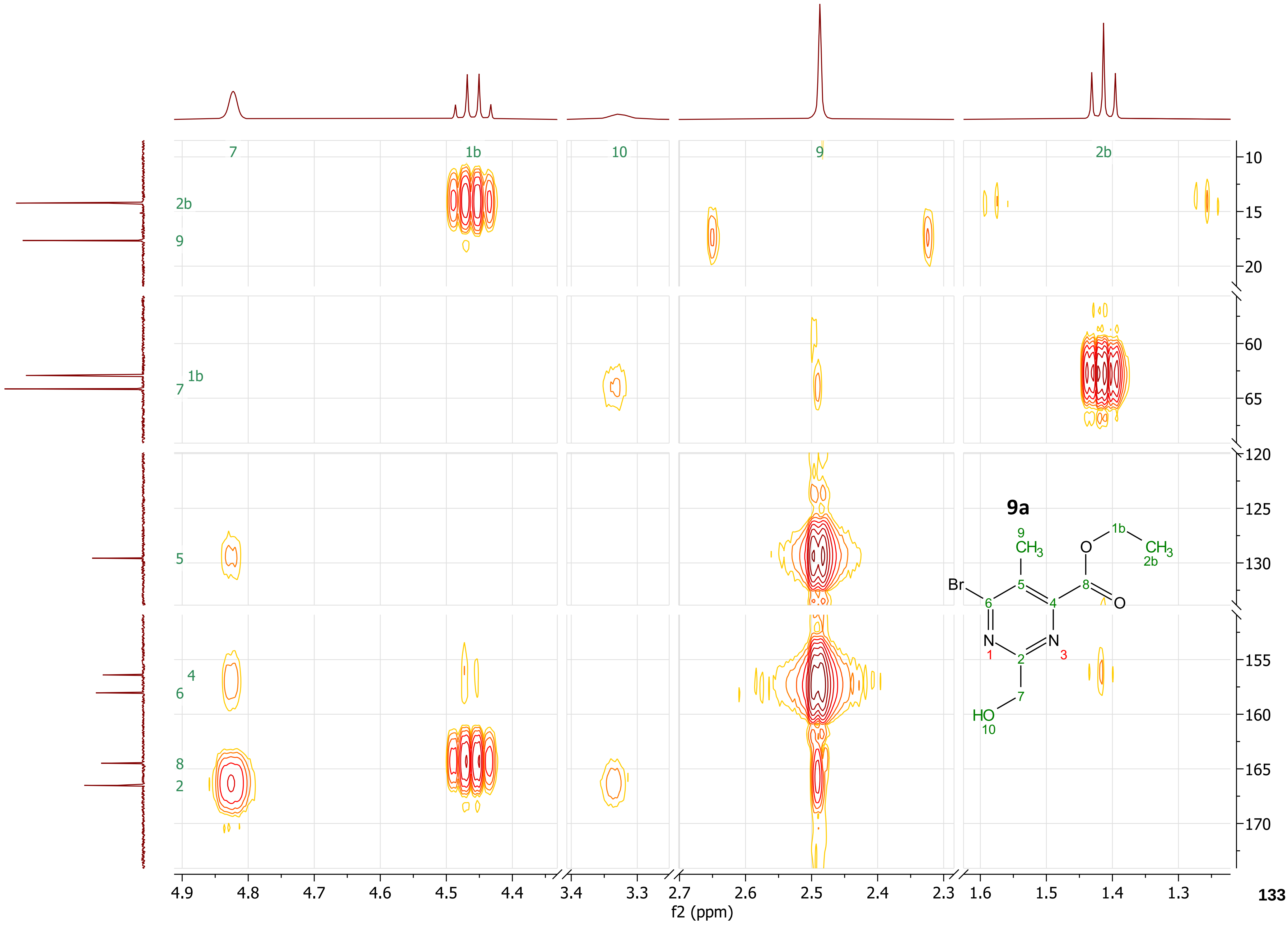

1H ^{1}H NMR (400 MHz, $CDCl_3$) δ 4.83 (d, *J* = 5.1 Hz, 2H), 4.41 (t, *J* = 6.8, 1.3 Hz, 2H), 3.27 (t, *J* = 5.5 Hz, 1H), 2.49 (app t, *J* = 0.8 Hz, 3H), 1.82 – 1.70 (m, 2H), 1.52 – 1.38 (m, 2H), 0.97 (t, *J* = 7.4 Hz, 3H).

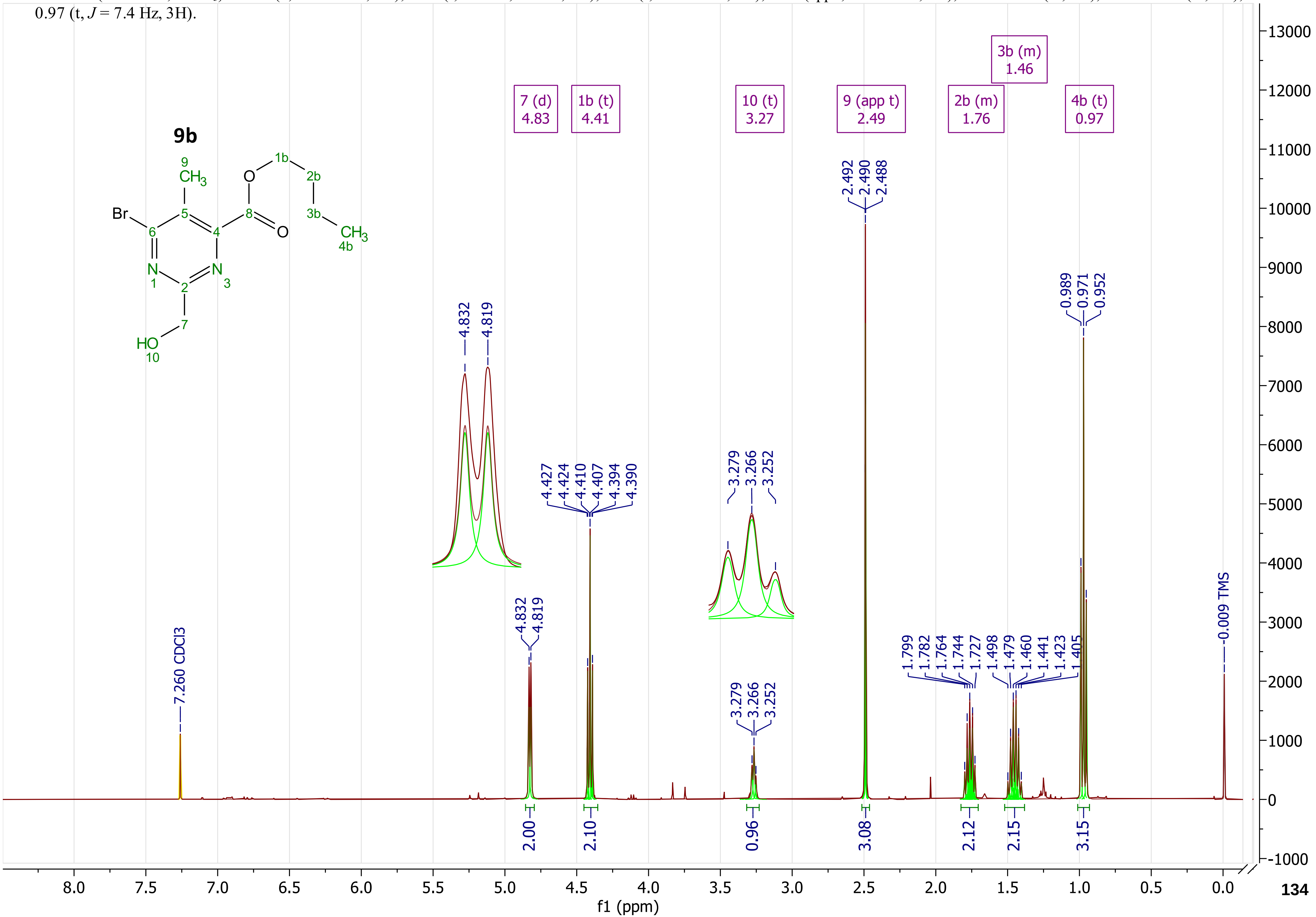

13C ^{13}C NMR (101 MHz, $CDCl_3$) δ 166.5, 164.6, 158.0, 156.5, 129.5, 66.7, 64.1, 30.6, 19.2, 17.7, 13.8.

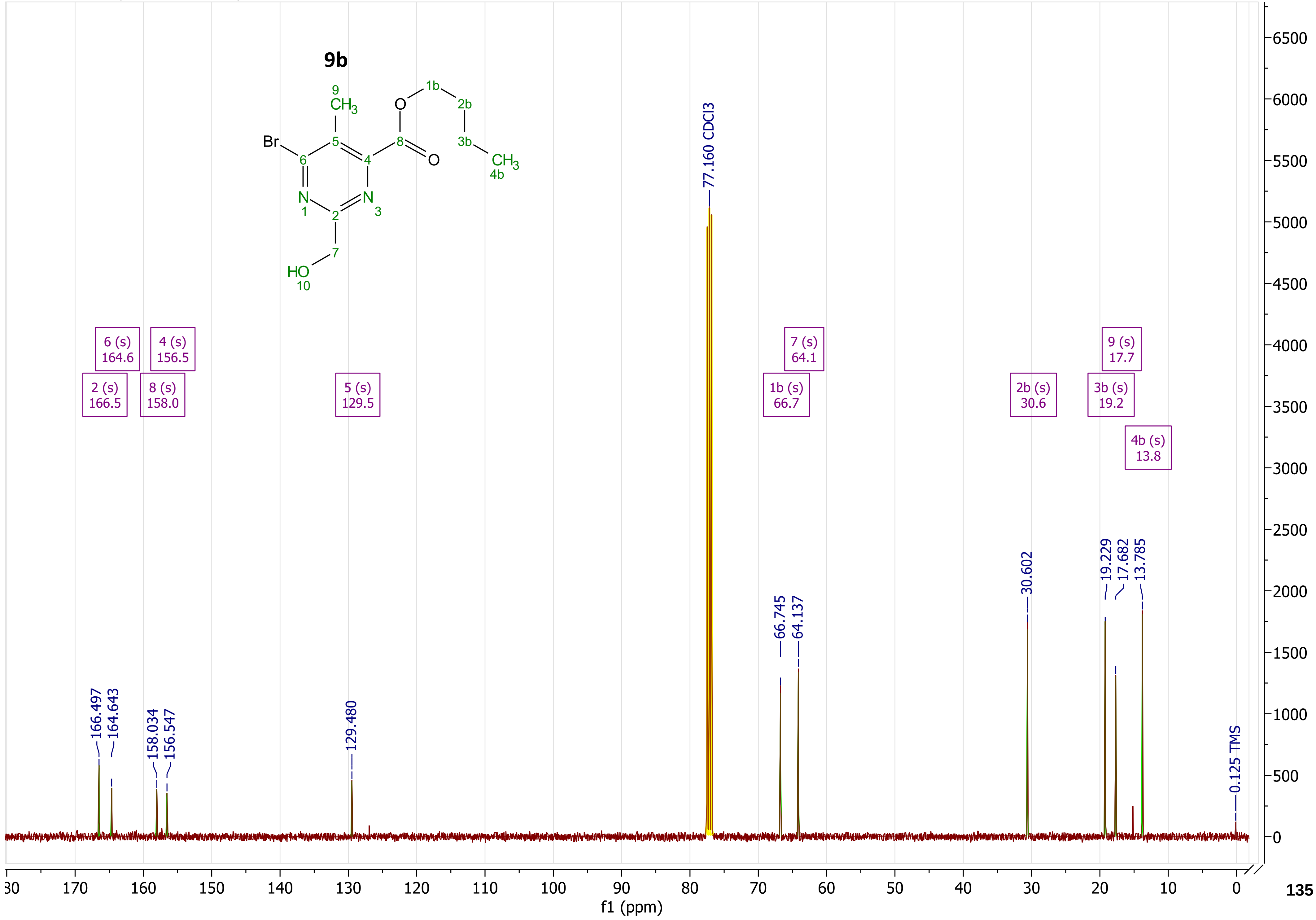

13C HSQC

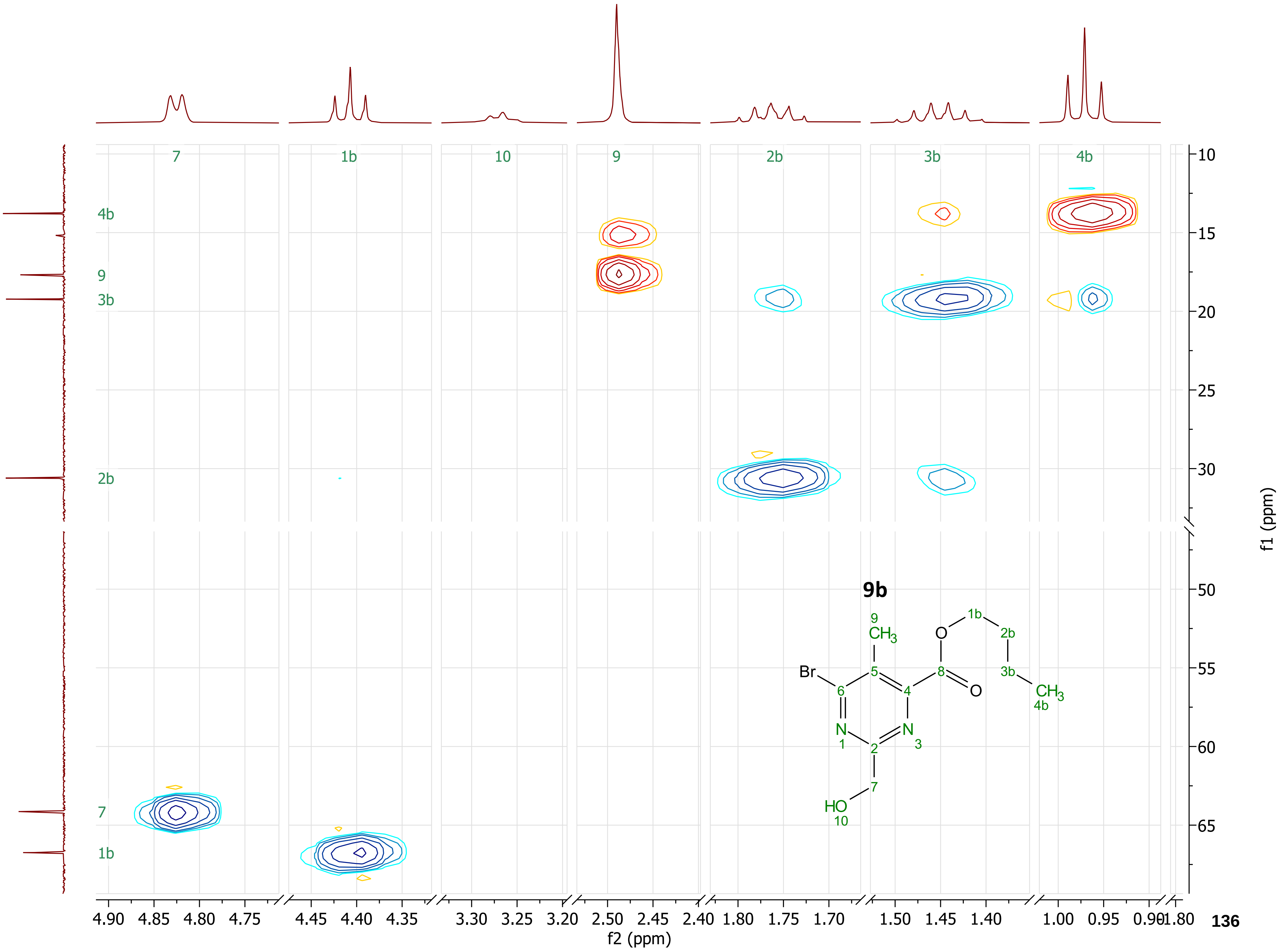

13C HMBC

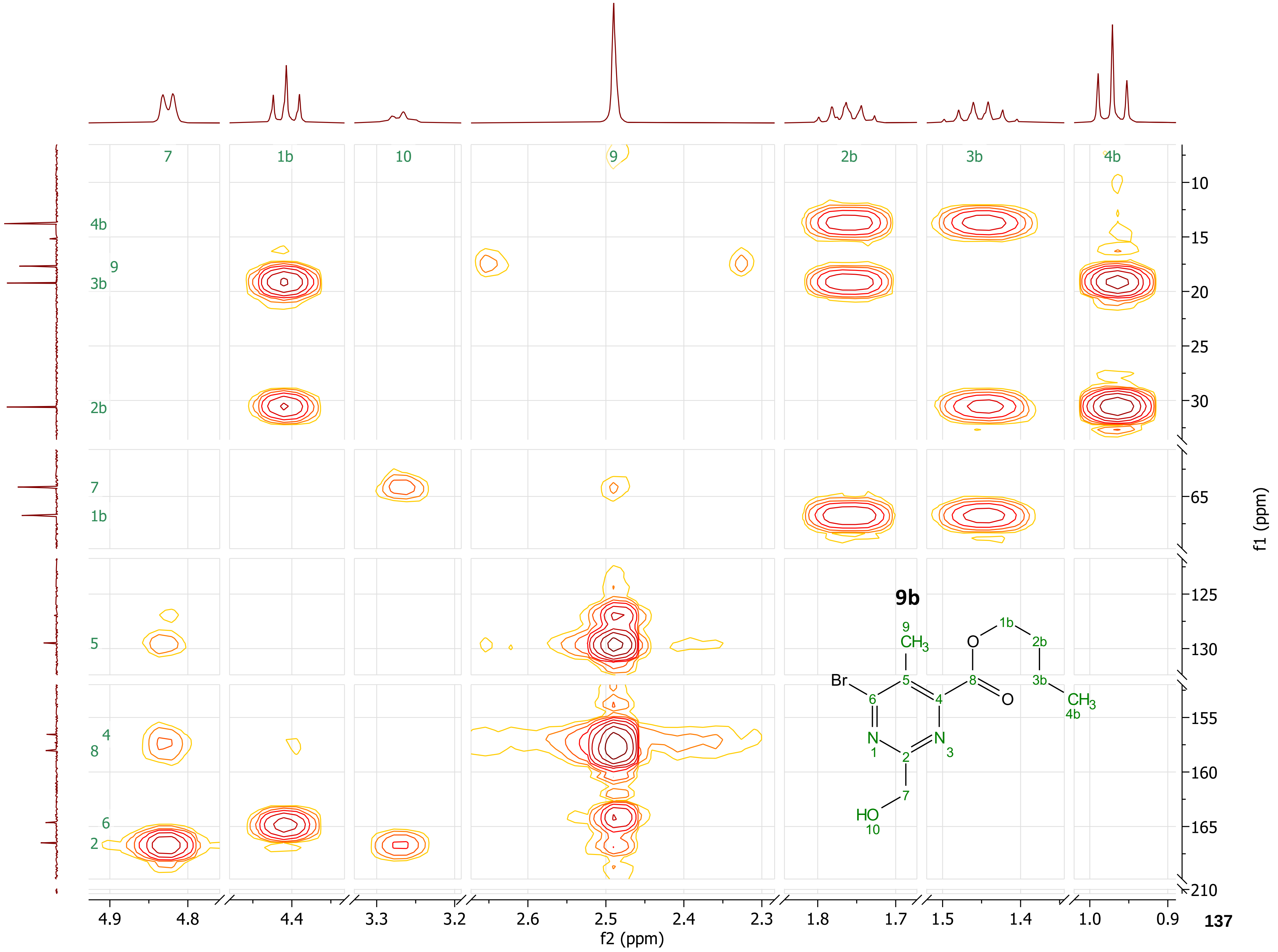

15N HMBC

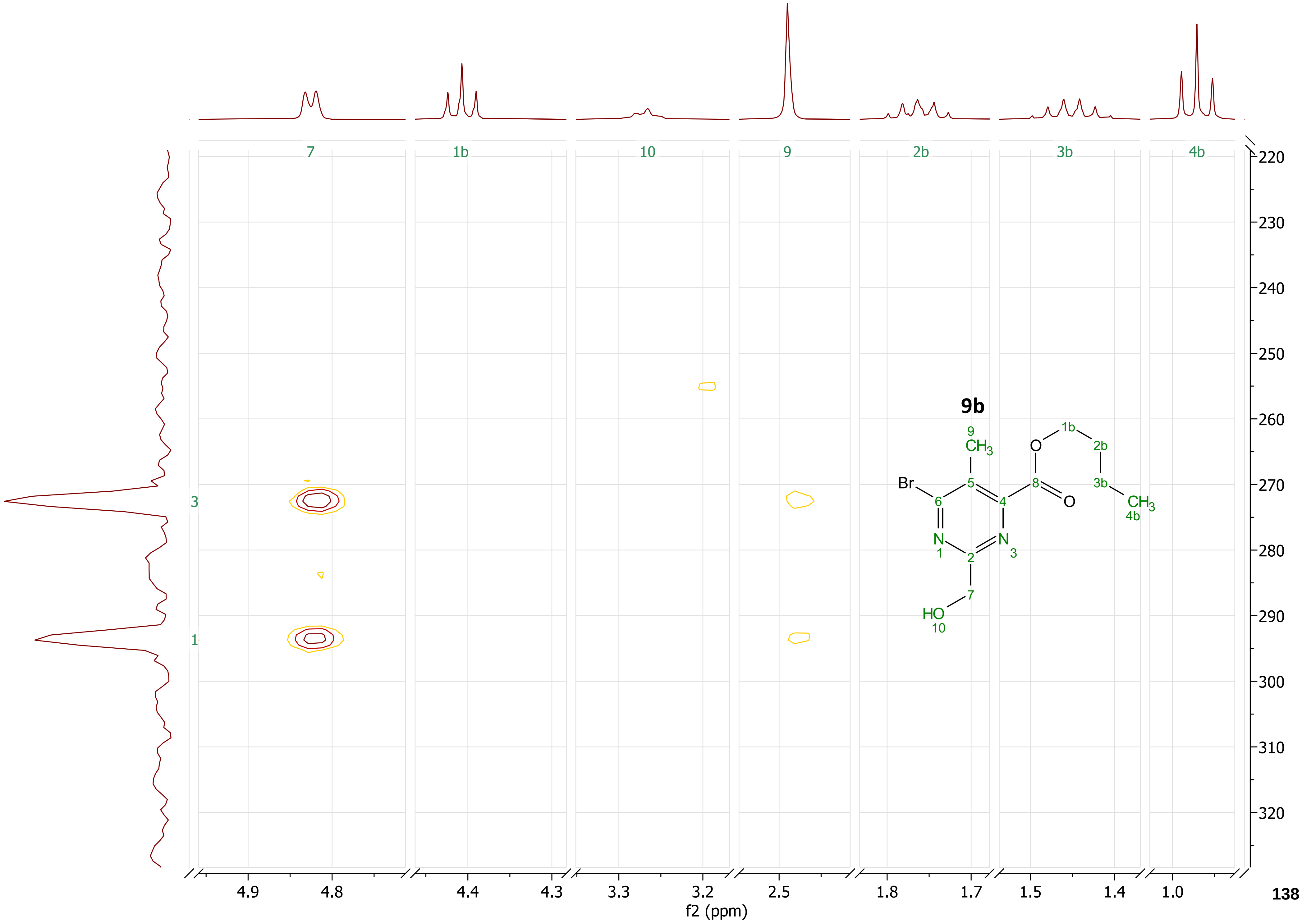

15N HMBC - f1 internal projection ^{15}N NMR (41 MHz, CDCl$_3$) δ 293.60, 272.54.

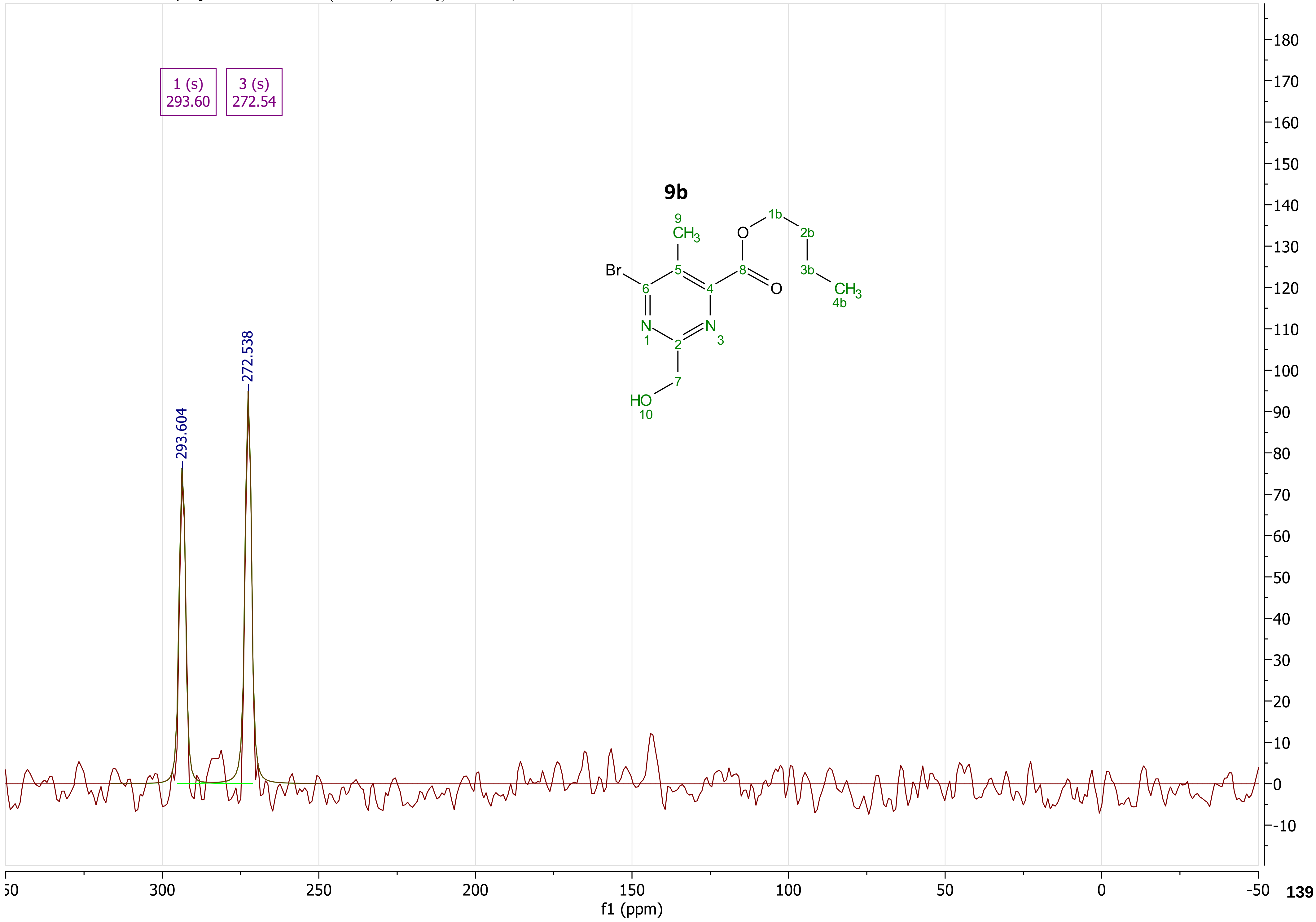

1H ^{1}H NMR (400 MHz, $CDCl_3$) δ 4.83 (d, *J* = 4.5 Hz, 2H), 4.40 (t, *J* = 6.8 Hz, 2H), 3.25 (t, *J* = 5.4 Hz, 1H), 2.50 (app t, *J* = 0.8 Hz, 3H), 1.84 – 1.72 (m, 2H), 1.47 – 1.38 (m, 2H), 1.37 – 1.24 (m, 6H), 0.89 (app t, *J* = 6.9 Hz, 3H).

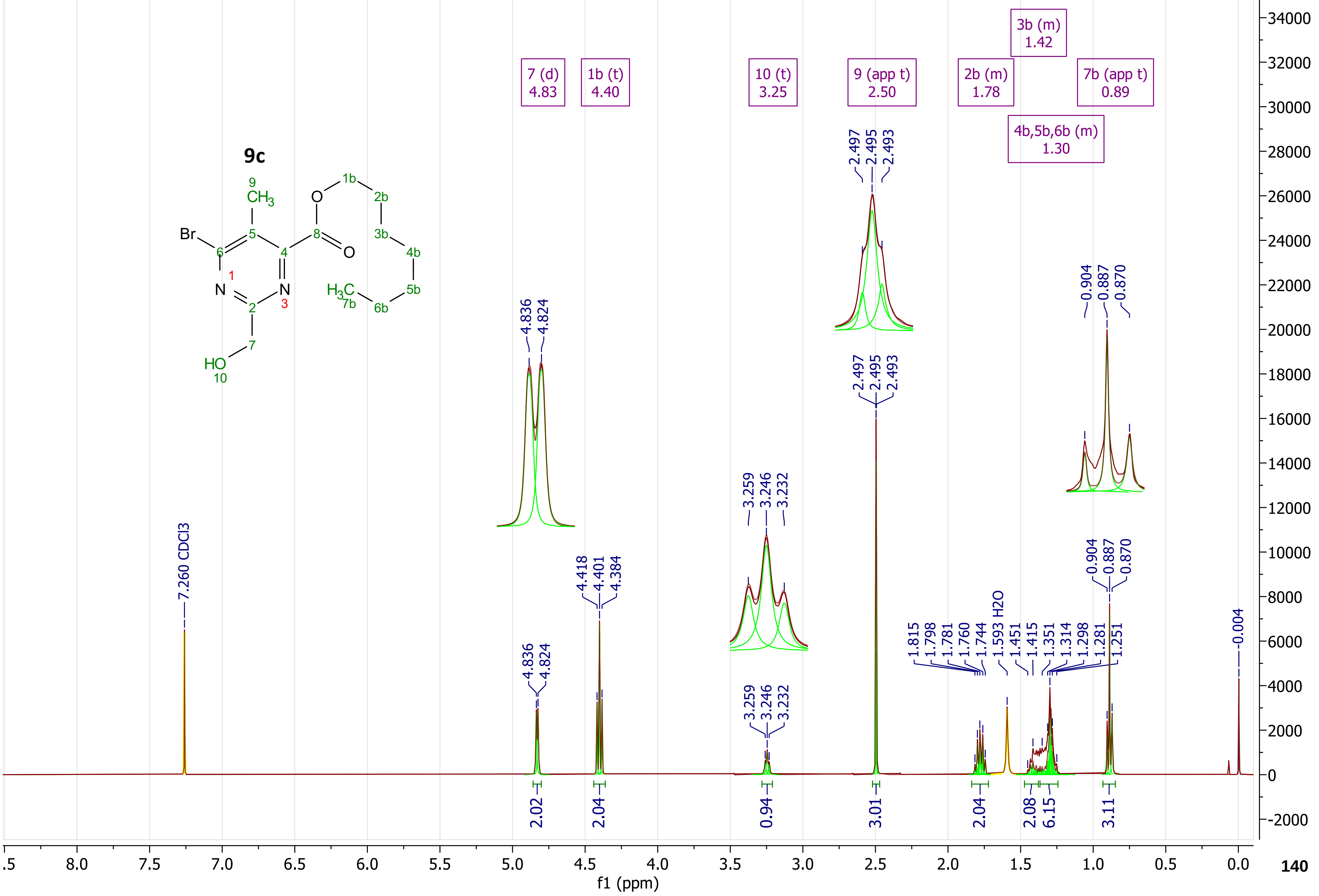

13C ^{13}C NMR (101 MHz, $CDCl_3$) δ 166.5, 164.7, 158.1, 156.6, 129.5, 67.1, 64.1, 31.8, 29.0, 28.6, 25.9, 22.7, 17.7, 14.2.

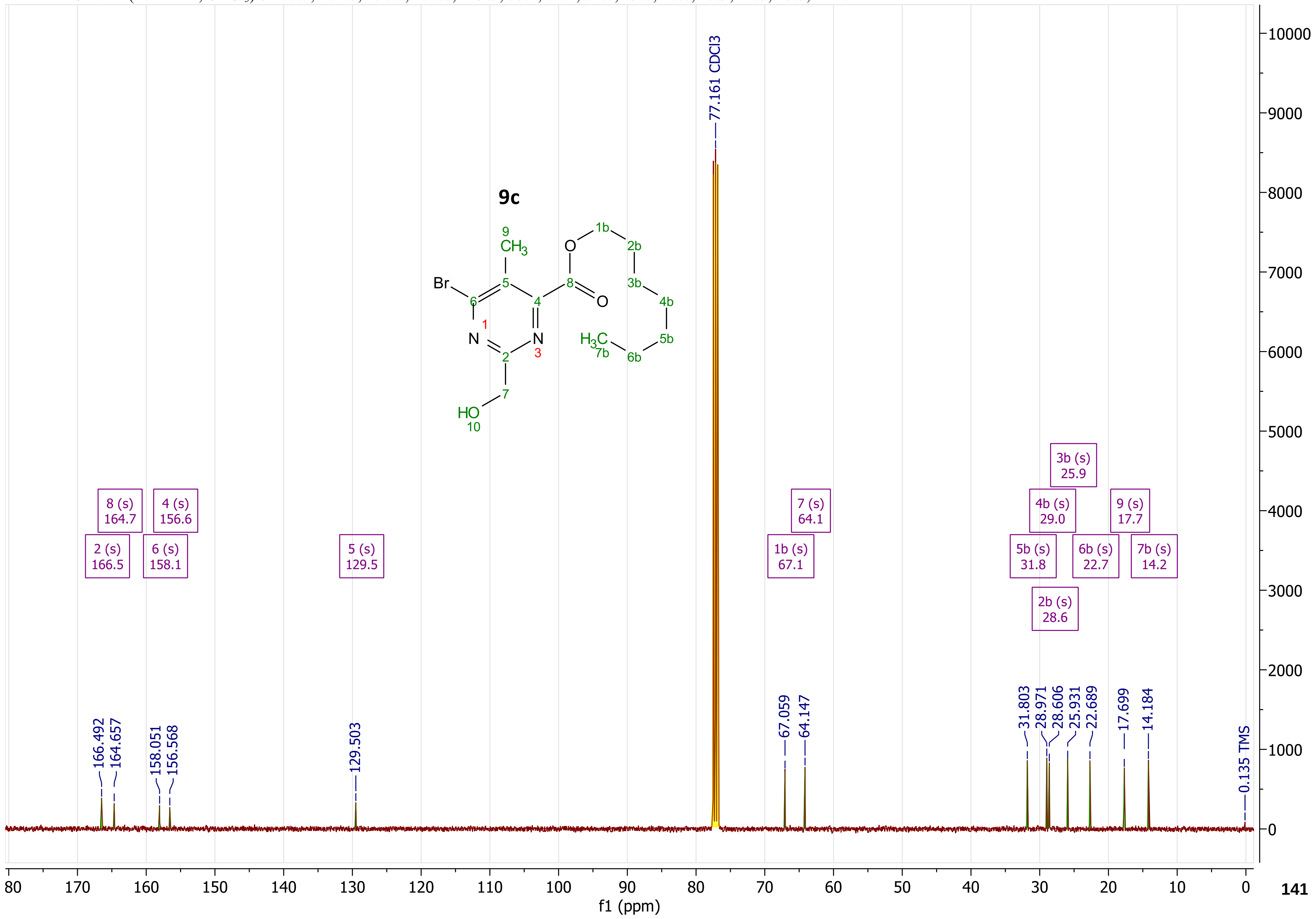

13C HSQC

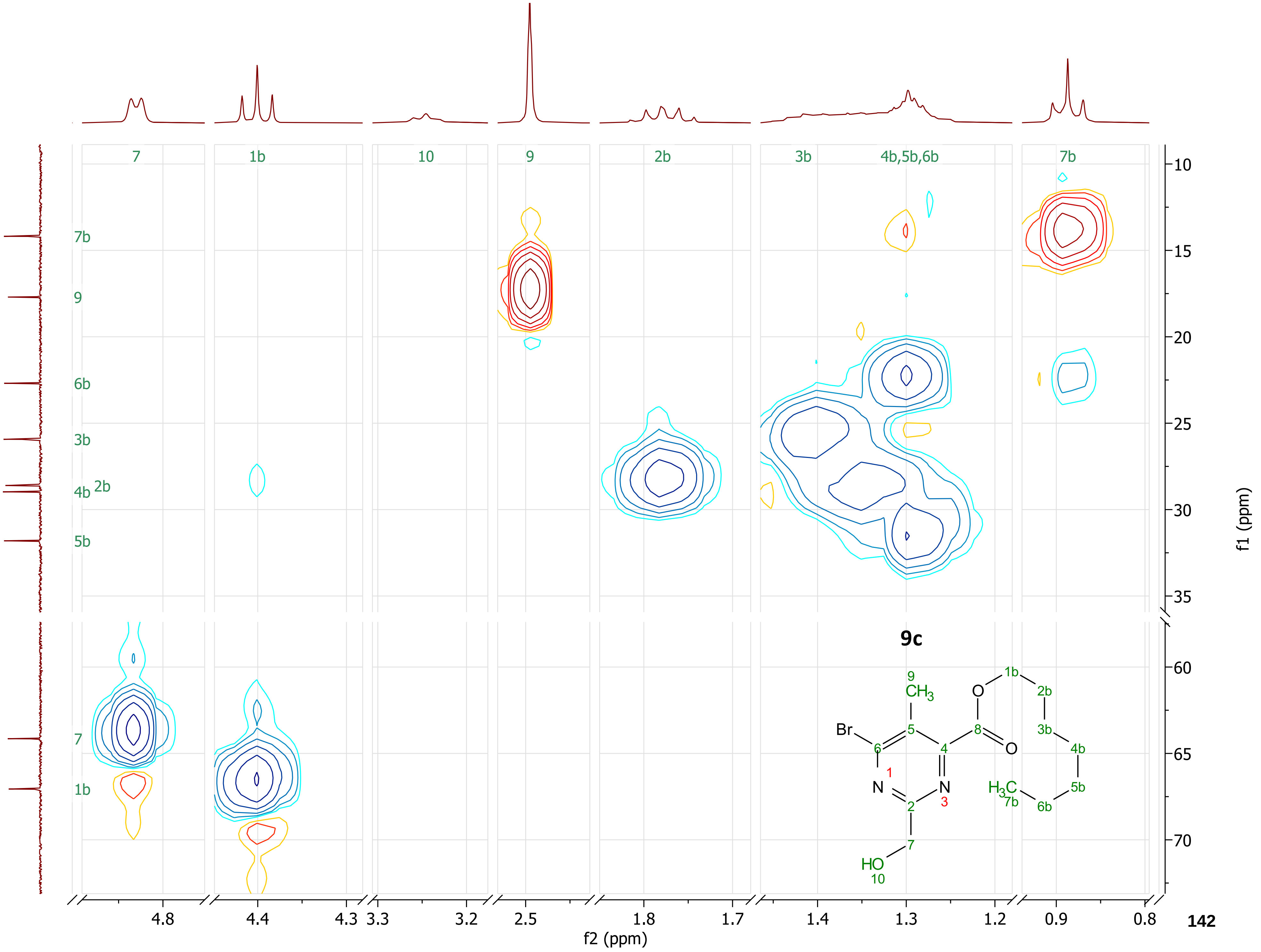
7
1b
10
9
2b
3b
4b,5b,6b
7b
7b
9
6b
3b
4b
2b
5b
7
1b
9c
9
CH3
Br
5
6
4
8
O
O
1b
2b
3b
4b
5b
6b
H3C
7b
N
1
N
3
2
7
HO
10
4.8
4.4
4.3
3.3
3.2
2.5
1.8
1.7
1.4
1.3
1.2
0.9
0.8
f2 (ppm)
10
15
20
25
30
35
60
65
70
f1 (ppm)

13C HMBC

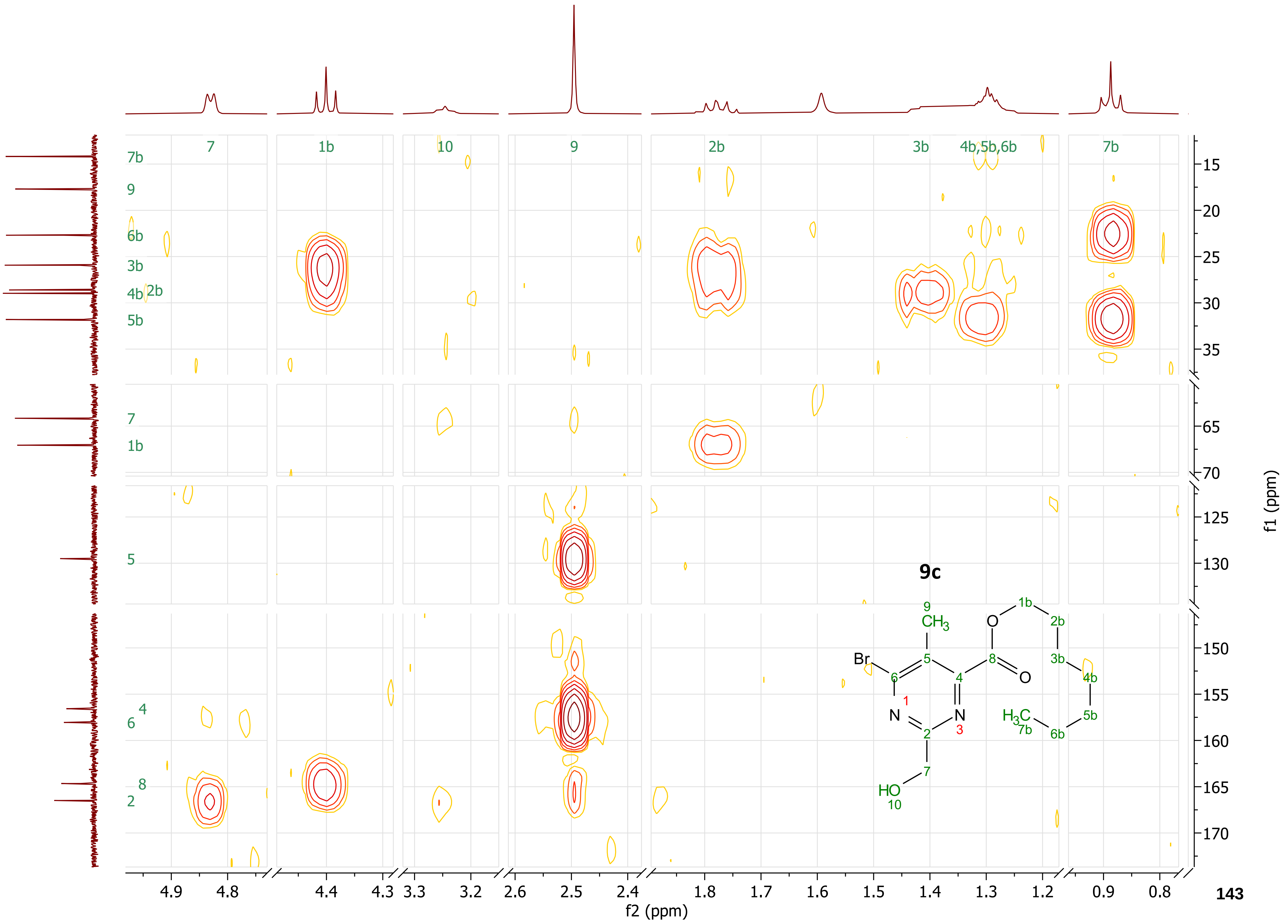

7b
9
6b
3b
4b
2b
5b
7
1b
5
4
6
8
2
10
4b,5b,6b
f2 (ppm)
f1 (ppm)
9c
CH3
Br
N
HO
O
H3C

1H ^{1}H NMR (400 MHz, $CDCl_3$) δ 4.83 (app q, J = 0.8 Hz, 2H), 4.40 (t, J = 6.8 Hz, 2H), 3.62 (br s, 1H), 2.50 (app t, J = 0.8 Hz, 3H), 1.84 – 1.72 (m, 2H), 1.47 – 1.37 (m, 2H), 1.40 – 1.21 (m, 8H), 0.88 (t, J = 6.9 Hz, 3H).

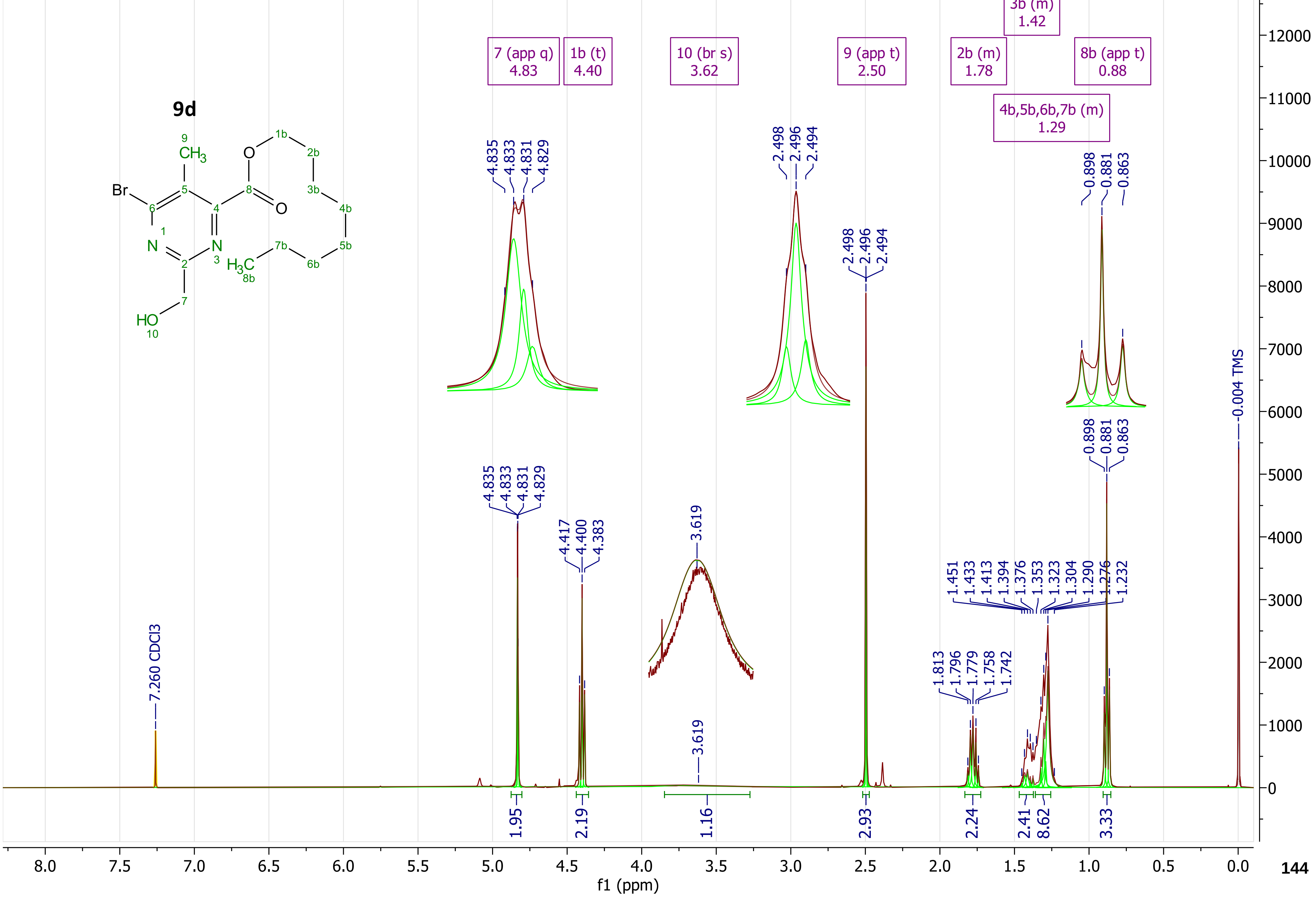

13C ^{13}C NMR (101 MHz, $CDCl_3$) δ 166.5, 164.6, 158.0, 156.6, 129.5, 67.0, 64.1, 31.9, 29.3 (2C), 28.6, 26.0, 22.7, 17.7, 14.2.

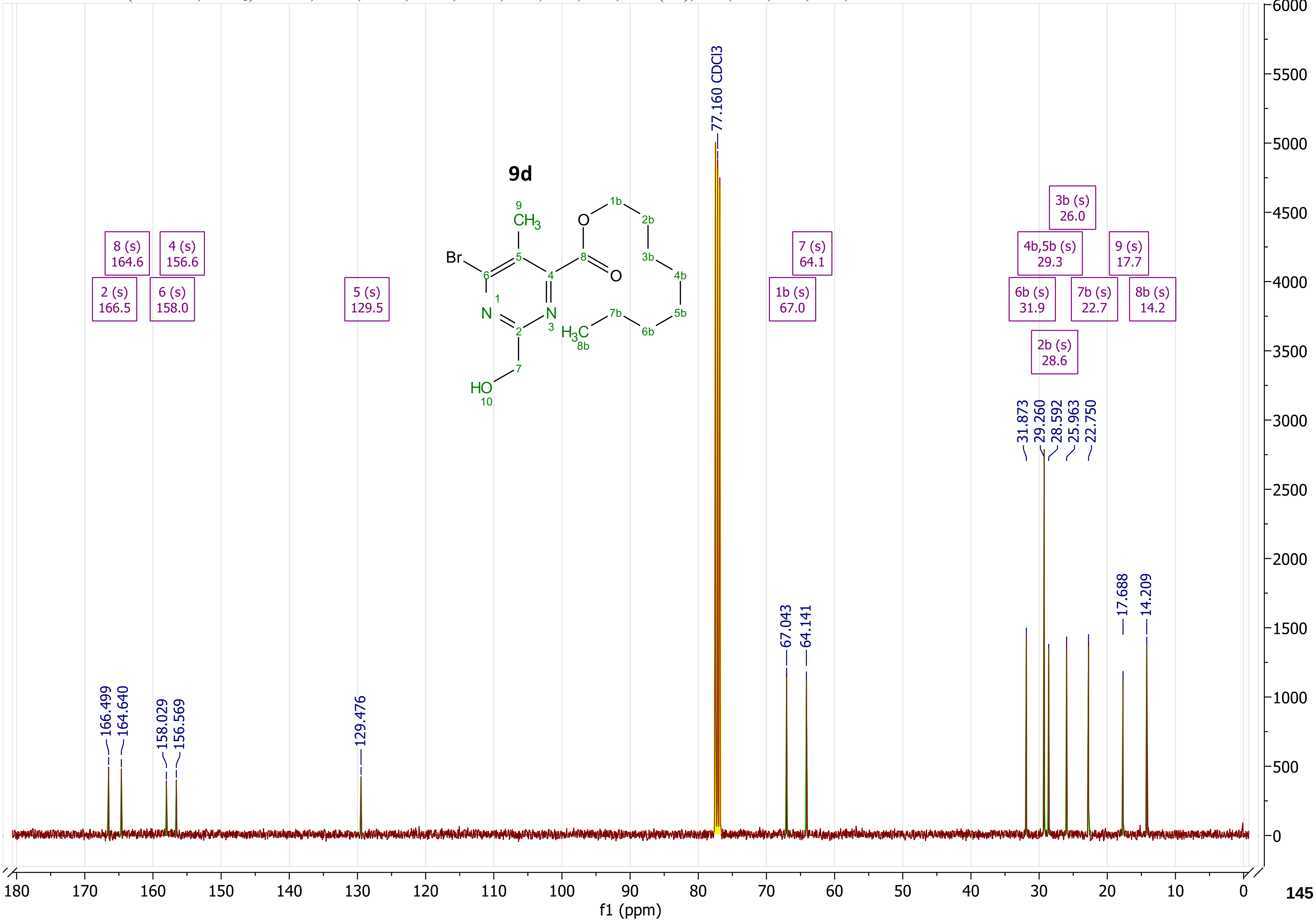

13C HSQC

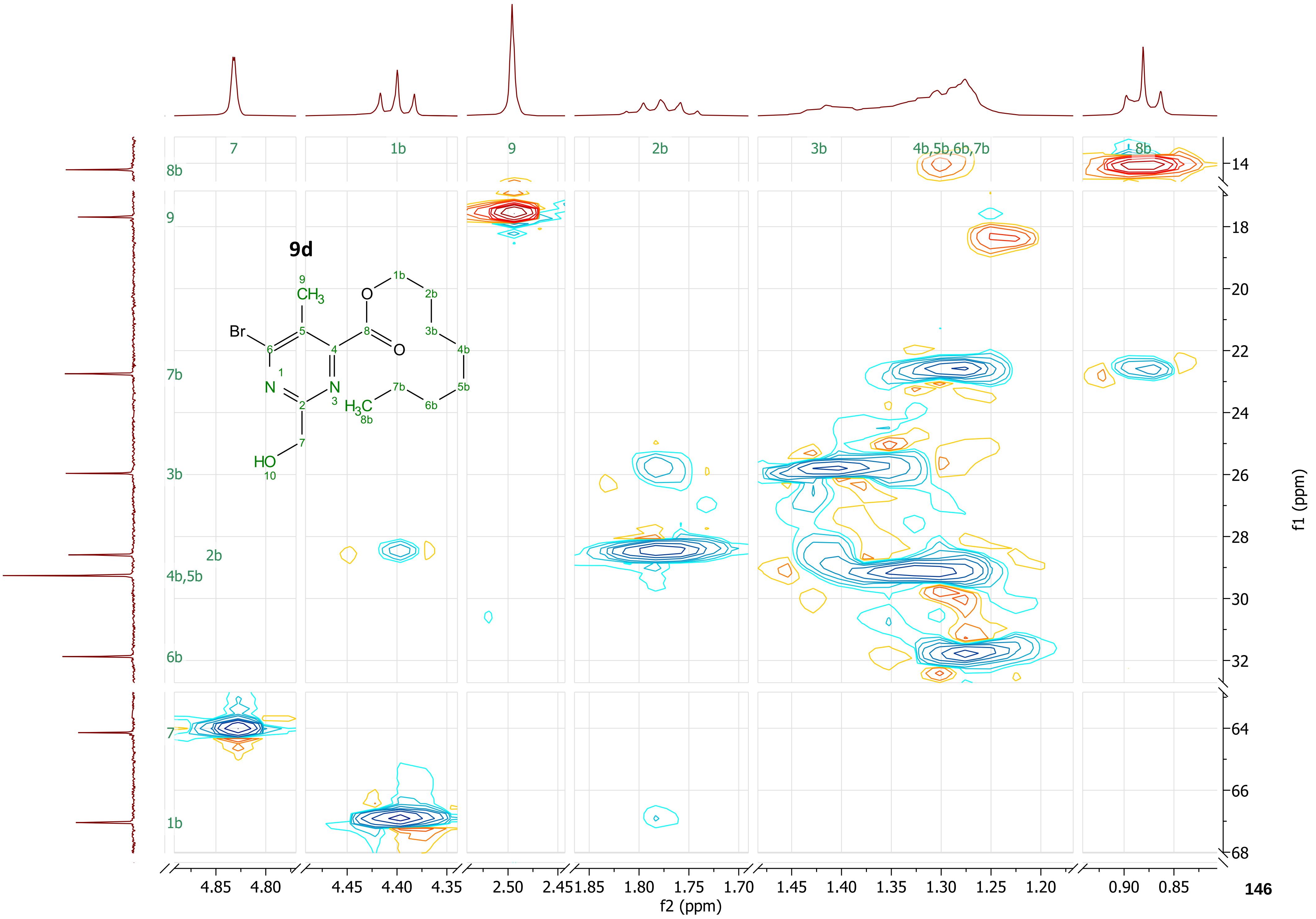

9d
7
1b
9
2b
3b
4b,5b,6b,7b
8b
9
7b
3b
2b
4b,5b
6b
7
1b
Br
CH3
O
O
N
N
H3C
HO
f2 (ppm)
f1 (ppm)

13C HMBC

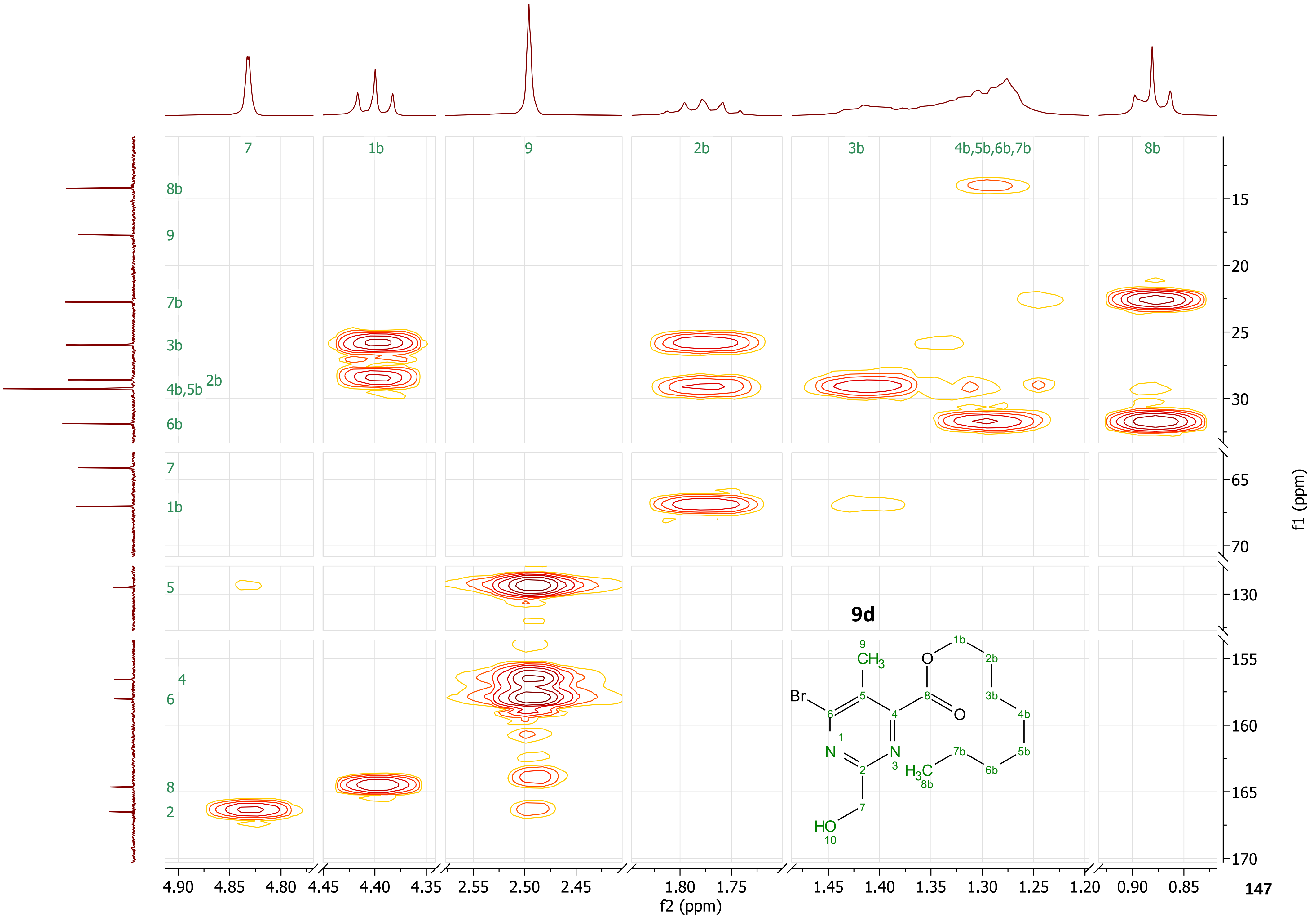

7
1b
9
2b
3b
4b,5b,6b,7b
8b
8b
9
7b
3b
4b,5b
2b
6b
7
1b
5
4
6
8
2
9d
f2 (ppm)
f1 (ppm)

15N HMBC

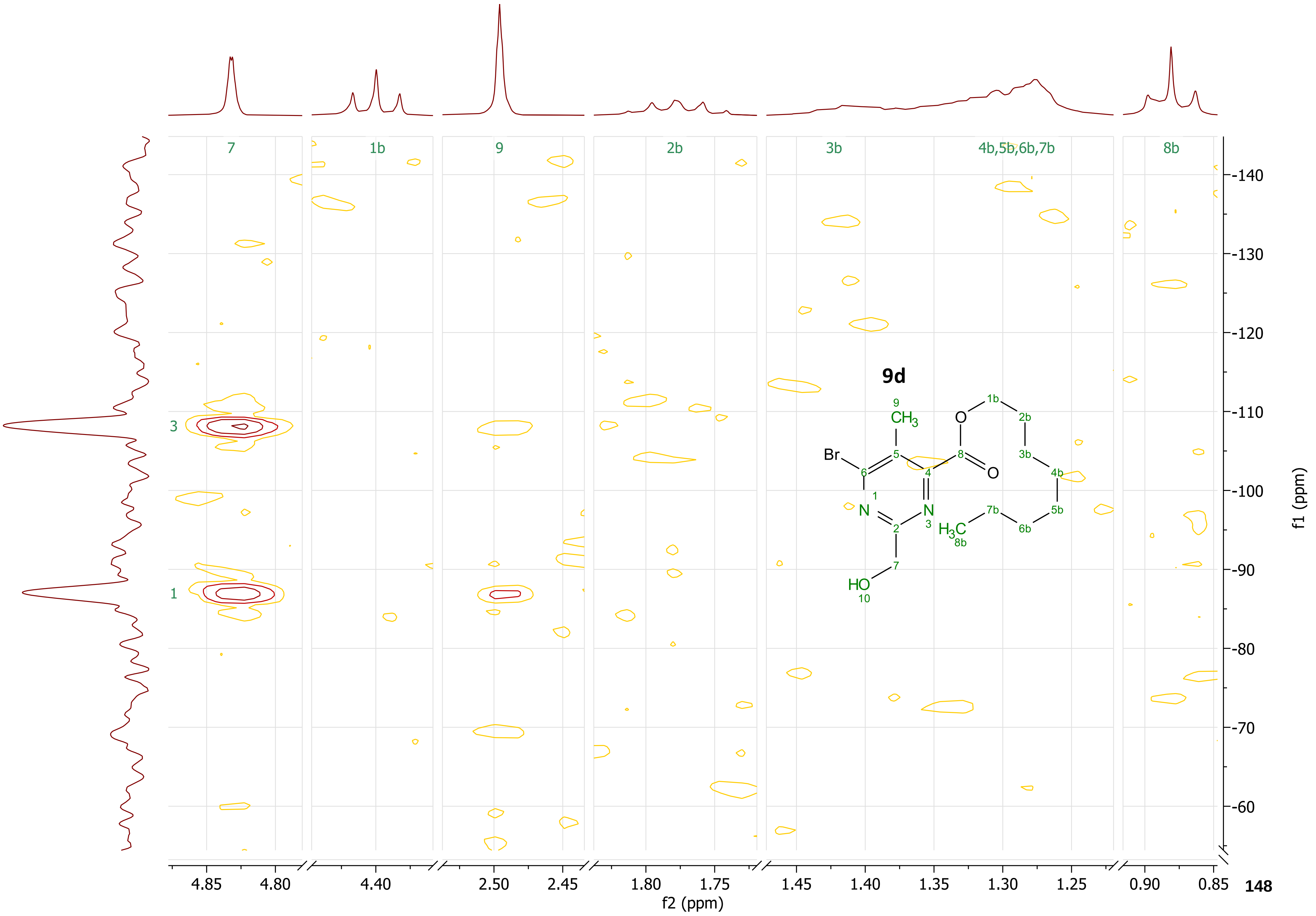

15N HMBC - f1 internal projection ^{15}N NMR (41 MHz, $CDCl_3$) δ -87.03, -108.22.

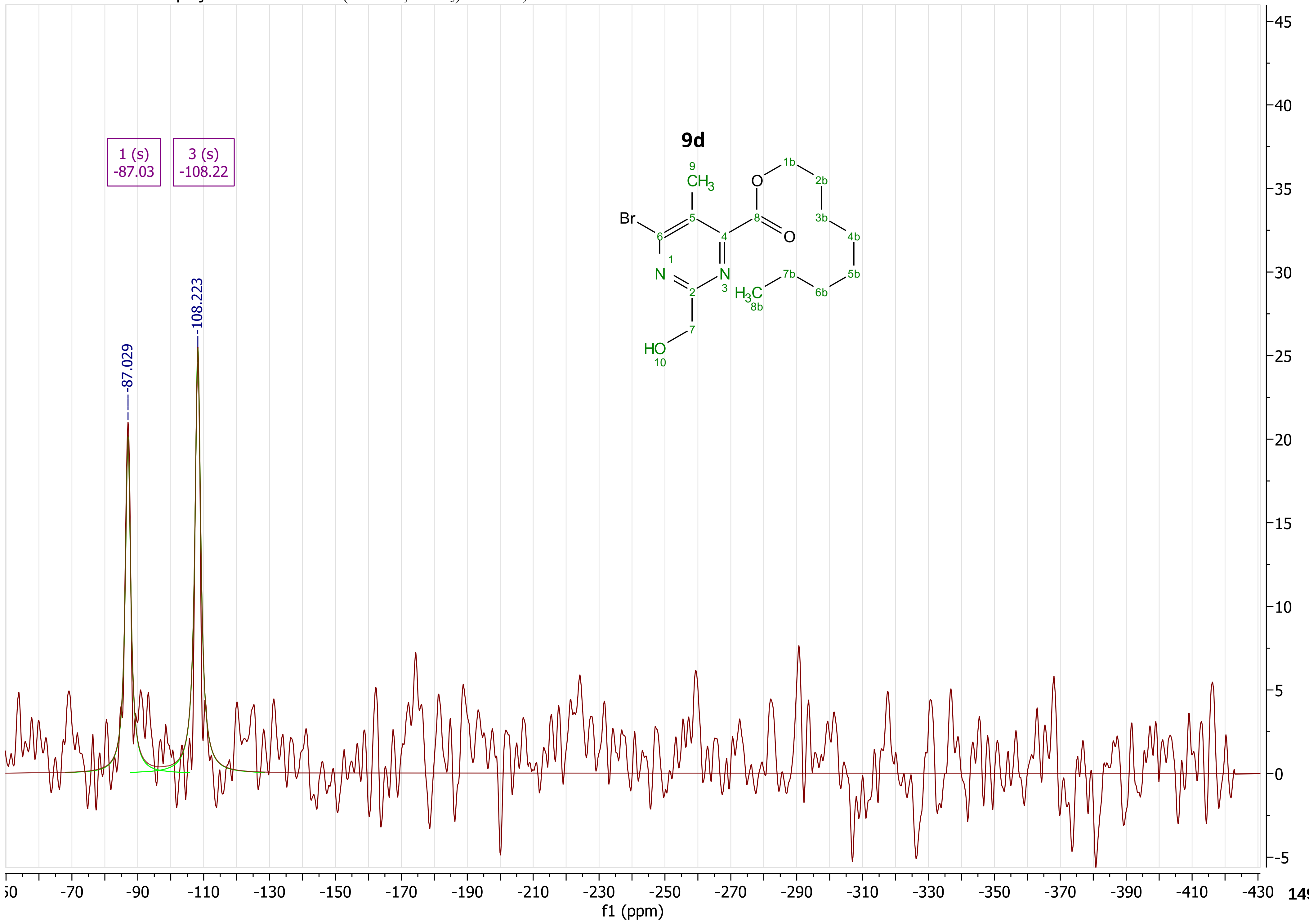

1H ^{1}H NMR (400 MHz, $CDCl_3$) δ 4.82 (d, *J* = 4.9 Hz, 2H), 4.39 (t, *J* = 6.8 Hz, 2H), 3.29 (t, *J* = 5.4 Hz, 1H), 2.49 (app t, *J* = 0.8 Hz, 3H), 1.82 – 1.72 (m, 2H), 1.46 – 1.37 (m, 2H), 1.36 – 1.19 (m, 10H), 0.87 (app t, *J* = 6.9 Hz, 3H).

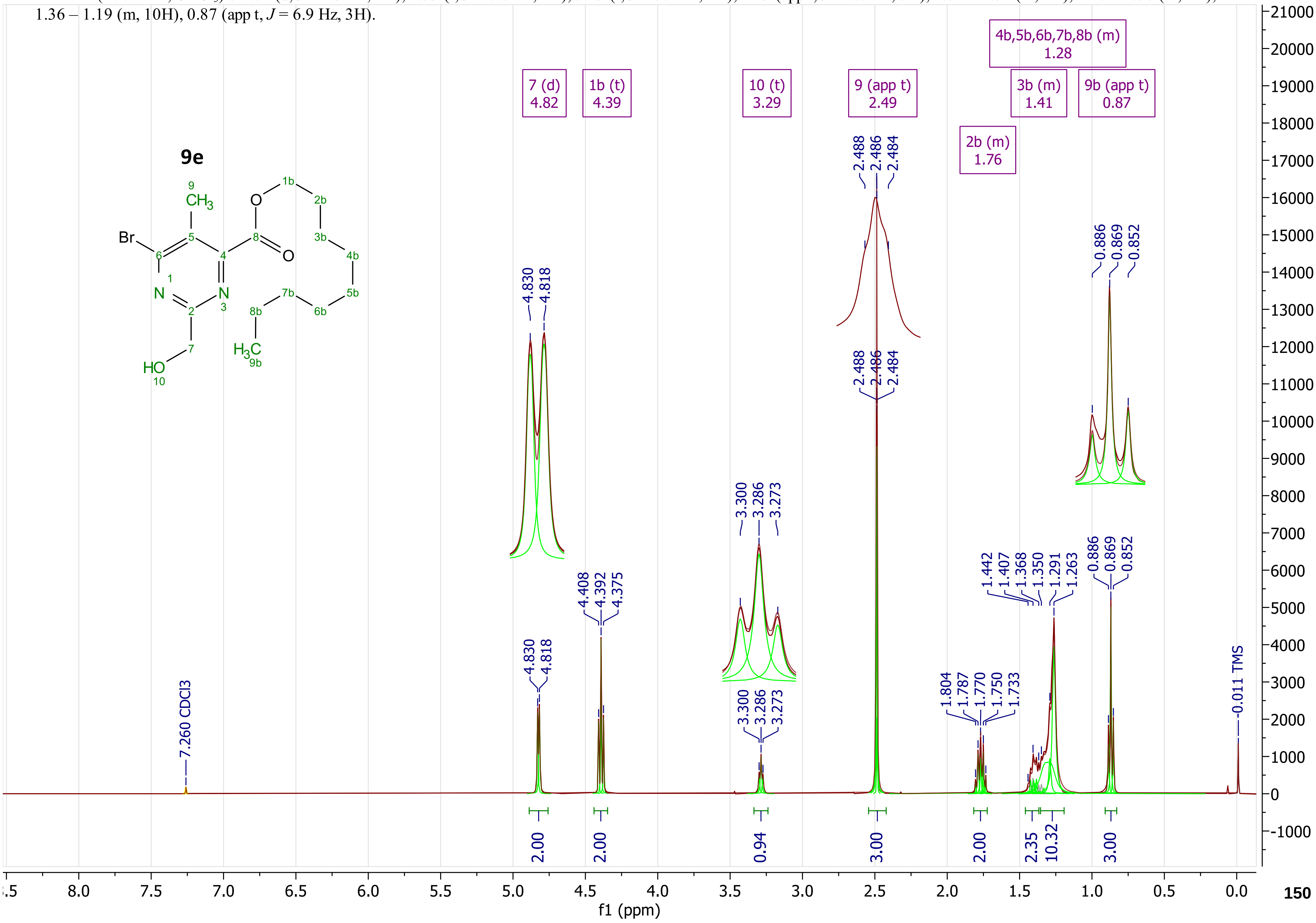

13C ^{13}C NMR (101 MHz, $CDCl_3$) δ 166.5, 164.6, 158.0, 156.6, 129.5, 67.0, 64.1, 32.0, 29.6, 29.33, 29.30, 28.6, 26.0, 22.8, 17.7, 14.2.

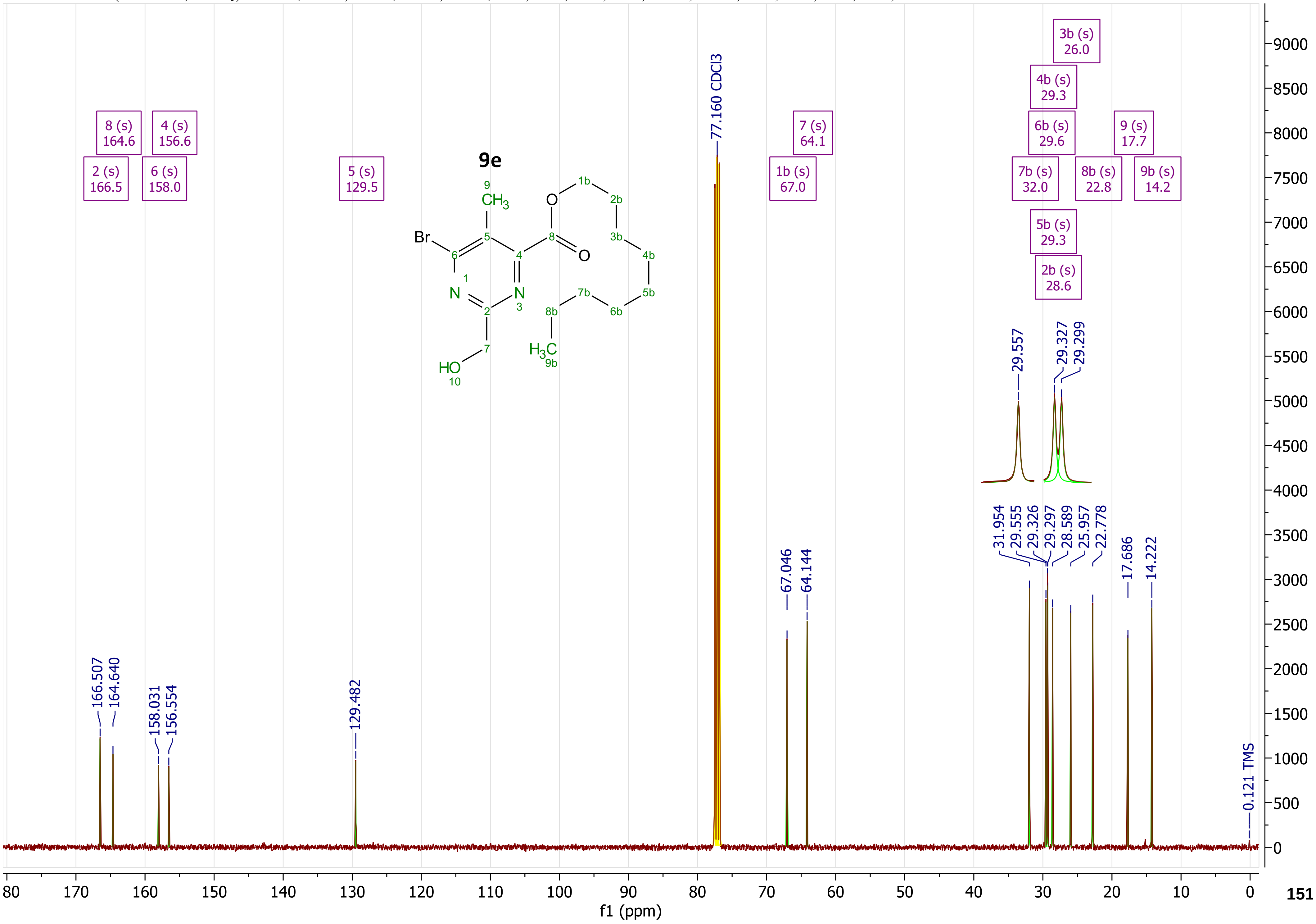

13C HSQC

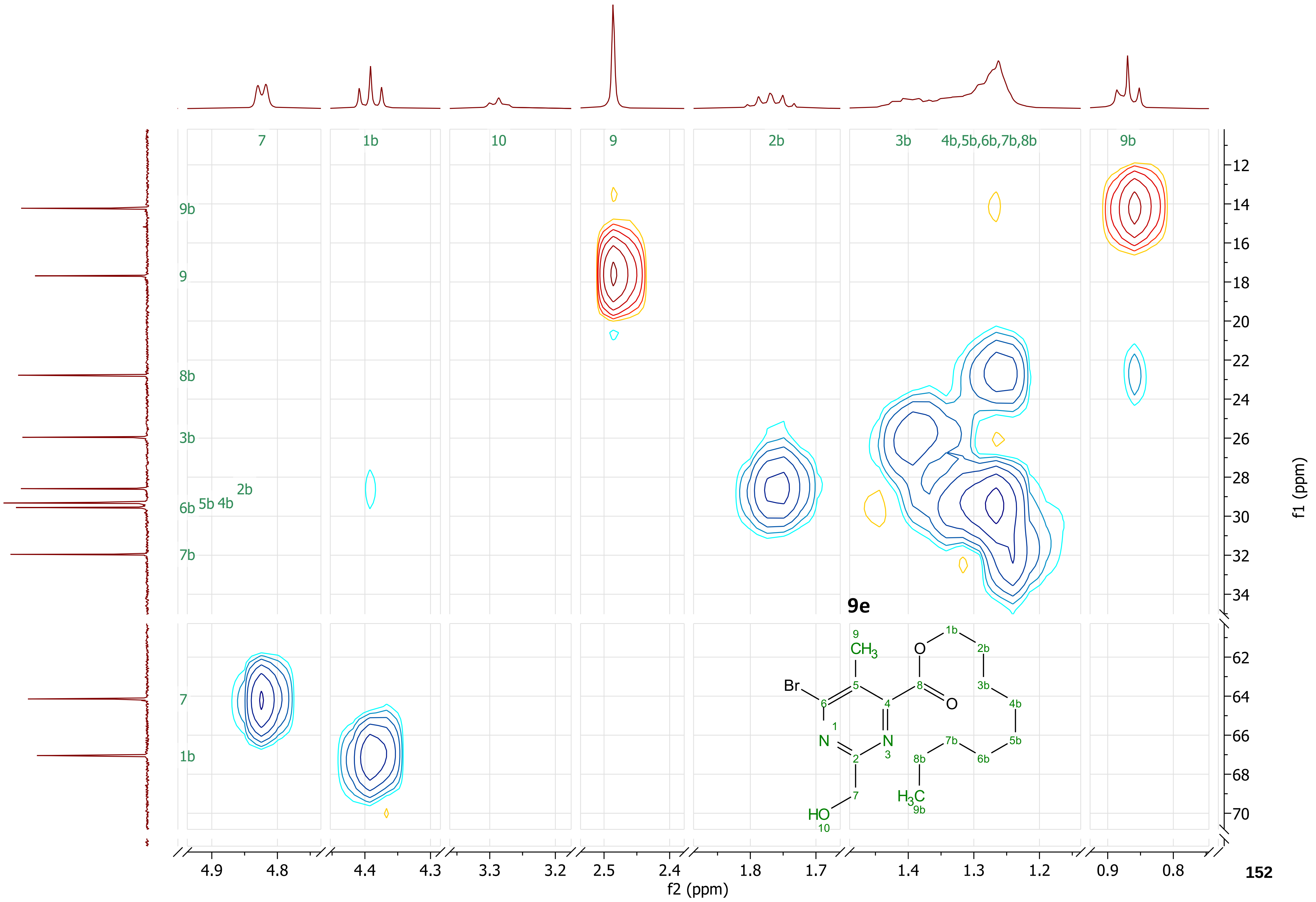

7
1b
10
9
2b
3b
4b,5b,6b,7b,8b
9b
9
8b
6b 5b 4b
9e
Br
CH3
O
HO
H3C
N
f2 (ppm)
f1 (ppm)

13C HMBC

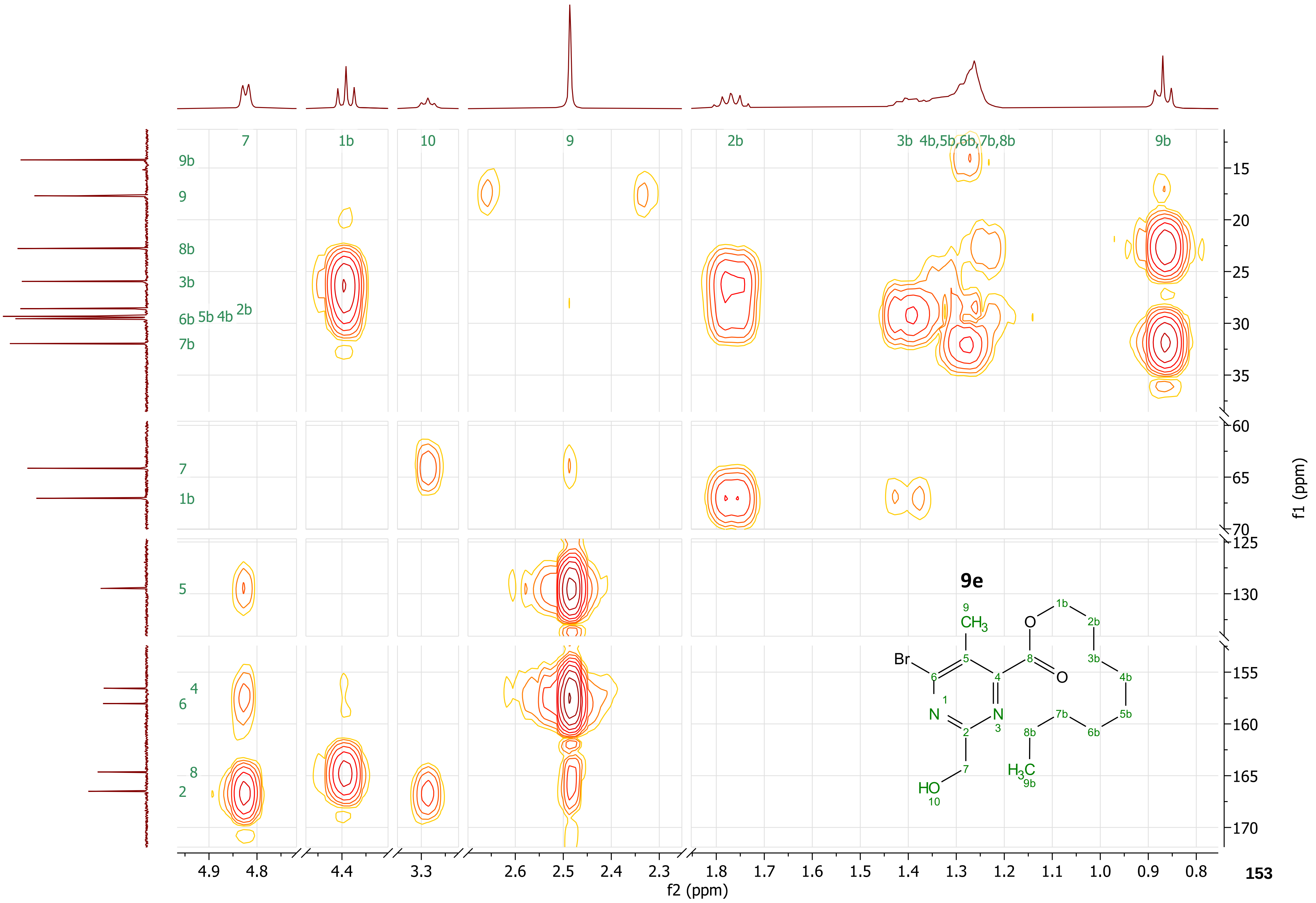

7
1b
10
9
2b
3b
4b,5b,6b,7b,8b
9b
9b
9
8b
3b
6b 5b 4b 2b
7b
7
1b
5
4
6
8
2
9e
f2 (ppm)
f1 (ppm)

15N HMBC

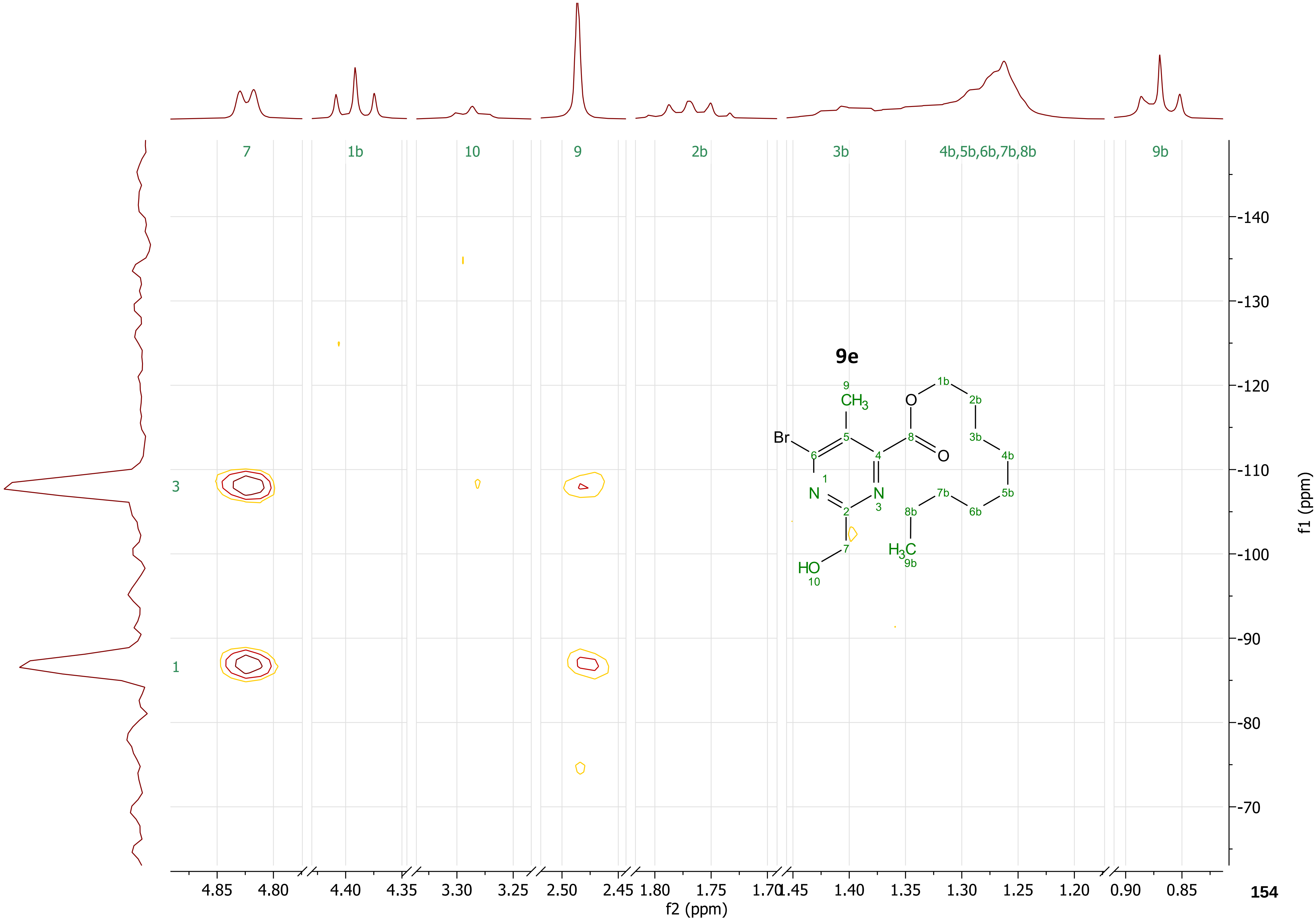

15N HMBC - f1 internal projection ^{15}N NMR (41 MHz, $CDCl_3$) δ -86.67, -108.07.

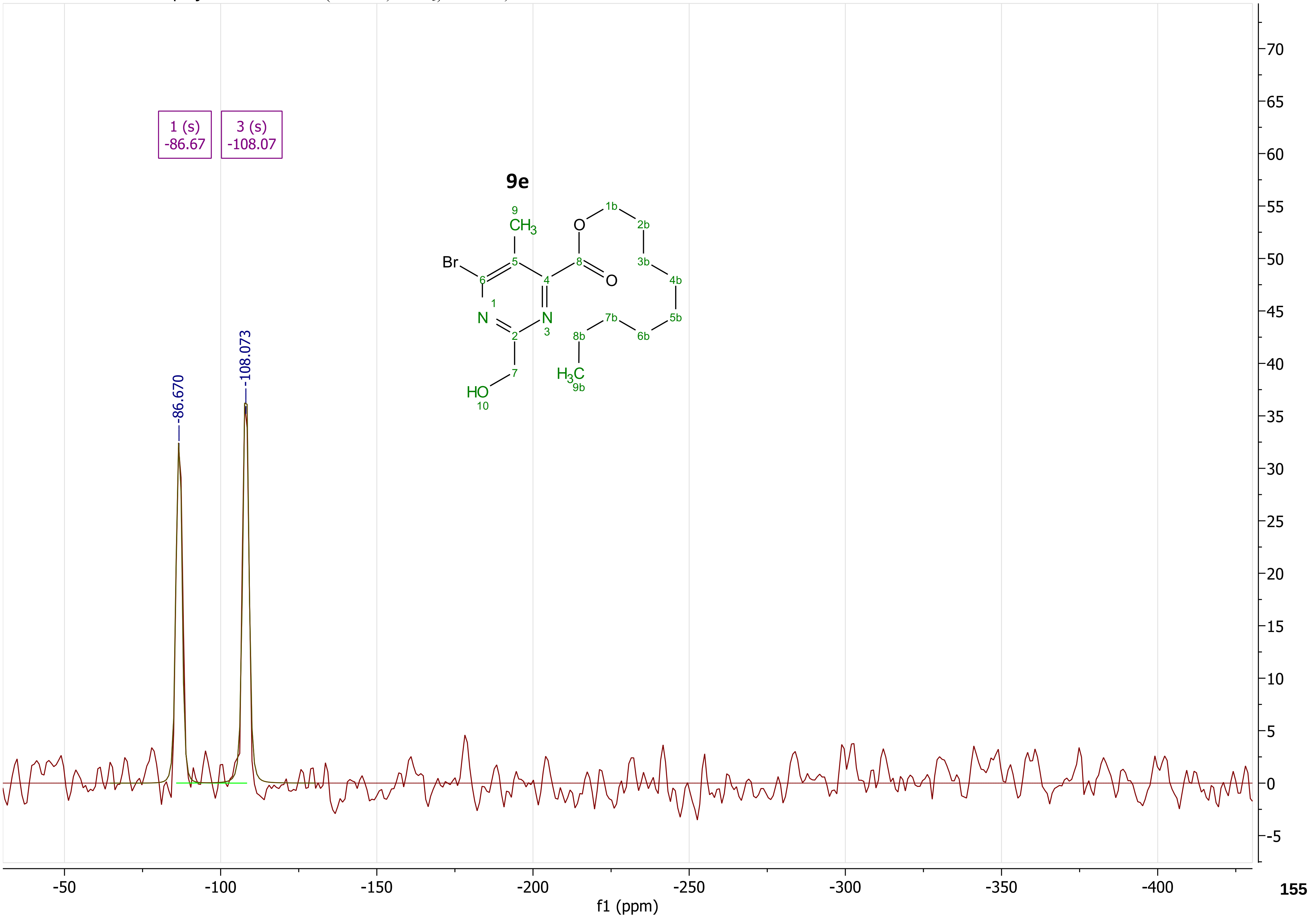

1H

^{1}H NMR (400 MHz, $CDCl_3$) δ 4.82 (app d, J = 0.8 Hz, 2H), 4.47 (q, J = 7.1 Hz, 2H), 3.30 (br s, 1H), 2.49 (app t, J = 0.8 Hz, 3H), 1.42 (t, J = 7.1 Hz, 3H).

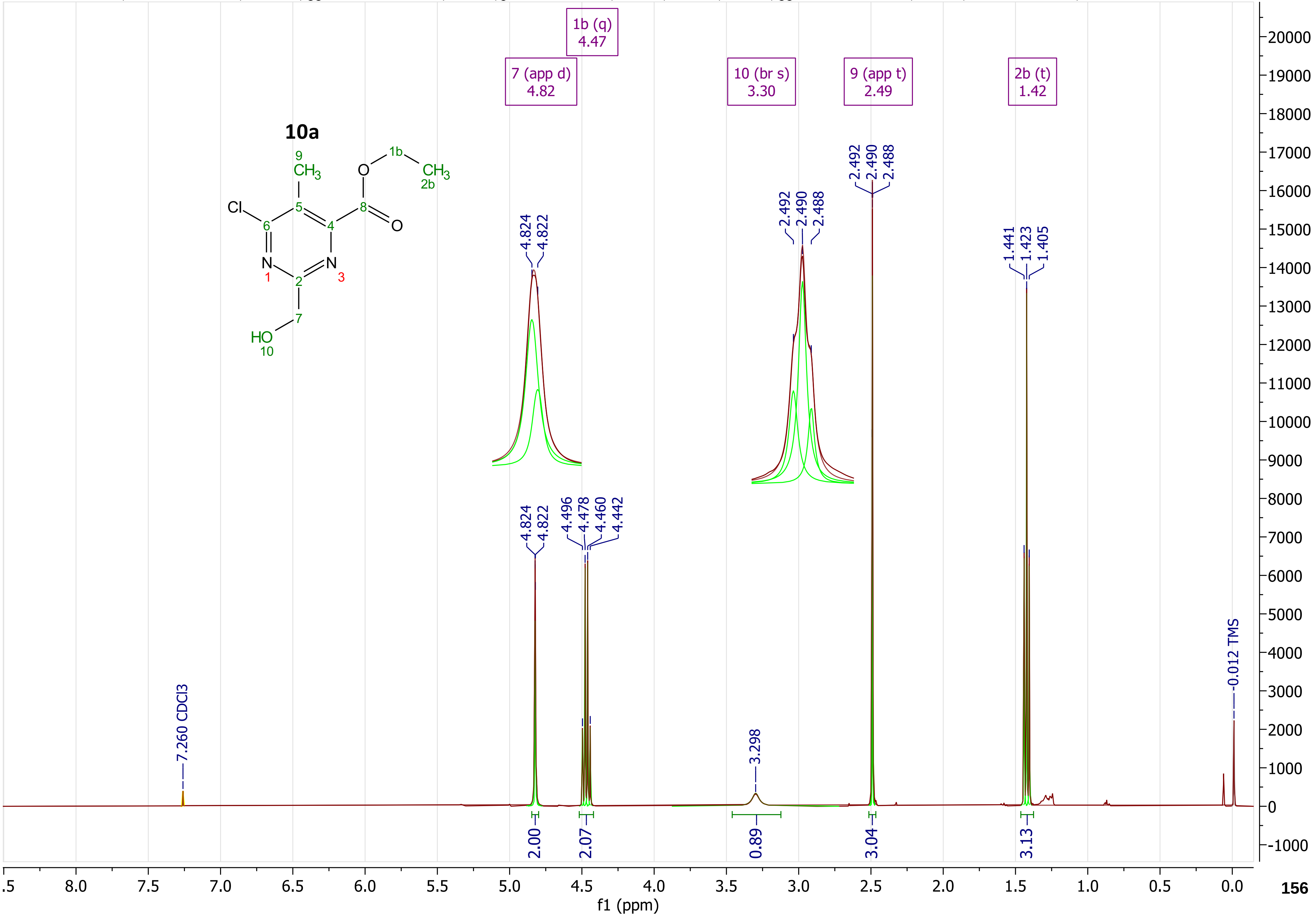

13C ^{13}C NMR (101 MHz, $CDCl_3$) δ 166.5, 164.6, 163.9, 157.2, 127.1, 64.2, 62.9, 15.2, 14.2.

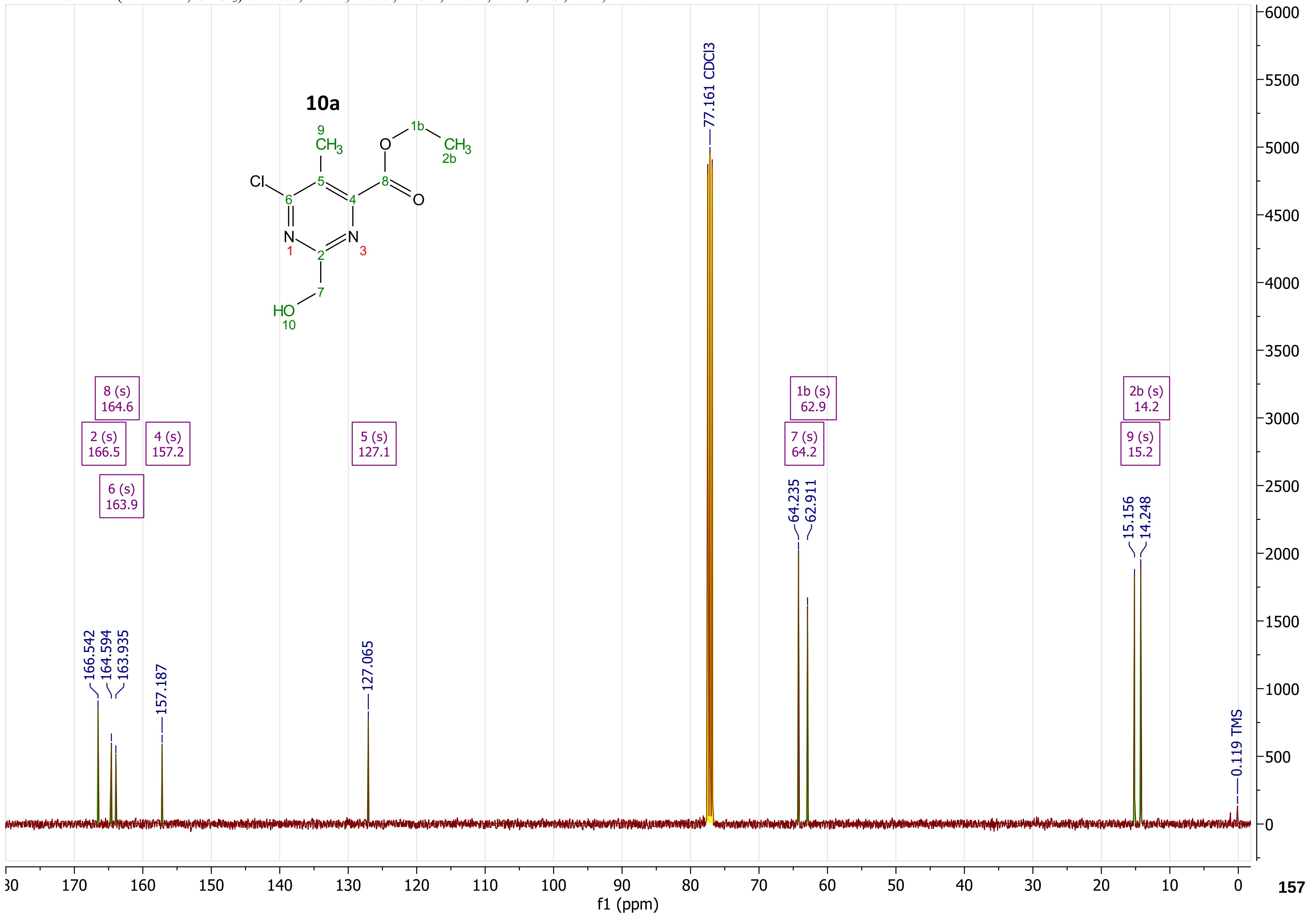

13C HSQC

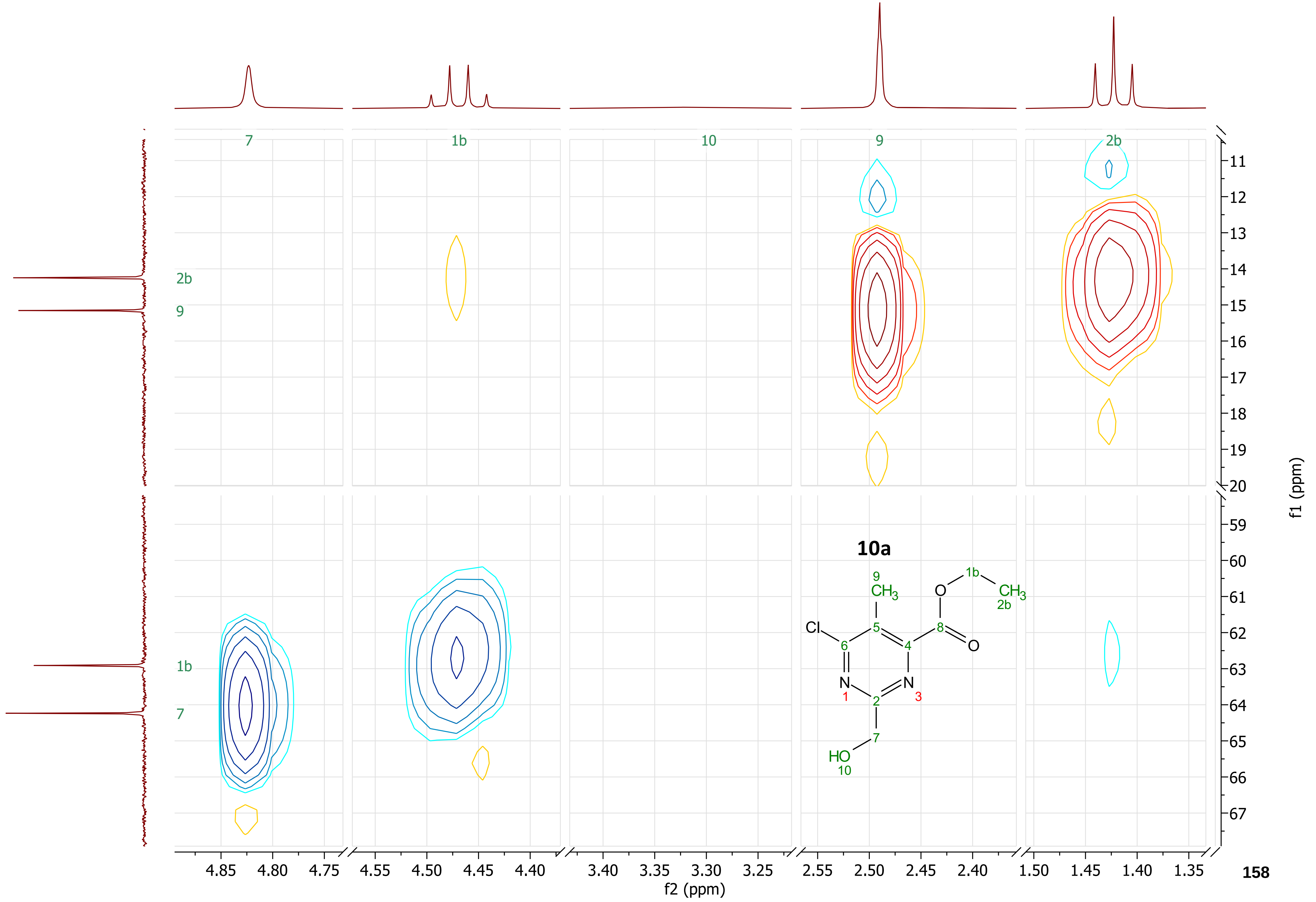

13C HMBC

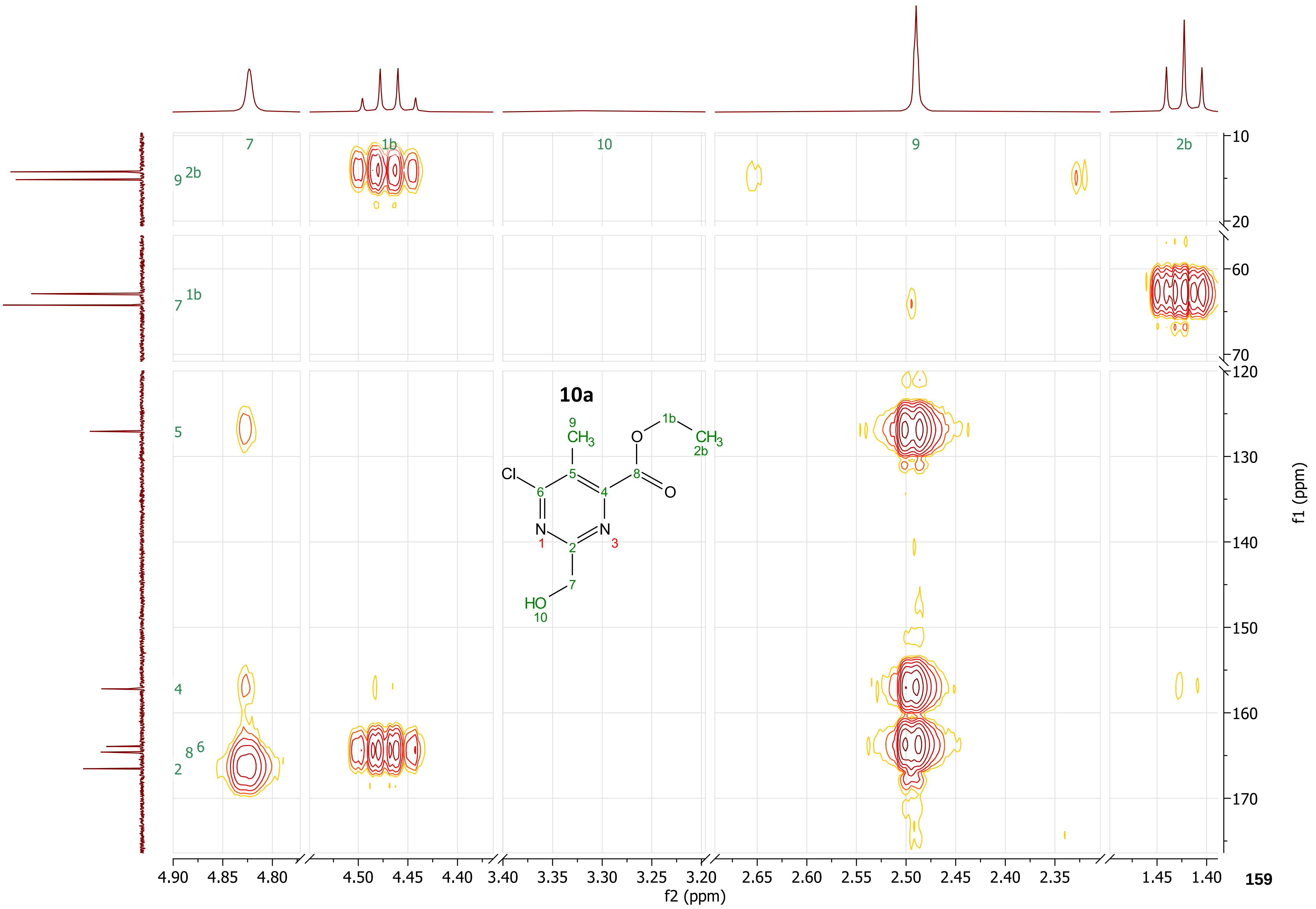

1H ^{1}H NMR (400 MHz, $CDCl_3$) δ 4.82 (app d, *J* = 0.8 Hz, 2H), 4.41 (t, *J* = 6.7 Hz, 2H), 3.28 (br s, 1H), 2.49 (app t, *J* = 0.8 Hz, 3H), 1.83 – 1.71 (m, 2H), 1.52 – 1.39 (m, 2H), 0.97 (t, *J* = 7.4 Hz, 3H).

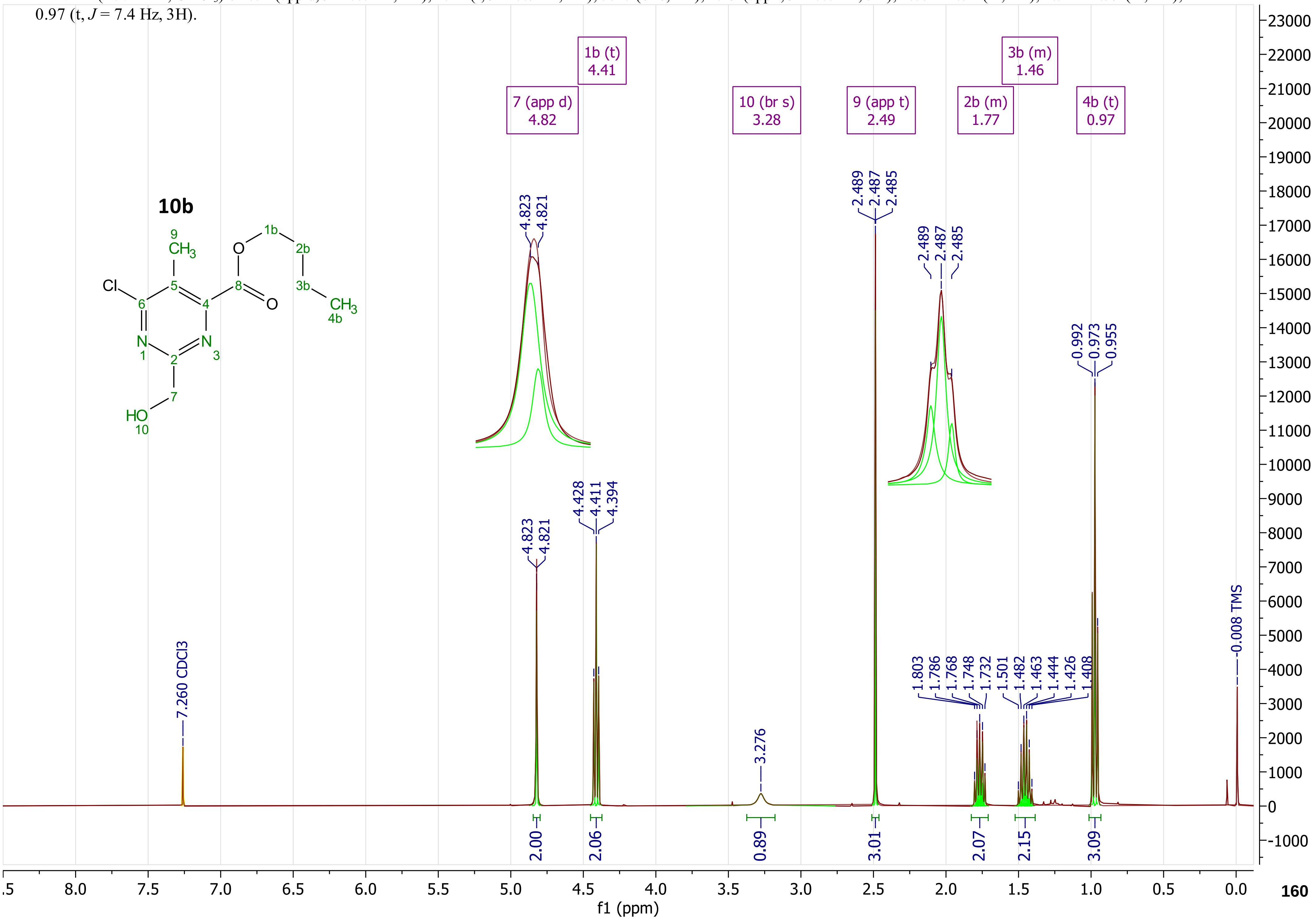

13C ^{13}C NMR (101 MHz, $CDCl_3$) δ 166.5, 164.7, 163.9, 157.3, 127.0, 66.7, 64.2, 30.6, 19.2, 15.2, 13.8.

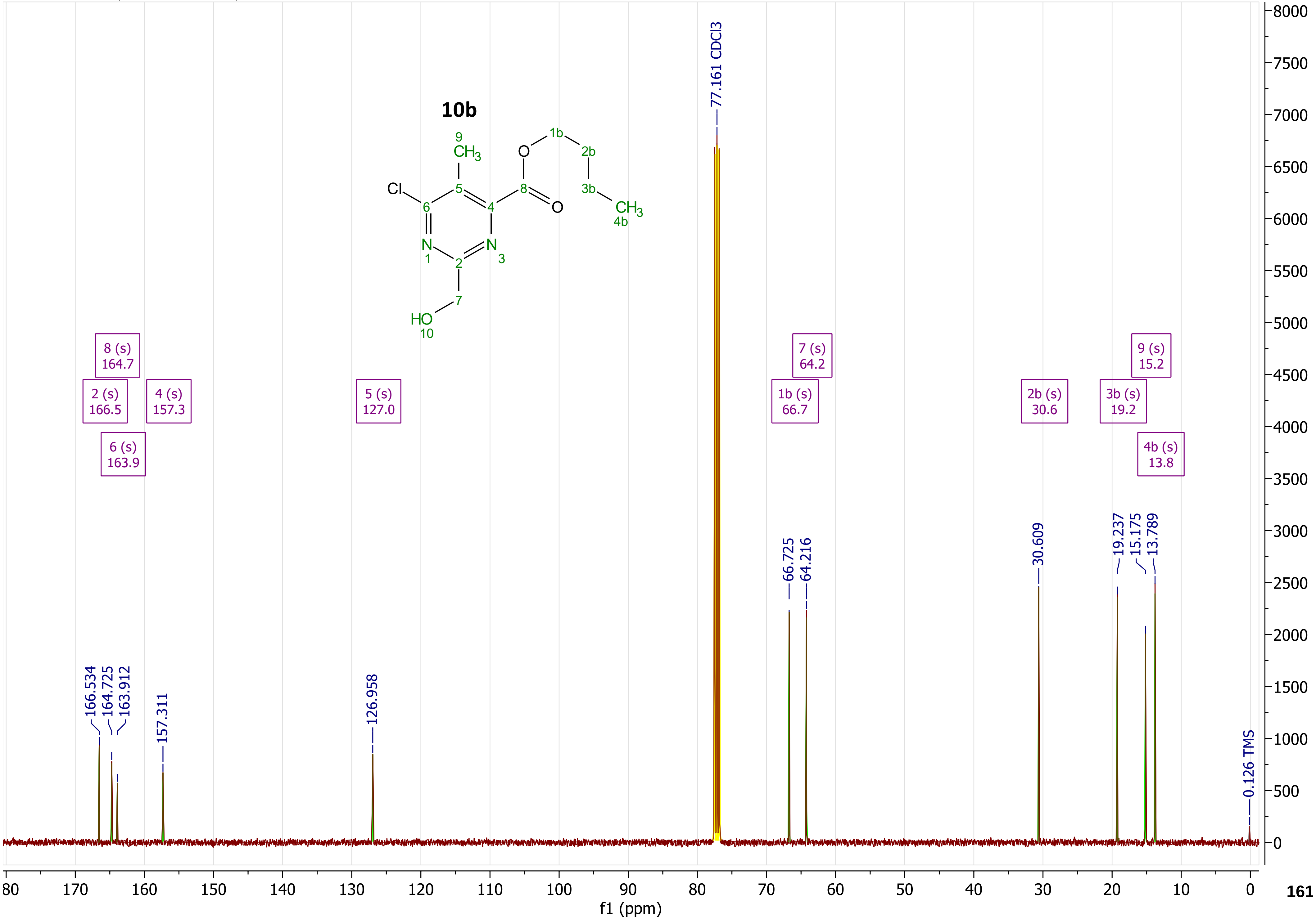

13C HSQC

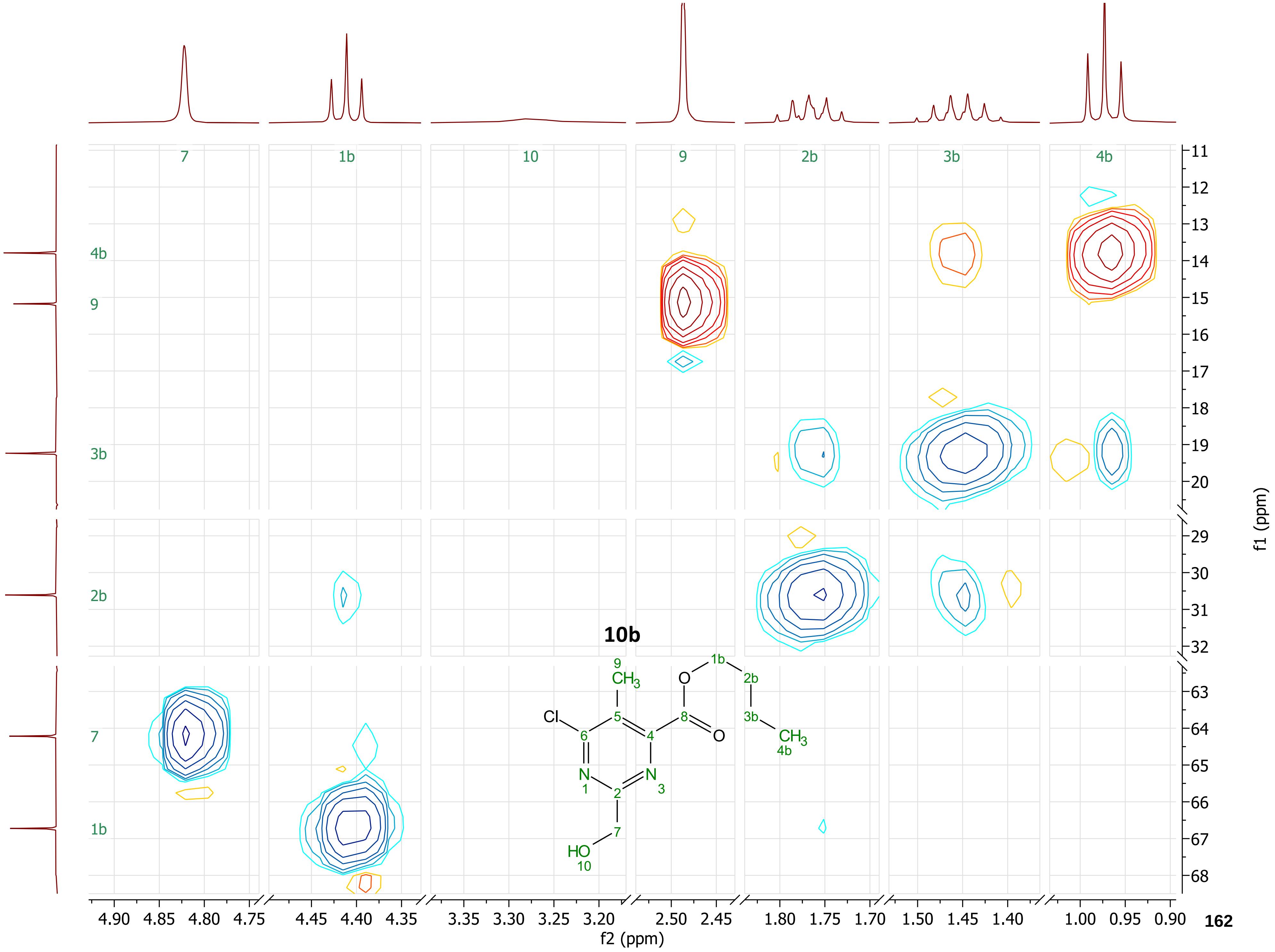
7
1b
10
9
2b
3b
4b
4b
9
3b
2b
7
1b
10b
f2 (ppm)
f1 (ppm)
4.90 4.85 4.80 4.75
4.45 4.40 4.35
3.35 3.30 3.25 3.20
2.50 2.45
1.80 1.75 1.70
1.50 1.45 1.40
1.00 0.95 0.90
11 12 13 14 15 16 17 18 19 20
29 30 31 32
63 64 65 66 67 68
Cl
CH_3
O
O
N
N
HO
CH_3

13C HMBC

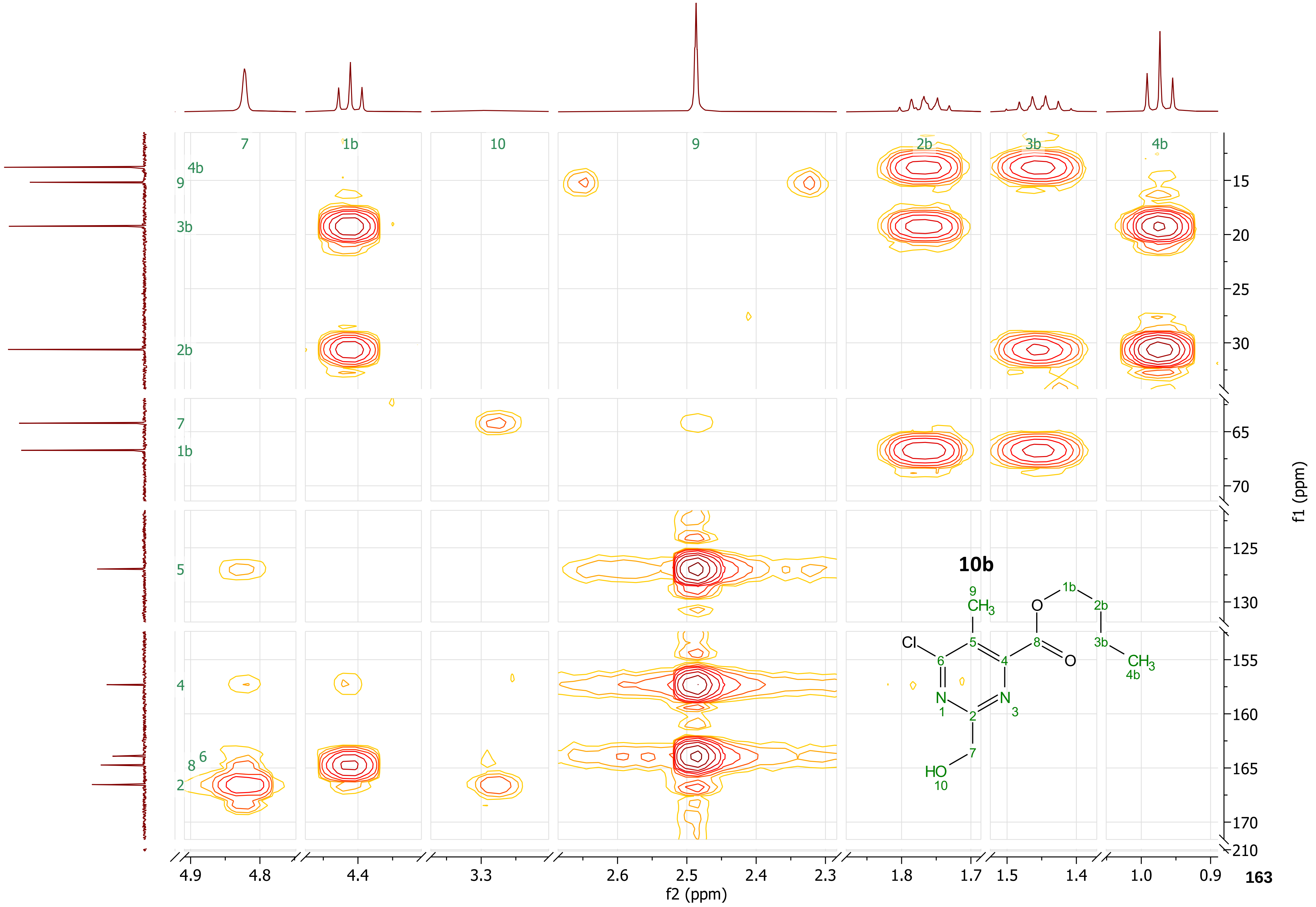

15N HMBC

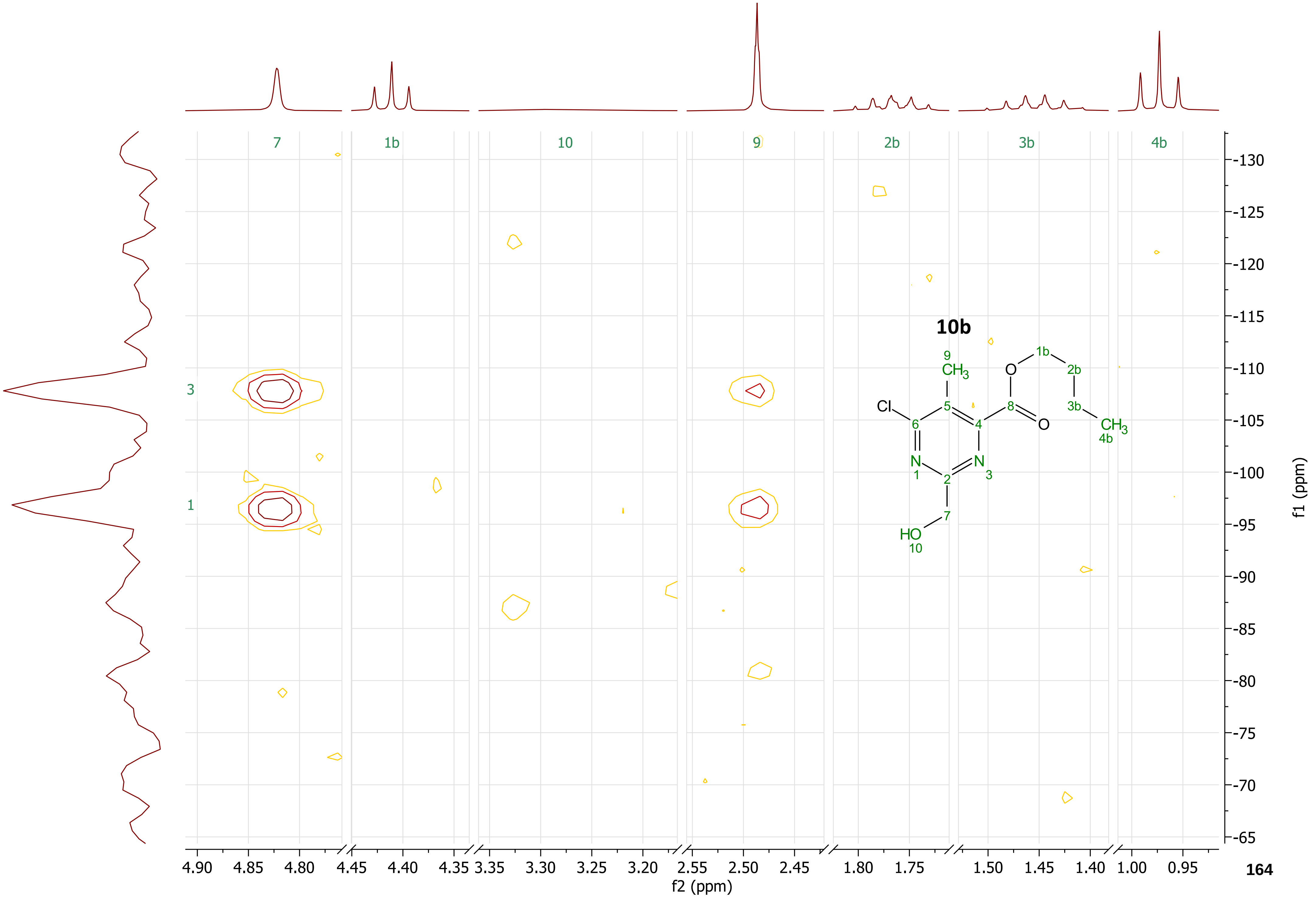

15N HMBC - f1 internal projection ^{15}N NMR (41 MHz, $CDCl_3$) δ -96.91, -107.99.

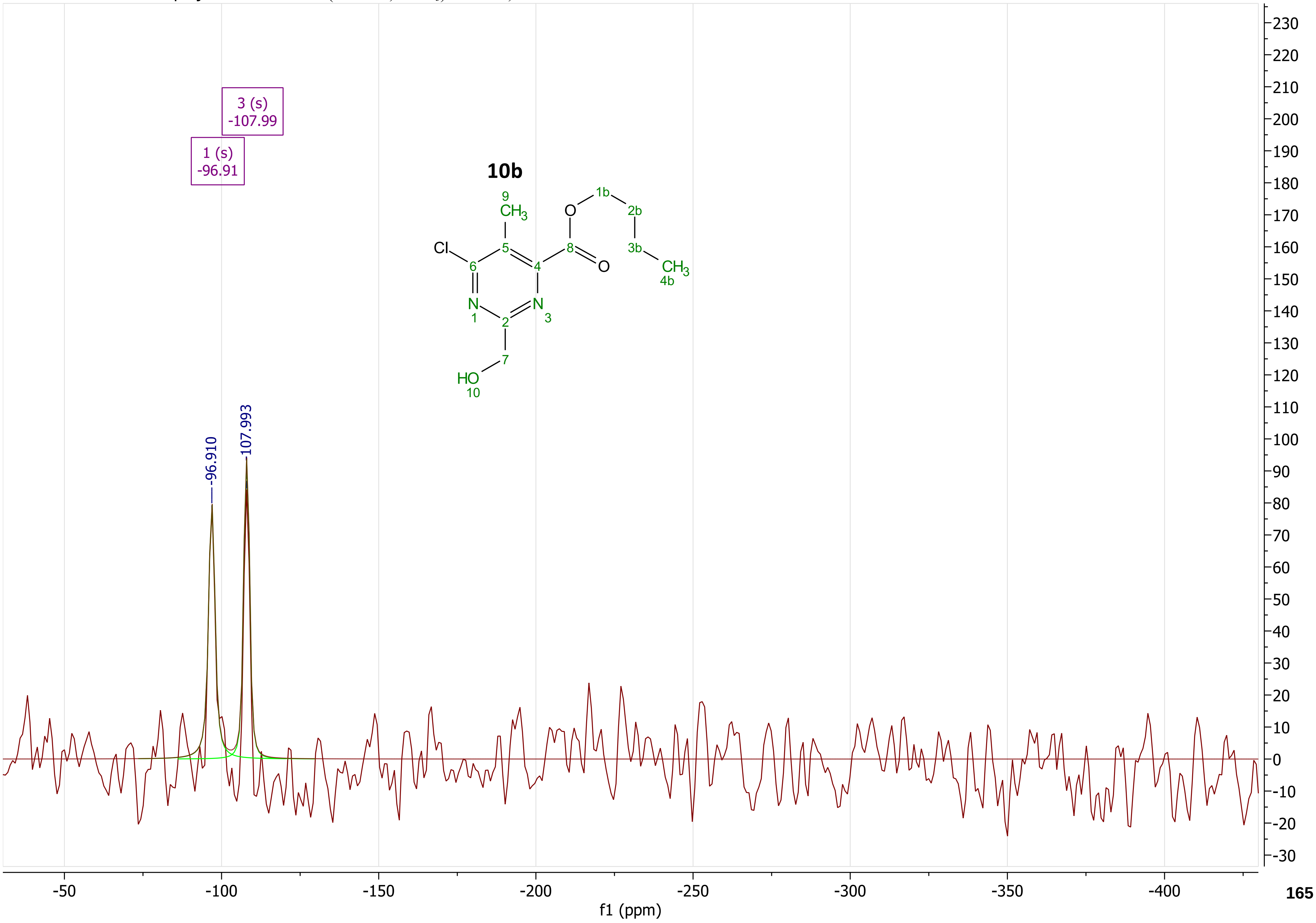

1H ^{1}H NMR (400 MHz, $CDCl_3$) δ 4.82 (d, J = 0.8 Hz, 2H), 4.40 (t, J = 6.8 Hz, 2H), 3.13 (br s, 1H), 2.48 (app t, J = 0.8 Hz, 3H), 1.83 – 1.72 (m, 2H), 1.46 – 1.37 (m, 2H), 1.37 – 1.23 (m, 6H), 0.88 (app t, J = 7.0 Hz, 3H).

10c

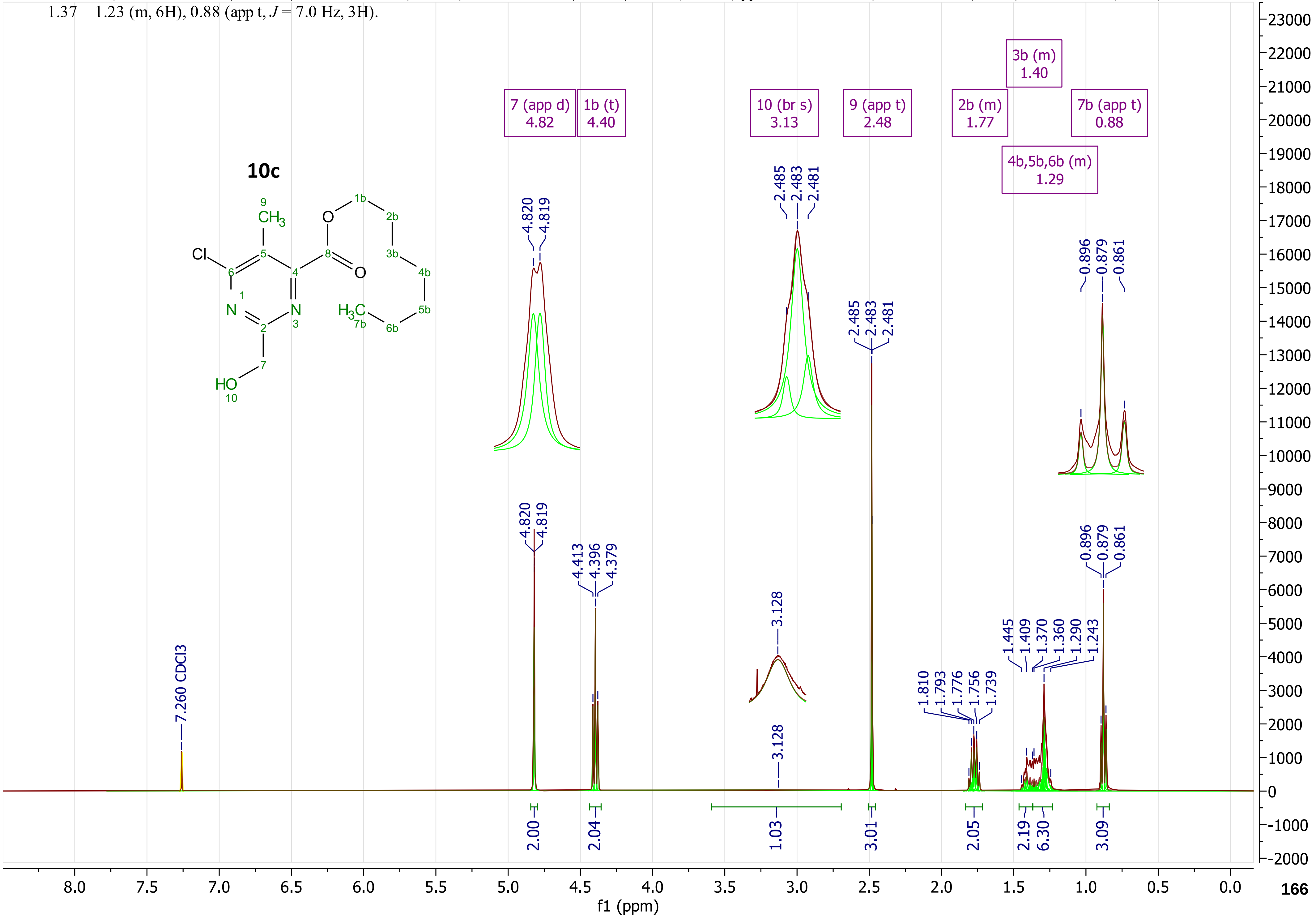

13C ^{13}C NMR (101 MHz, $CDCl_3$) δ 166.5, 164.7, 163.9, 157.3, 127.0, 67.0, 64.2, 31.8, 29.0, 28.6, 25.9, 22.7, 15.2, 14.2.

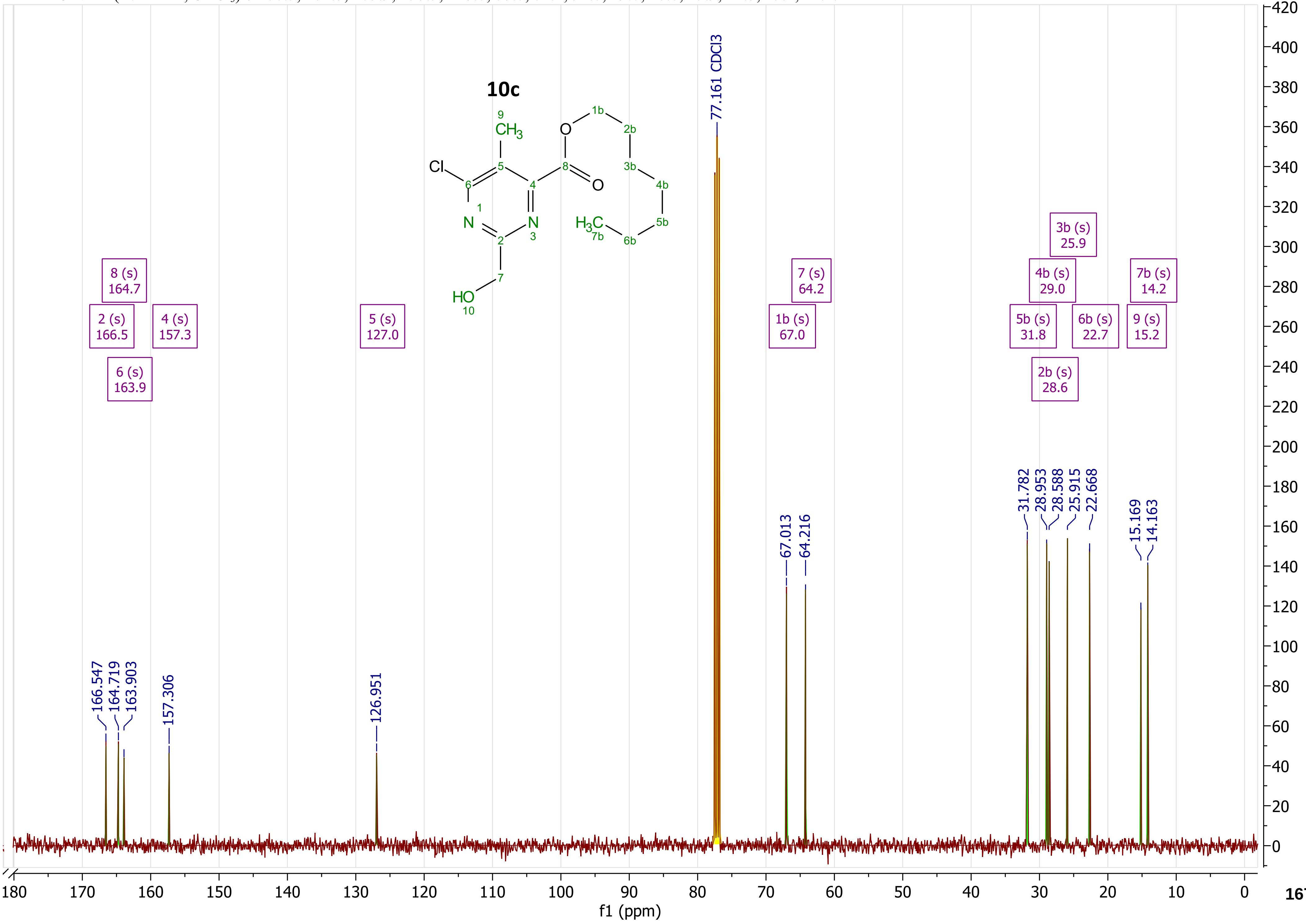

13C HSQC

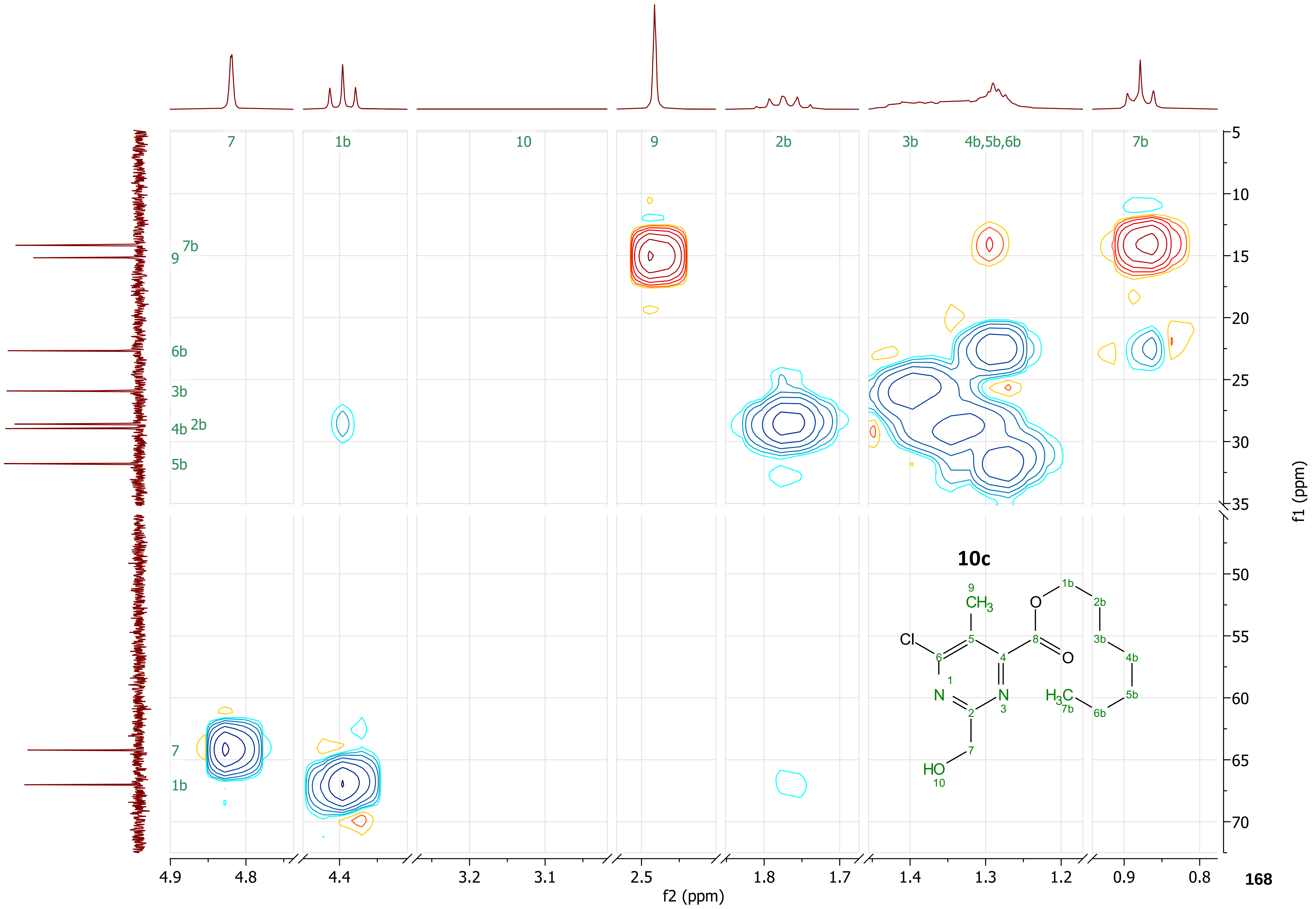
7
1b
10
9
2b
3b
4b,5b,6b
7b
9 7b
6b
3b
4b 2b
5b
7
1b
10c
Cl
CH3
O
O
N
N
HO
H3C
f1 (ppm)
f2 (ppm)

13C HMBC

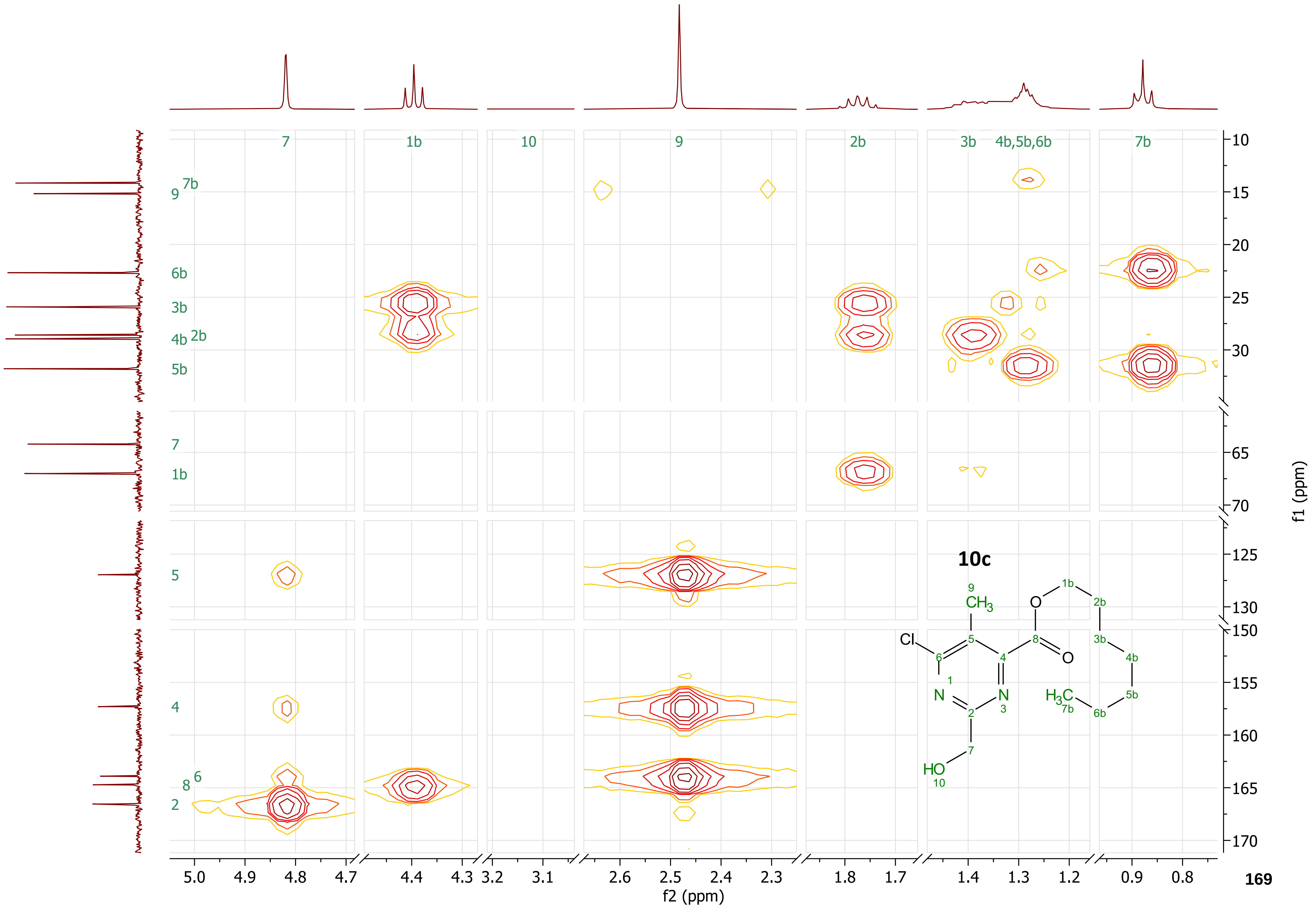
7
1b
10
9
2b
3b
4b,5b,6b
7b
9
7b
6b
3b
4b
2b
5b
7
1b
5
4
8
6
2
10c
9
CH3
O
1b
2b
Cl
5
8
3b
6
4
O
4b
1
N
N
3
H3C
7b
5b
2
6b
7
HO
10
f2 (ppm)
f1 (ppm)

15N HMBC

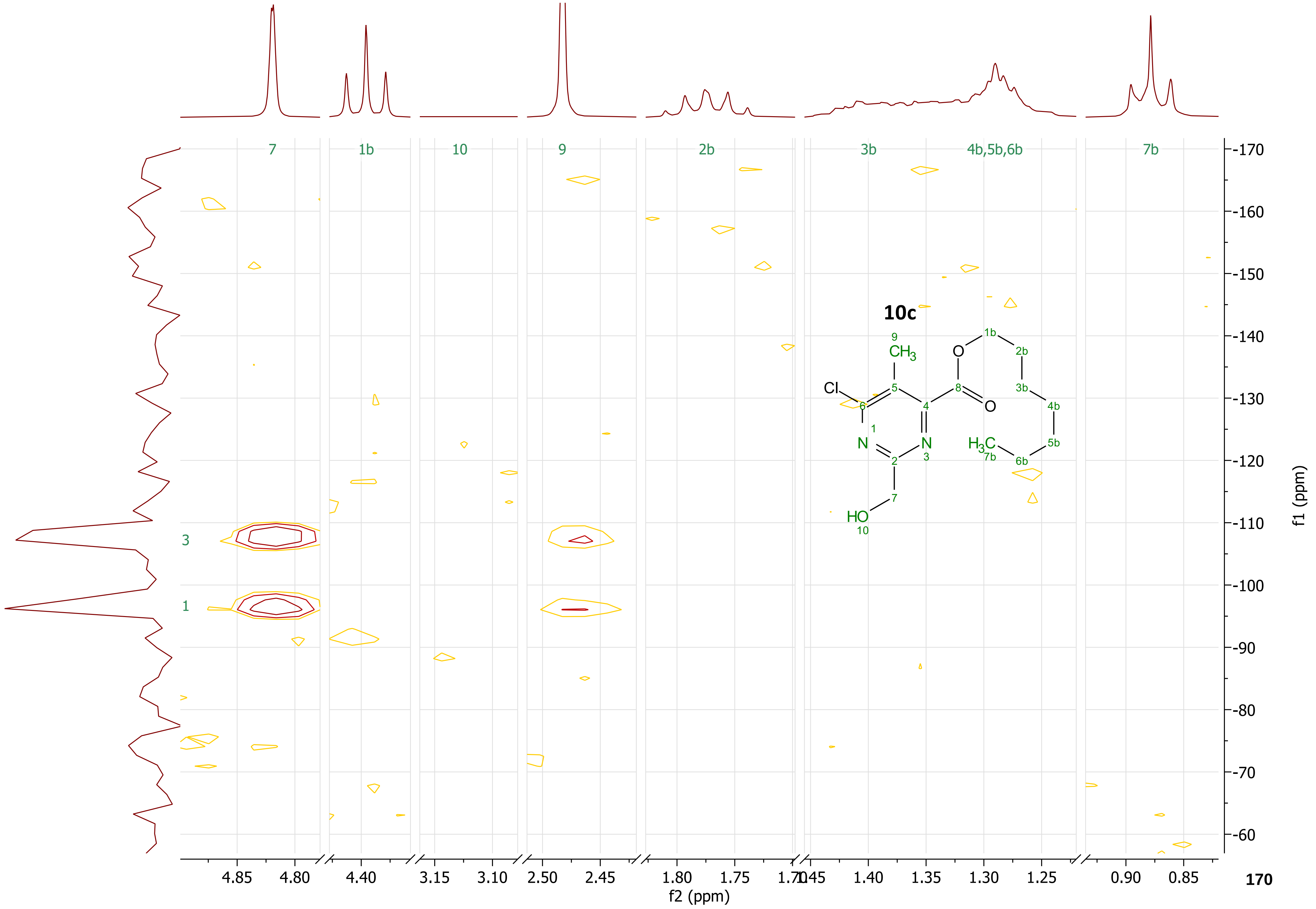

15N HMBC - f1 internal projection ^{15}N NMR (41 MHz, $CDCl_3$) δ -96.73, -107.28.

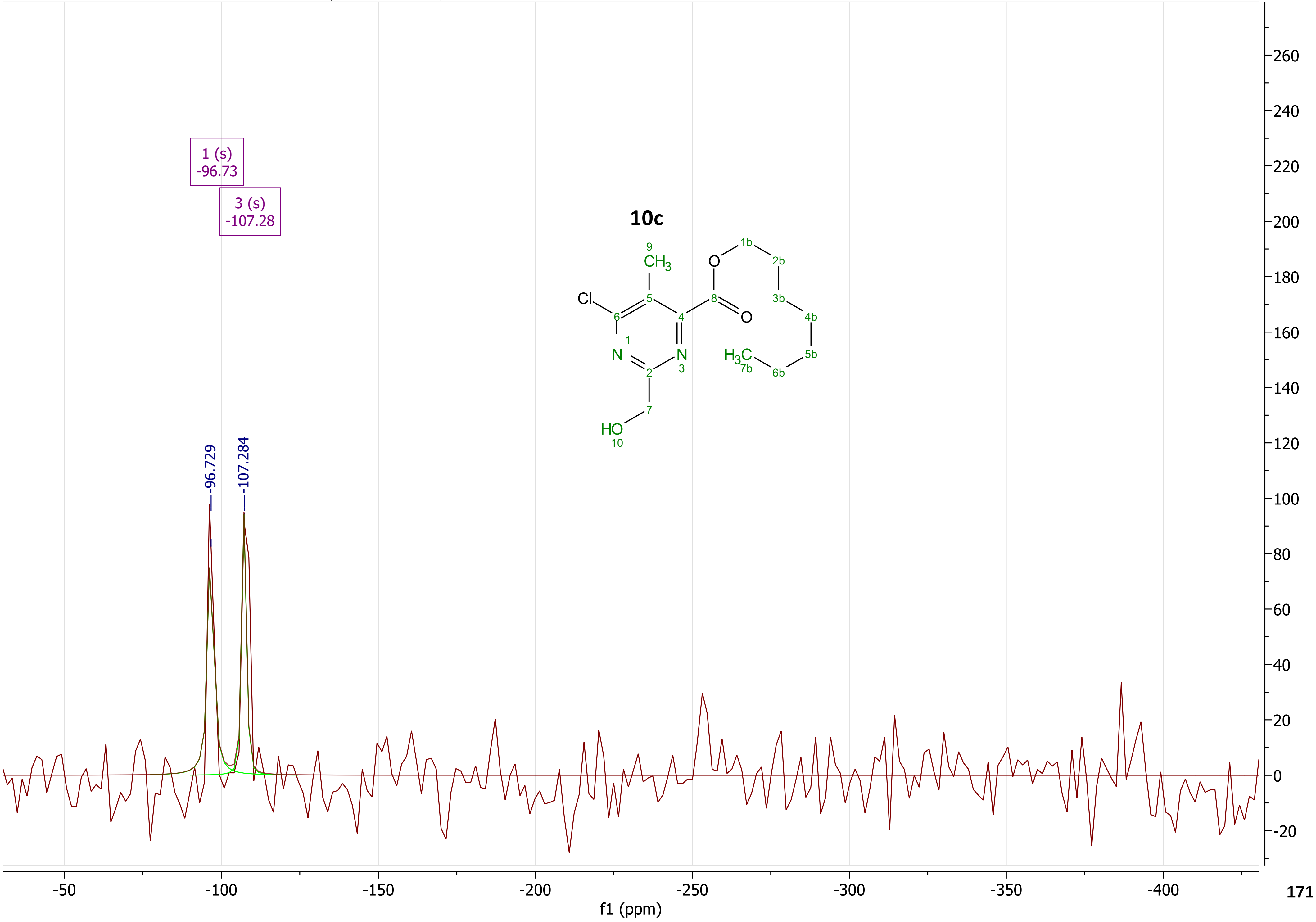

1H ^{1}H NMR (400 MHz, $CDCl_3$) δ 4.82 (app q, J = 0.8 Hz, 2H), 4.40 (t, J = 6.8 Hz, 2H), 3.12 (br s, 1H), 2.49 (app t, J = 0.8 Hz, 3H), 1.84 – 1.72 (m, 2H), 1.47 – 1.37 (m, 2H), 1.36 – 1.21 (m, 8H), 0.88 (app t, J = 7.0 Hz, 3H).

10d

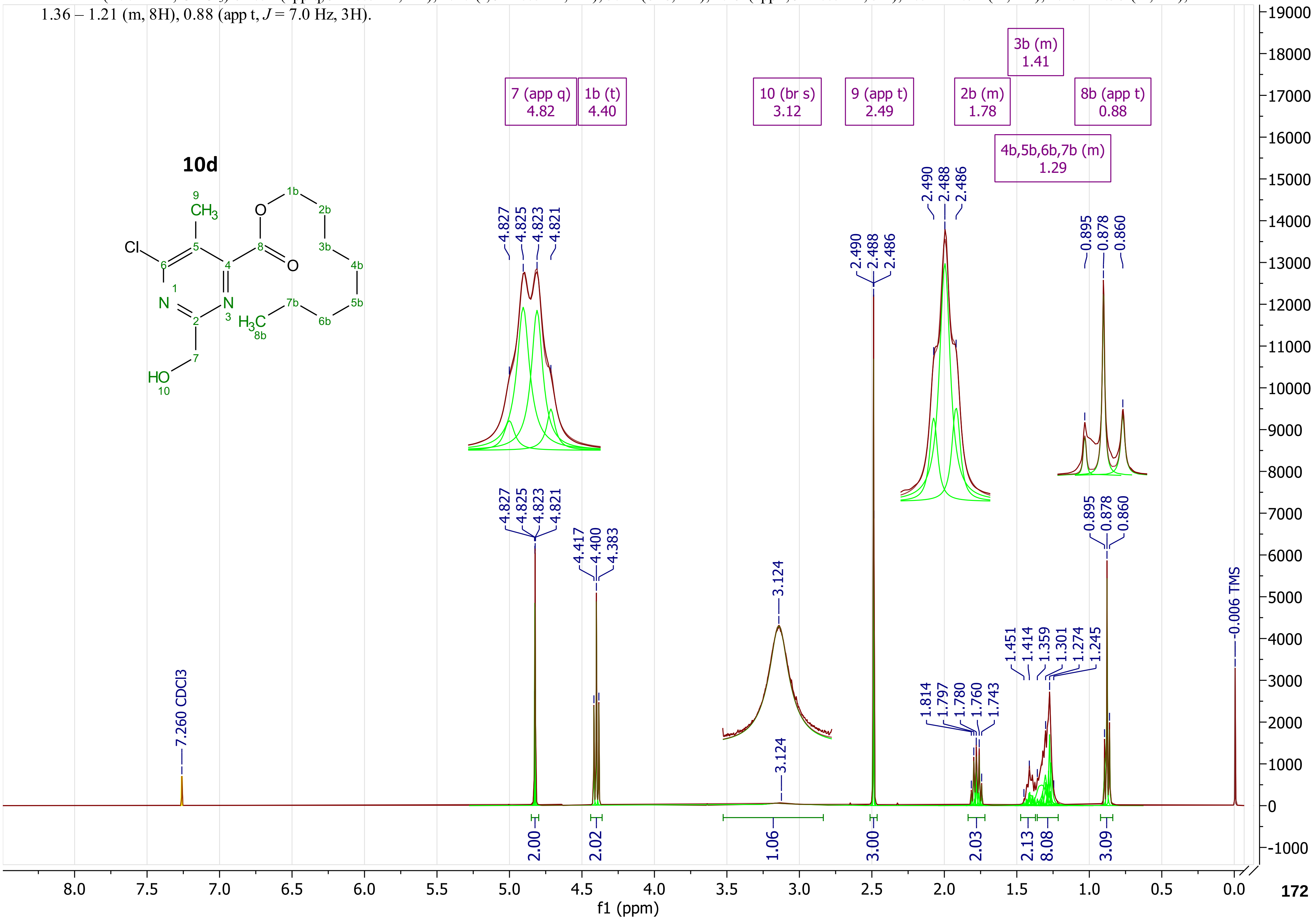

13C ^{13}C NMR (101 MHz, $CDCl_3$) δ 166.5, 164.7, 163.9, 157.3, 127.0, 67.0, 64.2, 31.9, 29.3 (2C), 28.6, 26.0, 22.8, 15.2, 14.2.

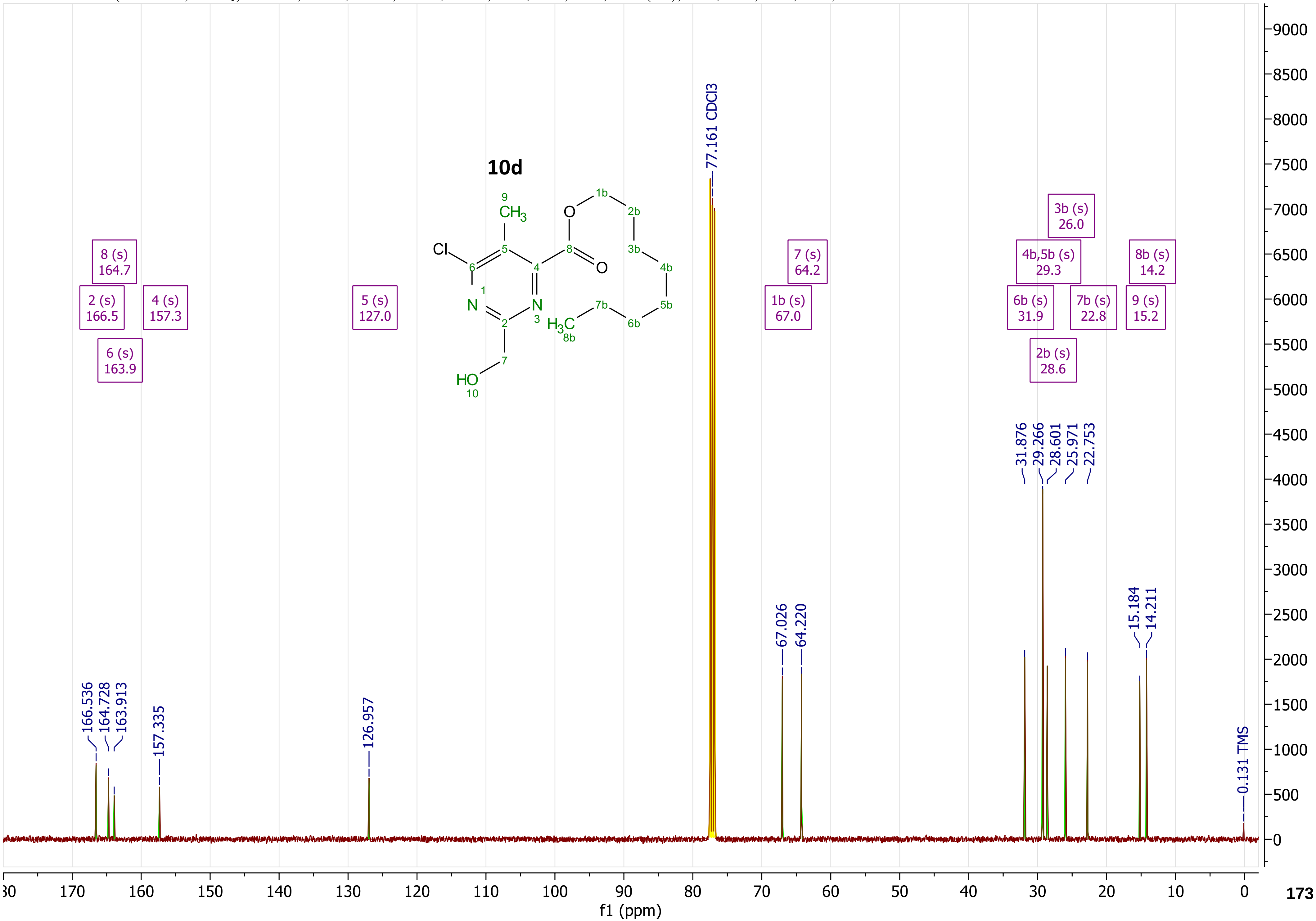

13C HSQC

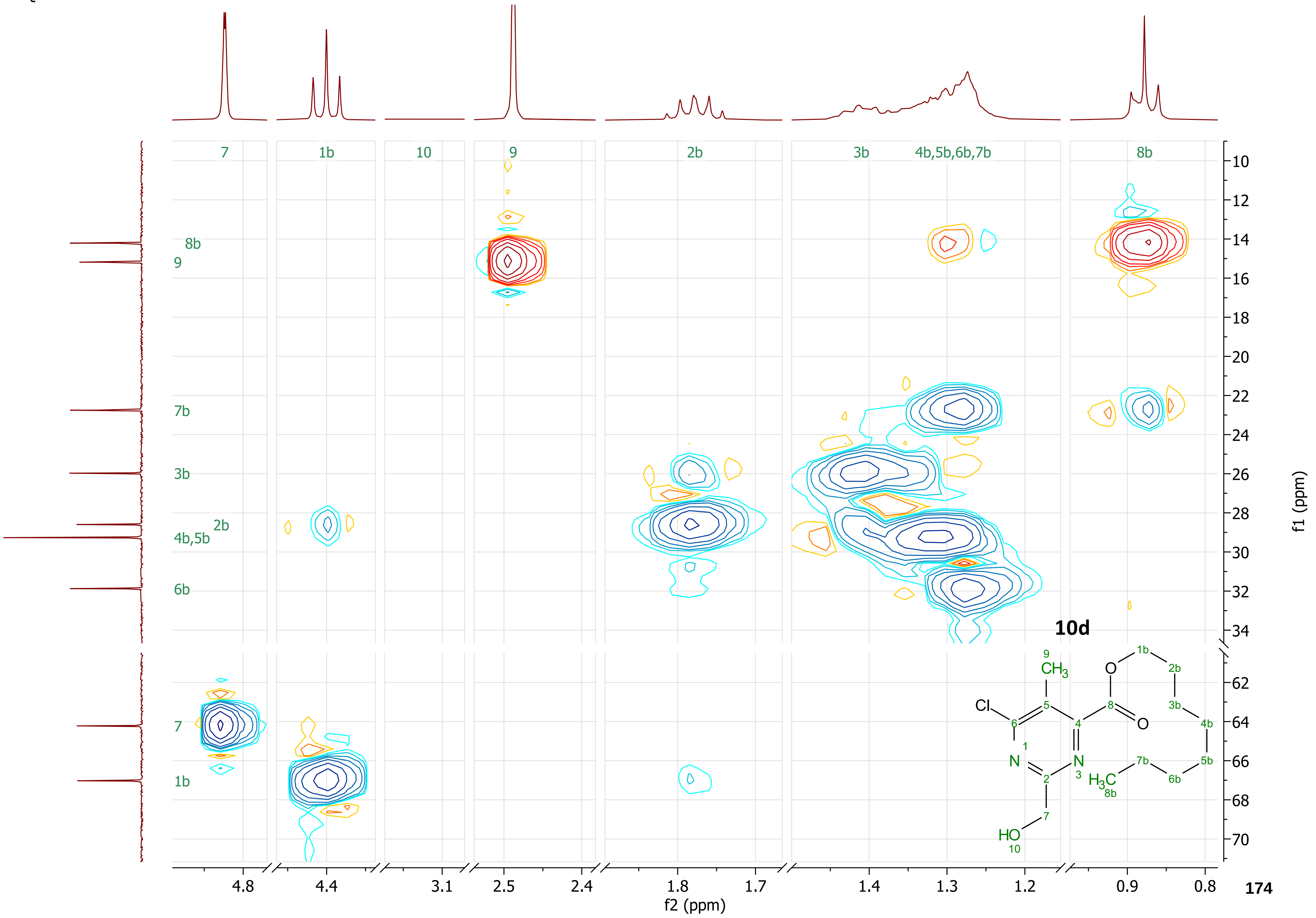
7
1b
10
9
2b
3b
4b,5b,6b,7b
8b
8b
9
7b
3b
2b
4b,5b
6b
7
1b
10d
9
CH3
Cl
O
1b
2b
3b
4b
5b
6b
7b
8b
H3C
O
N
N
HO
10
f2 (ppm)
f1 (ppm)
4.8
4.4
3.1
2.5
2.4
1.8
1.7
1.4
1.3
1.2
0.9
0.8
10
12
14
16
18
20
22
24
26
28
30
32
34
62
64
66
68
70

13C HMBC

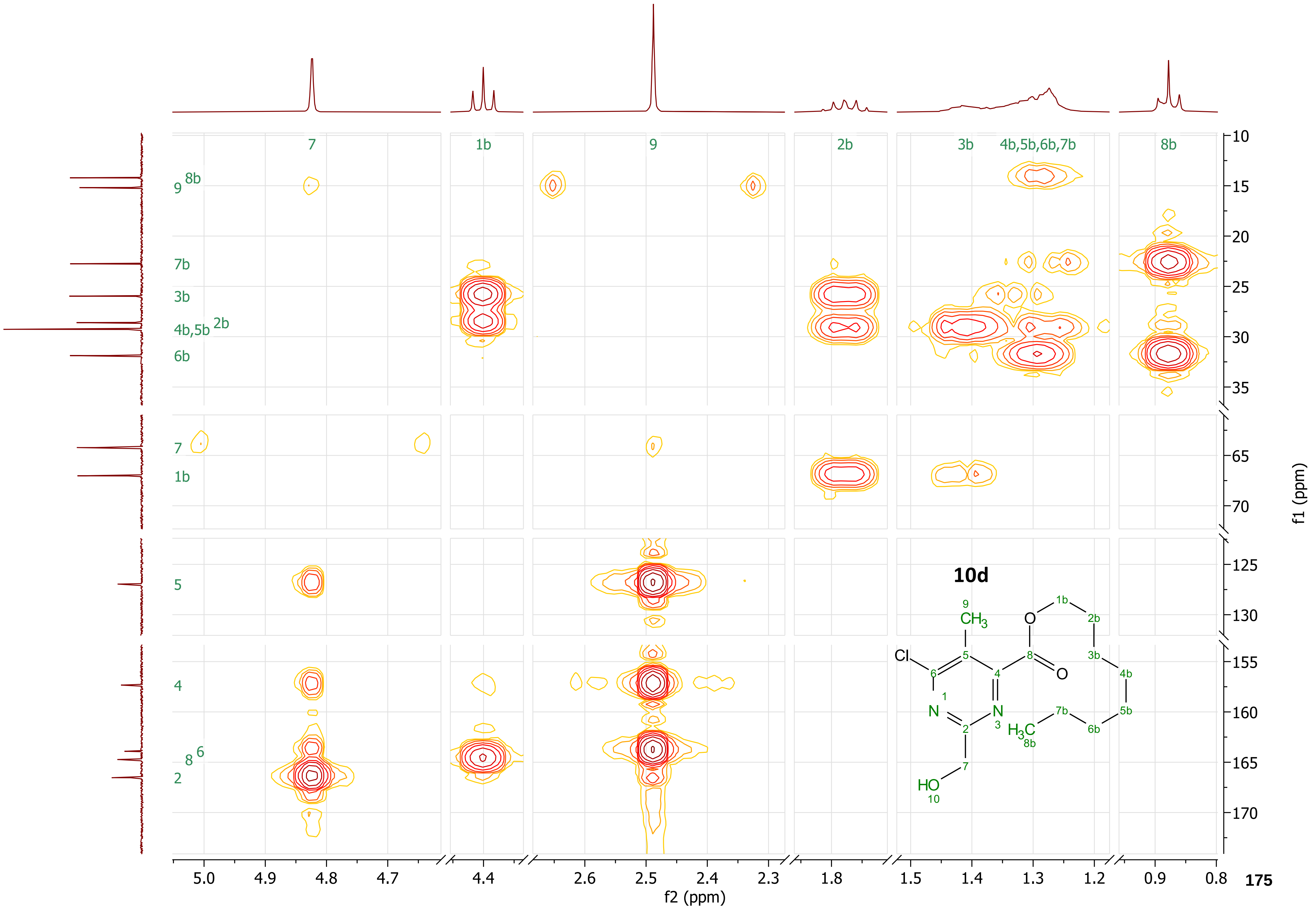

7
1b
9
2b
3b
4b,5b,6b,7b
8b
9
8b
7b
3b
4b,5b
2b
6b
7
1b
5
4
8
6
2
10d
CH3
O
1b
2b
Cl
5
8
3b
O
6
4
4b
N
N
1
3
2
7b
5b
H3C
8b
6b
7
HO
10
5.0
4.9
4.8
4.7
4.4
2.6
2.5
2.4
2.3
1.8
1.5
1.4
1.3
1.2
0.9
0.8
f2 (ppm)
f1 (ppm)
10
15
20
25
30
35
65
70
125
130
155
160
165
170

15N HMBC

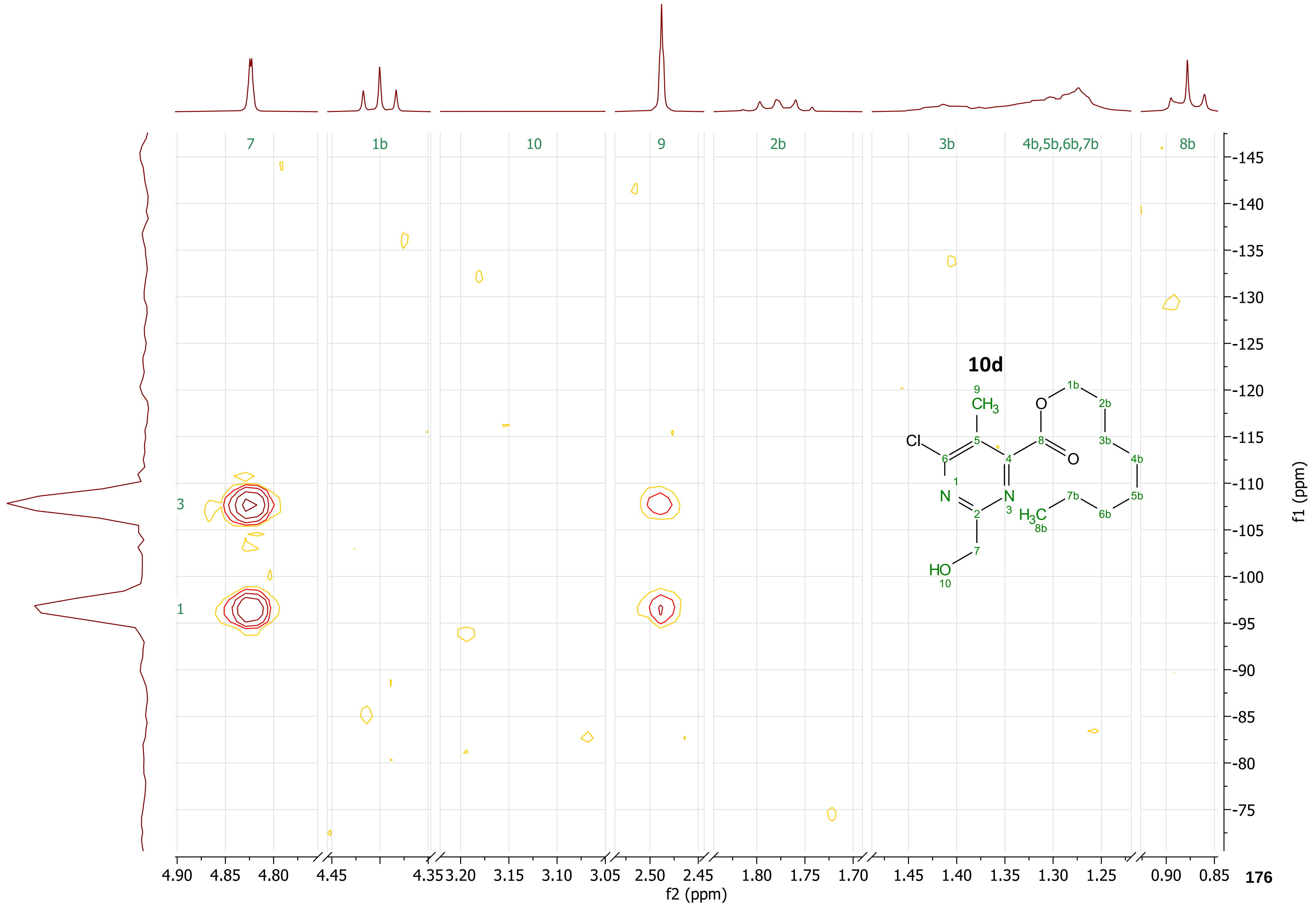

15N HMBC - f1 internal projection ^{15}N NMR (41 MHz, $CDCl_3$) δ -96.60, -107.83.

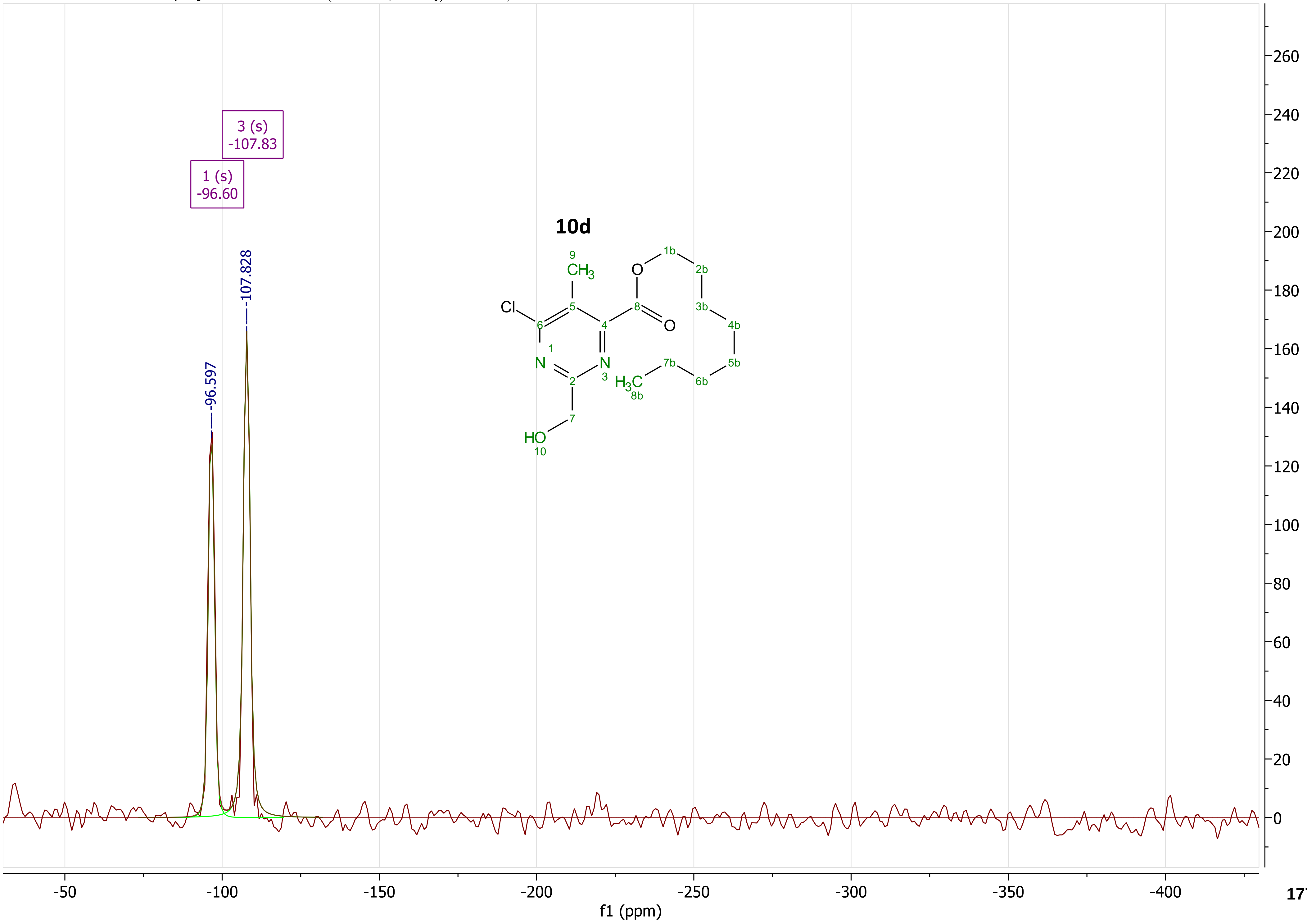

1H ^{1}H NMR (400 MHz, $CDCl_3$) δ 4.83 (app d, J = 3.4 Hz, 2H), 4.40 (t, J = 6.8 Hz, 2H), 3.25 (app t, J = 5.0 Hz, 1H), 2.49 (app t, J = 0.8 Hz, 3H), 1.84 – 1.72 (m, 2H), 1.47 – 1.36 (m, 2H), 1.38 – 1.19 (m, 10H), 0.88 (app t, J = 7.0 Hz, 3H).

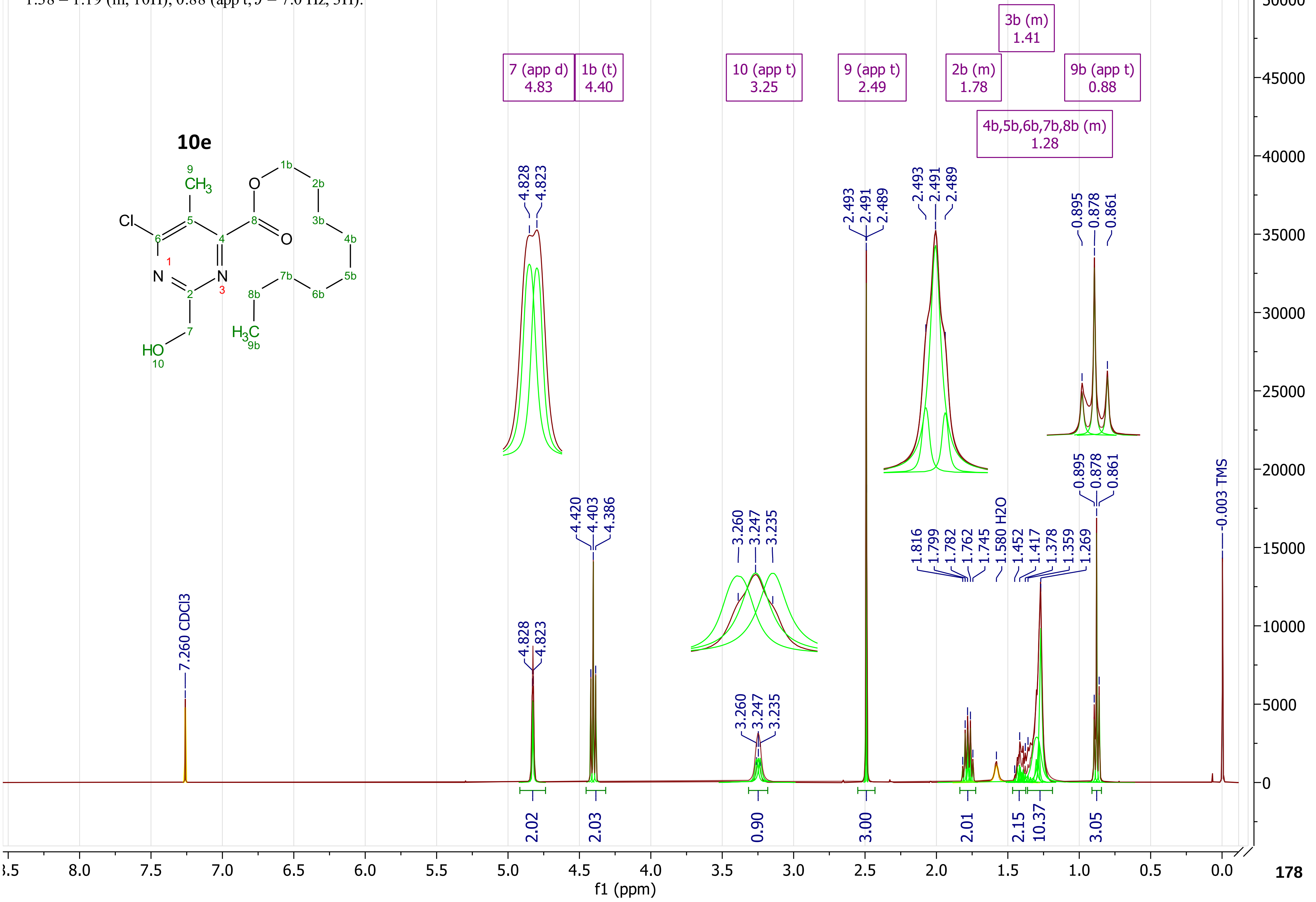

13C ^{13}C NMR (101 MHz, $CDCl_3$) δ 166.5, 164.7, 163.9, 157.3, 127.0, 67.0, 64.2, 32.0, 29.6, 29.34, 29.32, 28.6, 26.0, 22.8, 15.2, 14.2.

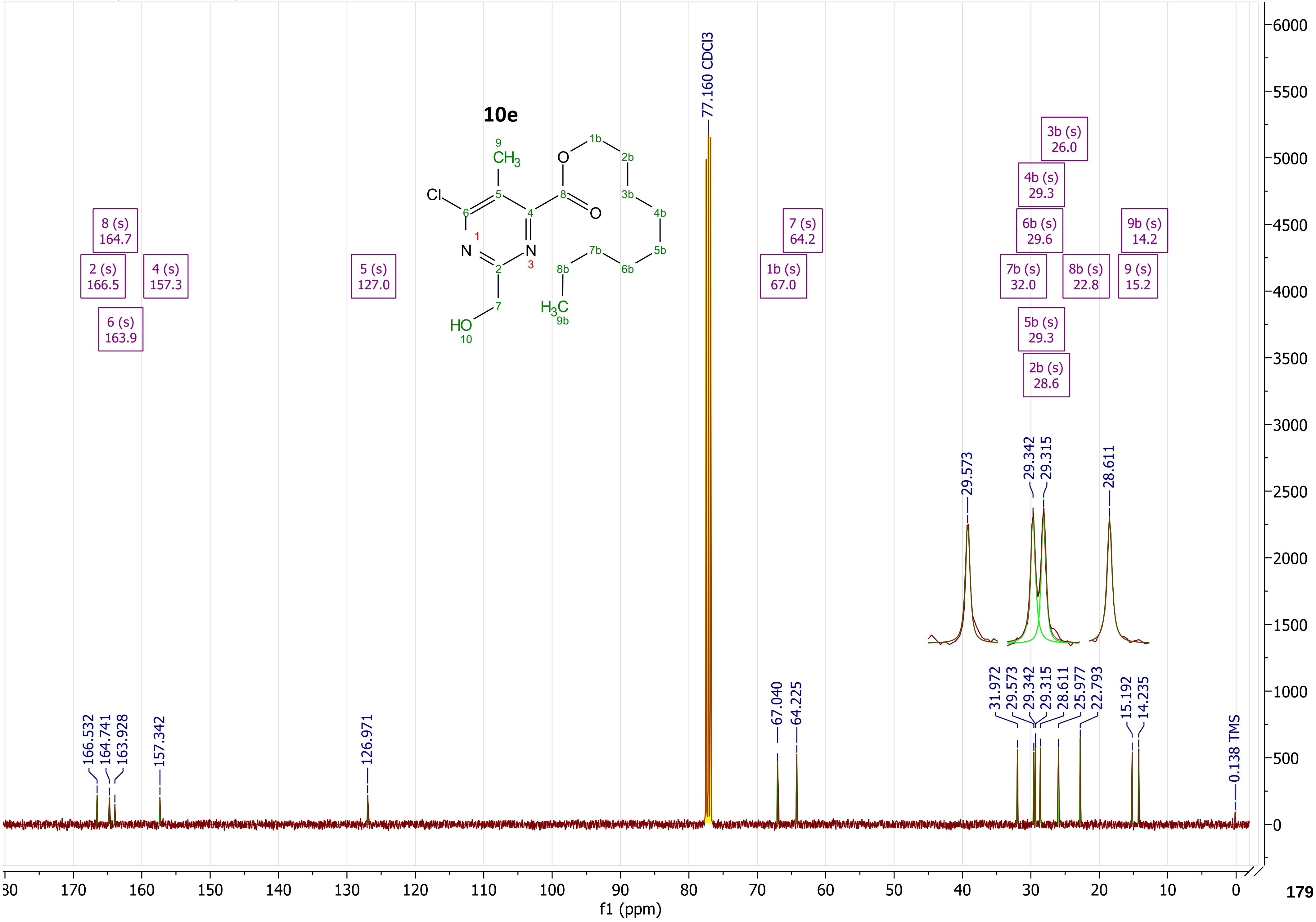

13C HSQC

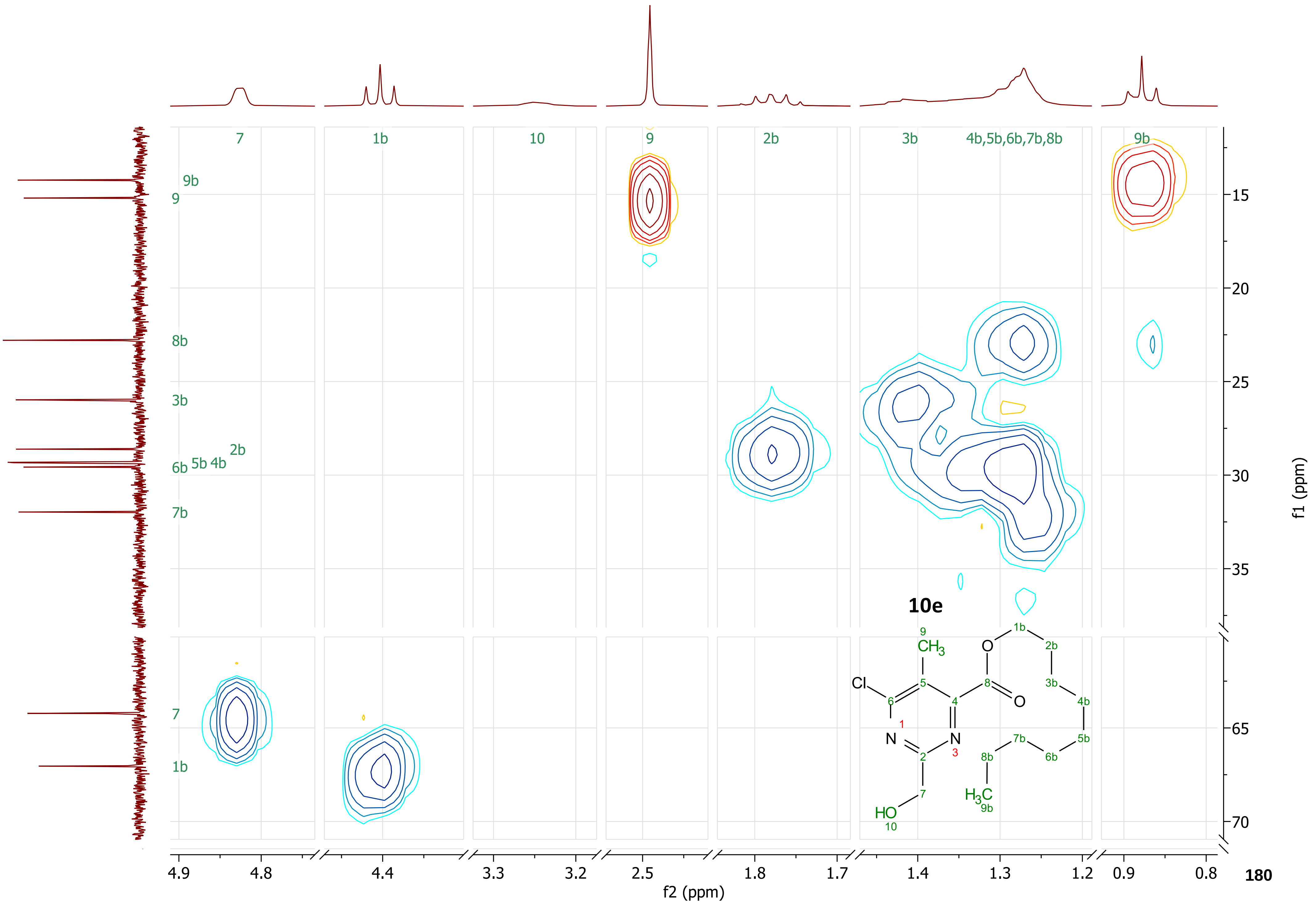

7
1b
10
9
2b
3b
4b,5b,6b,7b,8b
9b
9b
9
8b
3b
2b
6b 5b 4b
7b
7
1b
10e
f1 (ppm)
f2 (ppm)
4.9
4.8
4.4
3.3
3.2
2.5
1.8
1.7
1.4
1.3
1.2
0.9
0.8
15
20
25
30
35
65
70

13C HMBC

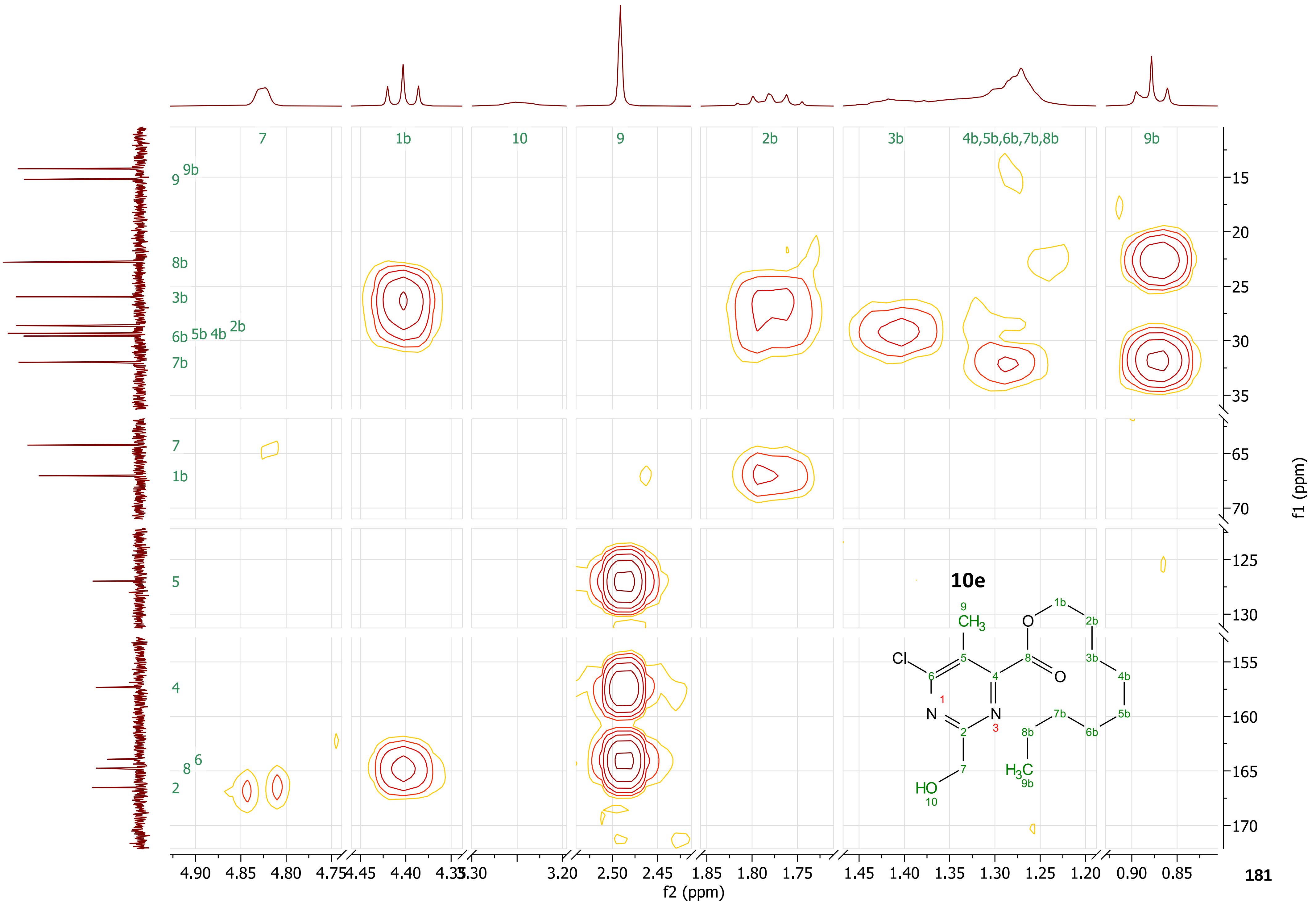

1H

^{1}H NMR (400 MHz, $CDCl_3$) δ 7.74 – 7.69 (m, 1H), 7.68 – 7.64 (m, 1H), 7.64 – 7.61 (m, 1H), 7.58 – 7.49 (m, 1H), 5.48 (s, 2H), 4.83 (app q, J = 0.8 Hz, 2H), 3.15 (br s, 1H), 2.48 (app t, J = 0.8 Hz, 3H).

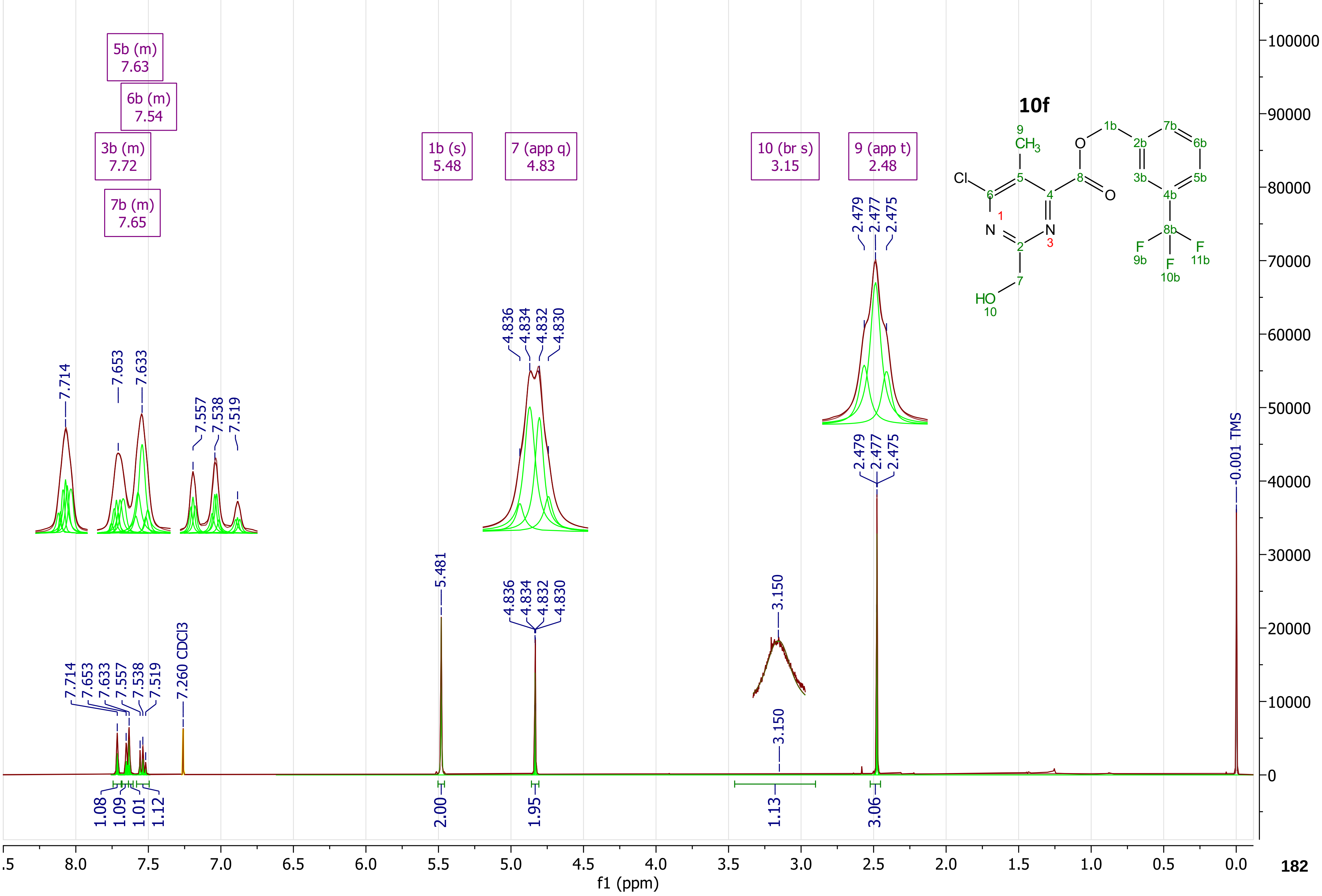

13C ^{13}C NMR (101 MHz, $CDCl_3$) δ 166.6, 164.24, 164.19, 156.3, 135.8, 131.8 (q, J = 1.1 Hz), 131.4 (q, J = 32.7 Hz), 129.5, 127.5, 125.8 (q, J = 3.7 Hz), 125.3 (q, J = 4.0 Hz), 124.0 (q, J = 272.4 Hz), 67.3, 64.2, 15.2.

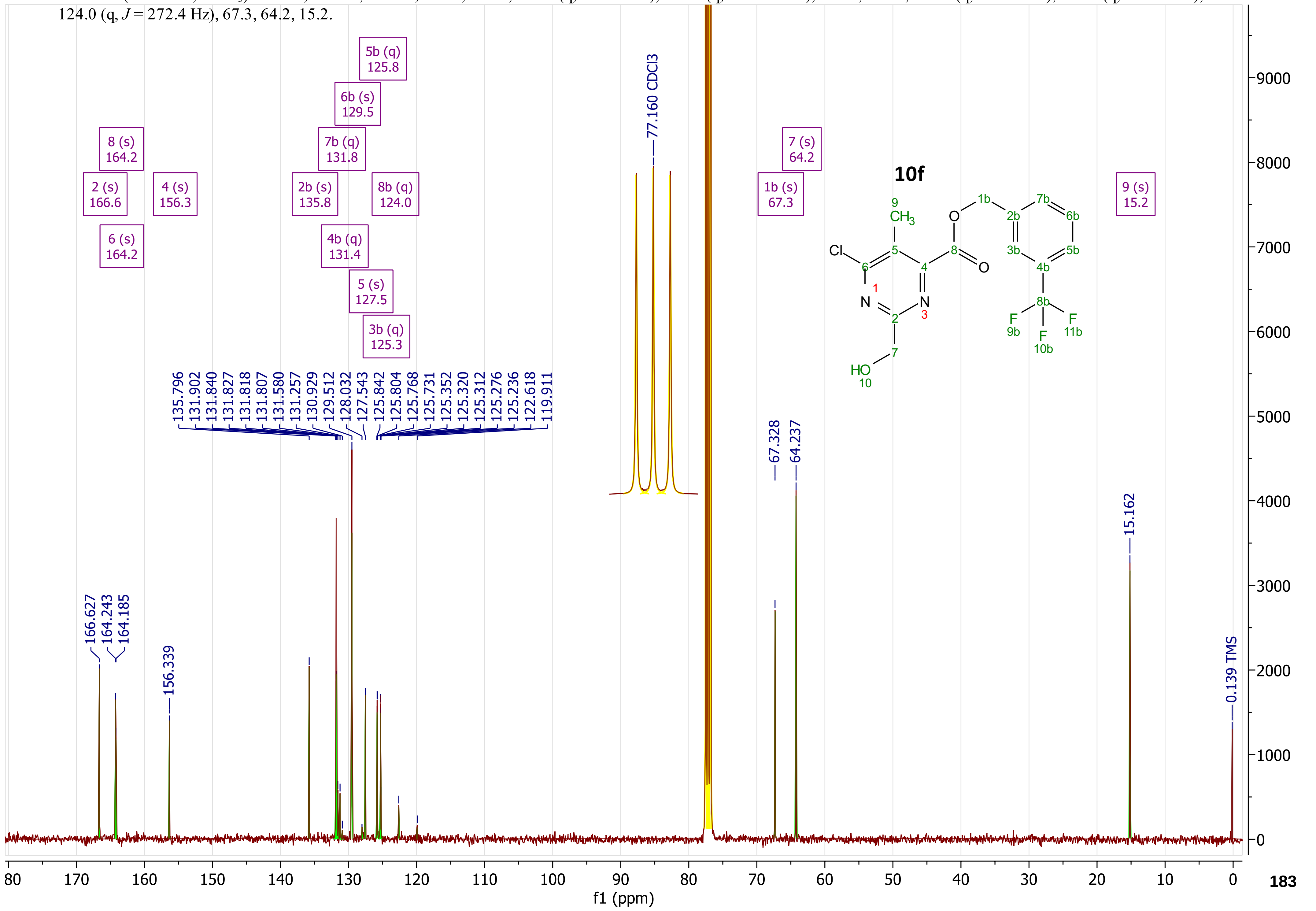

13C — 167.5–119.5 ppm

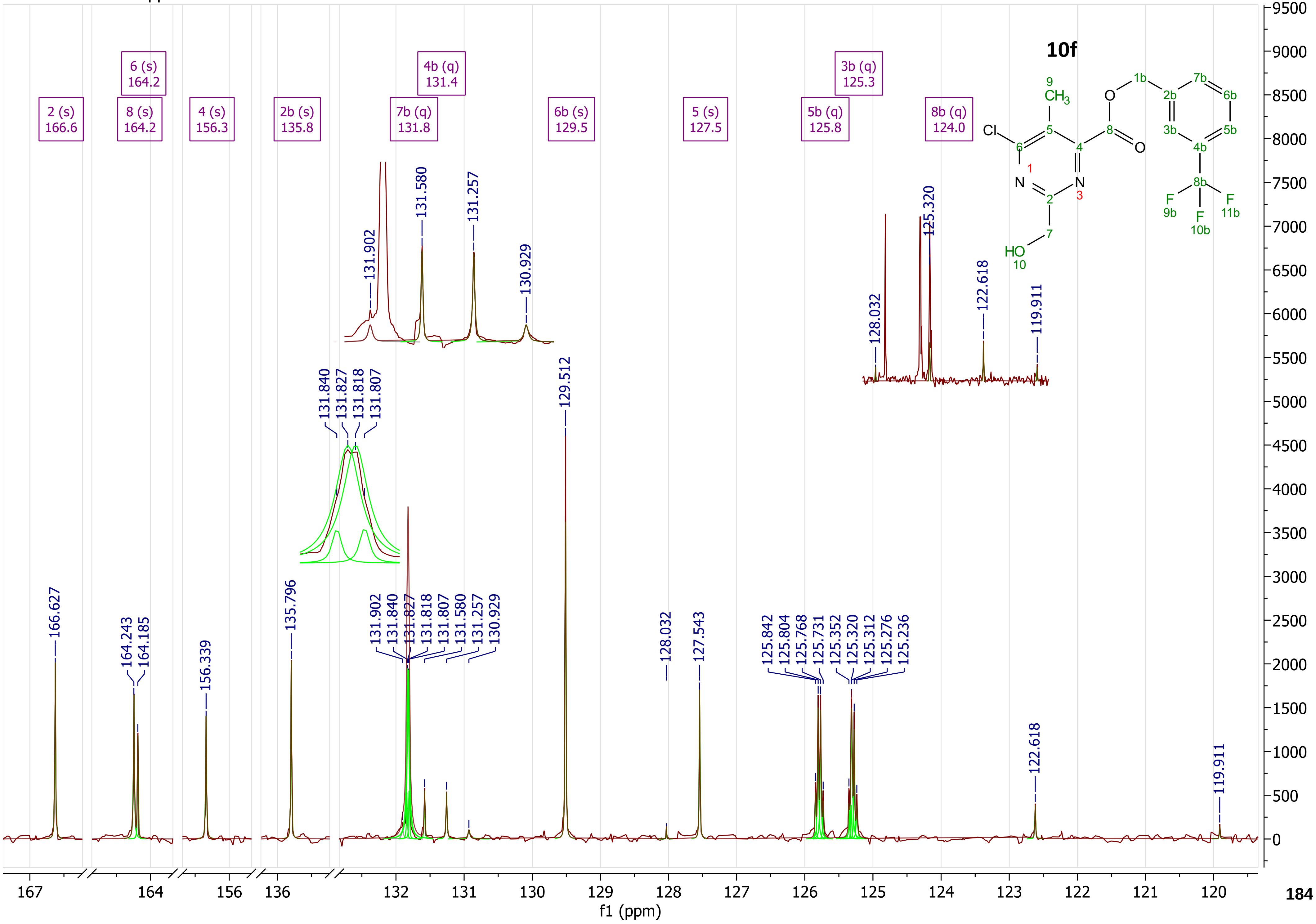

13C HSQC

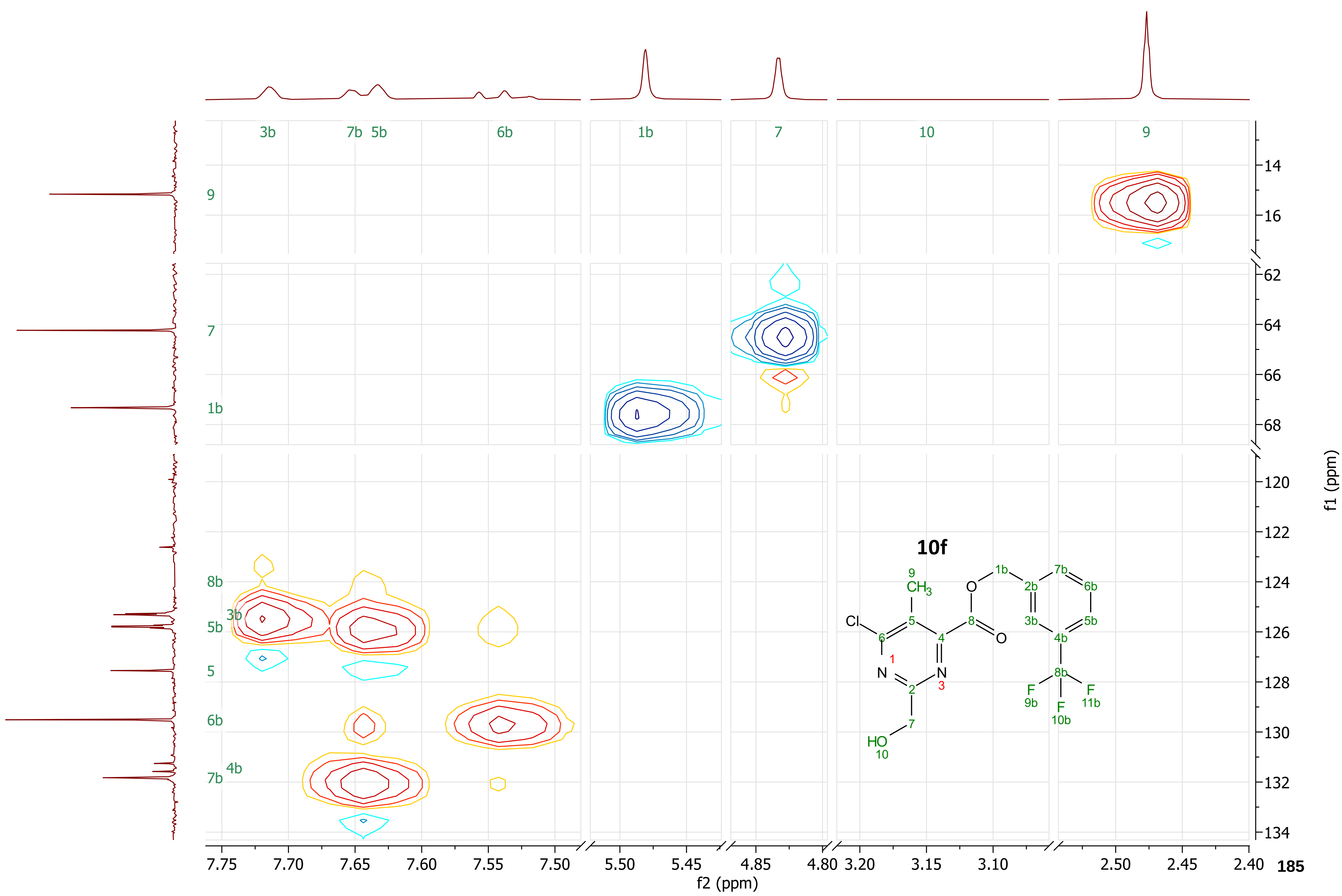

13C HMBC

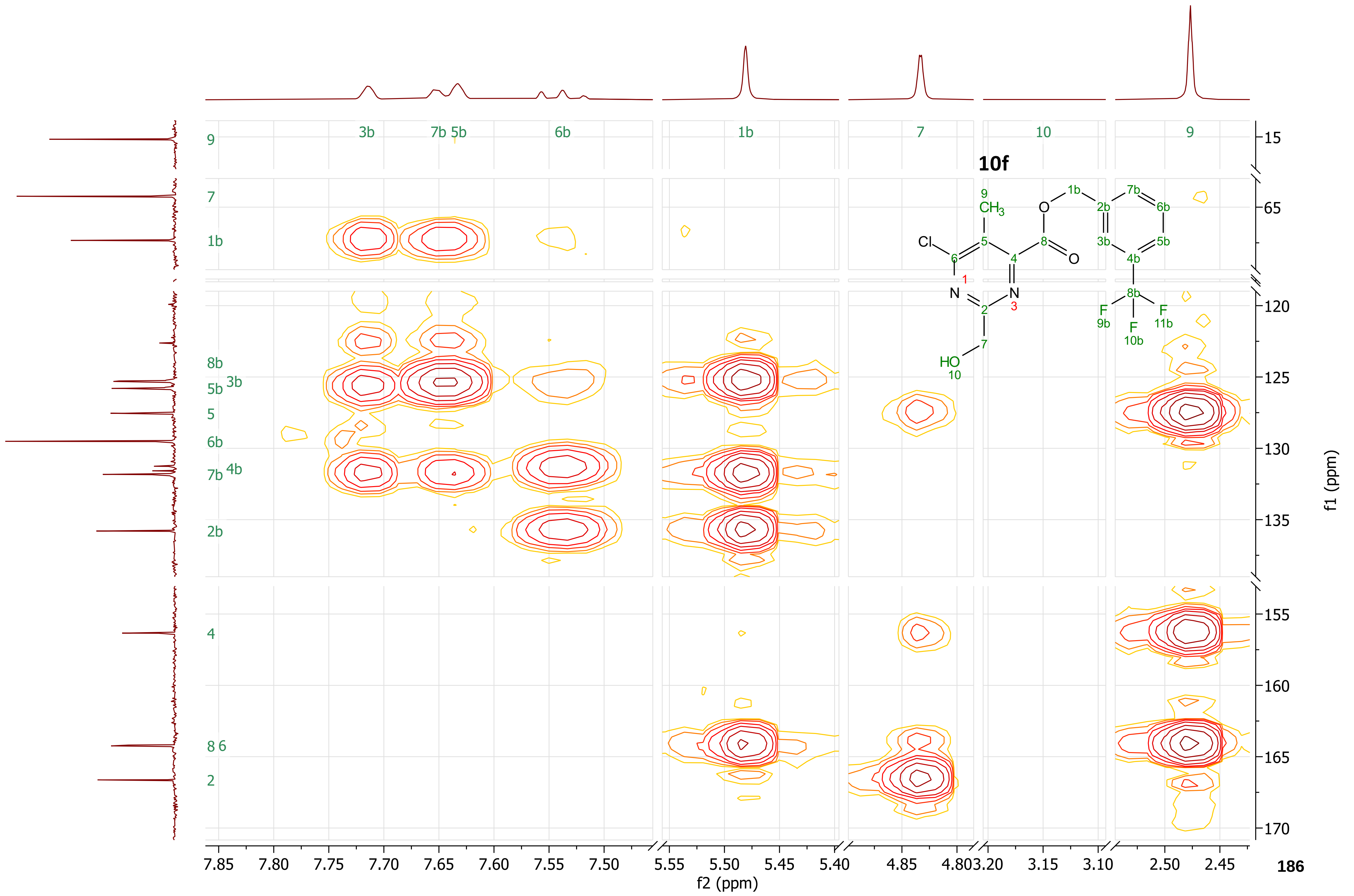
10f
f2 (ppm)
f1 (ppm)
3b
7b 5b
6b
1b
7
10
9
8b
5b 3b
5
6b
7b 4b
2b
4
8 6
2
Cl
CH3
O
O
N
N
HO
F
F
F

19F ^{19}F NMR (376 MHz, $CDCl_3$) δ -62.76.

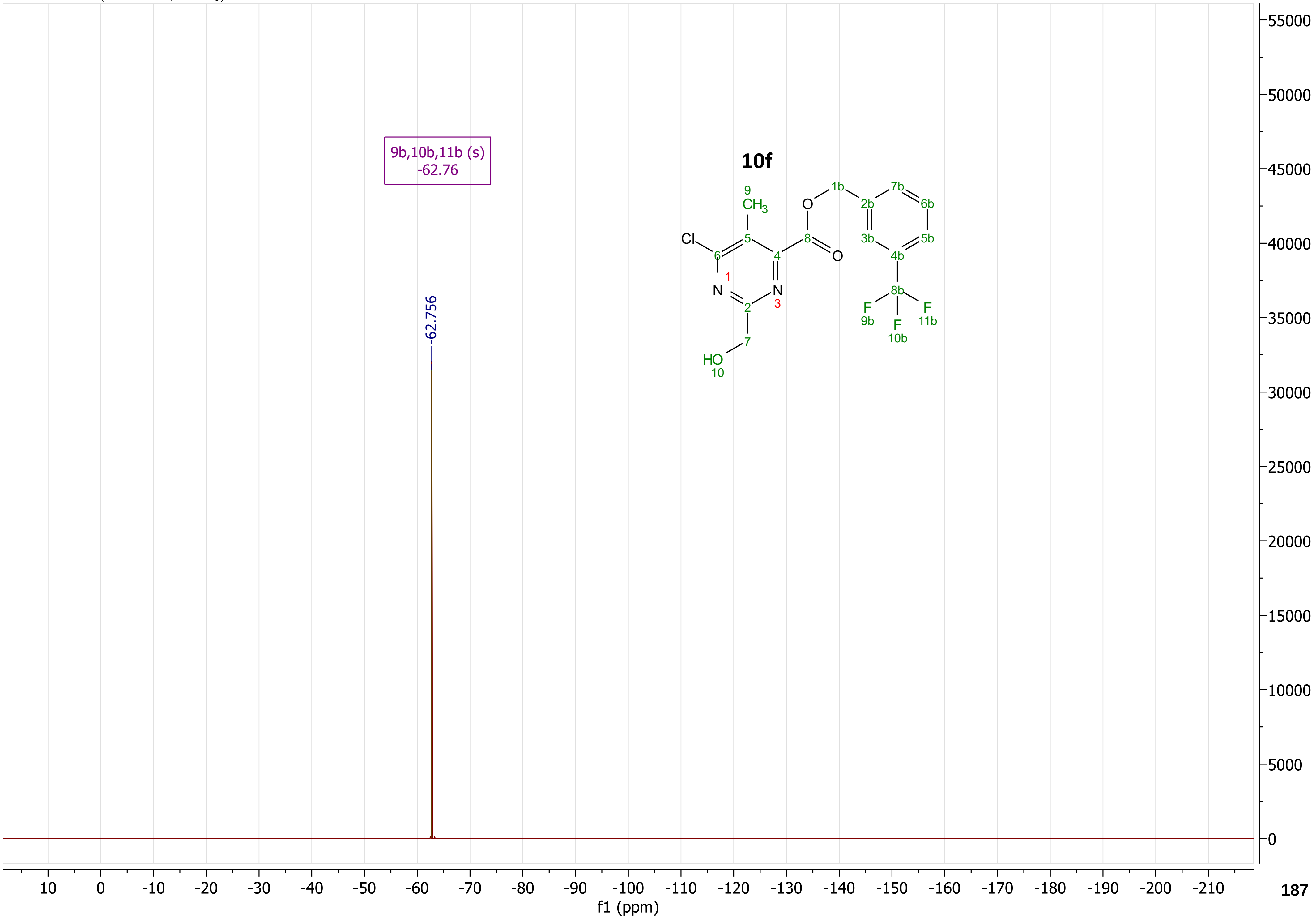

1H ^{1}H NMR (400 MHz, $CDCl_3$) δ 4.81 (app t, J = 0.9 Hz, 2H), 4.41 (t, J = 6.8 Hz, 2H), 2.86 (br s, 1H), 2.42 (app q, J = 0.8 Hz, 3H), 1.86 – 1.71 (m, 2H), 1.47 – 1.38 (m, 2H), 1.38 – 1.21 (m, 8H), 0.87 (app t, 3H).

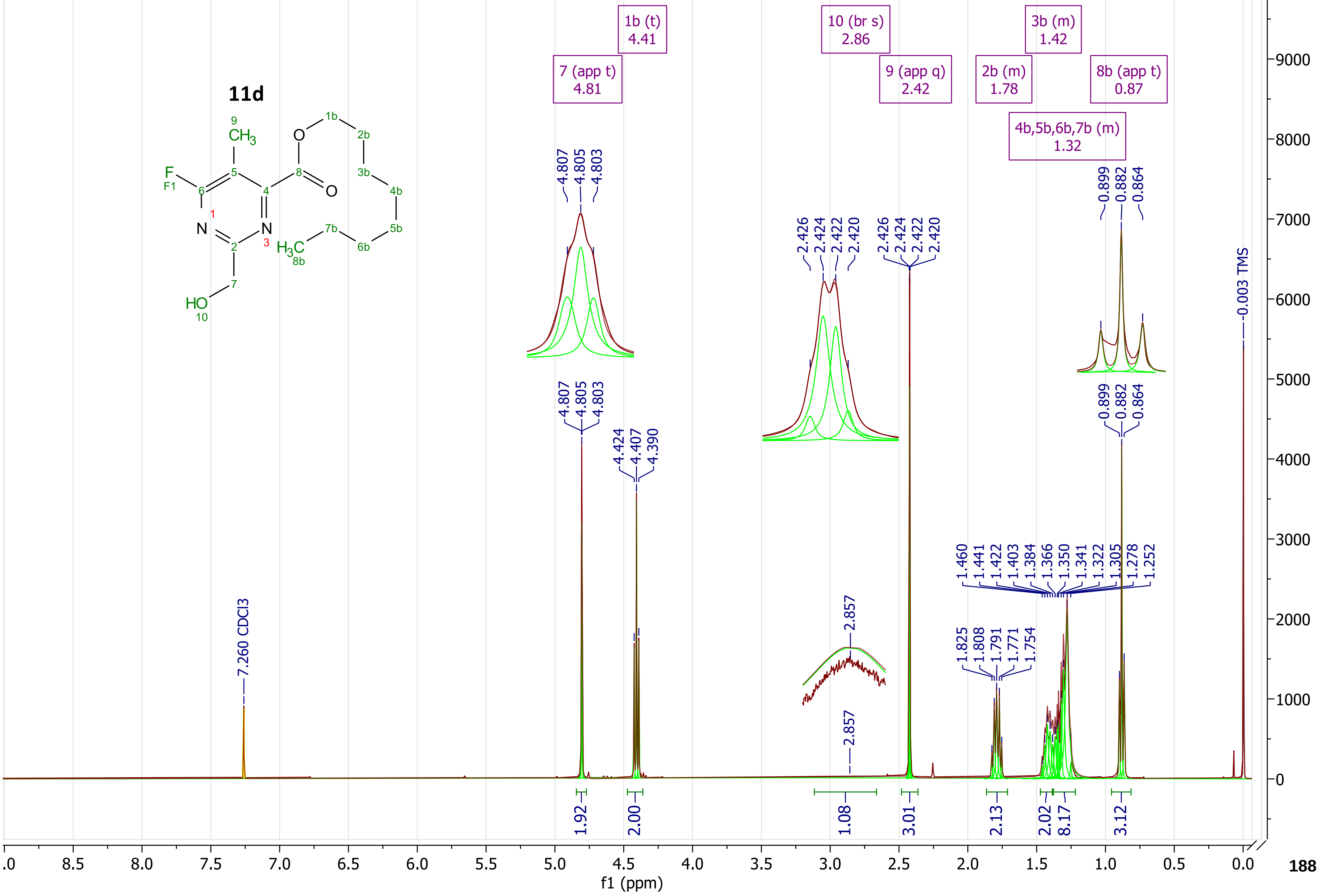

13C ^{13}C NMR (101 MHz, $CDCl_3$) δ 169.7 (d, J = 256.4 Hz), 167.0 (d, J = 13.3 Hz), 164.3 (d, J = 5.8 Hz), 158.9 (d, J = 5.9 Hz), 115.9 (d, J = 27.3 Hz), 67.0, 64.2, 31.9, 29.3 (2C), 28.6, 26.0, 22.8, 14.2, 10.4.

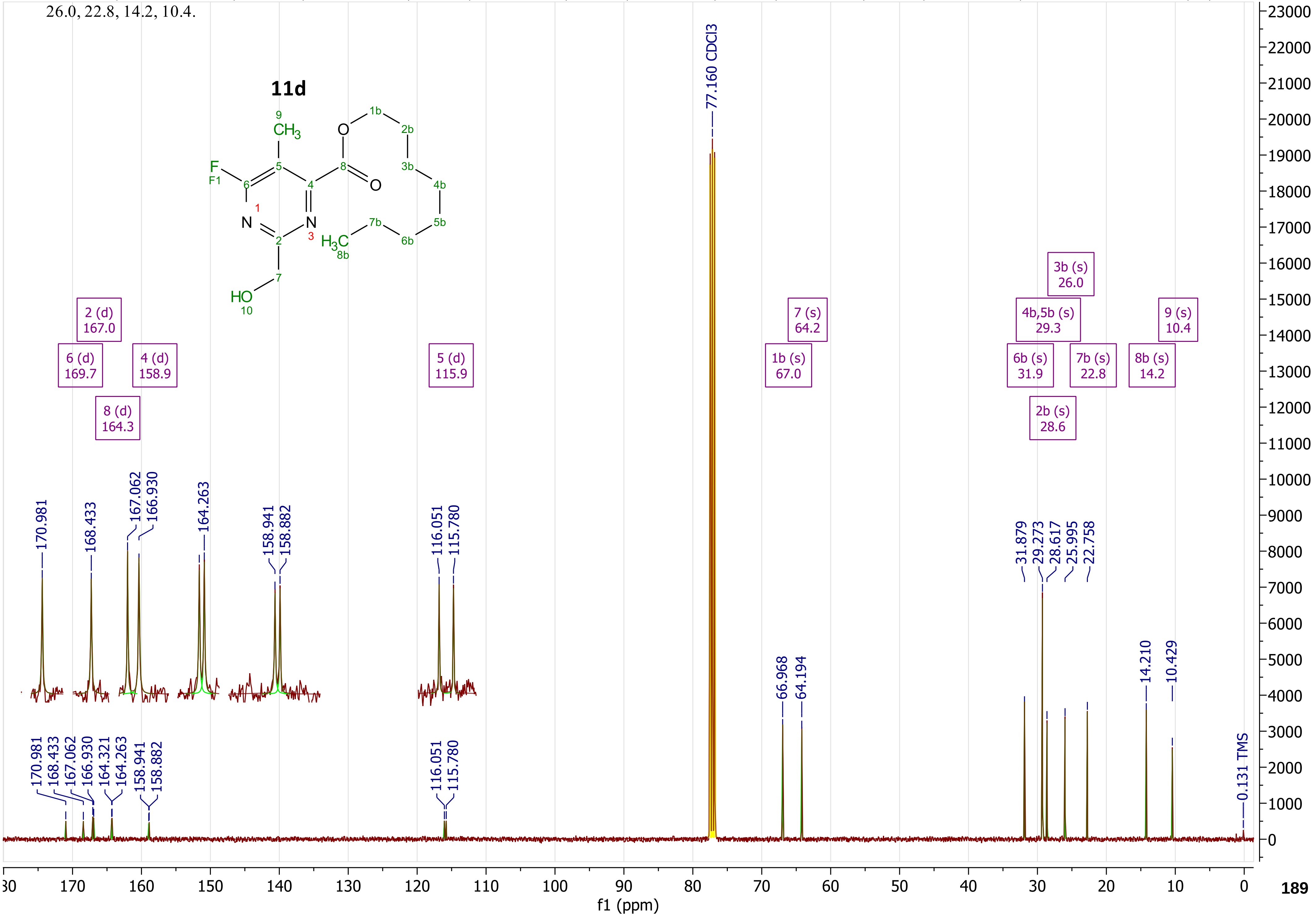

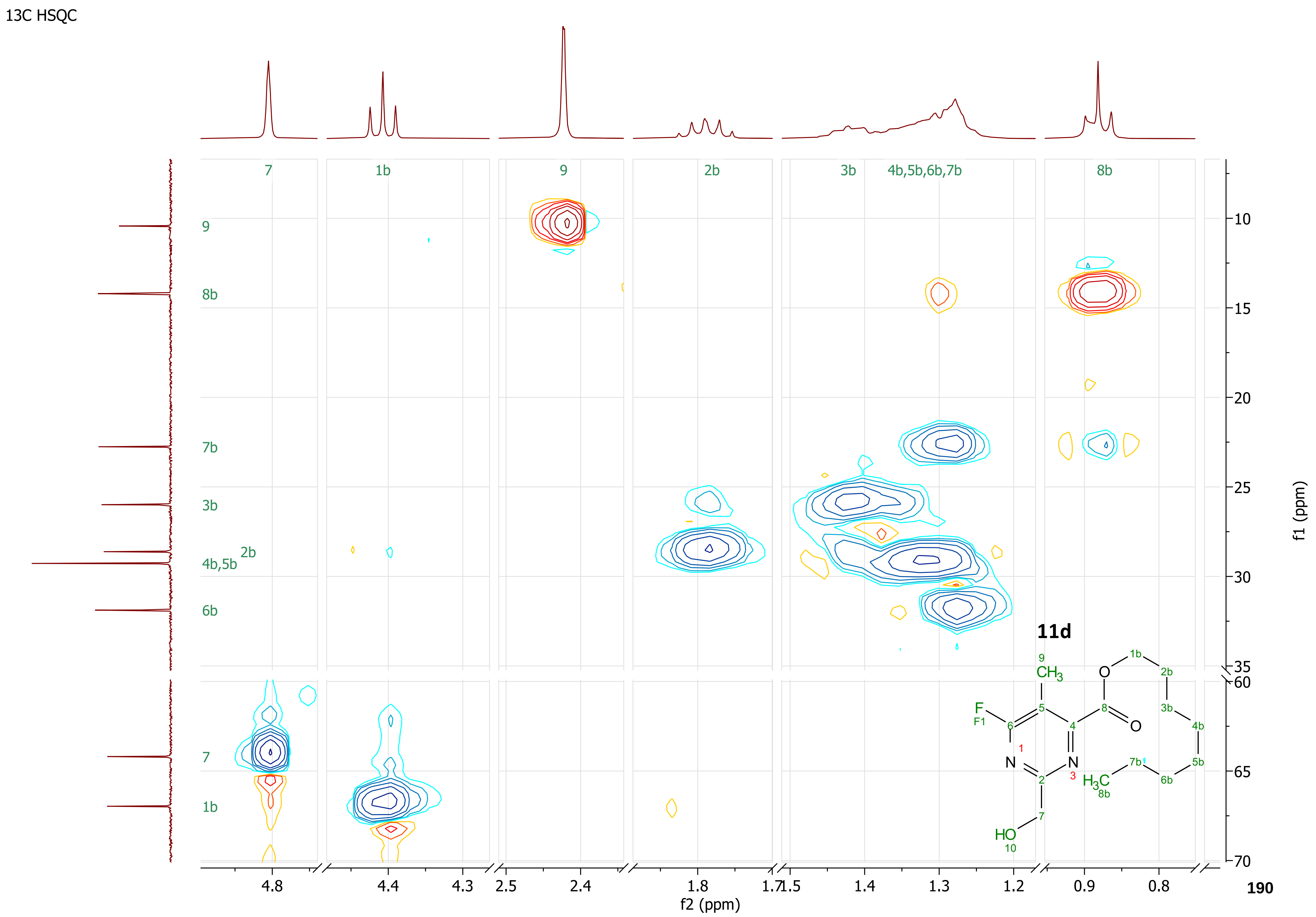
13C HSQC
7
1b
9
2b
3b
4b,5b,6b,7b
8b
9
8b
7b
3b
2b
4b,5b
6b
7
1b
f1 (ppm)
f2 (ppm)
11d

13C HMBC

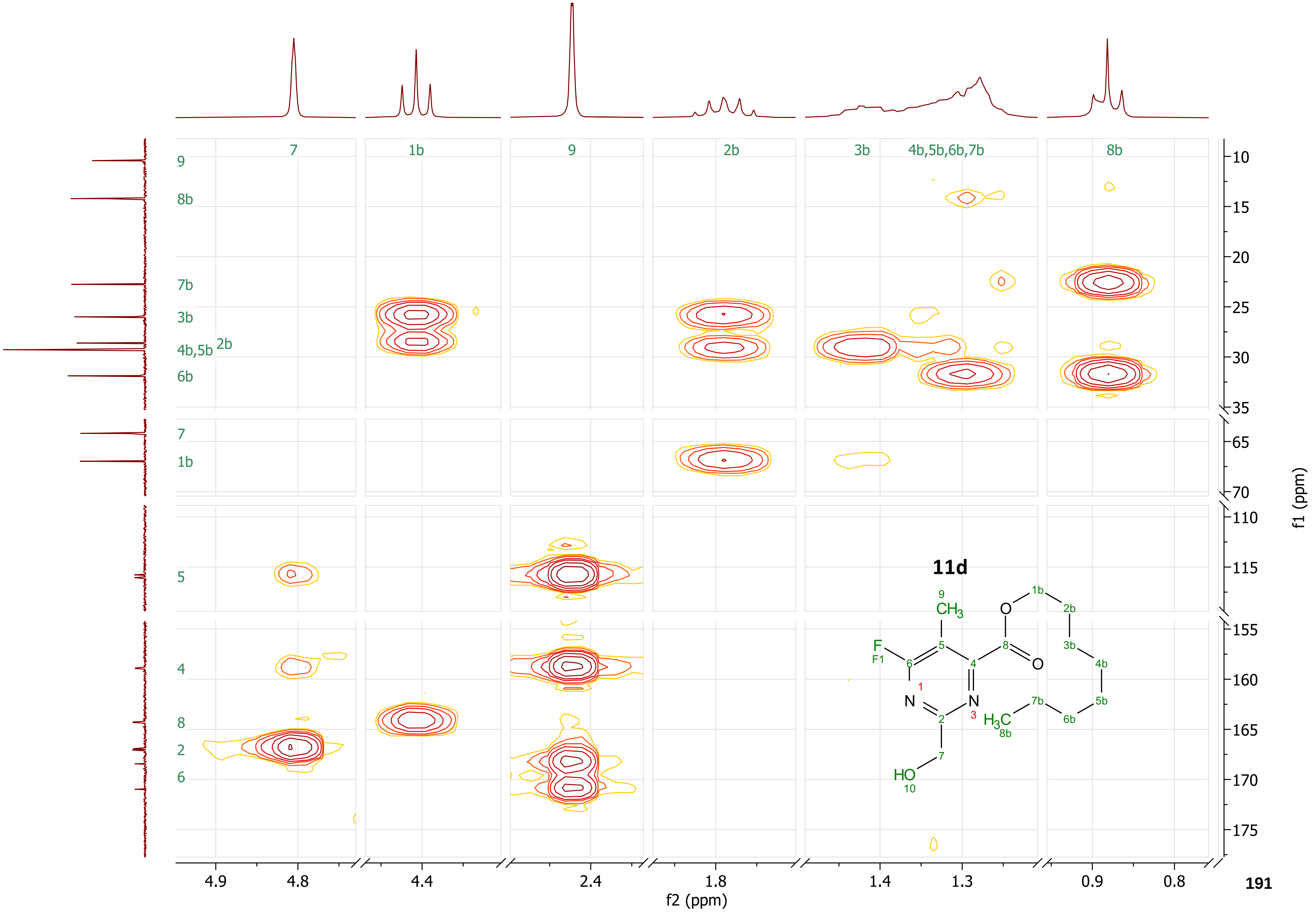
7
1b
9
2b
3b
4b,5b,6b,7b
8b
9
8b
7b
3b
4b,5b 2b
6b
7
1b
5
4
8
2
6
11d
f2 (ppm)
f1 (ppm)

19F ^{19}F NMR (376 MHz, $CDCl_3$) δ -61.28.

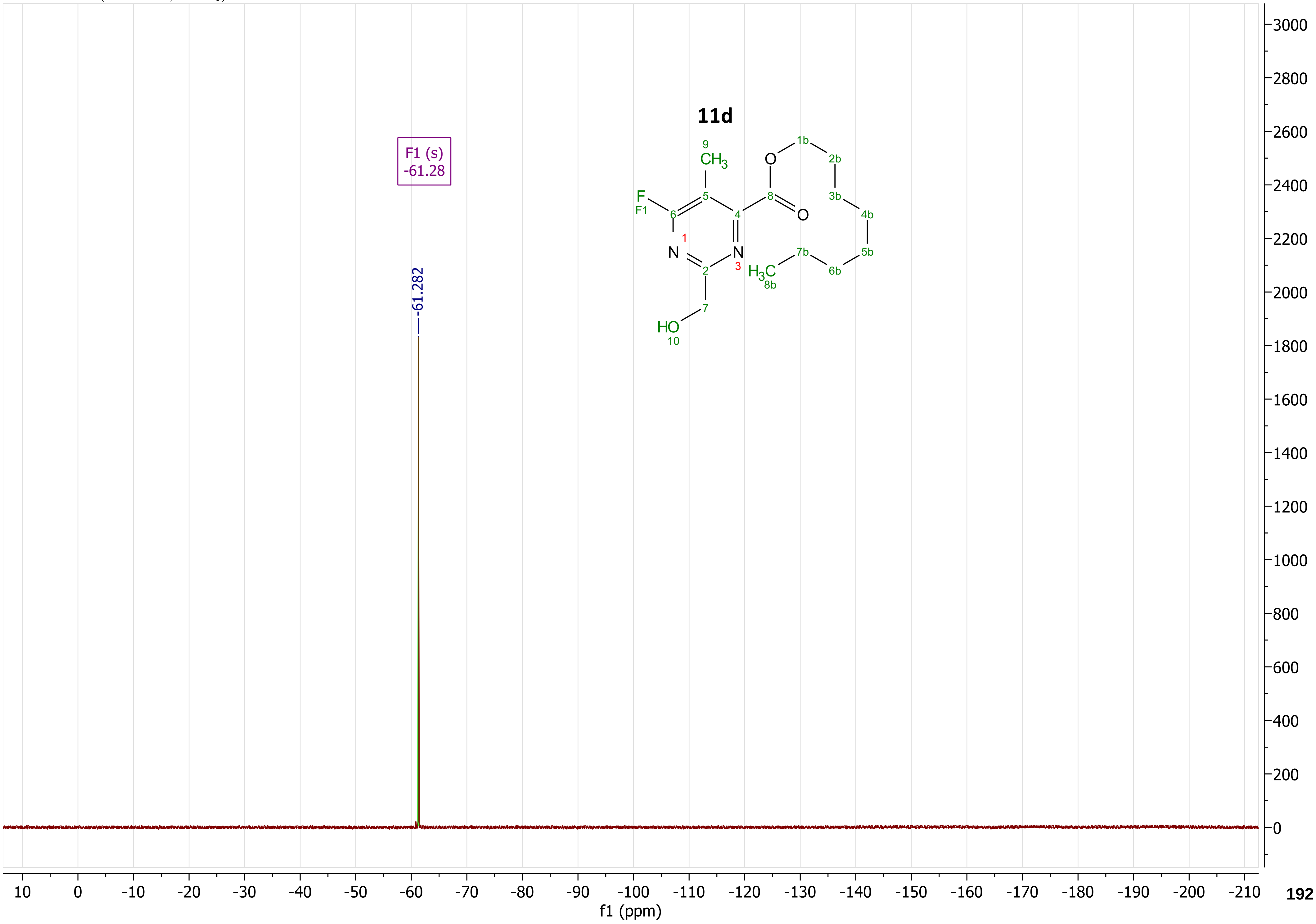

1H ^{1}H NMR (400 MHz, $CDCl_3$) δ 4.69 (app d, J = 0.8 Hz, 2H), 4.41 (t, J = 6.5 Hz, 2H), 4.38 (t, J = 6.8 Hz, 2H), 3.50 (br s, 1H), 2.27 (app t, J = 0.8 Hz, 3H), 1.86 – 1.68 (m, 4H), 1.55 – 1.37 (m, 4H), 0.98 (t, J = 7.4 Hz, 3H), 0.97 (t, J = 7.4 Hz, 3H).

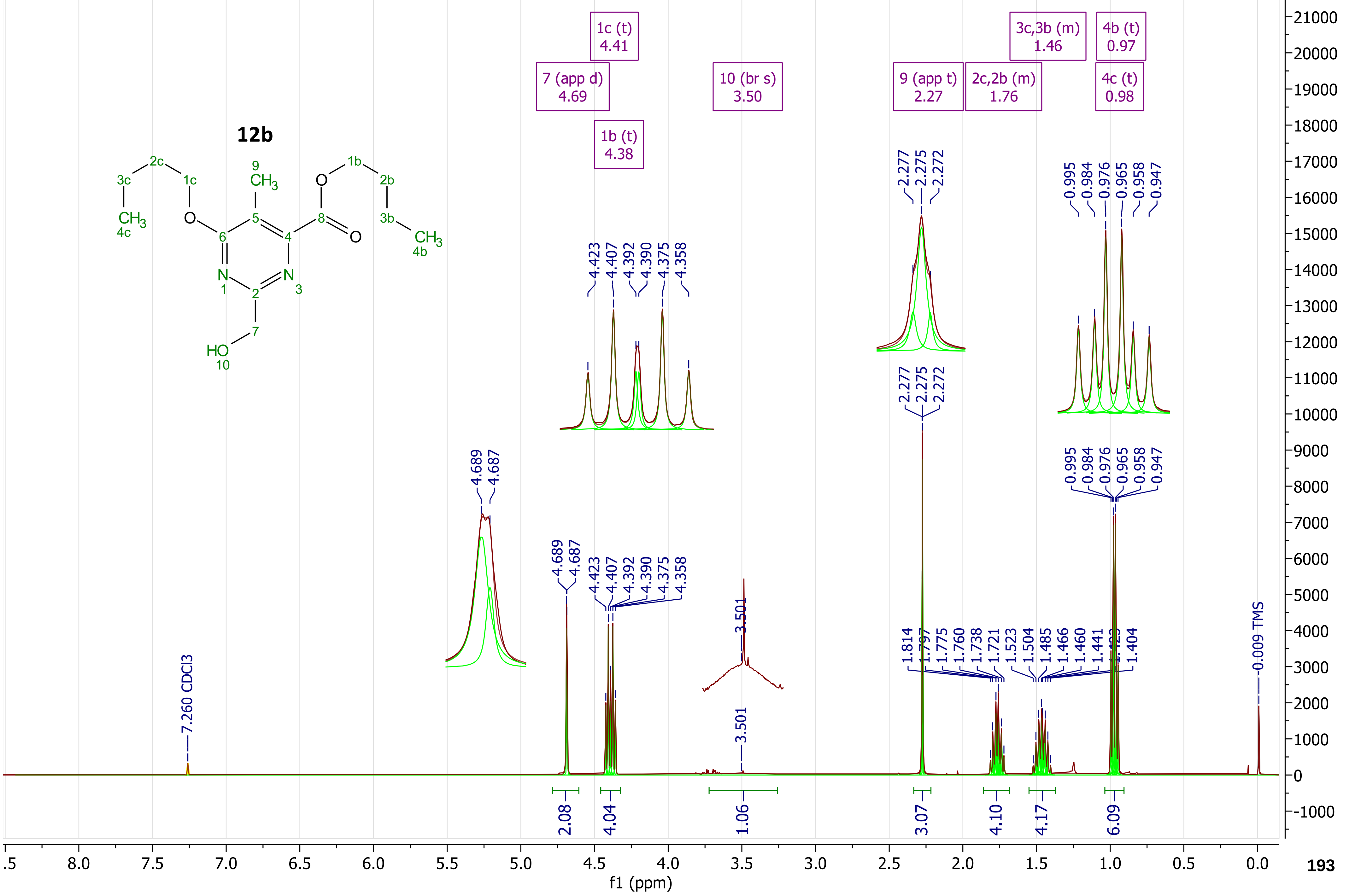

13C ^{13}C NMR (101 MHz, $CDCl_3$) δ 169.2, 165.8, 165.3, 154.5, 116.3, 67.4, 66.1, 64.2, 30.8, 30.7, 19.33, 19.29, 13.9, 13.8, 11.0.

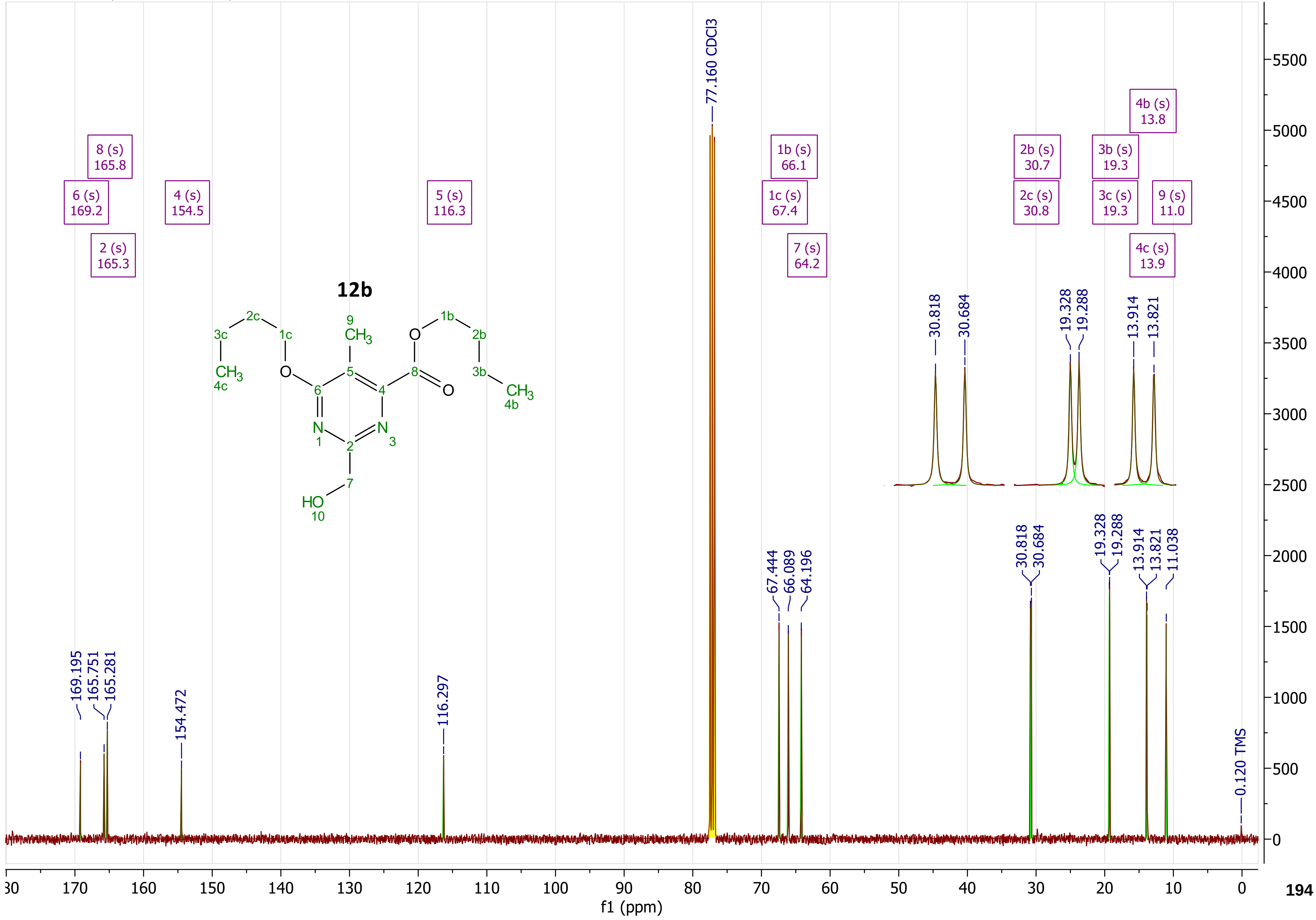

13C HSQC

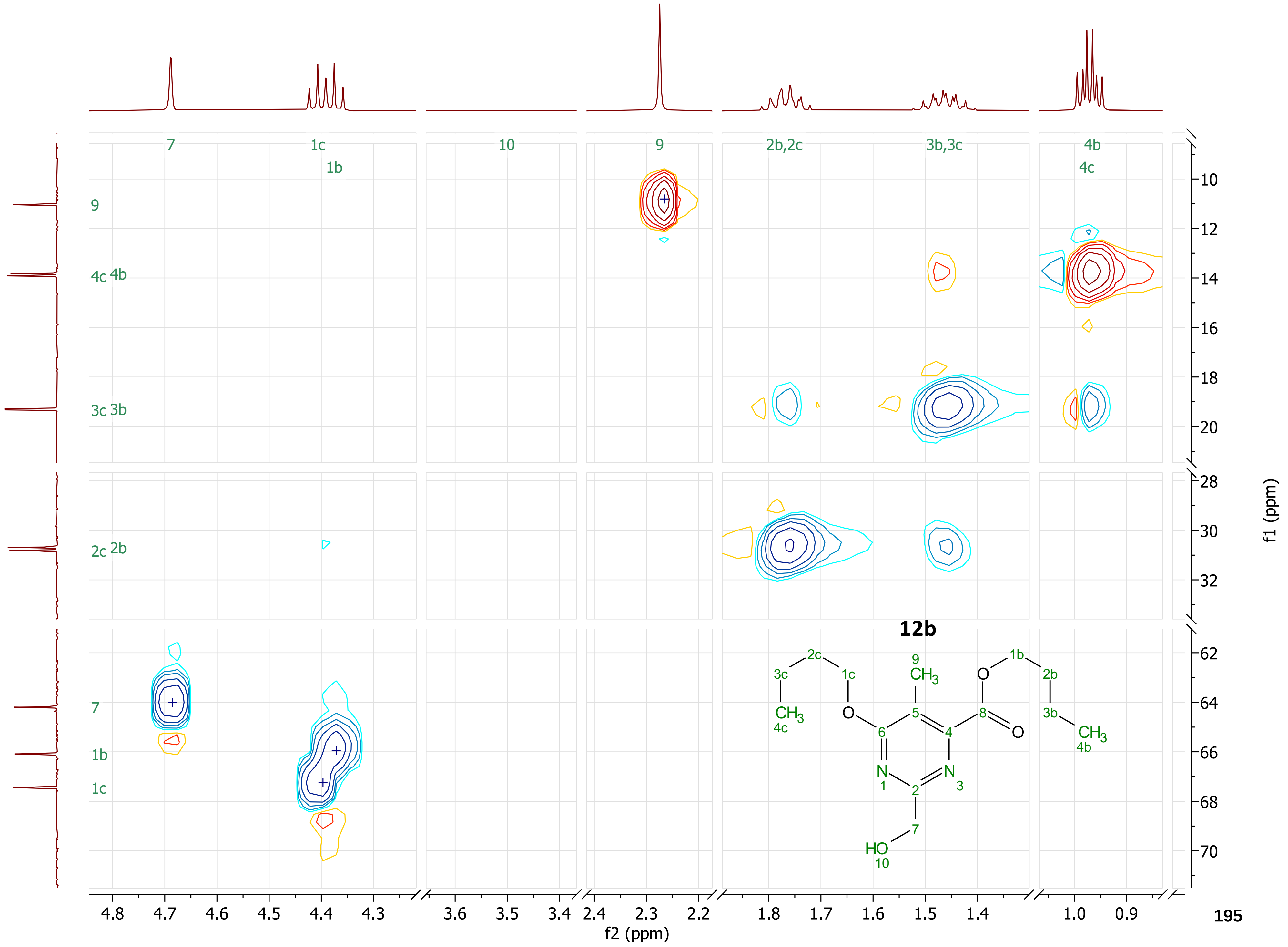

7
1c
1b
10
9
2b,2c
3b,3c
4b
4c
9
4c 4b
3c 3b
2c 2b
7
1b
1c
12b
f1 (ppm)
f2 (ppm)

13C HMBC

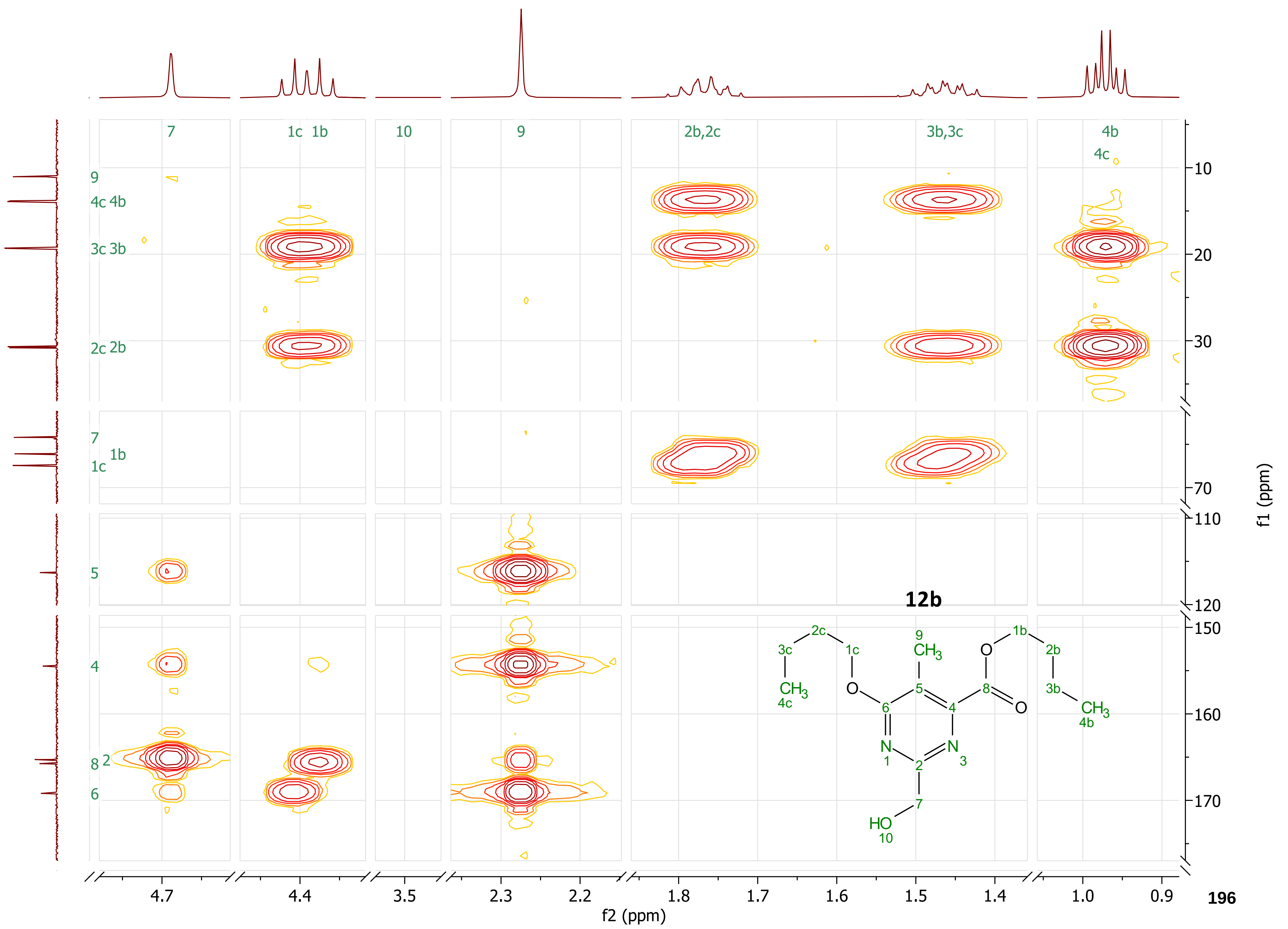

7
1c 1b
10
9
2b,2c
3b,3c
4b
4c
9
4c 4b
3c 3b
2c 2b
7
1b
1c
5
4
8 2
6
12b
f2 (ppm)
f1 (ppm)

15N HMBC

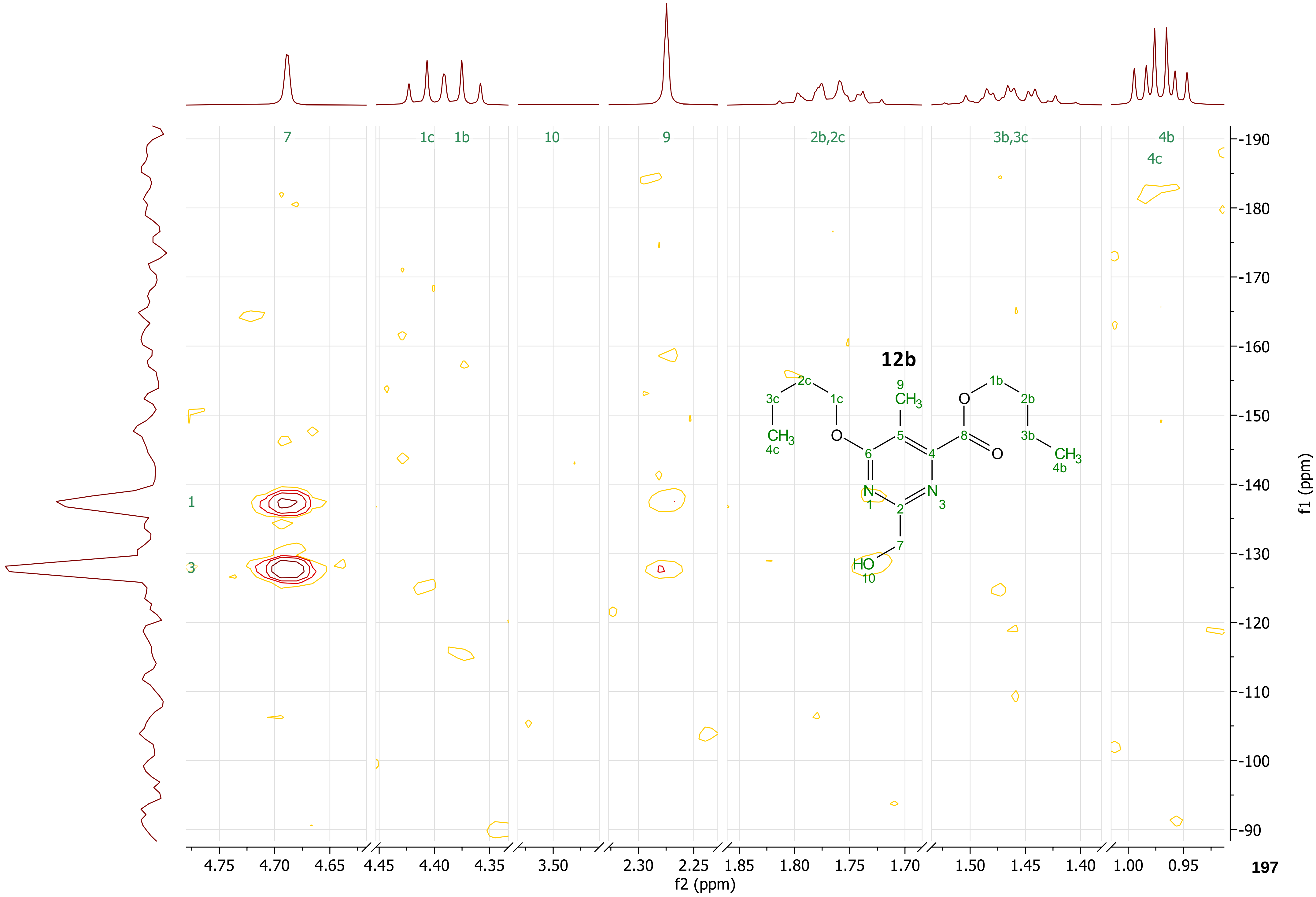

15N HMBC - f1 internal projection ^{15}N NMR (41 MHz, $CDCl_3$) δ -127.99, -137.56.

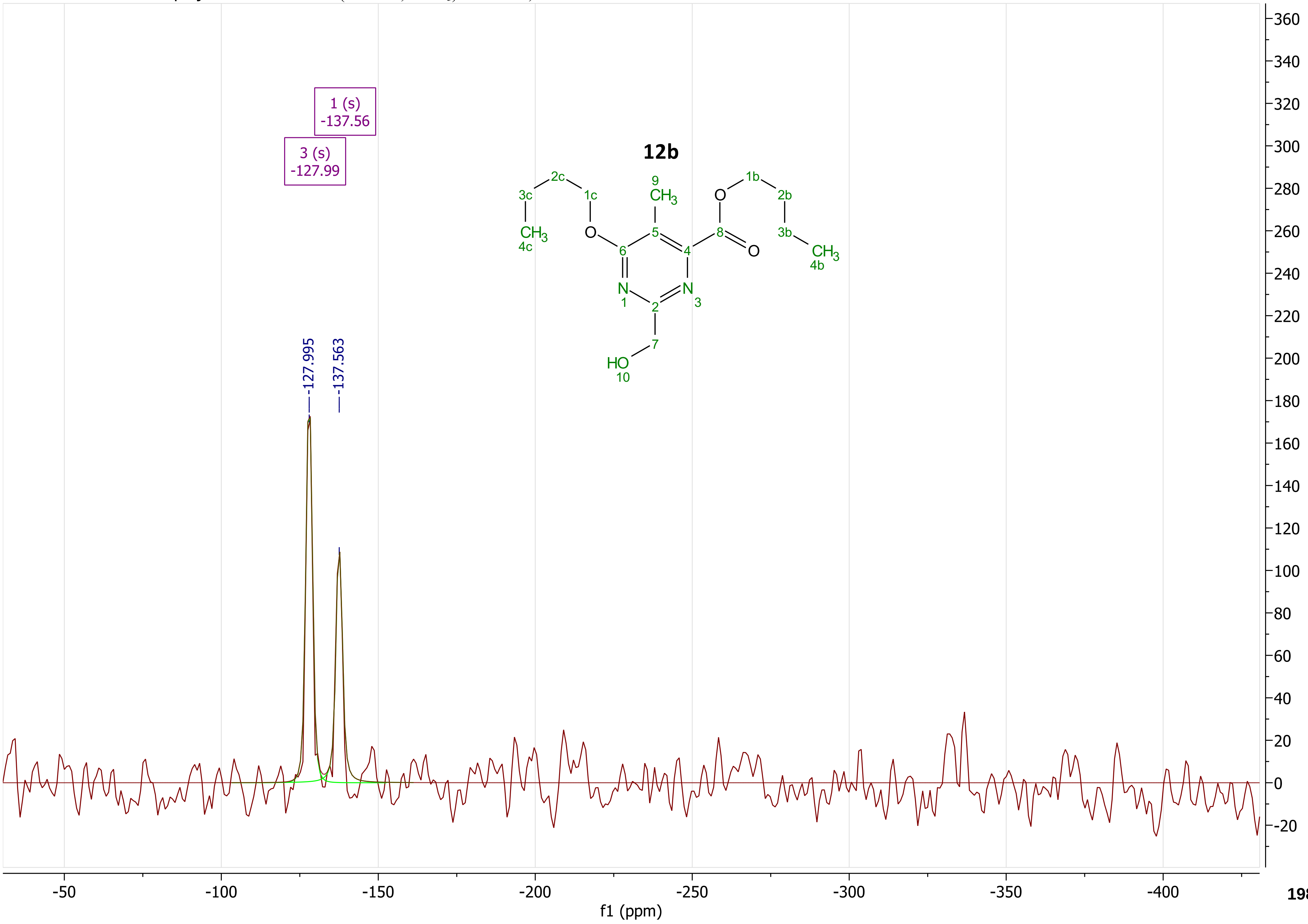

1H ^{1}H NMR (400 MHz, $CDCl_3$) δ 4.69 (s, 2H), 4.40 (t, J = 6.6 Hz, 2H), 4.37 (t, J = 6.8 Hz, 2H), 3.49 (br s, 1H), 2.28 (s, 3H), 1.85 – 1.71 (m, 4H), 1.50 – 1.38 (m, 4H), 1.37 – 1.22 (m, 16H), 0.885 (app t, J = 6.8 Hz, 3H), 0.877 (app t, J = 6.8 Hz, 3H).

12d

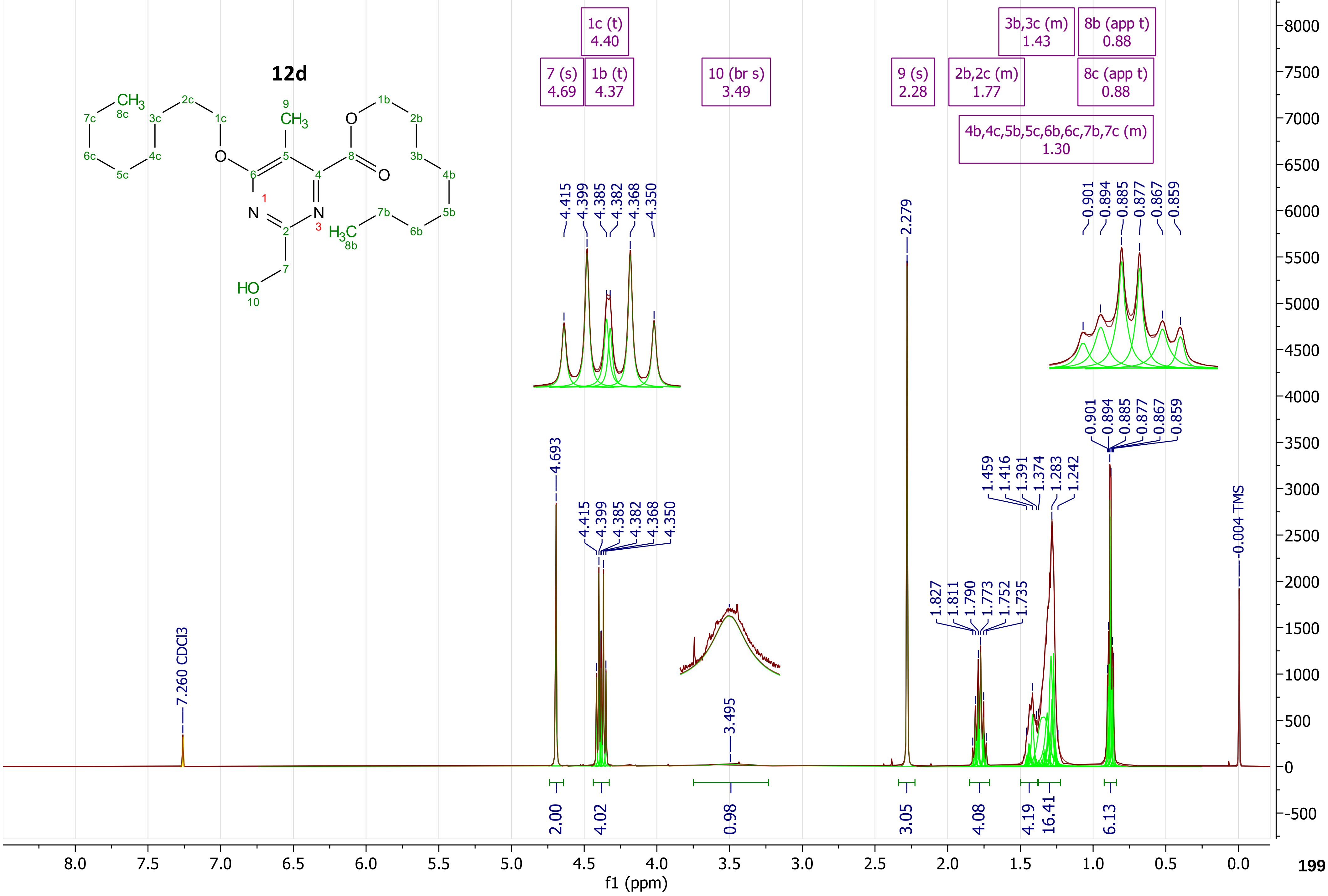

13C ^{13}C NMR (101 MHz, $CDCl_3$) δ 169.2, 165.7, 165.3, 154.5, 116.3, 67.8, 66.4, 64.2, 31.92, 31.89, 29.4, 29.34, 29.31, 29.29, 28.8, 28.7, 26.1, 26.0, 22.75, 22.67, 14.23, 14.22, 11.1.

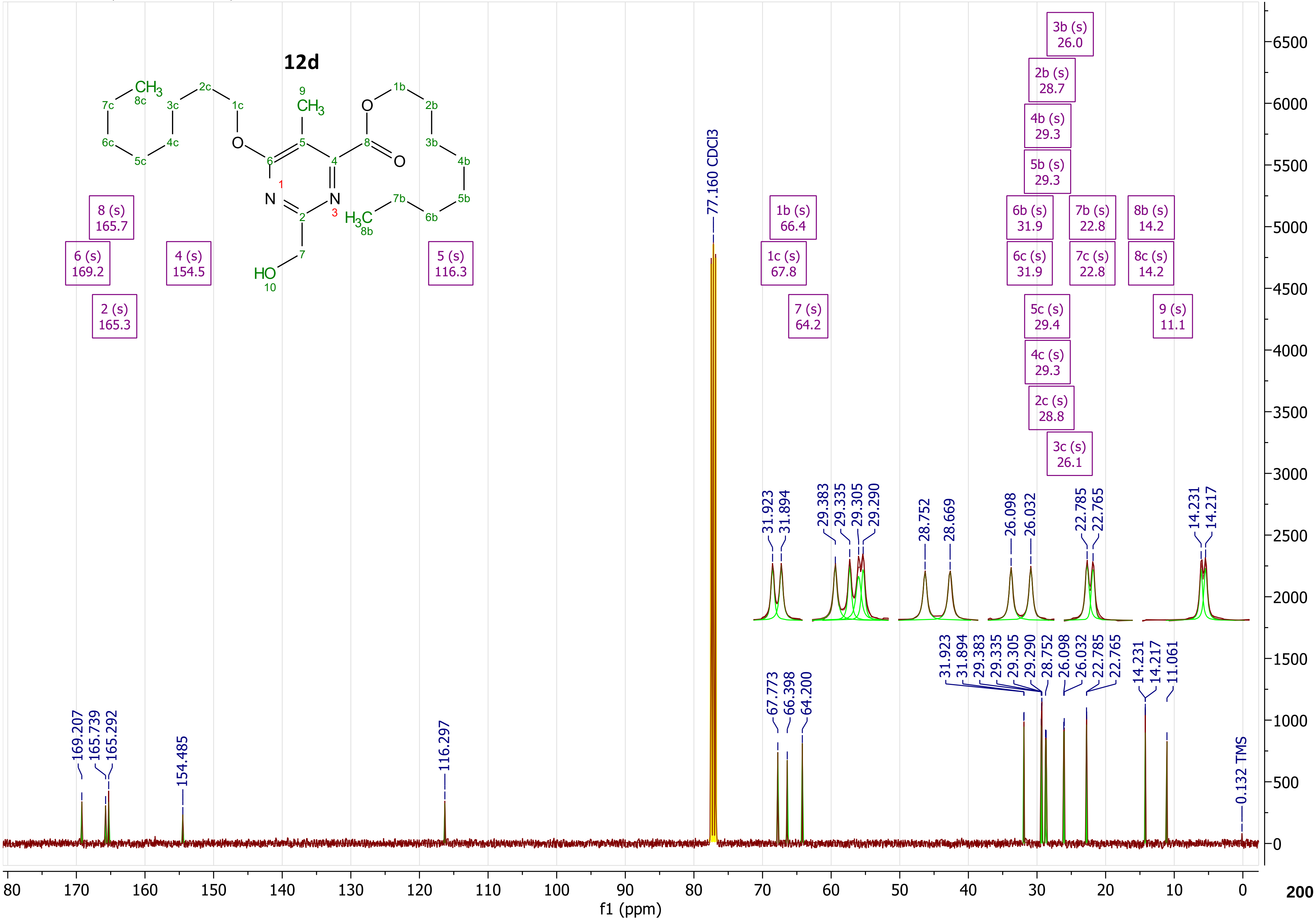

13C HSQC

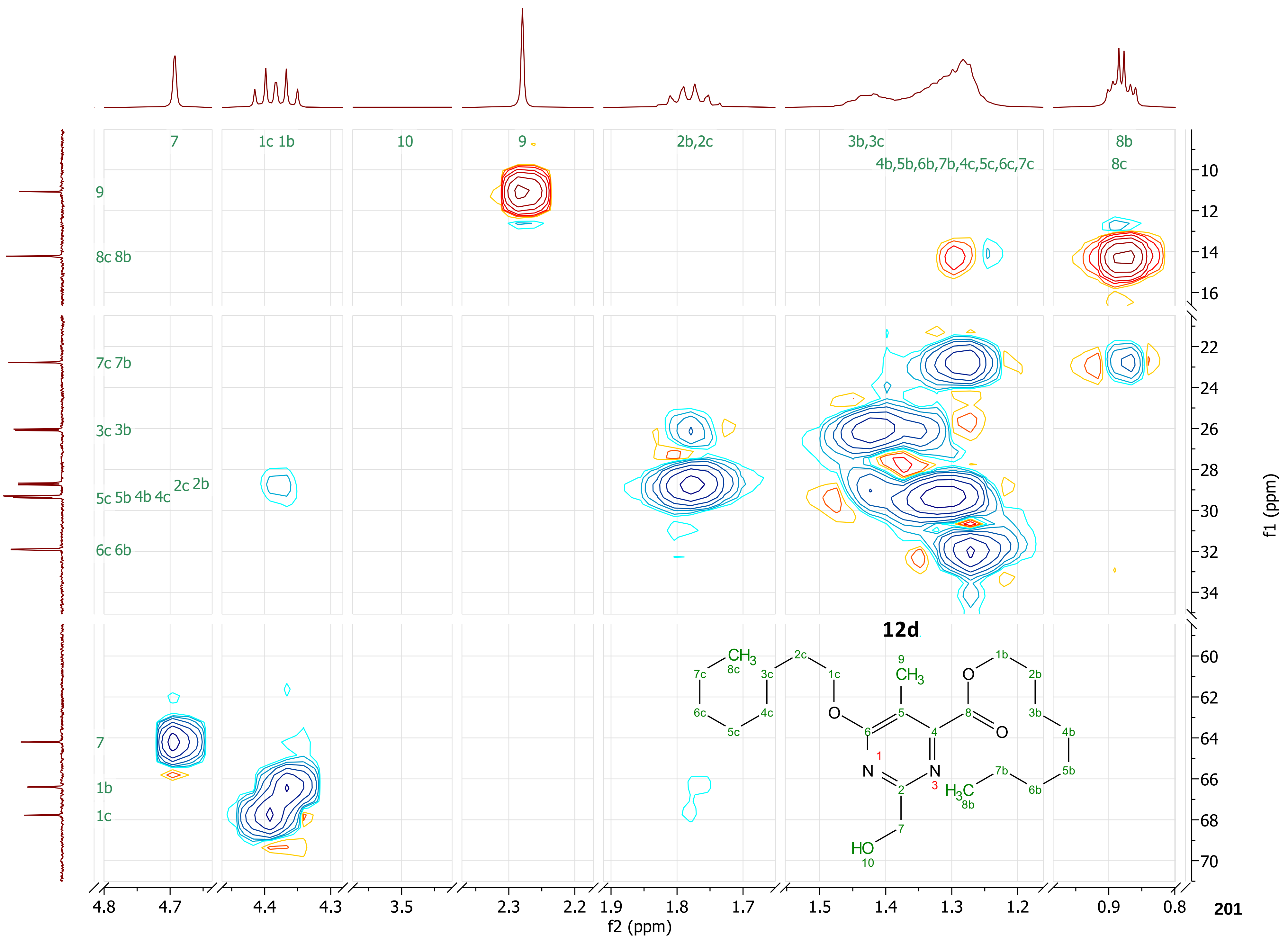

7
1c 1b
10
9
2b,2c
3b,3c
4b,5b,6b,7b,4c,5c,6c,7c
8b
8c
9
8c 8b
7c 7b
3c 3b
2c 2b
5c 5b 4b 4c
6c 6b
7
1b
1c
12d
f2 (ppm)
f1 (ppm)

13C HMBC

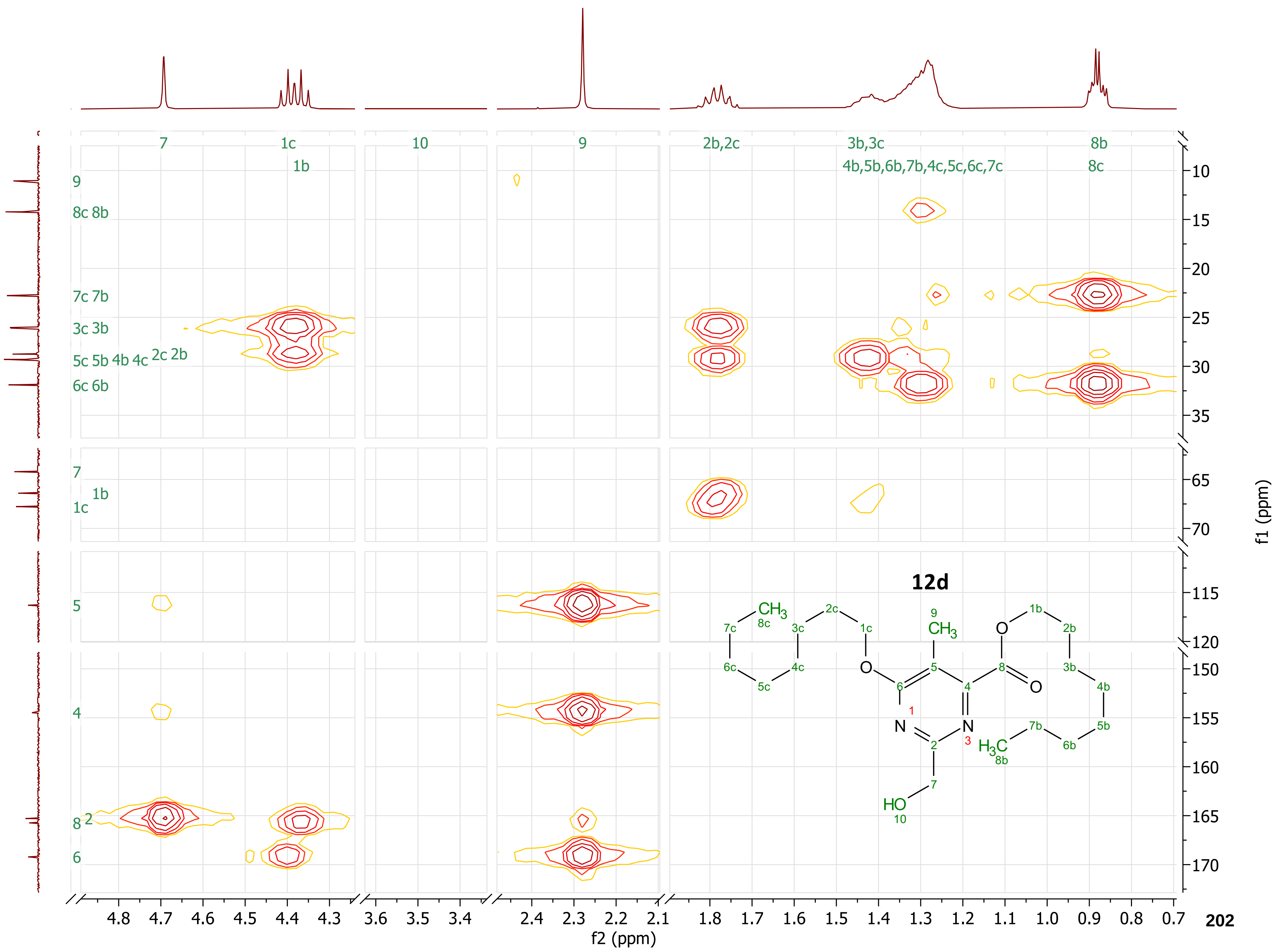
7
1c
1b
10
9
2b,2c
3b,3c
4b,5b,6b,7b,4c,5c,6c,7c
8b
8c
9
8c 8b
7c 7b
3c 3b
5c 5b 4b 4c 2c 2b
6c 6b
7
1b
1c
5
4
8 2
6
12d
f2 (ppm)
f1 (ppm)
